# Global lung cancer burden, trends, and projections from 2010 to 2050: A population-level severity framework integrating DALYs-per-case and mortality-to-incidence ratio

**DOI:** 10.1371/journal.pone.0354350

**Published:** 2026-07-23

**Authors:** Omar Freihat, Maria Aamir, Zsolt Cselik, Arpad Kovacs, David Sipos

**Affiliations:** 1 Department of Environmental and Public Health, College of Health Sciences, Abu Dhabi University, Abu Dhabi, UAE; 2 Radiation Oncology, Ferenc Csolnoky County Hospital, Veszprem, Hungary; 3 Department of Oncoradiology, Faculty of Medicine, University of Debrecen, Debrecen, Hungary; 4 Department of Medical Imaging, Faculty of Health Sciences, University of Pécs, Pécs, Hungary; 5 Dr. József Baka Diagnostic, Radiation Oncology, Research and Teaching Center, “Moritz Kaposi” Teaching Hospital, Kaposvár, Hungary; Taichung Veterans General Hospital, TAIWAN

## Abstract

**Background:**

Lung cancer remains the leading cause of cancer mortality worldwide. While incidence and mortality describe population burden, they do not fully capture disease severity at the level of the individual patient. This study quantified the contemporary global burden, temporal trends, and future trajectory of tracheal, bronchus, and lung (TBL) cancer, and applied a multidimensional severity framework integrating fatal and non-fatal outcomes.

**Methods:**

A global ecological analysis was conducted across 185 countries and territories using harmonized data from GLOBOCAN 2022, the Global Burden of Disease (GBD) 2023 study, and the UN World Population Prospects 2024. Contemporary burden was assessed using incident cases, deaths, age-standardized incidence and mortality rates (ASIR/ASMR), disability-adjusted life years (DALYs), mortality-to-incidence ratio (MIR), and DALYs per incident case. Temporal trends in ASIR and ASMR (2010–2023) were estimated using log-linear regression to derive average annual percent change (AAPC). Projections to 2050 were generated under two scenarios: (1) constant 2022 rates reflecting demographic change only, and (2) trend-based projections extrapolating country-specific AAPCs. Disease severity was evaluated using a two-dimensional framework combining MIR and DALYs-per-case, which captures average lifetime health loss per diagnosed individual independent of population size or age structure.

**Results:**

In 2022, an estimated 2.48 million new TBL cancer cases and 1.82 million deaths occurred globally, corresponding to ASIR and ASMR of 23.6 and 16.8 per 100,000, respectively. Global DALYs reached 46.7 million, yielding 18.8 DALYs per case, with substantially higher burden in males and in higher-SDI regions. From 2010-2023, age-standardized rates increased in most low- and middle-income countries and declined predominantly in high-income settings. By 2050, demographic change alone is projected to drive large increases in burden in many low-resource countries, with further amplification under trend-based scenarios. DALYs-per-case showed a moderate positive correlation with MIR (Spearman’s r_s = 0.46), revealing marked heterogeneity in severity profiles across countries.

**Conclusions:**

Global lung cancer burden is projected to rise substantially in many regions despite declining rates in high-income settings. Integrating DALYs-per-case with MIR provides a complementary severity lens that highlights inequities not captured by incidence and mortality alone and supports more targeted prevention, treatment, and survivorship strategies.

## Introduction

Lung cancer remains one of the most consequential diseases in global public health and continues to be the leading cause of cancer-related mortality worldwide. According to the most recent estimates from the Global Cancer Observatory, cancers of the trachea, bronchus, and lung account for the highest number of cancer deaths globally, exceeding those attributed to breast, colorectal, and prostate cancers combined [1]. Despite advances in tobacco control, screening, and systemic therapies, lung cancer mortality remains disproportionately high, reflecting aggressive tumour biology and the persistent predominance of late-stage diagnosis across many regions [2,3].

The global burden of lung cancer is enormous and multifaceted, encompassing millions of new diagnoses and deaths each year and contributing substantially to overall cancer-related health loss. Contemporary analyses from the Global Burden of Disease (GBD) Study consistently identify lung cancer as one of the largest contributors to cancer-attributable disability-adjusted life years (DALYs) worldwide [4–6]. These DALYs reflect the combined effects of premature mortality and prolonged morbidity associated with disease progression and treatment, underscoring lung cancer’s outsized impact on individuals, health systems, and societies [7,8]. While total disability-adjusted life years (DALYs) provide a useful summary of population-level health loss, they do not capture how severely lung cancer affects the average diagnosed individual, an issue specifically addressed in this study [9].

Crucially, lung cancer burden is marked by pronounced geographic and socioeconomic inequalities. High-income regions continue to experience the highest age-standardized incidence and mortality rates, largely reflecting historical tobacco exposure and population aging [10,11]. In contrast, many low- and middle-income countries particularly in South Asia, sub-Saharan Africa, and parts of the Middle East are undergoing rapid epidemiological transitions, with rising incidence and mortality often accompanied by poorer outcomes [12,13]. These disparities are driven by heterogeneity in risk factor exposure, stage at diagnosis, access to effective treatment, and overall health system capacity, resulting in substantial variation in lung cancer outcomes across countries and regions [14,15].

Looking ahead, global population aging is expected to further amplify the lung cancer burden [16]. United Nations population projections indicate a substantial increase in the number and proportion of adults aged 60 years and older worldwide over the coming decades, particularly in low- and middle-income regions [17]. Because lung cancer incidence rises steeply with age, demographic aging alone is projected to drive major increases in absolute case numbers and deaths by 2050, even in settings where age-specific rates stabilize or decline [10,18]. Together, these trends indicate that lung cancer will remain a dominant contributor to global cancer burden well into the mid-21st century.

Although lung cancer burden has been extensively quantified, current epidemiological metrics are limited in their ability to capture disease severity at the level of the individual patient [19,20]. Incidence and mortality counts describe the absolute volume of disease, while age-standardized rates enable comparisons across populations with differing demographic structures. The mortality-to-incidence ratio (MIR) is widely used as a proxy for survival and health system performance [21,22] and DALYs summarize the total population-level health loss attributable to disease [23].

However, these measures are inherently population-centred and do not reflect the severity experienced by each affected individual [24,25]. Countries may report similar total DALYs from lung cancer while differing substantially in survival duration, stage at diagnosis, treatment intensity, and disability burden among patients. Likewise, MIR values may appear comparable across settings, even when patient-level morbidity and long-term health loss differ markedly. As a result, current metrics quantify population burden but do not capture how much health loss is experienced on average per incident case, revealing an important and underexplored gap in global cancer epidemiology.[24–27].

To address this gap, we introduce DALYs per incident case (DALYs-per-case) as a novel metric of lung cancer severity. DALYs-per-case is defined as the total DALYs attributable to lung cancer divided by the number of incident cases, capturing the average lifetime health loss associated with each new diagnosis. This measure integrates both fatal and non-fatal components of disease burden and therefore reflects the combined impact of premature mortality and disability at the patient level. By shifting analytical focus from aggregate population burden to case-level severity, DALYs-per-case provides information not conveyed by incidence, mortality, MIR, or total DALYs alone. Its limited application in prior global cancer research reflects the historical separation of incidence and DALY analyses rather than a lack of conceptual relevance. Because DALYs-per-case distributions exhibit substantial right skewness, log-transformation is applied to stabilize variance and facilitate meaningful cross-country comparison.

Conceptually, DALYs-per-case represents a bridge between mortality-based severity (captured by MIR) and disability-based burden (embedded within DALYs), enabling a multidimensional assessment of lung cancer severity across health systems. Building on this concept, we propose a two-dimensional severity framework that integrates DALYs-per-case with MIR. Within this framework, MIR captures survival-related severity, while DALYs-per-case reflects the average disability and fatal burden experienced per diagnosed patient. Their joint consideration yields four interpretable severity profiles that facilitate cross-country comparisons and identify settings with particularly adverse lung cancer outcomes.

When combined with projections to 2050, this framework further provides insight into how current severity patterns may shape future lung cancer burden and health system demand. Accordingly, the objectives of this study were to quantify the contemporary global burden of lung cancer, characterize long-term temporal trends, project future burden under alternative scenarios, and introduce a novel severity framework based on DALYs-per-case and MIR to enhance understanding of patient-level lung cancer severity worldwide.

## Methods

### Study design and case definition

This study employed a global ecological design to quantify the burden, temporal evolution, and future trajectory of tracheal, bronchus, and lung cancer, defined according to the International Classification of Diseases, 10th Revision (ICD-10) codes C33-C34. The analysis had three prespecified objectives: (1) to characterise the contemporary burden in 2022–2023, (2) to estimate long-term temporal trends in age-standardised incidence and mortality rates from 2010 to 2023, and (3) to project the burden to 2050 under two contrasting modelling assumptions. The analytic dataset included 204 countries and territories and was stratified by sex (male and female), and aggregated according to Global Burden of Disease (GBD) super-regions and Sociodemographic Index (SDI) quintiles.

### Data sources

Three publicly available data systems were harmonised. First, GLOBOCAN 2022 provided country-specific estimates of incident cases, deaths, and age-standardised incidence and mortality rates (ASIR and ASMR, respectively) for the year 2022 using the Segi-Doll world standard population [1]. Second, the GBD 2023 study contributed annual national estimates for 2010–2023 of incidence, mortality, and disability-adjusted life years (DALYs), including absolute counts, age-standardised rates based on the GBD world population standard, and 95% uncertainty intervals (UIs) [28,29]. Third, the United Nations World Population Prospects (WPP) 2024 Revision provided mid-year population estimates for 2022 and projections to 2050 (medium-fertility variant) by country, sex, year, and five-year age group [17]. Countries lacking complete data across any of the sources were excluded from trend or projection analyses.

### Data processing and linkage

Country names were standardized through ISO-3166–1 alpha-3 codes, supplemented by manual reconciliation for nomenclature inconsistencies. All age-standardized rates from GLOBOCAN and GBD were converted to a uniform denominator of per 100,000 person-years using the GBD world standard population for internal consistency. A master dataset was constructed at the country-sex-year level covering 2010–2050 through linkage and integration of incidence, mortality, DALYs, and population denominator datasets. Regional and SDI summaries adhered to the official GBD classification.

### Estimation of contemporary burden and derived indicators

Global totals for incident cases and deaths in 2022 were obtained by summing GLOBOCAN country-level counts. The global ASIR and ASMR were calculated as population-weighted averages of country-specific age-standardised rates. DALY estimates corresponded to the most recent GBD year (2023) and were presented as absolute counts and age-standardised rates with 95% UIs.

Two derived indicators were calculated using standard definitions:

#### Mortality-to-Incidence Ratio (MIR), [22,30].


$$\begin{array}{c} \text{MIR}=\frac{\text{Total deaths}}{\text{Total incident cases}}. \end{array}$$

No additional modelling was applied to GBD 2023 age-standardised summary rates for super-regions and SDI quintiles; these values were extracted directly.

#### Temporal Trend Analysis (2010–2023).

Temporal trends in ASIR and ASMR were estimated using joinpoint-free log-linear regression, following established epidemiological practice [31,32]. For each country-sex stratum, the model


$$\begin{array}{c} \text{ln}\left(r_{t}\right)=\alpha +\beta t, \end{array}$$

where $r_{t}$ is the age-standardised rate in year t, was fitted to observed GBD 2010–2023 data. The Average Annual Percent Change (AAPC) was computed as [32]:


$$\begin{array}{c} \text{AAPC}=\left(e^{\beta }-\text{1}\right)\times \text{1}\text{0}\text{0}\%. \end{array}$$

Positive AAPC values denote increasing temporal trends; negative values indicate declining trends. Strata with fewer than four available years were excluded, and isolated missing years were linearly interpolated to maintain continuity.

### Projection methodology to 2050

Projections of incidence and mortality were generated for each country, sex, and year from 2023-2050 under two scenarios, implemented under two distinct scenarios. All projections used WPP 2024 population denominators.

#### Scenario 1: Constant-rate demographic projection.

Scenario 1 assumes that age-specific incidence and mortality rates remain fixed at their 2022 values; therefore, changes in absolute burden reflect demographic dynamics alone. For each year t:


$$\begin{array}{c} {\text{Cases}}_{t}^{\left(S\text{1}\right)}={\text{ASIR}}_{\text{2}\text{0}\text{2}\text{2}}\times \frac{P_{t}}{\text{1}\text{0}\text{0}\;\text{0}\text{0}\text{0}}, \end{array}$$

$$\begin{array}{c} {\text{Deaths}}_{t}^{\left(S\text{1}\right)}={\text{ASMR}}_{\text{2}\text{0}\text{2}\text{2}}\times \frac{P_{t}}{\text{1}\text{0}\text{0}\;\text{0}\text{0}\text{0}}, \end{array}$$

where $P_{t}$ denotes the projected population for year t.

#### Scenario 2: Trend-based dynamic projection.

Scenario 2 assumes a continuation of recent historical trends, operationalised by extrapolating age-standardised rates from 2022 forward according to country-sex specific AAPCs [33–35]:


$$\begin{array}{c} {\text{ASIR}}_{t}^{\left(S\text{2}\right)}={\text{ASIR}}_{\text{2}\text{0}\text{2}\text{2}}\times (\text{1}+{\text{AAPC}}_{\text{inc}}/\text{1}\text{0}\text{0}{)}^{t-\text{2}\text{0}\text{2}\text{2}}, \end{array}$$

$$\begin{array}{c} {\text{ASMR}}_{t}^{\left(S\text{2}\right)}={\text{ASMR}}_{\text{2}\text{0}\text{2}\text{2}}\times (\text{1}+{\text{AAPC}}_{\text{mort}}/\text{1}\text{0}\text{0}{)}^{t-\text{2}\text{0}\text{2}\text{2}}. \end{array}$$

Projected case and death counts were then computed via the same population-based formulas described for Scenario 1. To avoid unrealistic exponential rises, extrapolated rates were capped at 80 per 100 000 for incidence and 70 per 100 000 for mortality, following precedent from long-range cancer forecasting models [36,37]. All projections are reported as absolute numbers for 2050 and percentage changes relative to 2022.

### Severity assessment using DALYs-per-case framework

To complement conventional epidemiological indicators, disease severity was quantified using a DALYs-per-incident-case metric, following previously published methodological frameworks for cancer severity assessment [38]. DALYs-per-case represents the average disability-adjusted life years lost for each new incident case and captures the combined fatal and non-fatal burden attributable to tracheal, bronchus, and lung cancer. To ensure methodological consistency, DALYs-per-case was calculated using DALYs and incidence estimates derived exclusively from the GBD 2023 dataset. The formula used to calculate DALY per case [38]:


$$\begin{array}{c} DLAY-per-case=\;\frac{GDB\;\text{2}\text{0}\text{2}\text{3}\;DALYS}{GDB\;\text{2}\text{0}\text{2}\text{3}\;Incidence\;} \end{array}$$

A higher DALYs-per-case value denotes greater average severity, reflecting poorer survival outcomes, higher premature mortality, and prolonged disability. Unlike incidence or mortality rates, this metric is not a function of population size or age structure and therefore provides a complementary cross-sectional severity indicator [39,40].

To evaluate severity in a multidimensional framework, DALYs-per-case was compared with the mortality-to-incidence ratio (MIR), a widely used proxy for case fatality and system performance [38,41,42]. A two-dimensional scatter framework was constructed in which each country-sex stratum was plotted according to its DALYs-per-case (y-axis) and MIR (x-axis) as described by Freihat 2025 [38], this allowed classification into four interpretable quadrants:

High MIR/High DALYs-per-case: extremely severe settings with high fatality and large lifetime health loss per case; High MIR/Low DALYs-per-case: fatality-dominant burden with relatively lower disability contribution; Low MIR/High DALYs-per-case: substantial disability burden despite comparatively lower mortality; and Low MIR/Low DALYs-per-case: least severe profiles.

This 2-D severity visualisation has been validated in prior cancer burden research¹¹–¹³ and facilitates identification of countries where high fatality and high disability converge, thereby complementing incidence- and mortality-based analyses. Importantly, DALYs were not projected to 2050; DALYs-per-case was used solely as a baseline severity index to characterise the contemporary burden. To assess the relationship between MIR and DALYs-per-case, Spearman rank correlation was computed because both indicators are non-normally distributed and represent monotonic but non-linear severity gradients across countries. Extreme DALYs-per-case values (<5 or >60) were excluded from the severity framework due to instability arising from low incidence denominators. Moreover, to evaluate the robustness of the severity quadrant framework to threshold selection, a sensitivity analysis was performed by varying the global median thresholds used for quadrant classification. MIR thresholds were varied by ±5%, while DALYs-per-case thresholds were varied by ±10%, and country reassignment across quadrants was assessed under each alternative threshold scenario. These ranges were selected to examine the stability of the framework under modest variations in cut-point definition. Additionally, to evaluate whether DALY-per-case provides additional information beyond raw DALY and MIR, we compared quadrant classifications across three metrics. Countries were classified using each metric independently, and reclassification between metrics was assessed. Furthermore, uncertainty intervals for DALYs-per-case were estimated conservatively using propagated GBD uncertainty bounds, with lower estimates calculated as DALY lower UI divided by incidence upper UI, and upper estimates calculated as DALY upper UI divided by incidence lower UI.

For descriptive interpretation, lower MIR values indicate better survival outcomes relative to incidence, whereas higher MIR values indicate poorer outcomes. Similarly, lower DALYs-per-case values reflect a lower health loss per incident case, while higher values indicate greater disease severity and population burden. These metrics were interpreted comparatively across countries and regions using their observed distributions.

DALYs-per-case is not intended to measure individual patient severity; rather, it provides a population-level estimate of the average health loss associated with each incident case, complementing traditional burden metrics such as incidence, mortality, and total DALYs.

### Statistical analysis

Data linkage and preprocessing were performed in Microsoft Excel, including Power Query, to integrate data from multiple publicly available sources, standardize country identifiers, and prepare the analytical datasets. Trend estimation, regression modelling, and projection analyses were conducted in R version 4.5.2 (R Foundation for Statistical Computing, Vienna, Austria) using base R functions and packages from the tidyverse ecosystem. World heat maps were generated in RStudio using the ggplot2, sf, rnaturalearth, countrycode, viridis, and scales packages. Country-level data were linked to Natural Earth administrative boundaries using ISO 3166−1 alpha-3 country codes, with manual matching applied where required to ensure complete spatial representation. For the 2050 projection maps, a square-root transformation of the colour scale was applied to improve visual discrimination across countries with widely varying disease burdens, while legend values were formatted using comma separators for readability. All rates are reported per 100,000 person-years using the GBD world standard population. For baseline 2023 DALY estimates, 95% uncertainty intervals (UIs) are presented as reported by the GBD Study, whereas projection results are deterministic and do not incorporate simulation-based uncertainty.

## Results

In 2022, an estimated 2.48 million new cases of lung cancer (trachea, bronchus, and lung occurred globally, with an age-standardized incidence rate (ASIR) of 23.6 per 100,000 (world standard). Lung cancer accounted for 1.82 million deaths, with an age-standardized mortality rate (ASMR) of 16.8 per 100,000. The mortality-to-incidence ratio (MIR) was 0.73. The burden was substantially higher in males than in females. Males accounted for 1.57 million incident cases (63.4% of total) and an ASIR of 32.1 per 100,000, compared with 0.91 million cases (36.6%) and an ASIR of 16.2 per 100,000 in females. Mortality followed a similar pattern, with 1.23 million deaths in males (67.9%) and an ASMR of 24.8 per 100,000, versus 0.58 million deaths (32.1%) and an ASMR of 9.8 per 100,000 in females. The MIR was 0.78 in males and 0.64 in females, (Table 1 and S1 File). According to GBD 2023 estimates, lung cancer generated 46.7 million disability-adjusted life years (DALYs) globally (95% UI: 42.5-50.9 million), equivalent to an age-standardized DALY rate of 579 per 100,000. Males contributed 31.7 million DALYs (95% UI: 27.7-35.1 million) with a rate of 784 per 100,000, whereas females accounted for 14.9 million DALYs (95% UI: 13.6-16.6 million) and a rate of 372 per 100,000. The DALY-per-case ratio, calculated using 2022 incident cases, was 18.8 globally, 20.2 in males, and 16.5 in females, (Table 1 and S1 File).

**Table 1:** Global lung cancer burden in 2022, stratified by sex. Incidence, mortality, and rates are from GLOBOCAN 2022; DALYs and rates are from GBD 2023 (year 2023 reported as the most recent available). Uncertainty intervals (UIs) are 95%. MIR = deaths/ new cases; DALY-per-case = DALYs/ new cases. All rates are age-standardized to the world population. Large numbers are rounded to two decimal places for clarity.

Metric	Both Sexes	Males	Females
New Cases (millions)	2.48	1.57 (63.4%)	0.91 (36.6%)
ASIR (per 100,000)	23.6	32.1	16.2
Deaths (millions)	1.82	1.23 (67.9%)	0.58 (32.1%)
ASMR (per 100,000)	16.8	24.8	9.8
MIR	0.73	0.78	0.64
DALYs (millions, 2023)	46.7 (42.5-50.9)	31.7 (27.7-35.1)	14.9 (13.6-16.6)
DALY Rate (per 100,000, 2023)	579 (527-631)	784 (684-868)	372 (340-413)
DALY-per-Case	18.8	20.2	16.5

### Burden by socio-demographic index (SDI) level (2023)

**Fig 1:**
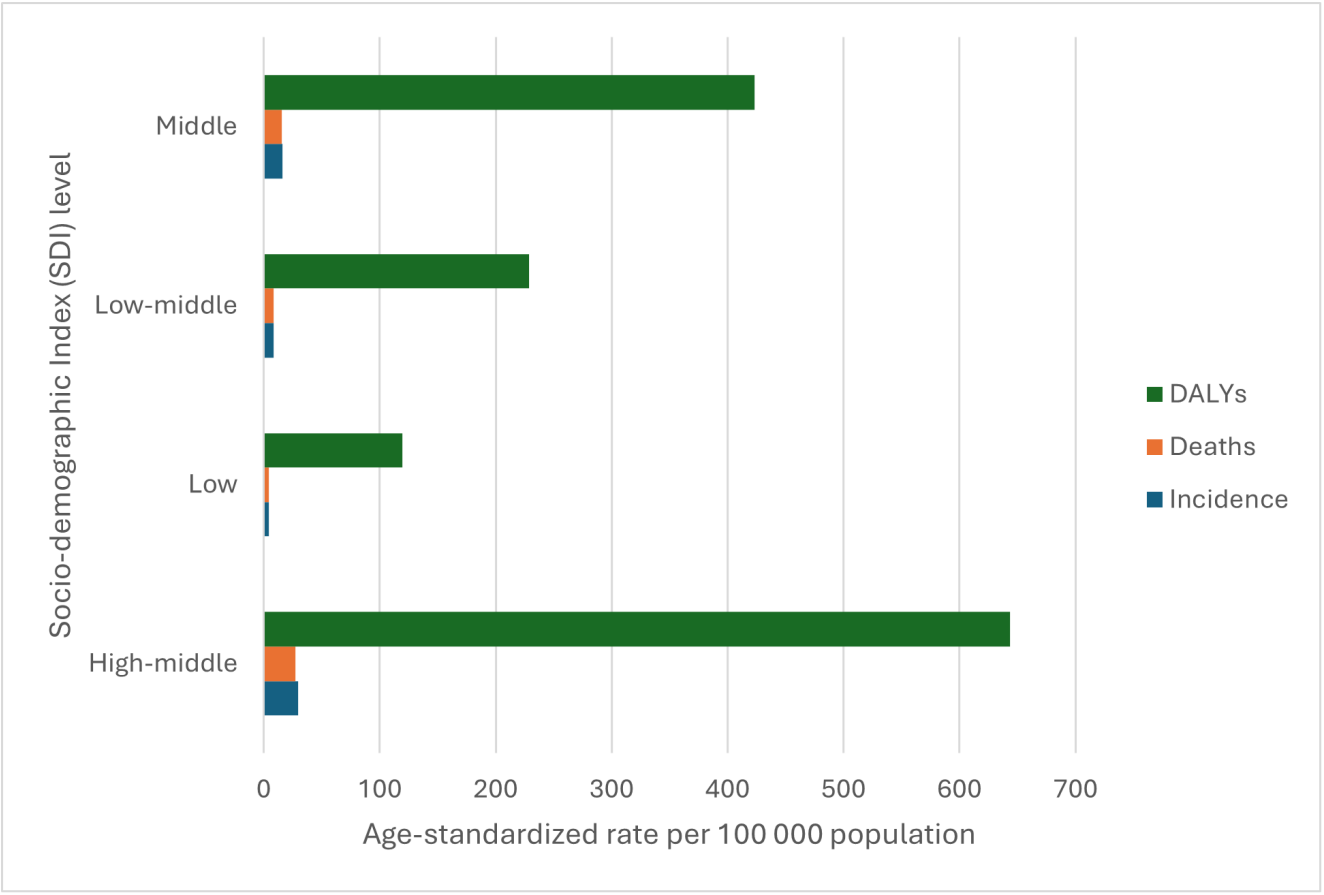
Age-standardized incidence, mortality, and disability-adjusted life-year (DALY) rates for tracheal, bronchus, and lung cancer by Socio-demographic Index (SDI) level, 2023.

GBD 2023 estimates revealed pronounced gradients in tracheal, bronchus, and lung cancer burden across Socio-demographic Index (SDI) levels. Age-standardized incidence rates increased steadily with development status, from 4.2 (3.3-5.4) per 100 000 in low SDI countries to 8.5 (7.0-10.7) in low-middle SDI, 16.2 (13.9-19.0) in middle SDI, and 29.7 (26.4-33.7) in high-middle SDI regions. Mortality rates followed a similar pattern, ranging from 4.1 (3.2-5.2) per 100 000 in low SDI countries to 8.3 (6.8-10.5) in low-middle, 15.5 (13.3-18.1) in middle, and 27.2 (24.1-30.7) in high-middle SDI levels. DALY rates displayed the same upward trend, from 119.5 (93.7-152.2) in low SDI to 228.8 (186.9-288.3) in low-middle, 423.1 (360.5-494.0) in middle, and 643.7 (573.6-725.9) per 100 000 in high-middle SDI settings. (Fig 1 and S1 File).

### Burden by GBD super region (2023)

in 2023 the highest age-standardized incidence rates were observed in high-income regions (68.4 (61.7-73.9) per 100 000), followed by Southeast Asia, East Asia, and Oceania (46.9 (40.7-54.3)) and Central Europe, Eastern Europe, and Central Asia (40.3 (38.2-42.0)). Intermediate rates occurred in North Africa and the Middle East (14.7 (11.8-17.7)) and Latin America and the Caribbean (14.0 (13.1-14.7)), while the lowest incidence rates were recorded in South Asia (7.1 (5.7-8.7)) and Sub-Saharan Africa (3.6 (2.9-4.5)).

A similar pattern was evident for mortality, with death rates highest in high-income regions (55.2 (49.9-59.0)) and Southeast Asia, East Asia, and Oceania (41.9 (36.5-47.8)), followed by Central Europe, Eastern Europe, and Central Asia (37.9 (36.3-39.1)). Mortality was lowest in Sub-Saharan Africa (3.5 (2.8-4.4)) and South Asia (6.9 (5.6-8.5)).

**Fig 2:**
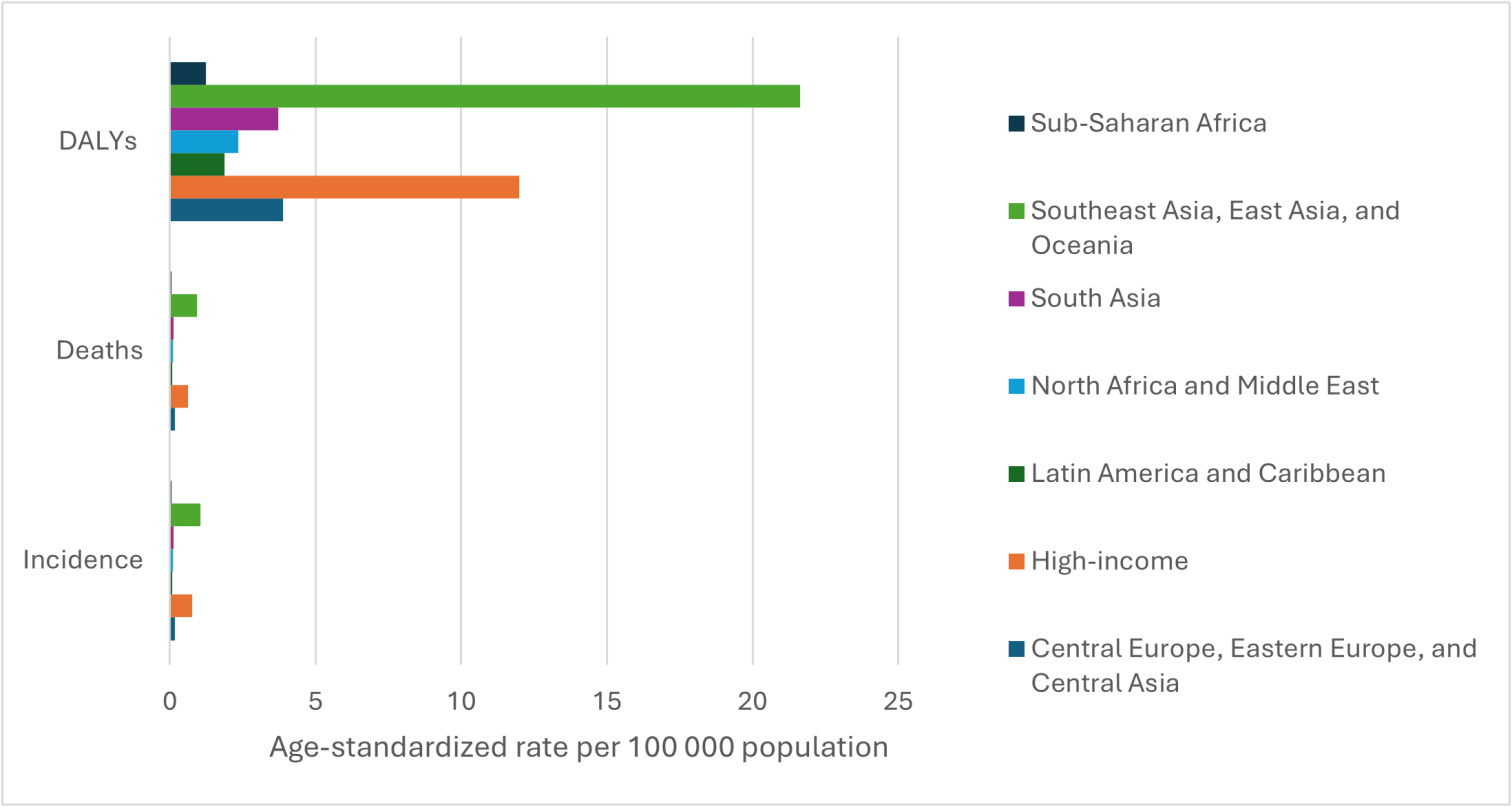
Age-standardized incidence, mortality, and disability-adjusted life-year (DALY) rates for tracheal, bronchus, and lung cancer across Global Burden of Disease (GBD) super regions, 2023.

In terms of disability-adjusted life years (DALYs), the highest regional rates were recorded in high-income areas (1 086.4 (1 011.8-1 151.9) per 100 000) and Southeast Asia, East Asia, and Oceania (979.1 (853.6-1 107.9)), followed by Central Europe, Eastern Europe, and Central Asia (918.1 (884.5-946.8)). DALY rates remained lowest in Sub-Saharan Africa (102.9 (81.5-129.9)) and South Asia (196.7 (157.6-241.6)). (Fig 2 and S1 File).

### Temporal trends in age-standardized rates, 2010−23

Between 2010 and 2023, age-standardized incidence and mortality rates for tracheal, bronchus, and lung cancer demonstrated substantial heterogeneity across countries and sexes. Most countries exhibited positive AAPCs, indicating continued increases in disease burden, although the magnitude and direction of change varied widely (S1 File).

Across all countries, male incidence AAPCs ranged from −0.030 to 0.071, with increases ≥ 0.03 observed in Bangladesh (0.0508), Sri Lanka (0.0443), Ghana (0.0340), Côte d’Ivoire (0.0403), Togo (0.0410), and Viet Nam (0.0452). Declining male incidence was confined primarily to higher-income settings, including the USA (−0.0195), Belgium (−0.0183), Sweden (−0.0209), Norway (−0.0103), and Australia (−0.0122). Female incidence AAPCs ranged from −0.033 to 0.068, with the highest annual increases recorded in Bangladesh (0.0687), Sri Lanka (0.0583), Ghana (0.0577), Lao People’s Democratic Republic (0.0601), Côte d’Ivoire (0.0510), and Morocco (0.0618). Reductions were observed in a small number of countries, including Kazakhstan (−0.0335), Sweden (0.00009), and the USA (−0.0096).

**Fig 3:**
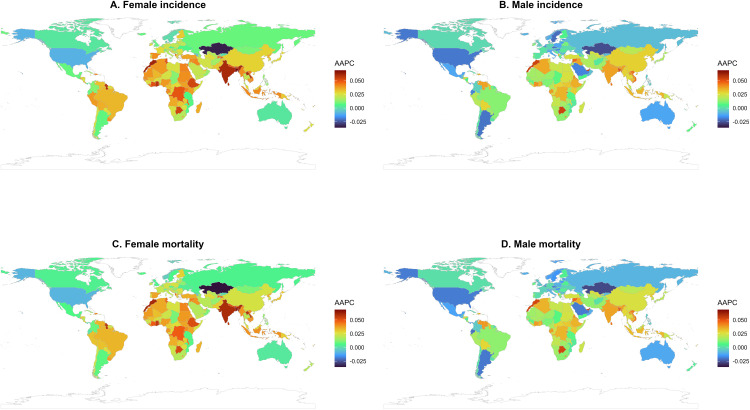
(A) world heat map AAPC in females incidence rates; (B) world heat map AAPC in males incidence rates; (C) world heat map AAPC in females mortality rates and (D) world heat map AAPC in males mortality rates. Source: Author-generated figure using Natural Earth public-domain map data (https://www.naturalearthdata.com/).

For mortality, male AAPCs varied from −0.029 to 0.049, with the largest increases in Bangladesh (0.0495), Rwanda (0.0483), Sri Lanka (0.0440), Côte d’Ivoire (0.0382), and Togo (0.0407). Declining male mortality trends were seen predominantly in high-income regions, including Sweden (−0.0153), Belgium (−0.0192), Norway (−0.0132), Australia (−0.0117), and the USA (−0.0189). Female mortality AAPCs ranged from −0.035 to 0.066, with the steepest increases in Bangladesh (0.0662), Sri Lanka (0.0562), Ghana (0.0560), Rwanda (0.0526), Côte d’Ivoire (0.0482), and Lao People’s Democratic Republic (0.0581). The most pronounced declines were noted in Kazakhstan (−0.0355), Sweden (−0.0075), the USA (−0.0088), and Norway (−0.0031). in general, female AAPC values exceeded male values in the majority of countries for both incidence and mortality, and the most rapid increases, frequently exceeding 0.04 annually, were concentrated in South Asia, sub-Saharan Africa, and selected Middle Eastern countries. In contrast, the most consistent declines were largely confined to Western Europe, North America, and parts of Oceania and Eastern Europe. (Fig 3A-Dand S1 File).

### Projected 2050

#### Scenario 1: Constant age-specific rates, population-growth only.

Projected lung cancer burden to 2050 varied substantially across countries when population growth and ageing were the only drivers of change. In most low- and lower-middle-income countries, demographic expansion resulted in marked increases in both incidence and mortality, although uncertainty ranges were wide in several data-sparse settings. In sub-Saharan Africa, projected incidence increases frequently exceeded 300%, including Burundi (+363%), Uganda (+363%), Tanzania (+316%), Chad (+406%), Central African Republic (+383%), Somalia (+380%), and Zambia (+458%). Similar increases were observed for mortality. Yemen (+277% incidence; + 280% mortality), Benin (+276%; + 290%), Mauritania (+241%; + 249%), Rwanda (+197%; + 204%), and Liberia (+179%; + 185%) also demonstrated substantial projected increases.

South Asian countries likewise showed strong demographic amplification. Pakistan (+132% incidence; + 133% mortality), Bangladesh (+43%; + 43%), Nepal (+35%; + 35%), and India (+21%; + 21%) all demonstrated sustained growth under constant 2022 rates. In North Africa and the Middle East, increases ranged from moderate to high, including Egypt (+94%; + 97%), Iraq (+249%; + 248%), Jordan (+130%; + 133%), and Saudi Arabia (+68%; + 74%). Several Gulf countries exhibited particularly large proportional increases despite relatively small baseline counts, including Bahrain (+168% incidence), Qatar (+153%), Kuwait (+94%), and the United Arab Emirates (+278%).

**Fig 4:**
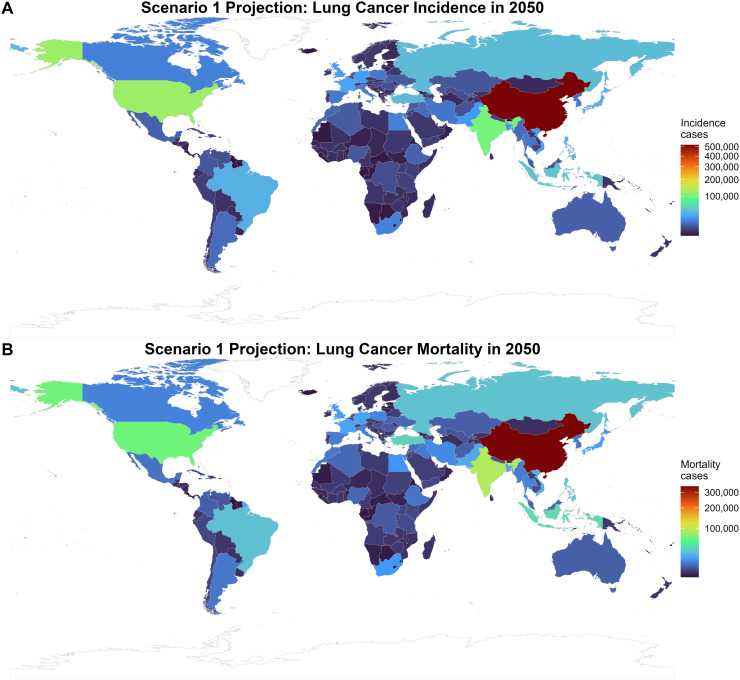
Scenario 1, 2050 constant age-specific rates, population-growth only. Author-generated figure using Natural Earth public-domain map data (https://www.naturalearthdata.com/).

By contrast, most high-income countries demonstrated substantial declines under Scenario 1, reflecting population ageing dynamics and projected demographic contraction. Incidence reductions exceeded 50% in Japan (−76%), Italy (−70%), Germany (−64%), Greece (−67%), Czechia (−61%), Republic of Korea (−61%), Spain (−58%), Poland (−59%), Canada (−52%), and the United Kingdom (−55%). Similar declines were observed across Eastern Europe and parts of Latin America, including Croatia, Serbia, Romania, Bulgaria, Cuba, Argentina, and Uruguay. (Fig 4A-B and S1 File).

#### Scenario 2: Trend-based model incorporating AAPCs.

incorporation of country-specific temporal trends (AAPCs) produced a substantially wider distribution of projected burden than Scenario 1. Countries with positive historical AAPCs experienced markedly greater increases in projected incidence and mortality, whereas countries with sustained annual declines demonstrated additional reductions beyond demographic effects alone.

Very large proportional increases (≥500%) were observed across several sub-Saharan African countries where positive AAPCs compounded demographic expansion. These included Ethiopia (+1043% incidence; + 999% mortality), Zambia (+908%; + 935%), Uganda (+948%; + 943%), Burundi (+963%; + 929%), Rwanda (+1078%; + 1074%), Angola (+1026%; + 1015%), and Mali (+844%; + 856%). Similar acceleration was observed in parts of West and Central Africa, including Ghana, Senegal, Sudan, Nigeria, and Tanzania.

Several countries in South Asia and the Middle East also showed major rate-driven escalation beyond that observed in Scenario 1. Pakistan (+293% incidence; + 288% mortality), Bangladesh (+554%; + 523%), Iraq (+876%; + 824%), Jordan (+324%; + 302%), Iran (+93%; + 73%), Yemen (+432%; + 435%), and the Syrian Arab Republic (+803%; + 829%) all exhibited substantially amplified future burden under trend continuation assumptions. In Southeast Asia, Indonesia (+203% incidence; + 202% mortality), Malaysia (+127%; + 128%), Philippines (+46%; + 48%), Viet Nam (+219%; + 203%), and Lao People’s Democratic Republic (+537%; + 511%) also demonstrated marked increases.

**Fig 5:**
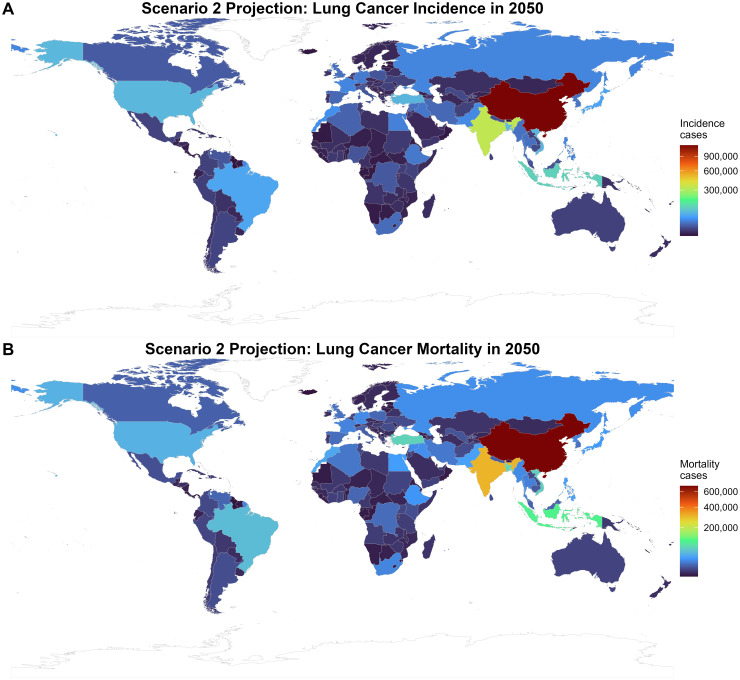
Scenario 2; 2050 Trend-based model incorporating AAPCs. Author-generated figure using Natural Earth public-domain map data (https://www.naturalearthdata.com/).

Conversely, countries with long-standing reductions in smoking prevalence and declining age-standardised rates demonstrated further decreases under Scenario 2. Incidence reductions exceeded 50%−60% in Japan (−58%), United States (−64%), Sweden (−65%), Republic of Korea (−15% but larger mortality reduction), Germany (−53%), United Kingdom (−52%), Netherlands (−52%), Italy (−69%), and Greece (−52%). Similar downward trends were observed across several Eastern European countries, including Serbia, Czechia, Poland, Romania, and Bulgaria. (Fig 5A-B and S1 File).

### DALYs per case

A moderate positive association was identified between national mortality-to-incidence ratios (MIR) and DALYs per case, indicating that countries with higher lethality tended to experience greater disability burden per diagnosed individual. Spearman’s rank correlation coefficient was Spearman’s r_s = 0.46 confirming a statistically meaningful, though not deterministic, monotonic relationship between the two-severity metrics. On the basis of the global medians (MIR = 0.962; DALYs per case = 25.14), countries were stratified into four severity quadrants to characterize heterogeneity in outcome profiles, Table 2.

**Table 2:** Country distribution across the two-Dimensional lung cancer severity framework: Top 10 countries per quadrant and characteristic severity patterns.

Quadrant	Top 10 Countries	Characteristic Severity Pattern
Low MIR/ Low DALY	Japan, Australia, South Korea, Singapore, Norway, Finland, Canada, United Kingdom, France, United States	MIR < 0.85 and DALYs per case < 18
Low MIR/ High DALY	Burundi, Eritrea, Zambia, Malawi, Uganda, Côte d’Ivoire, South Sudan, Eswatini, Namibia, Djibouti	DALYs per case ≥ 29 despite MIR < 0.96
High MIR/ Low DALY	Poland, Argentina, Brazil, Honduras, Dominican Republic, Paraguay, Armenia, Bolivia, Mexico, Croatia	MIR ≥ 0.96 but DALYs per case < 25
High MIR/ High DALY	Afghanistan, Cabo Verde, Burkina Faso, Comoros, Senegal, Timor-Leste, Ethiopia, Sierra Leone, Somalia, Nigeria	MIR ≥ 0.962 and DALYs per case ≥ 25.14

The Low MIR/Low DALY quadrant, representing comparatively favorable prognostic patterns, was dominated by high-performing health systems with well-established early detection and treatment infrastructure, including Japan, Australia, South Korea, Singapore, Norway, Finland, Canada, the United Kingdom, France, and the United States. These countries consistently exhibited MIR values below 0.85 and DALYs per case below 18, reflecting effective translation of incidence into survivorship with minimal disability accrual.

Countries positioned in the Low MIR/High DALY quadrant exhibited limited lethality relative to incidence but disproportionately elevated disability burden per case, most prominently Burundi, Eritrea, Zambia, Malawi, Uganda, Côte d’Ivoire, South Sudan, Eswatini, Namibia, and Djibouti. This pattern likely reflects constrained access to palliative, rehabilitative, and supportive care services, whereby patients survive initial diagnosis but sustain prolonged disease-related disability in the absence of adequate management.

The High MIR/ Low DALY quadrant, reflecting elevated case fatality relative to incidence despite comparatively moderate disability burden, comprised Poland, Argentina, Brazil, Honduras, Dominican Republic, Paraguay, Armenia, Bolivia, Mexico, and Croatia. This counterintuitive configuration may indicate that deaths occur early in the disease course before substantial disability accumulates, or alternatively that disability ascertainment is incomplete in some settings.

**Fig 6:**
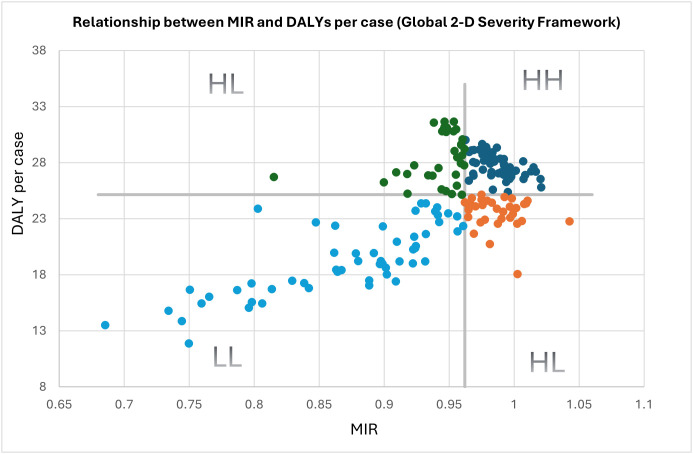
Relationship between mortality-incidence ratio (MIR) and DALYs per case (Global 2-D Severity Framework), (HL) High burden/ low severity; (HH) High burden/ High severity; (LL) Low burden/ low severity and (HL) High burden/ Low severity.

Finally, the High MIR/ High DALY quadrant, representing the most severe combined burden profile, characterized by both elevated lethality and high disability accrual per diagnosed case, included Afghanistan, Cabo Verde, Burkina Faso, Comoros, Senegal, Timor-Leste, Ethiopia, Sierra Leone, Somalia, and Nigeria. These nations face a dual disadvantage of high fatality and high morbidity, underscoring profound gaps in prevention, early diagnosis, and disease management capacity. To facilitate interpretation, countries were grouped according to relative severity patterns based on combinations of MIR and DALYs-per-case values. Countries with higher values for both metrics were considered to have a greater overall severity burden, whereas countries with lower values for both metrics were considered to have a lower severity burden. (Table 3, Fig 6, and S1 File).

**Table 3:** Sensitivity analysis of the 2D framework.

Sensitivity scenario	Threshold	% unchanged
MIR −5%	0.913	100%
MIR + 5%	0.99	100%
DALY −10%	22.63	78.90%
DALY +10%	27.66	78.40%
Raw DALYs vs DALYs-per-case	Median split	36.9%

Sensitivity analysis demonstrated complete stability of quadrant classification under ±5% variation in the MIR threshold, with no countries changing quadrant assignment. In contrast, ± 10% variation in the DALYs-per-case threshold resulted in approximately 21% of countries being reassigned, indicating moderate sensitivity around the DALYs-per-case median cut-point where many countries clustered near the threshold. Nevertheless, the overall clustering structure and the broad separation between high-burden and low-burden settings remained consistent across all sensitivity scenarios, Table 3.

To evaluate whether DALYs-per-case provides additional information beyond raw DALYs, quadrant classifications were compared across both metrics. Of 176 countries with complete data, 111 (63.1%) were assigned to a different quadrant when DALYs-per-case was used instead of raw DALYs (Cohen’s κ = 0.165, indicating only slight agreement). Countries reclassified under raw DALYs were predominantly high-population nations (e.g., China, United States, Brazil, India) which appeared in high-burden quadrants solely due to absolute case volume, despite having relatively low per-case health loss. Conversely, small low-income countries with high per-case severity (e.g., Burundi, Uganda, Côte d’Ivoire) were misclassified as low-burden under raw DALYs. This demonstrates that raw DALYs predominantly reflects population size, whereas DALYs-per-case isolates per-patient disease severity independent of country size, information that raw DALYs does not provide. Full country-level classifications are provided in S2 File, and sensitivity figures are provided in S1 and S2 Figs.

## Discussion

This study was designed to advance global lung cancer assessment by moving beyond conventional population-level indicators toward a severity-focused framework that captures how lung cancer is experienced by individual patients across health systems. Lung cancer remains the leading cause of cancer-related mortality worldwide, accounting for more deaths than any other malignancy and imposing a substantial and growing global health burden (4). Although incidence, mortality, MIR, and DALYs are essential for quantifying population burden, these measures primarily describe the scale of disease rather than the severity experienced by each affected individual.

The principal innovation of this study is the introduction of DALYs-per-case, together with its integration with the mortality-to-incidence ratio (MIR) into a two-dimensional global severity framework. DALYs-per-case quantifies the average lifetime health loss attributable to each newly diagnosed lung cancer case, thereby shifting analytical focus from population totals to patient-level severity. To our knowledge, this is the first global study to combine DALYs-per-case and MIR into a unified severity map, extending lung cancer assessment beyond traditional burden metrics and beyond incidence- or mortality-only projections [10,43,44].

Our analysis revealed marked heterogeneity in lung cancer severity across countries that is not captured by traditional indicators alone. DALYs-per-case varied widely across settings, reflecting differences in stage at diagnosis, survival duration, access to effective treatment, and quality of survivorship and palliative care [39,42]. Countries with high DALYs-per-case experienced substantially greater lifetime health loss per diagnosed patient, even when overall incidence or mortality rates were relatively modest [45,46].

By contrast, MIR primarily captured fatality relative to incidence, serving as a proxy for survival but providing limited insight into the duration or intensity of disability experienced by patients who survive beyond diagnosis [30,44,47,48]. The moderate correlation between DALYs-per-case and MIR (Spearman ρ = 0.44) provides empirical evidence that these metrics capture related but distinct dimensions of disease severity. Had DALYs-per-case simply mirrored MIR, a near-perfect correlation would be expected [22,25,47]. Instead, the observed relationship supports the conceptual justification for a two-dimensional severity framework capable of capturing both fatal and non-fatal dimensions of lung cancer burden.

The two-dimensional severity framework yielded four clinically and epidemiologically interpretable profiles. Countries in the high MIR/ high DALYs-per-case quadrant represent the most severe lung cancer settings, characterized by high fatality and extreme lifetime health loss per patient. These patterns are consistent with late-stage diagnosis, limited access to curative treatment, and prolonged inadequately managed disease, often reflecting structural weaknesses across prevention, detection, and care delivery [27,40].

The low MIR/ low DALYs-per-case quadrant reflects the most favourable outcomes, typically observed in high-performing health systems with effective tobacco control, early detection, timely treatment, and comprehensive survivorship and palliative care [27,49]. These settings demonstrate that lung cancer severity can be substantially mitigated through sustained investments in cancer control.

The low MIR/ high DALYs-per-case quadrant suggests relatively improved survival but disproportionately high disability burden, potentially reflecting prolonged treatment courses, suboptimal symptom control, or inadequate survivorship services [42,41]. Conversely, the high MIR/ low DALYs-per-case quadrant reflects rapid progression to death with comparatively shorter disability duration, consistent with late diagnosis and minimal therapeutic intervention [48]. Together, these mixed profiles reveal specific system-level weaknesses that remain obscured under single-metric approaches but become visible within the two-dimensional framework.

A key contribution of this study is the explicit linkage between contemporary severity patterns and future lung cancer burden. While population growth and aging are major drivers of projected increases in lung cancer incidence and mortality, our findings demonstrate that severity patterns substantially influence future health loss. Countries with high DALYs-per-case are likely to experience disproportionate increases in total health loss by 2050, even when incidence growth is modest, because each new case carries a greater lifetime burden [29,50,51].

Severity-adjusted projections therefore provide insights that are not captured by incidence- or mortality-only models. Incorporating DALYs-per-case enables identification of settings where future lung cancer burden will be amplified by poor survival and prolonged disability, supporting more accurate planning for treatment capacity, survivorship services, and palliative care [2]. This approach complements existing forecasting models by aligning projected case numbers with anticipated patient-level health loss.

### Implications for policy and cancer control

The proposed severity framework yields actionable policy insights. In countries characterized by high DALYs-per-case and high MIR, priorities should focus on strengthening early detection, diagnostic capacity, and access to curative treatment. Settings with moderate MIR but high DALYs-per-case may benefit most from investments in survivorship care, symptom management, and palliative services aimed at reducing prolonged disability [42,52].

Where incidence is high regardless of severity profile, primary prevention strategies, particularly tobacco control and environmental risk reduction, remain essential. By distinguishing between fatality-driven and disability-driven severity, this framework enables targeted, context-specific interventions rather than uniform policy responses based solely on incidence or mortality.

The quadrant framework demonstrated high robustness to MIR threshold variation, reflecting the constrained distribution of MIR values near the upper theoretical boundary. However, moderate reassignment was observed following variation of DALYs-per-case thresholds, likely due to the concentration of several countries near the global median. Accordingly, the framework should be interpreted primarily as a descriptive visualization tool for comparative severity assessment rather than a rigid categorical classification system.

Beyond threshold sensitivity, the low agreement between raw DALYs and DALYs-per-case quadrant classifications provides compelling justification for adopting DALYs-per-case within the severity framework. Raw DALYs, being directly proportional to population size, systematically privileges large-population countries in burden rankings regardless of the health loss experienced per patient. DALYs-per-case corrects for this by normalising to incidence, thereby revealing settings where each cancer case carries disproportionately high lifetime health loss, a characteristic more closely tied to late-stage diagnosis, treatment access, and healthcare system quality than to country size alone. This distinction carries important policy implications: prioritising countries by raw DALYs alone would direct resources toward the most populous nations, whereas DALYs-per-case identifies settings where the marginal health gain per intervention is greatest, enabling more equitable and efficient allocation of cancer control resources.

### Strengths & limitations

This study has several important strengths. It provides the first global estimates of DALYs-per-case for lung cancer, introduces the first two-dimensional severity map integrating DALYs-per-case and MIR, and combines burden, severity, temporal trends, and future projections within a unified analytical framework. This integrated approach offers a more comprehensive understanding of lung cancer burden than population-level metrics alone.

Several limitations should be acknowledged. DALY estimates are model-based and subject to uncertainty [8]; MIR is an indirect proxy for survival rather than a direct survival measure [22,27] and projection scenarios assume continuation of recent trends without explicitly incorporating future advances in screening, treatment, or policy [10,50]. Nevertheless, these limitations do not diminish the central contribution of this work, which lies in reframing global lung cancer assessment around patient-level severity rather than aggregate burden alone.

## Conclusion

Global lung cancer burden is projected to rise substantially in many regions despite declining rates in high-income settings. Integrating DALYs-per-case with MIR provides a complementary severity lens that highlights inequities not captured by incidence and mortality alone and supports more targeted prevention, treatment, and survivorship strategies.

## Supporting information

S1 File Supplementary tables and figures supporting the analyses presented in this study. Supplementary Table A. Country-specific lung cancer incidence, mortality, age-standardized incidence rates, age-standardized mortality rates, and mortality-to-incidence ratios in 2022. Supplementary Table B. Country-specific disability-adjusted life years, age-standardized DALY rates, and DALYs-per-case estimates in 2023. Supplementary Table C. Lung cancer burden indicators stratified by Socio-demographic Index level. Supplementary Table D. Lung cancer burden indicators stratified by GBD super-regions. Supplementary Table E. Country-specific average annual percentage changes in incidence and mortality between 2010 and 2023. Supplementary Table F. Scenario 1 projections to 2050. Supplementary Table G. Scenario 2 projections to 2050. Supplementary Table H. Severity framework classifications.
(PDF)

S2 File Country-level comparison of quadrant assignments under the MIR-DALYs and MIR-DALYs-per-case frameworks. (PDF)

S1 Fig Transition matrix. (PNG)

S2 Fig Sensitivity analysis/ reclassification. (PNG)
