# Supplementary material for: Global lung cancer burden, trends, and projections from 2010 to 2050: A population-level severity framework integrating DALYs-per-case and mortality-to-incidence ratio
Source: PLoS One. 2026 Jul 23;21(7):e0354350. doi: 10.1371/journal.pone.0354350 (PMC13395349; doi:10.1371/journal.pone.0354350)
Supplement: S1 File — Supplementary Table A. Country-specific lung cancer incidence, mortality, age-standardized incidence rates, age-standardized mortality rates, and mortality-to-incidence ratios in 2022. Supplementary Table B. Country-specific disability-adjusted life years, age-standardized DALY rates, and DALYs-per-case estimates in 2023. Supplementary Table C. Lung cancer burden indicators stratified by Socio-demographic Index level. Supplementary Table D. Lung cancer burden indicators stratified by GBD super-regions. Supplementary Table E. Country-specific average annual percentage changes in incidence and mortality between 2010 and 2023. Supplementary Table F. Scenario 1 projections to 2050. Supplementary Table G. Scenario 2 projections to 2050. Supplementary Table H. Severity framework classifications. (PDF) [file pone.0354350.s001.pdf]

Supplementary A:

Country	Sex	Measure	Number	95% UI low	95% UI high	ASR (World)	
Afghanistan	Male	Incidence	1019	715	1453	11	
Albania	Male	Incidence	1134	978	1315	41.5	
Algeria	Male	Incidence	4311	4065	4572	19.5	
Angola	Male	Incidence	223	223	223	3.3	
Azerbaijan	Male	Incidence	2004	1784	2251	37.5	
Argentina	Male	Incidence	8587	7998	9220	30	
Australia	Male	Incidence	7353	7042	7677	27.1	
Austria	Male	Incidence	3004	2835	3183	31.2	
Bahamas	Male	Incidence	30	7	128	13.1	
Bahrain	Male	Incidence	64	31	133	15.1	
Bangladesh	Male	Incidence	10033	9715	10362	13.5	
Armenia	Male	Incidence	1145	972	1349	57.4	
Barbados	Male	Incidence	33	15	74	13.2	
Belgium	Male	Incidence	5469	5266	5680	42.3	
Bhutan	Male	Incidence	26	26	26	6.5	
Bolivia (Plurin	Male	Incidence	769	642	921	11.6	
Bosnia Herze	Male	Incidence	1862	1738	1995	59.5	
Botswana	Male	Incidence	46	25	84	6.5	
Brazil	Male	Incidence	24804	23983	25653	18.3	
Belize	Male	Incidence	21	7	63	13.6	
Solomon Islar	Male	Incidence	21	13	34	9.4	
Brunei Daruss	Male	Incidence	57	36	90	25.6	
Bulgaria	Male	Incidence	2916	2738	3106	43.8	
Myanmar	Male	Incidence	4983	4735	5244	21.6	
Burundi	Male	Incidence	97	56	169	4.2	
Belarus	Male	Incidence	4006	3712	4323	56.5	
Cambodia	Male	Incidence	1374	1351	1397	23.9	
Cameroon	Male	Incidence	259	44	1519	4.4	
Canada	Male	Incidence	15900	15174	16661	34.9	
Cape Verde	Male	Incidence	3	1	10	1.8	
Central Africa	Male	Incidence	28	9	83	2.7	
Sri Lanka	Male	Incidence	1666	1519	1828	11.2	
Chad	Male	Incidence	134	112	161	3.5	
Chile	Male	Incidence	2619	2334	2939	17	

China	Male	Incidence	658722	649603	667969	52	
Colombia	Male	Incidence	4178	3855	4529	13.1	
Comoros	Male	Incidence	6	4	10	2.6	
Congo, Repul	Male	Incidence	27	13	57	2.1	
Congo, Demo	Male	Incidence	594	179	1975	2.8	
Costa Rica	Male	Incidence	360	298	435	9.7	
Croatia	Male	Incidence	2492	2286	2717	56.4	
Cuba	Male	Incidence	4426	4004	4893	38.3	
Cyprus	Male	Incidence	456	392	530	41.4	
Czechia	Male	Incidence	3709	3531	3896	31.3	
Benin	Male	Incidence	60	33	109	2.1	
Denmark	Male	Incidence	2408	2230	2600	34.3	
Dominican Re	Male	Incidence	896	750	1071	15.1	
Ecuador	Male	Incidence	664	543	811	6.6	
El Salvador	Male	Incidence	182	128	259	5.2	
Equatorial Gu	Male	Incidence	11	5	23	2.9	
Ethiopia	Male	Incidence	1226	1097	1370	3.9	
Eritrea	Male	Incidence	38	29	49	3.7	
Estonia	Male	Incidence	563	495	640	45	
Fiji	Male	Incidence	37	19	73	9.1	
Finland	Male	Incidence	1828	1737	1924	25.1	
France (metro	Male	Incidence	32812	31660	34005	49	
French Guyar	Male	Incidence	32	32	32	23.8	
French Polyne	Male	Incidence	111	107	115	57.4	
Djibouti	Male	Incidence	16	11	23	3.6	
Gabon	Male	Incidence	25	9	70	3.5	
Georgia	Male	Incidence	1363	1165	1595	45.9	
The Republic	Male	Incidence	50	29	87	9.5	
Gaza Strip an	Male	Incidence	485	407	578	35.8	
Germany	Male	Incidence	36871	35496	38299	35.2	
Ghana	Male	Incidence	299	235	380	3.1	
Greece	Male	Incidence	6357	5886	6866	49.6	
France, Guad	Male	Incidence	71	70	72	18.7	
Guam	Male	Incidence	55	54	56	45.5	
Guatemala	Male	Incidence	293	225	382	4.5	
Guinea	Male	Incidence	160	96	268	5.2	
Guyana	Male	Incidence	26	9	73	6.4	

Haiti	Male	Incidence	232	180	298	5.5	
Honduras	Male	Incidence	312	242	403	8.8	
Hungary	Male	Incidence	5706	5414	6014	64.4	
Iceland	Male	Incidence	92	64	132	26.1	
India	Male	Incidence	58970	57866	60095	8.5	
Indonesia	Male	Incidence	29107	17998	47073	21.3	
Iran, Islamic F	Male	Incidence	7576	7278	7886	16.7	
Iraq	Male	Incidence	2364	2186	2556	23.5	
Ireland	Male	Incidence	1557	1435	1689	32.6	
Israel	Male	Incidence	1818	1654	1998	29.9	
Italy	Male	Incidence	28450	27542	29388	34.5	
Côte d'Ivoire	Male	Incidence	243	188	314	3.5	
Jamaica	Male	Incidence	350	225	545	19	
Japan	Male	Incidence	95740	90953	100778	47.2	
Kazakhstan	Male	Incidence	3822	3601	4056	41.4	
Jordan	Male	Incidence	853	748	973	24.1	
Kenya	Male	Incidence	421	327	542	3.6	
Korea, Democ	Male	Incidence	7865	7764	7968	50.1	
Korea, Repub	Male	Incidence	21183	20798	21575	38.6	
Kuwait	Male	Incidence	155	117	206	7.7	
Kyrgyzstan	Male	Incidence	597	469	761	26.3	
Lao People's	Male	Incidence	695	682	708	27.1	
Lebanon	Male	Incidence	1004	892	1130	25.7	
Lesotho	Male	Incidence	31	11	87	5	
Latvia	Male	Incidence	776	695	867	45.7	
Liberia	Male	Incidence	63	36	109	4	
Libya	Male	Incidence	836	717	975	32.2	
Lithuania	Male	Incidence	1104	983	1239	44.9	
Luxembourg	Male	Incidence	210	155	284	31.6	
Madagascar	Male	Incidence	200	114	352	2.8	
Malawi	Male	Incidence	86	43	171	2.2	
Malaysia	Male	Incidence	4196	2455	7172	23.2	
Maldives	Male	Incidence	37	29	48	18.1	
Mali	Male	Incidence	193	152	245	4.5	
Malta	Male	Incidence	164	129	208	27.7	
France, Martir	Male	Incidence	55	54	56	12.7	
Mauritania	Male	Incidence	37	6	220	2.8	

Mauritius	Male	Incidence	124	91	169	12.2	
Mexico	Male	Incidence	5062	4662	5497	7.3	
Mongolia	Male	Incidence	418	347	503	35.9	
Republic of M	Male	Incidence	1178	1047	1326	42.8	
Montenegro	Male	Incidence	310	262	366	58.8	
Morocco	Male	Incidence	7884	7511	8276	38.4	
Mozambique	Male	Incidence	142	101	199	2.4	
Oman	Male	Incidence	124	90	171	7	
Namibia	Male	Incidence	52	31	88	8.3	
Nepal	Male	Incidence	1528	1491	1566	12.9	
The Netherlar	Male	Incidence	7793	7492	8106	36.5	
New Caledoni	Male	Incidence	115	112	118	59.3	
Vanuatu	Male	Incidence	12	7	22	11.8	
New Zealand	Male	Incidence	1659	1521	1809	33.1	
Nicaragua	Male	Incidence	188	132	268	7.1	
Niger	Male	Incidence	97	42	225	1.8	
Nigeria	Male	Incidence	812	678	973	1.9	
Norway	Male	Incidence	1799	1617	2001	28.7	
Pakistan	Male	Incidence	7571	7225	7933	9.5	
Panama	Male	Incidence	237	165	341	8.7	
Papua New G	Male	Incidence	384	194	761	13.6	
Paraguay	Male	Incidence	740	634	863	21.4	
Peru	Male	Incidence	1443	1274	1635	6.9	
Philippines	Male	Incidence	16588	15921	17283	37.7	
Poland	Male	Incidence	19206	18617	19813	52.5	
Portugal	Male	Incidence	4253	3960	4568	36.9	
Guinea-Bissau	Male	Incidence	15	3	87	3.1	
Timor-Leste	Male	Incidence	63	50	79	14.7	
Puerto Rico	Male	Incidence	471	401	554	14.9	
Qatar	Male	Incidence	77	50	119	6.1	
France, La Ré	Male	Incidence	267	261	273	36.3	
Romania	Male	Incidence	8499	8080	8940	49.3	
Russian Fede	Male	Incidence	56078	55062	57113	52.4	
Rwanda	Male	Incidence	161	118	220	4.4	
Saint Lucia	Male	Incidence	21	8	58	16.5	
Sao Tome an	Male	Incidence	10	1	147	16	
Saudi Arabia	Male	Incidence	939	828	1064	5.6	

Senegal	Male	Incidence	175	16	1942	4.2	
Serbia	Male	Incidence	4669	4408	4946	59.6	
Sierra Leone	Male	Incidence	52	14	192	2.7	
Singapore	Male	Incidence	1981	1828	2147	33.2	
Slovakia	Male	Incidence	1840	1615	2096	36.5	
Viet Nam	Male	Incidence	17007	16511	17518	31.5	
Slovenia	Male	Incidence	1042	948	1146	43.8	
Somalia	Male	Incidence	145	105	200	3.9	
South Africa	Male	Incidence	6053	6034	6072	27.6	
Zimbabwe	Male	Incidence	255	198	328	9.2	
Spain	Male	Incidence	21654	20814	22528	41.4	
South Sudan	Male	Incidence	72	22	231	2.4	
Sudan	Male	Incidence	407	322	514	3.1	
Suriname	Male	Incidence	73	37	144	24.1	
Eswatini	Male	Incidence	19	8	47	7.6	
Sweden	Male	Incidence	1978	1844	2122	15.8	
Switzerland	Male	Incidence	2795	2545	3070	27.9	
Syrian Arab R	Male	Incidence	1987	1827	2161	28.7	
Tajikistan	Male	Incidence	219	159	301	7.1	
Thailand	Male	Incidence	15200	14741	15674	25.9	
Togo	Male	Incidence	62	20	189	3.1	
Trinidad and T	Male	Incidence	199	135	293	19.2	
United Arab E	Male	Incidence	184	139	244	6.8	
Tunisia	Male	Incidence	2742	2539	2961	37.8	
Türkiye	Male	Incidence	33039	32342	33751	68	
Turkmenistan	Male	Incidence	372	153	903	16.1	
Uganda	Male	Incidence	293	218	394	3.5	
Ukraine	Male	Incidence	13660	13318	14011	41.7	
North Macedo	Male	Incidence	873	684	1114	49.4	
Egypt	Male	Incidence	5599	5198	6031	13.9	
United Kingdo	Male	Incidence	25711	25248	26183	31.9	
Tanzania, Uni	Male	Incidence	408	271	615	2.8	
United States	Male	Incidence	112343	110749	113960	33.7	
Burkina Faso	Male	Incidence	99	99	99	3	
Uruguay	Male	Incidence	1220	1101	1351	46.6	
Uzbekistan	Male	Incidence	1854	1627	2112	13.6	
Venezuela	Male	Incidence	3183	2872	3528	20.4	

Samoa	Male	Incidence	38	38	38	49.6	
Yemen	Male	Incidence	443	374	525	5	
Zambia	Male	Incidence	131	93	185	4.6	
Afghanistan	Male	Mortality	977	695	1373	10.3	
Albania	Male	Mortality	997	906	1097	39.9	
Algeria	Male	Mortality	3943	3696	4206	17.9	
Angola	Male	Mortality	200	200	200	3	
Azerbaijan	Male	Mortality	1665	1513	1832	31.4	
Argentina	Male	Mortality	7032	6706	7374	24.3	
Australia	Male	Mortality	5271	5050	5502	18.5	
Austria	Male	Mortality	2506	2363	2657	24.3	
Bahamas	Male	Mortality	25	9	68	10.9	
Bahrain	Male	Mortality	56	31	102	12.9	
Bangladesh	Male	Mortality	9313	9038	9596	12.5	
Armenia	Male	Mortality	952	857	1057	47.2	
Barbados	Male	Mortality	26	15	45	10.1	
Belgium	Male	Mortality	3888	3634	4159	28.3	
Bhutan	Male	Mortality	26	26	26	6.5	
Bolivia (Plurin	Male	Mortality	663	564	779	9.9	
Bosnia Herze	Male	Mortality	1528	1444	1617	46.9	
Botswana	Male	Mortality	42	21	84	6	
Brazil	Male	Mortality	21515	21024	22018	15.7	
Belize	Male	Mortality	19	8	43	12.2	
Solomon Islar	Male	Mortality	16	11	23	7.6	
Brunei Daruss	Male	Mortality	51	33	79	23.8	
Bulgaria	Male	Mortality	2538	2377	2710	37.2	
Myanmar	Male	Mortality	4580	4370	4800	19.9	
Burundi	Male	Mortality	90	52	155	4	
Belarus	Male	Mortality	3363	3224	3507	47.1	
Cambodia	Male	Mortality	1219	1201	1237	21.4	
Cameroon	Male	Mortality	241	46	1267	4.2	
Canada	Male	Mortality	11959	11660	12266	24.4	
Cape Verde	Male	Mortality	3	0	21	2	
Central Africa	Male	Mortality	26	10	71	2.6	
Sri Lanka	Male	Mortality	1520	1372	1684	10.3	
Chad	Male	Mortality	129	105	159	3.4	
Chile	Male	Mortality	2348	2166	2546	15.1	

China	Male	Mortality	515909	508009	523932	39.5	
Colombia	Male	Mortality	3532	3347	3727	11	
Comoros	Male	Mortality	6	3	11	2.6	
Congo, Repul	Male	Mortality	24	11	53	1.9	
Congo, Demo	Male	Mortality	549	181	1666	2.6	
Costa Rica	Male	Mortality	253	208	308	6.6	
Croatia	Male	Mortality	2063	1945	2188	46.3	
Cuba	Male	Mortality	3659	3431	3902	31.4	
Cyprus	Male	Mortality	394	315	493	35.5	
Czechia	Male	Mortality	3120	2925	3328	27.3	
Benin	Male	Mortality	55	28	107	2	
Denmark	Male	Mortality	1896	1763	2039	25.7	
Dominican Re	Male	Mortality	764	673	867	12.8	
Ecuador	Male	Mortality	602	519	698	6	
El Salvador	Male	Mortality	169	128	223	4.9	
Equatorial Gu	Male	Mortality	10	5	20	2.8	
Ethiopia	Male	Mortality	1202	1057	1366	3.8	
Eritrea	Male	Mortality	31	25	39	3.1	
Estonia	Male	Mortality	477	425	535	37.7	
Fiji	Male	Mortality	36	20	64	8.9	
Finland	Male	Mortality	1431	1275	1607	19.5	
France (metro	Male	Mortality	24829	24084	25597	35.1	
French Guyar	Male	Mortality	26	26	26	19.6	
French Polyne	Male	Mortality	101	98	104	51.9	
Djibouti	Male	Mortality	14	10	20	3.3	
Gabon	Male	Mortality	22	8	64	3.1	
Georgia	Male	Mortality	1122	1007	1250	37.5	
The Republic	Male	Mortality	50	26	97	9.5	
Gaza Strip an	Male	Mortality	453	384	534	33.4	
Germany	Male	Mortality	29018	28165	29897	27.4	
Ghana	Male	Mortality	290	223	378	3.1	
Greece	Male	Mortality	5417	5090	5766	40.1	
France, Guad	Male	Mortality	65	64	66	16.5	
Guam	Male	Mortality	54	53	55	44.3	
Guatemala	Male	Mortality	275	225	336	4.2	
Guinea	Male	Mortality	159	92	276	5.1	
Guyana	Male	Mortality	23	11	49	5.5	

Haiti	Male	Mortality	222	172	286	5.3	
Honduras	Male	Mortality	197	164	237	5.4	
Hungary	Male	Mortality	4890	4696	5092	54.5	
Iceland	Male	Mortality	57	44	73	15.8	
India	Male	Mortality	54220	53135	55327	7.8	
Indonesia	Male	Mortality	25589	16612	39416	19	
Iran, Islamic F	Male	Mortality	6781	6515	7058	14.9	
Iraq	Male	Mortality	2201	2015	2404	21.8	
Ireland	Male	Mortality	1051	954	1158	21.5	
Israel	Male	Mortality	1218	1099	1350	18.9	
Italy	Male	Mortality	23608	23021	24210	25.9	
Côte d'Ivoire	Male	Mortality	214	165	278	3.2	
Jamaica	Male	Mortality	297	216	408	16.1	
Japan	Male	Mortality	56905	55563	58279	22.7	
Kazakhstan	Male	Mortality	3142	2922	3379	34.1	
Jordan	Male	Mortality	791	682	918	22.5	
Kenya	Male	Mortality	382	293	498	3.3	
Korea, Democ	Male	Mortality	6506	6437	6576	42.1	
Korea, Repub	Male	Mortality	17258	16751	17780	30	
Kuwait	Male	Mortality	136	99	188	7	
Kyrgyzstan	Male	Mortality	466	403	539	20.2	
Lao People's I	Male	Mortality	626	615	637	24.3	
Lebanon	Male	Mortality	905	795	1031	23.1	
Lesotho	Male	Mortality	28	11	71	4.6	
Latvia	Male	Mortality	662	578	758	39	
Liberia	Male	Mortality	58	35	96	3.8	
Libya	Male	Mortality	766	663	885	29.9	
Lithuania	Male	Mortality	934	857	1017	38	
Luxembourg	Male	Mortality	158	125	199	25.3	
Madagascar	Male	Mortality	186	106	326	2.6	
Malawi	Male	Mortality	79	40	156	2.1	
Malaysia	Male	Mortality	3691	2208	6171	20.4	
Maldives	Male	Mortality	32	26	40	17	
Mali	Male	Mortality	189	143	250	4.5	
Malta	Male	Mortality	131	100	171	22.3	
France, Martir	Male	Mortality	51	50	52	11.8	
Mauritania	Male	Mortality	34	7	175	2.7	

Mauritius	Male	Mortality	119	86	164	11.6	
Mexico	Male	Mortality	4820	4539	5119	6.9	
Mongolia	Male	Mortality	355	318	396	31.6	
Republic of M	Male	Mortality	1022	935	1117	37	
Montenegro	Male	Mortality	257	223	296	47.5	
Morocco	Male	Mortality	7136	6774	7518	34.8	
Mozambique	Male	Mortality	139	95	203	2.3	
Oman	Male	Mortality	116	78	172	6.5	
Namibia	Male	Mortality	47	26	86	7.8	
Nepal	Male	Mortality	1389	1352	1427	11.8	
The Netherlar	Male	Mortality	6091	5867	6324	26.5	
New Caledoni	Male	Mortality	87	85	89	44.7	
Vanuatu	Male	Mortality	12	6	22	11.8	
New Zealand	Male	Mortality	1135	1022	1261	21.6	
Nicaragua	Male	Mortality	174	133	227	6.6	
Niger	Male	Mortality	96	40	229	1.8	
Nigeria	Male	Mortality	768	632	933	1.8	
Norway	Male	Mortality	1257	1144	1381	18.9	
Pakistan	Male	Mortality	6635	6336	6948	8.3	
Panama	Male	Mortality	196	154	249	7.1	
Papua New G	Male	Mortality	343	186	632	12.4	
Paraguay	Male	Mortality	628	564	699	18	
Peru	Male	Mortality	1316	1197	1447	6.2	
Philippines	Male	Mortality	14654	14062	15271	33.6	
Poland	Male	Mortality	16596	16183	17020	44.7	
Portugal	Male	Mortality	3686	3483	3901	30.4	
Guinea-Bissau	Male	Mortality	15	3	87	3.1	
Timor-Leste	Male	Mortality	55	46	66	13	
Puerto Rico	Male	Mortality	362	268	489	10.8	
Qatar	Male	Mortality	66	42	104	5.5	
France, La Ré	Male	Mortality	234	228	240	31.1	
Romania	Male	Mortality	7743	7445	8053	44.1	
Russian Fede	Male	Mortality	41249	40389	42127	38.1	
Rwanda	Male	Mortality	146	104	205	4.1	
Saint Lucia	Male	Mortality	17	9	34	13.2	
Sao Tome an	Male	Mortality	10	1	147	16	
Saudi Arabia	Male	Mortality	815	715	929	5	

Senegal	Male	Mortality	163	17	1534	3.9	
Serbia	Male	Mortality	3939	3765	4121	49.4	
Sierra Leone	Male	Mortality	47	12	180	2.6	
Singapore	Male	Mortality	1899	1571	2296	31.9	
Slovakia	Male	Mortality	1597	1445	1765	31.5	
Viet Nam	Male	Mortality	15772	15261	16300	29.3	
Slovenia	Male	Mortality	799	727	878	33.2	
Somalia	Male	Mortality	135	99	185	3.7	
South Africa	Male	Mortality	5526	5504	5548	25.5	
Zimbabwe	Male	Mortality	233	176	308	8.6	
Spain	Male	Mortality	17318	16788	17864	31.6	
South Sudan	Male	Mortality	65	22	190	2.2	
Sudan	Male	Mortality	376	303	466	2.9	
Suriname	Male	Mortality	61	37	99	20.2	
Eswatini	Male	Mortality	19	6	61	7.6	
Sweden	Male	Mortality	1718	1614	1828	12.9	
Switzerland	Male	Mortality	2029	1886	2182	19.1	
Syrian Arab R	Male	Mortality	1866	1718	2027	27.4	
Tajikistan	Male	Mortality	194	154	244	6.4	
Thailand	Male	Mortality	12853	12467	13251	21.5	
Togo	Male	Mortality	53	21	136	2.8	
Trinidad and T	Male	Mortality	161	123	210	15.5	
United Arab E	Male	Mortality	170	123	235	5.9	
Tunisia	Male	Mortality	2466	2291	2654	34	
Türkiye	Male	Mortality	32119	30865	33424	66.3	
Turkmenistan	Male	Mortality	352	152	815	15.2	
Uganda	Male	Mortality	253	187	342	3.1	
Ukraine	Male	Mortality	11634	11351	11924	35.4	
North Macedo	Male	Mortality	757	625	917	42.6	
Egypt	Male	Mortality	5000	4663	5361	12.6	
United Kingdo	Male	Mortality	18539	17982	19113	21.8	
Tanzania, Uni	Male	Mortality	359	234	551	2.5	
United States	Male	Mortality	66846	65744	67967	19	
Burkina Faso	Male	Mortality	97	97	97	3	
Uruguay	Male	Mortality	1140	1024	1269	41.8	
Uzbekistan	Male	Mortality	1561	1431	1703	11.5	
Venezuela	Male	Mortality	2673	2489	2870	17.1	

Samoa	Male	Mortality	28	28	28	37.4	
Yemen	Male	Mortality	434	364	518	4.9	
Zambia	Male	Mortality	116	81	166	4.3	
Afghanistan	Female	Mortality	341	262	443	3.4	
Albania	Female	Mortality	267	220	324	9.6	
Algeria	Female	Mortality	656	550	783	2.9	
Angola	Female	Mortality	109	109	109	1.5	
Azerbaijan	Female	Mortality	289	229	364	4.3	
Argentina	Female	Mortality	3641	3414	3883	9.8	
Australia	Female	Mortality	3920	3706	4147	13	
Austria	Female	Mortality	1753	1619	1898	15.4	
Bahamas	Female	Mortality	11	3	42	3.8	
Bahrain	Female	Mortality	21	7	61	4.7	
Bangladesh	Female	Mortality	2750	2626	2880	3.7	
Armenia	Female	Mortality	235	182	303	7.5	
Barbados	Female	Mortality	20	9	43	5.1	
Belgium	Female	Mortality	2214	2057	2383	15.6	
Bhutan	Female	Mortality	25	23	27	7.6	
Bolivia (Plurin	Female	Mortality	338	265	431	5.1	
Bosnia Herzeg	Female	Mortality	474	427	526	12.3	
Botswana	Female	Mortality	17	5	53	1.7	
Brazil	Female	Mortality	16777	16332	17234	9.9	
Belize	Female	Mortality	7	1	46	4	
Solomon Islar	Female	Mortality	7	5	10	3.2	
Brunei Daruss	Female	Mortality	40	22	71	18.4	
Bulgaria	Female	Mortality	897	803	1002	11.1	
Myanmar	Female	Mortality	3565	2996	4243	11.2	
Burundi	Female	Mortality	77	33	178	2.6	
Belarus	Female	Mortality	461	412	515	3.9	
Cambodia	Female	Mortality	694	675	714	8.8	
Cameroon	Female	Mortality	125	16	1004	1.9	
Canada	Female	Mortality	11011	10647	11388	19.1	
Cape Verde	Female	Mortality	4	1	21	1.4	
Central Africa	Female	Mortality	18	3	94	1.5	
Sri Lanka	Female	Mortality	515	432	615	2.9	
Chad	Female	Mortality	24	14	40	0.62	
Chile	Female	Mortality	1596	1457	1748	8.2	

China	Female	Mortality	217382	213519	221314	14.7	
Colombia	Female	Mortality	2592	2433	2762	6.5	
Comoros	Female	Mortality	3	1	7	1.1	
Congo, Reput	Female	Mortality	12	5	31	0.75	
Congo, Demo	Female	Mortality	371	61	2265	1.5	
Costa Rica	Female	Mortality	147	109	199	3.4	
Croatia	Female	Mortality	888	792	996	16.3	
Cuba	Female	Mortality	2407	2238	2589	18.1	
Cyprus	Female	Mortality	138	107	178	10.8	
Czechia	Female	Mortality	1886	1766	2014	13.5	
Benin	Female	Mortality	39	17	87	1	
Denmark	Female	Mortality	1842	1653	2053	23.6	
Dominican Re	Female	Mortality	615	531	712	9.1	
Ecuador	Female	Mortality	543	469	629	4.8	
El Salvador	Female	Mortality	179	137	235	3.7	
Equatorial Gu	Female	Mortality	9	4	20	2.5	
Ethiopia	Female	Mortality	1219	1072	1387	3.4	
Eritrea	Female	Mortality	32	21	49	2.6	
Estonia	Female	Mortality	176	140	222	7.7	
Fiji	Female	Mortality	22	11	45	4.6	
Finland	Female	Mortality	858	762	966	9.5	
France (metro	Female	Mortality	12047	11622	12487	14.8	
French Guyar	Female	Mortality	9	9	9	6.1	
French Polyne	Female	Mortality	35	34	36	18.1	
Djibouti	Female	Mortality	8	5	12	1.9	
Gabon	Female	Mortality	19	7	51	2.6	
Georgia	Female	Mortality	195	154	248	4	
The Republic	Female	Mortality	5	1	40	0.87	
Gaza Strip an	Female	Mortality	90	67	121	6	
Germany	Female	Mortality	18713	18102	19345	16.1	
Ghana	Female	Mortality	202	153	267	1.9	
Greece	Female	Mortality	1739	1606	1883	11.5	
France, Guad	Female	Mortality	27	27	27	5.7	
Guam	Female	Mortality	28	28	28	17.9	
Guatemala	Female	Mortality	237	193	291	3	
Guinea	Female	Mortality	82	32	210	1.9	
Guyana	Female	Mortality	11	3	35	2.4	

Haiti	Female	Mortality	287	228	360	5.7	
Honduras	Female	Mortality	195	159	239	4.5	
Hungary	Female	Mortality	3572	3407	3745	28.9	
Iceland	Female	Mortality	64	46	89	15	
India	Female	Mortality	20811	19935	21725	2.9	
Indonesia	Female	Mortality	8750	7395	10353	5.7	
Iran, Islamic F	Female	Mortality	3114	2863	3387	7	
Iraq	Female	Mortality	412	336	505	3.3	
Ireland	Female	Mortality	969	838	1121	17.5	
Israel	Female	Mortality	740	646	848	8.9	
Italy	Female	Mortality	12060	11628	12508	11.6	
Côte d'Ivoire	Female	Mortality	79	54	116	1.2	
Jamaica	Female	Mortality	164	103	262	7.9	
Japan	Female	Mortality	26338	25788	26900	7.1	
Kazakhstan	Female	Mortality	546	479	622	4.1	
Jordan	Female	Mortality	176	130	238	4.7	
Kenya	Female	Mortality	440	343	565	3	
Korea, Democ	Female	Mortality	5926	5784	6071	25	
Korea, Repub	Female	Mortality	5216	4993	5449	6.5	
Kuwait	Female	Mortality	42	26	69	3.8	
Kyrgyzstan	Female	Mortality	170	135	213	5.3	
Lao People's I	Female	Mortality	227	222	232	7.9	
Lebanon	Female	Mortality	503	409	619	12.7	
Lesotho	Female	Mortality	33	3	329	3.3	
Latvia	Female	Mortality	216	176	265	6.9	
Liberia	Female	Mortality	25	12	54	1.5	
Libya	Female	Mortality	91	60	139	3	
Lithuania	Female	Mortality	307	264	356	7.3	
Luxembourg	Female	Mortality	110	86	141	16.6	
Madagascar	Female	Mortality	119	43	327	1.4	
Malawi	Female	Mortality	50	16	155	0.97	
Malaysia	Female	Mortality	1092	912	1308	5.9	
Maldives	Female	Mortality	10	9	11	5.9	
Mali	Female	Mortality	100	62	160	2	
Malta	Female	Mortality	43	31	60	8.6	
France, Martir	Female	Mortality	28	28	28	4.9	
Mauritania	Female	Mortality	17	1	250	1.1	

Mauritius	Female	Mortality	57	36	90	4.5	
Mexico	Female	Mortality	2988	2797	3193	3.6	
Mongolia	Female	Mortality	81	64	102	5.4	
Republic of M	Female	Mortality	241	205	283	5.9	
Montenegro	Female	Mortality	99	78	126	15.4	
Morocco	Female	Mortality	834	729	955	3.7	
Mozambique	Female	Mortality	66	41	107	0.67	
Oman	Female	Mortality	30	14	65	2.7	
Namibia	Female	Mortality	36	17	76	4	
Nepal	Female	Mortality	818	762	878	5.7	
The Netherlar	Female	Mortality	4805	4583	5037	21.3	
New Caledoni	Female	Mortality	40	39	41	18.3	
Vanuatu	Female	Mortality	3	2	4	3.4	
New Zealand	Female	Mortality	987	882	1105	17.7	
Nicaragua	Female	Mortality	137	110	171	3.9	
Niger	Female	Mortality	5	0	74	0.09	
Nigeria	Female	Mortality	793	654	961	1.5	
Norway	Female	Mortality	1240	1132	1358	16.6	
Pakistan	Female	Mortality	1672	1524	1835	2.1	
Panama	Female	Mortality	110	78	155	3.5	
Papua New G	Female	Mortality	210	98	451	7.1	
Paraguay	Female	Mortality	144	114	181	3.8	
Peru	Female	Mortality	1314	1189	1453	5.9	
Philippines	Female	Mortality	6299	5951	6667	11.5	
Poland	Female	Mortality	9439	9110	9780	19.7	
Portugal	Female	Mortality	1391	1248	1550	8.9	
Guinea-Bissau	Female	Mortality	7	0	103	1.2	
Timor-Leste	Female	Mortality	26	23	29	5.5	
Puerto Rico	Female	Mortality	246	184	329	5.1	
Qatar	Female	Mortality	15	3	71	4.6	
France, La Ré	Female	Mortality	65	64	66	7.5	
Romania	Female	Mortality	2787	2597	2990	12.2	
Russian Fede	Female	Mortality	10638	10291	10997	5.9	
Rwanda	Female	Mortality	58	32	104	1.4	
Saint Lucia	Female	Mortality	4	1	29	3	
Sao Tome an	Female	Mortality	6	0	88	9.5	
Saudi Arabia	Female	Mortality	233	184	294	2.2	

Senegal	Female	Mortality	77	5	1133	1.4	
Serbia	Female	Mortality	1879	1740	2029	19.7	
Sierra Leone	Female	Mortality	1	0	11	0.02	
Singapore	Female	Mortality	848	702	1025	13.2	
Slovakia	Female	Mortality	686	580	811	10.3	
Viet Nam	Female	Mortality	6825	6499	7167	10	
Slovenia	Female	Mortality	513	454	580	18.5	
Somalia	Female	Mortality	117	94	146	3	
South Africa	Female	Mortality	3146	3128	3164	10.5	
Zimbabwe	Female	Mortality	161	112	232	3.6	
Spain	Female	Mortality	5979	5701	6271	10.6	
South Sudan	Female	Mortality	55	6	498	1.7	
Sudan	Female	Mortality	247	189	322	1.8	
Suriname	Female	Mortality	32	16	63	8.3	
Eswatini	Female	Mortality	10	3	36	2.4	
Sweden	Female	Mortality	2098	1974	2230	13.7	
Switzerland	Female	Mortality	1527	1418	1644	13	
Syrian Arab R	Female	Mortality	651	566	748	8.1	
Tajikistan	Female	Mortality	115	86	154	3.5	
Thailand	Female	Mortality	7011	6737	7296	9.6	
Togo	Female	Mortality	18	10	31	0.76	
Trinidad and T	Female	Mortality	55	35	87	4.3	
United Arab E	Female	Mortality	51	32	80	4.4	
Tunisia	Female	Mortality	321	251	410	3.9	
Türkiye	Female	Mortality	6386	6034	6759	10.1	
Turkmenistan	Female	Mortality	112	65	193	3.8	
Uganda	Female	Mortality	227	168	306	2.3	
Ukraine	Female	Mortality	2481	2352	2617	4.6	
North Macedo	Female	Mortality	245	180	334	12.5	
Egypt	Female	Mortality	1805	1609	2025	3.9	
United Kingdo	Female	Mortality	16855	16358	17367	17.4	
Tanzania, Uni	Female	Mortality	334	216	517	2	
United States	Female	Mortality	60807	59811	61819	14.7	
Burkina Faso	Female	Mortality	243	243	243	4.4	
Uruguay	Female	Mortality	434	362	521	12.7	
Uzbekistan	Female	Mortality	576	493	673	3.5	
Venezuela	Female	Mortality	1944	1778	2125	10.3	

Samoa	Female	Mortality	12	12	12	14.1	
Yemen	Female	Mortality	315	260	381	3.1	
Zambia	Female	Mortality	88	56	138	2	
Afghanistan	Female	Incidence	374	281	498	3.6	
Albania	Female	Incidence	304	257	359	10.3	
Algeria	Female	Incidence	729	619	859	3.2	
Angola	Female	Incidence	115	115	115	1.6	
Azerbaijan	Female	Incidence	346	262	457	5.2	
Argentina	Female	Incidence	4429	4023	4875	12.1	
Australia	Female	Incidence	6073	5450	6767	21.5	
Austria	Female	Incidence	2406	2233	2592	23.9	
Bahamas	Female	Incidence	15	2	137	5.3	
Bahrain	Female	Incidence	25	6	99	5.3	
Bangladesh	Female	Incidence	2967	2823	3119	4	
Armenia	Female	Incidence	283	190	422	9.1	
Barbados	Female	Incidence	24	8	71	6	
Belgium	Female	Incidence	3167	2969	3378	24.4	
Bhutan	Female	Incidence	25	23	27	7.6	
Bolivia (Plurin	Female	Incidence	394	301	517	6	
Bosnia Herze	Female	Incidence	574	506	652	15.7	
Botswana	Female	Incidence	17	7	42	1.7	
Brazil	Female	Incidence	19409	18661	20187	11.7	
Belize	Female	Incidence	7	1	68	4	
Solomon Islar	Female	Incidence	7	5	10	3.2	
Brunei Daruss	Female	Incidence	51	31	83	22.6	
Bulgaria	Female	Incidence	1050	936	1178	13.3	
Myanmar	Female	Incidence	3885	3214	4696	12.2	
Burundi	Female	Incidence	85	35	204	2.8	
Belarus	Female	Incidence	593	487	723	5.5	
Cambodia	Female	Incidence	787	762	812	9.9	
Cameroon	Female	Incidence	134	15	1228	1.9	
Canada	Female	Incidence	15257	14494	16060	30.4	
Cape Verde	Female	Incidence	6	3	14	2.5	
Central Africa	Female	Incidence	18	3	94	1.5	
Sri Lanka	Female	Incidence	563	480	660	3.1	
Chad	Female	Incidence	25	16	39	0.64	
Chile	Female	Incidence	1772	1557	2016	9.2	

China	Female	Incidence	401862	392725	411212	30.3	
Colombia	Female	Incidence	3018	2749	3313	7.6	
Comoros	Female	Incidence	3	1	7	1.1	
Congo, Repul	Female	Incidence	13	6	31	0.8	
Congo, Demo	Female	Incidence	404	56	2897	1.6	
Costa Rica	Female	Incidence	167	127	220	4.1	
Croatia	Female	Incidence	1157	1017	1317	21.6	
Cuba	Female	Incidence	2913	2599	3265	22.4	
Cyprus	Female	Incidence	169	110	261	14.6	
Czechia	Female	Incidence	2483	2316	2661	17.6	
Benin	Female	Incidence	44	21	94	1.1	
Denmark	Female	Incidence	2565	2350	2800	34.4	
Dominican Re	Female	Incidence	724	588	891	10.8	
Ecuador	Female	Incidence	684	545	858	6	
El Salvador	Female	Incidence	204	142	293	4.3	
Equatorial Gu	Female	Incidence	9	4	20	2.5	
Ethiopia	Female	Incidence	1260	1125	1411	3.5	
Eritrea	Female	Incidence	34	22	53	2.8	
Estonia	Female	Incidence	247	201	304	12.6	
Fiji	Female	Incidence	23	10	54	4.9	
Finland	Female	Incidence	1240	1158	1328	14.2	
France (metro	Female	Incidence	16801	16108	17523	24.7	
French Guyar	Female	Incidence	11	11	11	7.5	
French Polyne	Female	Incidence	43	42	44	22.6	
Djibouti	Female	Incidence	10	6	16	2.5	
Gabon	Female	Incidence	20	8	48	2.7	
Georgia	Female	Incidence	209	154	284	4.4	
The Republic	Female	Incidence	5	1	29	0.87	
Gaza Strip an	Female	Incidence	101	72	141	6.6	
Germany	Female	Incidence	25154	24112	26241	22	
Ghana	Female	Incidence	220	169	287	2	
Greece	Female	Incidence	2226	2018	2456	15.7	
France, Guad	Female	Incidence	35	34	36	7.2	
Guam	Female	Incidence	29	29	29	19.1	
Guatemala	Female	Incidence	252	193	330	3.2	
Guinea	Female	Incidence	81	34	192	1.9	
Guyana	Female	Incidence	11	3	45	2.4	

Haiti	Female	Incidence	329	257	422	6.6	
Honduras	Female	Incidence	206	155	274	4.9	
Hungary	Female	Incidence	4205	3955	4471	35	
Iceland	Female	Incidence	104	73	149	27.8	
India	Female	Incidence	22778	21875	23719	3.2	
Indonesia	Female	Incidence	9797	8149	11779	6.4	
Iran, Islamic R	Female	Incidence	3358	3148	3582	7.4	
Iraq	Female	Incidence	450	375	541	3.6	
Ireland	Female	Incidence	1529	1371	1705	30.4	
Israel	Female	Incidence	1188	985	1434	16.1	
Italy	Female	Incidence	15358	14654	16096	16.2	
Côte d'Ivoire	Female	Incidence	95	64	142	1.4	
Jamaica	Female	Incidence	195	101	378	9.4	
Japan	Female	Incidence	40983	39298	42740	15.8	
Kazakhstan	Female	Incidence	607	498	739	4.5	
Jordan	Female	Incidence	195	148	257	5.1	
Kenya	Female	Incidence	482	381	609	3.2	
Korea, Democ	Female	Incidence	6908	6716	7106	29.8	
Korea, Repub	Female	Incidence	10154	9782	10540	16.4	
Kuwait	Female	Incidence	52	32	83	4.3	
Kyrgyzstan	Female	Incidence	185	134	254	5.7	
Lao People's	Female	Incidence	266	259	273	9.1	
Lebanon	Female	Incidence	562	465	679	14.3	
Lesotho	Female	Incidence	37	3	487	3.7	
Latvia	Female	Incidence	242	197	298	9.2	
Liberia	Female	Incidence	25	12	54	1.5	
Libya	Female	Incidence	98	63	153	3.2	
Lithuania	Female	Incidence	390	330	461	10.4	
Luxembourg	Female	Incidence	150	111	203	21	
Madagascar	Female	Incidence	135	46	396	1.5	
Malawi	Female	Incidence	52	17	155	1	
Malaysia	Female	Incidence	1307	1072	1593	7.1	
Maldives	Female	Incidence	10	9	11	5.9	
Mali	Female	Incidence	102	68	152	2	
Malta	Female	Incidence	63	48	83	11.9	
France, Martin	Female	Incidence	35	34	36	6.1	
Mauritania	Female	Incidence	18	1	265	1.2	

Mauritius	Female	Incidence	63	45	89	5.3	
Mexico	Female	Incidence	3195	2913	3504	3.8	
Mongolia	Female	Incidence	96	67	137	6.4	
Republic of M	Female	Incidence	286	231	354	7.1	
Montenegro	Female	Incidence	109	82	145	17.2	
Morocco	Female	Incidence	941	828	1070	4.2	
Mozambique	Female	Incidence	68	44	105	0.68	
Oman	Female	Incidence	33	17	64	2.8	
Namibia	Female	Incidence	38	20	71	4.1	
Nepal	Female	Incidence	903	846	964	6.3	
The Netherlar	Female	Incidence	7013	6651	7394	33.9	
New Caledoni	Female	Incidence	55	53	57	25.8	
Vanuatu	Female	Incidence	3	2	4	3.4	
New Zealand	Female	Incidence	1137	1024	1262	22.2	
Nicaragua	Female	Incidence	144	108	193	4.2	
Niger	Female	Incidence	5	0	66	0.09	
Nigeria	Female	Incidence	863	718	1037	1.6	
Norway	Female	Incidence	1805	1639	1988	27.6	
Pakistan	Female	Incidence	1893	1724	2078	2.3	
Panama	Female	Incidence	133	79	223	4.3	
Papua New G	Female	Incidence	237	100	562	7.9	
Paraguay	Female	Incidence	170	122	236	4.5	
Peru	Female	Incidence	1476	1289	1690	6.7	
Philippines	Female	Incidence	7140	6747	7556	13	
Poland	Female	Incidence	11173	10694	11674	24.1	
Portugal	Female	Incidence	1902	1660	2180	13.3	
Guinea-Bissau	Female	Incidence	8	1	118	1.4	
Timor-Leste	Female	Incidence	28	25	31	6	
Puerto Rico	Female	Incidence	290	236	357	6.8	
Qatar	Female	Incidence	15	4	54	4.6	
France, La Ré	Female	Incidence	80	79	81	9.2	
Romania	Female	Incidence	3217	2938	3523	14.6	
Russian Fede	Female	Incidence	14284	14041	14531	8.6	
Rwanda	Female	Incidence	63	37	107	1.5	
Saint Lucia	Female	Incidence	5	0	74	3.4	
Sao Tome an	Female	Incidence	6	0	88	9.5	
Saudi Arabia	Female	Incidence	278	220	351	2.5	

Senegal	Female	Incidence	84	6	1236	1.5	
Serbia	Female	Incidence	2210	2004	2438	23.8	
Sierra Leone	Female	Incidence	1	0	8	0.02	
Singapore	Female	Incidence	959	853	1078	15.9	
Slovakia	Female	Incidence	801	644	996	12.3	
Viet Nam	Female	Incidence	7419	7098	7755	11	
Slovenia	Female	Incidence	687	594	794	25.7	
Somalia	Female	Incidence	126	100	158	3.1	
South Africa	Female	Incidence	3393	3378	3408	11.2	
Zimbabwe	Female	Incidence	176	127	244	3.9	
Spain	Female	Incidence	8387	7892	8912	16	
South Sudan	Female	Incidence	59	6	596	1.8	
Sudan	Female	Incidence	266	200	354	1.9	
Suriname	Female	Incidence	40	15	107	10.4	
Eswatini	Female	Incidence	10	4	27	2.4	
Sweden	Female	Incidence	2463	2299	2639	19.1	
Switzerland	Female	Incidence	2233	2030	2457	20.6	
Syrian Arab R	Female	Incidence	651	570	744	8.2	
Tajikistan	Female	Incidence	127	86	188	3.8	
Thailand	Female	Incidence	8294	7968	8634	11.7	
Togo	Female	Incidence	22	11	43	0.88	
Trinidad and T	Female	Incidence	65	34	123	5.1	
United Arab E	Female	Incidence	65	41	103	5.1	
Tunisia	Female	Incidence	366	281	476	4.5	
Türkiye	Female	Incidence	7993	7069	9037	13.5	
Turkmenistan	Female	Incidence	132	70	250	4.5	
Uganda	Female	Incidence	269	199	364	2.6	
Ukraine	Female	Incidence	3257	3095	3428	6.3	
North Macedo	Female	Incidence	277	187	411	14.3	
Egypt	Female	Incidence	2044	1806	2314	4.4	
United Kingdo	Female	Incidence	24989	24496	25491	28.5	
Tanzania, Uni	Female	Incidence	387	253	592	2.3	
United States	Female	Incidence	113690	111858	115552	30.4	
Burkina Faso	Female	Incidence	252	252	252	4.5	
Uruguay	Female	Incidence	532	430	658	16.4	
Uzbekistan	Female	Incidence	677	537	853	4.1	
Venezuela	Female	Incidence	2291	2017	2602	12.2	

Samoa	Female	Incidence	16	16	16	18.9	
Yemen	Female	Incidence	331	274	399	3.2	
Zambia	Female	Incidence	100	64	155	2.2	

Supplementary B:

Country	measure	sex	val	upper	lower	AAPC	
Afghanistan	DALYs	Male	68.52655	113.2866	43.10979	0.008369	
Afghanistan	DALYs	Female	40.51094	73.33626	23.38759	0.018754	
Albania	DALYs	Male	1562.778	2175.014	1281.463	0.030705	
Albania	DALYs	Female	484.299	626.9078	340.0444	0.03918	
Algeria	DALYs	Male	296.1588	464.0086	170.4307	0.032523	
Algeria	DALYs	Female	72.72535	101.5934	48.01949	0.034326	
Angola	DALYs	Male	140.1349	211.9248	68.77411	0.021268	
Angola	DALYs	Female	56.76281	79.14652	33.83266	0.035991	
Argentina	DALYs	Male	779.1651	880.2209	705.1256	-0.02503	
Argentina	DALYs	Female	426.6669	469.9787	382.9483	0.003203	
Armenia	DALYs	Male	1456.779	1622.794	1308.984	-0.01893	
Armenia	DALYs	Female	266.673	310.6698	225.3637	-0.01212	
Australia	DALYs	Male	839.0475	937.9166	753.0442	-0.01638	
Australia	DALYs	Female	629.666	677.5512	560.4661	-0.00318	
Austria	DALYs	Male	1304.65	1418.07	1211.424	-0.01282	
Austria	DALYs	Female	886.9035	965.5542	811.173	0.013255	
Azerbaijan	DALYs	Male	794.2714	1249.286	551.3039	0.007286	
Azerbaijan	DALYs	Female	184.9174	240.9829	141.6607	0.013104	
Bahamas	DALYs	Male	585.4609	697.8897	488.6026	0.007459	
Bahamas	DALYs	Female	270.3652	324.5828	220.0029	0.017162	
Bahrain	DALYs	Male	529.3295	738.3412	360.5115	0.05109	
Bahrain	DALYs	Female	186.0751	263.068	130.2376	0.016793	
Bangladesh	DALYs	Male	433.7188	654.4543	248.1857	0.050998	
Bangladesh	DALYs	Female	159.4839	236.5637	105.0859	0.07032	
Barbados	DALYs	Male	518.7039	628.5868	428.1867	0.019504	
Barbados	DALYs	Female	264.8206	313.0766	214.2612	0.030076	
Belarus	DALYs	Male	1933.533	2183.144	1675.607	0.002906	
Belarus	DALYs	Female	252.8726	282.4585	224.4956	0.013375	
Belgium	DALYs	Male	1705.164	1895.83	1568.218	-0.02472	
Belgium	DALYs	Female	904.4447	972.5249	823.1005	0.005817	

Belize	DALYs	Male	335.3896	386.178	280.1539	0.014211	
Belize	DALYs	Female	143.7446	174.5971	118.6084	0.020839	
Benin	DALYs	Male	28.61619	41.94093	19.44394	0.01162	
Benin	DALYs	Female	13.82188	18.8238	9.513189	0.025457	
Bhutan	DALYs	Male	205.9477	339.3344	125.462	0.036864	
Bhutan	DALYs	Female	167.5886	235.4278	108.9513	0.045075	
Bolivia (Plurin	DALYs	Male	377.8354	536.4326	258.0188	0.025133	
Bolivia (Plurin	DALYs	Female	222.8285	320.3961	156.4692	0.029734	
Bosnia Herze	DALYs	Male	2524.106	3332.395	2091.427	0.000802	
Bosnia Herze	DALYs	Female	853.8114	1035.977	676.4964	0.018595	
Botswana	DALYs	Male	168.1232	247.5185	114.8029	0.054255	
Botswana	DALYs	Female	57.57297	81.87012	36.71748	0.052847	
Brazil	DALYs	Male	498.5378	528.2257	472.0946	0.006877	
Brazil	DALYs	Female	374.1289	403.2459	343.1384	0.026883	
Brunei Daruss	DALYs	Male	568.4498	776.6971	425.4252	0.028705	
Brunei Daruss	DALYs	Female	522.2388	675.8611	388.248	0.036105	
Bulgaria	DALYs	Male	2039.474	2246.665	1865.105	-0.01191	
Bulgaria	DALYs	Female	641.2911	718.0993	574.5497	0.019758	
Burkina Faso	DALYs	Male	75.60442	109.4739	50.77219	0.01509	
Burkina Faso	DALYs	Female	33.34098	45.45678	22.7177	0.025932	
Burundi	DALYs	Male	97.88424	151.8755	53.14504	0.027439	
Burundi	DALYs	Female	41.28219	60.86971	24.95602	0.041749	
Côte d'Ivoire	DALYs	Male	65.43349	92.78702	45.01308	0.044102	
Côte d'Ivoire	DALYs	Female	51.70447	70.08816	34.69755	0.055793	
Cape Verde	DALYs	Male	376.4277	534.3754	255.4753	0.040656	
Cape Verde	DALYs	Female	256.0755	390.1573	174.9018	0.061275	
Cambodia	DALYs	Male	357.2174	545.191	250.3804	0.028949	
Cambodia	DALYs	Female	191.9602	274.6846	125.9123	0.031625	
Cameroon	DALYs	Male	111.9472	168.5349	69.36717	0.020036	
Cameroon	DALYs	Female	62.8202	87.77359	40.92752	0.036635	
Canada	DALYs	Male	1314.517	1440.522	1213.94	-0.00616	
Canada	DALYs	Female	1119.967	1192.094	1012.372	-0.00379	
Central Africa	DALYs	Male	75.5959	132.3947	36.81535	0.027229	
Central Africa	DALYs	Female	51.06231	93.96796	27.56305	0.028604	
Chad	DALYs	Male	84.25752	127.5978	56.7926	0.008327	
Chad	DALYs	Female	33.12878	47.02228	21.55439	0.015225	
Chile	DALYs	Male	514.8017	548.3133	481.7653	-0.00588	

Chile	DALYs	Female	378.7515	409.2039	342.5564	0.016585	
China	DALYs	Male	1673.07	2009.377	1356.451	0.018599	
China	DALYs	Female	731.2794	895.0794	624.5277	0.014968	
Colombia	DALYs	Male	271.7739	289.2798	254.4489	-0.0086	
Colombia	DALYs	Female	207.8861	221.2296	192.8129	0.003243	
Comoros	DALYs	Male	69.01926	106.4091	35.96321	0.020717	
Comoros	DALYs	Female	30.57796	44.42754	18.81308	0.010586	
Congo, Repul	DALYs	Male	160.6836	249.7438	76.07582	0.024473	
Congo, Repul	DALYs	Female	82.4937	118.1962	48.48801	0.029771	
Costa Rica	DALYs	Male	272.2377	307.7664	239.0027	0.013089	
Costa Rica	DALYs	Female	162.9202	190.6645	138.6911	0.027924	
Croatia	DALYs	Male	2384.406	2567.063	2202.516	-0.00581	
Croatia	DALYs	Female	1003.864	1119.291	871.6298	0.023876	
Cuba	DALYs	Male	1289.966	1484.62	1114.051	-0.00427	
Cuba	DALYs	Female	869.8974	1001.279	762.1998	0.007747	
Cyprus	DALYs	Male	1176.975	1521.962	859.2653	-0.00071	
Cyprus	DALYs	Female	350.4335	462.6422	262.249	0.006919	
Czechia	DALYs	Male	1442.346	1533.94	1362.679	-0.02872	
Czechia	DALYs	Female	758.3432	816.0556	681.0773	-0.00159	
Korea, Democ	DALYs	Male	1027.989	1570.003	678.1488	0.027316	
Korea, Democ	DALYs	Female	689.9152	992.565	444.8091	0.032901	
Congo, Demo	DALYs	Male	127.6675	225.3691	56.29061	0.033635	
Congo, Demo	DALYs	Female	90.62857	149.037	50.14972	0.052468	
Denmark	DALYs	Male	1580.129	1662.317	1494.033	-0.00893	
Denmark	DALYs	Female	1469.497	1636.424	1317.474	-0.00603	
Djibouti	DALYs	Male	203.1246	297.6584	110.6929	0.037699	
Djibouti	DALYs	Female	77.91616	108.5747	52.33032	0.047931	
Dominican Re	DALYs	Male	455.8798	595.2774	345.9209	0.034316	
Dominican Re	DALYs	Female	305.5991	386.5004	236.9445	0.038172	
Ecuador	DALYs	Male	154.0668	174.1082	137.0946	-0.02013	
Ecuador	DALYs	Female	140.9317	156.5742	129.4161	0.00048	
Egypt	DALYs	Male	435.3246	593.7618	312.6285	0.018535	
Egypt	DALYs	Female	171.7906	235.1764	121.693	0.037385	
El Salvador	DALYs	Male	302.602	424.7407	236.3852	0.029887	
El Salvador	DALYs	Female	280.1335	356.3669	227.5072	0.040138	
Equatorial Gu	DALYs	Male	195.7641	294.9174	88.09196	0.012853	
Equatorial Gu	DALYs	Female	130.1288	220.6112	68.95016	0.050918	

Eritrea	DALYs	Male	113.6257	171.9332	67.93596	0.039554	
Eritrea	DALYs	Female	79.01772	114.3725	50.02744	0.036328	
Estonia	DALYs	Male	1528.893	1658.067	1431.872	-0.02214	
Estonia	DALYs	Female	461.1968	530.1118	402.3304	0.006959	
Eswatini	DALYs	Male	76.33365	110.8957	51.40833	-0.00092	
Eswatini	DALYs	Female	63.87506	91.23533	42.25574	0.025048	
Ethiopia	DALYs	Male	199.1564	275.3908	138.6476	0.036501	
Ethiopia	DALYs	Female	54.4518	72.58369	37.2426	0.055503	
Fiji	DALYs	Male	349.0736	516.5413	246.3056	0.02428	
Fiji	DALYs	Female	210.8344	286.85	142.6913	0.028058	
Finland	DALYs	Male	1274.996	1393.082	1175.084	-0.00554	
Finland	DALYs	Female	763.9574	845.0029	674.2252	0.020232	
France (metropolita	DALYs	Male	1968.42	2186.471	1804.907	-0.00865	
France (metropolita	DALYs	Female	823.183	903.1381	739.2532	0.016001	
French Guyana	DALYs	Male	1968.42	2186.471	1804.907	-0.00865	
French Guyana	DALYs	Female	823.183	903.1381	739.2532	0.016001	
French Polynesia	DALYs	Male	1968.42	2186.471	1804.907	-0.00865	
French Polynesia	DALYs	Female	823.183	903.1381	739.2532	0.016001	
France, Guadeloup	DALYs	Male	1968.42	2186.471	1804.907	-0.00865	
France, Guadeloup	DALYs	Female	823.183	903.1381	739.2532	0.016001	
France, La Réunior	DALYs	Male	1968.42	2186.471	1804.907	-0.00865	
France, La Réunior	DALYs	Female	823.183	903.1381	739.2532	0.016001	
France, Martinique	DALYs	Male	1968.42	2186.471	1804.907	-0.00865	
France, Martinique	DALYs	Female	823.183	903.1381	739.2532	0.016001	
Gabon	DALYs	Male	241.9577	365.3226	126.5601	0.036124	
Gabon	DALYs	Female	150.3417	243.6988	82.78873	0.044217	
The Republic of the	DALYs	Male	47.73767	69.87103	29.13309	0.027263	
The Republic of the	DALYs	Female	15.25974	21.949	9.693068	0.046246	
Georgia	DALYs	Male	1843.427	2152.455	1573.821	0.002965	
Georgia	DALYs	Female	255.4275	301.5843	211.2147	-0.00776	
Germany	DALYs	Male	1763.086	1932.633	1643.865	-0.00577	
Germany	DALYs	Female	1077.314	1158.272	982.3008	0.017487	
Ghana	DALYs	Male	149.3138	214.8363	99.9105	0.034178	
Ghana	DALYs	Female	96.29664	143.0682	61.16732	0.05859	
Greece	DALYs	Male	2520.984	2756.301	2337.709	0.0029	
Greece	DALYs	Female	774.5835	860.2132	691.4591	0.025365	
Guam	DALYs	Male	1206.192	1392.173	1042.674	0.021108	

Guam	DALYs	Female	692.5899	821.6329	559.6481	0.029974	
Guatemala	DALYs	Male	102.1513	121.8933	82.31827	-0.00804	
Guatemala	DALYs	Female	96.88188	113.0985	81.19305	0.00406	
Guinea	DALYs	Male	110.9174	161.3538	74.89128	0.008761	
Guinea	DALYs	Female	44.22691	59.77568	29.04202	0.032558	
Guinea-Bissau	DALYs	Male	74.69612	110.8883	49.39749	0.027222	
Guinea-Bissau	DALYs	Female	49.96809	67.44903	34.25241	0.044423	
Guyana	DALYs	Male	260.8002	322.9268	207.8519	0.028735	
Guyana	DALYs	Female	202.011	253.5887	152.5116	0.053597	
Haiti	DALYs	Male	201.4356	309.9882	134.7769	0.026742	
Haiti	DALYs	Female	120.6558	179.0307	79.83252	0.045263	
Honduras	DALYs	Male	170.9547	241.0995	115.1033	0.017761	
Honduras	DALYs	Female	209.7153	301.7113	141.6972	0.023766	
Hungary	DALYs	Male	2382.285	2579.929	2222.459	-0.02479	
Hungary	DALYs	Female	1506.529	1586.857	1416.218	0.001117	
Iceland	DALYs	Male	809.5949	921.8463	720.166	-0.01205	
Iceland	DALYs	Female	1121.547	1273.611	975.727	0.005618	
India	DALYs	Male	271.0605	359.3222	207.276	0.035555	
India	DALYs	Female	119.3337	164.2164	87.61752	0.057859	
Indonesia	DALYs	Male	677.8321	968.0283	452.4941	0.032092	
Indonesia	DALYs	Female	292.9307	402.1804	199.3079	0.03979	
Iran, Islamic Repub	DALYs	Male	363.0584	459.0041	290.8926	0.015718	
Iran, Islamic Repub	DALYs	Female	164.9745	227.7329	119.4129	0.018536	
Iraq	DALYs	Male	626.5323	830.8707	454.7756	0.033259	
Iraq	DALYs	Female	256.1184	344.4763	189.8671	0.03669	
Ireland	DALYs	Male	1059.832	1197.857	961.5028	0.001848	
Ireland	DALYs	Female	896.9652	994.346	799.7744	0.016556	
Israel	DALYs	Male	747.6408	831.3524	659.0746	-0.00845	
Israel	DALYs	Female	357.6158	404.3546	313.7624	-0.00113	
Italy	DALYs	Male	1609.186	1750.659	1488.442	-0.01402	
Italy	DALYs	Female	741.9619	820.4379	644.1481	0.014983	
Jamaica	DALYs	Male	761.8337	913.2307	623.8279	0.022177	
Jamaica	DALYs	Female	250.4212	308.5638	200.2429	0.030338	
Japan	DALYs	Male	1878.764	2007.254	1701.332	0.008837	
Japan	DALYs	Female	721.5682	841.5996	568.3674	0.018299	
Jordan	DALYs	Male	456.8694	572.3128	363.3437	0.018553	
Jordan	DALYs	Female	131.1999	166.3497	105.743	0.019901	

Kazakhstan	DALYs	Male	691.9552	765.3214	633.5481	-0.02908	
Kazakhstan	DALYs	Female	129.1079	143.9752	113.2639	-0.03393	
Kenya	DALYs	Male	68.03314	97.59389	46.70504	0.027241	
Kenya	DALYs	Female	47.65927	67.17807	32.46932	0.037913	
Kuwait	DALYs	Male	142.0907	172.4034	116.5759	0.000355	
Kuwait	DALYs	Female	86.68121	103.9436	69.39879	0.01709	
Kyrgyzstan	DALYs	Male	323.5183	369.8046	279.8845	-0.01212	
Kyrgyzstan	DALYs	Female	100.2567	121.8615	84.4602	-0.00355	
Lao People's	DALYs	Male	508.286	728.2584	354.864	0.039888	
Lao People's	DALYs	Female	305.3781	424.7895	199.3122	0.06158	
Latvia	DALYs	Male	2013.415	2210.224	1870.565	-0.00699	
Latvia	DALYs	Female	458.5765	524.6018	401.1931	0.013806	
Lebanon	DALYs	Male	962.395	1239.182	752.9237	0.021225	
Lebanon	DALYs	Female	526.2455	657.891	399.6099	0.027558	
Lesotho	DALYs	Male	339.4275	550.7349	192.1073	0.037218	
Lesotho	DALYs	Female	111.0046	166.0989	66.15661	0.033509	
Liberia	DALYs	Male	129.255	201.4173	81.92634	0.032151	
Liberia	DALYs	Female	62.2599	92.73423	41.46254	0.051636	
Libya	DALYs	Male	708.7077	1088.531	456.1133	0.023429	
Libya	DALYs	Female	109.8145	153.69	71.40742	0.023986	
Lithuania	DALYs	Male	1683.055	1851.521	1569.487	-0.01792	
Lithuania	DALYs	Female	386.5055	426.7736	346.496	0.016435	
Luxembourg	DALYs	Male	1092.515	1237.614	974.8509	-0.03132	
Luxembourg	DALYs	Female	626.7321	723.8359	537.6806	-0.00263	
Madagascar	DALYs	Male	138.3291	205.2373	75.95003	0.023609	
Madagascar	DALYs	Female	69.63125	98.2685	43.02514	0.037392	
Malawi	DALYs	Male	61.92344	91.29467	41.06895	0.026546	
Malawi	DALYs	Female	30.97612	44.31637	19.9958	0.049842	
Malaysia	DALYs	Male	604.518	760.1931	499.7017	0.01631	
Malaysia	DALYs	Female	327.5441	418.8639	253.5867	0.025923	
Maldives	DALYs	Male	243.9227	323.4805	182.4412	-0.00276	
Maldives	DALYs	Female	146.6454	190.8146	107.3943	0.020089	
Mali	DALYs	Male	99.86558	142.4504	70.04014	0.014938	
Mali	DALYs	Female	45.20782	61.66773	31.4837	0.035879	
Malta	DALYs	Male	991.56	1142.803	859.1448	-0.02829	
Malta	DALYs	Female	357.1344	421.197	300.0337	0.000688	
Mauritania	DALYs	Male	196.1616	288.3072	126.372	0.015226	

Mauritania	DALYs	Female	86.1251	121.505	57.82257	0.039443	
Mauritius	DALYs	Male	508.7115	578.9891	448.0742	0.008706	
Mauritius	DALYs	Female	209.6334	243.7994	180.0425	0.018572	
Mexico	DALYs	Male	175.2919	192.4118	160.4885	-0.01382	
Mexico	DALYs	Female	121.3221	136.347	107.7614	0.004598	
Mongolia	DALYs	Male	812.6629	1167.54	611.3032	0.017903	
Mongolia	DALYs	Female	182.9335	242.217	137.249	0.007436	
Montenegro	DALYs	Male	2489.903	3140.075	1922.789	2.72E-05	
Montenegro	DALYs	Female	951.2032	1203.207	724.1722	0.007162	
Morocco	DALYs	Male	670.6403	1077.576	310.6381	0.05142	
Morocco	DALYs	Female	91.21099	129.8614	56.92223	0.059135	
Mozambique	DALYs	Male	59.44515	85.26061	39.16556	-0.00068	
Mozambique	DALYs	Female	13.21908	18.27897	8.87655	0.004943	
Myanmar	DALYs	Male	584.6824	883.897	394.1379	0.028982	
Myanmar	DALYs	Female	350.9452	472.801	236.5413	0.040895	
Namibia	DALYs	Male	100.9053	146.9546	67.96303	0.018608	
Namibia	DALYs	Female	68.49249	93.84464	45.05204	0.026082	
Nepal	DALYs	Male	207.3727	315.6982	133.9137	0.037031	
Nepal	DALYs	Female	103.882	149.095	69.46861	0.04692	
The Netherlands	DALYs	Male	1590.083	1743.719	1464.761	-0.01531	
The Netherlands	DALYs	Female	1263.049	1338.227	1161.541	0.00509	
New Zealand	DALYs	Male	912.3792	1031.374	833.5557	0.000137	
New Zealand	DALYs	Female	947.1682	1075.952	844.9866	0.014436	
Nicaragua	DALYs	Male	129.6759	169.0196	99.16124	0.004118	
Nicaragua	DALYs	Female	107.4343	143.584	84.59774	0.019321	
Niger	DALYs	Male	24.4622	36.56056	15.90494	0.012607	
Niger	DALYs	Female	9.227316	12.74685	6.186558	0.026483	
Nigeria	DALYs	Male	109.1941	163.0109	66.7611	0.011715	
Nigeria	DALYs	Female	52.65083	75.43955	35.61557	0.038411	
North Macedo	DALYs	Male	2133.2	2486.392	1712.519	0.007666	
North Macedo	DALYs	Female	646.1271	797.5144	476.5712	0.025639	
Norway	DALYs	Male	979.3193	1076.472	888.3209	-0.01802	
Norway	DALYs	Female	833.7322	936.1592	742.4547	-0.00613	
Oman	DALYs	Male	141.8283	208.8002	97.95018	-0.00602	
Oman	DALYs	Female	57.96386	78.63665	39.94916	0.007742	
Pakistan	DALYs	Male	133.6769	221.2662	87.03482	0.016177	
Pakistan	DALYs	Female	128.2653	183.0684	88.41803	0.031201	

Gaza Strip and We	DALYs	Male	399.5056	536.9938	313.2924	0.021163	
Gaza Strip and We	DALYs	Female	101.8984	129.4758	76.67123	0.020118	
Panama	DALYs	Male	234.2004	270.6297	203.5843	-0.01751	
Panama	DALYs	Female	132.7813	156.1419	110.4467	-0.00173	
Papua New G	DALYs	Male	438.183	682.07	252.4489	0.019834	
Papua New G	DALYs	Female	239.7508	371.3456	151.7635	0.031222	
Paraguay	DALYs	Male	510.1146	598.1314	336.801	0.012971	
Paraguay	DALYs	Female	192.3212	242.7983	149.1574	0.024426	
Peru	DALYs	Male	252.3504	329.5501	204.4795	0.014483	
Peru	DALYs	Female	267.1644	326.682	222.5633	0.028996	
Philippines	DALYs	Male	353.8917	480.6009	291.1396	-0.0067	
Philippines	DALYs	Female	183.9085	234.5923	146.7409	0.011084	
Poland	DALYs	Male	2087.833	2220.792	1969.483	-0.01867	
Poland	DALYs	Female	1090.626	1168.234	1017.02	0.012472	
Portugal	DALYs	Male	1663.51	1821.955	1520.865	0.003909	
Portugal	DALYs	Female	497.7318	553.8288	438.3073	0.024168	
Puerto Rico	DALYs	Male	598.1677	663.6199	548.528	-0.0026	
Puerto Rico	DALYs	Female	355.1871	408.9055	317.4218	0.013271	
Qatar	DALYs	Male	101.3229	159.8944	66.38134	0.010302	
Qatar	DALYs	Female	46.16249	69.61307	31.8405	0.000975	
Korea, Republic of	DALYs	Male	1317.053	1538.078	1098.18	0.010602	
Korea, Republic of	DALYs	Female	476.1593	586.9031	376.4219	0.011369	
Republic of M	DALYs	Male	1294.533	1450.838	1173.709	-0.00099	
Republic of M	DALYs	Female	254.2737	301.1965	218.7094	0.00249	
Romania	DALYs	Male	2083.536	2331.076	1899.572	-0.00234	
Romania	DALYs	Female	641.3023	738.298	559.2325	0.021478	
Russian Fede	DALYs	Male	1545.835	1654.877	1443.946	-0.0139	
Russian Fede	DALYs	Female	301.3426	344.605	260.9961	0.00127	
Rwanda	DALYs	Male	239.2754	359.2735	119.5661	0.049841	
Rwanda	DALYs	Female	98.50951	142.5168	58.98942	0.054824	
Saint Lucia	DALYs	Male	404.6054	491.2546	325.6203	0.006288	
Saint Lucia	DALYs	Female	235.5962	281.6981	189.978	0.028303	
Samoa	DALYs	Male	325.3922	497.6505	148.5317	0.038324	
Samoa	DALYs	Female	47.26038	65.61688	32.35249	0.030879	
Sao Tome and	DALYs	Male	382.6935	575.9464	262.3981	0.027745	
Sao Tome and	DALYs	Female	141.8642	210.9895	91.07517	0.039765	
Saudi Arabia	DALYs	Male	179.8585	285.797	136.9567	-0.02404	

Saudi Arabia	DALYs	Female	90.76934	132.8027	65.06846	0.014583	
Senegal	DALYs	Male	84.26593	125.6345	57.73054	0.011646	
Senegal	DALYs	Female	23.71684	31.80127	15.9884	0.041689	
Serbia	DALYs	Male	2476.949	3011.335	2036.079	-0.01419	
Serbia	DALYs	Female	1204.302	1482.693	939.7908	0.012347	
Sierra Leone	DALYs	Male	128.7811	190.4819	89.06969	0.021798	
Sierra Leone	DALYs	Female	36.20689	48.44293	25.07319	0.031534	
Singapore	DALYs	Male	543.1309	736.705	381.7997	-0.01668	
Singapore	DALYs	Female	394.4683	440.2029	343.9522	0.008121	
Slovakia	DALYs	Male	1622.11	2230.585	1444.035	-0.00681	
Slovakia	DALYs	Female	587.2338	687.1702	473.9184	0.012034	
Slovenia	DALYs	Male	1681.479	1860.618	1544.664	-0.01387	
Slovenia	DALYs	Female	901.6272	995.7722	808.2291	0.016479	
Solomon Islar	DALYs	Male	617.6487	919.9604	374.3749	0.042066	
Solomon Islar	DALYs	Female	748.6262	1037.208	504.9242	0.056205	
Somalia	DALYs	Male	44.18457	67.32933	25.45223	0.015311	
Somalia	DALYs	Female	19.57188	30.41817	11.61874	0.022887	
South Africa	DALYs	Male	667.5917	831.1472	528.7007	0.011188	
South Africa	DALYs	Female	276.9158	331.7403	223.3256	0.016801	
South Sudan	DALYs	Male	174.0652	261.9349	96.92323	0.029945	
South Sudan	DALYs	Female	73.88009	103.1289	45.86684	0.03693	
Spain	DALYs	Male	1753.851	1888.315	1628.112	-0.00492	
Spain	DALYs	Female	607.5278	660.3069	548.9625	0.03204	
Sri Lanka	DALYs	Male	687.9415	913.684	513.5485	0.038663	
Sri Lanka	DALYs	Female	258.6066	356.481	183.6256	0.052002	
Sudan	DALYs	Male	256.6945	422.7247	145.8556	0.023726	
Sudan	DALYs	Female	130.6349	189.6794	80.06943	0.039254	
Suriname	DALYs	Male	458.5856	613.3658	330.8489	0.006092	
Suriname	DALYs	Female	286.526	395.9589	192.4568	0.023295	
Sweden	DALYs	Male	679.3398	745.2438	624.0997	-0.02485	
Sweden	DALYs	Female	803.1484	859.743	716.6372	-0.00447	
Switzerland	DALYs	Male	1051.662	1141.742	958.1581	-0.01669	
Switzerland	DALYs	Female	678.3818	755.0835	597.0613	-0.00176	
Syrian Arab R	DALYs	Male	411.7052	569.675	299.7398	0.040277	
Syrian Arab R	DALYs	Female	129.2134	179.8549	91.52695	0.03174	
Türkiye	DALYs	Male	1620.678	2068.718	1084.291	0.018026	
Türkiye	DALYs	Female	321.5969	418.7179	244.0254	0.029469	

Tajikistan	DALYs	Male	177.9125	302.9797	130.0705	0.01473	
Tajikistan	DALYs	Female	121.8123	185.6523	87.50322	0.04526	
Thailand	DALYs	Male	1427.394	2016.986	1097.169	0.024754	
Thailand	DALYs	Female	630.8309	838.9282	490.1791	0.016865	
Timor-Leste	DALYs	Male	309.8058	521.4029	202.4293	0.015888	
Timor-Leste	DALYs	Female	195.0605	286.3136	121.4211	0.038148	
Togo	DALYs	Male	131.6336	184.4076	84.14727	0.039742	
Togo	DALYs	Female	63.07469	90.51211	42.73342	0.053061	
Trinidad and T	DALYs	Male	561.9387	651.8949	473.8017	0.018341	
Trinidad and T	DALYs	Female	207.7855	247.9067	174.2748	0.024736	
Tunisia	DALYs	Male	1656.496	2210.847	1282.093	0.026592	
Tunisia	DALYs	Female	305.7248	394.9189	225.4772	0.045409	
Turkmenistan	DALYs	Male	306.0554	369.4057	255.9673	0.015931	
Turkmenistan	DALYs	Female	112.013	136.2444	90.96137	0.024104	
Uganda	DALYs	Male	102.6395	142.0979	69.68696	0.016152	
Uganda	DALYs	Female	86.98822	129.7738	56.74639	0.045592	
Ukraine	DALYs	Male	1558.227	1834.225	1324.522	-0.00299	
Ukraine	DALYs	Female	272.6701	315.1719	232.3381	0.004629	
United Arab E	DALYs	Male	157.9129	229.7287	111.4114	0.044998	
United Arab E	DALYs	Female	78.51065	107.3019	54.62922	0.021711	
United Kingdo	DALYs	Male	1237.619	1295.99	1189.18	-0.01051	
United Kingdo	DALYs	Female	1081.492	1154.016	987.3521	0.000362	
Tanzania, United R	DALYs	Male	37.71209	54.01032	25.42167	0.006024	
Tanzania, United R	DALYs	Female	14.35077	19.71028	9.714679	0.004217	
United States	DALYs	Male	1049.095	1167.53	968.4065	-0.02363	
United States	DALYs	Female	911.5089	990.2232	833.364	-0.01305	
Uruguay	DALYs	Male	1887.449	2078.621	1689.627	-2.70E-06	
Uruguay	DALYs	Female	756.0995	860.1662	682.4517	0.037192	
Uzbekistan	DALYs	Male	261.3868	280.933	237.9221	0.019515	
Uzbekistan	DALYs	Female	97.01999	115.0073	81.90786	0.013526	
Venezuela	DALYs	Male	694.1157	824.1594	576.1719	0.033542	
Venezuela	DALYs	Female	392.984	559.1896	302.2801	0.028838	
Viet Nam	DALYs	Male	987.7227	1306.444	607.4361	0.043391	
Viet Nam	DALYs	Female	429.5907	602.666	287.5831	0.038818	
Yemen	DALYs	Male	176.4385	274.6472	114.446	0.004383	
Yemen	DALYs	Female	55.83857	87.84703	34.3101	0.021017	
Zambia	DALYs	Male	47.98946	69.81994	32.28436	0.016155	

Zambia	DALYs	Female	41.77592	59.73459	29.65798	0.030955	
Zimbabwe	DALYs	Male	289.5782	403.1862	202.8126	0.009473	
Zimbabwe	DALYs	Female	98.09954	133.0422	66.44316	0.020013	
Vanuatu	DALYs	Male	186.1438	306.9409	111.3054	0.016232	
Vanuatu	DALYs	Female	133.4756	204.5572	80.63093	0.03833	
New Caledonia	DALYs	Male	1968.42	2186.471	1804.907	-0.00865	
New Caledonia	DALYs	Female	823.183	903.1381	739.2532	0.016001	

Supplementary C:

Measure	Metric	Location	Sex	Value	Upper	Lower	
Incidence	Rate	High-middle S	Both sexes	29.68	33.74	26.41	
Incidence	Rate	Middle SDI	Both sexes	16.19	19.04	13.93	
Incidence	Rate	Low-middle S	Both sexes	8.49	10.7	6.98	
Incidence	Rate	Low SDI	Both sexes	4.2	5.37	3.28	
Incidence	Number	High-middle S	Both sexes	486,167.63	552,769.47	432,703.80	
Incidence	Number	Middle SDI	Both sexes	156,075.19	183,574.55	134,278.40	
Incidence	Number	Low-middle S	Both sexes	100,599.28	126,776.73	82,668.18	
Incidence	Number	Low SDI	Both sexes	72,661.11	92,865.19	56,753.04	
Deaths	Rate	High-middle S	Both sexes	27.2	30.67	24.05	
Deaths	Rate	Middle SDI	Both sexes	15.47	18.07	13.25	
Deaths	Rate	Low-middle S	Both sexes	8.32	10.49	6.8	
Deaths	Rate	Low SDI	Both sexes	4.09	5.24	3.19	
Deaths	Number	High-middle S	Both sexes	445,689.36	502,472.24	394,012.20	
Deaths	Number	Middle SDI	Both sexes	149,119.61	174,252.29	127,745.09	
Deaths	Number	Low-middle S	Both sexes	98,560.05	124,333.05	80,521.98	
Deaths	Number	Low SDI	Both sexes	70,717.91	90,618.95	55,141.70	
DALYs	Rate	High-middle S	Both sexes	643.67	725.91	573.56	
DALYs	Rate	Middle SDI	Both sexes	423.14	494.04	360.45	
DALYs	Rate	Low-middle S	Both sexes	228.83	288.29	186.94	
DALYs	Rate	Low SDI	Both sexes	119.5	152.21	93.65	
DALYs	Number	High-middle S	Both sexes	##########	##########	9,396,430.44	
DALYs	Number	Middle SDI	Both sexes	4,079,445.58	4,763,037.10	3,475,042.63	
DALYs	Number	Low-middle S	Both sexes	2,711,323.55	3,415,904.99	2,215,060.38	
DALYs	Number	Low SDI	Both sexes	2,068,291.05	2,634,515.90	1,620,989.97	

Supplementary D:							
Measure	Metric	Location	Sex	Value	Upper	Lower	
Deaths	Number	Southeast Asi	Both sexes	924,437.46	1,055,239.03	807,210.95	
Deaths	Rate	Southeast Asi	Both sexes	41.85	47.77	36.54	
Deaths	Number	Central Europ	Both sexes	160,247.22	165,614.10	153,531.14	
Deaths	Rate	Central Europ	Both sexes	37.88	39.14	36.29	
Deaths	Number	High-income	Both sexes	609,314.36	650,550.55	551,125.38	
Deaths	Rate	High-income	Both sexes	55.21	58.95	49.94	
Deaths	Number	Sub-Saharan	Both sexes	42,628.94	53,514.73	33,637.04	
Deaths	Rate	Sub-Saharan	Both sexes	3.53	4.43	2.79	
Deaths	Number	North Africa a	Both sexes	89,148.54	106,369.02	71,267.72	
Deaths	Rate	North Africa a	Both sexes	13.97	16.67	11.17	
Deaths	Number	South Asia	c	131,131.03	160,598.03	105,106.56	
Deaths	Rate	South Asia	Both sexes	6.93	8.48	5.55	
Deaths	Number	Latin America	Both sexes	80,235.17	83,592.26	75,487.58	
Deaths	Rate	Latin America	Both sexes	13.55	14.12	12.75	
DALYs (Disab	Number	Southeast Asi	Both sexes	##########	##########	##########	
DALYs (Disab	Rate	Southeast Asi	Both sexes	979.11	1,107.94	853.64	
DALYs (Disab	Number	High-income	Both sexes	##########	##########	##########	
DALYs (Disab	Rate	High-income	Both sexes	1,086.39	1,151.88	1,011.82	
DALYs (Disab	Number	South Asia	Both sexes	3,724,373.32	4,573,531.27	2,984,206.45	
DALYs (Disab	Rate	South Asia	Both sexes	196.74	241.6	157.64	
DALYs (Disab	Number	Latin America	Both sexes	1,879,804.25	1,955,398.14	1,791,598.11	
DALYs (Disab	Rate	Latin America	Both sexes	317.49	330.26	302.59	
DALYs (Disab	Number	Sub-Saharan	Both sexes	1,241,937.01	1,566,990.32	983,715.61	
DALYs (Disab	Rate	Sub-Saharan	Both sexes	102.91	129.85	81.51	
DALYs (Disab	Number	Central Europ	Both sexes	3,884,576.80	4,005,844.66	3,742,027.59	
DALYs (Disab	Rate	Central Europ	Both sexes	918.14	946.81	884.45	
DALYs (Disab	Number	North Africa a	Both sexes	2,338,424.14	2,814,693.54	1,861,725.42	
DALYs (Disab	Rate	North Africa a	Both sexes	366.48	441.12	291.77	
Incidence	Number	Southeast Asi	Both sexes	1,036,658.92	1,198,483.64	898,051.02	
Incidence	Rate	Southeast Asi	Both sexes	46.93	54.25	40.65	
Incidence	Number	Central Europ	Both sexes	170,445.26	177,613.75	161,782.69	
Incidence	Rate	Central Europ	Both sexes	40.29	41.98	38.24	
Incidence	Number	South Asia	Both sexes	134,706.29	165,126.29	107,612.47	
Incidence	Rate	South Asia	Both sexes	7.12	8.72	5.68	
Incidence	Number	Latin America	Both sexes	82,723.45	86,882.37	77,650.33	

Incidence	Rate	Latin America	Both sexes	13.97	14.67	13.11	
Incidence	Number	High-income	Both sexes	755,144.97	815,005.00	680,693.00	
Incidence	Rate	High-income	Both sexes	68.43	73.85	61.68	
Incidence	Number	Sub-Saharan	Both sexes	43,559.46	54,694.17	34,463.92	
Incidence	Rate	Sub-Saharan	Both sexes	3.61	4.53	2.86	
Incidence	Number	North Africa a	Both sexes	93,928.18	113,142.70	75,256.15	
Incidence	Rate	North Africa a	Both sexes	14.72	17.73	11.79	

Supplementary E:

Clean_country	measure_name	sex_name	year	val	upper	lower	
Afghanistan	Incidence	Male	2010	2.408993	3.684879	1.553247	
Afghanistan	Incidence	Male	2011	2.419929	3.63198	1.613789	
Afghanistan	Incidence	Male	2012	2.42398	3.657338	1.61208	
Afghanistan	Incidence	Male	2013	2.37	3.657351	1.593134	
Afghanistan	Incidence	Male	2014	2.360429	3.649894	1.555881	
Afghanistan	Incidence	Male	2015	2.328208	3.713478	1.502993	
Afghanistan	Incidence	Male	2016	2.297474	3.613739	1.433053	
Afghanistan	Incidence	Male	2017	2.261949	3.444174	1.420956	
Afghanistan	Incidence	Male	2018	2.26619	3.475844	1.442283	
Afghanistan	Incidence	Male	2019	2.239941	3.67911	1.444372	
Afghanistan	Incidence	Male	2020	2.186272	3.498986	1.424477	
Afghanistan	Incidence	Male	2021	2.397829	3.832922	1.554116	
Afghanistan	Incidence	Male	2022	2.323167	3.611587	1.5154	
Afghanistan	Incidence	Male	2023	2.499329	4.079538	1.564685	
Afghanistan	Incidence	Female	2010	1.131796	2.036424	0.689045	
Afghanistan	Incidence	Female	2011	1.147536	2.165028	0.704686	
Afghanistan	Incidence	Female	2012	1.154781	2.134384	0.70418	
Afghanistan	Incidence	Female	2013	1.164805	2.114378	0.694124	
Afghanistan	Incidence	Female	2014	1.182819	2.074748	0.699854	
Afghanistan	Incidence	Female	2015	1.194324	2.008488	0.682198	
Afghanistan	Incidence	Female	2016	1.191303	2.002739	0.670935	
Afghanistan	Incidence	Female	2017	1.199165	2.037033	0.688956	
Afghanistan	Incidence	Female	2018	1.207246	2.050612	0.702596	
Afghanistan	Incidence	Female	2019	1.233926	2.087887	0.728536	
Afghanistan	Incidence	Female	2020	1.243657	2.107313	0.723761	

Afghanistan	Incidence	Female	2021	1.364775	2.394377	0.792516	
Afghanistan	Incidence	Female	2022	1.314174	2.329753	0.757768	
Afghanistan	Incidence	Female	2023	1.376672	2.44907	0.810901	
Afghanistan	Deaths	Male	2010	2.517639	3.846699	1.632263	
Afghanistan	Deaths	Male	2011	2.529491	3.827079	1.683374	
Afghanistan	Deaths	Male	2012	2.532616	3.815036	1.693786	
Afghanistan	Deaths	Male	2013	2.475082	3.823388	1.667874	
Afghanistan	Deaths	Male	2014	2.462295	3.825809	1.632327	
Afghanistan	Deaths	Male	2015	2.42341	3.850149	1.572403	
Afghanistan	Deaths	Male	2016	2.387415	3.744822	1.489187	
Afghanistan	Deaths	Male	2017	2.344215	3.54261	1.473386	
Afghanistan	Deaths	Male	2018	2.341324	3.567065	1.496271	
Afghanistan	Deaths	Male	2019	2.303456	3.752498	1.48916	
Afghanistan	Deaths	Male	2020	2.251896	3.611295	1.454815	
Afghanistan	Deaths	Male	2021	2.449045	3.842489	1.581313	
Afghanistan	Deaths	Male	2022	2.382927	3.692342	1.547274	
Afghanistan	Deaths	Male	2023	2.56012	4.134728	1.606099	
Afghanistan	Deaths	Female	2010	1.128095	2.036155	0.694482	
Afghanistan	Deaths	Female	2011	1.142658	2.141169	0.709332	
Afghanistan	Deaths	Female	2012	1.147801	2.131573	0.69725	
Afghanistan	Deaths	Female	2013	1.155664	2.083955	0.693274	
Afghanistan	Deaths	Female	2014	1.171432	2.034965	0.698032	
Afghanistan	Deaths	Female	2015	1.179309	1.981324	0.679959	
Afghanistan	Deaths	Female	2016	1.173637	1.961833	0.663582	
Afghanistan	Deaths	Female	2017	1.178364	1.981321	0.681727	
Afghanistan	Deaths	Female	2018	1.183132	1.993393	0.691083	
Afghanistan	Deaths	Female	2019	1.206249	2.040261	0.717857	
Afghanistan	Deaths	Female	2020	1.215202	2.063612	0.710498	
Afghanistan	Deaths	Female	2021	1.323512	2.319302	0.771737	
Afghanistan	Deaths	Female	2022	1.279181	2.283591	0.740785	
Afghanistan	Deaths	Female	2023	1.341437	2.376087	0.796096	
Afghanistan	DALYs	Male	2010	61.49052	95.44983	38.98839	
Afghanistan	DALYs	Male	2011	62.06961	93.33469	40.88543	
Afghanistan	DALYs	Male	2012	62.5661	94.32259	40.94663	
Afghanistan	DALYs	Male	2013	61.45345	94.87683	40.68282	
Afghanistan	DALYs	Male	2014	61.61863	96.43786	40.11035	

Afghanistan	DALYs	Male	2015	61.25148	100.4134	39.00638	
Afghanistan	DALYs	Male	2016	60.84528	97.60189	37.63866	
Afghanistan	DALYs	Male	2017	60.31123	92.842	37.40627	
Afghanistan	DALYs	Male	2018	60.96938	96.25822	38.4228	
Afghanistan	DALYs	Male	2019	60.89819	101.7236	38.69509	
Afghanistan	DALYs	Male	2020	59.16784	95.09391	38.20559	
Afghanistan	DALYs	Male	2021	66.13943	108.1951	43.55042	
Afghanistan	DALYs	Male	2022	63.71613	101.1034	41.10684	
Afghanistan	DALYs	Male	2023	68.52655	113.2866	43.10979	
Afghanistan	DALYs	Female	2010	31.81769	57.12411	19.0619	
Afghanistan	DALYs	Female	2011	32.41128	60.90728	19.66021	
Afghanistan	DALYs	Female	2012	32.85041	61.48641	19.56631	
Afghanistan	DALYs	Female	2013	33.30814	61.11262	19.46404	
Afghanistan	DALYs	Female	2014	34.0051	60.72748	19.42854	
Afghanistan	DALYs	Female	2015	34.53314	58.6867	19.46293	
Afghanistan	DALYs	Female	2016	34.58541	58.60352	19.55234	
Afghanistan	DALYs	Female	2017	34.93377	59.99774	19.98835	
Afghanistan	DALYs	Female	2018	35.36919	61.63961	20.453	
Afghanistan	DALYs	Female	2019	36.31944	61.56083	21.28026	
Afghanistan	DALYs	Female	2020	36.3482	61.47296	21.20847	
Afghanistan	DALYs	Female	2021	40.39645	70.54609	23.35701	
Afghanistan	DALYs	Female	2022	38.77205	69.51789	22.06743	
Afghanistan	DALYs	Female	2023	40.51094	73.33626	23.38759	
Albania	Incidence	Male	2010	43.32999	61.25469	36.79743	
Albania	Incidence	Male	2011	45.08955	63.87375	38.12424	
Albania	Incidence	Male	2012	47.27311	66.86506	39.84483	
Albania	Incidence	Male	2013	49.81119	68.88326	42.05832	
Albania	Incidence	Male	2014	52.78666	74.30877	44.95416	
Albania	Incidence	Male	2015	55.53484	78.30074	46.924	
Albania	Incidence	Male	2016	58.35431	81.67984	49.07405	
Albania	Incidence	Male	2017	60.87624	84.37579	50.95552	
Albania	Incidence	Male	2018	63.17181	86.7034	52.80169	
Albania	Incidence	Male	2019	67.26238	92.41154	56.60798	
Albania	Incidence	Male	2020	70.32921	97.41647	60.54117	
Albania	Incidence	Male	2021	76.62271	109.804	66.14417	
Albania	Incidence	Male	2022	73.67319	102.3559	62.58324	

Albania	Incidence	Male	2023	67.2192	94.60458	55.2493	
Albania	Incidence	Female	2010	11.97032	14.39602	9.088429	
Albania	Incidence	Female	2011	12.44053	15.12574	9.14625	
Albania	Incidence	Female	2012	13.20429	16.08999	9.444982	
Albania	Incidence	Female	2013	14.18503	17.49976	10.00763	
Albania	Incidence	Female	2014	15.31985	18.56633	10.717	
Albania	Incidence	Female	2015	16.50676	20.46151	11.32081	
Albania	Incidence	Female	2016	17.66296	21.71258	11.88798	
Albania	Incidence	Female	2017	18.91896	23.16897	12.8733	
Albania	Incidence	Female	2018	19.20038	23.73433	12.98183	
Albania	Incidence	Female	2019	20.03153	24.35733	13.72526	
Albania	Incidence	Female	2020	20.42842	24.59185	14.97383	
Albania	Incidence	Female	2021	21.88128	26.42796	16.67035	
Albania	Incidence	Female	2022	23.16452	29.04766	16.17878	
Albania	Incidence	Female	2023	21.41549	27.5235	15.09762	
Albania	Deaths	Male	2010	43.26175	61.32444	37.18091	
Albania	Deaths	Male	2011	45.0817	64.25454	38.05034	
Albania	Deaths	Male	2012	47.33448	67.34003	39.84099	
Albania	Deaths	Male	2013	49.93731	69.47961	42.29167	
Albania	Deaths	Male	2014	52.9412	75.07238	45.19238	
Albania	Deaths	Male	2015	55.71666	79.24824	47.25179	
Albania	Deaths	Male	2016	58.54281	83.00907	48.9621	
Albania	Deaths	Male	2017	61.01844	85.75402	50.95957	
Albania	Deaths	Male	2018	63.18479	87.64051	52.66851	
Albania	Deaths	Male	2019	66.97305	93.23439	56.49944	
Albania	Deaths	Male	2020	70.0242	97.18547	60.37547	
Albania	Deaths	Male	2021	76.15261	108.9278	65.67656	
Albania	Deaths	Male	2022	73.21058	102.2752	62.29382	
Albania	Deaths	Male	2023	66.90657	93.88109	54.9761	
Albania	Deaths	Female	2010	11.93947	14.47442	9.0478	
Albania	Deaths	Female	2011	12.44179	15.24779	9.038329	
Albania	Deaths	Female	2012	13.23319	16.20346	9.395694	
Albania	Deaths	Female	2013	14.23437	17.53691	9.98388	
Albania	Deaths	Female	2014	15.37876	18.65234	10.59367	
Albania	Deaths	Female	2015	16.59626	20.5465	11.18223	
Albania	Deaths	Female	2016	17.78267	21.96103	11.93006	

Albania	Deaths	Female	2017	19.08141	23.39726	12.9343	
Albania	Deaths	Female	2018	19.30159	23.9	13.11549	
Albania	Deaths	Female	2019	20.09934	24.50343	13.68062	
Albania	Deaths	Female	2020	20.41034	24.43881	14.95025	
Albania	Deaths	Female	2021	21.84669	26.38232	16.69395	
Albania	Deaths	Female	2022	23.182	28.76706	16.08609	
Albania	Deaths	Female	2023	21.44997	27.33276	15.22152	
Albania	DALYs	Male	2010	1054.748	1464.4	895.183	
Albania	DALYs	Male	2011	1093.948	1531.83	925.9219	
Albania	DALYs	Male	2012	1142.078	1601.323	957.0178	
Albania	DALYs	Male	2013	1198.966	1636.819	1018.705	
Albania	DALYs	Male	2014	1266.06	1760.007	1074.004	
Albania	DALYs	Male	2015	1328.113	1866.309	1123.168	
Albania	DALYs	Male	2016	1390.887	1948.448	1180.839	
Albania	DALYs	Male	2017	1449.155	2006.65	1220.012	
Albania	DALYs	Male	2018	1504.216	2061.898	1256.019	
Albania	DALYs	Male	2019	1602.77	2204.615	1352.35	
Albania	DALYs	Male	2020	1660.411	2313.602	1435.754	
Albania	DALYs	Male	2021	1795.461	2608.42	1540.94	
Albania	DALYs	Male	2022	1726.23	2429.236	1450.063	
Albania	DALYs	Male	2023	1562.778	2175.014	1281.463	
Albania	DALYs	Female	2010	293.8549	357.4637	224.3989	
Albania	DALYs	Female	2011	303.2601	366.1871	223.5253	
Albania	DALYs	Female	2012	319.8088	389.6726	231.8295	
Albania	DALYs	Female	2013	341.5791	417.8809	244.314	
Albania	DALYs	Female	2014	366.4747	442.8917	257.3867	
Albania	DALYs	Female	2015	391.9348	480.0275	268.4771	
Albania	DALYs	Female	2016	416.0842	508.4941	283.2518	
Albania	DALYs	Female	2017	442.0781	544.5952	300.6677	
Albania	DALYs	Female	2018	448.7695	559.4693	307.2654	
Albania	DALYs	Female	2019	465.7836	570.8174	323.0951	
Albania	DALYs	Female	2020	472.8615	575.0388	349.7741	
Albania	DALYs	Female	2021	501.5088	608.474	386.3707	
Albania	DALYs	Female	2022	526.7378	662.5986	363.2359	
Albania	DALYs	Female	2023	484.299	626.9078	340.0444	
Algeria	Incidence	Male	2010	7.48099	10.40889	5.032523	

Algeria	Incidence	Male	2011	7.689325	10.95737	5.208824	
Algeria	Incidence	Male	2012	7.951623	11.41782	5.330371	
Algeria	Incidence	Male	2013	8.132928	11.96824	5.366908	
Algeria	Incidence	Male	2014	8.367682	12.42047	5.5663	
Algeria	Incidence	Male	2015	8.603881	12.69951	5.697967	
Algeria	Incidence	Male	2016	8.951532	13.52879	5.814497	
Algeria	Incidence	Male	2017	9.393565	14.01116	5.905685	
Algeria	Incidence	Male	2018	9.826697	14.77826	6.041837	
Algeria	Incidence	Male	2019	10.27588	15.68117	6.017912	
Algeria	Incidence	Male	2020	10.217	15.83109	5.923472	
Algeria	Incidence	Male	2021	10.47548	16.52034	6.142065	
Algeria	Incidence	Male	2022	11.2332	17.9627	6.472528	
Algeria	Incidence	Male	2023	11.86661	18.8449	6.952996	
Algeria	Incidence	Female	2010	1.69629	2.568843	1.140109	
Algeria	Incidence	Female	2011	1.770142	2.671096	1.20623	
Algeria	Incidence	Female	2012	1.838576	2.769975	1.266838	
Algeria	Incidence	Female	2013	1.900763	2.762475	1.272177	
Algeria	Incidence	Female	2014	1.962986	2.821354	1.297895	
Algeria	Incidence	Female	2015	2.047771	2.980608	1.326569	
Algeria	Incidence	Female	2016	2.121389	3.064425	1.386129	
Algeria	Incidence	Female	2017	2.247672	3.308949	1.50905	
Algeria	Incidence	Female	2018	2.382301	3.46955	1.632085	
Algeria	Incidence	Female	2019	2.516341	3.48514	1.734394	
Algeria	Incidence	Female	2020	2.457631	3.303294	1.717546	
Algeria	Incidence	Female	2021	2.493091	3.396848	1.721979	
Algeria	Incidence	Female	2022	2.641653	3.623446	1.75774	
Algeria	Incidence	Female	2023	2.776747	3.931626	1.841577	
Algeria	Deaths	Male	2010	7.340096	10.2741	4.886288	
Algeria	Deaths	Male	2011	7.539313	10.83119	5.131304	
Algeria	Deaths	Male	2012	7.789692	11.23781	5.267784	
Algeria	Deaths	Male	2013	7.962475	11.70625	5.352511	
Algeria	Deaths	Male	2014	8.187411	12.1846	5.550977	
Algeria	Deaths	Male	2015	8.409449	12.38216	5.582458	
Algeria	Deaths	Male	2016	8.733125	13.13646	5.740888	
Algeria	Deaths	Male	2017	9.145521	13.69918	5.76103	
Algeria	Deaths	Male	2018	9.543768	14.13821	5.889	

Algeria	Deaths	Male	2019	9.949766	15.18981	5.850957	
Algeria	Deaths	Male	2020	9.881776	15.20369	5.847251	
Algeria	Deaths	Male	2021	10.11178	15.75498	6.038398	
Algeria	Deaths	Male	2022	10.80587	17.26723	6.311242	
Algeria	Deaths	Male	2023	11.39115	17.92493	6.726609	
Algeria	Deaths	Female	2010	1.607605	2.437693	1.084951	
Algeria	Deaths	Female	2011	1.675279	2.53666	1.131612	
Algeria	Deaths	Female	2012	1.737224	2.609	1.181765	
Algeria	Deaths	Female	2013	1.79336	2.614232	1.212273	
Algeria	Deaths	Female	2014	1.848813	2.666535	1.234253	
Algeria	Deaths	Female	2015	1.924479	2.782556	1.252544	
Algeria	Deaths	Female	2016	1.988176	2.882204	1.299379	
Algeria	Deaths	Female	2017	2.100321	3.097556	1.395636	
Algeria	Deaths	Female	2018	2.218193	3.226407	1.509162	
Algeria	Deaths	Female	2019	2.334189	3.211241	1.593345	
Algeria	Deaths	Female	2020	2.274539	3.078449	1.588673	
Algeria	Deaths	Female	2021	2.301529	3.144693	1.577464	
Algeria	Deaths	Female	2022	2.429001	3.356068	1.601244	
Algeria	Deaths	Female	2023	2.547942	3.572475	1.683184	
Algeria	DALYs	Male	2010	195.357	269.9772	129.6759	
Algeria	DALYs	Male	2011	200.3704	284.5605	131.4905	
Algeria	DALYs	Male	2012	206.6717	298.3769	134.3021	
Algeria	DALYs	Male	2013	210.7256	310.4137	137.1464	
Algeria	DALYs	Male	2014	216.2969	323.1776	141.7901	
Algeria	DALYs	Male	2015	221.7345	330.118	143.963	
Algeria	DALYs	Male	2016	229.8952	348.8935	144.051	
Algeria	DALYs	Male	2017	240.2187	360.9199	147.9411	
Algeria	DALYs	Male	2018	250.2375	373.5785	151.9484	
Algeria	DALYs	Male	2019	260.6644	401.6917	153.7004	
Algeria	DALYs	Male	2020	257.5488	398.6189	148.1711	
Algeria	DALYs	Male	2021	262.6628	410.8369	149.0573	
Algeria	DALYs	Male	2022	281.102	448.9315	157.6019	
Algeria	DALYs	Male	2023	296.1588	464.0086	170.4307	
Algeria	DALYs	Female	2010	46.89659	70.3668	31.79441	
Algeria	DALYs	Female	2011	48.79982	73.31763	33.52414	
Algeria	DALYs	Female	2012	50.52854	75.23352	34.88782	

Algeria	DALYs	Female	2013	52.06917	76.03547	35.39613	
Algeria	DALYs	Female	2014	53.65411	76.23525	35.3473	
Algeria	DALYs	Female	2015	55.79313	79.97737	36.4845	
Algeria	DALYs	Female	2016	57.55033	82.54546	37.56483	
Algeria	DALYs	Female	2017	60.67795	88.83924	40.67693	
Algeria	DALYs	Female	2018	64.02663	92.73175	43.79156	
Algeria	DALYs	Female	2019	67.27997	92.3374	46.25096	
Algeria	DALYs	Female	2020	65.24563	87.66808	45.62734	
Algeria	DALYs	Female	2021	65.74215	88.47861	45.28971	
Algeria	DALYs	Female	2022	69.46071	95.43592	46.37427	
Algeria	DALYs	Female	2023	72.72535	101.5934	48.01949	
Angola	Incidence	Male	2010	3.659105	5.888987	1.97298	
Angola	Incidence	Male	2011	3.706362	6.056549	2.027992	
Angola	Incidence	Male	2012	3.722146	5.997334	2.009444	
Angola	Incidence	Male	2013	3.780487	5.908788	2.054387	
Angola	Incidence	Male	2014	3.863542	5.967738	2.098221	
Angola	Incidence	Male	2015	3.938376	6.103126	2.179685	
Angola	Incidence	Male	2016	3.954395	6.118951	2.230506	
Angola	Incidence	Male	2017	3.987277	6.154364	2.208386	
Angola	Incidence	Male	2018	4.011592	6.249282	2.21643	
Angola	Incidence	Male	2019	4.060773	6.278736	2.180207	
Angola	Incidence	Male	2020	4.193806	6.601128	2.202997	
Angola	Incidence	Male	2021	4.452952	7.030568	2.308646	
Angola	Incidence	Male	2022	4.632281	7.013814	2.407557	
Angola	Incidence	Male	2023	4.814247	7.232632	2.391565	
Angola	Incidence	Female	2010	1.225392	1.907947	0.70523	
Angola	Incidence	Female	2011	1.262006	1.960209	0.742172	
Angola	Incidence	Female	2012	1.292027	1.951551	0.753516	
Angola	Incidence	Female	2013	1.356462	1.993924	0.792682	
Angola	Incidence	Female	2014	1.434341	2.111876	0.838308	
Angola	Incidence	Female	2015	1.454295	2.087163	0.858244	
Angola	Incidence	Female	2016	1.42401	2.001941	0.828785	
Angola	Incidence	Female	2017	1.414662	1.977047	0.814065	
Angola	Incidence	Female	2018	1.438082	1.972525	0.809423	
Angola	Incidence	Female	2019	1.487725	2.00941	0.818218	
Angola	Incidence	Female	2020	1.509949	2.035501	0.819892	

Angola	Incidence	Female	2021	1.608496	2.220823	0.881841	
Angola	Incidence	Female	2022	1.809799	2.462768	0.987653	
Angola	Incidence	Female	2023	1.939302	2.705611	1.106489	
Angola	Deaths	Male	2010	3.576473	5.74305	1.916716	
Angola	Deaths	Male	2011	3.618889	5.923316	1.97709	
Angola	Deaths	Male	2012	3.630874	5.796878	1.962435	
Angola	Deaths	Male	2013	3.682835	5.734885	1.997838	
Angola	Deaths	Male	2014	3.75843	5.791231	2.0402	
Angola	Deaths	Male	2015	3.828931	5.927507	2.109049	
Angola	Deaths	Male	2016	3.844274	6.000071	2.162257	
Angola	Deaths	Male	2017	3.875663	5.989653	2.146659	
Angola	Deaths	Male	2018	3.90001	6.103802	2.151779	
Angola	Deaths	Male	2019	3.950607	6.11575	2.108295	
Angola	Deaths	Male	2020	4.093489	6.452447	2.150719	
Angola	Deaths	Male	2021	4.34477	6.845745	2.229112	
Angola	Deaths	Male	2022	4.506642	6.855743	2.315172	
Angola	Deaths	Male	2023	4.677929	7.071028	2.341759	
Angola	Deaths	Female	2010	1.210181	1.888831	0.689512	
Angola	Deaths	Female	2011	1.245927	1.933841	0.724198	
Angola	Deaths	Female	2012	1.274744	1.923782	0.735771	
Angola	Deaths	Female	2013	1.333383	1.978617	0.767231	
Angola	Deaths	Female	2014	1.403873	2.066646	0.804857	
Angola	Deaths	Female	2015	1.424002	2.042873	0.823649	
Angola	Deaths	Female	2016	1.399747	1.967033	0.805261	
Angola	Deaths	Female	2017	1.394806	1.970479	0.790695	
Angola	Deaths	Female	2018	1.418252	1.937342	0.785798	
Angola	Deaths	Female	2019	1.465628	1.980297	0.794576	
Angola	Deaths	Female	2020	1.486883	2.012208	0.798196	
Angola	Deaths	Female	2021	1.580755	2.186368	0.8523	
Angola	Deaths	Female	2022	1.771899	2.404413	0.949256	
Angola	Deaths	Female	2023	1.894827	2.657561	1.072066	
Angola	DALYs	Male	2010	106.593	172.9654	58.04378	
Angola	DALYs	Male	2011	108.1986	175.504	59.50381	
Angola	DALYs	Male	2012	108.8905	175.3863	58.57781	
Angola	DALYs	Male	2013	110.9141	172.8914	60.44492	
Angola	DALYs	Male	2014	113.5898	176.7972	61.48085	

Angola	DALYs	Male	2015	115.8695	180.606	63.98413	
Angola	DALYs	Male	2016	116.2707	178.5109	65.52632	
Angola	DALYs	Male	2017	117.2529	180.5357	64.84879	
Angola	DALYs	Male	2018	117.8447	184.1397	64.66048	
Angola	DALYs	Male	2019	118.9234	185.1626	63.41455	
Angola	DALYs	Male	2020	121.721	190.9111	63.65961	
Angola	DALYs	Male	2021	129.019	204.5706	67.37545	
Angola	DALYs	Male	2022	134.7328	202.9343	71.06965	
Angola	DALYs	Male	2023	140.1349	211.9248	68.77411	
Angola	DALYs	Female	2010	35.84546	56.38882	21.086	
Angola	DALYs	Female	2011	36.88791	57.31743	22.17581	
Angola	DALYs	Female	2012	37.76753	57.2739	22.80251	
Angola	DALYs	Female	2013	39.92318	58.81652	24.08482	
Angola	DALYs	Female	2014	42.52649	62.11743	25.8938	
Angola	DALYs	Female	2015	43.02333	62.30784	25.86877	
Angola	DALYs	Female	2016	41.70157	59.29	24.49015	
Angola	DALYs	Female	2017	41.10665	56.76482	24.06306	
Angola	DALYs	Female	2018	41.71013	57.37173	24.48386	
Angola	DALYs	Female	2019	43.17749	58.86034	24.7235	
Angola	DALYs	Female	2020	43.79216	59.97231	24.60035	
Angola	DALYs	Female	2021	46.71041	63.71752	26.52324	
Angola	DALYs	Female	2022	52.83692	71.24226	29.83653	
Angola	DALYs	Female	2023	56.76281	79.14652	33.83266	
Argentina	Incidence	Male	2010	44.15606	47.14875	41.24452	
Argentina	Incidence	Male	2011	43.63458	46.67905	40.84207	
Argentina	Incidence	Male	2012	42.58997	45.41002	40.14031	
Argentina	Incidence	Male	2013	42.01667	44.71652	39.04385	
Argentina	Incidence	Male	2014	40.26018	42.77884	37.68181	
Argentina	Incidence	Male	2015	39.90498	42.50632	37.32466	
Argentina	Incidence	Male	2016	39.78384	42.66033	37.39002	
Argentina	Incidence	Male	2017	37.98439	40.62174	35.5408	
Argentina	Incidence	Male	2018	37.33505	39.79016	34.8551	
Argentina	Incidence	Male	2019	36.17079	38.94866	33.60018	
Argentina	Incidence	Male	2020	34.09205	36.81964	31.63378	
Argentina	Incidence	Male	2021	34.70749	37.99747	32.17417	
Argentina	Incidence	Male	2022	38.04004	41.68005	35.02648	

Argentina	Incidence	Male	2023	33.90072	38.65239	30.56531	
Argentina	Incidence	Female	2010	17.18882	18.67392	16.01493	
Argentina	Incidence	Female	2011	17.60706	19.08229	16.34151	
Argentina	Incidence	Female	2012	17.63751	19.027	16.24518	
Argentina	Incidence	Female	2013	18.1772	19.58066	16.78017	
Argentina	Incidence	Female	2014	18.14753	19.45633	16.72755	
Argentina	Incidence	Female	2015	18.05087	19.30502	16.70621	
Argentina	Incidence	Female	2016	18.47988	19.84743	17.14075	
Argentina	Incidence	Female	2017	18.10539	19.62517	16.80054	
Argentina	Incidence	Female	2018	17.83855	19.58017	16.41726	
Argentina	Incidence	Female	2019	18.03488	19.72842	16.4796	
Argentina	Incidence	Female	2020	17.15936	18.72273	15.71643	
Argentina	Incidence	Female	2021	18.54695	20.21143	16.93861	
Argentina	Incidence	Female	2022	19.74844	21.44208	17.82459	
Argentina	Incidence	Female	2023	19.01567	20.94522	16.84932	
Argentina	Deaths	Male	2010	44.07711	46.84078	41.37482	
Argentina	Deaths	Male	2011	43.5356	46.33164	40.69473	
Argentina	Deaths	Male	2012	42.49568	45.09237	40.05934	
Argentina	Deaths	Male	2013	42.00598	44.63619	39.30776	
Argentina	Deaths	Male	2014	40.34882	42.80602	37.77318	
Argentina	Deaths	Male	2015	40.03085	42.59789	37.57961	
Argentina	Deaths	Male	2016	39.98497	42.61801	37.63724	
Argentina	Deaths	Male	2017	38.20841	40.86675	35.74917	
Argentina	Deaths	Male	2018	37.52237	39.94301	35.16628	
Argentina	Deaths	Male	2019	36.37341	39.18068	33.97988	
Argentina	Deaths	Male	2020	34.28225	36.99736	32.13202	
Argentina	Deaths	Male	2021	34.79125	38.1055	32.46393	
Argentina	Deaths	Male	2022	38.00463	41.75533	35.24	
Argentina	Deaths	Male	2023	34.13516	38.73347	30.84329	
Argentina	Deaths	Female	2010	17.32472	18.84831	16.18273	
Argentina	Deaths	Female	2011	17.70085	19.25673	16.4893	
Argentina	Deaths	Female	2012	17.69582	19.05284	16.32403	
Argentina	Deaths	Female	2013	18.24158	19.52796	16.7665	
Argentina	Deaths	Female	2014	18.22029	19.46511	16.80134	
Argentina	Deaths	Female	2015	18.15028	19.42849	16.70808	
Argentina	Deaths	Female	2016	18.60012	19.99157	17.19074	

Argentina	Deaths	Female	2017	18.20482	19.77714	16.82649	
Argentina	Deaths	Female	2018	17.90045	19.51245	16.43292	
Argentina	Deaths	Female	2019	18.08988	19.78995	16.60049	
Argentina	Deaths	Female	2020	17.17508	18.93084	15.75478	
Argentina	Deaths	Female	2021	18.51648	20.36378	17.12069	
Argentina	Deaths	Female	2022	19.78615	21.76257	17.96362	
Argentina	Deaths	Female	2023	19.10141	21.18647	16.98041	
Argentina	DALYs	Male	2010	1083.239	1148.746	1013.947	
Argentina	DALYs	Male	2011	1066.433	1130.418	1001.309	
Argentina	DALYs	Male	2012	1037.174	1100.05	979.0775	
Argentina	DALYs	Male	2013	1018.005	1080.101	953.9888	
Argentina	DALYs	Male	2014	969.8671	1029.419	906.3095	
Argentina	DALYs	Male	2015	955.5327	1017.689	897.1446	
Argentina	DALYs	Male	2016	947.5201	1008.674	893.1274	
Argentina	DALYs	Male	2017	901.9159	968.3821	847.0148	
Argentina	DALYs	Male	2018	883.3307	940.2615	827.3659	
Argentina	DALYs	Male	2019	849.6237	916.9503	792.9812	
Argentina	DALYs	Male	2020	795.676	861.5236	745.046	
Argentina	DALYs	Male	2021	807.3075	886.1025	754.8258	
Argentina	DALYs	Male	2022	895.7001	984.5913	827.4002	
Argentina	DALYs	Male	2023	779.1651	880.2209	705.1256	
Argentina	DALYs	Female	2010	409.2907	441.8392	385.9346	
Argentina	DALYs	Female	2011	418.5714	451.3616	392.5155	
Argentina	DALYs	Female	2012	418.5871	448.4866	389.6789	
Argentina	DALYs	Female	2013	430.4866	457.4038	402.2855	
Argentina	DALYs	Female	2014	428.8243	455.9267	400.2089	
Argentina	DALYs	Female	2015	424.2582	449.0029	398.9168	
Argentina	DALYs	Female	2016	430.9922	457.4169	403.9617	
Argentina	DALYs	Female	2017	421.7301	451.2478	396.2798	
Argentina	DALYs	Female	2018	414.4035	448.3586	386.547	
Argentina	DALYs	Female	2019	416.1268	451.8118	386.5543	
Argentina	DALYs	Female	2020	393.7224	428.0683	364.7005	
Argentina	DALYs	Female	2021	421.7112	458.6626	391.2427	
Argentina	DALYs	Female	2022	449.5307	485.3423	415.968	
Argentina	DALYs	Female	2023	426.6669	469.9787	382.9483	
Armenia	Incidence	Male	2010	72.57404	79.24468	67.43749	

Armenia	Incidence	Male	2011	74.52217	80.68574	67.99785	
Armenia	Incidence	Male	2012	69.89924	76.02591	64.04051	
Armenia	Incidence	Male	2013	67.6297	73.09171	62.26482	
Armenia	Incidence	Male	2014	69.34544	74.57999	64.27683	
Armenia	Incidence	Male	2015	68.22028	72.88038	63.16412	
Armenia	Incidence	Male	2016	66.97705	71.63761	61.69226	
Armenia	Incidence	Male	2017	65.4008	70.51328	61.07767	
Armenia	Incidence	Male	2018	65.67516	70.37416	61.41568	
Armenia	Incidence	Male	2019	69.34003	74.36876	64.83823	
Armenia	Incidence	Male	2020	67.8133	71.40398	62.34821	
Armenia	Incidence	Male	2021	68.20973	72.78728	63.00208	
Armenia	Incidence	Male	2022	67.74411	75.36695	60.83913	
Armenia	Incidence	Male	2023	60.37417	66.96367	54.15539	
Armenia	Incidence	Female	2010	12.85471	14.56866	11.50867	
Armenia	Incidence	Female	2011	13.36221	15.32161	11.98501	
Armenia	Incidence	Female	2012	12.95204	14.91095	11.54804	
Armenia	Incidence	Female	2013	12.29802	14.07465	10.96987	
Armenia	Incidence	Female	2014	12.21308	14.14872	10.98969	
Armenia	Incidence	Female	2015	12.10073	13.85874	10.86014	
Armenia	Incidence	Female	2016	11.91145	13.63634	10.65958	
Armenia	Incidence	Female	2017	12.91262	14.80934	11.44468	
Armenia	Incidence	Female	2018	12.37735	14.12129	10.78701	
Armenia	Incidence	Female	2019	13.93267	15.79327	12.08945	
Armenia	Incidence	Female	2020	14.6643	16.56719	12.72976	
Armenia	Incidence	Female	2021	14.09311	15.96736	12.181	
Armenia	Incidence	Female	2022	13.27757	15.41351	11.388	
Armenia	Incidence	Female	2023	11.54113	13.54219	9.6932	
Armenia	Deaths	Male	2010	71.64911	77.61418	66.5983	
Armenia	Deaths	Male	2011	73.74195	80.04602	67.39439	
Armenia	Deaths	Male	2012	69.37057	75.41422	63.86448	
Armenia	Deaths	Male	2013	67.18525	72.38334	62.00033	
Armenia	Deaths	Male	2014	68.95579	74.06582	63.91208	
Armenia	Deaths	Male	2015	68.02367	72.64925	63.1029	
Armenia	Deaths	Male	2016	66.80067	71.45107	61.63424	
Armenia	Deaths	Male	2017	65.03495	69.72157	60.72235	
Armenia	Deaths	Male	2018	65.13411	70.0267	60.97274	

Armenia	Deaths	Male	2019	68.77055	73.29864	64.33097	
Armenia	Deaths	Male	2020	67.31341	71.35338	62.28706	
Armenia	Deaths	Male	2021	67.60791	72.41384	62.69125	
Armenia	Deaths	Male	2022	66.95219	74.29889	60.56714	
Armenia	Deaths	Male	2023	60.12793	66.08353	54.06073	
Armenia	Deaths	Female	2010	13.08264	14.85236	11.73932	
Armenia	Deaths	Female	2011	13.64264	15.56845	12.17816	
Armenia	Deaths	Female	2012	13.28336	15.37133	11.87362	
Armenia	Deaths	Female	2013	12.62044	14.42384	11.2927	
Armenia	Deaths	Female	2014	12.61926	14.57685	11.3449	
Armenia	Deaths	Female	2015	12.55352	14.35781	11.23936	
Armenia	Deaths	Female	2016	12.3407	14.05577	11.02493	
Armenia	Deaths	Female	2017	13.33563	15.21075	11.82995	
Armenia	Deaths	Female	2018	12.73861	14.48763	11.17904	
Armenia	Deaths	Female	2019	14.20796	16.09987	12.34386	
Armenia	Deaths	Female	2020	15.00334	16.88309	13.01137	
Armenia	Deaths	Female	2021	14.34356	16.1501	12.40642	
Armenia	Deaths	Female	2022	13.48547	15.47904	11.58967	
Armenia	Deaths	Female	2023	11.86119	13.79531	9.974879	
Armenia	DALYs	Male	2010	1867.664	2029.637	1732.421	
Armenia	DALYs	Male	2011	1905.538	2064.381	1742.346	
Armenia	DALYs	Male	2012	1773.711	1931.161	1629.208	
Armenia	DALYs	Male	2013	1707.556	1847.7	1570.899	
Armenia	DALYs	Male	2014	1743.428	1870.27	1614.369	
Armenia	DALYs	Male	2015	1706.762	1818.495	1586.099	
Armenia	DALYs	Male	2016	1667.749	1788.948	1536.723	
Armenia	DALYs	Male	2017	1629.247	1749.684	1520.835	
Armenia	DALYs	Male	2018	1628.048	1738.582	1525.404	
Armenia	DALYs	Male	2019	1702.06	1820.037	1595.471	
Armenia	DALYs	Male	2020	1654.063	1748.362	1535.828	
Armenia	DALYs	Male	2021	1659.536	1767.833	1544.92	
Armenia	DALYs	Male	2022	1653.695	1847.319	1495.01	
Armenia	DALYs	Male	2023	1456.779	1622.794	1308.984	
Armenia	DALYs	Female	2010	312.4731	357.1	277.4817	
Armenia	DALYs	Female	2011	321.6386	370.2599	285.371	
Armenia	DALYs	Female	2012	309.6067	360.2676	274.3362	

Armenia	DALYs	Female	2013	292.4931	337.5017	258.8787	
Armenia	DALYs	Female	2014	285.7432	332.2164	254.2311	
Armenia	DALYs	Female	2015	281.137	323.321	251.7872	
Armenia	DALYs	Female	2016	277.7571	318.5953	247.2914	
Armenia	DALYs	Female	2017	300.439	346.7638	265.8261	
Armenia	DALYs	Female	2018	288.5456	330.2158	253.3623	
Armenia	DALYs	Female	2019	327.6457	370.8439	287.3908	
Armenia	DALYs	Female	2020	341.5452	387.5871	297.9266	
Armenia	DALYs	Female	2021	328.3227	372.9372	287.8179	
Armenia	DALYs	Female	2022	311.4091	358.7158	265.9478	
Armenia	DALYs	Female	2023	266.673	310.6698	225.3637	
Australia	Incidence	Male	2010	71.79922	75.9774	66.81371	
Australia	Incidence	Male	2011	72.98531	77.72151	68.38757	
Australia	Incidence	Male	2012	71.66896	76.53839	67.05467	
Australia	Incidence	Male	2013	71.29466	76.51992	66.58851	
Australia	Incidence	Male	2014	72.04958	77.65024	66.98496	
Australia	Incidence	Male	2015	66.73874	71.81803	61.36956	
Australia	Incidence	Male	2016	67.07385	73.11403	61.49654	
Australia	Incidence	Male	2017	65.4742	70.96804	60.12723	
Australia	Incidence	Male	2018	65.20886	70.88571	58.91038	
Australia	Incidence	Male	2019	66.40403	72.14621	59.57426	
Australia	Incidence	Male	2020	61.17894	66.72836	55.0051	
Australia	Incidence	Male	2021	62.93445	68.90896	56.56439	
Australia	Incidence	Male	2022	64.87859	71.5132	58.34552	
Australia	Incidence	Male	2023	61.1826	68.65571	53.1495	
Australia	Incidence	Female	2010	46.17513	50.55783	41.32464	
Australia	Incidence	Female	2011	46.83442	51.32126	42.11575	
Australia	Incidence	Female	2012	47.02922	51.04856	41.76951	
Australia	Incidence	Female	2013	46.50009	50.29018	41.45897	
Australia	Incidence	Female	2014	47.77457	51.81214	42.61747	
Australia	Incidence	Female	2015	46.79004	50.83905	41.17562	
Australia	Incidence	Female	2016	45.97028	49.43477	40.17779	
Australia	Incidence	Female	2017	45.50472	49.38896	39.15092	
Australia	Incidence	Female	2018	46.47641	50.41101	40.37234	
Australia	Incidence	Female	2019	46.84744	50.55354	40.40436	
Australia	Incidence	Female	2020	45.41294	48.95815	39.28672	

Australia	Incidence	Female	2021	46.7733	50.4998	40.19362	
Australia	Incidence	Female	2022	47.87601	51.51009	41.50368	
Australia	Incidence	Female	2023	47.54501	51.77084	40.59691	
Australia	Deaths	Male	2010	49.50074	52.53966	46.58229	
Australia	Deaths	Male	2011	50.30798	53.06843	47.33434	
Australia	Deaths	Male	2012	49.4786	52.16263	46.37772	
Australia	Deaths	Male	2013	49.26135	51.86847	46.12373	
Australia	Deaths	Male	2014	49.99774	52.94985	46.73284	
Australia	Deaths	Male	2015	46.55665	49.34777	43.50249	
Australia	Deaths	Male	2016	47.03406	50.00207	44.04432	
Australia	Deaths	Male	2017	45.96364	48.85879	43.08341	
Australia	Deaths	Male	2018	45.56222	48.30851	42.76902	
Australia	Deaths	Male	2019	46.44053	49.42772	43.35383	
Australia	Deaths	Male	2020	42.6079	45.74713	39.79254	
Australia	Deaths	Male	2021	43.83516	47.05473	40.47242	
Australia	Deaths	Male	2022	45.05656	48.63734	41.34649	
Australia	Deaths	Male	2023	42.47892	47.33109	38.00818	
Australia	Deaths	Female	2010	31.10195	33.59045	27.66298	
Australia	Deaths	Female	2011	31.48436	33.90918	27.89664	
Australia	Deaths	Female	2012	31.61189	33.95328	27.88876	
Australia	Deaths	Female	2013	31.20038	33.52124	27.44655	
Australia	Deaths	Female	2014	32.06285	34.43764	28.22762	
Australia	Deaths	Female	2015	31.40127	33.82806	27.37396	
Australia	Deaths	Female	2016	30.88988	32.96661	27.13866	
Australia	Deaths	Female	2017	30.62622	32.92182	26.97054	
Australia	Deaths	Female	2018	31.20808	33.59966	27.35557	
Australia	Deaths	Female	2019	31.54957	33.88689	27.34859	
Australia	Deaths	Female	2020	30.56279	32.79988	26.51514	
Australia	Deaths	Female	2021	31.48119	33.91427	27.46043	
Australia	Deaths	Female	2022	32.21401	34.62882	27.82457	
Australia	Deaths	Female	2023	32.0651	34.86297	27.48885	
Australia	DALYs	Male	2010	1039.935	1102.313	982.6612	
Australia	DALYs	Male	2011	1054.258	1109.377	996.9716	
Australia	DALYs	Male	2012	1030.823	1085.247	974.0816	
Australia	DALYs	Male	2013	1025.762	1079.34	969.1526	
Australia	DALYs	Male	2014	1037.819	1095.508	979.8492	

Australia	DALYs	Male	2015	960.9336	1014.392	905.7914	
Australia	DALYs	Male	2016	961.9305	1019.191	909.6468	
Australia	DALYs	Male	2017	937.6324	992.0613	891.2474	
Australia	DALYs	Male	2018	933.5317	988.2297	885.426	
Australia	DALYs	Male	2019	940.1545	999.0893	883.5948	
Australia	DALYs	Male	2020	860.6658	926.879	811.3277	
Australia	DALYs	Male	2021	873.6131	941.1222	813.9823	
Australia	DALYs	Male	2022	895.2837	965.7247	831.3436	
Australia	DALYs	Male	2023	839.0475	937.9166	753.0442	
Australia	DALYs	Female	2010	656.2912	702.3946	598.3803	
Australia	DALYs	Female	2011	664.145	709.3696	604.721	
Australia	DALYs	Female	2012	661.515	703.7866	599.8328	
Australia	DALYs	Female	2013	653.9748	694.8945	593.42	
Australia	DALYs	Female	2014	671.0687	711.1589	610.4822	
Australia	DALYs	Female	2015	657.6613	701.4268	594.456	
Australia	DALYs	Female	2016	642.1628	678.1452	576.0221	
Australia	DALYs	Female	2017	630.8698	668.1471	565.7999	
Australia	DALYs	Female	2018	641.0832	679.9535	572.4169	
Australia	DALYs	Female	2019	638.5038	677.1532	573.5988	
Australia	DALYs	Female	2020	612.9961	650.5123	547.1606	
Australia	DALYs	Female	2021	626.259	667.2202	563.7145	
Australia	DALYs	Female	2022	638.2807	680.1277	569.9062	
Australia	DALYs	Female	2023	629.666	677.5512	560.4661	
Austria	Incidence	Male	2010	74.23545	80.36321	68.93125	
Austria	Incidence	Male	2011	74.47651	80.77481	68.69745	
Austria	Incidence	Male	2012	74.24413	80.35661	68.31141	
Austria	Incidence	Male	2013	75.56845	81.32802	69.16853	
Austria	Incidence	Male	2014	75.02313	81.30918	68.92	
Austria	Incidence	Male	2015	73.04987	79.13158	66.88147	
Austria	Incidence	Male	2016	71.26719	77.55026	65.16642	
Austria	Incidence	Male	2017	70.57735	76.59999	64.66593	
Austria	Incidence	Male	2018	71.56042	77.94298	65.641	
Austria	Incidence	Male	2019	68.91249	74.76963	62.80039	
Austria	Incidence	Male	2020	69.34556	75.75616	62.94728	
Austria	Incidence	Male	2021	68.31255	75.09119	62.00468	
Austria	Incidence	Male	2022	70.36118	77.65199	63.05022	

Austria	Incidence	Male	2023	69.03043	76.96877	61.8149	
Austria	Incidence	Female	2010	38.21703	42.4449	34.58407	
Austria	Incidence	Female	2011	39.38179	43.55772	35.42101	
Austria	Incidence	Female	2012	41.20321	45.59146	37.19308	
Austria	Incidence	Female	2013	41.54145	45.7767	37.31752	
Austria	Incidence	Female	2014	43.85289	48.77547	39.20455	
Austria	Incidence	Female	2015	44.68608	49.2315	39.98694	
Austria	Incidence	Female	2016	44.65108	49.23604	40.20099	
Austria	Incidence	Female	2017	44.57522	49.15589	39.68286	
Austria	Incidence	Female	2018	45.98367	51.29173	40.83599	
Austria	Incidence	Female	2019	47.06161	52.31504	41.83772	
Austria	Incidence	Female	2020	47.7059	53.13329	42.47255	
Austria	Incidence	Female	2021	48.80847	53.91281	43.26852	
Austria	Incidence	Female	2022	50.71883	56.16131	44.6231	
Austria	Incidence	Female	2023	49.85979	55.37835	43.31152	
Austria	Deaths	Male	2010	65.39897	69.738	62.07376	
Austria	Deaths	Male	2011	65.63858	70.04895	61.6466	
Austria	Deaths	Male	2012	65.45334	69.65815	61.92229	
Austria	Deaths	Male	2013	66.64191	70.50585	63.25748	
Austria	Deaths	Male	2014	66.16429	70.25472	62.18053	
Austria	Deaths	Male	2015	64.54082	68.23333	60.65922	
Austria	Deaths	Male	2016	63.00056	66.95013	59.28917	
Austria	Deaths	Male	2017	62.39754	66.2142	58.6131	
Austria	Deaths	Male	2018	63.08496	67.37608	59.76545	
Austria	Deaths	Male	2019	60.80303	65.28381	57.53958	
Austria	Deaths	Male	2020	61.14586	65.81107	57.57174	
Austria	Deaths	Male	2021	60.05366	65.4862	56.12511	
Austria	Deaths	Male	2022	61.98096	67.30665	57.37816	
Austria	Deaths	Male	2023	60.984	65.99586	56.36067	
Austria	Deaths	Female	2010	32.937	35.18765	29.80534	
Austria	Deaths	Female	2011	33.77057	35.98275	30.58575	
Austria	Deaths	Female	2012	35.23528	37.83669	31.89359	
Austria	Deaths	Female	2013	35.41874	37.82785	32.34957	
Austria	Deaths	Female	2014	37.21962	40.03396	33.80811	
Austria	Deaths	Female	2015	37.97547	40.83814	34.63095	
Austria	Deaths	Female	2016	37.81321	40.46173	34.44068	

Austria	Deaths	Female	2017	37.77698	40.57059	34.37877	
Austria	Deaths	Female	2018	38.96627	42.03257	35.35861	
Austria	Deaths	Female	2019	39.78074	42.73125	36.00176	
Austria	Deaths	Female	2020	40.44102	43.31377	36.89132	
Austria	Deaths	Female	2021	41.13421	44.09705	37.48937	
Austria	Deaths	Female	2022	42.82292	46.10979	38.55763	
Austria	Deaths	Female	2023	42.14014	46.12987	37.34172	
Austria	DALYs	Male	2010	1543.011	1647.158	1464.821	
Austria	DALYs	Male	2011	1536.601	1643.4	1460.59	
Austria	DALYs	Male	2012	1520.037	1616.877	1440.771	
Austria	DALYs	Male	2013	1534.199	1625.03	1458.958	
Austria	DALYs	Male	2014	1510.273	1603.753	1429.019	
Austria	DALYs	Male	2015	1457.842	1545.004	1378.718	
Austria	DALYs	Male	2016	1414.818	1502.855	1339.545	
Austria	DALYs	Male	2017	1389.045	1475.998	1311.691	
Austria	DALYs	Male	2018	1400.269	1493.573	1324.782	
Austria	DALYs	Male	2019	1332.835	1427.992	1257.155	
Austria	DALYs	Male	2020	1333.801	1440.131	1252.959	
Austria	DALYs	Male	2021	1307.835	1426.91	1224.988	
Austria	DALYs	Male	2022	1337.928	1457.586	1249.134	
Austria	DALYs	Male	2023	1304.65	1418.07	1211.424	
Austria	DALYs	Female	2010	747.3686	789.0173	693.2639	
Austria	DALYs	Female	2011	769.8809	813.3267	712.4031	
Austria	DALYs	Female	2012	800.5818	850.0473	741.2708	
Austria	DALYs	Female	2013	802.1825	850.3833	747.0237	
Austria	DALYs	Female	2014	843.5105	895.0838	784.2018	
Austria	DALYs	Female	2015	853.6344	909.6163	796.0584	
Austria	DALYs	Female	2016	848.4738	900.2851	790.9925	
Austria	DALYs	Female	2017	838.6243	888.4568	784.049	
Austria	DALYs	Female	2018	853.4325	910.3529	787.3658	
Austria	DALYs	Female	2019	864.1707	922.1159	800.0768	
Austria	DALYs	Female	2020	861.9928	920.6383	802.4099	
Austria	DALYs	Female	2021	877.0595	934.2041	815.1016	
Austria	DALYs	Female	2022	905.957	969.8878	831.2527	
Austria	DALYs	Female	2023	886.9035	965.5542	811.173	
Azerbaijan	Incidence	Male	2010	25.13158	43.16626	18.47408	

Azerbaijan	Incidence	Male	2011	26.27177	43.87428	19.39864	
Azerbaijan	Incidence	Male	2012	27.10356	45.33253	19.43247	
Azerbaijan	Incidence	Male	2013	27.14822	45.10309	19.26568	
Azerbaijan	Incidence	Male	2014	27.24665	45.35081	19.01726	
Azerbaijan	Incidence	Male	2015	23.30376	38.99295	16.37464	
Azerbaijan	Incidence	Male	2016	23.83489	39.84774	16.77889	
Azerbaijan	Incidence	Male	2017	24.32381	40.61685	17.16281	
Azerbaijan	Incidence	Male	2018	24.95117	42.02428	17.38563	
Azerbaijan	Incidence	Male	2019	26.76142	44.71156	18.25072	
Azerbaijan	Incidence	Male	2020	29.18313	47.82621	20.02547	
Azerbaijan	Incidence	Male	2021	27.08272	43.72727	19.04122	
Azerbaijan	Incidence	Male	2022	25.82052	42.17188	17.97509	
Azerbaijan	Incidence	Male	2023	28.1107	44.82598	19.60814	
Azerbaijan	Incidence	Female	2010	5.655121	7.04627	4.595181	
Azerbaijan	Incidence	Female	2011	5.750424	7.230382	4.7173	
Azerbaijan	Incidence	Female	2012	5.828803	7.332392	4.690952	
Azerbaijan	Incidence	Female	2013	5.934831	7.644895	4.672402	
Azerbaijan	Incidence	Female	2014	6.066577	7.720437	4.85784	
Azerbaijan	Incidence	Female	2015	5.90802	7.4227	4.556904	
Azerbaijan	Incidence	Female	2016	6.08274	7.807809	4.648083	
Azerbaijan	Incidence	Female	2017	6.232353	8.214593	4.799076	
Azerbaijan	Incidence	Female	2018	6.439697	8.35457	4.893263	
Azerbaijan	Incidence	Female	2019	7.044029	9.153297	5.364751	
Azerbaijan	Incidence	Female	2020	7.803933	10.04343	6.002984	
Azerbaijan	Incidence	Female	2021	7.888746	10.31179	6.218327	
Azerbaijan	Incidence	Female	2022	6.606892	8.644346	5.11831	
Azerbaijan	Incidence	Female	2023	6.917684	9.086684	5.300455	
Azerbaijan	Deaths	Male	2010	24.19115	41.76421	17.82675	
Azerbaijan	Deaths	Male	2011	25.25983	42.41596	18.64004	
Azerbaijan	Deaths	Male	2012	26.03955	43.73743	18.72173	
Azerbaijan	Deaths	Male	2013	26.06675	43.46122	18.49331	
Azerbaijan	Deaths	Male	2014	26.13456	43.55513	18.19026	
Azerbaijan	Deaths	Male	2015	22.32817	37.48468	15.69645	
Azerbaijan	Deaths	Male	2016	22.82292	38.41079	16.16818	
Azerbaijan	Deaths	Male	2017	23.27486	39.09105	16.37348	
Azerbaijan	Deaths	Male	2018	23.85529	40.58882	16.59065	

Azerbaijan	Deaths	Male	2019	25.55658	42.83878	17.46148	
Azerbaijan	Deaths	Male	2020	27.88068	45.90331	19.14368	
Azerbaijan	Deaths	Male	2021	25.92263	41.98345	18.24705	
Azerbaijan	Deaths	Male	2022	24.65901	40.42417	17.11396	
Azerbaijan	Deaths	Male	2023	26.80331	42.94627	18.76983	
Azerbaijan	Deaths	Female	2010	5.581338	6.955255	4.511351	
Azerbaijan	Deaths	Female	2011	5.67696	7.091599	4.657799	
Azerbaijan	Deaths	Female	2012	5.761281	7.262454	4.615865	
Azerbaijan	Deaths	Female	2013	5.876167	7.576551	4.615761	
Azerbaijan	Deaths	Female	2014	6.012289	7.705664	4.783105	
Azerbaijan	Deaths	Female	2015	5.852276	7.325797	4.51415	
Azerbaijan	Deaths	Female	2016	6.024994	7.756611	4.606212	
Azerbaijan	Deaths	Female	2017	6.164016	8.088205	4.716758	
Azerbaijan	Deaths	Female	2018	6.351571	8.254892	4.852398	
Azerbaijan	Deaths	Female	2019	6.919376	9.021584	5.283942	
Azerbaijan	Deaths	Female	2020	7.663467	9.876081	5.902973	
Azerbaijan	Deaths	Female	2021	7.770999	10.18168	6.141547	
Azerbaijan	Deaths	Female	2022	6.50415	8.464735	5.024264	
Azerbaijan	Deaths	Female	2023	6.791148	8.929071	5.18895	
Azerbaijan	DALYs	Male	2010	722.7436	1223.341	531.9424	
Azerbaijan	DALYs	Male	2011	757.097	1250.028	558.2068	
Azerbaijan	DALYs	Male	2012	780.7663	1294.56	558.2533	
Azerbaijan	DALYs	Male	2013	781.2796	1286.618	554.3412	
Azerbaijan	DALYs	Male	2014	783.9316	1290.878	548.0529	
Azerbaijan	DALYs	Male	2015	669.9072	1104.35	468.8208	
Azerbaijan	DALYs	Male	2016	684.5458	1128.162	479.2026	
Azerbaijan	DALYs	Male	2017	697.3988	1150.779	489.3531	
Azerbaijan	DALYs	Male	2018	713.8927	1179.01	495.9958	
Azerbaijan	DALYs	Male	2019	763.8933	1255.222	522.2619	
Azerbaijan	DALYs	Male	2020	827.5675	1335.614	569.07	
Azerbaijan	DALYs	Male	2021	764.0404	1214.913	534.6655	
Azerbaijan	DALYs	Male	2022	730.6436	1188.046	505.1659	
Azerbaijan	DALYs	Male	2023	794.2714	1249.286	551.3039	
Azerbaijan	DALYs	Female	2010	156.1258	194.0055	127.424	
Azerbaijan	DALYs	Female	2011	158.7276	197.9375	129.3093	
Azerbaijan	DALYs	Female	2012	160.2198	201.7683	129.6064	

Azerbaijan	DALYs	Female	2013	162.2559	208.2355	128.9017	
Azerbaijan	DALYs	Female	2014	165.2184	207.7272	133.5171	
Azerbaijan	DALYs	Female	2015	160.5241	199.8032	124.5429	
Azerbaijan	DALYs	Female	2016	164.9578	208.4752	126.5477	
Azerbaijan	DALYs	Female	2017	168.9323	219.7408	130.8265	
Azerbaijan	DALYs	Female	2018	174.7495	228.0255	134.6343	
Azerbaijan	DALYs	Female	2019	191.2905	248.6526	147.2091	
Azerbaijan	DALYs	Female	2020	209.881	269.7271	162.9894	
Azerbaijan	DALYs	Female	2021	210.1175	274.9055	166.3487	
Azerbaijan	DALYs	Female	2022	176.9364	231.8008	139.5301	
Azerbaijan	DALYs	Female	2023	184.9174	240.9829	141.6607	
Bahamas	Incidence	Male	2010	19.68401	22.86656	17.33781	
Bahamas	Incidence	Male	2011	19.64497	22.80928	17.21841	
Bahamas	Incidence	Male	2012	18.68156	21.56234	16.40454	
Bahamas	Incidence	Male	2013	18.50047	21.53075	16.19387	
Bahamas	Incidence	Male	2014	18.66307	21.83884	16.41423	
Bahamas	Incidence	Male	2015	19.17006	22.66673	16.89422	
Bahamas	Incidence	Male	2016	19.46114	22.61718	16.83983	
Bahamas	Incidence	Male	2017	19.88094	22.93828	17.1749	
Bahamas	Incidence	Male	2018	20.44093	23.75389	17.61825	
Bahamas	Incidence	Male	2019	21.87244	25.57331	18.65428	
Bahamas	Incidence	Male	2020	23.5966	27.86817	20.1969	
Bahamas	Incidence	Male	2021	30.05404	35.00259	25.74673	
Bahamas	Incidence	Male	2022	24.10304	28.55147	20.42948	
Bahamas	Incidence	Male	2023	22.66821	26.96042	18.87169	
Bahamas	Incidence	Female	2010	8.363802	9.957215	7.062214	
Bahamas	Incidence	Female	2011	8.516862	10.17586	7.168082	
Bahamas	Incidence	Female	2012	8.444549	10.05732	7.07306	
Bahamas	Incidence	Female	2013	8.59131	10.19528	7.23499	
Bahamas	Incidence	Female	2014	8.601696	10.13129	7.241148	
Bahamas	Incidence	Female	2015	8.753116	10.32404	7.192813	
Bahamas	Incidence	Female	2016	9.018256	10.64084	7.50464	
Bahamas	Incidence	Female	2017	9.22793	10.89593	7.725608	
Bahamas	Incidence	Female	2018	9.471124	11.21226	7.952873	
Bahamas	Incidence	Female	2019	10.35534	12.36176	8.712005	
Bahamas	Incidence	Female	2020	10.24801	12.15526	8.582821	

Bahamas	Incidence	Female	2021	12.90169	15.53488	10.71481	
Bahamas	Incidence	Female	2022	10.96622	13.30988	9.057208	
Bahamas	Incidence	Female	2023	10.90015	13.14487	8.935573	
Bahamas	Deaths	Male	2010	18.68111	21.47367	16.47525	
Bahamas	Deaths	Male	2011	18.65422	21.49677	16.40319	
Bahamas	Deaths	Male	2012	17.79157	20.38184	15.68244	
Bahamas	Deaths	Male	2013	17.6322	20.39646	15.63601	
Bahamas	Deaths	Male	2014	17.78044	20.75205	15.65723	
Bahamas	Deaths	Male	2015	18.30446	21.62553	16.10898	
Bahamas	Deaths	Male	2016	18.57405	21.65705	16.0865	
Bahamas	Deaths	Male	2017	18.97964	22.2146	16.47675	
Bahamas	Deaths	Male	2018	19.51441	22.99969	16.95967	
Bahamas	Deaths	Male	2019	20.87309	24.63462	17.97564	
Bahamas	Deaths	Male	2020	22.50714	26.27075	19.31995	
Bahamas	Deaths	Male	2021	28.46117	32.79864	24.38428	
Bahamas	Deaths	Male	2022	22.96594	27.06407	19.52721	
Bahamas	Deaths	Male	2023	21.59661	25.57151	18.05907	
Bahamas	Deaths	Female	2010	7.869856	9.220211	6.676693	
Bahamas	Deaths	Female	2011	8.00608	9.440158	6.798169	
Bahamas	Deaths	Female	2012	7.940491	9.396864	6.685994	
Bahamas	Deaths	Female	2013	8.084628	9.542756	6.860895	
Bahamas	Deaths	Female	2014	8.098941	9.453595	6.871152	
Bahamas	Deaths	Female	2015	8.241057	9.68986	6.817519	
Bahamas	Deaths	Female	2016	8.48244	9.908026	7.067012	
Bahamas	Deaths	Female	2017	8.679496	10.17394	7.305287	
Bahamas	Deaths	Female	2018	8.905275	10.48343	7.521936	
Bahamas	Deaths	Female	2019	9.722587	11.46744	8.213135	
Bahamas	Deaths	Female	2020	9.615498	11.40345	8.115678	
Bahamas	Deaths	Female	2021	11.97875	14.26172	10.08258	
Bahamas	Deaths	Female	2022	10.26165	12.33066	8.447703	
Bahamas	Deaths	Female	2023	10.21589	12.22481	8.359467	
Bahamas	DALYs	Male	2010	531.5503	615.4771	464.7883	
Bahamas	DALYs	Male	2011	529.4882	617.0312	464.0617	
Bahamas	DALYs	Male	2012	499.1782	579.3484	440.0029	
Bahamas	DALYs	Male	2013	492.399	574.3161	433.6974	
Bahamas	DALYs	Male	2014	497.2787	580.8115	435.1405	

Bahamas	DALYs	Male	2015	506.683	599.0898	443.2993	
Bahamas	DALYs	Male	2016	513.2669	599.754	441.8055	
Bahamas	DALYs	Male	2017	522.7326	612.8615	449.9054	
Bahamas	DALYs	Male	2018	535.9837	630.0374	461.6974	
Bahamas	DALYs	Male	2019	571.2443	673.7144	488.1155	
Bahamas	DALYs	Male	2020	614.7968	717.184	523.2115	
Bahamas	DALYs	Male	2021	789.9282	916.7204	673.4521	
Bahamas	DALYs	Male	2022	624.1452	739.9118	525.6954	
Bahamas	DALYs	Male	2023	585.4609	697.8897	488.6026	
Bahamas	DALYs	Female	2010	216.7113	254.53	182.1789	
Bahamas	DALYs	Female	2011	220.4283	261.3748	186.3395	
Bahamas	DALYs	Female	2012	218.3863	258.7352	183.4152	
Bahamas	DALYs	Female	2013	221.2475	262.2598	186.3579	
Bahamas	DALYs	Female	2014	220.9555	259.5518	186.1805	
Bahamas	DALYs	Female	2015	224.8217	263.7473	184.9778	
Bahamas	DALYs	Female	2016	230.965	271.7479	190.7365	
Bahamas	DALYs	Female	2017	235.4417	275.8453	195.4982	
Bahamas	DALYs	Female	2018	240.7654	285.1115	201.9678	
Bahamas	DALYs	Female	2019	262.1307	309.6744	220.1384	
Bahamas	DALYs	Female	2020	258.5882	309.175	218.4236	
Bahamas	DALYs	Female	2021	330.5847	398.7139	276.8497	
Bahamas	DALYs	Female	2022	274.4797	331.9299	225.3757	
Bahamas	DALYs	Female	2023	270.3652	324.5828	220.0029	
Bahrain	Incidence	Male	2010	9.922206	12.76418	7.903087	
Bahrain	Incidence	Male	2011	9.921199	12.9312	7.957746	
Bahrain	Incidence	Male	2012	10.07771	13.42543	8.042261	
Bahrain	Incidence	Male	2013	10.07232	13.4412	7.965341	
Bahrain	Incidence	Male	2014	10.17877	14.13329	7.846614	
Bahrain	Incidence	Male	2015	11.05092	15.64011	8.560552	
Bahrain	Incidence	Male	2016	11.38203	15.65598	8.782019	
Bahrain	Incidence	Male	2017	11.74938	15.77252	8.940172	
Bahrain	Incidence	Male	2018	12.53208	17.12459	9.43367	
Bahrain	Incidence	Male	2019	13.05989	18.11734	9.664896	
Bahrain	Incidence	Male	2020	13.44204	18.67763	9.96671	
Bahrain	Incidence	Male	2021	15.26818	21.26331	11.19798	
Bahrain	Incidence	Male	2022	17.54916	24.86037	12.2372	

Bahrain	Incidence	Male	2023	19.98465	28.19937	13.38787	
Bahrain	Incidence	Female	2010	5.501475	7.339247	4.094344	
Bahrain	Incidence	Female	2011	5.554381	7.526386	4.147394	
Bahrain	Incidence	Female	2012	5.713262	7.774177	4.139187	
Bahrain	Incidence	Female	2013	5.732171	7.886962	4.128697	
Bahrain	Incidence	Female	2014	5.677952	7.770619	3.948581	
Bahrain	Incidence	Female	2015	5.971506	8.112823	4.111104	
Bahrain	Incidence	Female	2016	6.166161	8.410905	4.240942	
Bahrain	Incidence	Female	2017	6.296835	8.565473	4.308522	
Bahrain	Incidence	Female	2018	6.342827	8.66734	4.430574	
Bahrain	Incidence	Female	2019	6.296768	8.777401	4.504833	
Bahrain	Incidence	Female	2020	6.131312	8.25569	4.358057	
Bahrain	Incidence	Female	2021	6.493274	8.637358	4.757251	
Bahrain	Incidence	Female	2022	6.579985	8.901835	4.6412	
Bahrain	Incidence	Female	2023	7.400835	10.49408	5.157608	
Bahrain	Deaths	Male	2010	9.299296	11.85507	7.429611	
Bahrain	Deaths	Male	2011	9.260348	12.01857	7.452452	
Bahrain	Deaths	Male	2012	9.374266	12.40059	7.498466	
Bahrain	Deaths	Male	2013	9.356014	12.46822	7.369126	
Bahrain	Deaths	Male	2014	9.450121	13.11615	7.357587	
Bahrain	Deaths	Male	2015	10.23999	14.46227	7.995369	
Bahrain	Deaths	Male	2016	10.505	14.32403	8.132108	
Bahrain	Deaths	Male	2017	10.80448	14.36116	8.242063	
Bahrain	Deaths	Male	2018	11.48603	15.42535	8.789774	
Bahrain	Deaths	Male	2019	11.91195	16.51171	8.966837	
Bahrain	Deaths	Male	2020	12.27226	16.95685	9.151509	
Bahrain	Deaths	Male	2021	13.86919	19.48662	10.24294	
Bahrain	Deaths	Male	2022	15.83354	22.3989	11.12156	
Bahrain	Deaths	Male	2023	18.00757	25.18211	11.95198	
Bahrain	Deaths	Female	2010	5.101658	6.81174	3.809141	
Bahrain	Deaths	Female	2011	5.127137	6.938388	3.776445	
Bahrain	Deaths	Female	2012	5.256441	7.083713	3.775033	
Bahrain	Deaths	Female	2013	5.258993	7.135707	3.807523	
Bahrain	Deaths	Female	2014	5.212677	7.016333	3.650006	
Bahrain	Deaths	Female	2015	5.473232	7.435892	3.752145	
Bahrain	Deaths	Female	2016	5.631426	7.584324	3.826608	

Bahrain	Deaths	Female	2017	5.735523	7.755535	3.909255	
Bahrain	Deaths	Female	2018	5.760748	7.82275	4.061313	
Bahrain	Deaths	Female	2019	5.688592	7.62279	4.077465	
Bahrain	Deaths	Female	2020	5.538829	7.419545	3.979057	
Bahrain	Deaths	Female	2021	5.834204	7.650854	4.28203	
Bahrain	Deaths	Female	2022	5.896414	7.869994	4.126837	
Bahrain	Deaths	Female	2023	6.611943	9.434495	4.600564	
Bahrain	DALYs	Male	2010	276.9555	360.4395	218.3494	
Bahrain	DALYs	Male	2011	278.0818	368.8013	222.1697	
Bahrain	DALYs	Male	2012	281.993	379.9826	223.7805	
Bahrain	DALYs	Male	2013	280.064	377.5446	219.4766	
Bahrain	DALYs	Male	2014	281.3414	387.5204	215.2993	
Bahrain	DALYs	Male	2015	304.8824	427.3637	236.0895	
Bahrain	DALYs	Male	2016	313.6179	438.6496	239.1338	
Bahrain	DALYs	Male	2017	321.4621	434.6903	241.5469	
Bahrain	DALYs	Male	2018	340.4329	460.2077	255.7759	
Bahrain	DALYs	Male	2019	353.0216	488.0448	261.4451	
Bahrain	DALYs	Male	2020	358.6254	497.3757	265.0405	
Bahrain	DALYs	Male	2021	407.2471	578.7504	301.7126	
Bahrain	DALYs	Male	2022	469.4969	667.8645	328.9295	
Bahrain	DALYs	Male	2023	529.3295	738.3412	360.5115	
Bahrain	DALYs	Female	2010	149.8536	202.7071	111.3546	
Bahrain	DALYs	Female	2011	151.5008	203.3586	110.7181	
Bahrain	DALYs	Female	2012	154.9458	210.5228	109.5671	
Bahrain	DALYs	Female	2013	154.5343	211.0168	111.0404	
Bahrain	DALYs	Female	2014	151.8837	204.6391	105.8046	
Bahrain	DALYs	Female	2015	158.7511	214.1925	106.039	
Bahrain	DALYs	Female	2016	162.8272	221.6236	108.1703	
Bahrain	DALYs	Female	2017	164.4887	223.3898	110.3238	
Bahrain	DALYs	Female	2018	164.2863	225.4472	114.5928	
Bahrain	DALYs	Female	2019	162.4086	217.5644	116.5071	
Bahrain	DALYs	Female	2020	156.3037	209.8628	112.6972	
Bahrain	DALYs	Female	2021	165.2559	217.6208	121.3798	
Bahrain	DALYs	Female	2022	166.5603	224.3074	117.9845	
Bahrain	DALYs	Female	2023	186.0751	263.068	130.2376	
Bangladesh	Incidence	Male	2010	8.046062	12.23739	5.260168	

Bangladesh	Incidence	Male	2011	8.520782	12.72836	5.71375	
Bangladesh	Incidence	Male	2012	9.22887	12.83971	6.171585	
Bangladesh	Incidence	Male	2013	9.788636	13.86778	6.601355	
Bangladesh	Incidence	Male	2014	10.40466	14.7947	6.972255	
Bangladesh	Incidence	Male	2015	10.83996	15.24346	6.942275	
Bangladesh	Incidence	Male	2016	11.32106	15.95105	7.334265	
Bangladesh	Incidence	Male	2017	11.64568	16.63994	7.603907	
Bangladesh	Incidence	Male	2018	12.1458	17.70103	8.102464	
Bangladesh	Incidence	Male	2019	12.44517	18.13977	8.143021	
Bangladesh	Incidence	Male	2020	12.47632	18.351	8.352349	
Bangladesh	Incidence	Male	2021	13.83511	20.32821	8.768123	
Bangladesh	Incidence	Male	2022	14.7801	22.00761	9.372385	
Bangladesh	Incidence	Male	2023	15.31947	23.04571	8.76213	
Bangladesh	Incidence	Female	2010	2.323681	3.482437	1.533598	
Bangladesh	Incidence	Female	2011	2.38281	3.535636	1.601809	
Bangladesh	Incidence	Female	2012	2.502102	3.684225	1.667279	
Bangladesh	Incidence	Female	2013	2.66721	3.882926	1.76481	
Bangladesh	Incidence	Female	2014	2.847569	4.180633	1.866997	
Bangladesh	Incidence	Female	2015	3.025494	4.389184	1.957709	
Bangladesh	Incidence	Female	2016	3.225633	4.540667	2.108087	
Bangladesh	Incidence	Female	2017	3.428728	4.92125	2.313286	
Bangladesh	Incidence	Female	2018	3.64198	5.188681	2.488238	
Bangladesh	Incidence	Female	2019	3.877402	5.596175	2.642622	
Bangladesh	Incidence	Female	2020	4.101022	5.922719	2.740788	
Bangladesh	Incidence	Female	2021	4.747846	6.84017	3.08446	
Bangladesh	Incidence	Female	2022	5.068769	7.293484	3.437117	
Bangladesh	Incidence	Female	2023	5.510558	8.240017	3.576642	
Bangladesh	Deaths	Male	2010	7.847737	11.88936	5.117491	
Bangladesh	Deaths	Male	2011	8.321965	12.41655	5.550384	
Bangladesh	Deaths	Male	2012	9.019214	12.46865	6.022116	
Bangladesh	Deaths	Male	2013	9.557034	13.59449	6.429547	
Bangladesh	Deaths	Male	2014	10.13921	14.4447	6.773192	
Bangladesh	Deaths	Male	2015	10.55054	14.72274	6.759847	
Bangladesh	Deaths	Male	2016	11.00235	15.57472	7.142595	
Bangladesh	Deaths	Male	2017	11.29675	16.19697	7.439839	
Bangladesh	Deaths	Male	2018	11.76894	17.21489	7.807344	

Bangladesh	Deaths	Male	2019	12.05055	17.71604	7.940106	
Bangladesh	Deaths	Male	2020	12.10972	17.79618	8.143497	
Bangladesh	Deaths	Male	2021	13.40302	19.63289	8.51703	
Bangladesh	Deaths	Male	2022	14.22609	20.98808	9.00831	
Bangladesh	Deaths	Male	2023	14.69827	22.23161	8.382673	
Bangladesh	Deaths	Female	2010	2.268141	3.413666	1.505785	
Bangladesh	Deaths	Female	2011	2.325287	3.441824	1.559135	
Bangladesh	Deaths	Female	2012	2.439978	3.592673	1.628761	
Bangladesh	Deaths	Female	2013	2.596068	3.793939	1.715151	
Bangladesh	Deaths	Female	2014	2.764423	4.066174	1.810455	
Bangladesh	Deaths	Female	2015	2.931669	4.259342	1.887838	
Bangladesh	Deaths	Female	2016	3.118492	4.402516	2.0469	
Bangladesh	Deaths	Female	2017	3.308503	4.719116	2.228296	
Bangladesh	Deaths	Female	2018	3.507735	5.010525	2.388677	
Bangladesh	Deaths	Female	2019	3.730051	5.411085	2.539319	
Bangladesh	Deaths	Female	2020	3.941253	5.717386	2.621191	
Bangladesh	Deaths	Female	2021	4.530166	6.541147	2.924433	
Bangladesh	Deaths	Female	2022	4.820521	6.984715	3.26566	
Bangladesh	Deaths	Female	2023	5.220179	7.891065	3.381648	
Bangladesh	DALYs	Male	2010	227.1885	348.9706	148.6299	
Bangladesh	DALYs	Male	2011	239.5274	357.7042	158.7938	
Bangladesh	DALYs	Male	2012	258.7808	367.319	171.3425	
Bangladesh	DALYs	Male	2013	274.3692	388.4301	184.9708	
Bangladesh	DALYs	Male	2014	291.8812	414.9523	194.9389	
Bangladesh	DALYs	Male	2015	304.0509	431.4926	193.8669	
Bangladesh	DALYs	Male	2016	317.851	448.7091	203.298	
Bangladesh	DALYs	Male	2017	327.5948	469.5096	210.5722	
Bangladesh	DALYs	Male	2018	342.1181	501.1546	227.0255	
Bangladesh	DALYs	Male	2019	350.6229	509.6217	229.5415	
Bangladesh	DALYs	Male	2020	348.1377	509.5629	226.9803	
Bangladesh	DALYs	Male	2021	385.3839	565.24	242.29	
Bangladesh	DALYs	Male	2022	417.3101	632.1053	263.5067	
Bangladesh	DALYs	Male	2023	433.7188	654.4543	248.1857	
Bangladesh	DALYs	Female	2010	65.92327	98.77453	43.68493	
Bangladesh	DALYs	Female	2011	67.5295	100.0664	45.11515	
Bangladesh	DALYs	Female	2012	70.86998	104.8452	47.74195	

Bangladesh	DALYs	Female	2013	75.59372	110.7903	50.98254	
Bangladesh	DALYs	Female	2014	80.81924	119.1671	53.65487	
Bangladesh	DALYs	Female	2015	85.91747	125.1615	56.51033	
Bangladesh	DALYs	Female	2016	91.78222	129.611	60.1805	
Bangladesh	DALYs	Female	2017	97.68645	140.7827	65.64826	
Bangladesh	DALYs	Female	2018	104.0186	147.9898	71.80679	
Bangladesh	DALYs	Female	2019	110.7781	159.4846	77.01031	
Bangladesh	DALYs	Female	2020	116.9577	166.899	77.95969	
Bangladesh	DALYs	Female	2021	136.6987	195.6555	89.94008	
Bangladesh	DALYs	Female	2022	146.3219	207.7561	99.21043	
Bangladesh	DALYs	Female	2023	159.4839	236.5637	105.0859	
Barbados	Incidence	Male	2010	17.2742	20.61935	15.11374	
Barbados	Incidence	Male	2011	17.29163	20.66381	14.91414	
Barbados	Incidence	Male	2012	17.72819	20.87071	15.03721	
Barbados	Incidence	Male	2013	17.86431	21.04741	15.10448	
Barbados	Incidence	Male	2014	18.41254	21.8932	15.56408	
Barbados	Incidence	Male	2015	19.31613	22.9893	15.95887	
Barbados	Incidence	Male	2016	19.93326	23.41962	16.57455	
Barbados	Incidence	Male	2017	20.10954	23.86087	16.90352	
Barbados	Incidence	Male	2018	20.54543	24.24074	17.41487	
Barbados	Incidence	Male	2019	21.09672	24.92432	17.80261	
Barbados	Incidence	Male	2020	22.39733	26.34632	18.97628	
Barbados	Incidence	Male	2021	23.4649	28.04688	19.76644	
Barbados	Incidence	Male	2022	24.60821	29.47093	20.7291	
Barbados	Incidence	Male	2023	22.9494	28.06277	18.87611	
Barbados	Incidence	Female	2010	8.243976	9.756183	6.977424	
Barbados	Incidence	Female	2011	8.673646	10.23491	7.269356	
Barbados	Incidence	Female	2012	8.630776	10.24048	7.145291	
Barbados	Incidence	Female	2013	8.848608	10.45318	7.256632	
Barbados	Incidence	Female	2014	9.081322	10.83908	7.472763	
Barbados	Incidence	Female	2015	9.341539	11.20514	7.653503	
Barbados	Incidence	Female	2016	9.644572	11.53527	8.00346	
Barbados	Incidence	Female	2017	9.914885	11.75949	8.249738	
Barbados	Incidence	Female	2018	10.2005	11.96519	8.60496	
Barbados	Incidence	Female	2019	10.59888	12.36503	8.940899	
Barbados	Incidence	Female	2020	11.08186	12.94013	9.298367	

Barbados	Incidence	Female	2021	11.76376	13.85374	9.872285	
Barbados	Incidence	Female	2022	12.29214	14.44871	9.974557	
Barbados	Incidence	Female	2023	12.82179	15.22146	10.40175	
Barbados	Deaths	Male	2010	17.04212	20.44867	15.01404	
Barbados	Deaths	Male	2011	16.99985	20.24892	14.77042	
Barbados	Deaths	Male	2012	17.31655	20.49538	14.76102	
Barbados	Deaths	Male	2013	17.46372	20.6087	14.7844	
Barbados	Deaths	Male	2014	17.99754	21.19835	15.25273	
Barbados	Deaths	Male	2015	18.87376	22.55028	15.73809	
Barbados	Deaths	Male	2016	19.45942	22.78149	16.2183	
Barbados	Deaths	Male	2017	19.6286	23.04229	16.49074	
Barbados	Deaths	Male	2018	20.04355	23.42079	16.9581	
Barbados	Deaths	Male	2019	20.52705	24.1497	17.43779	
Barbados	Deaths	Male	2020	21.75037	25.53165	18.45214	
Barbados	Deaths	Male	2021	22.72831	27.22621	19.06722	
Barbados	Deaths	Male	2022	23.84544	28.4456	19.889	
Barbados	Deaths	Male	2023	22.1475	26.96424	18.27023	
Barbados	Deaths	Female	2010	8.057981	9.441425	6.856779	
Barbados	Deaths	Female	2011	8.46387	10.04073	7.225163	
Barbados	Deaths	Female	2012	8.363099	9.953768	7.010987	
Barbados	Deaths	Female	2013	8.568793	10.15131	7.12364	
Barbados	Deaths	Female	2014	8.778918	10.32442	7.293841	
Barbados	Deaths	Female	2015	9.013588	10.64969	7.497914	
Barbados	Deaths	Female	2016	9.281692	10.94398	7.760895	
Barbados	Deaths	Female	2017	9.527667	11.26953	8.01221	
Barbados	Deaths	Female	2018	9.788306	11.45586	8.249374	
Barbados	Deaths	Female	2019	10.13611	11.89103	8.594701	
Barbados	Deaths	Female	2020	10.54743	12.35735	8.814233	
Barbados	Deaths	Female	2021	11.12523	12.9337	9.228076	
Barbados	Deaths	Female	2022	11.60234	13.67648	9.56552	
Barbados	Deaths	Female	2023	12.08205	14.33155	9.789016	
Barbados	DALYs	Male	2010	403.5164	480.8079	352.2829	
Barbados	DALYs	Male	2011	405.7485	483.4798	349.1158	
Barbados	DALYs	Male	2012	418.8582	496.1283	356.1699	
Barbados	DALYs	Male	2013	418.445	493.6526	353.2805	
Barbados	DALYs	Male	2014	430.9792	505.5403	364.4936	

Barbados	DALYs	Male	2015	451.2202	538.5324	375.6999	
Barbados	DALYs	Male	2016	464.0433	543.2846	385.36	
Barbados	DALYs	Male	2017	466.6576	547.9836	393.7401	
Barbados	DALYs	Male	2018	475.7779	553.0043	402.658	
Barbados	DALYs	Male	2019	487.8635	571.6707	415.1283	
Barbados	DALYs	Male	2020	514.3632	601.0343	437.5896	
Barbados	DALYs	Male	2021	535.437	637.2815	448.7939	
Barbados	DALYs	Male	2022	555.9917	663.8061	464.7983	
Barbados	DALYs	Male	2023	518.7039	628.5868	428.1867	
Barbados	DALYs	Female	2010	180.1566	209.3467	152.4846	
Barbados	DALYs	Female	2011	188.8195	221.6722	159.966	
Barbados	DALYs	Female	2012	188.7308	223.6114	156.4257	
Barbados	DALYs	Female	2013	192.5638	228.018	160.7761	
Barbados	DALYs	Female	2014	197.6388	232.9647	163.3995	
Barbados	DALYs	Female	2015	202.9509	241.2446	167.0276	
Barbados	DALYs	Female	2016	208.7706	248.6921	172.7572	
Barbados	DALYs	Female	2017	213.8615	253.3113	179.6551	
Barbados	DALYs	Female	2018	219.0985	257.7642	186.7823	
Barbados	DALYs	Female	2019	226.2156	265.3542	190.7631	
Barbados	DALYs	Female	2020	234.903	274.4153	198.2059	
Barbados	DALYs	Female	2021	248.4187	288.7392	205.1598	
Barbados	DALYs	Female	2022	256.2821	302.5279	210.0826	
Barbados	DALYs	Female	2023	264.8206	313.0766	214.2612	
Belarus	Incidence	Male	2010	74.69685	80.27552	69.89851	
Belarus	Incidence	Male	2011	71.9593	77.71033	66.86466	
Belarus	Incidence	Male	2012	69.70959	75.57614	64.08109	
Belarus	Incidence	Male	2013	68.26386	73.89738	62.53574	
Belarus	Incidence	Male	2014	69.14001	75.15449	63.1644	
Belarus	Incidence	Male	2015	61.80851	68.79946	56.46837	
Belarus	Incidence	Male	2016	65.42807	72.6961	58.8864	
Belarus	Incidence	Male	2017	72.53502	80.30124	65.85501	
Belarus	Incidence	Male	2018	77.02509	84.4595	69.7271	
Belarus	Incidence	Male	2019	77.83354	85.78228	69.51619	
Belarus	Incidence	Male	2020	77.39068	86.9381	68.62536	
Belarus	Incidence	Male	2021	80.03832	90.40698	70.45179	
Belarus	Incidence	Male	2022	84.08084	94.22236	73.38437	

Belarus	Incidence	Male	2023	84.93998	97.2471	73.72938	
Belarus	Incidence	Female	2010	9.783729	10.84371	9.003372	
Belarus	Incidence	Female	2011	9.633517	10.66164	8.904683	
Belarus	Incidence	Female	2012	9.516429	10.54582	8.842637	
Belarus	Incidence	Female	2013	9.245591	10.09276	8.537257	
Belarus	Incidence	Female	2014	9.244154	10.10639	8.495373	
Belarus	Incidence	Female	2015	8.927203	10.00103	8.046139	
Belarus	Incidence	Female	2016	9.416958	10.56323	8.482839	
Belarus	Incidence	Female	2017	10.19772	11.22659	9.30925	
Belarus	Incidence	Female	2018	10.55981	11.63612	9.640944	
Belarus	Incidence	Female	2019	10.87448	11.83419	9.902124	
Belarus	Incidence	Female	2020	10.74725	11.79335	9.627055	
Belarus	Incidence	Female	2021	11.08832	12.27976	9.827745	
Belarus	Incidence	Female	2022	12.07731	13.72272	10.75918	
Belarus	Incidence	Female	2023	12.58832	14.23124	11.10027	
Belarus	Deaths	Male	2010	68.91373	73.56811	64.61422	
Belarus	Deaths	Male	2011	65.97976	70.93451	61.53176	
Belarus	Deaths	Male	2012	63.2245	68.2861	58.77274	
Belarus	Deaths	Male	2013	61.15968	65.86179	56.71831	
Belarus	Deaths	Male	2014	61.20152	66.24099	56.52385	
Belarus	Deaths	Male	2015	54.3913	59.73871	50.17817	
Belarus	Deaths	Male	2016	57.44703	63.27015	52.56174	
Belarus	Deaths	Male	2017	63.54833	69.55585	58.04825	
Belarus	Deaths	Male	2018	67.2738	73.13998	61.8744	
Belarus	Deaths	Male	2019	67.78369	73.44603	61.35014	
Belarus	Deaths	Male	2020	67.20518	74.33046	60.42312	
Belarus	Deaths	Male	2021	69.25529	76.33297	61.26587	
Belarus	Deaths	Male	2022	72.66665	80.56483	63.57056	
Belarus	Deaths	Male	2023	73.3135	82.73612	64.39662	
Belarus	Deaths	Female	2010	9.206107	10.00244	8.479087	
Belarus	Deaths	Female	2011	8.985602	9.727013	8.344801	
Belarus	Deaths	Female	2012	8.733478	9.400986	8.164416	
Belarus	Deaths	Female	2013	8.351038	8.892292	7.807283	
Belarus	Deaths	Female	2014	8.246903	8.75665	7.658413	
Belarus	Deaths	Female	2015	7.922497	8.690558	7.242402	
Belarus	Deaths	Female	2016	8.325105	8.951623	7.677414	

Belarus	Deaths	Female	2017	8.968753	9.634653	8.369435	
Belarus	Deaths	Female	2018	9.243634	9.916115	8.654224	
Belarus	Deaths	Female	2019	9.477798	10.13984	8.836141	
Belarus	Deaths	Female	2020	9.33721	10.06839	8.58828	
Belarus	Deaths	Female	2021	9.576954	10.35007	8.684828	
Belarus	Deaths	Female	2022	10.38123	11.44637	9.387883	
Belarus	Deaths	Female	2023	10.79569	12.03636	9.585517	
Belarus	DALYs	Male	2010	1861.954	1991.694	1740.182	
Belarus	DALYs	Male	2011	1776.786	1915.573	1656.303	
Belarus	DALYs	Male	2012	1705.109	1848.096	1581.017	
Belarus	DALYs	Male	2013	1650.829	1783.931	1523.764	
Belarus	DALYs	Male	2014	1661.482	1799.192	1533.145	
Belarus	DALYs	Male	2015	1469.259	1619.357	1354.186	
Belarus	DALYs	Male	2016	1536.821	1697.345	1402.294	
Belarus	DALYs	Male	2017	1683.929	1844.102	1535.747	
Belarus	DALYs	Male	2018	1782.879	1941.479	1629.679	
Belarus	DALYs	Male	2019	1798.343	1953.49	1619.793	
Belarus	DALYs	Male	2020	1778.779	1974.55	1583.194	
Belarus	DALYs	Male	2021	1832.934	2030.78	1616.044	
Belarus	DALYs	Male	2022	1920.287	2131.806	1664.506	
Belarus	DALYs	Male	2023	1933.533	2183.144	1675.607	
Belarus	DALYs	Female	2010	212.7601	232.4321	196.8664	
Belarus	DALYs	Female	2011	206.9891	225.2645	192.5599	
Belarus	DALYs	Female	2012	201.7271	218.7504	188.4166	
Belarus	DALYs	Female	2013	192.9457	206.1365	179.9465	
Belarus	DALYs	Female	2014	190.8275	204.726	176.223	
Belarus	DALYs	Female	2015	181.5821	200.2718	166.8754	
Belarus	DALYs	Female	2016	189.9914	205.8764	174.6881	
Belarus	DALYs	Female	2017	204.9819	220.8826	192.9721	
Belarus	DALYs	Female	2018	212.2694	227.9049	200.6404	
Belarus	DALYs	Female	2019	218.8613	235.8557	205.3318	
Belarus	DALYs	Female	2020	215.2141	233.6961	198.9787	
Belarus	DALYs	Female	2021	222.1292	242.8752	201.8943	
Belarus	DALYs	Female	2022	243.0587	270.1783	219.2647	
Belarus	DALYs	Female	2023	252.8726	282.4585	224.4956	
Belgium	Incidence	Male	2010	115.434	123.6294	106.1452	

Belgium	Incidence	Male	2011	115.2401	124.0835	106.7333	
Belgium	Incidence	Male	2012	114.9671	123.7291	106.4709	
Belgium	Incidence	Male	2013	114.6572	123.6877	106.2327	
Belgium	Incidence	Male	2014	112.2691	122.508	103.0343	
Belgium	Incidence	Male	2015	106.1411	115.4065	98.04355	
Belgium	Incidence	Male	2016	104.6739	113.8933	95.48177	
Belgium	Incidence	Male	2017	100.3633	109.6142	90.11351	
Belgium	Incidence	Male	2018	97.43736	106.2102	87.30073	
Belgium	Incidence	Male	2019	93.02168	101.3536	83.48872	
Belgium	Incidence	Male	2020	90.68453	99.61033	82.07997	
Belgium	Incidence	Male	2021	91.87882	101.2347	82.70648	
Belgium	Incidence	Male	2022	93.9905	105.1865	84.38188	
Belgium	Incidence	Male	2023	90.81486	101.6288	81.76538	
Belgium	Incidence	Female	2010	41.30383	45.9747	36.99925	
Belgium	Incidence	Female	2011	42.85426	47.91918	38.19214	
Belgium	Incidence	Female	2012	43.88665	48.69395	38.93212	
Belgium	Incidence	Female	2013	45.65771	50.29525	40.13508	
Belgium	Incidence	Female	2014	46.56169	51.43061	41.34456	
Belgium	Incidence	Female	2015	46.5202	51.04086	41.25957	
Belgium	Incidence	Female	2016	46.55558	51.27925	41.49861	
Belgium	Incidence	Female	2017	45.97086	50.65451	40.71993	
Belgium	Incidence	Female	2018	46.5573	51.98649	40.7721	
Belgium	Incidence	Female	2019	46.8293	51.72911	40.77356	
Belgium	Incidence	Female	2020	47.45889	51.91915	42.07817	
Belgium	Incidence	Female	2021	47.06987	51.88113	41.92055	
Belgium	Incidence	Female	2022	50.81733	56.31723	44.77779	
Belgium	Incidence	Female	2023	49.23987	54.14237	43.1423	
Belgium	Deaths	Male	2010	107.4914	113.8192	100.6582	
Belgium	Deaths	Male	2011	107.3551	114.1168	100.598	
Belgium	Deaths	Male	2012	106.964	113.2974	100.2741	
Belgium	Deaths	Male	2013	106.2071	112.6337	99.697	
Belgium	Deaths	Male	2014	103.5536	109.8094	97.15399	
Belgium	Deaths	Male	2015	97.89359	104.3523	91.97382	
Belgium	Deaths	Male	2016	96.66722	103.8329	90.2276	
Belgium	Deaths	Male	2017	92.87283	100.17	85.95926	
Belgium	Deaths	Male	2018	90.43628	97.82875	83.99264	

Belgium	Deaths	Male	2019	86.21608	93.77761	79.52925	
Belgium	Deaths	Male	2020	83.58682	90.23197	77.46291	
Belgium	Deaths	Male	2021	84.13632	91.71387	76.97811	
Belgium	Deaths	Male	2022	86.366	96.14066	79.05402	
Belgium	Deaths	Male	2023	83.56178	93.15673	75.88098	
Belgium	Deaths	Female	2010	36.72063	39.91664	32.69864	
Belgium	Deaths	Female	2011	37.93502	41.1405	33.80337	
Belgium	Deaths	Female	2012	38.79292	42.20822	34.62821	
Belgium	Deaths	Female	2013	40.12979	43.54593	35.84662	
Belgium	Deaths	Female	2014	40.76395	44.16823	36.70789	
Belgium	Deaths	Female	2015	40.76727	43.87239	36.362	
Belgium	Deaths	Female	2016	40.76622	43.99906	36.3585	
Belgium	Deaths	Female	2017	40.35459	43.40681	35.94245	
Belgium	Deaths	Female	2018	41.0199	44.34296	36.49518	
Belgium	Deaths	Female	2019	41.13127	44.69293	36.54298	
Belgium	Deaths	Female	2020	41.47634	44.62585	37.39814	
Belgium	Deaths	Female	2021	40.64053	43.85197	36.67586	
Belgium	Deaths	Female	2022	44.15228	47.61516	39.52311	
Belgium	Deaths	Female	2023	42.72429	46.32283	38.19654	
Belgium	DALYs	Male	2010	2361.008	2500.761	2228.915	
Belgium	DALYs	Male	2011	2339.037	2470.72	2208.455	
Belgium	DALYs	Male	2012	2310.208	2452.479	2181.784	
Belgium	DALYs	Male	2013	2285.047	2423.351	2160.173	
Belgium	DALYs	Male	2014	2223.76	2355.394	2102.302	
Belgium	DALYs	Male	2015	2090.111	2226.078	1974.631	
Belgium	DALYs	Male	2016	2045.534	2187.288	1928.509	
Belgium	DALYs	Male	2017	1951.932	2097.674	1830.123	
Belgium	DALYs	Male	2018	1876.945	2022.825	1751.161	
Belgium	DALYs	Male	2019	1778.764	1924.424	1650.225	
Belgium	DALYs	Male	2020	1723.779	1862.361	1607.216	
Belgium	DALYs	Male	2021	1744.471	1901.44	1611.542	
Belgium	DALYs	Male	2022	1772.317	1970.886	1629.226	
Belgium	DALYs	Male	2023	1705.164	1895.83	1568.218	
Belgium	DALYs	Female	2010	838.7526	897.3425	766.0926	
Belgium	DALYs	Female	2011	868.4534	930.2449	796.9679	
Belgium	DALYs	Female	2012	879.647	942.8417	811.7245	

Belgium	DALYs	Female	2013	903.406	971.7032	832.8843	
Belgium	DALYs	Female	2014	910.7368	972.6105	845.5932	
Belgium	DALYs	Female	2015	901.6915	960.5611	834.4811	
Belgium	DALYs	Female	2016	897.4031	958.5359	823.1834	
Belgium	DALYs	Female	2017	879.6977	940.6708	806.8512	
Belgium	DALYs	Female	2018	882.4964	947.7388	808.8924	
Belgium	DALYs	Female	2019	878.3206	945.0516	802.3303	
Belgium	DALYs	Female	2020	880.5137	937.3631	811.6957	
Belgium	DALYs	Female	2021	875.5172	932.8136	802.9318	
Belgium	DALYs	Female	2022	935.7052	996.8026	854.5891	
Belgium	DALYs	Female	2023	904.4447	972.5249	823.1005	
Belize	Incidence	Male	2010	10.39909	12.15441	9.168355	
Belize	Incidence	Male	2011	11.07204	12.87292	9.807339	
Belize	Incidence	Male	2012	10.84092	12.53502	9.561181	
Belize	Incidence	Male	2013	11.19628	12.79834	9.849206	
Belize	Incidence	Male	2014	11.3461	13.08846	10.07827	
Belize	Incidence	Male	2015	11.64146	13.45625	10.18835	
Belize	Incidence	Male	2016	11.64509	13.57352	10.11799	
Belize	Incidence	Male	2017	11.78968	13.67705	10.20804	
Belize	Incidence	Male	2018	11.96624	13.86974	10.32744	
Belize	Incidence	Male	2019	12.04085	13.97964	10.37511	
Belize	Incidence	Male	2020	12.63662	14.65996	10.71317	
Belize	Incidence	Male	2021	12.45645	14.37494	10.76522	
Belize	Incidence	Male	2022	12.06773	13.98289	10.2447	
Belize	Incidence	Male	2023	12.95274	14.89723	10.87174	
Belize	Incidence	Female	2010	4.146856	5.003656	3.516694	
Belize	Incidence	Female	2011	4.198877	5.098142	3.536098	
Belize	Incidence	Female	2012	4.249984	5.275275	3.568969	
Belize	Incidence	Female	2013	4.260596	5.249402	3.594958	
Belize	Incidence	Female	2014	4.277903	5.250959	3.60044	
Belize	Incidence	Female	2015	4.472676	5.443407	3.749443	
Belize	Incidence	Female	2016	4.621289	5.690888	3.806452	
Belize	Incidence	Female	2017	4.725506	5.85482	3.857413	
Belize	Incidence	Female	2018	4.845119	5.938712	4.05342	
Belize	Incidence	Female	2019	5.035576	6.15106	4.196907	
Belize	Incidence	Female	2020	5.142719	6.252229	4.303617	

Belize	Incidence	Female	2021	5.356738	6.562884	4.447588	
Belize	Incidence	Female	2022	5.216203	6.410526	4.353222	
Belize	Incidence	Female	2023	5.515894	6.696975	4.53803	
Belize	Deaths	Male	2010	10.10095	11.72918	8.958512	
Belize	Deaths	Male	2011	10.74721	12.45545	9.622086	
Belize	Deaths	Male	2012	10.50927	12.14245	9.295718	
Belize	Deaths	Male	2013	10.84987	12.32128	9.571066	
Belize	Deaths	Male	2014	10.97855	12.54083	9.762135	
Belize	Deaths	Male	2015	11.26891	12.98131	9.86937	
Belize	Deaths	Male	2016	11.32997	13.14486	9.867604	
Belize	Deaths	Male	2017	11.50781	13.2131	9.907022	
Belize	Deaths	Male	2018	11.68297	13.44542	10.03009	
Belize	Deaths	Male	2019	11.74735	13.5495	10.14697	
Belize	Deaths	Male	2020	12.31854	14.28955	10.50587	
Belize	Deaths	Male	2021	12.10764	14.05028	10.46915	
Belize	Deaths	Male	2022	11.70529	13.5393	9.992027	
Belize	Deaths	Male	2023	12.51091	14.49739	10.43164	
Belize	Deaths	Female	2010	3.975392	4.818529	3.368798	
Belize	Deaths	Female	2011	4.007647	4.905952	3.382717	
Belize	Deaths	Female	2012	4.034899	4.963785	3.420093	
Belize	Deaths	Female	2013	4.025132	4.921056	3.397667	
Belize	Deaths	Female	2014	4.045763	4.963974	3.420795	
Belize	Deaths	Female	2015	4.246958	5.175576	3.575612	
Belize	Deaths	Female	2016	4.36212	5.390109	3.634681	
Belize	Deaths	Female	2017	4.483316	5.533477	3.681429	
Belize	Deaths	Female	2018	4.58967	5.657131	3.842203	
Belize	Deaths	Female	2019	4.759679	5.823853	3.977399	
Belize	Deaths	Female	2020	4.838318	5.931131	4.076638	
Belize	Deaths	Female	2021	5.013951	6.13171	4.181016	
Belize	Deaths	Female	2022	4.878461	5.88724	4.085493	
Belize	Deaths	Female	2023	5.150583	6.268553	4.288995	
Belize	DALYs	Male	2010	279.1781	326.6399	246.7817	
Belize	DALYs	Male	2011	295.9533	344.1715	265.3189	
Belize	DALYs	Male	2012	290.3816	334.7305	256.2223	
Belize	DALYs	Male	2013	299.3391	342.7574	265.0453	
Belize	DALYs	Male	2014	302.7578	348.0937	266.6454	

Belize	DALYs	Male	2015	309.8865	358.3349	271.3482	
Belize	DALYs	Male	2016	307.0246	357.6666	266.2284	
Belize	DALYs	Male	2017	310.3664	359.9825	268.0238	
Belize	DALYs	Male	2018	314.4056	362.839	271.3156	
Belize	DALYs	Male	2019	315.1457	368.0135	273.6017	
Belize	DALYs	Male	2020	329.6946	383.5468	280.9726	
Belize	DALYs	Male	2021	325.4887	378.6311	281.302	
Belize	DALYs	Male	2022	313.6573	365.2702	266.625	
Belize	DALYs	Male	2023	335.3896	386.178	280.1539	
Belize	DALYs	Female	2010	109.9373	132.3481	92.98793	
Belize	DALYs	Female	2011	111.766	136.9202	94.20828	
Belize	DALYs	Female	2012	113.2796	140.9971	95.1153	
Belize	DALYs	Female	2013	114.3189	141.0616	96.00294	
Belize	DALYs	Female	2014	114.4594	139.9401	96.88607	
Belize	DALYs	Female	2015	119.2377	146.4741	100.4111	
Belize	DALYs	Female	2016	123.2907	152.7182	102.3293	
Belize	DALYs	Female	2017	125.5966	156.0677	103.7043	
Belize	DALYs	Female	2018	128.6631	158.5235	106.5177	
Belize	DALYs	Female	2019	133.4131	163.6793	111.5441	
Belize	DALYs	Female	2020	136.1018	167.1002	113.8782	
Belize	DALYs	Female	2021	142.08	174.5863	117.7644	
Belize	DALYs	Female	2022	137.347	168.1719	114.4645	
Belize	DALYs	Female	2023	143.7446	174.5971	118.6084	
Benin	Incidence	Male	2010	0.877762	1.249989	0.623597	
Benin	Incidence	Male	2011	0.893407	1.230194	0.611734	
Benin	Incidence	Male	2012	0.897008	1.216402	0.607042	
Benin	Incidence	Male	2013	0.887971	1.227192	0.623081	
Benin	Incidence	Male	2014	0.888765	1.240068	0.614513	
Benin	Incidence	Male	2015	0.955704	1.367905	0.629776	
Benin	Incidence	Male	2016	0.934555	1.3447	0.629739	
Benin	Incidence	Male	2017	0.937245	1.360882	0.640854	
Benin	Incidence	Male	2018	0.925609	1.353427	0.634903	
Benin	Incidence	Male	2019	0.941725	1.415027	0.628942	
Benin	Incidence	Male	2020	0.972868	1.450402	0.643658	
Benin	Incidence	Male	2021	0.958707	1.431609	0.652075	
Benin	Incidence	Male	2022	0.970615	1.407291	0.659323	

Benin	Incidence	Male	2023	1.014302	1.497036	0.690312	
Benin	Incidence	Female	2010	0.335575	0.487615	0.21873	
Benin	Incidence	Female	2011	0.3449	0.494796	0.220611	
Benin	Incidence	Female	2012	0.349757	0.50214	0.225	
Benin	Incidence	Female	2013	0.357412	0.521918	0.233815	
Benin	Incidence	Female	2014	0.361974	0.511325	0.238401	
Benin	Incidence	Female	2015	0.370272	0.506209	0.242637	
Benin	Incidence	Female	2016	0.372608	0.510293	0.242953	
Benin	Incidence	Female	2017	0.380025	0.53696	0.255239	
Benin	Incidence	Female	2018	0.383572	0.524466	0.259451	
Benin	Incidence	Female	2019	0.402656	0.541345	0.275829	
Benin	Incidence	Female	2020	0.411281	0.565157	0.284425	
Benin	Incidence	Female	2021	0.409528	0.567729	0.277477	
Benin	Incidence	Female	2022	0.424595	0.578987	0.285802	
Benin	Incidence	Female	2023	0.457192	0.623498	0.311992	
Benin	Deaths	Male	2010	0.870255	1.240887	0.624358	
Benin	Deaths	Male	2011	0.88476	1.215402	0.611541	
Benin	Deaths	Male	2012	0.88748	1.199727	0.605671	
Benin	Deaths	Male	2013	0.878754	1.215978	0.614609	
Benin	Deaths	Male	2014	0.878229	1.219209	0.606841	
Benin	Deaths	Male	2015	0.94325	1.347669	0.627809	
Benin	Deaths	Male	2016	0.92048	1.318722	0.62069	
Benin	Deaths	Male	2017	0.922291	1.334763	0.629999	
Benin	Deaths	Male	2018	0.911559	1.332236	0.620268	
Benin	Deaths	Male	2019	0.927167	1.396403	0.621268	
Benin	Deaths	Male	2020	0.956841	1.42618	0.635403	
Benin	Deaths	Male	2021	0.942866	1.41024	0.642614	
Benin	Deaths	Male	2022	0.95407	1.391628	0.646311	
Benin	Deaths	Male	2023	0.997308	1.482112	0.679798	
Benin	Deaths	Female	2010	0.329297	0.477731	0.213676	
Benin	Deaths	Female	2011	0.338133	0.481209	0.216119	
Benin	Deaths	Female	2012	0.342326	0.48954	0.218814	
Benin	Deaths	Female	2013	0.34968	0.510021	0.228378	
Benin	Deaths	Female	2014	0.353623	0.502216	0.233181	
Benin	Deaths	Female	2015	0.361017	0.489853	0.236557	
Benin	Deaths	Female	2016	0.362391	0.496887	0.236015	

Benin	Deaths	Female	2017	0.369097	0.522021	0.246033	
Benin	Deaths	Female	2018	0.372218	0.513417	0.25208	
Benin	Deaths	Female	2019	0.390212	0.530517	0.268927	
Benin	Deaths	Female	2020	0.397714	0.55061	0.275131	
Benin	Deaths	Female	2021	0.395782	0.54761	0.270219	
Benin	Deaths	Female	2022	0.409527	0.558306	0.276768	
Benin	Deaths	Female	2023	0.44066	0.599758	0.300185	
Benin	DALYs	Male	2010	24.6255	34.95805	17.15053	
Benin	DALYs	Male	2011	25.1384	34.80313	17.04752	
Benin	DALYs	Male	2012	25.28887	34.55327	16.80524	
Benin	DALYs	Male	2013	25.04302	34.53189	17.53406	
Benin	DALYs	Male	2014	25.11903	35.29338	17.36756	
Benin	DALYs	Male	2015	27.04578	38.71491	18.18592	
Benin	DALYs	Male	2016	26.53348	38.73914	18.27535	
Benin	DALYs	Male	2017	26.62199	39.18311	18.25701	
Benin	DALYs	Male	2018	26.26603	38.849	17.99973	
Benin	DALYs	Male	2019	26.70664	39.74212	17.60308	
Benin	DALYs	Male	2020	27.5884	41.35612	18.11815	
Benin	DALYs	Male	2021	27.13668	40.42498	18.38212	
Benin	DALYs	Male	2022	27.40824	39.63325	18.71887	
Benin	DALYs	Male	2023	28.61619	41.94093	19.44394	
Benin	DALYs	Female	2010	9.968712	14.72183	6.460724	
Benin	DALYs	Female	2011	10.27193	14.92783	6.579267	
Benin	DALYs	Female	2012	10.45909	15.0666	6.679432	
Benin	DALYs	Female	2013	10.69694	15.67825	7.00184	
Benin	DALYs	Female	2014	10.85342	15.16816	7.156657	
Benin	DALYs	Female	2015	11.13788	15.35135	7.306535	
Benin	DALYs	Female	2016	11.25104	15.36389	7.304794	
Benin	DALYs	Female	2017	11.48849	16.25561	7.734683	
Benin	DALYs	Female	2018	11.61196	15.82134	7.821726	
Benin	DALYs	Female	2019	12.20483	16.40677	8.334929	
Benin	DALYs	Female	2020	12.47894	17.11108	8.58885	
Benin	DALYs	Female	2021	12.39622	16.9259	8.371568	
Benin	DALYs	Female	2022	12.83446	17.57966	8.751957	
Benin	DALYs	Female	2023	13.82188	18.8238	9.513189	
Bhutan	Incidence	Male	2010	4.476306	7.033197	2.818755	

Bhutan	Incidence	Male	2011	4.620916	7.140974	2.947152	
Bhutan	Incidence	Male	2012	4.810247	7.59981	3.12866	
Bhutan	Incidence	Male	2013	4.891153	7.716389	3.23218	
Bhutan	Incidence	Male	2014	5.061881	7.913541	3.406516	
Bhutan	Incidence	Male	2015	5.19042	8.124574	3.389068	
Bhutan	Incidence	Male	2016	5.40685	8.593365	3.400633	
Bhutan	Incidence	Male	2017	5.603723	9.045651	3.532449	
Bhutan	Incidence	Male	2018	5.83062	9.339734	3.754083	
Bhutan	Incidence	Male	2019	6.009337	9.840446	3.885186	
Bhutan	Incidence	Male	2020	6.204075	10.30354	4.050675	
Bhutan	Incidence	Male	2021	6.645278	10.68199	4.291993	
Bhutan	Incidence	Male	2022	7.021331	11.46131	4.334192	
Bhutan	Incidence	Male	2023	7.142748	11.77116	4.396442	
Bhutan	Incidence	Female	2010	3.271231	4.918077	2.097805	
Bhutan	Incidence	Female	2011	3.376353	4.979728	2.224439	
Bhutan	Incidence	Female	2012	3.504811	5.114026	2.306765	
Bhutan	Incidence	Female	2013	3.585086	5.171242	2.382982	
Bhutan	Incidence	Female	2014	3.696035	5.355959	2.41151	
Bhutan	Incidence	Female	2015	3.840035	5.496515	2.483335	
Bhutan	Incidence	Female	2016	4.033105	5.632618	2.610958	
Bhutan	Incidence	Female	2017	4.239626	5.897115	2.773281	
Bhutan	Incidence	Female	2018	4.421996	6.144791	2.924378	
Bhutan	Incidence	Female	2019	4.606597	6.326344	3.111127	
Bhutan	Incidence	Female	2020	4.876588	6.676211	3.240841	
Bhutan	Incidence	Female	2021	5.154833	7.126558	3.442144	
Bhutan	Incidence	Female	2022	5.469314	7.687387	3.612786	
Bhutan	Incidence	Female	2023	5.759434	8.20209	3.734964	
Bhutan	Deaths	Male	2010	4.384186	6.85481	2.750628	
Bhutan	Deaths	Male	2011	4.524212	6.992202	2.874183	
Bhutan	Deaths	Male	2012	4.707693	7.43448	3.057276	
Bhutan	Deaths	Male	2013	4.783717	7.535103	3.149542	
Bhutan	Deaths	Male	2014	4.947301	7.715389	3.314768	
Bhutan	Deaths	Male	2015	5.06967	8.016242	3.296889	
Bhutan	Deaths	Male	2016	5.2782	8.393447	3.345211	
Bhutan	Deaths	Male	2017	5.467496	8.88163	3.417997	
Bhutan	Deaths	Male	2018	5.684779	9.050094	3.630168	

Bhutan	Deaths	Male	2019	5.855829	9.590724	3.799804	
Bhutan	Deaths	Male	2020	6.043585	9.985808	3.934271	
Bhutan	Deaths	Male	2021	6.472948	10.33281	4.188539	
Bhutan	Deaths	Male	2022	6.828309	11.0777	4.242285	
Bhutan	Deaths	Male	2023	6.933034	11.40873	4.279034	
Bhutan	Deaths	Female	2010	3.177013	4.777578	2.036384	
Bhutan	Deaths	Female	2011	3.277871	4.807127	2.165282	
Bhutan	Deaths	Female	2012	3.399908	4.961372	2.242386	
Bhutan	Deaths	Female	2013	3.474007	5.028465	2.312155	
Bhutan	Deaths	Female	2014	3.576428	5.194023	2.331582	
Bhutan	Deaths	Female	2015	3.710783	5.284099	2.39806	
Bhutan	Deaths	Female	2016	3.893132	5.431183	2.534297	
Bhutan	Deaths	Female	2017	4.088238	5.731984	2.701897	
Bhutan	Deaths	Female	2018	4.258675	5.94844	2.816491	
Bhutan	Deaths	Female	2019	4.433087	6.082982	3.007999	
Bhutan	Deaths	Female	2020	4.686781	6.350352	3.128581	
Bhutan	Deaths	Female	2021	4.950952	6.82099	3.321186	
Bhutan	Deaths	Female	2022	5.240455	7.415018	3.421593	
Bhutan	Deaths	Female	2023	5.519363	7.780291	3.575215	
Bhutan	DALYs	Male	2010	128.64	204.3594	81.82794	
Bhutan	DALYs	Male	2011	132.8922	205.1864	86.28633	
Bhutan	DALYs	Male	2012	138.4467	217.32	89.51679	
Bhutan	DALYs	Male	2013	140.9495	221.3419	94.14585	
Bhutan	DALYs	Male	2014	145.9985	228.7228	95.8685	
Bhutan	DALYs	Male	2015	149.7905	230.1496	97.38637	
Bhutan	DALYs	Male	2016	156.011	251.6141	97.04697	
Bhutan	DALYs	Male	2017	161.5995	261.2103	103.2554	
Bhutan	DALYs	Male	2018	168.1817	270.3455	108.8012	
Bhutan	DALYs	Male	2019	173.3262	285.4908	112.2551	
Bhutan	DALYs	Male	2020	178.7737	294.333	114.476	
Bhutan	DALYs	Male	2021	191.1076	308.4445	122.7179	
Bhutan	DALYs	Male	2022	201.9976	329.1083	126.4776	
Bhutan	DALYs	Male	2023	205.9477	339.3344	125.462	
Bhutan	DALYs	Female	2010	94.47697	140.3227	60.68122	
Bhutan	DALYs	Female	2011	97.53755	143.7394	64.03856	
Bhutan	DALYs	Female	2012	101.3594	148.987	65.80554	

Bhutan	DALYs	Female	2013	103.8764	150.6539	68.53815	
Bhutan	DALYs	Female	2014	107.3564	155.4305	69.84005	
Bhutan	DALYs	Female	2015	111.7071	161.0976	71.78983	
Bhutan	DALYs	Female	2016	117.3982	163.9669	76.30882	
Bhutan	DALYs	Female	2017	123.4242	172.1019	80.07974	
Bhutan	DALYs	Female	2018	128.8404	179.6853	84.4872	
Bhutan	DALYs	Female	2019	134.216	183.242	89.68893	
Bhutan	DALYs	Female	2020	142.159	195.6946	93.98441	
Bhutan	DALYs	Female	2021	150.0477	209.1561	98.38918	
Bhutan	DALYs	Female	2022	159.489	220.5768	107.7257	
Bhutan	DALYs	Female	2023	167.5886	235.4278	108.9513	
Bolivia (Plurina	Incidence	Male	2010	10.51715	14.42882	7.291856	
Bolivia (Plurina	Incidence	Male	2011	10.75874	14.94173	7.602656	
Bolivia (Plurina	Incidence	Male	2012	11.11684	15.73224	8.013748	
Bolivia (Plurina	Incidence	Male	2013	11.45144	16.35806	8.535893	
Bolivia (Plurina	Incidence	Male	2014	11.81236	16.99821	8.653603	
Bolivia (Plurina	Incidence	Male	2015	12.12982	17.35172	8.614397	
Bolivia (Plurina	Incidence	Male	2016	12.55221	17.47012	8.638875	
Bolivia (Plurina	Incidence	Male	2017	12.90331	17.92812	9.05081	
Bolivia (Plurina	Incidence	Male	2018	13.39091	18.62193	9.061033	
Bolivia (Plurina	Incidence	Male	2019	13.88998	19.41989	9.145959	
Bolivia (Plurina	Incidence	Male	2020	20.87441	29.43432	14.15861	
Bolivia (Plurina	Incidence	Male	2021	19.15637	26.93639	12.88539	
Bolivia (Plurina	Incidence	Male	2022	15.31773	21.55535	10.2002	
Bolivia (Plurina	Incidence	Male	2023	15.16834	21.51376	10.27044	
Bolivia (Plurina	Incidence	Female	2010	5.763327	8.279014	3.877513	
Bolivia (Plurina	Incidence	Female	2011	5.994853	8.665067	3.96977	
Bolivia (Plurina	Incidence	Female	2012	6.242754	8.963693	4.138141	
Bolivia (Plurina	Incidence	Female	2013	6.462627	9.104681	4.172993	
Bolivia (Plurina	Incidence	Female	2014	6.6339	9.273169	4.251697	
Bolivia (Plurina	Incidence	Female	2015	6.888343	9.492185	4.404301	
Bolivia (Plurina	Incidence	Female	2016	7.223871	9.823649	4.696159	
Bolivia (Plurina	Incidence	Female	2017	7.510882	10.19164	4.99825	
Bolivia (Plurina	Incidence	Female	2018	7.870303	10.35713	5.233945	
Bolivia (Plurina	Incidence	Female	2019	8.216244	10.9203	5.607017	
Bolivia (Plurina	Incidence	Female	2020	8.405026	11.48394	5.787646	

Bolivia (Plurina	Incidence	Female	2021	8.27726	11.38464	5.863674	
Bolivia (Plurina	Incidence	Female	2022	8.784757	11.98071	6.186703	
Bolivia (Plurina	Incidence	Female	2023	9.019196	12.75666	6.270748	
Bolivia (Plurina	Deaths	Male	2010	10.59618	14.4443	7.363303	
Bolivia (Plurina	Deaths	Male	2011	10.83699	14.98229	7.656753	
Bolivia (Plurina	Deaths	Male	2012	11.19153	15.76375	8.056464	
Bolivia (Plurina	Deaths	Male	2013	11.52388	16.40139	8.555484	
Bolivia (Plurina	Deaths	Male	2014	11.88444	17.12576	8.731901	
Bolivia (Plurina	Deaths	Male	2015	12.20012	17.54015	8.680101	
Bolivia (Plurina	Deaths	Male	2016	12.6209	17.54776	8.708809	
Bolivia (Plurina	Deaths	Male	2017	12.96719	17.98797	9.092517	
Bolivia (Plurina	Deaths	Male	2018	13.44769	18.6068	9.106055	
Bolivia (Plurina	Deaths	Male	2019	13.93801	19.36769	9.173579	
Bolivia (Plurina	Deaths	Male	2020	20.80441	29.17964	14.05746	
Bolivia (Plurina	Deaths	Male	2021	19.0023	26.58435	12.86898	
Bolivia (Plurina	Deaths	Male	2022	15.32409	21.43743	10.25582	
Bolivia (Plurina	Deaths	Male	2023	15.17907	21.43473	10.29939	
Bolivia (Plurina	Deaths	Female	2010	5.469313	7.773525	3.679196	
Bolivia (Plurina	Deaths	Female	2011	5.674273	8.15541	3.768555	
Bolivia (Plurina	Deaths	Female	2012	5.895216	8.389941	3.842209	
Bolivia (Plurina	Deaths	Female	2013	6.091182	8.618345	3.912064	
Bolivia (Plurina	Deaths	Female	2014	6.240049	8.810736	3.994106	
Bolivia (Plurina	Deaths	Female	2015	6.464324	8.618251	4.132842	
Bolivia (Plurina	Deaths	Female	2016	6.75998	9.059518	4.427126	
Bolivia (Plurina	Deaths	Female	2017	7.005745	9.446389	4.70917	
Bolivia (Plurina	Deaths	Female	2018	7.318121	9.591469	4.888636	
Bolivia (Plurina	Deaths	Female	2019	7.610545	10.15705	5.178057	
Bolivia (Plurina	Deaths	Female	2020	7.713693	10.54959	5.38044	
Bolivia (Plurina	Deaths	Female	2021	7.543944	10.36582	5.345398	
Bolivia (Plurina	Deaths	Female	2022	8.027054	11.14791	5.617736	
Bolivia (Plurina	Deaths	Female	2023	8.229984	11.83827	5.762175	
Bolivia (Plurina	DALYs	Male	2010	273.629	379.0982	189.7925	
Bolivia (Plurina	DALYs	Male	2011	279.1241	390.6478	197.8022	
Bolivia (Plurina	DALYs	Male	2012	287.8734	407.4476	208.3595	
Bolivia (Plurina	DALYs	Male	2013	296.1186	422.689	220.875	
Bolivia (Plurina	DALYs	Male	2014	304.8249	436.723	223.3175	

Bolivia (Plurina	DALYs	Male	2015	312.2937	443.013	221.1224	
Bolivia (Plurina	DALYs	Male	2016	322.2273	447.6498	220.2807	
Bolivia (Plurina	DALYs	Male	2017	330.188	461.7123	231.2255	
Bolivia (Plurina	DALYs	Male	2018	342.0042	480.9766	232.3816	
Bolivia (Plurina	DALYs	Male	2019	353.7513	497.9239	235.2684	
Bolivia (Plurina	DALYs	Male	2020	527.7676	749.9328	359.0477	
Bolivia (Plurina	DALYs	Male	2021	487.1771	693.5374	332.0641	
Bolivia (Plurina	DALYs	Male	2022	383.4022	539.0497	260.0965	
Bolivia (Plurina	DALYs	Male	2023	377.8354	536.4326	258.0188	
Bolivia (Plurina	DALYs	Female	2010	152.2448	219.0039	103.1393	
Bolivia (Plurina	DALYs	Female	2011	157.977	227.5555	105.319	
Bolivia (Plurina	DALYs	Female	2012	164.3852	234.452	109.6591	
Bolivia (Plurina	DALYs	Female	2013	169.963	241.2229	109.2654	
Bolivia (Plurina	DALYs	Female	2014	174.2975	245.4627	110.0906	
Bolivia (Plurina	DALYs	Female	2015	180.6196	240.9763	114.6617	
Bolivia (Plurina	DALYs	Female	2016	188.7769	253.3598	121.7452	
Bolivia (Plurina	DALYs	Female	2017	195.4341	263.3925	131.5239	
Bolivia (Plurina	DALYs	Female	2018	204.1207	271.4766	135.5139	
Bolivia (Plurina	DALYs	Female	2019	211.9577	285.8712	143.3883	
Bolivia (Plurina	DALYs	Female	2020	216.1143	297.0151	150.3668	
Bolivia (Plurina	DALYs	Female	2021	212.1913	292.0028	152.9963	
Bolivia (Plurina	DALYs	Female	2022	219.0682	304.8832	154.3274	
Bolivia (Plurina	DALYs	Female	2023	222.8285	320.3961	156.4692	
Bosnia Herzego	Incidence	Male	2010	99.1574	120.7514	82.41329	
Bosnia Herzego	Incidence	Male	2011	101.1723	123.279	84.93428	
Bosnia Herzego	Incidence	Male	2012	102.1147	124.4612	86.956	
Bosnia Herzego	Incidence	Male	2013	101.9577	124.3914	87.78589	
Bosnia Herzego	Incidence	Male	2014	102.0163	124.7002	88.3576	
Bosnia Herzego	Incidence	Male	2015	102.5392	125.7722	88.19312	
Bosnia Herzego	Incidence	Male	2016	102.0161	122.8466	88.29514	
Bosnia Herzego	Incidence	Male	2017	103.734	124.6151	90.34634	
Bosnia Herzego	Incidence	Male	2018	105.5048	126.7889	92.31541	
Bosnia Herzego	Incidence	Male	2019	106.8071	129.9863	92.98307	
Bosnia Herzego	Incidence	Male	2020	104.7109	127.7389	90.63834	
Bosnia Herzego	Incidence	Male	2021	105.2576	129.9314	90.61728	
Bosnia Herzego	Incidence	Male	2022	105.0452	131.3298	88.82054	

Bosnia Herzegc	Incidence	Male	2023	105.7863	136.6143	88.19848	
Bosnia Herzegc	Incidence	Female	2010	27.74418	33.94721	23.1491	
Bosnia Herzegc	Incidence	Female	2011	28.43277	34.52478	23.80592	
Bosnia Herzegc	Incidence	Female	2012	29.19462	34.67792	24.3366	
Bosnia Herzegc	Incidence	Female	2013	29.81197	35.52292	24.7977	
Bosnia Herzegc	Incidence	Female	2014	30.57579	36.26563	25.28535	
Bosnia Herzegc	Incidence	Female	2015	31.71872	37.1573	26.75928	
Bosnia Herzegc	Incidence	Female	2016	32.32131	38.12281	27.27966	
Bosnia Herzegc	Incidence	Female	2017	33.42936	39.81261	28.20938	
Bosnia Herzegc	Incidence	Female	2018	34.3055	41.00747	29.25393	
Bosnia Herzegc	Incidence	Female	2019	35.42207	42.98895	30.01001	
Bosnia Herzegc	Incidence	Female	2020	36.19711	43.41899	30.34391	
Bosnia Herzegc	Incidence	Female	2021	36.27584	42.62574	29.77218	
Bosnia Herzegc	Incidence	Female	2022	37.055	44.77157	29.9531	
Bosnia Herzegc	Incidence	Female	2023	37.19761	45.54192	29.69846	
Bosnia Herzegc	Deaths	Male	2010	97.63508	118.0707	82.07994	
Bosnia Herzegc	Deaths	Male	2011	99.73396	120.4612	84.10052	
Bosnia Herzegc	Deaths	Male	2012	100.8585	122.5133	86.08024	
Bosnia Herzegc	Deaths	Male	2013	100.8472	122.4528	86.94345	
Bosnia Herzegc	Deaths	Male	2014	101.0267	122.4815	87.57037	
Bosnia Herzegc	Deaths	Male	2015	101.6566	124.5624	87.51619	
Bosnia Herzegc	Deaths	Male	2016	101.109	121.7171	87.44697	
Bosnia Herzegc	Deaths	Male	2017	102.835	122.8945	89.48544	
Bosnia Herzegc	Deaths	Male	2018	104.5045	124.1842	92.03842	
Bosnia Herzegc	Deaths	Male	2019	105.773	127.6904	92.59379	
Bosnia Herzegc	Deaths	Male	2020	103.8035	126.6373	90.04794	
Bosnia Herzegc	Deaths	Male	2021	104.4273	128.5064	89.98089	
Bosnia Herzegc	Deaths	Male	2022	103.8648	129.6072	88.37137	
Bosnia Herzegc	Deaths	Male	2023	104.5892	133.9164	87.43671	
Bosnia Herzegc	Deaths	Female	2010	27.60958	33.7605	23.07068	
Bosnia Herzegc	Deaths	Female	2011	28.28377	34.40096	23.61092	
Bosnia Herzegc	Deaths	Female	2012	29.06252	34.5036	24.25737	
Bosnia Herzegc	Deaths	Female	2013	29.69292	35.23792	24.62612	
Bosnia Herzegc	Deaths	Female	2014	30.48929	36.35239	25.29841	
Bosnia Herzegc	Deaths	Female	2015	31.6755	37.19685	26.5493	
Bosnia Herzegc	Deaths	Female	2016	32.24613	38.26539	27.35879	

Bosnia Herzego	Deaths	Female	2017	33.36959	39.6824	28.38853	
Bosnia Herzego	Deaths	Female	2018	34.24492	41.05095	29.07508	
Bosnia Herzego	Deaths	Female	2019	35.37387	42.45706	29.90253	
Bosnia Herzego	Deaths	Female	2020	36.1847	43.26795	30.35676	
Bosnia Herzego	Deaths	Female	2021	36.11999	42.42935	29.58078	
Bosnia Herzego	Deaths	Female	2022	36.88413	44.72214	29.73796	
Bosnia Herzego	Deaths	Female	2023	37.16566	45.54877	29.45615	
Bosnia Herzego	DALYs	Male	2010	2497.932	3098.128	2084.767	
Bosnia Herzego	DALYs	Male	2011	2537.938	3167.047	2140.433	
Bosnia Herzego	DALYs	Male	2012	2548.729	3178.679	2150.743	
Bosnia Herzego	DALYs	Male	2013	2537.131	3151.291	2180.503	
Bosnia Herzego	DALYs	Male	2014	2533.946	3148.381	2186.423	
Bosnia Herzego	DALYs	Male	2015	2538.695	3172.645	2191.197	
Bosnia Herzego	DALYs	Male	2016	2515.922	3109.29	2182.001	
Bosnia Herzego	DALYs	Male	2017	2546.059	3137.612	2212.115	
Bosnia Herzego	DALYs	Male	2018	2580.8	3189.293	2262.701	
Bosnia Herzego	DALYs	Male	2019	2600.756	3264.22	2270.614	
Bosnia Herzego	DALYs	Male	2020	2529.6	3190.644	2194.483	
Bosnia Herzego	DALYs	Male	2021	2518.198	3199.762	2171.191	
Bosnia Herzego	DALYs	Male	2022	2517.716	3251.873	2149.452	
Bosnia Herzego	DALYs	Male	2023	2524.106	3332.395	2091.427	
Bosnia Herzego	DALYs	Female	2010	671.9603	820.0738	559.2769	
Bosnia Herzego	DALYs	Female	2011	687.4613	836.5839	575.5489	
Bosnia Herzego	DALYs	Female	2012	704.405	835.6347	584.5253	
Bosnia Herzego	DALYs	Female	2013	718.4853	848.2409	593.152	
Bosnia Herzego	DALYs	Female	2014	735.8806	863.3725	610.1211	
Bosnia Herzego	DALYs	Female	2015	760.5714	886.5526	636.7551	
Bosnia Herzego	DALYs	Female	2016	772.3495	895.1718	653.6631	
Bosnia Herzego	DALYs	Female	2017	793.9431	930.8703	672.5615	
Bosnia Herzego	DALYs	Female	2018	810.9036	964.8446	693.5651	
Bosnia Herzego	DALYs	Female	2019	832.3058	992.7438	704.1319	
Bosnia Herzego	DALYs	Female	2020	844.9004	1011.34	710.0957	
Bosnia Herzego	DALYs	Female	2021	846.0682	995.826	695.5831	
Bosnia Herzego	DALYs	Female	2022	861.0497	1037.542	690.2996	
Bosnia Herzego	DALYs	Female	2023	853.8114	1035.977	676.4964	
Botswana	Incidence	Male	2010	3.056529	4.404608	2.108485	

Botswana	Incidence	Male	2011	3.133815	4.489032	2.160992	
Botswana	Incidence	Male	2012	3.24614	4.735893	2.266754	
Botswana	Incidence	Male	2013	3.377153	4.935602	2.369757	
Botswana	Incidence	Male	2014	3.518219	5.10602	2.470561	
Botswana	Incidence	Male	2015	3.728208	5.402453	2.501803	
Botswana	Incidence	Male	2016	3.980666	5.793499	2.670924	
Botswana	Incidence	Male	2017	4.127638	5.963727	2.760983	
Botswana	Incidence	Male	2018	4.386995	6.396432	2.940895	
Botswana	Incidence	Male	2019	4.680936	6.849182	3.114838	
Botswana	Incidence	Male	2020	4.91825	7.146175	3.278299	
Botswana	Incidence	Male	2021	5.474981	7.906845	3.678736	
Botswana	Incidence	Male	2022	5.663262	8.19316	3.846452	
Botswana	Incidence	Male	2023	6.078429	8.817185	4.226022	
Botswana	Incidence	Female	2010	0.983021	1.362627	0.609228	
Botswana	Incidence	Female	2011	0.989722	1.359608	0.594634	
Botswana	Incidence	Female	2012	1.015138	1.414822	0.606958	
Botswana	Incidence	Female	2013	1.035629	1.464414	0.616656	
Botswana	Incidence	Female	2014	1.074069	1.509125	0.644026	
Botswana	Incidence	Female	2015	1.142852	1.602957	0.692217	
Botswana	Incidence	Female	2016	1.229777	1.760948	0.754979	
Botswana	Incidence	Female	2017	1.30676	1.799538	0.808547	
Botswana	Incidence	Female	2018	1.396171	1.920055	0.902574	
Botswana	Incidence	Female	2019	1.480164	2.066701	0.981724	
Botswana	Incidence	Female	2020	1.562582	2.177797	1.029554	
Botswana	Incidence	Female	2021	1.902429	2.569175	1.253931	
Botswana	Incidence	Female	2022	1.84661	2.52494	1.188944	
Botswana	Incidence	Female	2023	1.937039	2.680498	1.221058	
Botswana	Deaths	Male	2010	3.039399	4.393806	2.114018	
Botswana	Deaths	Male	2011	3.115662	4.514048	2.178557	
Botswana	Deaths	Male	2012	3.22673	4.659664	2.278238	
Botswana	Deaths	Male	2013	3.355525	4.934705	2.394634	
Botswana	Deaths	Male	2014	3.493847	5.042125	2.461879	
Botswana	Deaths	Male	2015	3.700076	5.339276	2.516903	
Botswana	Deaths	Male	2016	3.950227	5.661687	2.696777	
Botswana	Deaths	Male	2017	4.093532	5.835209	2.754968	
Botswana	Deaths	Male	2018	4.342396	6.232868	2.914103	

Botswana	Deaths	Male	2019	4.624719	6.701802	3.116253	
Botswana	Deaths	Male	2020	4.856944	7.158239	3.233881	
Botswana	Deaths	Male	2021	5.425729	7.842426	3.683972	
Botswana	Deaths	Male	2022	5.577656	8.084417	3.767647	
Botswana	Deaths	Male	2023	5.974782	8.602881	4.149338	
Botswana	Deaths	Female	2010	0.955494	1.366623	0.596209	
Botswana	Deaths	Female	2011	0.962667	1.315627	0.579625	
Botswana	Deaths	Female	2012	0.988426	1.381778	0.59264	
Botswana	Deaths	Female	2013	1.008918	1.422422	0.613001	
Botswana	Deaths	Female	2014	1.045764	1.48427	0.634683	
Botswana	Deaths	Female	2015	1.111458	1.566492	0.678899	
Botswana	Deaths	Female	2016	1.195138	1.724282	0.739799	
Botswana	Deaths	Female	2017	1.268074	1.76755	0.792446	
Botswana	Deaths	Female	2018	1.351698	1.884113	0.879863	
Botswana	Deaths	Female	2019	1.429724	1.986142	0.955003	
Botswana	Deaths	Female	2020	1.506085	2.080154	0.989772	
Botswana	Deaths	Female	2021	1.845225	2.492921	1.204852	
Botswana	Deaths	Female	2022	1.77129	2.421955	1.137208	
Botswana	Deaths	Female	2023	1.855085	2.58669	1.168025	
Botswana	DALYs	Male	2010	84.59256	121.618	58.5909	
Botswana	DALYs	Male	2011	86.77195	126.3258	59.46942	
Botswana	DALYs	Male	2012	89.81097	133.2723	61.69556	
Botswana	DALYs	Male	2013	93.35456	138.7145	64.13543	
Botswana	DALYs	Male	2014	97.23916	144.6002	66.65332	
Botswana	DALYs	Male	2015	102.9485	151.8972	69.01176	
Botswana	DALYs	Male	2016	109.6649	163.3818	72.12906	
Botswana	DALYs	Male	2017	113.5653	167.9896	74.45014	
Botswana	DALYs	Male	2018	120.8349	178.3723	79.87168	
Botswana	DALYs	Male	2019	129.0914	190.091	84.33468	
Botswana	DALYs	Male	2020	135.5511	199.0655	88.95739	
Botswana	DALYs	Male	2021	150.2711	217.9945	100.8852	
Botswana	DALYs	Male	2022	156.3169	230.3833	105.8918	
Botswana	DALYs	Male	2023	168.1232	247.5185	114.8029	
Botswana	DALYs	Female	2010	29.47624	41.77052	17.92185	
Botswana	DALYs	Female	2011	29.62592	41.34491	17.519	
Botswana	DALYs	Female	2012	30.25717	42.81357	17.79654	

Botswana	DALYs	Female	2013	30.76806	43.94588	18.15488	
Botswana	DALYs	Female	2014	31.88754	44.76731	18.55441	
Botswana	DALYs	Female	2015	33.87929	47.92977	19.85065	
Botswana	DALYs	Female	2016	36.34644	51.61138	21.48428	
Botswana	DALYs	Female	2017	38.56105	54.22362	23.20433	
Botswana	DALYs	Female	2018	41.2188	57.70193	25.83259	
Botswana	DALYs	Female	2019	43.75346	61.9132	28.37966	
Botswana	DALYs	Female	2020	46.24994	64.93841	29.75602	
Botswana	DALYs	Female	2021	55.52123	74.98838	36.79248	
Botswana	DALYs	Female	2022	54.84221	76.25117	34.81376	
Botswana	DALYs	Female	2023	57.57297	81.87012	36.71748	
Brazil	Incidence	Male	2010	18.11822	19.19481	17.18683	
Brazil	Incidence	Male	2011	18.41182	19.57433	17.56751	
Brazil	Incidence	Male	2012	18.52281	19.5477	17.56874	
Brazil	Incidence	Male	2013	18.90832	19.86304	17.89708	
Brazil	Incidence	Male	2014	19.14501	20.12137	18.15795	
Brazil	Incidence	Male	2015	19.48498	20.4402	18.48359	
Brazil	Incidence	Male	2016	19.94966	20.85076	18.93395	
Brazil	Incidence	Male	2017	19.95847	20.97222	18.7968	
Brazil	Incidence	Male	2018	20.09684	21.20362	18.81563	
Brazil	Incidence	Male	2019	20.41559	21.60915	19.16918	
Brazil	Incidence	Male	2020	20.15877	21.19997	19.07236	
Brazil	Incidence	Male	2021	20.24884	21.37944	19.15384	
Brazil	Incidence	Male	2022	20.83303	22.05585	19.67933	
Brazil	Incidence	Male	2023	21.16486	22.4358	19.92612	
Brazil	Incidence	Female	2010	10.46359	11.15443	9.642071	
Brazil	Incidence	Female	2011	11.00908	11.68793	10.13769	
Brazil	Incidence	Female	2012	11.39382	12.13946	10.49698	
Brazil	Incidence	Female	2013	11.80644	12.55516	10.8243	
Brazil	Incidence	Female	2014	12.27981	13.07627	11.09543	
Brazil	Incidence	Female	2015	12.961	13.80817	11.68645	
Brazil	Incidence	Female	2016	13.34665	14.14703	12.22777	
Brazil	Incidence	Female	2017	13.61892	14.50909	12.46314	
Brazil	Incidence	Female	2018	13.97649	14.95669	12.82388	
Brazil	Incidence	Female	2019	14.30869	15.50387	12.88777	
Brazil	Incidence	Female	2020	14.22426	15.37231	13.00883	

Brazil	Incidence	Female	2021	14.77141	16.0007	13.53209	
Brazil	Incidence	Female	2022	15.61034	16.91626	14.10681	
Brazil	Incidence	Female	2023	16.08324	17.44851	14.51914	
Brazil	Deaths	Male	2010	18.07977	18.94432	17.0249	
Brazil	Deaths	Male	2011	18.40564	19.45397	17.49553	
Brazil	Deaths	Male	2012	18.52234	19.46269	17.46354	
Brazil	Deaths	Male	2013	18.92899	19.80052	17.86182	
Brazil	Deaths	Male	2014	19.17662	20.12723	18.18435	
Brazil	Deaths	Male	2015	19.55989	20.47546	18.5914	
Brazil	Deaths	Male	2016	20.05759	20.98025	19.0251	
Brazil	Deaths	Male	2017	20.07864	21.05025	18.95559	
Brazil	Deaths	Male	2018	20.22126	21.20382	19.06422	
Brazil	Deaths	Male	2019	20.57711	21.6026	19.41383	
Brazil	Deaths	Male	2020	20.30147	21.2745	19.24762	
Brazil	Deaths	Male	2021	20.36266	21.39238	19.31443	
Brazil	Deaths	Male	2022	21.02024	22.12159	19.90283	
Brazil	Deaths	Male	2023	21.30175	22.47993	20.05891	
Brazil	Deaths	Female	2010	10.30876	10.92648	9.391745	
Brazil	Deaths	Female	2011	10.82767	11.49879	9.921603	
Brazil	Deaths	Female	2012	11.21396	11.89607	10.234	
Brazil	Deaths	Female	2013	11.62541	12.33628	10.5095	
Brazil	Deaths	Female	2014	12.10866	12.86314	10.82227	
Brazil	Deaths	Female	2015	12.79917	13.63891	11.47869	
Brazil	Deaths	Female	2016	13.18996	14.12637	11.96979	
Brazil	Deaths	Female	2017	13.46973	14.40158	12.21075	
Brazil	Deaths	Female	2018	13.82169	14.79007	12.6296	
Brazil	Deaths	Female	2019	14.16366	15.18764	12.82533	
Brazil	Deaths	Female	2020	14.08749	15.14563	12.87315	
Brazil	Deaths	Female	2021	14.61559	15.73332	13.37981	
Brazil	Deaths	Female	2022	15.49959	16.69534	14.02594	
Brazil	Deaths	Female	2023	15.91664	17.2128	14.25851	
Brazil	DALYs	Male	2010	456.0439	480.3693	433.1615	
Brazil	DALYs	Male	2011	461.0123	488.3678	438.9574	
Brazil	DALYs	Male	2012	462.2679	487.4313	441.1225	
Brazil	DALYs	Male	2013	469.4482	493.6966	447.5136	
Brazil	DALYs	Male	2014	473.7828	497.5057	453.9459	

Brazil	DALYs	Male	2015	478.7614	502.6488	457.7475	
Brazil	DALYs	Male	2016	487.0132	510.8151	463.4807	
Brazil	DALYs	Male	2017	484.3239	508.7638	461.9758	
Brazil	DALYs	Male	2018	484.3994	510.5336	457.6481	
Brazil	DALYs	Male	2019	487.7945	514.1868	463.1802	
Brazil	DALYs	Male	2020	481.0321	507.0158	457.4973	
Brazil	DALYs	Male	2021	482.6756	508.7253	457.3159	
Brazil	DALYs	Male	2022	491.1097	517.9236	465.1867	
Brazil	DALYs	Male	2023	498.5378	528.2257	472.0946	
Brazil	DALYs	Female	2010	265.0003	280.6961	246.7496	
Brazil	DALYs	Female	2011	277.9503	294.1559	259.2735	
Brazil	DALYs	Female	2012	286.35	302.096	266.7943	
Brazil	DALYs	Female	2013	295.3466	311.9334	274.8827	
Brazil	DALYs	Female	2014	305.3033	322.2155	281.7612	
Brazil	DALYs	Female	2015	319.7775	336.7333	292.65	
Brazil	DALYs	Female	2016	327.0975	346.2937	304.0952	
Brazil	DALYs	Female	2017	331.0422	351.4574	307.6047	
Brazil	DALYs	Female	2018	337.1674	359.0612	313.5165	
Brazil	DALYs	Female	2019	341.9243	363.9868	314.1426	
Brazil	DALYs	Female	2020	337.8404	362.8054	312.7269	
Brazil	DALYs	Female	2021	348.9833	373.2229	325.62	
Brazil	DALYs	Female	2022	364.4019	391.597	338.7851	
Brazil	DALYs	Female	2023	374.1289	403.2459	343.1384	
Brunei Darussa	Incidence	Male	2010	15.84348	19.48286	13.09345	
Brunei Darussa	Incidence	Male	2011	16.3172	20.41445	13.51275	
Brunei Darussa	Incidence	Male	2012	16.85493	20.92103	13.7755	
Brunei Darussa	Incidence	Male	2013	17.19314	21.02359	14.22939	
Brunei Darussa	Incidence	Male	2014	17.57133	21.42235	14.1045	
Brunei Darussa	Incidence	Male	2015	17.3557	21.68081	13.57165	
Brunei Darussa	Incidence	Male	2016	17.7471	22.40352	13.85415	
Brunei Darussa	Incidence	Male	2017	18.20511	23.28791	14.30486	
Brunei Darussa	Incidence	Male	2018	18.80636	24.61518	14.88559	
Brunei Darussa	Incidence	Male	2019	19.79642	26.36503	14.89405	
Brunei Darussa	Incidence	Male	2020	20.88726	27.91568	15.96096	
Brunei Darussa	Incidence	Male	2021	21.862	28.89654	16.63793	
Brunei Darussa	Incidence	Male	2022	22.20905	29.30212	16.77915	

Brunei Darussa	Incidence	Male	2023	23.37396	32.18426	17.45048	
Brunei Darussa	Incidence	Female	2010	13.10247	17.32029	9.963013	
Brunei Darussa	Incidence	Female	2011	13.64695	17.88163	10.50886	
Brunei Darussa	Incidence	Female	2012	14.22901	18.69936	10.82516	
Brunei Darussa	Incidence	Female	2013	14.87778	19.41965	11.19878	
Brunei Darussa	Incidence	Female	2014	15.16526	19.53562	11.40553	
Brunei Darussa	Incidence	Female	2015	15.40171	20.02116	11.44562	
Brunei Darussa	Incidence	Female	2016	15.84246	20.34434	11.84375	
Brunei Darussa	Incidence	Female	2017	16.3306	20.76089	12.31191	
Brunei Darussa	Incidence	Female	2018	16.99389	21.09801	12.85573	
Brunei Darussa	Incidence	Female	2019	18.18219	22.46732	13.90133	
Brunei Darussa	Incidence	Female	2020	19.27541	23.88581	14.70387	
Brunei Darussa	Incidence	Female	2021	20.42237	25.31219	15.35894	
Brunei Darussa	Incidence	Female	2022	21.19629	26.11013	15.74121	
Brunei Darussa	Incidence	Female	2023	22.07115	28.37101	16.18939	
Brunei Darussa	Deaths	Male	2010	15.3389	18.93084	12.68199	
Brunei Darussa	Deaths	Male	2011	15.79787	19.66173	13.00358	
Brunei Darussa	Deaths	Male	2012	16.29784	20.14801	13.26772	
Brunei Darussa	Deaths	Male	2013	16.59503	20.2893	13.52222	
Brunei Darussa	Deaths	Male	2014	16.96253	20.41621	13.56466	
Brunei Darussa	Deaths	Male	2015	16.78156	20.55327	13.12301	
Brunei Darussa	Deaths	Male	2016	17.13947	21.59636	13.37397	
Brunei Darussa	Deaths	Male	2017	17.57656	22.34977	13.80614	
Brunei Darussa	Deaths	Male	2018	18.18237	23.51452	14.19368	
Brunei Darussa	Deaths	Male	2019	19.1014	25.3848	14.46975	
Brunei Darussa	Deaths	Male	2020	20.10187	26.93125	15.30166	
Brunei Darussa	Deaths	Male	2021	20.94495	27.94547	15.95979	
Brunei Darussa	Deaths	Male	2022	21.17451	28.15492	16.01655	
Brunei Darussa	Deaths	Male	2023	22.25699	30.33155	16.8058	
Brunei Darussa	Deaths	Female	2010	12.2602	16.0419	9.33322	
Brunei Darussa	Deaths	Female	2011	12.75956	16.78078	9.884955	
Brunei Darussa	Deaths	Female	2012	13.28837	17.41482	10.22793	
Brunei Darussa	Deaths	Female	2013	13.89057	18.01659	10.54078	
Brunei Darussa	Deaths	Female	2014	14.18283	18.09579	10.70295	
Brunei Darussa	Deaths	Female	2015	14.43487	18.53017	10.85017	
Brunei Darussa	Deaths	Female	2016	14.82023	18.98152	11.25379	

Brunei Darussa	Deaths	Female	2017	15.2861	19.39497	11.49719	
Brunei Darussa	Deaths	Female	2018	15.92801	19.8494	12.11049	
Brunei Darussa	Deaths	Female	2019	17.01819	21.16194	13.1062	
Brunei Darussa	Deaths	Female	2020	17.99509	22.17425	13.98197	
Brunei Darussa	Deaths	Female	2021	18.96549	23.28322	14.45693	
Brunei Darussa	Deaths	Female	2022	19.63327	24.22637	14.86587	
Brunei Darussa	Deaths	Female	2023	20.46989	26.32889	15.19529	
Brunei Darussa	DALYs	Male	2010	393.4674	493.3624	325.1485	
Brunei Darussa	DALYs	Male	2011	403.6976	507.6663	333.6361	
Brunei Darussa	DALYs	Male	2012	415.9398	518.9167	338.3863	
Brunei Darussa	DALYs	Male	2013	425.243	521.3839	346.3675	
Brunei Darussa	DALYs	Male	2014	434.9108	531.4708	348.4456	
Brunei Darussa	DALYs	Male	2015	429.7399	530.5166	335.5927	
Brunei Darussa	DALYs	Male	2016	439.5688	551.2254	344.1412	
Brunei Darussa	DALYs	Male	2017	449.1246	573.235	352.7179	
Brunei Darussa	DALYs	Male	2018	460.3868	599.7323	362.6657	
Brunei Darussa	DALYs	Male	2019	481.9397	640.4505	366.703	
Brunei Darussa	DALYs	Male	2020	505.1213	677.3734	386.5353	
Brunei Darussa	DALYs	Male	2021	527.8934	701.1931	401.8437	
Brunei Darussa	DALYs	Male	2022	539.3321	716.3457	407.5181	
Brunei Darussa	DALYs	Male	2023	568.4498	776.6971	425.4252	
Brunei Darussa	DALYs	Female	2010	329.3227	434.9153	251.7947	
Brunei Darussa	DALYs	Female	2011	341.5154	452.9791	264.029	
Brunei Darussa	DALYs	Female	2012	354.633	467.8172	269.0235	
Brunei Darussa	DALYs	Female	2013	370.3417	483.5324	276.5776	
Brunei Darussa	DALYs	Female	2014	376.6594	481.854	279.1594	
Brunei Darussa	DALYs	Female	2015	382.4271	488.1917	282.6901	
Brunei Darussa	DALYs	Female	2016	393.8677	503.3731	293.6038	
Brunei Darussa	DALYs	Female	2017	403.2824	509.6132	303.6546	
Brunei Darussa	DALYs	Female	2018	415.3469	516.2162	313.8998	
Brunei Darussa	DALYs	Female	2019	439.4149	542.9819	333.7312	
Brunei Darussa	DALYs	Female	2020	460.9385	566.6408	360.0695	
Brunei Darussa	DALYs	Female	2021	485.7796	598.0214	370.6951	
Brunei Darussa	DALYs	Female	2022	503.6063	624.1892	377.8944	
Brunei Darussa	DALYs	Female	2023	522.2388	675.8611	388.248	
Bulgaria	Incidence	Male	2010	90.66964	96.52906	85.53149	

Bulgaria	Incidence	Male	2011	94.5365	101.2938	89.12987	
Bulgaria	Incidence	Male	2012	93.94214	100.156	89.09059	
Bulgaria	Incidence	Male	2013	93.53609	99.08136	88.50877	
Bulgaria	Incidence	Male	2014	92.51146	97.86311	87.15186	
Bulgaria	Incidence	Male	2015	92.11871	98.26916	86.83541	
Bulgaria	Incidence	Male	2016	91.30317	97.35896	85.80966	
Bulgaria	Incidence	Male	2017	91.19751	97.45072	85.56809	
Bulgaria	Incidence	Male	2018	89.89523	96.73138	84.64216	
Bulgaria	Incidence	Male	2019	89.39982	96.55585	83.76978	
Bulgaria	Incidence	Male	2020	88.38771	95.84896	83.02277	
Bulgaria	Incidence	Male	2021	87.91712	95.83263	82.15143	
Bulgaria	Incidence	Male	2022	84.24903	92.55723	77.53681	
Bulgaria	Incidence	Male	2023	82.30805	90.3753	75.1933	
Bulgaria	Incidence	Female	2010	20.84333	22.78374	19.2804	
Bulgaria	Incidence	Female	2011	22.36079	24.56153	20.60263	
Bulgaria	Incidence	Female	2012	23.40983	25.54752	21.42895	
Bulgaria	Incidence	Female	2013	23.51847	25.77201	21.32305	
Bulgaria	Incidence	Female	2014	24.55959	26.87814	22.20962	
Bulgaria	Incidence	Female	2015	25.62345	27.92057	23.29478	
Bulgaria	Incidence	Female	2016	25.75851	28.09539	23.59466	
Bulgaria	Incidence	Female	2017	26.82351	29.29444	24.45307	
Bulgaria	Incidence	Female	2018	27.99283	31.18937	25.2555	
Bulgaria	Incidence	Female	2019	29.00247	32.53003	26.24631	
Bulgaria	Incidence	Female	2020	30.06594	33.52882	27.10594	
Bulgaria	Incidence	Female	2021	30.61726	34.30311	27.68581	
Bulgaria	Incidence	Female	2022	29.51549	33.27561	26.4351	
Bulgaria	Incidence	Female	2023	28.33863	32.18396	25.17542	
Bulgaria	Deaths	Male	2010	88.78284	94.27862	83.95009	
Bulgaria	Deaths	Male	2011	92.60822	98.51678	87.50763	
Bulgaria	Deaths	Male	2012	92.26474	98.04962	87.55251	
Bulgaria	Deaths	Male	2013	91.89709	97.48695	87.07373	
Bulgaria	Deaths	Male	2014	90.95022	96.13369	85.97735	
Bulgaria	Deaths	Male	2015	90.50522	96.34172	84.99381	
Bulgaria	Deaths	Male	2016	89.93929	95.57493	84.83976	
Bulgaria	Deaths	Male	2017	90.06435	95.94072	84.77289	
Bulgaria	Deaths	Male	2018	89.00194	95.61748	83.85429	

Bulgaria	Deaths	Male	2019	88.75653	95.0753	83.09748	
Bulgaria	Deaths	Male	2020	87.98192	94.78874	82.86029	
Bulgaria	Deaths	Male	2021	87.33141	94.87462	81.8496	
Bulgaria	Deaths	Male	2022	83.60504	91.77579	77.34389	
Bulgaria	Deaths	Male	2023	81.53361	89.09007	74.85018	
Bulgaria	Deaths	Female	2010	19.76732	21.67146	18.26599	
Bulgaria	Deaths	Female	2011	21.1235	23.33147	19.52786	
Bulgaria	Deaths	Female	2012	22.08072	24.12096	20.38538	
Bulgaria	Deaths	Female	2013	22.14173	23.88949	20.18068	
Bulgaria	Deaths	Female	2014	23.09502	25.04769	21.07122	
Bulgaria	Deaths	Female	2015	24.04289	25.99152	21.99465	
Bulgaria	Deaths	Female	2016	24.1606	26.17915	22.12227	
Bulgaria	Deaths	Female	2017	25.22175	27.36145	23.08941	
Bulgaria	Deaths	Female	2018	26.32616	28.92712	24.00346	
Bulgaria	Deaths	Female	2019	27.26298	30.17365	24.8238	
Bulgaria	Deaths	Female	2020	28.25953	31.3501	25.75821	
Bulgaria	Deaths	Female	2021	28.73298	32.05649	26.05627	
Bulgaria	Deaths	Female	2022	27.66347	31.07976	25.1219	
Bulgaria	Deaths	Female	2023	26.50019	29.58601	23.77964	
Bulgaria	DALYs	Male	2010	2383.264	2533.955	2249.987	
Bulgaria	DALYs	Male	2011	2474.64	2642.15	2324.562	
Bulgaria	DALYs	Male	2012	2441.481	2605.898	2308.241	
Bulgaria	DALYs	Male	2013	2418.1	2564.21	2284.139	
Bulgaria	DALYs	Male	2014	2379.996	2521.477	2239.739	
Bulgaria	DALYs	Male	2015	2369.094	2521.323	2219.277	
Bulgaria	DALYs	Male	2016	2328.208	2480.96	2188.906	
Bulgaria	DALYs	Male	2017	2311.303	2465.249	2169.791	
Bulgaria	DALYs	Male	2018	2263.088	2435.439	2127.287	
Bulgaria	DALYs	Male	2019	2230.517	2407.194	2079.087	
Bulgaria	DALYs	Male	2020	2186.414	2372.46	2050.143	
Bulgaria	DALYs	Male	2021	2180.183	2389.168	2037.266	
Bulgaria	DALYs	Male	2022	2088.607	2315.799	1915.158	
Bulgaria	DALYs	Male	2023	2039.474	2246.665	1865.105	
Bulgaria	DALYs	Female	2010	497.2727	545.5743	460.9207	
Bulgaria	DALYs	Female	2011	531.9418	587.3135	493.7315	
Bulgaria	DALYs	Female	2012	552.8116	601.6188	512.8152	

Bulgaria	DALYs	Female	2013	552.8596	598.864	508.2674	
Bulgaria	DALYs	Female	2014	574.9794	623.8386	523.5079	
Bulgaria	DALYs	Female	2015	600.0987	649.1116	549.9733	
Bulgaria	DALYs	Female	2016	603.5104	655.024	549.8531	
Bulgaria	DALYs	Female	2017	623.8199	678.6628	565.2055	
Bulgaria	DALYs	Female	2018	650.4878	712.4496	592.5092	
Bulgaria	DALYs	Female	2019	673.1275	744.1209	610.1332	
Bulgaria	DALYs	Female	2020	691.6144	766.8386	626.66	
Bulgaria	DALYs	Female	2021	699.5568	779.7091	632.7374	
Bulgaria	DALYs	Female	2022	671.7942	758.0072	610.5368	
Bulgaria	DALYs	Female	2023	641.2911	718.0993	574.5497	
Burkina Faso	Incidence	Male	2010	2.354891	3.38842	1.5762	
Burkina Faso	Incidence	Male	2011	2.259486	3.167624	1.527618	
Burkina Faso	Incidence	Male	2012	2.280048	3.229087	1.566431	
Burkina Faso	Incidence	Male	2013	2.209916	3.058054	1.558441	
Burkina Faso	Incidence	Male	2014	2.210299	3.069863	1.554343	
Burkina Faso	Incidence	Male	2015	2.443447	3.464647	1.702626	
Burkina Faso	Incidence	Male	2016	2.410133	3.37904	1.623779	
Burkina Faso	Incidence	Male	2017	2.389031	3.357183	1.599051	
Burkina Faso	Incidence	Male	2018	2.360893	3.394082	1.577645	
Burkina Faso	Incidence	Male	2019	2.323043	3.441964	1.540062	
Burkina Faso	Incidence	Male	2020	2.321855	3.519368	1.567274	
Burkina Faso	Incidence	Male	2021	2.289012	3.435555	1.53631	
Burkina Faso	Incidence	Male	2022	2.667938	3.947072	1.808423	
Burkina Faso	Incidence	Male	2023	2.897863	4.21312	1.950505	
Burkina Faso	Incidence	Female	2010	0.868648	1.334132	0.589457	
Burkina Faso	Incidence	Female	2011	0.877759	1.367276	0.591383	
Burkina Faso	Incidence	Female	2012	0.896926	1.380753	0.611189	
Burkina Faso	Incidence	Female	2013	0.898729	1.351361	0.593478	
Burkina Faso	Incidence	Female	2014	0.890973	1.295149	0.578546	
Burkina Faso	Incidence	Female	2015	0.949652	1.346047	0.605677	
Burkina Faso	Incidence	Female	2016	0.955707	1.363517	0.614747	
Burkina Faso	Incidence	Female	2017	0.963981	1.344836	0.625122	
Burkina Faso	Incidence	Female	2018	0.957454	1.304587	0.647385	
Burkina Faso	Incidence	Female	2019	0.951616	1.283293	0.6466	
Burkina Faso	Incidence	Female	2020	0.916697	1.224494	0.636059	

Burkina Faso	Incidence	Female	2021	0.904702	1.229432	0.601765	
Burkina Faso	Incidence	Female	2022	1.126023	1.568626	0.743576	
Burkina Faso	Incidence	Female	2023	1.212137	1.67872	0.829669	
Burkina Faso	Deaths	Male	2010	2.405586	3.458745	1.612139	
Burkina Faso	Deaths	Male	2011	2.313743	3.259946	1.563107	
Burkina Faso	Deaths	Male	2012	2.340898	3.318563	1.603078	
Burkina Faso	Deaths	Male	2013	2.274111	3.143345	1.600202	
Burkina Faso	Deaths	Male	2014	2.274145	3.15991	1.587452	
Burkina Faso	Deaths	Male	2015	2.519598	3.588004	1.752212	
Burkina Faso	Deaths	Male	2016	2.481835	3.467754	1.669432	
Burkina Faso	Deaths	Male	2017	2.460979	3.45771	1.650547	
Burkina Faso	Deaths	Male	2018	2.432828	3.495595	1.626066	
Burkina Faso	Deaths	Male	2019	2.393189	3.535377	1.588989	
Burkina Faso	Deaths	Male	2020	2.395524	3.643663	1.619368	
Burkina Faso	Deaths	Male	2021	2.358692	3.555969	1.570392	
Burkina Faso	Deaths	Male	2022	2.743606	4.056066	1.854986	
Burkina Faso	Deaths	Male	2023	2.970328	4.340916	2.002812	
Burkina Faso	Deaths	Female	2010	0.881247	1.358203	0.595752	
Burkina Faso	Deaths	Female	2011	0.889139	1.391696	0.592538	
Burkina Faso	Deaths	Female	2012	0.909019	1.410629	0.620492	
Burkina Faso	Deaths	Female	2013	0.91332	1.388496	0.603313	
Burkina Faso	Deaths	Female	2014	0.905211	1.31719	0.585976	
Burkina Faso	Deaths	Female	2015	0.967671	1.385421	0.614932	
Burkina Faso	Deaths	Female	2016	0.971807	1.389316	0.623932	
Burkina Faso	Deaths	Female	2017	0.98084	1.370147	0.636075	
Burkina Faso	Deaths	Female	2018	0.974633	1.326966	0.659285	
Burkina Faso	Deaths	Female	2019	0.968878	1.304004	0.654759	
Burkina Faso	Deaths	Female	2020	0.933069	1.247603	0.645033	
Burkina Faso	Deaths	Female	2021	0.919157	1.254792	0.605853	
Burkina Faso	Deaths	Female	2022	1.140025	1.584473	0.7395	
Burkina Faso	Deaths	Female	2023	1.22399	1.705213	0.828766	
Burkina Faso	DALYs	Male	2010	62.22842	89.28548	41.76657	
Burkina Faso	DALYs	Male	2011	59.24153	82.67835	39.56494	
Burkina Faso	DALYs	Male	2012	59.44612	84.22987	41.40231	
Burkina Faso	DALYs	Male	2013	57.3236	79.54728	40.482	
Burkina Faso	DALYs	Male	2014	57.37194	79.76418	40.65019	

Burkina Faso	DALYs	Male	2015	63.17293	88.4488	43.96431	
Burkina Faso	DALYs	Male	2016	62.3683	87.92499	41.99527	
Burkina Faso	DALYs	Male	2017	61.78908	87.22877	41.14092	
Burkina Faso	DALYs	Male	2018	61.07116	87.98615	40.39723	
Burkina Faso	DALYs	Male	2019	60.16075	90.15527	39.38925	
Burkina Faso	DALYs	Male	2020	60.17108	90.85167	40.50516	
Burkina Faso	DALYs	Male	2021	59.34831	88.48002	39.66301	
Burkina Faso	DALYs	Male	2022	69.15645	101.8367	46.62173	
Burkina Faso	DALYs	Male	2023	75.60442	109.4739	50.77219	
Burkina Faso	DALYs	Female	2010	23.90224	36.35889	16.21045	
Burkina Faso	DALYs	Female	2011	24.14252	37.57537	16.35861	
Burkina Faso	DALYs	Female	2012	24.66644	37.28092	16.7068	
Burkina Faso	DALYs	Female	2013	24.56547	36.86258	16.17801	
Burkina Faso	DALYs	Female	2014	24.37465	35.47751	15.78431	
Burkina Faso	DALYs	Female	2015	25.82966	36.35245	16.59596	
Burkina Faso	DALYs	Female	2016	26.02985	37.00035	16.76494	
Burkina Faso	DALYs	Female	2017	26.22675	36.36944	17.10218	
Burkina Faso	DALYs	Female	2018	26.02586	35.14846	17.39774	
Burkina Faso	DALYs	Female	2019	25.85872	35.16179	17.78049	
Burkina Faso	DALYs	Female	2020	24.92598	33.04367	17.3429	
Burkina Faso	DALYs	Female	2021	24.65221	33.45003	16.84977	
Burkina Faso	DALYs	Female	2022	30.74486	43.09619	20.94441	
Burkina Faso	DALYs	Female	2023	33.34098	45.45678	22.7177	
Burundi	Incidence	Male	2010	2.278498	3.488403	1.280406	
Burundi	Incidence	Male	2011	2.305455	3.556271	1.289483	
Burundi	Incidence	Male	2012	2.369516	3.694318	1.326355	
Burundi	Incidence	Male	2013	2.407912	3.774284	1.357493	
Burundi	Incidence	Male	2014	2.481757	3.811634	1.389555	
Burundi	Incidence	Male	2015	2.538128	3.904665	1.404627	
Burundi	Incidence	Male	2016	2.607279	3.999435	1.435664	
Burundi	Incidence	Male	2017	2.675357	4.146404	1.466133	
Burundi	Incidence	Male	2018	2.707239	4.208857	1.460109	
Burundi	Incidence	Male	2019	2.754812	4.362686	1.46023	
Burundi	Incidence	Male	2020	2.85122	4.61529	1.514155	
Burundi	Incidence	Male	2021	3.245438	5.227239	1.750996	
Burundi	Incidence	Male	2022	3.015871	4.880844	1.550025	

Burundi	Incidence	Male	2023	3.153635	4.927058	1.713483	
Burundi	Incidence	Female	2010	0.781388	1.286296	0.459297	
Burundi	Incidence	Female	2011	0.799309	1.305003	0.475174	
Burundi	Incidence	Female	2012	0.827458	1.33796	0.501324	
Burundi	Incidence	Female	2013	0.835175	1.334492	0.50304	
Burundi	Incidence	Female	2014	0.851115	1.34582	0.508751	
Burundi	Incidence	Female	2015	0.876714	1.349727	0.529478	
Burundi	Incidence	Female	2016	0.904529	1.391143	0.547941	
Burundi	Incidence	Female	2017	0.942498	1.43541	0.576866	
Burundi	Incidence	Female	2018	0.964279	1.417738	0.587759	
Burundi	Incidence	Female	2019	0.996804	1.441144	0.602174	
Burundi	Incidence	Female	2020	1.000068	1.489305	0.614865	
Burundi	Incidence	Female	2021	1.120756	1.761625	0.688871	
Burundi	Incidence	Female	2022	1.151896	1.70234	0.695889	
Burundi	Incidence	Female	2023	1.240828	1.845812	0.74679	
Burundi	Deaths	Male	2010	2.205417	3.361675	1.236774	
Burundi	Deaths	Male	2011	2.226077	3.434182	1.239936	
Burundi	Deaths	Male	2012	2.285382	3.544595	1.27361	
Burundi	Deaths	Male	2013	2.31951	3.625781	1.305798	
Burundi	Deaths	Male	2014	2.384928	3.660185	1.326897	
Burundi	Deaths	Male	2015	2.437164	3.749214	1.347186	
Burundi	Deaths	Male	2016	2.499939	3.850718	1.37398	
Burundi	Deaths	Male	2017	2.562218	3.981083	1.397869	
Burundi	Deaths	Male	2018	2.589874	4.021039	1.396158	
Burundi	Deaths	Male	2019	2.63202	4.171035	1.396798	
Burundi	Deaths	Male	2020	2.730702	4.427106	1.449969	
Burundi	Deaths	Male	2021	3.108613	4.992796	1.668452	
Burundi	Deaths	Male	2022	2.879231	4.65878	1.476701	
Burundi	Deaths	Male	2023	3.006111	4.73592	1.63364	
Burundi	Deaths	Female	2010	0.756058	1.241236	0.44411	
Burundi	Deaths	Female	2011	0.770751	1.257838	0.457491	
Burundi	Deaths	Female	2012	0.795363	1.28481	0.481397	
Burundi	Deaths	Female	2013	0.799939	1.274635	0.481951	
Burundi	Deaths	Female	2014	0.812368	1.273645	0.487701	
Burundi	Deaths	Female	2015	0.834764	1.292373	0.503434	
Burundi	Deaths	Female	2016	0.858034	1.3111	0.518242	

Burundi	Deaths	Female	2017	0.891011	1.350824	0.543611	
Burundi	Deaths	Female	2018	0.908744	1.332692	0.550934	
Burundi	Deaths	Female	2019	0.936593	1.35356	0.563278	
Burundi	Deaths	Female	2020	0.938493	1.396731	0.573584	
Burundi	Deaths	Female	2021	1.049894	1.645754	0.640635	
Burundi	Deaths	Female	2022	1.074399	1.579317	0.644644	
Burundi	Deaths	Female	2023	1.153548	1.704305	0.69237	
Burundi	DALYs	Male	2010	68.84723	106.9253	38.42883	
Burundi	DALYs	Male	2011	69.97911	108.8516	39.53151	
Burundi	DALYs	Male	2012	71.98142	114.3318	40.06017	
Burundi	DALYs	Male	2013	73.20626	114.6146	40.92167	
Burundi	DALYs	Male	2014	75.73557	116.6248	42.67311	
Burundi	DALYs	Male	2015	77.64031	119.559	43.01301	
Burundi	DALYs	Male	2016	79.98516	122.7867	44.07484	
Burundi	DALYs	Male	2017	82.2346	127.5381	45.48759	
Burundi	DALYs	Male	2018	83.40192	130.0637	45.05906	
Burundi	DALYs	Male	2019	85.07742	134.5095	45.32283	
Burundi	DALYs	Male	2020	87.73105	141.475	46.59655	
Burundi	DALYs	Male	2021	99.67711	159.0166	52.76344	
Burundi	DALYs	Male	2022	93.34567	151.3586	48.01022	
Burundi	DALYs	Male	2023	97.88424	151.8755	53.14504	
Burundi	DALYs	Female	2010	24.25729	39.95114	14.45846	
Burundi	DALYs	Female	2011	24.93151	40.77404	14.959	
Burundi	DALYs	Female	2012	25.89801	41.90094	15.79433	
Burundi	DALYs	Female	2013	26.2649	41.8106	15.72782	
Burundi	DALYs	Female	2014	26.9014	42.55782	16.13563	
Burundi	DALYs	Female	2015	27.84172	42.48978	16.88891	
Burundi	DALYs	Female	2016	28.92326	44.80608	17.75893	
Burundi	DALYs	Female	2017	30.31338	46.17539	18.78351	
Burundi	DALYs	Female	2018	31.18443	45.65959	19.16648	
Burundi	DALYs	Female	2019	32.40892	46.55794	19.76066	
Burundi	DALYs	Female	2020	32.62294	48.24233	20.44202	
Burundi	DALYs	Female	2021	36.59609	57.08916	22.64519	
Burundi	DALYs	Female	2022	38.02379	55.84477	23.49479	
Burundi	DALYs	Female	2023	41.28219	60.86971	24.95602	
Côte d'Ivoire	Incidence	Male	2010	1.302057	1.812729	0.996991	

Côte d'Ivoire	Incidence	Male	2011	1.352326	1.880453	1.059174	
Côte d'Ivoire	Incidence	Male	2012	1.467887	2.004164	1.122004	
Côte d'Ivoire	Incidence	Male	2013	1.500192	2.009171	1.116268	
Côte d'Ivoire	Incidence	Male	2014	1.534224	2.00784	1.145321	
Côte d'Ivoire	Incidence	Male	2015	1.70667	2.270129	1.260983	
Côte d'Ivoire	Incidence	Male	2016	1.717946	2.352498	1.22703	
Côte d'Ivoire	Incidence	Male	2017	1.717714	2.3561	1.219561	
Côte d'Ivoire	Incidence	Male	2018	1.753305	2.474223	1.216288	
Côte d'Ivoire	Incidence	Male	2019	1.73451	2.513377	1.217179	
Côte d'Ivoire	Incidence	Male	2020	1.816544	2.60858	1.277248	
Côte d'Ivoire	Incidence	Male	2021	1.808272	2.506768	1.27111	
Côte d'Ivoire	Incidence	Male	2022	2.054884	2.837686	1.403083	
Côte d'Ivoire	Incidence	Male	2023	2.177329	3.054524	1.506151	
Côte d'Ivoire	Incidence	Female	2010	0.834278	1.183697	0.59011	
Côte d'Ivoire	Incidence	Female	2011	0.857631	1.19185	0.595175	
Côte d'Ivoire	Incidence	Female	2012	0.918387	1.256025	0.638478	
Côte d'Ivoire	Incidence	Female	2013	0.977364	1.337891	0.664906	
Côte d'Ivoire	Incidence	Female	2014	1.010066	1.388372	0.672985	
Côte d'Ivoire	Incidence	Female	2015	1.113768	1.51435	0.723791	
Côte d'Ivoire	Incidence	Female	2016	1.171605	1.60038	0.77431	
Côte d'Ivoire	Incidence	Female	2017	1.208392	1.639523	0.823333	
Côte d'Ivoire	Incidence	Female	2018	1.243126	1.678309	0.845535	
Côte d'Ivoire	Incidence	Female	2019	1.225051	1.652853	0.836461	
Côte d'Ivoire	Incidence	Female	2020	1.256765	1.690084	0.872588	
Côte d'Ivoire	Incidence	Female	2021	1.251661	1.627701	0.880793	
Côte d'Ivoire	Incidence	Female	2022	1.479324	1.985241	1.003687	
Côte d'Ivoire	Incidence	Female	2023	1.59316	2.14586	1.063391	
Côte d'Ivoire	Deaths	Male	2010	1.282193	1.776077	0.985448	
Côte d'Ivoire	Deaths	Male	2011	1.330828	1.849626	1.038183	
Côte d'Ivoire	Deaths	Male	2012	1.439624	1.966197	1.10129	
Côte d'Ivoire	Deaths	Male	2013	1.468689	1.974149	1.094138	
Côte d'Ivoire	Deaths	Male	2014	1.498123	1.96767	1.11134	
Côte d'Ivoire	Deaths	Male	2015	1.663422	2.213791	1.234439	
Côte d'Ivoire	Deaths	Male	2016	1.669466	2.277169	1.194164	
Côte d'Ivoire	Deaths	Male	2017	1.665335	2.293095	1.176313	
Côte d'Ivoire	Deaths	Male	2018	1.694792	2.384194	1.176854	

Côte d'Ivoire	Deaths	Male	2019	1.673405	2.421288	1.172348	
Côte d'Ivoire	Deaths	Male	2020	1.750949	2.504243	1.227885	
Côte d'Ivoire	Deaths	Male	2021	1.738835	2.399584	1.219531	
Côte d'Ivoire	Deaths	Male	2022	1.97616	2.716352	1.3456	
Côte d'Ivoire	Deaths	Male	2023	2.088669	2.926475	1.453747	
Côte d'Ivoire	Deaths	Female	2010	0.800609	1.140421	0.563491	
Côte d'Ivoire	Deaths	Female	2011	0.821963	1.144309	0.567808	
Côte d'Ivoire	Deaths	Female	2012	0.877364	1.20853	0.612152	
Côte d'Ivoire	Deaths	Female	2013	0.93214	1.273685	0.635039	
Côte d'Ivoire	Deaths	Female	2014	0.960411	1.317495	0.640965	
Côte d'Ivoire	Deaths	Female	2015	1.053238	1.436327	0.690527	
Côte d'Ivoire	Deaths	Female	2016	1.101777	1.498985	0.729206	
Côte d'Ivoire	Deaths	Female	2017	1.13048	1.527506	0.771409	
Côte d'Ivoire	Deaths	Female	2018	1.157741	1.552547	0.795397	
Côte d'Ivoire	Deaths	Female	2019	1.138786	1.531881	0.77966	
Côte d'Ivoire	Deaths	Female	2020	1.165982	1.562182	0.811656	
Côte d'Ivoire	Deaths	Female	2021	1.159975	1.513587	0.817094	
Côte d'Ivoire	Deaths	Female	2022	1.371657	1.824768	0.935278	
Côte d'Ivoire	Deaths	Female	2023	1.475851	1.99391	0.998343	
Côte d'Ivoire	DALYs	Male	2010	37.33728	52.013	28.64981	
Côte d'Ivoire	DALYs	Male	2011	38.80198	53.9565	30.0096	
Côte d'Ivoire	DALYs	Male	2012	42.39706	58.22212	32.19887	
Côte d'Ivoire	DALYs	Male	2013	43.48472	57.91438	32.03726	
Côte d'Ivoire	DALYs	Male	2014	44.68136	58.27132	33.02139	
Côte d'Ivoire	DALYs	Male	2015	49.90167	66.22882	36.05021	
Côte d'Ivoire	DALYs	Male	2016	50.50471	68.71351	36.28288	
Côte d'Ivoire	DALYs	Male	2017	50.74071	68.9167	35.86898	
Côte d'Ivoire	DALYs	Male	2018	52.11543	74.09329	36.18242	
Côte d'Ivoire	DALYs	Male	2019	51.74916	74.61384	35.38804	
Côte d'Ivoire	DALYs	Male	2020	54.29703	78.95651	37.91333	
Côte d'Ivoire	DALYs	Male	2021	54.29094	76.13607	37.858	
Côte d'Ivoire	DALYs	Male	2022	61.526	85.62726	42.45685	
Côte d'Ivoire	DALYs	Male	2023	65.43349	92.78702	45.01308	
Côte d'Ivoire	DALYs	Female	2010	25.52721	36.30071	17.87747	
Côte d'Ivoire	DALYs	Female	2011	26.26764	36.39212	18.34695	
Côte d'Ivoire	DALYs	Female	2012	28.31454	38.58934	19.47463	

Côte d'Ivoire	DALYs	Female	2013	30.2088	41.0617	20.2533	
Côte d'Ivoire	DALYs	Female	2014	31.36823	43.42529	20.91392	
Côte d'Ivoire	DALYs	Female	2015	34.95607	48.02404	22.69564	
Côte d'Ivoire	DALYs	Female	2016	37.14298	50.94712	24.40202	
Côte d'Ivoire	DALYs	Female	2017	38.69945	52.78001	26.33374	
Côte d'Ivoire	DALYs	Female	2018	40.15986	54.81288	26.81013	
Côte d'Ivoire	DALYs	Female	2019	39.69134	53.93913	26.77165	
Côte d'Ivoire	DALYs	Female	2020	40.81141	55.16571	28.3098	
Côte d'Ivoire	DALYs	Female	2021	40.70134	52.87059	28.53386	
Côte d'Ivoire	DALYs	Female	2022	47.95545	64.81869	32.37374	
Côte d'Ivoire	DALYs	Female	2023	51.70447	70.08816	34.69755	
Cape Verde	Incidence	Male	2010	9.171881	12.74984	6.63816	
Cape Verde	Incidence	Male	2011	9.860298	13.66314	7.359925	
Cape Verde	Incidence	Male	2012	10.37296	14.32951	7.801797	
Cape Verde	Incidence	Male	2013	10.40053	14.40113	7.764674	
Cape Verde	Incidence	Male	2014	10.62824	15.08237	7.669999	
Cape Verde	Incidence	Male	2015	11.46922	16.21383	8.003479	
Cape Verde	Incidence	Male	2016	11.41749	15.85149	8.132365	
Cape Verde	Incidence	Male	2017	11.85814	16.41267	8.12634	
Cape Verde	Incidence	Male	2018	11.38499	15.98992	7.738196	
Cape Verde	Incidence	Male	2019	11.77724	16.49549	8.064039	
Cape Verde	Incidence	Male	2020	11.94868	17.15847	8.034212	
Cape Verde	Incidence	Male	2021	12.28297	18.04896	8.215201	
Cape Verde	Incidence	Male	2022	13.47455	19.52142	9.10841	
Cape Verde	Incidence	Male	2023	14.17256	19.88034	9.550436	
Cape Verde	Incidence	Female	2010	5.139047	7.63426	3.447952	
Cape Verde	Incidence	Female	2011	5.462109	8.044821	3.688347	
Cape Verde	Incidence	Female	2012	5.854119	8.524174	3.856834	
Cape Verde	Incidence	Female	2013	6.193842	9.022764	4.13568	
Cape Verde	Incidence	Female	2014	6.608123	9.497282	4.404873	
Cape Verde	Incidence	Female	2015	7.039147	10.12843	4.610082	
Cape Verde	Incidence	Female	2016	7.32515	10.89374	4.848623	
Cape Verde	Incidence	Female	2017	7.489724	11.10133	4.864038	
Cape Verde	Incidence	Female	2018	7.66442	11.28718	5.170206	
Cape Verde	Incidence	Female	2019	7.866742	11.68186	5.338478	
Cape Verde	Incidence	Female	2020	7.572467	10.89191	5.13321	

Cape Verde	Incidence	Female	2021	7.764026	10.89144	5.144611	
Cape Verde	Incidence	Female	2022	9.549471	13.84213	6.500038	
Cape Verde	Incidence	Female	2023	10.35525	15.23346	7.006014	
Cape Verde	Deaths	Male	2010	9.723367	13.49409	7.091413	
Cape Verde	Deaths	Male	2011	10.42043	14.26278	7.829713	
Cape Verde	Deaths	Male	2012	10.94116	14.9366	8.3061	
Cape Verde	Deaths	Male	2013	10.94169	15.07462	8.184146	
Cape Verde	Deaths	Male	2014	11.1423	15.44936	8.082558	
Cape Verde	Deaths	Male	2015	11.96777	16.92027	8.452834	
Cape Verde	Deaths	Male	2016	11.8894	16.39261	8.454	
Cape Verde	Deaths	Male	2017	12.30401	16.82609	8.44613	
Cape Verde	Deaths	Male	2018	11.65079	16.38187	7.970061	
Cape Verde	Deaths	Male	2019	12.02294	17.06017	8.295322	
Cape Verde	Deaths	Male	2020	12.17428	17.53569	8.274159	
Cape Verde	Deaths	Male	2021	12.46823	18.37409	8.332944	
Cape Verde	Deaths	Male	2022	13.62694	19.59885	9.200025	
Cape Verde	Deaths	Male	2023	14.26713	19.89052	9.616095	
Cape Verde	Deaths	Female	2010	5.568216	8.034864	3.744228	
Cape Verde	Deaths	Female	2011	5.891656	8.455463	3.960301	
Cape Verde	Deaths	Female	2012	6.342461	9.103233	4.191449	
Cape Verde	Deaths	Female	2013	6.702238	9.651365	4.534566	
Cape Verde	Deaths	Female	2014	7.125416	10.16859	4.790428	
Cape Verde	Deaths	Female	2015	7.549451	10.56927	4.994819	
Cape Verde	Deaths	Female	2016	7.818816	11.28768	5.221254	
Cape Verde	Deaths	Female	2017	7.977495	11.54971	5.190585	
Cape Verde	Deaths	Female	2018	8.161235	11.75492	5.461486	
Cape Verde	Deaths	Female	2019	8.355321	11.96731	5.687109	
Cape Verde	Deaths	Female	2020	8.079058	11.38463	5.514347	
Cape Verde	Deaths	Female	2021	8.277081	11.53074	5.493134	
Cape Verde	Deaths	Female	2022	10.00695	14.36427	6.7972	
Cape Verde	Deaths	Female	2023	10.77705	15.4775	7.277091	
Cape Verde	DALYs	Male	2010	224.2274	323.7709	162.2211	
Cape Verde	DALYs	Male	2011	243.0038	344.2576	181.7164	
Cape Verde	DALYs	Male	2012	257.0713	370.0632	190.566	
Cape Verde	DALYs	Male	2013	258.9728	373.2017	190.3372	
Cape Verde	DALYs	Male	2014	265.6804	389.7581	191.5107	

Cape Verde	DALYs	Male	2015	288.7877	415.7676	200.8588	
Cape Verde	DALYs	Male	2016	288.966	407.7409	203.7552	
Cape Verde	DALYs	Male	2017	303.202	428.6746	209.1859	
Cape Verde	DALYs	Male	2018	300.5567	426.5349	204.3847	
Cape Verde	DALYs	Male	2019	312.4657	453.8102	212.0049	
Cape Verde	DALYs	Male	2020	319.4206	463.4344	217.0023	
Cape Verde	DALYs	Male	2021	329.1441	478.4476	219.2508	
Cape Verde	DALYs	Male	2022	358.1856	533.4056	243.5138	
Cape Verde	DALYs	Male	2023	376.4277	534.3754	255.4753	
Cape Verde	DALYs	Female	2010	118.1959	178.59	79.7612	
Cape Verde	DALYs	Female	2011	126.821	187.8945	85.7893	
Cape Verde	DALYs	Female	2012	135.8649	201.9644	89.23422	
Cape Verde	DALYs	Female	2013	143.8999	214.8764	93.11052	
Cape Verde	DALYs	Female	2014	154.1745	228.685	99.82102	
Cape Verde	DALYs	Female	2015	165.8972	247.4699	106.2979	
Cape Verde	DALYs	Female	2016	174.7828	262.9303	112.7175	
Cape Verde	DALYs	Female	2017	180.2874	276.296	116.0311	
Cape Verde	DALYs	Female	2018	185.5829	280.9184	125.7073	
Cape Verde	DALYs	Female	2019	191.8371	301.1261	129.7402	
Cape Verde	DALYs	Female	2020	183.5618	279.8405	125.909	
Cape Verde	DALYs	Female	2021	187.6263	277.2534	126.1729	
Cape Verde	DALYs	Female	2022	235.0686	353.7563	157.7342	
Cape Verde	DALYs	Female	2023	256.0755	390.1573	174.9018	
Cambodia	Incidence	Male	2010	8.696076	13.01294	5.810663	
Cambodia	Incidence	Male	2011	8.664066	13.00178	5.845485	
Cambodia	Incidence	Male	2012	8.915231	13.35604	5.841554	
Cambodia	Incidence	Male	2013	9.230149	13.77169	5.972317	
Cambodia	Incidence	Male	2014	9.339114	14.26982	6.327727	
Cambodia	Incidence	Male	2015	9.883106	14.90067	6.719105	
Cambodia	Incidence	Male	2016	10.22524	15.41482	6.782164	
Cambodia	Incidence	Male	2017	10.33709	15.81353	6.680865	
Cambodia	Incidence	Male	2018	10.72853	16.30228	7.2303	
Cambodia	Incidence	Male	2019	11.13031	16.93528	7.403981	
Cambodia	Incidence	Male	2020	11.71191	18.08595	7.823804	
Cambodia	Incidence	Male	2021	11.57824	18.49895	7.64831	
Cambodia	Incidence	Male	2022	12.37412	18.87726	8.339648	

Cambodia	Incidence	Male	2023	13.14324	20.09846	9.191887	
Cambodia	Incidence	Female	2010	4.358297	6.721342	2.723891	
Cambodia	Incidence	Female	2011	4.469595	6.690038	2.737649	
Cambodia	Incidence	Female	2012	4.683514	7.146027	2.783183	
Cambodia	Incidence	Female	2013	4.885549	7.560115	3.025704	
Cambodia	Incidence	Female	2014	4.986582	7.453013	3.165321	
Cambodia	Incidence	Female	2015	5.183227	7.811073	3.262063	
Cambodia	Incidence	Female	2016	5.367486	7.814565	3.2915	
Cambodia	Incidence	Female	2017	5.45017	8.064115	3.37867	
Cambodia	Incidence	Female	2018	5.662908	8.117708	3.650862	
Cambodia	Incidence	Female	2019	5.936082	8.536884	3.840802	
Cambodia	Incidence	Female	2020	6.20638	8.893285	4.017474	
Cambodia	Incidence	Female	2021	6.049441	8.510294	3.93268	
Cambodia	Incidence	Female	2022	6.58691	9.367774	3.992856	
Cambodia	Incidence	Female	2023	7.066723	10.0936	4.610923	
Cambodia	Deaths	Male	2010	8.645334	13.03473	5.779752	
Cambodia	Deaths	Male	2011	8.620295	13.05726	5.811545	
Cambodia	Deaths	Male	2012	8.878574	13.37836	5.785159	
Cambodia	Deaths	Male	2013	9.201283	13.80128	5.946544	
Cambodia	Deaths	Male	2014	9.3207	14.26508	6.312686	
Cambodia	Deaths	Male	2015	9.872116	14.93536	6.691021	
Cambodia	Deaths	Male	2016	10.22223	15.49941	6.761606	
Cambodia	Deaths	Male	2017	10.34363	15.98422	6.703727	
Cambodia	Deaths	Male	2018	10.74939	16.47095	7.203697	
Cambodia	Deaths	Male	2019	11.16683	17.14306	7.420422	
Cambodia	Deaths	Male	2020	11.77578	18.47629	7.912038	
Cambodia	Deaths	Male	2021	11.67508	18.94123	7.738598	
Cambodia	Deaths	Male	2022	12.44954	19.07324	8.354228	
Cambodia	Deaths	Male	2023	13.1922	20.14272	9.185142	
Cambodia	Deaths	Female	2010	4.234273	6.516843	2.647258	
Cambodia	Deaths	Female	2011	4.349695	6.483596	2.654298	
Cambodia	Deaths	Female	2012	4.566754	6.959237	2.707139	
Cambodia	Deaths	Female	2013	4.775255	7.416161	2.944307	
Cambodia	Deaths	Female	2014	4.887093	7.314436	3.106469	
Cambodia	Deaths	Female	2015	5.093324	7.678301	3.185372	
Cambodia	Deaths	Female	2016	5.286394	7.707457	3.23768	

Cambodia	Deaths	Female	2017	5.381474	7.995412	3.322199	
Cambodia	Deaths	Female	2018	5.602962	8.049982	3.601246	
Cambodia	Deaths	Female	2019	5.88588	8.507493	3.787474	
Cambodia	Deaths	Female	2020	6.167279	8.887511	3.998825	
Cambodia	Deaths	Female	2021	6.022648	8.498496	3.921908	
Cambodia	Deaths	Female	2022	6.546991	9.353405	3.929167	
Cambodia	Deaths	Female	2023	7.003914	10.02979	4.529348	
Cambodia	DALYs	Male	2010	246.4986	359.4973	165.1774	
Cambodia	DALYs	Male	2011	244.7375	351.586	166.5067	
Cambodia	DALYs	Male	2012	250.7872	368.3409	167.098	
Cambodia	DALYs	Male	2013	258.6073	383.0616	169.8166	
Cambodia	DALYs	Male	2014	260.5324	393.1378	175.9733	
Cambodia	DALYs	Male	2015	274.7403	411.2832	185.9951	
Cambodia	DALYs	Male	2016	283.3077	421.105	189.2784	
Cambodia	DALYs	Male	2017	285.4552	428.6292	187.9382	
Cambodia	DALYs	Male	2018	295.031	436.4616	199.0913	
Cambodia	DALYs	Male	2019	304.6405	451.8822	205.9118	
Cambodia	DALYs	Male	2020	318.4546	478.295	211.4244	
Cambodia	DALYs	Male	2021	312.5685	484.8235	207.9899	
Cambodia	DALYs	Male	2022	334.5334	506.472	226.6293	
Cambodia	DALYs	Male	2023	357.2174	545.191	250.3804	
Cambodia	DALYs	Female	2010	128.0633	197.5916	78.79095	
Cambodia	DALYs	Female	2011	130.5254	197.2138	78.77891	
Cambodia	DALYs	Female	2012	135.7717	207.6135	80.28361	
Cambodia	DALYs	Female	2013	140.4365	215.786	87.55658	
Cambodia	DALYs	Female	2014	142.0527	214.0229	88.13488	
Cambodia	DALYs	Female	2015	146.3853	217.8672	90.43496	
Cambodia	DALYs	Female	2016	150.3771	220.5702	91.99384	
Cambodia	DALYs	Female	2017	151.3813	221.3194	94.47986	
Cambodia	DALYs	Female	2018	156.1313	224.509	100.4193	
Cambodia	DALYs	Female	2019	162.3755	231.159	104.5915	
Cambodia	DALYs	Female	2020	168.4951	238.2855	109.4293	
Cambodia	DALYs	Female	2021	163.2934	227.0143	106.298	
Cambodia	DALYs	Female	2022	177.6473	250.0237	108.1803	
Cambodia	DALYs	Female	2023	191.9602	274.6846	125.9123	
Cameroon	Incidence	Male	2010	3.155977	4.424154	2.145939	

Cameroon	Incidence	Male	2011	3.180495	4.560902	2.165745	
Cameroon	Incidence	Male	2012	3.212509	4.583942	2.147554	
Cameroon	Incidence	Male	2013	3.201043	4.457852	2.097943	
Cameroon	Incidence	Male	2014	3.210672	4.629476	2.063144	
Cameroon	Incidence	Male	2015	3.436619	4.97811	2.26652	
Cameroon	Incidence	Male	2016	3.34651	4.848727	2.151335	
Cameroon	Incidence	Male	2017	3.335477	4.809266	2.172742	
Cameroon	Incidence	Male	2018	3.37671	4.874592	2.146873	
Cameroon	Incidence	Male	2019	3.427996	4.941419	2.162897	
Cameroon	Incidence	Male	2020	3.485809	5.156254	2.217413	
Cameroon	Incidence	Male	2021	3.566872	5.511387	2.285333	
Cameroon	Incidence	Male	2022	3.760274	5.775607	2.408032	
Cameroon	Incidence	Male	2023	3.993128	6.089067	2.442812	
Cameroon	Incidence	Female	2010	1.391782	2.07373	0.935131	
Cameroon	Incidence	Female	2011	1.421729	2.069856	0.975319	
Cameroon	Incidence	Female	2012	1.458424	2.138131	0.986806	
Cameroon	Incidence	Female	2013	1.47417	2.178688	0.991805	
Cameroon	Incidence	Female	2014	1.455409	2.139416	0.963235	
Cameroon	Incidence	Female	2015	1.519541	2.152532	1.004007	
Cameroon	Incidence	Female	2016	1.538857	2.159911	1.019855	
Cameroon	Incidence	Female	2017	1.586432	2.173949	1.090459	
Cameroon	Incidence	Female	2018	1.657795	2.256962	1.139901	
Cameroon	Incidence	Female	2019	1.704044	2.301177	1.165412	
Cameroon	Incidence	Female	2020	1.770906	2.362543	1.201761	
Cameroon	Incidence	Female	2021	1.837366	2.516868	1.221421	
Cameroon	Incidence	Female	2022	1.969637	2.756111	1.307502	
Cameroon	Incidence	Female	2023	2.103261	3.015673	1.367254	
Cameroon	Deaths	Male	2010	3.163138	4.428116	2.15232	
Cameroon	Deaths	Male	2011	3.186857	4.55055	2.170462	
Cameroon	Deaths	Male	2012	3.218417	4.606787	2.150536	
Cameroon	Deaths	Male	2013	3.209779	4.457451	2.108007	
Cameroon	Deaths	Male	2014	3.214671	4.610637	2.071319	
Cameroon	Deaths	Male	2015	3.439872	4.976047	2.264593	
Cameroon	Deaths	Male	2016	3.341446	4.846435	2.146309	
Cameroon	Deaths	Male	2017	3.322007	4.7933	2.15536	
Cameroon	Deaths	Male	2018	3.358334	4.840245	2.13033	

Cameroon	Deaths	Male	2019	3.404813	4.889894	2.146008	
Cameroon	Deaths	Male	2020	3.460815	5.102513	2.201768	
Cameroon	Deaths	Male	2021	3.538258	5.455752	2.268201	
Cameroon	Deaths	Male	2022	3.716307	5.720808	2.37783	
Cameroon	Deaths	Male	2023	3.940024	6.023958	2.395997	
Cameroon	Deaths	Female	2010	1.386651	2.063027	0.929499	
Cameroon	Deaths	Female	2011	1.415315	2.059405	0.96721	
Cameroon	Deaths	Female	2012	1.447365	2.139075	0.969581	
Cameroon	Deaths	Female	2013	1.462034	2.154717	0.982247	
Cameroon	Deaths	Female	2014	1.443744	2.119005	0.956169	
Cameroon	Deaths	Female	2015	1.505791	2.126916	0.992236	
Cameroon	Deaths	Female	2016	1.519128	2.125364	1.007322	
Cameroon	Deaths	Female	2017	1.557911	2.132023	1.067213	
Cameroon	Deaths	Female	2018	1.621811	2.213989	1.116602	
Cameroon	Deaths	Female	2019	1.662617	2.246024	1.13628	
Cameroon	Deaths	Female	2020	1.724365	2.296697	1.171388	
Cameroon	Deaths	Female	2021	1.784294	2.426641	1.184867	
Cameroon	Deaths	Female	2022	1.900582	2.670139	1.258878	
Cameroon	Deaths	Female	2023	2.023541	2.927328	1.321683	
Cameroon	DALYs	Male	2010	86.4992	121.9835	58.30442	
Cameroon	DALYs	Male	2011	87.0672	125.7309	58.73322	
Cameroon	DALYs	Male	2012	87.63153	124.9094	58.34822	
Cameroon	DALYs	Male	2013	87.04601	122.8322	56.95477	
Cameroon	DALYs	Male	2014	87.57317	127.5426	56.30153	
Cameroon	DALYs	Male	2015	93.86472	136.2457	61.62708	
Cameroon	DALYs	Male	2016	91.89192	133.0154	58.70758	
Cameroon	DALYs	Male	2017	91.98849	132.9741	59.37388	
Cameroon	DALYs	Male	2018	93.46039	135.8465	59.75014	
Cameroon	DALYs	Male	2019	95.15642	138.6493	60.25565	
Cameroon	DALYs	Male	2020	96.95223	144.3509	61.76111	
Cameroon	DALYs	Male	2021	99.41239	154.2448	64.60748	
Cameroon	DALYs	Male	2022	105.2278	159.9561	67.27414	
Cameroon	DALYs	Male	2023	111.9472	168.5349	69.36717	
Cameroon	DALYs	Female	2010	39.35185	57.99914	26.81183	
Cameroon	DALYs	Female	2011	40.17248	58.31926	27.99654	
Cameroon	DALYs	Female	2012	41.40852	60.22686	28.26508	

Cameroon	DALYs	Female	2013	41.90567	61.49917	28.23101	
Cameroon	DALYs	Female	2014	41.32709	61.25792	27.33627	
Cameroon	DALYs	Female	2015	43.22416	61.31552	28.78632	
Cameroon	DALYs	Female	2016	44.05811	61.97281	29.36691	
Cameroon	DALYs	Female	2017	45.86561	63.2708	31.21482	
Cameroon	DALYs	Female	2018	48.29229	65.82385	32.77332	
Cameroon	DALYs	Female	2019	49.88922	66.73338	34.16567	
Cameroon	DALYs	Female	2020	51.96879	69.77811	35.53353	
Cameroon	DALYs	Female	2021	53.95989	74.54177	35.71228	
Cameroon	DALYs	Female	2022	58.5402	82.20465	38.76684	
Cameroon	DALYs	Female	2023	62.8202	87.77359	40.92752	
Canada	Incidence	Male	2010	82.07296	89.66179	74.82737	
Canada	Incidence	Male	2011	84.04186	92.24088	77.24374	
Canada	Incidence	Male	2012	84.69176	92.23567	77.6049	
Canada	Incidence	Male	2013	83.48897	91.99162	76.20233	
Canada	Incidence	Male	2014	85.71353	94.9948	77.62833	
Canada	Incidence	Male	2015	79.81841	88.42909	71.82387	
Canada	Incidence	Male	2016	79.46253	89.08572	70.98107	
Canada	Incidence	Male	2017	80.0527	89.09597	71.5018	
Canada	Incidence	Male	2018	79.21894	89.56117	70.77277	
Canada	Incidence	Male	2019	77.54815	86.66958	69.24741	
Canada	Incidence	Male	2020	76.5594	85.57837	68.03393	
Canada	Incidence	Male	2021	75.74498	84.3089	67.10778	
Canada	Incidence	Male	2022	75.98768	85.65222	67.24356	
Canada	Incidence	Male	2023	81.16408	93.37847	71.16461	
Canada	Incidence	Female	2010	73.01647	80.71912	66.09266	
Canada	Incidence	Female	2011	74.01276	82.63116	65.76464	
Canada	Incidence	Female	2012	74.65144	83.49936	66.09782	
Canada	Incidence	Female	2013	75.23341	83.73329	66.733	
Canada	Incidence	Female	2014	76.191	85.29302	67.14264	
Canada	Incidence	Female	2015	74.39668	82.58052	65.20039	
Canada	Incidence	Female	2016	74.771	84.71938	65.19663	
Canada	Incidence	Female	2017	74.28678	83.82902	64.58397	
Canada	Incidence	Female	2018	73.70495	83.34291	63.84068	
Canada	Incidence	Female	2019	72.41229	82.42147	63.42949	
Canada	Incidence	Female	2020	72.15483	81.69689	62.64037	

Canada	Incidence	Female	2021	72.40041	82.31646	62.14716	
Canada	Incidence	Female	2022	73.63162	83.58875	63.77371	
Canada	Incidence	Female	2023	75.14697	86.19497	64.46477	
Canada	Deaths	Male	2010	66.55817	70.11242	62.92619	
Canada	Deaths	Male	2011	68.1747	71.71809	64.46158	
Canada	Deaths	Male	2012	68.92584	72.35155	65.45115	
Canada	Deaths	Male	2013	68.02965	71.74569	64.06414	
Canada	Deaths	Male	2014	70.05117	74.11848	65.47826	
Canada	Deaths	Male	2015	65.41669	69.3571	61.65442	
Canada	Deaths	Male	2016	65.31141	69.21303	61.42095	
Canada	Deaths	Male	2017	66.02832	70.09431	62.05866	
Canada	Deaths	Male	2018	65.38485	69.46162	61.54983	
Canada	Deaths	Male	2019	63.99744	68.46788	60.31533	
Canada	Deaths	Male	2020	63.02629	67.78048	59.18222	
Canada	Deaths	Male	2021	62.16239	67.24846	57.96448	
Canada	Deaths	Male	2022	62.47902	67.46283	57.68991	
Canada	Deaths	Male	2023	66.92613	73.55141	60.99423	
Canada	Deaths	Female	2010	54.69557	58.51958	49.34328	
Canada	Deaths	Female	2011	55.53153	59.21168	50.17945	
Canada	Deaths	Female	2012	56.20673	59.84268	50.53482	
Canada	Deaths	Female	2013	56.98456	60.63779	51.23327	
Canada	Deaths	Female	2014	58.01474	61.90304	52.38255	
Canada	Deaths	Female	2015	56.82134	60.39748	50.99545	
Canada	Deaths	Female	2016	57.36281	61.07885	51.57108	
Canada	Deaths	Female	2017	57.24597	60.77893	51.64548	
Canada	Deaths	Female	2018	57.01483	60.32628	51.23963	
Canada	Deaths	Female	2019	56.00427	59.5277	50.32118	
Canada	Deaths	Female	2020	55.62392	58.96794	50.22161	
Canada	Deaths	Female	2021	55.61517	58.95279	50.47684	
Canada	Deaths	Female	2022	56.63208	60.03837	51.44039	
Canada	Deaths	Female	2023	57.91132	62.08817	51.46924	
Canada	DALYs	Male	2010	1424.426	1502.872	1349.769	
Canada	DALYs	Male	2011	1447.941	1519.445	1380.985	
Canada	DALYs	Male	2012	1449.97	1519.455	1376.933	
Canada	DALYs	Male	2013	1421.429	1494.244	1347.953	
Canada	DALYs	Male	2014	1445.597	1525.785	1364.623	

Canada	DALYs	Male	2015	1340.665	1415.478	1269.296	
Canada	DALYs	Male	2016	1335.413	1408.041	1264.202	
Canada	DALYs	Male	2017	1335.481	1408.786	1263.613	
Canada	DALYs	Male	2018	1316.389	1392.561	1243.422	
Canada	DALYs	Male	2019	1278.944	1368.768	1211.371	
Canada	DALYs	Male	2020	1256.548	1344.378	1186.235	
Canada	DALYs	Male	2021	1237.976	1340.989	1159.739	
Canada	DALYs	Male	2022	1235.754	1334.668	1153.099	
Canada	DALYs	Male	2023	1314.517	1440.522	1213.94	
Canada	DALYs	Female	2010	1176.649	1252.891	1081.971	
Canada	DALYs	Female	2011	1186.098	1262.655	1090.447	
Canada	DALYs	Female	2012	1191.652	1270.918	1096.156	
Canada	DALYs	Female	2013	1196.504	1266.443	1099.275	
Canada	DALYs	Female	2014	1207.456	1272.359	1107.706	
Canada	DALYs	Female	2015	1174.151	1238.023	1072.527	
Canada	DALYs	Female	2016	1174.512	1237.465	1081.552	
Canada	DALYs	Female	2017	1157.18	1215.396	1063.79	
Canada	DALYs	Female	2018	1135.946	1195.041	1043.658	
Canada	DALYs	Female	2019	1105.749	1165.279	1023.79	
Canada	DALYs	Female	2020	1092.298	1152.986	1012.003	
Canada	DALYs	Female	2021	1085.536	1144.487	1006.533	
Canada	DALYs	Female	2022	1098.505	1162.834	1014.208	
Canada	DALYs	Female	2023	1119.967	1192.094	1012.372	
Central African	Incidence	Male	2010	1.841363	3.238083	0.90186	
Central African	Incidence	Male	2011	1.89213	3.237792	0.938188	
Central African	Incidence	Male	2012	1.887551	3.166655	0.960966	
Central African	Incidence	Male	2013	1.874229	3.239188	0.944982	
Central African	Incidence	Male	2014	1.859847	3.172141	0.93325	
Central African	Incidence	Male	2015	1.955825	3.294592	0.963234	
Central African	Incidence	Male	2016	1.981007	3.342597	0.950934	
Central African	Incidence	Male	2017	2.020894	3.490135	0.962259	
Central African	Incidence	Male	2018	2.025341	3.529149	0.978113	
Central African	Incidence	Male	2019	2.105724	3.758642	1.022352	
Central African	Incidence	Male	2020	2.206884	3.944449	1.0831	
Central African	Incidence	Male	2021	2.209843	3.918096	1.098199	
Central African	Incidence	Male	2022	2.537359	4.535991	1.219905	

Central African	Incidence	Male	2023	2.656613	4.696328	1.280678	
Central African	Incidence	Female	2010	1.210252	2.195302	0.679737	
Central African	Incidence	Female	2011	1.242501	2.190418	0.708048	
Central African	Incidence	Female	2012	1.229497	2.150852	0.706579	
Central African	Incidence	Female	2013	1.224661	2.205231	0.695839	
Central African	Incidence	Female	2014	1.215869	2.206422	0.676757	
Central African	Incidence	Female	2015	1.255433	2.196002	0.673055	
Central African	Incidence	Female	2016	1.293393	2.310835	0.687244	
Central African	Incidence	Female	2017	1.332738	2.428558	0.703262	
Central African	Incidence	Female	2018	1.340796	2.37085	0.720077	
Central African	Incidence	Female	2019	1.394402	2.468515	0.738011	
Central African	Incidence	Female	2020	1.407821	2.578686	0.747913	
Central African	Incidence	Female	2021	1.417478	2.678197	0.765512	
Central African	Incidence	Female	2022	1.642803	2.985476	0.875113	
Central African	Incidence	Female	2023	1.722863	3.268183	0.910081	
Central African	Deaths	Male	2010	1.818134	3.198366	0.891361	
Central African	Deaths	Male	2011	1.865711	3.190721	0.920923	
Central African	Deaths	Male	2012	1.859458	3.102787	0.947168	
Central African	Deaths	Male	2013	1.844895	3.168372	0.929313	
Central African	Deaths	Male	2014	1.830358	3.110996	0.917866	
Central African	Deaths	Male	2015	1.925506	3.214301	0.948716	
Central African	Deaths	Male	2016	1.951755	3.303522	0.932764	
Central African	Deaths	Male	2017	1.991659	3.423163	0.944251	
Central African	Deaths	Male	2018	1.995828	3.436654	0.960027	
Central African	Deaths	Male	2019	2.075201	3.701112	1.002179	
Central African	Deaths	Male	2020	2.181176	3.909276	1.064582	
Central African	Deaths	Male	2021	2.18598	3.865261	1.08182	
Central African	Deaths	Male	2022	2.505421	4.461422	1.206778	
Central African	Deaths	Male	2023	2.623225	4.630665	1.263197	
Central African	Deaths	Female	2010	1.195166	2.17006	0.668029	
Central African	Deaths	Female	2011	1.226112	2.167993	0.690663	
Central African	Deaths	Female	2012	1.213078	2.125192	0.695896	
Central African	Deaths	Female	2013	1.207753	2.174235	0.684785	
Central African	Deaths	Female	2014	1.198464	2.174932	0.661458	
Central African	Deaths	Female	2015	1.235955	2.160479	0.662831	
Central African	Deaths	Female	2016	1.271539	2.256673	0.667779	

Central African	Deaths	Female	2017	1.308022	2.391758	0.679637	
Central African	Deaths	Female	2018	1.316135	2.320824	0.694393	
Central African	Deaths	Female	2019	1.368201	2.412285	0.71579	
Central African	Deaths	Female	2020	1.381867	2.525398	0.733145	
Central African	Deaths	Female	2021	1.390954	2.625551	0.741445	
Central African	Deaths	Female	2022	1.607033	2.926078	0.850962	
Central African	Deaths	Female	2023	1.684098	3.174182	0.879611	
Central African	DALYs	Male	2010	53.31189	94.02632	26.12026	
Central African	DALYs	Male	2011	54.90523	94.50877	27.48313	
Central African	DALYs	Male	2012	54.83866	92.39303	28.02325	
Central African	DALYs	Male	2013	54.53379	95.4349	27.888	
Central African	DALYs	Male	2014	54.11089	92.94615	27.22385	
Central African	DALYs	Male	2015	56.80842	97.04654	28.01314	
Central African	DALYs	Male	2016	57.35228	96.90897	27.93964	
Central African	DALYs	Male	2017	58.36924	101.3479	28.06384	
Central African	DALYs	Male	2018	58.43998	104.3787	28.46063	
Central African	DALYs	Male	2019	60.70261	109.9424	29.65002	
Central African	DALYs	Male	2020	63.18439	112.2227	31.46415	
Central African	DALYs	Male	2021	63.10187	110.9043	31.62867	
Central African	DALYs	Male	2022	72.39926	128.0514	34.81058	
Central African	DALYs	Male	2023	75.5959	132.3947	36.81535	
Central African	DALYs	Female	2010	35.38934	63.86977	20.30918	
Central African	DALYs	Female	2011	36.36869	63.83203	20.80973	
Central African	DALYs	Female	2012	35.9809	62.59969	20.9544	
Central African	DALYs	Female	2013	35.86563	63.9208	20.60586	
Central African	DALYs	Female	2014	35.64201	64.35558	20.009	
Central African	DALYs	Female	2015	36.89327	64.57611	20.19628	
Central African	DALYs	Female	2016	38.10229	68.9333	20.5698	
Central African	DALYs	Female	2017	39.39226	71.63911	20.87225	
Central African	DALYs	Female	2018	39.59682	70.28073	21.62176	
Central African	DALYs	Female	2019	41.20176	73.42758	21.86236	
Central African	DALYs	Female	2020	41.54926	75.54734	22.65086	
Central African	DALYs	Female	2021	41.83595	77.09846	23.11775	
Central African	DALYs	Female	2022	48.6653	85.70047	26.46808	
Central African	DALYs	Female	2023	51.06231	93.96796	27.56305	
Chad	Incidence	Male	2010	2.784959	4.11374	1.815814	

Chad	Incidence	Male	2011	2.796918	4.136302	1.841087	
Chad	Incidence	Male	2012	2.80956	4.192749	1.874074	
Chad	Incidence	Male	2013	2.751658	4.001265	1.784636	
Chad	Incidence	Male	2014	2.720286	3.982291	1.767867	
Chad	Incidence	Male	2015	2.852298	4.097329	1.861686	
Chad	Incidence	Male	2016	2.784974	4.073948	1.82331	
Chad	Incidence	Male	2017	2.738542	4.044484	1.800512	
Chad	Incidence	Male	2018	2.713508	4.03776	1.776134	
Chad	Incidence	Male	2019	2.688742	4.139965	1.732986	
Chad	Incidence	Male	2020	2.771958	4.217616	1.814398	
Chad	Incidence	Male	2021	2.63745	3.960947	1.697462	
Chad	Incidence	Male	2022	2.986589	4.441173	2.037292	
Chad	Incidence	Male	2023	3.074725	4.631375	2.047175	
Chad	Incidence	Female	2010	0.917418	1.414691	0.601326	
Chad	Incidence	Female	2011	0.920437	1.348758	0.595082	
Chad	Incidence	Female	2012	0.916745	1.384593	0.604197	
Chad	Incidence	Female	2013	0.912024	1.351857	0.592278	
Chad	Incidence	Female	2014	0.9028	1.342962	0.578393	
Chad	Incidence	Female	2015	0.919997	1.368057	0.58023	
Chad	Incidence	Female	2016	0.919103	1.315694	0.577889	
Chad	Incidence	Female	2017	0.914747	1.293127	0.578108	
Chad	Incidence	Female	2018	0.918965	1.299459	0.599859	
Chad	Incidence	Female	2019	0.920782	1.325016	0.607136	
Chad	Incidence	Female	2020	0.903443	1.298523	0.607526	
Chad	Incidence	Female	2021	0.89065	1.261885	0.592462	
Chad	Incidence	Female	2022	1.029761	1.404039	0.665717	
Chad	Incidence	Female	2023	1.069499	1.499821	0.710425	
Chad	Deaths	Male	2010	2.811283	4.132318	1.834574	
Chad	Deaths	Male	2011	2.821379	4.165165	1.863098	
Chad	Deaths	Male	2012	2.831994	4.228222	1.884934	
Chad	Deaths	Male	2013	2.773287	4.041288	1.800782	
Chad	Deaths	Male	2014	2.7447	4.024279	1.78469	
Chad	Deaths	Male	2015	2.873924	4.133327	1.862911	
Chad	Deaths	Male	2016	2.803957	4.113934	1.819662	
Chad	Deaths	Male	2017	2.756053	4.089539	1.805502	
Chad	Deaths	Male	2018	2.72969	4.053004	1.791554	

Chad	Deaths	Male	2019	2.706704	4.166026	1.751387	
Chad	Deaths	Male	2020	2.796566	4.248317	1.844581	
Chad	Deaths	Male	2021	2.651606	3.990354	1.696924	
Chad	Deaths	Male	2022	2.995663	4.486762	2.045933	
Chad	Deaths	Male	2023	3.082063	4.648881	2.036087	
Chad	Deaths	Female	2010	0.903567	1.394645	0.59359	
Chad	Deaths	Female	2011	0.906024	1.330142	0.584924	
Chad	Deaths	Female	2012	0.901535	1.368308	0.597806	
Chad	Deaths	Female	2013	0.896444	1.330279	0.583203	
Chad	Deaths	Female	2014	0.886972	1.323611	0.572566	
Chad	Deaths	Female	2015	0.901186	1.332866	0.572867	
Chad	Deaths	Female	2016	0.898315	1.290406	0.559695	
Chad	Deaths	Female	2017	0.892394	1.257225	0.56464	
Chad	Deaths	Female	2018	0.894586	1.26394	0.585671	
Chad	Deaths	Female	2019	0.894848	1.286456	0.590976	
Chad	Deaths	Female	2020	0.877058	1.252878	0.591174	
Chad	Deaths	Female	2021	0.862436	1.214478	0.577227	
Chad	Deaths	Female	2022	0.993448	1.35407	0.642007	
Chad	Deaths	Female	2023	1.030205	1.437829	0.6899	
Chad	DALYs	Male	2010	75.64653	113.9168	48.79498	
Chad	DALYs	Male	2011	75.99388	113.776	49.37605	
Chad	DALYs	Male	2012	76.40216	114.3073	50.59207	
Chad	DALYs	Male	2013	74.75918	108.0677	48.20877	
Chad	DALYs	Male	2014	74.01695	108.3423	48.72357	
Chad	DALYs	Male	2015	77.7492	112.4147	50.94451	
Chad	DALYs	Male	2016	75.95797	111.4628	49.97743	
Chad	DALYs	Male	2017	74.68225	109.8978	49.08742	
Chad	DALYs	Male	2018	73.98287	110.5531	47.83644	
Chad	DALYs	Male	2019	73.40546	113.0578	48.1052	
Chad	DALYs	Male	2020	75.35023	115.2038	48.59588	
Chad	DALYs	Male	2021	72.18302	108.4225	46.77395	
Chad	DALYs	Male	2022	81.86015	120.7644	55.00734	
Chad	DALYs	Male	2023	84.25752	127.5978	56.7926	
Chad	DALYs	Female	2010	27.22063	41.80144	17.71688	
Chad	DALYs	Female	2011	27.32843	40.08472	17.64506	
Chad	DALYs	Female	2012	27.26128	40.6707	17.69203	

Chad	DALYs	Female	2013	27.12582	40.48064	17.2269	
Chad	DALYs	Female	2014	26.91272	40.04335	16.89619	
Chad	DALYs	Female	2015	27.60049	41.37191	17.39725	
Chad	DALYs	Female	2016	27.69166	40.01039	17.1008	
Chad	DALYs	Female	2017	27.66806	39.7142	17.38106	
Chad	DALYs	Female	2018	27.91307	40.28106	18.05726	
Chad	DALYs	Female	2019	28.09262	40.71726	18.37135	
Chad	DALYs	Female	2020	27.61442	40.07274	18.47744	
Chad	DALYs	Female	2021	27.38733	39.43539	18.07958	
Chad	DALYs	Female	2022	31.80551	43.28342	20.44282	
Chad	DALYs	Female	2023	33.12878	47.02228	21.55439	
Chile	Incidence	Male	2010	23.91346	24.98034	22.92835	
Chile	Incidence	Male	2011	24.4071	25.65624	23.29087	
Chile	Incidence	Male	2012	23.83487	24.87968	22.72868	
Chile	Incidence	Male	2013	24.63157	25.70765	23.46169	
Chile	Incidence	Male	2014	24.46354	25.57375	23.22996	
Chile	Incidence	Male	2015	24.6404	25.65721	23.51641	
Chile	Incidence	Male	2016	24.74319	26.07336	23.5699	
Chile	Incidence	Male	2017	24.78029	26.06787	23.63131	
Chile	Incidence	Male	2018	25.00815	26.32838	23.73319	
Chile	Incidence	Male	2019	24.80123	26.27836	23.28997	
Chile	Incidence	Male	2020	24.03797	25.34387	22.4982	
Chile	Incidence	Male	2021	24.20643	25.58818	22.57834	
Chile	Incidence	Male	2022	25.42671	26.98755	23.75522	
Chile	Incidence	Male	2023	24.36036	25.96591	22.4439	
Chile	Incidence	Female	2010	13.81119	14.77188	12.68141	
Chile	Incidence	Female	2011	14.66521	15.74632	13.47981	
Chile	Incidence	Female	2012	15.42166	16.45673	14.32443	
Chile	Incidence	Female	2013	15.78148	16.80378	14.55535	
Chile	Incidence	Female	2014	15.93685	17.01279	14.5431	
Chile	Incidence	Female	2015	16.11121	17.15297	14.62541	
Chile	Incidence	Female	2016	16.32431	17.37855	14.72134	
Chile	Incidence	Female	2017	17.03116	18.2111	15.43078	
Chile	Incidence	Female	2018	17.00377	18.16756	15.46208	
Chile	Incidence	Female	2019	17.43753	18.80976	15.70282	
Chile	Incidence	Female	2020	16.675	17.87537	15.24337	

Chile	Incidence	Female	2021	17.20474	18.48404	15.6979	
Chile	Incidence	Female	2022	18.48231	19.93192	16.77175	
Chile	Incidence	Female	2023	18.71338	20.40245	16.65692	
Chile	Deaths	Male	2010	23.67271	24.59165	22.67259	
Chile	Deaths	Male	2011	24.16023	25.27403	23.01833	
Chile	Deaths	Male	2012	23.56955	24.49232	22.47217	
Chile	Deaths	Male	2013	24.39167	25.36285	23.27722	
Chile	Deaths	Male	2014	24.24419	25.23335	23.14731	
Chile	Deaths	Male	2015	24.4081	25.3444	23.46391	
Chile	Deaths	Male	2016	24.55895	25.60219	23.51024	
Chile	Deaths	Male	2017	24.67049	25.67521	23.6981	
Chile	Deaths	Male	2018	24.82913	25.82936	23.69674	
Chile	Deaths	Male	2019	24.57224	25.65912	23.36858	
Chile	Deaths	Male	2020	23.71017	24.72629	22.46288	
Chile	Deaths	Male	2021	23.81047	24.97691	22.49554	
Chile	Deaths	Male	2022	25.09262	26.41447	23.56719	
Chile	Deaths	Male	2023	23.98877	25.59631	22.26209	
Chile	Deaths	Female	2010	13.76741	14.60527	12.60641	
Chile	Deaths	Female	2011	14.54217	15.3951	13.38525	
Chile	Deaths	Female	2012	15.31543	16.26838	14.11317	
Chile	Deaths	Female	2013	15.69622	16.62366	14.37633	
Chile	Deaths	Female	2014	15.85798	16.81422	14.35602	
Chile	Deaths	Female	2015	16.00729	16.93367	14.43393	
Chile	Deaths	Female	2016	16.19945	17.09816	14.612	
Chile	Deaths	Female	2017	16.89487	17.83546	15.31511	
Chile	Deaths	Female	2018	16.80849	17.78748	15.30053	
Chile	Deaths	Female	2019	17.19502	18.27484	15.64922	
Chile	Deaths	Female	2020	16.37605	17.28188	15.00361	
Chile	Deaths	Female	2021	16.86958	17.83673	15.46995	
Chile	Deaths	Female	2022	18.17606	19.31656	16.4181	
Chile	Deaths	Female	2023	18.28035	19.55813	16.24205	
Chile	DALYs	Male	2010	555.8508	576.0054	536.5047	
Chile	DALYs	Male	2011	563.2546	585.8241	542.6595	
Chile	DALYs	Male	2012	547.1473	568.2224	525.4067	
Chile	DALYs	Male	2013	562.6356	583.6499	539.5917	
Chile	DALYs	Male	2014	556.0496	576.903	532.7583	

Chile	DALYs	Male	2015	556.7985	577.2368	536.9074	
Chile	DALYs	Male	2016	552.787	574.5455	532.7473	
Chile	DALYs	Male	2017	546.5896	566.757	526.5645	
Chile	DALYs	Male	2018	548.7708	570.8961	526.8338	
Chile	DALYs	Male	2019	537.6614	560.7067	514.4917	
Chile	DALYs	Male	2020	516.1327	538.4376	490.303	
Chile	DALYs	Male	2021	516.0355	539.9603	490.8328	
Chile	DALYs	Male	2022	538.9205	564.1321	509.0587	
Chile	DALYs	Male	2023	514.8017	548.3133	481.7653	
Chile	DALYs	Female	2010	305.8357	324.7061	283.7734	
Chile	DALYs	Female	2011	324.6176	342.9718	300.3255	
Chile	DALYs	Female	2012	338.1311	357.0308	315.0017	
Chile	DALYs	Female	2013	344.4834	361.7858	319.8472	
Chile	DALYs	Female	2014	346.331	365.7311	319.7179	
Chile	DALYs	Female	2015	347.177	366.3927	320.7964	
Chile	DALYs	Female	2016	348.586	366.509	321.0964	
Chile	DALYs	Female	2017	360.9473	382.6007	332.8572	
Chile	DALYs	Female	2018	358.4624	378.1673	330.3332	
Chile	DALYs	Female	2019	363.1514	386.5695	335.3339	
Chile	DALYs	Female	2020	341.5366	361.4657	317.256	
Chile	DALYs	Female	2021	349.4995	370.1065	324.9245	
Chile	DALYs	Female	2022	372.3597	396.8509	340.6826	
Chile	DALYs	Female	2023	378.7515	409.2039	342.5564	
China	Incidence	Male	2010	56.25702	66.27399	45.57473	
China	Incidence	Male	2011	57.07517	67.05843	46.4993	
China	Incidence	Male	2012	57.59378	67.24084	46.99494	
China	Incidence	Male	2013	59.51401	69.88961	48.96745	
China	Incidence	Male	2014	61.15418	72.11503	50.72481	
China	Incidence	Male	2015	64.06791	74.73417	53.43437	
China	Incidence	Male	2016	68.28045	81.58268	56.87647	
China	Incidence	Male	2017	71.81729	85.77419	59.67274	
China	Incidence	Male	2018	70.69571	84.56723	58.85263	
China	Incidence	Male	2019	67.9628	79.09161	55.63448	
China	Incidence	Male	2020	66.96221	77.95738	54.7402	
China	Incidence	Male	2021	76.20508	87.22314	63.48389	
China	Incidence	Male	2022	85.04616	102.4172	67.81736	

China	Incidence	Male	2023	81.84191	98.78017	65.6564	
China	Incidence	Female	2010	26.99987	30.48641	24.06958	
China	Incidence	Female	2011	26.95318	30.75027	23.88052	
China	Incidence	Female	2012	26.81678	30.00744	23.44281	
China	Incidence	Female	2013	27.01527	30.43709	23.56888	
China	Incidence	Female	2014	27.895	31.02749	24.49875	
China	Incidence	Female	2015	29.02811	32.53062	25.72745	
China	Incidence	Female	2016	30.76462	35.18056	26.98022	
China	Incidence	Female	2017	32.20788	36.97856	27.86631	
China	Incidence	Female	2018	31.92876	36.99068	27.05767	
China	Incidence	Female	2019	30.99565	35.67031	26.64109	
China	Incidence	Female	2020	30.80247	34.79569	26.68933	
China	Incidence	Female	2021	34.13801	39.30993	29.08033	
China	Incidence	Female	2022	37.48149	45.37045	30.70827	
China	Incidence	Female	2023	39.11187	48.00513	31.78881	
China	Deaths	Male	2010	52.45534	61.58878	42.82102	
China	Deaths	Male	2011	52.96228	62.11906	42.73616	
China	Deaths	Male	2012	53.20491	62.13771	43.45819	
China	Deaths	Male	2013	54.62342	64.03721	45.53857	
China	Deaths	Male	2014	55.85033	65.74847	46.40868	
China	Deaths	Male	2015	58.38044	67.99068	48.71242	
China	Deaths	Male	2016	62.10004	74.26451	51.32761	
China	Deaths	Male	2017	65.0313	77.01562	53.54427	
China	Deaths	Male	2018	63.69356	75.35184	52.56988	
China	Deaths	Male	2019	60.87462	69.88735	49.58578	
China	Deaths	Male	2020	59.72434	68.77923	48.08057	
China	Deaths	Male	2021	67.79016	77.45592	54.83871	
China	Deaths	Male	2022	75.26362	89.67924	59.83246	
China	Deaths	Male	2023	72.2695	87.56061	56.84338	
China	Deaths	Female	2010	24.84625	27.45581	22.12106	
China	Deaths	Female	2011	24.64122	27.83808	21.92279	
China	Deaths	Female	2012	24.39205	27.479	21.50916	
China	Deaths	Female	2013	24.38673	27.43968	21.4114	
China	Deaths	Female	2014	25.04841	27.8901	22.3121	
China	Deaths	Female	2015	26.03359	28.88771	23.19459	
China	Deaths	Female	2016	27.57287	30.97915	24.47294	

China	Deaths	Female	2017	28.74452	32.27354	25.29881	
China	Deaths	Female	2018	28.33189	32.17522	24.62805	
China	Deaths	Female	2019	27.29498	30.85817	23.55114	
China	Deaths	Female	2020	26.98215	29.83656	23.93443	
China	Deaths	Female	2021	29.84942	33.47314	26.00029	
China	Deaths	Female	2022	32.51599	37.9913	26.88914	
China	Deaths	Female	2023	33.93047	40.6435	28.43706	
China	DALYs	Male	2010	1316.651	1565.661	1078.284	
China	DALYs	Male	2011	1321.469	1563.668	1092.483	
China	DALYs	Male	2012	1319.324	1562.245	1095.529	
China	DALYs	Male	2013	1351.263	1592.46	1127.661	
China	DALYs	Male	2014	1376.189	1633.628	1153.983	
China	DALYs	Male	2015	1428.737	1673.026	1189.686	
China	DALYs	Male	2016	1504.447	1825.178	1263.267	
China	DALYs	Male	2017	1560.086	1891.859	1316.534	
China	DALYs	Male	2018	1518.741	1830.759	1278.25	
China	DALYs	Male	2019	1445.461	1690.224	1196.298	
China	DALYs	Male	2020	1407.012	1651.804	1161.161	
China	DALYs	Male	2021	1578.123	1838.588	1301.313	
China	DALYs	Male	2022	1751.979	2080.994	1401.511	
China	DALYs	Male	2023	1673.07	2009.377	1356.451	
China	DALYs	Female	2010	602.8403	664.5255	542.8745	
China	DALYs	Female	2011	592.7438	673.5794	525.8008	
China	DALYs	Female	2012	580.5053	648.5356	523.6219	
China	DALYs	Female	2013	576.6664	645.0376	512.874	
China	DALYs	Female	2014	587.1172	653.8406	528.0881	
China	DALYs	Female	2015	603.1441	663.9741	544.5123	
China	DALYs	Female	2016	628.6657	700.0848	564.2901	
China	DALYs	Female	2017	646.2659	723.8543	574.5821	
China	DALYs	Female	2018	632.4178	716.4391	558.1566	
China	DALYs	Female	2019	607.9398	689.6196	537.6968	
China	DALYs	Female	2020	596.9724	668.6049	539.3305	
China	DALYs	Female	2021	650.9703	736.7628	577.5223	
China	DALYs	Female	2022	712.5951	821.1877	609.2836	
China	DALYs	Female	2023	731.2794	895.0794	624.5277	
Colombia	Incidence	Male	2010	12.95179	13.58855	12.33039	

Colombia	Incidence	Male	2011	13.24441	14.07977	12.52447	
Colombia	Incidence	Male	2012	13.02194	13.81724	12.3381	
Colombia	Incidence	Male	2013	13.69532	14.44616	13.03276	
Colombia	Incidence	Male	2014	14.16699	14.92415	13.46658	
Colombia	Incidence	Male	2015	14.2189	15.05722	13.54119	
Colombia	Incidence	Male	2016	14.34496	15.16131	13.60013	
Colombia	Incidence	Male	2017	14.55645	15.54658	13.6978	
Colombia	Incidence	Male	2018	14.1483	15.14163	13.42237	
Colombia	Incidence	Male	2019	13.45287	14.4562	12.59078	
Colombia	Incidence	Male	2020	13.18816	14.28743	12.30617	
Colombia	Incidence	Male	2021	12.41862	13.44144	11.53951	
Colombia	Incidence	Male	2022	12.49291	13.48278	11.62589	
Colombia	Incidence	Male	2023	12.48622	13.47994	11.40337	
Colombia	Incidence	Female	2010	9.30687	10.11275	8.490216	
Colombia	Incidence	Female	2011	9.552252	10.37223	8.730986	
Colombia	Incidence	Female	2012	9.881247	10.84247	9.06772	
Colombia	Incidence	Female	2013	10.05367	10.97031	9.182502	
Colombia	Incidence	Female	2014	10.59525	11.53693	9.612001	
Colombia	Incidence	Female	2015	10.97223	11.88167	9.949346	
Colombia	Incidence	Female	2016	11.02648	11.99961	9.988355	
Colombia	Incidence	Female	2017	11.20457	12.23939	10.04535	
Colombia	Incidence	Female	2018	11.20916	12.22555	10.02015	
Colombia	Incidence	Female	2019	10.62808	11.52724	9.560788	
Colombia	Incidence	Female	2020	10.20436	11.05857	9.255786	
Colombia	Incidence	Female	2021	10.38304	11.1922	9.563276	
Colombia	Incidence	Female	2022	10.53063	11.55208	9.605214	
Colombia	Incidence	Female	2023	10.81731	11.93502	9.744567	
Colombia	Deaths	Male	2010	12.77347	13.38507	12.10563	
Colombia	Deaths	Male	2011	13.02336	13.66326	12.28033	
Colombia	Deaths	Male	2012	12.79983	13.44102	12.08866	
Colombia	Deaths	Male	2013	13.47883	14.07298	12.82614	
Colombia	Deaths	Male	2014	13.95433	14.51341	13.35245	
Colombia	Deaths	Male	2015	14.01317	14.62013	13.42006	
Colombia	Deaths	Male	2016	14.0936	14.67243	13.41859	
Colombia	Deaths	Male	2017	14.32943	15.10722	13.62141	
Colombia	Deaths	Male	2018	13.90207	14.71829	13.22173	

Colombia	Deaths	Male	2019	13.19798	14.00114	12.46957	
Colombia	Deaths	Male	2020	12.92782	13.72421	12.2257	
Colombia	Deaths	Male	2021	12.09591	12.84491	11.39862	
Colombia	Deaths	Male	2022	12.13263	12.82039	11.26794	
Colombia	Deaths	Male	2023	12.07077	12.83687	11.1722	
Colombia	Deaths	Female	2010	8.507329	9.059149	7.859329	
Colombia	Deaths	Female	2011	8.643291	9.220774	7.907374	
Colombia	Deaths	Female	2012	8.907392	9.478253	8.167007	
Colombia	Deaths	Female	2013	9.077149	9.64148	8.269404	
Colombia	Deaths	Female	2014	9.579447	10.12134	8.765954	
Colombia	Deaths	Female	2015	9.885939	10.45485	9.092216	
Colombia	Deaths	Female	2016	9.887545	10.48224	9.114856	
Colombia	Deaths	Female	2017	10.05297	10.67242	9.142973	
Colombia	Deaths	Female	2018	10.05624	10.73587	9.211084	
Colombia	Deaths	Female	2019	9.505999	10.09421	8.744035	
Colombia	Deaths	Female	2020	9.039295	9.553223	8.392065	
Colombia	Deaths	Female	2021	9.133374	9.619124	8.541914	
Colombia	Deaths	Female	2022	9.302696	9.903015	8.587085	
Colombia	Deaths	Female	2023	9.487668	10.15264	8.592214	
Colombia	DALYs	Male	2010	304.049	317.8857	289.8782	
Colombia	DALYs	Male	2011	307.6409	321.1972	291.2343	
Colombia	DALYs	Male	2012	299.5373	314.6782	284.7295	
Colombia	DALYs	Male	2013	311.0134	323.374	297.8677	
Colombia	DALYs	Male	2014	320.0581	331.8785	308.002	
Colombia	DALYs	Male	2015	318.9314	331.5469	307.6425	
Colombia	DALYs	Male	2016	321.1314	334.3645	309.2696	
Colombia	DALYs	Male	2017	322.149	338.2193	309.8576	
Colombia	DALYs	Male	2018	313.4062	330.92	299.8352	
Colombia	DALYs	Male	2019	297.8453	314.7157	281.1855	
Colombia	DALYs	Male	2020	289.1082	306.5181	273.4407	
Colombia	DALYs	Male	2021	273.3464	290.5462	257.3058	
Colombia	DALYs	Male	2022	273.7373	290.2376	255.3407	
Colombia	DALYs	Male	2023	271.7739	289.2798	254.4489	
Colombia	DALYs	Female	2010	199.3188	211.1554	186.7332	
Colombia	DALYs	Female	2011	202.4626	215.6869	187.8004	
Colombia	DALYs	Female	2012	207.7371	220.0005	192.7241	

Colombia	DALYs	Female	2013	208.7148	221.7246	193.3062	
Colombia	DALYs	Female	2014	217.8107	229.8317	202.9229	
Colombia	DALYs	Female	2015	225.2817	237.7189	211.9891	
Colombia	DALYs	Female	2016	225.0615	237.3766	210.8115	
Colombia	DALYs	Female	2017	226.257	239.1024	209.0347	
Colombia	DALYs	Female	2018	225.9634	240.2772	208.7893	
Colombia	DALYs	Female	2019	213.1306	224.9782	197.1689	
Colombia	DALYs	Female	2020	204.2236	215.5275	191.292	
Colombia	DALYs	Female	2021	205.7192	215.6055	194.9546	
Colombia	DALYs	Female	2022	204.8056	216.5216	192.5043	
Colombia	DALYs	Female	2023	207.8861	221.2296	192.8129	
Comoros	Incidence	Male	2010	1.955841	2.94688	1.026729	
Comoros	Incidence	Male	2011	2.006072	2.995428	1.073993	
Comoros	Incidence	Male	2012	1.959749	2.961475	1.046641	
Comoros	Incidence	Male	2013	1.971412	2.817053	1.054649	
Comoros	Incidence	Male	2014	2.077982	2.996963	1.083306	
Comoros	Incidence	Male	2015	2.083726	3.073501	1.074438	
Comoros	Incidence	Male	2016	2.105821	3.135884	1.092143	
Comoros	Incidence	Male	2017	2.11173	3.040957	1.079873	
Comoros	Incidence	Male	2018	2.094755	3.051822	1.069383	
Comoros	Incidence	Male	2019	2.170551	3.248718	1.149889	
Comoros	Incidence	Male	2020	2.551962	4.048053	1.25252	
Comoros	Incidence	Male	2021	3.20368	5.288754	1.5749	
Comoros	Incidence	Male	2022	2.494643	3.849344	1.303809	
Comoros	Incidence	Male	2023	2.550186	3.923746	1.308714	
Comoros	Incidence	Female	2010	0.926618	1.427146	0.584009	
Comoros	Incidence	Female	2011	0.938446	1.43276	0.569145	
Comoros	Incidence	Female	2012	0.91719	1.380134	0.550109	
Comoros	Incidence	Female	2013	0.91181	1.387027	0.547779	
Comoros	Incidence	Female	2014	0.968985	1.465906	0.586453	
Comoros	Incidence	Female	2015	0.960085	1.401846	0.58728	
Comoros	Incidence	Female	2016	0.965289	1.383031	0.587397	
Comoros	Incidence	Female	2017	0.969418	1.366263	0.588278	
Comoros	Incidence	Female	2018	0.95056	1.323748	0.575924	
Comoros	Incidence	Female	2019	0.982548	1.377768	0.60823	
Comoros	Incidence	Female	2020	1.002594	1.404936	0.621934	

Comoros	Incidence	Female	2021	1.064354	1.614183	0.593051	
Comoros	Incidence	Female	2022	1.036515	1.504147	0.627045	
Comoros	Incidence	Female	2023	1.113627	1.605448	0.682618	
Comoros	Deaths	Male	2010	1.997434	3.013191	1.049037	
Comoros	Deaths	Male	2011	2.051882	3.063412	1.096289	
Comoros	Deaths	Male	2012	2.006443	3.02815	1.067447	
Comoros	Deaths	Male	2013	2.019376	2.888002	1.085957	
Comoros	Deaths	Male	2014	2.130047	3.087087	1.114815	
Comoros	Deaths	Male	2015	2.136703	3.17307	1.106503	
Comoros	Deaths	Male	2016	2.159628	3.226644	1.107954	
Comoros	Deaths	Male	2017	2.165749	3.130067	1.109626	
Comoros	Deaths	Male	2018	2.149839	3.145477	1.096344	
Comoros	Deaths	Male	2019	2.227196	3.326455	1.16764	
Comoros	Deaths	Male	2020	2.636473	4.164305	1.302411	
Comoros	Deaths	Male	2021	3.315546	5.529016	1.65033	
Comoros	Deaths	Male	2022	2.563137	3.953858	1.313624	
Comoros	Deaths	Male	2023	2.604669	4.003701	1.311924	
Comoros	Deaths	Female	2010	0.914502	1.407206	0.576132	
Comoros	Deaths	Female	2011	0.92889	1.412143	0.566633	
Comoros	Deaths	Female	2012	0.910381	1.376283	0.547139	
Comoros	Deaths	Female	2013	0.907442	1.379374	0.547833	
Comoros	Deaths	Female	2014	0.967071	1.462999	0.584739	
Comoros	Deaths	Female	2015	0.9602	1.406975	0.584555	
Comoros	Deaths	Female	2016	0.967684	1.394718	0.586022	
Comoros	Deaths	Female	2017	0.973468	1.379888	0.590725	
Comoros	Deaths	Female	2018	0.956661	1.341557	0.582265	
Comoros	Deaths	Female	2019	0.989872	1.394395	0.614235	
Comoros	Deaths	Female	2020	1.013094	1.41761	0.63252	
Comoros	Deaths	Female	2021	1.079614	1.621801	0.602468	
Comoros	Deaths	Female	2022	1.047048	1.52444	0.629517	
Comoros	Deaths	Female	2023	1.120764	1.609465	0.683401	
Comoros	DALYs	Male	2010	52.86866	79.85386	27.98892	
Comoros	DALYs	Male	2011	54.15005	80.97078	29.27022	
Comoros	DALYs	Male	2012	52.89174	79.69152	28.13307	
Comoros	DALYs	Male	2013	53.25791	75.94623	28.37776	
Comoros	DALYs	Male	2014	56.07382	80.67724	29.20809	

Comoros	DALYs	Male	2015	56.2243	82.47136	29.85618	
Comoros	DALYs	Male	2016	56.82713	82.26632	29.84114	
Comoros	DALYs	Male	2017	56.94235	81.6692	29.61799	
Comoros	DALYs	Male	2018	56.3804	82.52592	29.4072	
Comoros	DALYs	Male	2019	58.40771	87.29554	31.65633	
Comoros	DALYs	Male	2020	67.92886	106.9168	33.7715	
Comoros	DALYs	Male	2021	84.95146	143.4507	41.8957	
Comoros	DALYs	Male	2022	66.96911	103.3585	36.45998	
Comoros	DALYs	Male	2023	69.01926	106.4091	35.96321	
Comoros	DALYs	Female	2010	26.66591	41.133	16.72709	
Comoros	DALYs	Female	2011	26.85623	40.96892	16.20436	
Comoros	DALYs	Female	2012	26.11964	39.0285	15.84384	
Comoros	DALYs	Female	2013	25.85899	39.03899	15.46377	
Comoros	DALYs	Female	2014	27.3122	41.21806	16.71738	
Comoros	DALYs	Female	2015	26.937	39.34785	16.59405	
Comoros	DALYs	Female	2016	26.94943	38.36806	16.62311	
Comoros	DALYs	Female	2017	26.92011	37.66714	16.38054	
Comoros	DALYs	Female	2018	26.23403	36.3142	15.79435	
Comoros	DALYs	Female	2019	27.01622	37.84111	16.62554	
Comoros	DALYs	Female	2020	27.39853	38.16935	17.15026	
Comoros	DALYs	Female	2021	28.79411	43.23343	16.08926	
Comoros	DALYs	Female	2022	28.23222	40.85599	17.10761	
Comoros	DALYs	Female	2023	30.57796	44.42754	18.81308	
Congo, Republi	Incidence	Male	2010	4.122197	6.034111	2.059953	
Congo, Republi	Incidence	Male	2011	4.056577	6.065537	2.057619	
Congo, Republi	Incidence	Male	2012	4.102233	6.168918	2.058289	
Congo, Republi	Incidence	Male	2013	4.114374	6.348206	2.037363	
Congo, Republi	Incidence	Male	2014	4.189567	6.553581	2.07411	
Congo, Republi	Incidence	Male	2015	4.29577	6.384128	2.122004	
Congo, Republi	Incidence	Male	2016	4.343874	6.490122	2.111292	
Congo, Republi	Incidence	Male	2017	4.383675	6.623273	2.158081	
Congo, Republi	Incidence	Male	2018	4.433192	6.677603	2.198342	
Congo, Republi	Incidence	Male	2019	4.653578	7.196816	2.29894	
Congo, Republi	Incidence	Male	2020	4.897867	7.808822	2.435378	
Congo, Republi	Incidence	Male	2021	5.08928	8.004548	2.525044	
Congo, Republi	Incidence	Male	2022	5.458692	8.332976	2.681283	

Congo, Republi	Incidence	Male	2023	5.668027	8.751168	2.702512	
Congo, Republi	Incidence	Female	2010	2.070595	3.095662	1.153089	
Congo, Republi	Incidence	Female	2011	2.088437	3.072326	1.145843	
Congo, Republi	Incidence	Female	2012	2.108024	3.105317	1.134283	
Congo, Republi	Incidence	Female	2013	2.102476	3.081124	1.138471	
Congo, Republi	Incidence	Female	2014	2.095675	3.068392	1.131925	
Congo, Republi	Incidence	Female	2015	2.129353	3.058621	1.149994	
Congo, Republi	Incidence	Female	2016	2.175656	3.057033	1.184838	
Congo, Republi	Incidence	Female	2017	2.188259	3.050304	1.181305	
Congo, Republi	Incidence	Female	2018	2.210631	3.020526	1.201497	
Congo, Republi	Incidence	Female	2019	2.313464	3.173769	1.245048	
Congo, Republi	Incidence	Female	2020	2.369611	3.261824	1.272711	
Congo, Republi	Incidence	Female	2021	2.427322	3.368398	1.323419	
Congo, Republi	Incidence	Female	2022	2.806421	3.996075	1.54105	
Congo, Republi	Incidence	Female	2023	2.974626	4.18993	1.653223	
Congo, Republi	Deaths	Male	2010	4.065519	5.946906	2.023747	
Congo, Republi	Deaths	Male	2011	4.005647	6.041191	2.021488	
Congo, Republi	Deaths	Male	2012	4.05471	6.119447	2.028442	
Congo, Republi	Deaths	Male	2013	4.066565	6.222201	2.002622	
Congo, Republi	Deaths	Male	2014	4.13928	6.501392	2.036662	
Congo, Republi	Deaths	Male	2015	4.239943	6.350889	2.080472	
Congo, Republi	Deaths	Male	2016	4.28249	6.442164	2.067534	
Congo, Republi	Deaths	Male	2017	4.318487	6.51657	2.113603	
Congo, Republi	Deaths	Male	2018	4.364233	6.570056	2.165385	
Congo, Republi	Deaths	Male	2019	4.579387	7.06744	2.269066	
Congo, Republi	Deaths	Male	2020	4.821917	7.662092	2.4107	
Congo, Republi	Deaths	Male	2021	5.006696	7.92061	2.478619	
Congo, Republi	Deaths	Male	2022	5.368291	8.137174	2.637789	
Congo, Republi	Deaths	Male	2023	5.559059	8.562431	2.636313	
Congo, Republi	Deaths	Female	2010	2.09063	3.125308	1.155739	
Congo, Republi	Deaths	Female	2011	2.107319	3.097346	1.148941	
Congo, Republi	Deaths	Female	2012	2.128018	3.14034	1.140421	
Congo, Republi	Deaths	Female	2013	2.125834	3.137831	1.142946	
Congo, Republi	Deaths	Female	2014	2.120025	3.123841	1.137198	
Congo, Republi	Deaths	Female	2015	2.153049	3.11258	1.148443	
Congo, Republi	Deaths	Female	2016	2.196037	3.09996	1.183792	

Congo, Republi	Deaths	Female	2017	2.207122	3.078468	1.180259	
Congo, Republi	Deaths	Female	2018	2.227963	3.029253	1.198636	
Congo, Republi	Deaths	Female	2019	2.327824	3.209274	1.241073	
Congo, Republi	Deaths	Female	2020	2.383405	3.263104	1.267565	
Congo, Republi	Deaths	Female	2021	2.439821	3.366605	1.323728	
Congo, Republi	Deaths	Female	2022	2.801289	3.998268	1.503859	
Congo, Republi	Deaths	Female	2023	2.956679	4.188317	1.596301	
Congo, Republi	DALYs	Male	2010	117.3449	172.1478	59.15352	
Congo, Republi	DALYs	Male	2011	115.0699	170.7379	58.60619	
Congo, Republi	DALYs	Male	2012	115.9369	172.7643	58.18098	
Congo, Republi	DALYs	Male	2013	116.0958	181.8555	57.75329	
Congo, Republi	DALYs	Male	2014	118.2674	183.995	58.91552	
Congo, Republi	DALYs	Male	2015	121.3689	177.305	60.35352	
Congo, Republi	DALYs	Male	2016	122.999	183.5526	59.91033	
Congo, Republi	DALYs	Male	2017	124.2646	188.6636	61.06315	
Congo, Republi	DALYs	Male	2018	125.7168	190.3792	62.14	
Congo, Republi	DALYs	Male	2019	131.954	204.3393	64.97028	
Congo, Republi	DALYs	Male	2020	138.2931	220.7745	68.01295	
Congo, Republi	DALYs	Male	2021	143.6376	227.0309	71.50776	
Congo, Republi	DALYs	Male	2022	154.0691	240.016	75.7847	
Congo, Republi	DALYs	Male	2023	160.6836	249.7438	76.07582	
Congo, Republi	DALYs	Female	2010	56.33712	84.34648	31.9317	
Congo, Republi	DALYs	Female	2011	56.85868	83.4969	31.82239	
Congo, Republi	DALYs	Female	2012	57.26785	83.65787	31.63294	
Congo, Republi	DALYs	Female	2013	56.83097	82.35904	31.45111	
Congo, Republi	DALYs	Female	2014	56.52356	81.50518	31.13333	
Congo, Republi	DALYs	Female	2015	57.38139	81.56761	31.56851	
Congo, Republi	DALYs	Female	2016	58.84055	83.51161	32.79698	
Congo, Republi	DALYs	Female	2017	59.27752	82.39148	32.6982	
Congo, Republi	DALYs	Female	2018	59.94784	82.1688	33.33562	
Congo, Republi	DALYs	Female	2019	62.87275	85.71029	35.14654	
Congo, Republi	DALYs	Female	2020	64.33278	89.78616	35.97725	
Congo, Republi	DALYs	Female	2021	65.90565	93.69987	37.05882	
Congo, Republi	DALYs	Female	2022	77.12471	110.6941	42.99231	
Congo, Republi	DALYs	Female	2023	82.4937	118.1962	48.48801	
Costa Rica	Incidence	Male	2010	10.49938	11.64786	9.51308	

Costa Rica	Incidence	Male	2011	11.27677	12.49287	10.20833	
Costa Rica	Incidence	Male	2012	11.1091	12.35889	10.12887	
Costa Rica	Incidence	Male	2013	10.99651	12.4094	9.96864	
Costa Rica	Incidence	Male	2014	10.74178	12.12638	9.733979	
Costa Rica	Incidence	Male	2015	10.81415	12.23974	9.770351	
Costa Rica	Incidence	Male	2016	10.61523	12.06639	9.610433	
Costa Rica	Incidence	Male	2017	10.77997	12.28369	9.67623	
Costa Rica	Incidence	Male	2018	10.89892	12.42651	9.744085	
Costa Rica	Incidence	Male	2019	10.67339	12.19243	9.501803	
Costa Rica	Incidence	Male	2020	11.14734	12.61162	9.849088	
Costa Rica	Incidence	Male	2021	10.5576	11.86362	9.309219	
Costa Rica	Incidence	Male	2022	11.55362	13.00427	10.1668	
Costa Rica	Incidence	Male	2023	13.01216	14.79514	11.22296	
Costa Rica	Incidence	Female	2010	5.812446	6.995222	4.977772	
Costa Rica	Incidence	Female	2011	6.092035	7.29362	5.23083	
Costa Rica	Incidence	Female	2012	6.203269	7.437812	5.332368	
Costa Rica	Incidence	Female	2013	6.421254	7.692977	5.529319	
Costa Rica	Incidence	Female	2014	6.594344	7.907669	5.629304	
Costa Rica	Incidence	Female	2015	6.820228	8.026103	5.807488	
Costa Rica	Incidence	Female	2016	6.809531	8.029213	5.853897	
Costa Rica	Incidence	Female	2017	6.779354	8.028553	5.763684	
Costa Rica	Incidence	Female	2018	6.975486	8.208584	5.93317	
Costa Rica	Incidence	Female	2019	6.817975	7.990933	5.777805	
Costa Rica	Incidence	Female	2020	6.542336	7.641694	5.589076	
Costa Rica	Incidence	Female	2021	7.110001	8.400127	6.102922	
Costa Rica	Incidence	Female	2022	7.994486	9.494186	6.810897	
Costa Rica	Incidence	Female	2023	8.811076	10.70825	7.491068	
Costa Rica	Deaths	Male	2010	10.12871	11.06669	9.182507	
Costa Rica	Deaths	Male	2011	10.82612	11.8692	9.885874	
Costa Rica	Deaths	Male	2012	10.64517	11.79211	9.690177	
Costa Rica	Deaths	Male	2013	10.51623	11.6254	9.549009	
Costa Rica	Deaths	Male	2014	10.29599	11.43302	9.361089	
Costa Rica	Deaths	Male	2015	10.37067	11.57152	9.405295	
Costa Rica	Deaths	Male	2016	10.20169	11.43371	9.231497	
Costa Rica	Deaths	Male	2017	10.35275	11.63248	9.354709	
Costa Rica	Deaths	Male	2018	10.46579	11.85231	9.419884	

Costa Rica	Deaths	Male	2019	10.1766	11.51592	9.139277	
Costa Rica	Deaths	Male	2020	10.60051	11.87149	9.489999	
Costa Rica	Deaths	Male	2021	10.02105	11.14037	8.896916	
Costa Rica	Deaths	Male	2022	10.9355	12.19276	9.672432	
Costa Rica	Deaths	Male	2023	12.26939	13.80255	10.72032	
Costa Rica	Deaths	Female	2010	4.998621	5.855579	4.384252	
Costa Rica	Deaths	Female	2011	5.207834	6.085274	4.534715	
Costa Rica	Deaths	Female	2012	5.276722	6.213395	4.644574	
Costa Rica	Deaths	Female	2013	5.451579	6.386907	4.788271	
Costa Rica	Deaths	Female	2014	5.609198	6.518906	4.926694	
Costa Rica	Deaths	Female	2015	5.775988	6.641961	5.064467	
Costa Rica	Deaths	Female	2016	5.756555	6.683356	5.040785	
Costa Rica	Deaths	Female	2017	5.74071	6.719902	4.99488	
Costa Rica	Deaths	Female	2018	5.906448	6.851295	5.119604	
Costa Rica	Deaths	Female	2019	5.750072	6.648052	5.04088	
Costa Rica	Deaths	Female	2020	5.467825	6.233054	4.759632	
Costa Rica	Deaths	Female	2021	5.87691	6.705178	5.093588	
Costa Rica	Deaths	Female	2022	6.587915	7.622563	5.67225	
Costa Rica	Deaths	Female	2023	7.198836	8.40316	6.193846	
Costa Rica	DALYs	Male	2010	229.8961	253.6067	208.405	
Costa Rica	DALYs	Male	2011	246.4493	271.6072	223.3135	
Costa Rica	DALYs	Male	2012	240.8372	266.5857	218.3598	
Costa Rica	DALYs	Male	2013	237.5372	263.1685	215.8985	
Costa Rica	DALYs	Male	2014	231.261	256.0997	209.8677	
Costa Rica	DALYs	Male	2015	232.9735	259.2721	211.6703	
Costa Rica	DALYs	Male	2016	227.998	256.7864	206.0618	
Costa Rica	DALYs	Male	2017	232.8783	261.951	211.7004	
Costa Rica	DALYs	Male	2018	233.3436	263.7084	208.2639	
Costa Rica	DALYs	Male	2019	230.4488	260.9332	206.6454	
Costa Rica	DALYs	Male	2020	237.8631	265.7342	213.7441	
Costa Rica	DALYs	Male	2021	224.2959	249.4283	199.825	
Costa Rica	DALYs	Male	2022	244.8052	272.2688	215.7071	
Costa Rica	DALYs	Male	2023	272.2377	307.7664	239.0027	
Costa Rica	DALYs	Female	2010	113.8886	133.5178	100.5581	
Costa Rica	DALYs	Female	2011	118.6383	140.248	104.649	
Costa Rica	DALYs	Female	2012	119.6334	140.7028	105.8996	

Costa Rica	DALYs	Female	2013	122.6731	144.9742	108.9866	
Costa Rica	DALYs	Female	2014	125.8108	147.5469	111.5832	
Costa Rica	DALYs	Female	2015	130.2529	150.761	113.9664	
Costa Rica	DALYs	Female	2016	130.6778	151.4675	114.866	
Costa Rica	DALYs	Female	2017	130.3676	152.9231	114.6982	
Costa Rica	DALYs	Female	2018	133.6215	155.8104	117.3576	
Costa Rica	DALYs	Female	2019	129.2101	149.1642	113.4603	
Costa Rica	DALYs	Female	2020	123.8033	140.6113	106.8127	
Costa Rica	DALYs	Female	2021	135.0177	153.8601	115.8576	
Costa Rica	DALYs	Female	2022	149.9508	173.5909	128.4679	
Costa Rica	DALYs	Female	2023	162.9202	190.6645	138.6911	
Croatia	Incidence	Male	2010	107.2929	113.0194	101.2098	
Croatia	Incidence	Male	2011	111.4565	118.8469	105.0929	
Croatia	Incidence	Male	2012	109.742	115.7053	103.7495	
Croatia	Incidence	Male	2013	112.0638	118.3907	105.6653	
Croatia	Incidence	Male	2014	114.3377	120.7679	107.842	
Croatia	Incidence	Male	2015	107.2303	112.7895	101.137	
Croatia	Incidence	Male	2016	110.9362	116.7527	104.4009	
Croatia	Incidence	Male	2017	113.69	119.185	108.627	
Croatia	Incidence	Male	2018	113.5241	119.1479	107.9832	
Croatia	Incidence	Male	2019	108.7982	114.565	103.465	
Croatia	Incidence	Male	2020	107.2571	112.6235	102.6113	
Croatia	Incidence	Male	2021	106.5882	111.9326	101.8378	
Croatia	Incidence	Male	2022	107.7541	113.75	101.7086	
Croatia	Incidence	Male	2023	108.959	117.1923	100.1804	
Croatia	Incidence	Female	2010	32.38156	35.15805	28.94614	
Croatia	Incidence	Female	2011	34.90556	37.9145	31.32036	
Croatia	Incidence	Female	2012	35.1141	38.15691	31.62176	
Croatia	Incidence	Female	2013	36.47649	39.42231	33.04069	
Croatia	Incidence	Female	2014	37.95504	40.96383	34.62471	
Croatia	Incidence	Female	2015	36.53554	39.54662	33.15109	
Croatia	Incidence	Female	2016	38.92463	41.85881	35.60528	
Croatia	Incidence	Female	2017	42.07785	45.37117	38.50407	
Croatia	Incidence	Female	2018	43.97501	47.27282	40.11816	
Croatia	Incidence	Female	2019	45.6595	49.76643	41.59662	
Croatia	Incidence	Female	2020	47.16866	51.58584	42.78146	

Croatia	Incidence	Female	2021	47.85792	52.65888	43.14812	
Croatia	Incidence	Female	2022	49.37743	55.13752	43.42783	
Croatia	Incidence	Female	2023	47.32601	53.12461	40.43926	
Croatia	Deaths	Male	2010	104.1273	109.7634	99.11867	
Croatia	Deaths	Male	2011	108.1863	114.8757	102.6287	
Croatia	Deaths	Male	2012	106.6879	112.3524	101.6456	
Croatia	Deaths	Male	2013	108.8306	114.6232	103.5945	
Croatia	Deaths	Male	2014	111.1328	116.7508	105.3208	
Croatia	Deaths	Male	2015	104.3546	109.2434	98.89062	
Croatia	Deaths	Male	2016	108.0374	113.185	102.583	
Croatia	Deaths	Male	2017	110.7564	114.9659	106.2113	
Croatia	Deaths	Male	2018	110.7046	115.3779	105.2614	
Croatia	Deaths	Male	2019	106.0693	110.291	101.7211	
Croatia	Deaths	Male	2020	104.2616	107.6332	100.1297	
Croatia	Deaths	Male	2021	103.6727	107.7136	99.53084	
Croatia	Deaths	Male	2022	104.6121	109.6535	99.92058	
Croatia	Deaths	Male	2023	105.6952	113.6129	98.17153	
Croatia	Deaths	Female	2010	31.77608	34.24009	28.52853	
Croatia	Deaths	Female	2011	34.25473	37.00522	30.78263	
Croatia	Deaths	Female	2012	34.40844	37.06724	31.04367	
Croatia	Deaths	Female	2013	35.60526	38.18242	32.26285	
Croatia	Deaths	Female	2014	36.99774	39.92494	33.67328	
Croatia	Deaths	Female	2015	35.67352	38.17012	32.45404	
Croatia	Deaths	Female	2016	38.06664	40.76364	35.05919	
Croatia	Deaths	Female	2017	41.08929	43.93263	37.83832	
Croatia	Deaths	Female	2018	42.75255	45.79112	39.39144	
Croatia	Deaths	Female	2019	44.34527	47.96747	40.71465	
Croatia	Deaths	Female	2020	45.7918	49.59651	41.79557	
Croatia	Deaths	Female	2021	46.45018	50.67605	42.28489	
Croatia	Deaths	Female	2022	47.82932	53.04195	42.90897	
Croatia	Deaths	Female	2023	45.77553	51.28643	39.57409	
Croatia	DALYs	Male	2010	2572.187	2708.72	2436.678	
Croatia	DALYs	Male	2011	2649.72	2807.448	2513.133	
Croatia	DALYs	Male	2012	2580.24	2725.343	2448.934	
Croatia	DALYs	Male	2013	2627.424	2778.53	2489.071	
Croatia	DALYs	Male	2014	2665.064	2807.235	2529.802	

Croatia	DALYs	Male	2015	2488.495	2606.706	2363.674	
Croatia	DALYs	Male	2016	2555.265	2678.867	2436.597	
Croatia	DALYs	Male	2017	2599.122	2699.265	2494.658	
Croatia	DALYs	Male	2018	2568.967	2693.394	2448.481	
Croatia	DALYs	Male	2019	2432.414	2534.82	2335.859	
Croatia	DALYs	Male	2020	2385.323	2478.972	2288.409	
Croatia	DALYs	Male	2021	2343.6	2440.935	2247.797	
Croatia	DALYs	Male	2022	2368.716	2481.183	2251.429	
Croatia	DALYs	Male	2023	2384.406	2567.063	2202.516	
Croatia	DALYs	Female	2010	738.6816	800.5466	665.105	
Croatia	DALYs	Female	2011	789.3458	852.5876	710.733	
Croatia	DALYs	Female	2012	790.6557	848.7572	714.6762	
Croatia	DALYs	Female	2013	820.8001	879.5236	746.4968	
Croatia	DALYs	Female	2014	852.7957	916.3777	779.9131	
Croatia	DALYs	Female	2015	818.3516	875.9311	746.9734	
Croatia	DALYs	Female	2016	861.122	917.8777	795.0624	
Croatia	DALYs	Female	2017	926.5435	991.97	853.41	
Croatia	DALYs	Female	2018	965.0232	1033.382	894.9558	
Croatia	DALYs	Female	2019	991.8596	1067.439	922.7089	
Croatia	DALYs	Female	2020	1016.393	1097.659	932.4934	
Croatia	DALYs	Female	2021	1021.303	1109.285	925.4765	
Croatia	DALYs	Female	2022	1051.765	1162.809	941.2717	
Croatia	DALYs	Female	2023	1003.864	1119.291	871.6298	
Cuba	Incidence	Male	2010	61.63135	65.87191	57.67203	
Cuba	Incidence	Male	2011	62.46071	66.73536	58.46327	
Cuba	Incidence	Male	2012	62.72458	67.34873	58.56649	
Cuba	Incidence	Male	2013	63.99593	69.00974	59.69794	
Cuba	Incidence	Male	2014	66.55016	71.09425	62.59475	
Cuba	Incidence	Male	2015	67.60087	72.45366	63.01765	
Cuba	Incidence	Male	2016	67.9188	73.22206	63.72569	
Cuba	Incidence	Male	2017	68.8676	73.75156	63.90205	
Cuba	Incidence	Male	2018	69.27147	74.89374	64.4256	
Cuba	Incidence	Male	2019	67.25157	73.11187	62.12953	
Cuba	Incidence	Male	2020	67.78894	73.53747	62.95444	
Cuba	Incidence	Male	2021	71.3525	77.45795	65.7698	
Cuba	Incidence	Male	2022	58.91089	68.56781	50.59279	

Cuba	Incidence	Male	2023	61.71135	71.93783	52.88127	
Cuba	Incidence	Female	2010	36.59449	40.04664	33.7576	
Cuba	Incidence	Female	2011	37.08028	40.67249	34.26318	
Cuba	Incidence	Female	2012	37.66724	41.18922	34.58253	
Cuba	Incidence	Female	2013	39.00942	42.59805	35.93643	
Cuba	Incidence	Female	2014	40.5601	43.92673	37.31772	
Cuba	Incidence	Female	2015	40.5409	43.80153	37.2968	
Cuba	Incidence	Female	2016	42.0078	45.43473	38.28561	
Cuba	Incidence	Female	2017	42.99727	46.10252	39.39567	
Cuba	Incidence	Female	2018	43.73248	46.93053	40.15841	
Cuba	Incidence	Female	2019	44.60673	47.85018	40.92914	
Cuba	Incidence	Female	2020	45.21932	48.54632	41.43336	
Cuba	Incidence	Female	2021	49.81529	53.48604	46.25689	
Cuba	Incidence	Female	2022	42.00457	47.41314	36.85107	
Cuba	Incidence	Female	2023	44.90697	51.24431	38.22952	
Cuba	Deaths	Male	2010	59.01775	62.32659	55.79296	
Cuba	Deaths	Male	2011	59.59567	62.73251	56.34926	
Cuba	Deaths	Male	2012	59.93044	63.31939	56.66568	
Cuba	Deaths	Male	2013	61.23493	64.83187	57.99136	
Cuba	Deaths	Male	2014	63.89588	67.30321	60.65557	
Cuba	Deaths	Male	2015	64.9681	69.12351	61.22715	
Cuba	Deaths	Male	2016	65.18639	69.08489	61.42068	
Cuba	Deaths	Male	2017	66.38097	70.32348	62.81545	
Cuba	Deaths	Male	2018	66.57136	70.96622	62.82761	
Cuba	Deaths	Male	2019	64.43328	68.88764	60.68694	
Cuba	Deaths	Male	2020	64.73429	69.84657	60.90211	
Cuba	Deaths	Male	2021	67.99437	73.95954	63.94614	
Cuba	Deaths	Male	2022	55.89891	64.79048	48.66213	
Cuba	Deaths	Male	2023	58.3488	67.64541	50.37972	
Cuba	Deaths	Female	2010	33.10166	35.28808	30.76864	
Cuba	Deaths	Female	2011	33.49472	35.89278	31.09183	
Cuba	Deaths	Female	2012	34.005	36.13236	31.51482	
Cuba	Deaths	Female	2013	35.24092	37.48896	32.89608	
Cuba	Deaths	Female	2014	36.77841	38.86382	34.62009	
Cuba	Deaths	Female	2015	36.87844	38.93926	34.67521	
Cuba	Deaths	Female	2016	38.24674	40.1948	35.6544	

Cuba	Deaths	Female	2017	39.21683	40.97482	36.83717	
Cuba	Deaths	Female	2018	39.77658	41.63434	37.2594	
Cuba	Deaths	Female	2019	40.51267	42.66492	38.06247	
Cuba	Deaths	Female	2020	40.82148	42.63769	38.54323	
Cuba	Deaths	Female	2021	44.60763	46.27663	42.66456	
Cuba	Deaths	Female	2022	37.65405	42.6424	33.72683	
Cuba	Deaths	Female	2023	40.0194	45.70259	34.73857	
Cuba	DALYs	Male	2010	1363.754	1434.992	1287.114	
Cuba	DALYs	Male	2011	1382.619	1454.184	1307.661	
Cuba	DALYs	Male	2012	1375.432	1452.969	1295.21	
Cuba	DALYs	Male	2013	1393.235	1477.329	1314.859	
Cuba	DALYs	Male	2014	1448.686	1531.012	1377.081	
Cuba	DALYs	Male	2015	1468.488	1553.916	1389.746	
Cuba	DALYs	Male	2016	1471.559	1551.436	1391.383	
Cuba	DALYs	Male	2017	1474.332	1558.83	1394.085	
Cuba	DALYs	Male	2018	1483.977	1585.016	1402.27	
Cuba	DALYs	Male	2019	1439.166	1536.161	1352.236	
Cuba	DALYs	Male	2020	1440.647	1555.406	1351.674	
Cuba	DALYs	Male	2021	1494.218	1627.398	1405.39	
Cuba	DALYs	Male	2022	1236.677	1428.307	1068.46	
Cuba	DALYs	Male	2023	1289.966	1484.62	1114.051	
Cuba	DALYs	Female	2010	786.8619	839.0871	734.5344	
Cuba	DALYs	Female	2011	790.3408	847.1194	741.4313	
Cuba	DALYs	Female	2012	796.2383	844.0298	746.8902	
Cuba	DALYs	Female	2013	819.8187	876.8567	764.0479	
Cuba	DALYs	Female	2014	850.981	906.377	797.0743	
Cuba	DALYs	Female	2015	845.7398	898.0691	788.3688	
Cuba	DALYs	Female	2016	867.2735	921.9843	816.8922	
Cuba	DALYs	Female	2017	879.8959	927.2575	831.5404	
Cuba	DALYs	Female	2018	891.7598	936.7845	843.7854	
Cuba	DALYs	Female	2019	902.8009	946.5806	855.2197	
Cuba	DALYs	Female	2020	906.3458	945.4861	862.484	
Cuba	DALYs	Female	2021	986.2752	1025.432	943.3061	
Cuba	DALYs	Female	2022	817.7576	928.7105	727.5736	
Cuba	DALYs	Female	2023	869.8974	1001.279	762.1998	
Cyprus	Incidence	Male	2010	56.19549	69.02529	46.21861	

Cyprus	Incidence	Male	2011	56.26099	69.45428	46.27853	
Cyprus	Incidence	Male	2012	61.15812	72.89214	49.54006	
Cyprus	Incidence	Male	2013	63.62749	74.64346	49.47802	
Cyprus	Incidence	Male	2014	65.37282	78.70409	50.28735	
Cyprus	Incidence	Male	2015	59.83967	70.66264	46.60599	
Cyprus	Incidence	Male	2016	59.27271	72.1216	46.93091	
Cyprus	Incidence	Male	2017	63.32028	76.90914	46.29172	
Cyprus	Incidence	Male	2018	63.38836	77.91782	49.01916	
Cyprus	Incidence	Male	2019	65.69683	84.13156	52.08639	
Cyprus	Incidence	Male	2020	64.68955	82.78167	48.71737	
Cyprus	Incidence	Male	2021	66.28239	83.34067	50.00042	
Cyprus	Incidence	Male	2022	65.53961	85.93162	48.72113	
Cyprus	Incidence	Male	2023	61.46027	80.43368	44.87412	
Cyprus	Incidence	Female	2010	15.95947	21.26061	12.06243	
Cyprus	Incidence	Female	2011	16.72791	22.1364	12.66396	
Cyprus	Incidence	Female	2012	17.21906	22.41266	12.82978	
Cyprus	Incidence	Female	2013	17.37654	23.35377	12.78092	
Cyprus	Incidence	Female	2014	17.97454	23.51586	13.43918	
Cyprus	Incidence	Female	2015	18.47325	23.72903	13.8725	
Cyprus	Incidence	Female	2016	17.30106	22.15838	13.27453	
Cyprus	Incidence	Female	2017	18.56204	23.84281	14.25839	
Cyprus	Incidence	Female	2018	18.65412	23.39904	14.05834	
Cyprus	Incidence	Female	2019	19.19049	24.18147	14.44851	
Cyprus	Incidence	Female	2020	19.72011	25.0106	14.85388	
Cyprus	Incidence	Female	2021	20.86962	26.20773	15.90138	
Cyprus	Incidence	Female	2022	20.77512	26.17354	15.49779	
Cyprus	Incidence	Female	2023	19.04272	24.86863	14.10571	
Cyprus	Deaths	Male	2010	51.97983	63.98033	42.74215	
Cyprus	Deaths	Male	2011	51.99958	63.23463	42.53897	
Cyprus	Deaths	Male	2012	56.12077	66.46901	44.98446	
Cyprus	Deaths	Male	2013	57.99513	68.88436	45.06985	
Cyprus	Deaths	Male	2014	59.35765	71.39758	45.58188	
Cyprus	Deaths	Male	2015	54.52356	64.82362	41.31337	
Cyprus	Deaths	Male	2016	53.9601	64.22835	41.08076	
Cyprus	Deaths	Male	2017	57.67988	69.56674	40.92855	
Cyprus	Deaths	Male	2018	57.50478	69.67936	44.02539	

Cyprus	Deaths	Male	2019	59.78925	75.46047	46.7778	
Cyprus	Deaths	Male	2020	58.52724	74.32192	43.57647	
Cyprus	Deaths	Male	2021	59.88129	74.82621	44.35161	
Cyprus	Deaths	Male	2022	59.27927	77.43277	44.12987	
Cyprus	Deaths	Male	2023	55.65885	72.39748	39.40739	
Cyprus	Deaths	Female	2010	14.53376	19.14293	11.17372	
Cyprus	Deaths	Female	2011	15.26389	19.99352	11.57798	
Cyprus	Deaths	Female	2012	15.62806	20.3248	11.65186	
Cyprus	Deaths	Female	2013	15.41819	20.25552	11.31757	
Cyprus	Deaths	Female	2014	16.06853	21.07218	12.10033	
Cyprus	Deaths	Female	2015	16.48988	20.9104	12.46112	
Cyprus	Deaths	Female	2016	15.34377	19.46716	11.75045	
Cyprus	Deaths	Female	2017	16.59103	21.34662	12.74252	
Cyprus	Deaths	Female	2018	16.54844	20.89177	12.50437	
Cyprus	Deaths	Female	2019	17.08832	21.98935	13.05293	
Cyprus	Deaths	Female	2020	17.38651	21.81208	13.32393	
Cyprus	Deaths	Female	2021	18.24693	23.62554	13.9277	
Cyprus	Deaths	Female	2022	18.20316	23.00205	13.75504	
Cyprus	Deaths	Female	2023	16.70977	22.05785	12.57789	
Cyprus	DALYs	Male	2010	1187.939	1476.545	989.4949	
Cyprus	DALYs	Male	2011	1174.863	1434.72	978.9641	
Cyprus	DALYs	Male	2012	1266.123	1518.992	1033.543	
Cyprus	DALYs	Male	2013	1306.638	1551.857	1031.365	
Cyprus	DALYs	Male	2014	1336.415	1574.452	1058.599	
Cyprus	DALYs	Male	2015	1210.955	1436.635	967.9953	
Cyprus	DALYs	Male	2016	1195.083	1409.889	970.442	
Cyprus	DALYs	Male	2017	1268.03	1511.985	940.9387	
Cyprus	DALYs	Male	2018	1271.529	1539.382	1017.887	
Cyprus	DALYs	Male	2019	1302.703	1628.496	1058.975	
Cyprus	DALYs	Male	2020	1275.663	1601.941	997.8409	
Cyprus	DALYs	Male	2021	1286.702	1599.604	999.9485	
Cyprus	DALYs	Male	2022	1259.092	1621.291	979.7838	
Cyprus	DALYs	Male	2023	1176.975	1521.962	859.2653	
Cyprus	DALYs	Female	2010	320.3897	423.7201	244.0878	
Cyprus	DALYs	Female	2011	328.977	428.7604	251.7654	
Cyprus	DALYs	Female	2012	335.422	429.8596	254.4925	

Cyprus	DALYs	Female	2013	341.4642	441.0343	254.6513	
Cyprus	DALYs	Female	2014	347.5814	449.4091	260.6274	
Cyprus	DALYs	Female	2015	354.0084	456.0729	271.9405	
Cyprus	DALYs	Female	2016	335.6986	424.1771	264.1363	
Cyprus	DALYs	Female	2017	354.4768	452.0186	273.8527	
Cyprus	DALYs	Female	2018	356.6263	450.7996	268.2715	
Cyprus	DALYs	Female	2019	362.9469	471.4367	280.1002	
Cyprus	DALYs	Female	2020	370.4727	457.4465	283.2518	
Cyprus	DALYs	Female	2021	390.8561	509.1449	296.8271	
Cyprus	DALYs	Female	2022	384.1485	484.5687	290.2385	
Cyprus	DALYs	Female	2023	350.4335	462.6422	262.249	
Czechia	Incidence	Male	2010	97.09794	101.9514	92.17183	
Czechia	Incidence	Male	2011	95.32504	100.6585	89.77913	
Czechia	Incidence	Male	2012	92.54768	97.67871	87.40837	
Czechia	Incidence	Male	2013	90.2611	95.13664	84.92345	
Czechia	Incidence	Male	2014	89.27174	94.62014	83.42283	
Czechia	Incidence	Male	2015	85.87514	91.16724	80.71058	
Czechia	Incidence	Male	2016	87.17183	92.74078	82.40799	
Czechia	Incidence	Male	2017	87.13455	93.1758	81.46258	
Czechia	Incidence	Male	2018	85.44764	91.50817	79.8302	
Czechia	Incidence	Male	2019	84.38655	89.45287	78.44375	
Czechia	Incidence	Male	2020	84.10453	89.77162	78.94673	
Czechia	Incidence	Male	2021	79.262	84.35331	74.28447	
Czechia	Incidence	Male	2022	77.62993	83.42646	72.6573	
Czechia	Incidence	Male	2023	74.11118	80.40134	68.52844	
Czechia	Incidence	Female	2010	39.05223	43.01735	35.75361	
Czechia	Incidence	Female	2011	40.46713	44.96983	36.61287	
Czechia	Incidence	Female	2012	41.58008	46.43095	37.32671	
Czechia	Incidence	Female	2013	41.36226	45.90783	37.2057	
Czechia	Incidence	Female	2014	41.61862	46.22325	37.39678	
Czechia	Incidence	Female	2015	41.40157	45.47946	37.6264	
Czechia	Incidence	Female	2016	42.06117	45.79773	38.02794	
Czechia	Incidence	Female	2017	43.96086	47.79221	39.77085	
Czechia	Incidence	Female	2018	44.63137	48.77632	40.45818	
Czechia	Incidence	Female	2019	44.98884	49.1843	40.60314	
Czechia	Incidence	Female	2020	45.96663	50.24527	41.83681	

Czechia	Incidence	Female	2021	45.08999	49.27132	40.39999	
Czechia	Incidence	Female	2022	44.41838	48.35494	39.36608	
Czechia	Incidence	Female	2023	41.97361	46.37879	36.57627	
Czechia	Deaths	Male	2010	86.57151	90.12209	82.51499	
Czechia	Deaths	Male	2011	85.11816	88.98386	81.37084	
Czechia	Deaths	Male	2012	82.71092	86.44686	78.50796	
Czechia	Deaths	Male	2013	80.82411	85.00464	76.12729	
Czechia	Deaths	Male	2014	80.13748	84.04804	75.96002	
Czechia	Deaths	Male	2015	77.42813	81.56241	73.55299	
Czechia	Deaths	Male	2016	78.77215	82.76615	75.04015	
Czechia	Deaths	Male	2017	78.95732	82.82859	75.09495	
Czechia	Deaths	Male	2018	77.49059	81.69877	73.33432	
Czechia	Deaths	Male	2019	76.63294	80.606	72.1481	
Czechia	Deaths	Male	2020	76.37979	80.25392	72.73537	
Czechia	Deaths	Male	2021	71.82026	76.02615	68.72115	
Czechia	Deaths	Male	2022	70.43431	75.28064	66.53474	
Czechia	Deaths	Male	2023	67.30927	72.46394	63.36868	
Czechia	Deaths	Female	2010	33.61387	36.32903	31.18716	
Czechia	Deaths	Female	2011	34.83322	37.67242	31.99412	
Czechia	Deaths	Female	2012	35.82489	38.88528	32.85015	
Czechia	Deaths	Female	2013	35.66168	38.7877	32.74434	
Czechia	Deaths	Female	2014	35.95995	38.73674	32.75603	
Czechia	Deaths	Female	2015	35.94013	38.26467	32.80763	
Czechia	Deaths	Female	2016	36.66491	38.82564	33.39949	
Czechia	Deaths	Female	2017	38.39029	40.6066	35.28864	
Czechia	Deaths	Female	2018	39.08636	41.58983	36.2151	
Czechia	Deaths	Female	2019	39.47972	42.05699	36.67562	
Czechia	Deaths	Female	2020	40.35617	42.6857	37.56685	
Czechia	Deaths	Female	2021	39.4706	41.54957	36.59061	
Czechia	Deaths	Female	2022	38.97679	41.22648	35.76367	
Czechia	Deaths	Female	2023	36.82706	39.4878	33.03721	
Czechia	DALYs	Male	2010	2106.712	2177.928	2018.591	
Czechia	DALYs	Male	2011	2047.4	2131.603	1968.292	
Czechia	DALYs	Male	2012	1974.835	2058.249	1879.798	
Czechia	DALYs	Male	2013	1905.163	1990.135	1803.468	
Czechia	DALYs	Male	2014	1870.564	1952.629	1777.739	

Czechia	DALYs	Male	2015	1781.387	1868.683	1696.609	
Czechia	DALYs	Male	2016	1798.604	1885.563	1721.483	
Czechia	DALYs	Male	2017	1777.325	1860.244	1695.343	
Czechia	DALYs	Male	2018	1727.084	1814.197	1642.119	
Czechia	DALYs	Male	2019	1683.04	1761.026	1591.711	
Czechia	DALYs	Male	2020	1655.507	1731.759	1586.689	
Czechia	DALYs	Male	2021	1561.727	1631.771	1501.143	
Czechia	DALYs	Male	2022	1518.016	1602.443	1444.052	
Czechia	DALYs	Male	2023	1442.346	1533.94	1362.679	
Czechia	DALYs	Female	2010	774.2367	833.4003	721.1279	
Czechia	DALYs	Female	2011	797.8125	857.3106	737.9856	
Czechia	DALYs	Female	2012	814.993	886.2662	752.4131	
Czechia	DALYs	Female	2013	806.8125	873.8772	742.3754	
Czechia	DALYs	Female	2014	808.2937	867.9765	742.752	
Czechia	DALYs	Female	2015	802.0193	851.14	739.2127	
Czechia	DALYs	Female	2016	808.2872	854.0541	742.8747	
Czechia	DALYs	Female	2017	839.5833	886.2852	774.7371	
Czechia	DALYs	Female	2018	842.8313	892.0981	781.8287	
Czechia	DALYs	Female	2019	836.3919	887.4079	775.3052	
Czechia	DALYs	Female	2020	842.767	893.3698	784.1932	
Czechia	DALYs	Female	2021	824.86	870.8623	768.0074	
Czechia	DALYs	Female	2022	805.3396	853.6875	738.9285	
Czechia	DALYs	Female	2023	758.3432	816.0556	681.0773	
Korea, Democr	Incidence	Male	2010	26.46809	38.11045	18.38252	
Korea, Democr	Incidence	Male	2011	27.00785	38.58139	19.14863	
Korea, Democr	Incidence	Male	2012	27.51203	39.9792	19.89943	
Korea, Democr	Incidence	Male	2013	28.48892	41.7938	20.68408	
Korea, Democr	Incidence	Male	2014	28.90321	41.42937	20.80735	
Korea, Democr	Incidence	Male	2015	29.44409	42.57069	20.5687	
Korea, Democr	Incidence	Male	2016	30.05047	44.50871	20.48386	
Korea, Democr	Incidence	Male	2017	31.01271	47.22571	20.81663	
Korea, Democr	Incidence	Male	2018	31.74847	48.48337	21.42476	
Korea, Democr	Incidence	Male	2019	32.68473	50.51234	21.3515	
Korea, Democr	Incidence	Male	2020	33.62003	51.41335	21.66555	
Korea, Democr	Incidence	Male	2021	35.27772	54.76349	22.6253	
Korea, Democr	Incidence	Male	2022	37.5555	58.29978	24.66665	

Korea, Democr	Incidence	Male	2023	38.94527	60.1455	25.79067	
Korea, Democr	Incidence	Female	2010	18.65732	26.88603	12.20425	
Korea, Democr	Incidence	Female	2011	19.30635	28.63999	12.57796	
Korea, Democr	Incidence	Female	2012	20.04466	30.88901	12.65325	
Korea, Democr	Incidence	Female	2013	20.66079	31.94693	13.29438	
Korea, Democr	Incidence	Female	2014	21.13722	32.06574	13.27199	
Korea, Democr	Incidence	Female	2015	21.69391	31.26995	13.64094	
Korea, Democr	Incidence	Female	2016	22.4395	31.79111	14.50164	
Korea, Democr	Incidence	Female	2017	23.3026	33.00716	15.20411	
Korea, Democr	Incidence	Female	2018	24.17498	34.76368	15.92703	
Korea, Democr	Incidence	Female	2019	25.09587	35.5867	16.61986	
Korea, Democr	Incidence	Female	2020	25.66255	35.9402	17.18098	
Korea, Democr	Incidence	Female	2021	26.79785	36.82309	17.82094	
Korea, Democr	Incidence	Female	2022	28.60821	39.83491	18.58369	
Korea, Democr	Incidence	Female	2023	29.29943	42.74202	18.60712	
Korea, Democr	Deaths	Male	2010	25.56197	36.84554	17.72804	
Korea, Democr	Deaths	Male	2011	26.10996	37.08873	18.48851	
Korea, Democr	Deaths	Male	2012	26.6295	38.90584	19.16027	
Korea, Democr	Deaths	Male	2013	27.59394	40.63803	19.89444	
Korea, Democr	Deaths	Male	2014	28.00509	39.94188	20.02881	
Korea, Democr	Deaths	Male	2015	28.53593	41.16743	19.94335	
Korea, Democr	Deaths	Male	2016	29.1284	43.1329	19.95027	
Korea, Democr	Deaths	Male	2017	30.06464	45.3676	20.34267	
Korea, Democr	Deaths	Male	2018	30.78845	46.94847	20.72626	
Korea, Democr	Deaths	Male	2019	31.68798	48.76325	20.73469	
Korea, Democr	Deaths	Male	2020	32.61483	50.04382	21.02954	
Korea, Democr	Deaths	Male	2021	34.21677	52.64533	21.98921	
Korea, Democr	Deaths	Male	2022	36.43042	56.22055	23.97088	
Korea, Democr	Deaths	Male	2023	37.7953	59.10672	25.05128	
Korea, Democr	Deaths	Female	2010	18.47951	26.8812	12.17092	
Korea, Democr	Deaths	Female	2011	19.12981	28.56307	12.39731	
Korea, Democr	Deaths	Female	2012	19.86836	30.46244	12.68388	
Korea, Democr	Deaths	Female	2013	20.47348	31.99641	13.33693	
Korea, Democr	Deaths	Female	2014	20.94781	31.9207	13.24663	
Korea, Democr	Deaths	Female	2015	21.49365	31.04893	13.59105	
Korea, Democr	Deaths	Female	2016	22.21431	31.38061	14.20629	

Korea, Democr	Deaths	Female	2017	23.04346	32.76492	14.94263	
Korea, Democr	Deaths	Female	2018	23.8834	34.53107	15.62061	
Korea, Democr	Deaths	Female	2019	24.75431	35.36647	16.3197	
Korea, Democr	Deaths	Female	2020	25.28444	35.42939	16.79642	
Korea, Democr	Deaths	Female	2021	26.35967	36.46646	17.7858	
Korea, Democr	Deaths	Female	2022	28.0863	39.24213	18.31152	
Korea, Democr	Deaths	Female	2023	28.73491	41.47134	18.58255	
Korea, Democr	DALYs	Male	2010	724.16	1051.908	503.2906	
Korea, Democr	DALYs	Male	2011	736.9317	1045.45	527.0537	
Korea, Democr	DALYs	Male	2012	748.3986	1089.299	539.5665	
Korea, Democr	DALYs	Male	2013	773.3674	1132.767	559.6644	
Korea, Democr	DALYs	Male	2014	782.8478	1123.734	559.4143	
Korea, Democr	DALYs	Male	2015	795.085	1170.574	557.1071	
Korea, Democr	DALYs	Male	2016	808.7761	1212.969	549.578	
Korea, Democr	DALYs	Male	2017	832.193	1275.873	567.3845	
Korea, Democr	DALYs	Male	2018	849.2098	1320.676	569.9198	
Korea, Democr	DALYs	Male	2019	871.3652	1353.459	565.0049	
Korea, Democr	DALYs	Male	2020	892.1133	1371.29	571.3452	
Korea, Democr	DALYs	Male	2021	933.6554	1479.255	605.6457	
Korea, Democr	DALYs	Male	2022	992.5096	1541.73	645.9961	
Korea, Democr	DALYs	Male	2023	1027.989	1570.003	678.1488	
Korea, Democr	DALYs	Female	2010	452.9325	659.6941	296.5545	
Korea, Democr	DALYs	Female	2011	467.3445	682.1899	305.5029	
Korea, Democr	DALYs	Female	2012	483.3851	740.6443	308.0127	
Korea, Democr	DALYs	Female	2013	496.757	760.9289	320.5286	
Korea, Democr	DALYs	Female	2014	506.7161	766.7142	323.6512	
Korea, Democr	DALYs	Female	2015	518.3253	746.9913	331.6875	
Korea, Democr	DALYs	Female	2016	533.9683	751.8235	346.8478	
Korea, Democr	DALYs	Female	2017	552.735	770.3166	364.1269	
Korea, Democr	DALYs	Female	2018	571.6911	821.9044	378.7587	
Korea, Democr	DALYs	Female	2019	592.0284	829.7533	398.5449	
Korea, Democr	DALYs	Female	2020	603.435	841.5097	407.757	
Korea, Democr	DALYs	Female	2021	628.7394	857.5718	421.2538	
Korea, Democr	DALYs	Female	2022	672.4892	929.2476	442.5102	
Korea, Democr	DALYs	Female	2023	689.9152	992.565	444.8091	
Congo, Democr	Incidence	Male	2010	2.877622	5.061097	1.358563	

Congo, Democr	Incidence	Male	2011	2.917601	5.078849	1.413759	
Congo, Democr	Incidence	Male	2012	2.962092	5.1149	1.417869	
Congo, Democr	Incidence	Male	2013	3.031717	5.21755	1.473159	
Congo, Democr	Incidence	Male	2014	3.134828	5.292168	1.508305	
Congo, Democr	Incidence	Male	2015	3.201023	5.406178	1.541347	
Congo, Democr	Incidence	Male	2016	3.256637	5.576686	1.539055	
Congo, Democr	Incidence	Male	2017	3.322903	5.749598	1.558884	
Congo, Democr	Incidence	Male	2018	3.422616	5.966088	1.605361	
Congo, Democr	Incidence	Male	2019	3.53412	6.305778	1.651959	
Congo, Democr	Incidence	Male	2020	3.914768	6.752033	1.832521	
Congo, Democr	Incidence	Male	2021	3.971365	7.10024	1.913755	
Congo, Democr	Incidence	Male	2022	4.370316	8.030754	1.979118	
Congo, Democr	Incidence	Male	2023	4.470592	7.878485	1.922037	
Congo, Democr	Incidence	Female	2010	1.667129	3.211718	0.869458	
Congo, Democr	Incidence	Female	2011	1.704911	3.328868	0.892288	
Congo, Democr	Incidence	Female	2012	1.747482	3.354645	0.934415	
Congo, Democr	Incidence	Female	2013	1.800696	3.448059	0.979094	
Congo, Democr	Incidence	Female	2014	1.873511	3.557612	1.03387	
Congo, Democr	Incidence	Female	2015	1.932075	3.491551	1.031832	
Congo, Democr	Incidence	Female	2016	2.001238	3.463622	1.074489	
Congo, Democr	Incidence	Female	2017	2.082413	3.42579	1.116807	
Congo, Democr	Incidence	Female	2018	2.192419	3.534609	1.170519	
Congo, Democr	Incidence	Female	2019	2.305105	3.7821	1.266254	
Congo, Democr	Incidence	Female	2020	2.433307	4.106855	1.365725	
Congo, Democr	Incidence	Female	2021	2.522505	4.170026	1.398639	
Congo, Democr	Incidence	Female	2022	3.003671	4.905177	1.64716	
Congo, Democr	Incidence	Female	2023	3.13468	5.140112	1.728155	
Congo, Democr	Deaths	Male	2010	2.838255	4.94493	1.334318	
Congo, Democr	Deaths	Male	2011	2.87497	4.972136	1.390266	
Congo, Democr	Deaths	Male	2012	2.916194	5.004821	1.395933	
Congo, Democr	Deaths	Male	2013	2.980479	5.106602	1.436796	
Congo, Democr	Deaths	Male	2014	3.07763	5.182062	1.474086	
Congo, Democr	Deaths	Male	2015	3.140053	5.289542	1.508198	
Congo, Democr	Deaths	Male	2016	3.192735	5.469798	1.504656	
Congo, Democr	Deaths	Male	2017	3.256954	5.646397	1.520306	
Congo, Democr	Deaths	Male	2018	3.354521	5.844687	1.570047	

Congo, Democr	Deaths	Male	2019	3.464181	6.170331	1.614497	
Congo, Democr	Deaths	Male	2020	3.864262	6.634549	1.799801	
Congo, Democr	Deaths	Male	2021	3.911023	6.972103	1.886262	
Congo, Democr	Deaths	Male	2022	4.296873	7.864356	1.933344	
Congo, Democr	Deaths	Male	2023	4.386336	7.676849	1.869401	
Congo, Democr	Deaths	Female	2010	1.673387	3.216607	0.875099	
Congo, Democr	Deaths	Female	2011	1.709861	3.314713	0.900037	
Congo, Democr	Deaths	Female	2012	1.750226	3.341457	0.920863	
Congo, Democr	Deaths	Female	2013	1.799613	3.446371	0.966082	
Congo, Democr	Deaths	Female	2014	1.867438	3.526764	1.018899	
Congo, Democr	Deaths	Female	2015	1.922805	3.470353	1.025799	
Congo, Democr	Deaths	Female	2016	1.988566	3.446262	1.067251	
Congo, Democr	Deaths	Female	2017	2.06441	3.407629	1.103895	
Congo, Democr	Deaths	Female	2018	2.167881	3.511954	1.158318	
Congo, Democr	Deaths	Female	2019	2.275302	3.741129	1.239963	
Congo, Democr	Deaths	Female	2020	2.401536	4.081557	1.329801	
Congo, Democr	Deaths	Female	2021	2.484557	4.140024	1.367943	
Congo, Democr	Deaths	Female	2022	2.952134	4.835773	1.596058	
Congo, Democr	Deaths	Female	2023	3.076437	5.06533	1.68486	
Congo, Democr	DALYs	Male	2010	83.04334	148.1333	39.791	
Congo, Democr	DALYs	Male	2011	84.33836	148.8644	40.99085	
Congo, Democr	DALYs	Male	2012	85.7486	149.0307	41.18511	
Congo, Democr	DALYs	Male	2013	87.9745	152.5805	43.29072	
Congo, Democr	DALYs	Male	2014	91.13301	155.5696	44.50888	
Congo, Democr	DALYs	Male	2015	92.9545	156.8536	45.27888	
Congo, Democr	DALYs	Male	2016	94.58158	164.2338	45.30891	
Congo, Democr	DALYs	Male	2017	96.45596	167.386	46.14644	
Congo, Democr	DALYs	Male	2018	99.23898	173.6075	47.50413	
Congo, Democr	DALYs	Male	2019	102.2845	182.608	48.37371	
Congo, Democr	DALYs	Male	2020	111.5989	193.7777	52.70131	
Congo, Democr	DALYs	Male	2021	113.4837	203.2363	54.62046	
Congo, Democr	DALYs	Male	2022	124.6409	229.5477	57.8738	
Congo, Democr	DALYs	Male	2023	127.6675	225.3691	56.29061	
Congo, Democr	DALYs	Female	2010	46.61766	90.39435	24.55003	
Congo, Democr	DALYs	Female	2011	47.79758	93.84222	24.87005	
Congo, Democr	DALYs	Female	2012	49.1673	96.59215	26.71975	

Congo, Democr	DALYs	Female	2013	50.9345	97.65957	28.2566	
Congo, Democr	DALYs	Female	2014	53.28305	99.83512	29.4044	
Congo, Democr	DALYs	Female	2015	55.04109	99.77031	29.51235	
Congo, Democr	DALYs	Female	2016	57.16786	97.42938	30.78515	
Congo, Democr	DALYs	Female	2017	59.7592	97.18502	32.1836	
Congo, Democr	DALYs	Female	2018	63.22526	100.8324	33.94222	
Congo, Democr	DALYs	Female	2019	66.64611	109.2483	36.59893	
Congo, Democr	DALYs	Female	2020	70.19399	117.3748	40.16633	
Congo, Democr	DALYs	Female	2021	72.90296	119.4447	40.74296	
Congo, Democr	DALYs	Female	2022	86.70539	139.2762	48.52001	
Congo, Democr	DALYs	Female	2023	90.62857	149.037	50.14972	
Denmark	Incidence	Male	2010	95.88947	101.0079	89.45197	
Denmark	Incidence	Male	2011	95.5793	101.4452	89.32909	
Denmark	Incidence	Male	2012	96.64154	102.2759	90.72818	
Denmark	Incidence	Male	2013	97.64562	103.865	91.39877	
Denmark	Incidence	Male	2014	98.9238	105.7446	92.38922	
Denmark	Incidence	Male	2015	92.76404	98.76919	85.93355	
Denmark	Incidence	Male	2016	90.70182	96.62512	84.34397	
Denmark	Incidence	Male	2017	91.21711	97.11386	85.33733	
Denmark	Incidence	Male	2018	87.95821	93.42785	82.01275	
Denmark	Incidence	Male	2019	86.84254	92.22894	81.68235	
Denmark	Incidence	Male	2020	84.80901	89.82083	79.61974	
Denmark	Incidence	Male	2021	89.09408	94.50072	84.3482	
Denmark	Incidence	Male	2022	90.06999	95.69757	84.88592	
Denmark	Incidence	Male	2023	91.08929	98.05172	85.08846	
Denmark	Incidence	Female	2010	88.14582	94.74831	80.97423	
Denmark	Incidence	Female	2011	88.44308	95.94959	81.41199	
Denmark	Incidence	Female	2012	89.2565	96.08541	81.92779	
Denmark	Incidence	Female	2013	89.58663	97.92689	81.48909	
Denmark	Incidence	Female	2014	89.41943	97.37959	81.26726	
Denmark	Incidence	Female	2015	88.73441	95.86475	79.73487	
Denmark	Incidence	Female	2016	87.60927	95.73653	78.79966	
Denmark	Incidence	Female	2017	87.34725	95.4384	79.00867	
Denmark	Incidence	Female	2018	85.64783	94.39919	77.10412	
Denmark	Incidence	Female	2019	86.97579	96.13579	78.47131	
Denmark	Incidence	Female	2020	84.90312	93.47351	76.89715	

Denmark	Incidence	Female	2021	88.16076	97.21034	79.21792	
Denmark	Incidence	Female	2022	91.26768	100.9578	81.37874	
Denmark	Incidence	Female	2023	87.84971	97.67641	77.83397	
Denmark	Deaths	Male	2010	82.22166	85.97409	77.41948	
Denmark	Deaths	Male	2011	81.50801	85.49328	77.04504	
Denmark	Deaths	Male	2012	82.26543	86.03686	77.44406	
Denmark	Deaths	Male	2013	82.87298	86.95399	78.01608	
Denmark	Deaths	Male	2014	84.09212	88.32407	79.19928	
Denmark	Deaths	Male	2015	79.75807	83.91157	75.16639	
Denmark	Deaths	Male	2016	78.5893	82.48308	74.16883	
Denmark	Deaths	Male	2017	79.63446	83.73706	74.81091	
Denmark	Deaths	Male	2018	77.50553	81.81709	72.55829	
Denmark	Deaths	Male	2019	76.72522	80.25471	71.59192	
Denmark	Deaths	Male	2020	75.1536	79.11056	70.54517	
Denmark	Deaths	Male	2021	79.20016	83.57686	74.70762	
Denmark	Deaths	Male	2022	80.45424	84.75795	75.74663	
Denmark	Deaths	Male	2023	81.75085	86.80801	76.55802	
Denmark	Deaths	Female	2010	73.86382	78.88632	67.81602	
Denmark	Deaths	Female	2011	73.81463	79.43259	67.07345	
Denmark	Deaths	Female	2012	74.26969	79.66451	67.59007	
Denmark	Deaths	Female	2013	74.47791	80.67769	67.29833	
Denmark	Deaths	Female	2014	74.08329	80.76764	66.54502	
Denmark	Deaths	Female	2015	73.93377	79.87119	66.2088	
Denmark	Deaths	Female	2016	73.76134	80.23778	66.52651	
Denmark	Deaths	Female	2017	74.49512	80.83109	67.48682	
Denmark	Deaths	Female	2018	73.67382	80.9419	66.07656	
Denmark	Deaths	Female	2019	74.91603	82.88884	67.18978	
Denmark	Deaths	Female	2020	73.56246	81.27194	65.74119	
Denmark	Deaths	Female	2021	76.65875	85.01145	68.61695	
Denmark	Deaths	Female	2022	79.62352	88.6686	70.39517	
Denmark	Deaths	Female	2023	77.25016	86.42413	68.59793	
Denmark	DALYs	Male	2010	1775.581	1855.469	1684.509	
Denmark	DALYs	Male	2011	1752.178	1829.72	1661.663	
Denmark	DALYs	Male	2012	1755.561	1836.904	1665.648	
Denmark	DALYs	Male	2013	1768.795	1846.797	1681.862	
Denmark	DALYs	Male	2014	1785.403	1864.402	1696.824	

Denmark	DALYs	Male	2015	1658.949	1736.113	1578.273	
Denmark	DALYs	Male	2016	1620.051	1693.435	1539.583	
Denmark	DALYs	Male	2017	1629.597	1702.2	1545.469	
Denmark	DALYs	Male	2018	1562.64	1639.294	1476.738	
Denmark	DALYs	Male	2019	1535.028	1598.191	1451.451	
Denmark	DALYs	Male	2020	1490.493	1555.732	1414.039	
Denmark	DALYs	Male	2021	1549.575	1617.744	1477.114	
Denmark	DALYs	Male	2022	1562.888	1637.404	1490.402	
Denmark	DALYs	Male	2023	1580.129	1662.317	1494.033	
Denmark	DALYs	Female	2010	1589.663	1686.133	1484.7	
Denmark	DALYs	Female	2011	1571.684	1680.739	1467.616	
Denmark	DALYs	Female	2012	1563.479	1660.921	1450.269	
Denmark	DALYs	Female	2013	1550.452	1662.684	1432.032	
Denmark	DALYs	Female	2014	1546.121	1674.399	1419.998	
Denmark	DALYs	Female	2015	1533.824	1639.388	1410.134	
Denmark	DALYs	Female	2016	1509.453	1623.445	1390.219	
Denmark	DALYs	Female	2017	1499.235	1611.267	1381.773	
Denmark	DALYs	Female	2018	1464.082	1586.324	1340.821	
Denmark	DALYs	Female	2019	1484.662	1624.613	1357.163	
Denmark	DALYs	Female	2020	1435.031	1557.976	1310.239	
Denmark	DALYs	Female	2021	1479.598	1623.688	1348.854	
Denmark	DALYs	Female	2022	1527.2	1681.885	1378.742	
Denmark	DALYs	Female	2023	1469.497	1636.424	1317.474	
Djibouti	Incidence	Male	2010	4.09005	6.031094	2.291107	
Djibouti	Incidence	Male	2011	4.233174	5.992115	2.356377	
Djibouti	Incidence	Male	2012	4.433829	6.380003	2.444412	
Djibouti	Incidence	Male	2013	4.526415	6.759164	2.501065	
Djibouti	Incidence	Male	2014	4.501727	6.805048	2.448552	
Djibouti	Incidence	Male	2015	4.825296	7.176379	2.600386	
Djibouti	Incidence	Male	2016	5.002522	7.318947	2.679487	
Djibouti	Incidence	Male	2017	5.220331	7.703896	2.797138	
Djibouti	Incidence	Male	2018	5.203599	7.963888	2.71294	
Djibouti	Incidence	Male	2019	5.421643	8.362195	3.027403	
Djibouti	Incidence	Male	2020	5.526034	8.141739	2.941141	
Djibouti	Incidence	Male	2021	6.366636	9.896942	3.30806	
Djibouti	Incidence	Male	2022	6.123918	8.858412	3.40383	

Djibouti	Incidence	Male	2023	6.69922	9.716984	3.671476	
Djibouti	Incidence	Female	2010	1.331141	2.024042	0.832936	
Djibouti	Incidence	Female	2011	1.369133	1.950906	0.852243	
Djibouti	Incidence	Female	2012	1.426003	1.998211	0.905293	
Djibouti	Incidence	Female	2013	1.472185	2.100954	0.931717	
Djibouti	Incidence	Female	2014	1.474642	2.088167	0.922583	
Djibouti	Incidence	Female	2015	1.596571	2.23744	1.00278	
Djibouti	Incidence	Female	2016	1.658948	2.335442	1.046745	
Djibouti	Incidence	Female	2017	1.788997	2.535193	1.153132	
Djibouti	Incidence	Female	2018	1.816145	2.546795	1.13553	
Djibouti	Incidence	Female	2019	1.908465	2.692885	1.314343	
Djibouti	Incidence	Female	2020	1.871043	2.642009	1.266098	
Djibouti	Incidence	Female	2021	2.003397	2.845437	1.332398	
Djibouti	Incidence	Female	2022	2.196608	3.12678	1.476159	
Djibouti	Incidence	Female	2023	2.426201	3.3823	1.619932	
Djibouti	Deaths	Male	2010	3.904271	5.745719	2.185983	
Djibouti	Deaths	Male	2011	4.039025	5.653551	2.244027	
Djibouti	Deaths	Male	2012	4.226954	6.094691	2.321296	
Djibouti	Deaths	Male	2013	4.314232	6.42278	2.371941	
Djibouti	Deaths	Male	2014	4.293353	6.480428	2.328152	
Djibouti	Deaths	Male	2015	4.597959	6.816454	2.488566	
Djibouti	Deaths	Male	2016	4.762346	6.943792	2.549102	
Djibouti	Deaths	Male	2017	4.970247	7.325525	2.680674	
Djibouti	Deaths	Male	2018	4.957636	7.577261	2.599695	
Djibouti	Deaths	Male	2019	5.168396	7.985359	2.875594	
Djibouti	Deaths	Male	2020	5.277401	7.79808	2.788403	
Djibouti	Deaths	Male	2021	6.082767	9.523046	3.143966	
Djibouti	Deaths	Male	2022	5.840575	8.45447	3.234567	
Djibouti	Deaths	Male	2023	6.384541	9.275767	3.489914	
Djibouti	Deaths	Female	2010	1.260807	1.918917	0.787302	
Djibouti	Deaths	Female	2011	1.29552	1.854989	0.805435	
Djibouti	Deaths	Female	2012	1.346511	1.891317	0.856577	
Djibouti	Deaths	Female	2013	1.387684	1.989065	0.878808	
Djibouti	Deaths	Female	2014	1.388108	1.970044	0.866052	
Djibouti	Deaths	Female	2015	1.500365	2.082273	0.942529	
Djibouti	Deaths	Female	2016	1.555342	2.195687	0.976252	

Djibouti	Deaths	Female	2017	1.671727	2.380533	1.078694	
Djibouti	Deaths	Female	2018	1.694322	2.361328	1.052266	
Djibouti	Deaths	Female	2019	1.780039	2.488719	1.214072	
Djibouti	Deaths	Female	2020	1.746484	2.456122	1.17969	
Djibouti	Deaths	Female	2021	1.866942	2.649073	1.242913	
Djibouti	Deaths	Female	2022	2.045015	2.915977	1.366843	
Djibouti	Deaths	Female	2023	2.258121	3.179143	1.503302	
Djibouti	DALYs	Male	2010	125.5546	186.4109	70.74108	
Djibouti	DALYs	Male	2011	130.0364	185.8108	72.81088	
Djibouti	DALYs	Male	2012	136.3491	196.2179	75.61087	
Djibouti	DALYs	Male	2013	139.1473	208.3765	76.47498	
Djibouti	DALYs	Male	2014	138.085	209.1375	74.4098	
Djibouti	DALYs	Male	2015	148.0846	220.9734	80.18311	
Djibouti	DALYs	Male	2016	153.5952	225.059	82.54076	
Djibouti	DALYs	Male	2017	159.9938	238.3356	84.89458	
Djibouti	DALYs	Male	2018	159.0784	244.7092	82.39567	
Djibouti	DALYs	Male	2019	165.3533	253.86	93.08904	
Djibouti	DALYs	Male	2020	167.7158	246.6447	90.29956	
Djibouti	DALYs	Male	2021	192.595	297.5635	100.4255	
Djibouti	DALYs	Male	2022	185.7402	268.4754	103.5387	
Djibouti	DALYs	Male	2023	203.1246	297.6584	110.6929	
Djibouti	DALYs	Female	2010	42.39408	64.74767	26.70569	
Djibouti	DALYs	Female	2011	43.64874	62.3131	27.52676	
Djibouti	DALYs	Female	2012	45.57659	64.13644	28.87301	
Djibouti	DALYs	Female	2013	47.10875	67.6559	29.46891	
Djibouti	DALYs	Female	2014	47.17098	66.76669	29.84664	
Djibouti	DALYs	Female	2015	51.16979	72.3363	31.81582	
Djibouti	DALYs	Female	2016	53.28493	75.02137	33.58896	
Djibouti	DALYs	Female	2017	57.73739	81.76695	36.63984	
Djibouti	DALYs	Female	2018	58.70565	82.93347	37.19537	
Djibouti	DALYs	Female	2019	61.5781	87.97863	41.61978	
Djibouti	DALYs	Female	2020	60.21134	85.64939	40.63036	
Djibouti	DALYs	Female	2021	64.47932	90.51174	42.9829	
Djibouti	DALYs	Female	2022	70.66695	101.4805	48.14989	
Djibouti	DALYs	Female	2023	77.91616	108.5747	52.33032	
Dominican Rep	Incidence	Male	2010	11.86303	14.57233	9.767556	

Dominican Rep	Incidence	Male	2011	12.26345	14.72572	10.09235	
Dominican Rep	Incidence	Male	2012	12.47839	14.97923	10.27356	
Dominican Rep	Incidence	Male	2013	12.50353	15.14816	10.28391	
Dominican Rep	Incidence	Male	2014	12.56418	15.15316	10.333	
Dominican Rep	Incidence	Male	2015	13.05038	15.73182	10.7043	
Dominican Rep	Incidence	Male	2016	13.51984	16.29588	11.10603	
Dominican Rep	Incidence	Male	2017	14.01029	16.74539	11.27362	
Dominican Rep	Incidence	Male	2018	14.63075	17.97329	11.58423	
Dominican Rep	Incidence	Male	2019	15.4069	19.05895	11.95377	
Dominican Rep	Incidence	Male	2020	17.40523	21.67482	13.4916	
Dominican Rep	Incidence	Male	2021	17.77727	22.44423	13.5775	
Dominican Rep	Incidence	Male	2022	18.43764	23.87519	13.71578	
Dominican Rep	Incidence	Male	2023	18.78845	24.55265	14.27107	
Dominican Rep	Incidence	Female	2010	7.451734	9.221879	5.866498	
Dominican Rep	Incidence	Female	2011	7.774096	9.603943	6.304645	
Dominican Rep	Incidence	Female	2012	7.982352	9.61176	6.501609	
Dominican Rep	Incidence	Female	2013	8.086737	9.682331	6.658902	
Dominican Rep	Incidence	Female	2014	8.267018	9.899375	6.837967	
Dominican Rep	Incidence	Female	2015	8.71697	10.39652	7.172317	
Dominican Rep	Incidence	Female	2016	9.163167	10.85831	7.522761	
Dominican Rep	Incidence	Female	2017	9.606037	11.44138	7.84493	
Dominican Rep	Incidence	Female	2018	10.01382	12.12641	8.028301	
Dominican Rep	Incidence	Female	2019	10.34765	12.61583	8.139139	
Dominican Rep	Incidence	Female	2020	10.58975	13.17152	8.336331	
Dominican Rep	Incidence	Female	2021	11.27974	14.22508	8.776082	
Dominican Rep	Incidence	Female	2022	11.92239	15.21545	9.264328	
Dominican Rep	Incidence	Female	2023	12.5317	16.14527	9.609423	
Dominican Rep	Deaths	Male	2010	12.05587	14.82467	9.874626	
Dominican Rep	Deaths	Male	2011	12.48547	15.12352	10.24193	
Dominican Rep	Deaths	Male	2012	12.73684	15.40215	10.49294	
Dominican Rep	Deaths	Male	2013	12.79483	15.49395	10.55348	
Dominican Rep	Deaths	Male	2014	12.88783	15.63715	10.66374	
Dominican Rep	Deaths	Male	2015	13.38948	16.19167	11.05681	
Dominican Rep	Deaths	Male	2016	13.87258	16.70489	11.52444	
Dominican Rep	Deaths	Male	2017	14.36845	17.239	11.72729	
Dominican Rep	Deaths	Male	2018	14.98458	18.27926	11.89796	

Dominican Rep	Deaths	Male	2019	15.75536	19.4341	12.13395	
Dominican Rep	Deaths	Male	2020	17.7636	22.2028	13.75942	
Dominican Rep	Deaths	Male	2021	18.12087	23.10731	13.82219	
Dominican Rep	Deaths	Male	2022	18.79261	24.55745	14.08421	
Dominican Rep	Deaths	Male	2023	19.09676	25.30742	14.51804	
Dominican Rep	Deaths	Female	2010	7.372682	9.145924	5.830228	
Dominican Rep	Deaths	Female	2011	7.70555	9.446015	6.163141	
Dominican Rep	Deaths	Female	2012	7.936666	9.547869	6.464618	
Dominican Rep	Deaths	Female	2013	8.077437	9.712042	6.730161	
Dominican Rep	Deaths	Female	2014	8.293195	9.895951	6.874238	
Dominican Rep	Deaths	Female	2015	8.746291	10.40254	7.185227	
Dominican Rep	Deaths	Female	2016	9.19168	10.87116	7.520132	
Dominican Rep	Deaths	Female	2017	9.646617	11.55118	7.82215	
Dominican Rep	Deaths	Female	2018	10.0455	12.17074	7.969195	
Dominican Rep	Deaths	Female	2019	10.35079	12.71129	8.130312	
Dominican Rep	Deaths	Female	2020	10.57379	13.15801	8.269278	
Dominican Rep	Deaths	Female	2021	11.24508	14.22844	8.723153	
Dominican Rep	Deaths	Female	2022	11.87448	15.22406	9.133679	
Dominican Rep	Deaths	Female	2023	12.47235	15.93932	9.479272	
Dominican Rep	DALYs	Male	2010	294.0068	359.3765	242.5906	
Dominican Rep	DALYs	Male	2011	303.2968	363.3176	248.2336	
Dominican Rep	DALYs	Male	2012	307.7491	365.7078	251.5152	
Dominican Rep	DALYs	Male	2013	307.3192	369.2244	251.0419	
Dominican Rep	DALYs	Male	2014	307.8308	368.3969	252.5129	
Dominican Rep	DALYs	Male	2015	318.8996	381.7914	262.6748	
Dominican Rep	DALYs	Male	2016	329.5931	394.4861	270.168	
Dominican Rep	DALYs	Male	2017	340.9057	408.0949	273.8478	
Dominican Rep	DALYs	Male	2018	355.2762	437.4891	280.7039	
Dominican Rep	DALYs	Male	2019	373.9885	465.9496	289.917	
Dominican Rep	DALYs	Male	2020	422.4164	529.2211	322.1633	
Dominican Rep	DALYs	Male	2021	431.7258	546.2364	329.5911	
Dominican Rep	DALYs	Male	2022	446.67	579.4549	329.3021	
Dominican Rep	DALYs	Male	2023	455.8798	595.2774	345.9209	
Dominican Rep	DALYs	Female	2010	187.7818	232.9802	150.1296	
Dominican Rep	DALYs	Female	2011	195.4588	242.9222	158.0183	
Dominican Rep	DALYs	Female	2012	200.2459	241.231	163.7608	

Dominican Rep	DALYs	Female	2013	201.8322	240.9509	165.9736	
Dominican Rep	DALYs	Female	2014	205.0601	244.6043	169.6502	
Dominican Rep	DALYs	Female	2015	215.7259	256.0726	176.3642	
Dominican Rep	DALYs	Female	2016	225.9643	267.9621	183.7449	
Dominican Rep	DALYs	Female	2017	235.4722	278.6011	188.1635	
Dominican Rep	DALYs	Female	2018	244.8834	297.4396	194.3924	
Dominican Rep	DALYs	Female	2019	253.3058	312.1198	197.7466	
Dominican Rep	DALYs	Female	2020	259.9951	323.8543	202.5303	
Dominican Rep	DALYs	Female	2021	276.6454	346.5877	216.0276	
Dominican Rep	DALYs	Female	2022	291.6631	371.142	224.4949	
Dominican Rep	DALYs	Female	2023	305.5991	386.5004	236.9445	
Ecuador	Incidence	Male	2010	8.399154	9.016749	7.785135	
Ecuador	Incidence	Male	2011	8.24032	8.916538	7.595607	
Ecuador	Incidence	Male	2012	7.971481	8.600607	7.267028	
Ecuador	Incidence	Male	2013	7.301084	7.905775	6.637265	
Ecuador	Incidence	Male	2014	7.055564	7.660839	6.396286	
Ecuador	Incidence	Male	2015	6.641601	7.261442	6.023921	
Ecuador	Incidence	Male	2016	6.459411	7.073031	5.896088	
Ecuador	Incidence	Male	2017	6.769929	7.392974	6.15263	
Ecuador	Incidence	Male	2018	6.992881	7.567532	6.369585	
Ecuador	Incidence	Male	2019	7.549658	8.212051	6.908037	
Ecuador	Incidence	Male	2020	6.677188	7.214264	6.126803	
Ecuador	Incidence	Male	2021	6.605298	7.150638	6.076424	
Ecuador	Incidence	Male	2022	6.825589	7.410323	6.266067	
Ecuador	Incidence	Male	2023	6.609848	7.469697	5.811251	
Ecuador	Incidence	Female	2010	6.290508	6.978298	5.692193	
Ecuador	Incidence	Female	2011	6.607747	7.42074	5.952496	
Ecuador	Incidence	Female	2012	6.541554	7.34811	5.920919	
Ecuador	Incidence	Female	2013	6.237841	6.947636	5.609272	
Ecuador	Incidence	Female	2014	6.323648	7.051175	5.687796	
Ecuador	Incidence	Female	2015	5.835157	6.464328	5.212494	
Ecuador	Incidence	Female	2016	5.986744	6.645462	5.33334	
Ecuador	Incidence	Female	2017	6.250919	6.917988	5.528937	
Ecuador	Incidence	Female	2018	6.164465	6.813499	5.418784	
Ecuador	Incidence	Female	2019	6.706012	7.424392	5.890319	
Ecuador	Incidence	Female	2020	6.360542	7.052941	5.638771	

Ecuador	Incidence	Female	2021	6.345819	7.1241	5.652553	
Ecuador	Incidence	Female	2022	6.60159	7.525905	5.909704	
Ecuador	Incidence	Female	2023	7.000607	8.08889	6.168523	
Ecuador	Deaths	Male	2010	8.771506	9.422575	8.149834	
Ecuador	Deaths	Male	2011	8.601839	9.240412	7.948243	
Ecuador	Deaths	Male	2012	8.30099	8.925798	7.556663	
Ecuador	Deaths	Male	2013	7.565608	8.114696	6.854651	
Ecuador	Deaths	Male	2014	7.314584	7.946405	6.650247	
Ecuador	Deaths	Male	2015	6.897274	7.514795	6.295765	
Ecuador	Deaths	Male	2016	6.697444	7.300169	6.124778	
Ecuador	Deaths	Male	2017	7.014786	7.564101	6.388461	
Ecuador	Deaths	Male	2018	7.252551	7.808814	6.622347	
Ecuador	Deaths	Male	2019	7.777332	8.463726	7.168243	
Ecuador	Deaths	Male	2020	6.841905	7.362667	6.3261	
Ecuador	Deaths	Male	2021	6.706417	7.258269	6.238193	
Ecuador	Deaths	Male	2022	6.901598	7.47188	6.403016	
Ecuador	Deaths	Male	2023	6.680241	7.560953	5.931556	
Ecuador	Deaths	Female	2010	5.900916	6.605572	5.38998	
Ecuador	Deaths	Female	2011	6.152664	6.851374	5.631444	
Ecuador	Deaths	Female	2012	6.027794	6.670743	5.518539	
Ecuador	Deaths	Female	2013	5.732442	6.323941	5.205126	
Ecuador	Deaths	Female	2014	5.782358	6.380098	5.268665	
Ecuador	Deaths	Female	2015	5.327186	5.827015	4.853964	
Ecuador	Deaths	Female	2016	5.453479	5.974549	4.968812	
Ecuador	Deaths	Female	2017	5.671019	6.208776	5.134535	
Ecuador	Deaths	Female	2018	5.572635	6.081362	5.071142	
Ecuador	Deaths	Female	2019	5.993251	6.552577	5.456015	
Ecuador	Deaths	Female	2020	5.608792	6.083567	5.104309	
Ecuador	Deaths	Female	2021	5.506526	6.016479	5.058901	
Ecuador	Deaths	Female	2022	5.695063	6.289669	5.195988	
Ecuador	Deaths	Female	2023	6.033256	6.682751	5.500158	
Ecuador	DALYs	Male	2010	200.6839	215.4448	185.8189	
Ecuador	DALYs	Male	2011	195.4263	210.5461	178.5729	
Ecuador	DALYs	Male	2012	188.828	203.9885	171.3077	
Ecuador	DALYs	Male	2013	173.0773	186.987	156.5521	
Ecuador	DALYs	Male	2014	166.866	182.0762	150.5892	

Ecuador	DALYs	Male	2015	155.7166	170.885	141.1218	
Ecuador	DALYs	Male	2016	151.1096	164.7678	137.9795	
Ecuador	DALYs	Male	2017	157.6819	171.6879	143.7029	
Ecuador	DALYs	Male	2018	161.8992	174.7164	147.9451	
Ecuador	DALYs	Male	2019	174.5391	188.603	160.1582	
Ecuador	DALYs	Male	2020	153.7909	166.3618	141.855	
Ecuador	DALYs	Male	2021	153.5257	166.1661	142.1214	
Ecuador	DALYs	Male	2022	159.5005	172.4793	148.0805	
Ecuador	DALYs	Male	2023	154.0668	174.1082	137.0946	
Ecuador	DALYs	Female	2010	140.0552	158.1862	126.9181	
Ecuador	DALYs	Female	2011	144.9262	163.3607	132.2511	
Ecuador	DALYs	Female	2012	142.9513	160.9399	130.3927	
Ecuador	DALYs	Female	2013	134.331	149.6204	121.9925	
Ecuador	DALYs	Female	2014	134.8896	149.8445	122.9426	
Ecuador	DALYs	Female	2015	122.5521	135.8167	111.6614	
Ecuador	DALYs	Female	2016	124.5975	137.542	114.7178	
Ecuador	DALYs	Female	2017	130.0342	142.4219	119.2874	
Ecuador	DALYs	Female	2018	128.0151	139.7719	117.57	
Ecuador	DALYs	Female	2019	137.8451	150.208	126.3202	
Ecuador	DALYs	Female	2020	128.7782	139.7764	117.9672	
Ecuador	DALYs	Female	2021	128.3819	139.3758	117.3437	
Ecuador	DALYs	Female	2022	133.7627	147.9308	122.6196	
Ecuador	DALYs	Female	2023	140.9317	156.5742	129.4161	
Egypt	Incidence	Male	2010	11.98613	15.22499	9.101521	
Egypt	Incidence	Male	2011	11.75292	14.90508	9.114906	
Egypt	Incidence	Male	2012	11.94944	15.0742	9.369903	
Egypt	Incidence	Male	2013	11.79747	14.67493	9.238252	
Egypt	Incidence	Male	2014	11.99351	14.70705	9.410725	
Egypt	Incidence	Male	2015	12.5818	15.18107	9.742125	
Egypt	Incidence	Male	2016	12.35327	14.8175	9.571014	
Egypt	Incidence	Male	2017	12.15974	14.55344	9.613885	
Egypt	Incidence	Male	2018	12.50912	15.12714	9.771497	
Egypt	Incidence	Male	2019	12.51202	15.53854	9.909357	
Egypt	Incidence	Male	2020	12.10969	15.75103	9.328588	
Egypt	Incidence	Male	2021	13.33813	17.41125	10.20294	
Egypt	Incidence	Male	2022	15.92925	21.20591	11.47163	

Egypt	Incidence	Male	2023	16.26744	22.19174	11.83695	
Egypt	Incidence	Female	2010	3.658759	4.858793	2.576815	
Egypt	Incidence	Female	2011	3.729781	4.995574	2.607132	
Egypt	Incidence	Female	2012	3.832808	5.095639	2.667549	
Egypt	Incidence	Female	2013	3.75968	4.920537	2.682781	
Egypt	Incidence	Female	2014	3.913165	5.037673	2.838906	
Egypt	Incidence	Female	2015	4.162085	5.29107	3.086053	
Egypt	Incidence	Female	2016	4.187377	5.342715	3.236177	
Egypt	Incidence	Female	2017	4.230291	5.338224	3.278016	
Egypt	Incidence	Female	2018	4.314652	5.397488	3.373126	
Egypt	Incidence	Female	2019	4.325914	5.368318	3.318296	
Egypt	Incidence	Female	2020	4.344884	5.489469	3.335217	
Egypt	Incidence	Female	2021	4.818616	6.120421	3.641719	
Egypt	Incidence	Female	2022	6.017441	8.16359	4.455445	
Egypt	Incidence	Female	2023	6.267545	8.722339	4.43194	
Egypt	Deaths	Male	2010	11.4816	14.52694	8.727667	
Egypt	Deaths	Male	2011	11.25382	14.2458	8.742258	
Egypt	Deaths	Male	2012	11.44634	14.385	8.974283	
Egypt	Deaths	Male	2013	11.28165	13.97629	8.916521	
Egypt	Deaths	Male	2014	11.4744	14.11093	9.057719	
Egypt	Deaths	Male	2015	12.05894	14.62177	9.377183	
Egypt	Deaths	Male	2016	11.84351	14.25482	9.189965	
Egypt	Deaths	Male	2017	11.65402	14.0214	9.189189	
Egypt	Deaths	Male	2018	11.99042	14.52561	9.435373	
Egypt	Deaths	Male	2019	11.99674	14.95165	9.566074	
Egypt	Deaths	Male	2020	11.64426	15.04032	8.958182	
Egypt	Deaths	Male	2021	12.85387	16.64134	9.807862	
Egypt	Deaths	Male	2022	15.35021	20.36981	11.02576	
Egypt	Deaths	Male	2023	15.6224	21.30078	11.34906	
Egypt	Deaths	Female	2010	3.464363	4.607825	2.465984	
Egypt	Deaths	Female	2011	3.533212	4.737776	2.485788	
Egypt	Deaths	Female	2012	3.635432	4.840225	2.56555	
Egypt	Deaths	Female	2013	3.556461	4.650471	2.554667	
Egypt	Deaths	Female	2014	3.700836	4.763348	2.711331	
Egypt	Deaths	Female	2015	3.940931	4.979454	2.933079	
Egypt	Deaths	Female	2016	3.958235	5.014675	3.073695	

Egypt	Deaths	Female	2017	3.992887	5.027177	3.09285	
Egypt	Deaths	Female	2018	4.070653	5.071876	3.194462	
Egypt	Deaths	Female	2019	4.084526	5.050872	3.14723	
Egypt	Deaths	Female	2020	4.124965	5.205352	3.208678	
Egypt	Deaths	Female	2021	4.586566	5.830875	3.474385	
Egypt	Deaths	Female	2022	5.700747	7.706853	4.203555	
Egypt	Deaths	Female	2023	5.899217	8.19955	4.128839	
Egypt	DALYs	Male	2010	342.8671	437.7953	257.8442	
Egypt	DALYs	Male	2011	336.1294	428.2771	255.7041	
Egypt	DALYs	Male	2012	340.4614	430.7754	262.5772	
Egypt	DALYs	Male	2013	336.8148	416.9557	261.7313	
Egypt	DALYs	Male	2014	340.8323	419.8013	266.6365	
Egypt	DALYs	Male	2015	354.0713	422.0147	274.5529	
Egypt	DALYs	Male	2016	345.2769	412.4123	266.3554	
Egypt	DALYs	Male	2017	337.764	406.1639	264.942	
Egypt	DALYs	Male	2018	345.3238	418.3951	265.0063	
Egypt	DALYs	Male	2019	343.9803	430.7117	264.4704	
Egypt	DALYs	Male	2020	329.5976	427.5835	253.761	
Egypt	DALYs	Male	2021	359.0099	469.7031	275.2079	
Egypt	DALYs	Male	2022	425.6628	559.4894	307.9737	
Egypt	DALYs	Male	2023	435.3246	593.7618	312.6285	
Egypt	DALYs	Female	2010	106.6057	141.2995	75.45266	
Egypt	DALYs	Female	2011	108.3066	145.6914	75.66393	
Egypt	DALYs	Female	2012	110.4559	147.8331	76.87785	
Egypt	DALYs	Female	2013	108.6686	142.1929	77.46322	
Egypt	DALYs	Female	2014	112.7128	144.664	81.95005	
Egypt	DALYs	Female	2015	118.847	149.6858	88.60918	
Egypt	DALYs	Female	2016	119.201	151.2473	92.38267	
Egypt	DALYs	Female	2017	119.9212	151.7638	92.22982	
Egypt	DALYs	Female	2018	121.602	152.3897	93.84068	
Egypt	DALYs	Female	2019	121.1952	150.5202	92.80286	
Egypt	DALYs	Female	2020	119.556	151.7598	90.65809	
Egypt	DALYs	Female	2021	130.474	166.2474	96.68557	
Egypt	DALYs	Female	2022	163.6178	219.9054	121.6288	
Egypt	DALYs	Female	2023	171.7906	235.1764	121.693	
El Salvador	Incidence	Male	2010	8.515744	11.44412	7.003039	

El Salvador	Incidence	Male	2011	8.444643	11.49947	6.97267	
El Salvador	Incidence	Male	2012	9.309996	12.72764	7.690809	
El Salvador	Incidence	Male	2013	9.597657	13.19256	8.064904	
El Salvador	Incidence	Male	2014	9.807491	13.50182	8.195847	
El Salvador	Incidence	Male	2015	9.809112	13.83992	8.292125	
El Salvador	Incidence	Male	2016	9.807866	13.96983	8.423062	
El Salvador	Incidence	Male	2017	10.22551	14.5744	8.879596	
El Salvador	Incidence	Male	2018	10.57608	15.10138	9.046618	
El Salvador	Incidence	Male	2019	11.15461	16.03038	9.465127	
El Salvador	Incidence	Male	2020	11.9617	17.20147	10.13293	
El Salvador	Incidence	Male	2021	12.90091	18.15824	10.81135	
El Salvador	Incidence	Male	2022	12.61099	18.48113	10.12744	
El Salvador	Incidence	Male	2023	12.94302	18.33155	10.22016	
El Salvador	Incidence	Female	2010	7.360279	8.799166	6.097173	
El Salvador	Incidence	Female	2011	7.492503	8.899621	6.227416	
El Salvador	Incidence	Female	2012	7.96433	9.606915	6.7089	
El Salvador	Incidence	Female	2013	8.237489	10.22083	6.862375	
El Salvador	Incidence	Female	2014	8.459539	10.42068	7.177131	
El Salvador	Incidence	Female	2015	8.529306	10.69535	7.286261	
El Salvador	Incidence	Female	2016	8.511739	10.97501	7.314423	
El Salvador	Incidence	Female	2017	8.721795	11.17477	7.573733	
El Salvador	Incidence	Female	2018	9.170947	11.69847	7.890023	
El Salvador	Incidence	Female	2019	9.782245	12.34567	8.396386	
El Salvador	Incidence	Female	2020	10.3735	13.11232	8.785087	
El Salvador	Incidence	Female	2021	11.5443	14.65991	9.839555	
El Salvador	Incidence	Female	2022	12.21511	15.64348	10.30483	
El Salvador	Incidence	Female	2023	13.04647	16.94924	10.54663	
El Salvador	Deaths	Male	2010	8.609645	11.62338	7.077596	
El Salvador	Deaths	Male	2011	8.548912	11.70104	7.021387	
El Salvador	Deaths	Male	2012	9.419886	12.93092	7.794684	
El Salvador	Deaths	Male	2013	9.721015	13.32724	8.192533	
El Salvador	Deaths	Male	2014	9.945437	13.7017	8.36625	
El Salvador	Deaths	Male	2015	9.95057	14.10195	8.415361	
El Salvador	Deaths	Male	2016	9.920876	14.28343	8.535855	
El Salvador	Deaths	Male	2017	10.31272	14.9631	8.969801	
El Salvador	Deaths	Male	2018	10.65168	15.48141	9.145443	

El Salvador	Deaths	Male	2019	11.21564	16.36612	9.556268	
El Salvador	Deaths	Male	2020	12.02303	17.54503	10.18574	
El Salvador	Deaths	Male	2021	12.94683	18.10279	10.92456	
El Salvador	Deaths	Male	2022	12.63176	18.64395	10.33009	
El Salvador	Deaths	Male	2023	12.90326	18.06121	10.22813	
El Salvador	Deaths	Female	2010	7.033375	8.480216	5.914442	
El Salvador	Deaths	Female	2011	7.150723	8.52401	6.021397	
El Salvador	Deaths	Female	2012	7.590992	9.163182	6.42725	
El Salvador	Deaths	Female	2013	7.866248	9.736837	6.666637	
El Salvador	Deaths	Female	2014	8.107102	9.987779	6.946201	
El Salvador	Deaths	Female	2015	8.183671	10.16391	7.044909	
El Salvador	Deaths	Female	2016	8.139676	10.34753	7.100867	
El Salvador	Deaths	Female	2017	8.302974	10.47599	7.22512	
El Salvador	Deaths	Female	2018	8.696445	10.84937	7.509472	
El Salvador	Deaths	Female	2019	9.244747	11.40814	7.947283	
El Salvador	Deaths	Female	2020	9.775859	12.23653	8.354995	
El Salvador	Deaths	Female	2021	10.83632	13.69147	9.254778	
El Salvador	Deaths	Female	2022	11.43825	14.24819	9.536655	
El Salvador	Deaths	Female	2023	12.12756	15.55903	10.00491	
El Salvador	DALYs	Male	2010	206.3516	273.4266	167.5844	
El Salvador	DALYs	Male	2011	202.8748	271.9235	167.0476	
El Salvador	DALYs	Male	2012	222.7968	299.408	185.3007	
El Salvador	DALYs	Male	2013	229.2723	311.4149	191.6951	
El Salvador	DALYs	Male	2014	234.5244	323.3229	196.6691	
El Salvador	DALYs	Male	2015	234.6599	328.8842	200.0094	
El Salvador	DALYs	Male	2016	236.0201	333.0357	203.6611	
El Salvador	DALYs	Male	2017	246.0364	348.3487	214.0394	
El Salvador	DALYs	Male	2018	253.1885	357.8842	217.9171	
El Salvador	DALYs	Male	2019	265.4667	377.1076	227.9444	
El Salvador	DALYs	Male	2020	282.9787	404.3884	241.4618	
El Salvador	DALYs	Male	2021	303.2158	426.0371	257.1883	
El Salvador	DALYs	Male	2022	294.7183	429.1829	242.9326	
El Salvador	DALYs	Male	2023	302.602	424.7407	236.3852	
El Salvador	DALYs	Female	2010	167.9513	199.3954	141.4453	
El Salvador	DALYs	Female	2011	169.6914	204.4474	143.151	
El Salvador	DALYs	Female	2012	179.4995	220.4006	152.5986	

El Salvador	DALYs	Female	2013	184.8482	234.1689	155.9532	
El Salvador	DALYs	Female	2014	189.6096	239.3946	162.102	
El Salvador	DALYs	Female	2015	191.1058	242.5156	162.2865	
El Salvador	DALYs	Female	2016	190.7401	246.0456	160.289	
El Salvador	DALYs	Female	2017	195.1896	249.7707	164.7437	
El Salvador	DALYs	Female	2018	204.5615	259.5417	173.4411	
El Salvador	DALYs	Female	2019	216.4205	269.6617	184.1345	
El Salvador	DALYs	Female	2020	228.1125	286.7534	193.6036	
El Salvador	DALYs	Female	2021	252.1596	315.675	212.5911	
El Salvador	DALYs	Female	2022	263.8117	333.8834	218.0784	
El Salvador	DALYs	Female	2023	280.1335	356.3669	227.5072	
Equatorial Guir	Incidence	Male	2010	5.654331	8.139726	2.848586	
Equatorial Guir	Incidence	Male	2011	5.470263	8.013942	2.806899	
Equatorial Guir	Incidence	Male	2012	5.173633	7.613887	2.662616	
Equatorial Guir	Incidence	Male	2013	5.083249	7.601479	2.570962	
Equatorial Guir	Incidence	Male	2014	5.071137	7.547891	2.586877	
Equatorial Guir	Incidence	Male	2015	5.056658	7.193904	2.585695	
Equatorial Guir	Incidence	Male	2016	4.996131	7.325957	2.516694	
Equatorial Guir	Incidence	Male	2017	4.967463	7.416889	2.42817	
Equatorial Guir	Incidence	Male	2018	4.963781	7.406137	2.439941	
Equatorial Guir	Incidence	Male	2019	4.985626	7.535336	2.443291	
Equatorial Guir	Incidence	Male	2020	5.2775	7.997171	2.527453	
Equatorial Guir	Incidence	Male	2021	5.600556	8.707624	2.605848	
Equatorial Guir	Incidence	Male	2022	6.152486	9.499883	2.956698	
Equatorial Guir	Incidence	Male	2023	6.490891	9.660928	2.96872	
Equatorial Guir	Incidence	Female	2010	2.502704	3.821359	1.365529	
Equatorial Guir	Incidence	Female	2011	2.497304	3.712063	1.330198	
Equatorial Guir	Incidence	Female	2012	2.714569	3.989664	1.52664	
Equatorial Guir	Incidence	Female	2013	2.810515	4.160155	1.536028	
Equatorial Guir	Incidence	Female	2014	2.982566	4.511127	1.604974	
Equatorial Guir	Incidence	Female	2015	2.913347	4.454003	1.547993	
Equatorial Guir	Incidence	Female	2016	2.911427	4.481567	1.522804	
Equatorial Guir	Incidence	Female	2017	3.175346	4.997179	1.67642	
Equatorial Guir	Incidence	Female	2018	3.270608	4.949602	1.751038	
Equatorial Guir	Incidence	Female	2019	3.278044	5.038995	1.833997	
Equatorial Guir	Incidence	Female	2020	3.278103	5.275521	1.780089	

Equatorial Guir	Incidence	Female	2021	3.670649	6.026981	1.996236	
Equatorial Guir	Incidence	Female	2022	4.200278	6.967441	2.229992	
Equatorial Guir	Incidence	Female	2023	4.531014	7.702125	2.362555	
Equatorial Guir	Deaths	Male	2010	5.502629	7.872547	2.81256	
Equatorial Guir	Deaths	Male	2011	5.337283	7.852338	2.736316	
Equatorial Guir	Deaths	Male	2012	5.060512	7.470477	2.604684	
Equatorial Guir	Deaths	Male	2013	4.967062	7.432018	2.53358	
Equatorial Guir	Deaths	Male	2014	4.946246	7.401982	2.520988	
Equatorial Guir	Deaths	Male	2015	4.938747	7.047161	2.494986	
Equatorial Guir	Deaths	Male	2016	4.883409	7.118194	2.473609	
Equatorial Guir	Deaths	Male	2017	4.847505	7.164191	2.3714	
Equatorial Guir	Deaths	Male	2018	4.836459	7.208535	2.374567	
Equatorial Guir	Deaths	Male	2019	4.847892	7.25446	2.380098	
Equatorial Guir	Deaths	Male	2020	5.123927	7.702942	2.427869	
Equatorial Guir	Deaths	Male	2021	5.403166	8.398673	2.524229	
Equatorial Guir	Deaths	Male	2022	5.906671	9.111772	2.831746	
Equatorial Guir	Deaths	Male	2023	6.186423	9.171095	2.861342	
Equatorial Guir	Deaths	Female	2010	2.513191	3.825247	1.367202	
Equatorial Guir	Deaths	Female	2011	2.515316	3.748328	1.337363	
Equatorial Guir	Deaths	Female	2012	2.722014	4.020406	1.524226	
Equatorial Guir	Deaths	Female	2013	2.802856	4.141317	1.521253	
Equatorial Guir	Deaths	Female	2014	2.957717	4.490398	1.583155	
Equatorial Guir	Deaths	Female	2015	2.901133	4.438681	1.535117	
Equatorial Guir	Deaths	Female	2016	2.902919	4.465068	1.511217	
Equatorial Guir	Deaths	Female	2017	3.149527	4.937241	1.676962	
Equatorial Guir	Deaths	Female	2018	3.236839	4.905968	1.746236	
Equatorial Guir	Deaths	Female	2019	3.242801	4.95496	1.801147	
Equatorial Guir	Deaths	Female	2020	3.236378	5.181267	1.73797	
Equatorial Guir	Deaths	Female	2021	3.606035	5.897536	1.944925	
Equatorial Guir	Deaths	Female	2022	4.096311	6.789231	2.160639	
Equatorial Guir	Deaths	Female	2023	4.393574	7.430212	2.233013	
Equatorial Guir	DALYs	Male	2010	165.8176	243.1149	85.16952	
Equatorial Guir	DALYs	Male	2011	159.437	232.2195	82.67983	
Equatorial Guir	DALYs	Male	2012	149.9282	220.0295	76.90411	
Equatorial Guir	DALYs	Male	2013	147.5781	220.2276	75.33589	
Equatorial Guir	DALYs	Male	2014	147.6588	218.9538	76.41069	

Equatorial Guir	DALYs	Male	2015	146.6465	209.9546	75.65653	
Equatorial Guir	DALYs	Male	2016	144.5706	216.2836	72.69024	
Equatorial Guir	DALYs	Male	2017	144.264	219.6905	70.98664	
Equatorial Guir	DALYs	Male	2018	144.4771	218.8551	70.90475	
Equatorial Guir	DALYs	Male	2019	145.5981	222.3538	71.58641	
Equatorial Guir	DALYs	Male	2020	154.1308	237.0494	74.04309	
Equatorial Guir	DALYs	Male	2021	165.4499	258.8347	76.85676	
Equatorial Guir	DALYs	Male	2022	183.2817	284.1063	86.01701	
Equatorial Guir	DALYs	Male	2023	195.7641	294.9174	88.09196	
Equatorial Guir	DALYs	Female	2010	68.23058	103.7167	39.04774	
Equatorial Guir	DALYs	Female	2011	67.56365	101.3767	37.72208	
Equatorial Guir	DALYs	Female	2012	73.91604	110.3496	42.10952	
Equatorial Guir	DALYs	Female	2013	77.52807	116.1208	43.47245	
Equatorial Guir	DALYs	Female	2014	83.13987	127.5354	45.03809	
Equatorial Guir	DALYs	Female	2015	80.31357	124.5442	43.81483	
Equatorial Guir	DALYs	Female	2016	80.01199	125.1233	42.77819	
Equatorial Guir	DALYs	Female	2017	88.09819	138.1524	47.01096	
Equatorial Guir	DALYs	Female	2018	91.10346	141.0336	49.6532	
Equatorial Guir	DALYs	Female	2019	91.28478	143.7767	51.61644	
Equatorial Guir	DALYs	Female	2020	91.49513	148.6668	51.15808	
Equatorial Guir	DALYs	Female	2021	103.0834	170.3186	57.66898	
Equatorial Guir	DALYs	Female	2022	119.5437	194.8345	64.1415	
Equatorial Guir	DALYs	Female	2023	130.1288	220.6112	68.95016	
Eritrea	Incidence	Male	2010	2.180298	3.192173	1.299233	
Eritrea	Incidence	Male	2011	2.234781	3.218086	1.329202	
Eritrea	Incidence	Male	2012	2.332326	3.23157	1.381428	
Eritrea	Incidence	Male	2013	2.409643	3.314987	1.419021	
Eritrea	Incidence	Male	2014	2.426345	3.473349	1.42711	
Eritrea	Incidence	Male	2015	2.565863	3.634487	1.518147	
Eritrea	Incidence	Male	2016	2.639824	3.82404	1.545051	
Eritrea	Incidence	Male	2017	2.728317	4.073529	1.580983	
Eritrea	Incidence	Male	2018	2.820434	4.288074	1.631179	
Eritrea	Incidence	Male	2019	2.864004	4.355054	1.666216	
Eritrea	Incidence	Male	2020	2.989345	4.485029	1.714339	
Eritrea	Incidence	Male	2021	3.075947	4.552201	1.744801	
Eritrea	Incidence	Male	2022	3.735034	5.680277	2.158024	

Eritrea	Incidence	Male	2023	3.633126	5.424895	2.177249	
Eritrea	Incidence	Female	2010	1.545364	2.237338	0.92355	
Eritrea	Incidence	Female	2011	1.591131	2.305185	0.93701	
Eritrea	Incidence	Female	2012	1.647675	2.370892	0.950775	
Eritrea	Incidence	Female	2013	1.682224	2.397999	1.0042	
Eritrea	Incidence	Female	2014	1.707669	2.4591	1.04733	
Eritrea	Incidence	Female	2015	1.778554	2.479816	1.1085	
Eritrea	Incidence	Female	2016	1.823844	2.531454	1.145161	
Eritrea	Incidence	Female	2017	1.886991	2.653978	1.200195	
Eritrea	Incidence	Female	2018	1.961463	2.71549	1.264863	
Eritrea	Incidence	Female	2019	2.009037	2.774833	1.319697	
Eritrea	Incidence	Female	2020	2.030741	2.804553	1.346386	
Eritrea	Incidence	Female	2021	2.011702	2.783072	1.315062	
Eritrea	Incidence	Female	2022	2.361545	3.296436	1.576956	
Eritrea	Incidence	Female	2023	2.448306	3.5507	1.547167	
Eritrea	Deaths	Male	2010	2.101631	3.067537	1.256698	
Eritrea	Deaths	Male	2011	2.153091	3.086806	1.284537	
Eritrea	Deaths	Male	2012	2.244846	3.113281	1.329565	
Eritrea	Deaths	Male	2013	2.317647	3.16977	1.364636	
Eritrea	Deaths	Male	2014	2.332644	3.342446	1.357234	
Eritrea	Deaths	Male	2015	2.466098	3.498152	1.463323	
Eritrea	Deaths	Male	2016	2.537638	3.697482	1.477096	
Eritrea	Deaths	Male	2017	2.623663	3.941692	1.518461	
Eritrea	Deaths	Male	2018	2.711867	4.135196	1.558813	
Eritrea	Deaths	Male	2019	2.752873	4.182975	1.59488	
Eritrea	Deaths	Male	2020	2.8778	4.305194	1.647983	
Eritrea	Deaths	Male	2021	2.96918	4.38513	1.687625	
Eritrea	Deaths	Male	2022	3.62361	5.493756	2.08705	
Eritrea	Deaths	Male	2023	3.491274	5.200927	2.08614	
Eritrea	Deaths	Female	2010	1.465332	2.129189	0.872307	
Eritrea	Deaths	Female	2011	1.507285	2.183591	0.884022	
Eritrea	Deaths	Female	2012	1.559377	2.242327	0.898973	
Eritrea	Deaths	Female	2013	1.591056	2.25139	0.953645	
Eritrea	Deaths	Female	2014	1.613774	2.327773	0.99547	
Eritrea	Deaths	Female	2015	1.679706	2.325547	1.05291	
Eritrea	Deaths	Female	2016	1.722826	2.396256	1.08702	

Eritrea	Deaths	Female	2017	1.781777	2.504397	1.136333	
Eritrea	Deaths	Female	2018	1.850466	2.543171	1.195428	
Eritrea	Deaths	Female	2019	1.894922	2.612968	1.2279	
Eritrea	Deaths	Female	2020	1.913674	2.640628	1.24829	
Eritrea	Deaths	Female	2021	1.897546	2.625618	1.233947	
Eritrea	Deaths	Female	2022	2.225783	3.095334	1.453341	
Eritrea	Deaths	Female	2023	2.306574	3.34897	1.446173	
Eritrea	DALYs	Male	2010	68.62184	101.5065	40.94145	
Eritrea	DALYs	Male	2011	70.45527	103.1238	41.5727	
Eritrea	DALYs	Male	2012	73.67689	102.6928	43.42272	
Eritrea	DALYs	Male	2013	76.22804	107.0606	44.84206	
Eritrea	DALYs	Male	2014	76.77376	109.2118	45.04157	
Eritrea	DALYs	Male	2015	81.21319	115.2794	48.2457	
Eritrea	DALYs	Male	2016	83.51876	119.7335	49.00298	
Eritrea	DALYs	Male	2017	86.23503	129.8958	50.10072	
Eritrea	DALYs	Male	2018	89.11102	136.0551	52.22364	
Eritrea	DALYs	Male	2019	90.43527	139.315	52.74095	
Eritrea	DALYs	Male	2020	94.01819	142.4615	54.36879	
Eritrea	DALYs	Male	2021	96.03392	140.6301	54.93849	
Eritrea	DALYs	Male	2022	114.9019	174.7349	67.30469	
Eritrea	DALYs	Male	2023	113.6257	171.9332	67.93596	
Eritrea	DALYs	Female	2010	49.68919	73.0003	29.86874	
Eritrea	DALYs	Female	2011	51.25094	74.31253	30.38742	
Eritrea	DALYs	Female	2012	53.16449	77.26669	31.11706	
Eritrea	DALYs	Female	2013	54.33374	76.72559	32.60485	
Eritrea	DALYs	Female	2014	55.21293	79.88755	33.18709	
Eritrea	DALYs	Female	2015	57.55782	80.99442	35.06016	
Eritrea	DALYs	Female	2016	58.98882	81.64691	36.42746	
Eritrea	DALYs	Female	2017	61.066	86.25956	38.37941	
Eritrea	DALYs	Female	2018	63.54924	89.6817	40.82855	
Eritrea	DALYs	Female	2019	65.0414	89.58718	43.0365	
Eritrea	DALYs	Female	2020	65.82976	89.55262	44.78053	
Eritrea	DALYs	Female	2021	65.08375	89.78236	42.81168	
Eritrea	DALYs	Female	2022	76.18823	108.3678	51.52503	
Eritrea	DALYs	Female	2023	79.01772	114.3725	50.02744	
Estonia	Incidence	Male	2010	95.75331	102.616	90.22149	

Estonia	Incidence	Male	2011	94.82977	101.9426	89.35626	
Estonia	Incidence	Male	2012	95.65029	103.035	89.33536	
Estonia	Incidence	Male	2013	95.15705	102.6322	88.53281	
Estonia	Incidence	Male	2014	99.23094	107.4189	92.12112	
Estonia	Incidence	Male	2015	95.6971	103.9246	88.43769	
Estonia	Incidence	Male	2016	93.38825	101.3917	86.42298	
Estonia	Incidence	Male	2017	91.81346	100.3942	84.21263	
Estonia	Incidence	Male	2018	93.53555	102.0164	85.37096	
Estonia	Incidence	Male	2019	90.32094	98.29986	82.71674	
Estonia	Incidence	Male	2020	87.89606	96.12127	80.6466	
Estonia	Incidence	Male	2021	87.45948	95.63142	79.87196	
Estonia	Incidence	Male	2022	80.64097	88.27882	73.68798	
Estonia	Incidence	Male	2023	80.80526	89.52508	73.82434	
Estonia	Incidence	Female	2010	21.94178	25.06856	19.45701	
Estonia	Incidence	Female	2011	23.6222	27.14738	20.89012	
Estonia	Incidence	Female	2012	25.23263	29.11166	22.05689	
Estonia	Incidence	Female	2013	26.49385	30.63038	23.0488	
Estonia	Incidence	Female	2014	26.95106	31.0823	23.68449	
Estonia	Incidence	Female	2015	26.44737	30.63161	23.37213	
Estonia	Incidence	Female	2016	27.70699	31.97504	24.51036	
Estonia	Incidence	Female	2017	26.91508	31.25192	23.86724	
Estonia	Incidence	Female	2018	26.57901	30.73082	23.24303	
Estonia	Incidence	Female	2019	26.75194	30.90036	23.286	
Estonia	Incidence	Female	2020	24.44444	28.03615	21.17462	
Estonia	Incidence	Female	2021	27.08878	30.82181	23.50214	
Estonia	Incidence	Female	2022	28.32789	32.64837	24.43209	
Estonia	Incidence	Female	2023	27.89121	32.95467	23.1918	
Estonia	Deaths	Male	2010	86.43249	90.75683	81.73095	
Estonia	Deaths	Male	2011	85.12198	89.65345	80.63765	
Estonia	Deaths	Male	2012	85.33793	90.4389	80.82913	
Estonia	Deaths	Male	2013	84.37932	90.13038	79.82908	
Estonia	Deaths	Male	2014	87.75476	93.18993	82.97186	
Estonia	Deaths	Male	2015	84.45436	89.07945	80.04407	
Estonia	Deaths	Male	2016	82.21385	86.46393	77.81188	
Estonia	Deaths	Male	2017	80.49664	84.60326	76.03458	
Estonia	Deaths	Male	2018	81.62481	86.18206	76.9134	

Estonia	Deaths	Male	2019	78.47018	83.07585	73.54367	
Estonia	Deaths	Male	2020	76.12988	80.231	71.90601	
Estonia	Deaths	Male	2021	75.64421	80.07831	71.62043	
Estonia	Deaths	Male	2022	69.68069	74.80818	64.90249	
Estonia	Deaths	Male	2023	69.79998	75.67009	64.90234	
Estonia	Deaths	Female	2010	19.67369	22.03866	17.65526	
Estonia	Deaths	Female	2011	21.02713	23.85207	18.86626	
Estonia	Deaths	Female	2012	22.34332	25.41926	20.01629	
Estonia	Deaths	Female	2013	23.29598	26.55293	20.77681	
Estonia	Deaths	Female	2014	23.74416	26.91744	21.42034	
Estonia	Deaths	Female	2015	23.30148	26.37133	21.2193	
Estonia	Deaths	Female	2016	24.37517	27.33665	22.30167	
Estonia	Deaths	Female	2017	23.5954	26.48046	21.3922	
Estonia	Deaths	Female	2018	23.27747	26.4574	21.06737	
Estonia	Deaths	Female	2019	23.35581	26.49642	20.90309	
Estonia	Deaths	Female	2020	21.2811	24.06511	18.89003	
Estonia	Deaths	Female	2021	23.39843	26.35455	20.82965	
Estonia	Deaths	Female	2022	24.5367	27.81285	21.56933	
Estonia	Deaths	Female	2023	24.12571	28.04656	20.65957	
Estonia	DALYs	Male	2010	2045.277	2139.832	1940.721	
Estonia	DALYs	Male	2011	2001.498	2098.733	1905.428	
Estonia	DALYs	Male	2012	1997.689	2107.887	1892.763	
Estonia	DALYs	Male	2013	1957.794	2077.803	1854.824	
Estonia	DALYs	Male	2014	2005.865	2125.816	1902.215	
Estonia	DALYs	Male	2015	1918.782	2017.706	1813.763	
Estonia	DALYs	Male	2016	1862.165	1949.258	1765.673	
Estonia	DALYs	Male	2017	1807.609	1894.823	1710.194	
Estonia	DALYs	Male	2018	1834.399	1927.834	1731.269	
Estonia	DALYs	Male	2019	1771.055	1858.379	1664.707	
Estonia	DALYs	Male	2020	1719.604	1802.257	1621.582	
Estonia	DALYs	Male	2021	1691.122	1784.192	1601.548	
Estonia	DALYs	Male	2022	1541.344	1651.806	1441.081	
Estonia	DALYs	Male	2023	1528.893	1658.067	1431.872	
Estonia	DALYs	Female	2010	421.4369	476.9596	378.6619	
Estonia	DALYs	Female	2011	445.4993	505.3777	398.2715	
Estonia	DALYs	Female	2012	465.3698	531.8405	413.6692	

Estonia	DALYs	Female	2013	482.4042	551.7601	428.1329	
Estonia	DALYs	Female	2014	484.2834	554.9609	438.8651	
Estonia	DALYs	Female	2015	471.4739	538.5194	429.7192	
Estonia	DALYs	Female	2016	488.1677	553.7237	447.1549	
Estonia	DALYs	Female	2017	469.0793	532.9866	428.0812	
Estonia	DALYs	Female	2018	457.6645	526.0701	415.9935	
Estonia	DALYs	Female	2019	454.4953	516.8824	410.4609	
Estonia	DALYs	Female	2020	415.5752	466.6878	377.6337	
Estonia	DALYs	Female	2021	455.6563	512.378	414.5629	
Estonia	DALYs	Female	2022	472.0179	534.9899	423.1077	
Estonia	DALYs	Female	2023	461.1968	530.1118	402.3304	
Eswatini	Incidence	Male	2010	2.453846	3.56745	1.57563	
Eswatini	Incidence	Male	2011	2.310651	3.319931	1.518625	
Eswatini	Incidence	Male	2012	2.22032	3.20458	1.458373	
Eswatini	Incidence	Male	2013	2.107392	3.008754	1.370355	
Eswatini	Incidence	Male	2014	1.959391	2.719617	1.298635	
Eswatini	Incidence	Male	2015	1.972719	2.779915	1.298683	
Eswatini	Incidence	Male	2016	1.887354	2.673417	1.224535	
Eswatini	Incidence	Male	2017	1.939413	2.835895	1.284677	
Eswatini	Incidence	Male	2018	2.063013	3.054661	1.366799	
Eswatini	Incidence	Male	2019	2.167364	3.262345	1.454364	
Eswatini	Incidence	Male	2020	2.337369	3.518148	1.596255	
Eswatini	Incidence	Male	2021	2.589918	3.876701	1.73529	
Eswatini	Incidence	Male	2022	2.569593	3.883591	1.695677	
Eswatini	Incidence	Male	2023	2.615719	3.780732	1.755638	
Eswatini	Incidence	Female	2010	1.332398	1.87131	0.839962	
Eswatini	Incidence	Female	2011	1.358999	1.911197	0.86062	
Eswatini	Incidence	Female	2012	1.366215	1.90519	0.823259	
Eswatini	Incidence	Female	2013	1.356756	1.923889	0.800636	
Eswatini	Incidence	Female	2014	1.3352	1.869168	0.774854	
Eswatini	Incidence	Female	2015	1.337406	1.880248	0.788098	
Eswatini	Incidence	Female	2016	1.354567	1.950401	0.81226	
Eswatini	Incidence	Female	2017	1.41246	1.999346	0.885348	
Eswatini	Incidence	Female	2018	1.47405	2.088094	0.947038	
Eswatini	Incidence	Female	2019	1.542001	2.170888	1.015763	
Eswatini	Incidence	Female	2020	1.772496	2.437764	1.223754	

Eswatini	Incidence	Female	2021	1.967402	2.615829	1.299788	
Eswatini	Incidence	Female	2022	1.932246	2.707778	1.296829	
Eswatini	Incidence	Female	2023	1.941077	2.737829	1.293513	
Eswatini	Deaths	Male	2010	2.316276	3.360859	1.49821	
Eswatini	Deaths	Male	2011	2.189099	3.107557	1.444616	
Eswatini	Deaths	Male	2012	2.110827	3.03176	1.386278	
Eswatini	Deaths	Male	2013	2.011861	2.837894	1.317785	
Eswatini	Deaths	Male	2014	1.879453	2.591256	1.257493	
Eswatini	Deaths	Male	2015	1.900988	2.646422	1.258749	
Eswatini	Deaths	Male	2016	1.825816	2.553949	1.190254	
Eswatini	Deaths	Male	2017	1.878352	2.725968	1.256885	
Eswatini	Deaths	Male	2018	1.997295	2.942013	1.335506	
Eswatini	Deaths	Male	2019	2.098288	3.126421	1.418383	
Eswatini	Deaths	Male	2020	2.272874	3.413304	1.550927	
Eswatini	Deaths	Male	2021	2.522716	3.711094	1.71519	
Eswatini	Deaths	Male	2022	2.499782	3.739925	1.682014	
Eswatini	Deaths	Male	2023	2.541277	3.698079	1.727513	
Eswatini	Deaths	Female	2010	1.225257	1.718741	0.782183	
Eswatini	Deaths	Female	2011	1.25252	1.749836	0.804162	
Eswatini	Deaths	Female	2012	1.262523	1.750672	0.766368	
Eswatini	Deaths	Female	2013	1.257903	1.779699	0.746487	
Eswatini	Deaths	Female	2014	1.242242	1.742701	0.725048	
Eswatini	Deaths	Female	2015	1.247858	1.755464	0.736999	
Eswatini	Deaths	Female	2016	1.26587	1.81706	0.759659	
Eswatini	Deaths	Female	2017	1.320239	1.854154	0.833153	
Eswatini	Deaths	Female	2018	1.377455	1.953853	0.882132	
Eswatini	Deaths	Female	2019	1.439947	2.016671	0.946925	
Eswatini	Deaths	Female	2020	1.65711	2.282942	1.149287	
Eswatini	Deaths	Female	2021	1.839486	2.433414	1.22326	
Eswatini	Deaths	Female	2022	1.797746	2.505184	1.210832	
Eswatini	Deaths	Female	2023	1.804842	2.528893	1.195611	
Eswatini	DALYs	Male	2010	77.25703	113.1759	49.70228	
Eswatini	DALYs	Male	2011	72.09587	105.9632	46.30879	
Eswatini	DALYs	Male	2012	68.69711	99.96473	44.80478	
Eswatini	DALYs	Male	2013	64.55514	93.41537	41.31032	
Eswatini	DALYs	Male	2014	59.37878	83.55436	38.52954	

Eswatini	DALYs	Male	2015	59.17041	84.33667	38.69452	
Eswatini	DALYs	Male	2016	56.10826	81.36103	36.38701	
Eswatini	DALYs	Male	2017	57.44943	85.10761	36.40128	
Eswatini	DALYs	Male	2018	61.07451	91.49931	39.95986	
Eswatini	DALYs	Male	2019	64.08713	97.33409	42.34484	
Eswatini	DALYs	Male	2020	68.37775	103.5188	45.5673	
Eswatini	DALYs	Male	2021	75.4381	111.953	50.30032	
Eswatini	DALYs	Male	2022	74.88953	114.7379	48.51804	
Eswatini	DALYs	Male	2023	76.33365	110.8957	51.40833	
Eswatini	DALYs	Female	2010	46.30812	66.08113	28.68087	
Eswatini	DALYs	Female	2011	46.89819	66.12446	29.06298	
Eswatini	DALYs	Female	2012	46.77683	65.88102	27.69865	
Eswatini	DALYs	Female	2013	46.05572	67.27967	26.67561	
Eswatini	DALYs	Female	2014	44.93262	64.05158	25.67871	
Eswatini	DALYs	Female	2015	44.63301	63.68152	25.89033	
Eswatini	DALYs	Female	2016	44.93788	66.08045	26.11628	
Eswatini	DALYs	Female	2017	46.70939	67.72046	28.26639	
Eswatini	DALYs	Female	2018	48.63897	69.42354	31.06606	
Eswatini	DALYs	Female	2019	50.81902	73.78214	33.20812	
Eswatini	DALYs	Female	2020	57.77222	82.50712	38.56625	
Eswatini	DALYs	Female	2021	63.65068	85.3354	41.39015	
Eswatini	DALYs	Female	2022	63.48444	89.11751	41.43034	
Eswatini	DALYs	Female	2023	63.87506	91.23533	42.25574	
Ethiopia	Incidence	Male	2010	4.357541	6.163964	3.223611	
Ethiopia	Incidence	Male	2011	4.555434	6.195887	3.355048	
Ethiopia	Incidence	Male	2012	4.598742	6.260044	3.400273	
Ethiopia	Incidence	Male	2013	4.732348	6.489463	3.446353	
Ethiopia	Incidence	Male	2014	4.765603	6.573395	3.408969	
Ethiopia	Incidence	Male	2015	4.732083	6.623003	3.450934	
Ethiopia	Incidence	Male	2016	4.81706	6.794739	3.397976	
Ethiopia	Incidence	Male	2017	4.948067	6.929416	3.540752	
Ethiopia	Incidence	Male	2018	5.097439	7.137946	3.516501	
Ethiopia	Incidence	Male	2019	5.307545	7.421302	3.686399	
Ethiopia	Incidence	Male	2020	5.438572	7.667845	3.799862	
Ethiopia	Incidence	Male	2021	6.147674	8.674772	4.336881	
Ethiopia	Incidence	Male	2022	6.339057	9.178951	4.510678	

Ethiopia	Incidence	Male	2023	6.841586	9.633496	4.767893	
Ethiopia	Incidence	Female	2010	0.908311	1.272034	0.661571	
Ethiopia	Incidence	Female	2011	0.955725	1.318637	0.689103	
Ethiopia	Incidence	Female	2012	0.981247	1.35238	0.682058	
Ethiopia	Incidence	Female	2013	1.010043	1.416179	0.686998	
Ethiopia	Incidence	Female	2014	1.018692	1.400462	0.684507	
Ethiopia	Incidence	Female	2015	1.035486	1.419442	0.700132	
Ethiopia	Incidence	Female	2016	1.089124	1.451148	0.730514	
Ethiopia	Incidence	Female	2017	1.136114	1.519412	0.786023	
Ethiopia	Incidence	Female	2018	1.195755	1.560467	0.838997	
Ethiopia	Incidence	Female	2019	1.262006	1.661828	0.87991	
Ethiopia	Incidence	Female	2020	1.26511	1.66569	0.892352	
Ethiopia	Incidence	Female	2021	1.34505	1.787845	0.941756	
Ethiopia	Incidence	Female	2022	1.632143	2.185141	1.134305	
Ethiopia	Incidence	Female	2023	1.78226	2.355303	1.208842	
Ethiopia	Deaths	Male	2010	4.339892	6.172145	3.197753	
Ethiopia	Deaths	Male	2011	4.537532	6.174823	3.32899	
Ethiopia	Deaths	Male	2012	4.583404	6.148062	3.383777	
Ethiopia	Deaths	Male	2013	4.719502	6.37961	3.443243	
Ethiopia	Deaths	Male	2014	4.753414	6.477288	3.427232	
Ethiopia	Deaths	Male	2015	4.723075	6.580751	3.455526	
Ethiopia	Deaths	Male	2016	4.801476	6.729951	3.413133	
Ethiopia	Deaths	Male	2017	4.922865	6.789451	3.531806	
Ethiopia	Deaths	Male	2018	5.059382	7.03671	3.499147	
Ethiopia	Deaths	Male	2019	5.262397	7.379033	3.662731	
Ethiopia	Deaths	Male	2020	5.414082	7.562828	3.798313	
Ethiopia	Deaths	Male	2021	6.134809	8.682132	4.352933	
Ethiopia	Deaths	Male	2022	6.255161	9.093254	4.472846	
Ethiopia	Deaths	Male	2023	6.730627	9.368191	4.721994	
Ethiopia	Deaths	Female	2010	0.888983	1.257022	0.646781	
Ethiopia	Deaths	Female	2011	0.935149	1.307011	0.676994	
Ethiopia	Deaths	Female	2012	0.960435	1.326164	0.67053	
Ethiopia	Deaths	Female	2013	0.989276	1.384044	0.676147	
Ethiopia	Deaths	Female	2014	0.996976	1.371593	0.673143	
Ethiopia	Deaths	Female	2015	1.012004	1.384833	0.67879	
Ethiopia	Deaths	Female	2016	1.063596	1.422343	0.71463	

Ethiopia	Deaths	Female	2017	1.107921	1.490152	0.767467	
Ethiopia	Deaths	Female	2018	1.163944	1.531047	0.817618	
Ethiopia	Deaths	Female	2019	1.225782	1.616696	0.860333	
Ethiopia	Deaths	Female	2020	1.226453	1.620339	0.865984	
Ethiopia	Deaths	Female	2021	1.300242	1.732811	0.911836	
Ethiopia	Deaths	Female	2022	1.568593	2.108005	1.088795	
Ethiopia	Deaths	Female	2023	1.707258	2.264264	1.157739	
Ethiopia	DALYs	Male	2010	124.9647	175.4759	92.30312	
Ethiopia	DALYs	Male	2011	130.4921	179.2675	97.44903	
Ethiopia	DALYs	Male	2012	131.4736	182.1662	96.9083	
Ethiopia	DALYs	Male	2013	135.1444	188.2878	98.95141	
Ethiopia	DALYs	Male	2014	136.0649	189.5665	96.88163	
Ethiopia	DALYs	Male	2015	134.8749	189.662	97.23393	
Ethiopia	DALYs	Male	2016	137.5624	191.0023	95.99361	
Ethiopia	DALYs	Male	2017	141.7656	201.2129	100.8036	
Ethiopia	DALYs	Male	2018	146.5875	204.8668	101.0653	
Ethiopia	DALYs	Male	2019	152.8773	216.4239	105.7122	
Ethiopia	DALYs	Male	2020	155.614	221.6715	108.325	
Ethiopia	DALYs	Male	2021	174.3843	247.2949	121.7155	
Ethiopia	DALYs	Male	2022	183.5748	264.7065	128.7197	
Ethiopia	DALYs	Male	2023	199.1564	275.3908	138.6476	
Ethiopia	DALYs	Female	2010	26.97984	37.85351	19.50582	
Ethiopia	DALYs	Female	2011	28.33543	38.85804	19.98117	
Ethiopia	DALYs	Female	2012	29.02919	40.22331	20.05064	
Ethiopia	DALYs	Female	2013	29.78883	41.62492	20.2435	
Ethiopia	DALYs	Female	2014	30.0385	41.40329	20.19306	
Ethiopia	DALYs	Female	2015	30.58597	41.66073	20.47323	
Ethiopia	DALYs	Female	2016	32.20031	43.08553	21.64037	
Ethiopia	DALYs	Female	2017	33.66741	44.62158	23.18949	
Ethiopia	DALYs	Female	2018	35.54374	46.14694	24.82048	
Ethiopia	DALYs	Female	2019	37.64901	49.85876	26.20419	
Ethiopia	DALYs	Female	2020	37.84079	50.94319	26.59436	
Ethiopia	DALYs	Female	2021	40.34781	53.36148	28.47832	
Ethiopia	DALYs	Female	2022	49.46248	66.65856	34.08275	
Ethiopia	DALYs	Female	2023	54.4518	72.58369	37.2426	
Fiji	Incidence	Male	2010	9.187447	12.38786	7.125353	

Fiji	Incidence	Male	2011	9.288179	12.62924	7.259693	
Fiji	Incidence	Male	2012	9.34483	12.7225	7.302154	
Fiji	Incidence	Male	2013	9.4711	12.82301	7.225847	
Fiji	Incidence	Male	2014	9.686532	13.35829	7.177162	
Fiji	Incidence	Male	2015	9.956168	14.0082	7.276305	
Fiji	Incidence	Male	2016	10.1766	14.36504	7.137388	
Fiji	Incidence	Male	2017	10.38422	14.88802	7.371814	
Fiji	Incidence	Male	2018	10.69265	15.76515	7.612283	
Fiji	Incidence	Male	2019	10.91262	16.11391	7.628119	
Fiji	Incidence	Male	2020	11.18154	16.64425	7.879608	
Fiji	Incidence	Male	2021	11.7199	17.371	8.356845	
Fiji	Incidence	Male	2022	12.36882	18.36577	8.851496	
Fiji	Incidence	Male	2023	13.0449	19.14396	9.279439	
Fiji	Incidence	Female	2010	5.403445	7.406701	3.993326	
Fiji	Incidence	Female	2011	5.565882	7.687537	4.026126	
Fiji	Incidence	Female	2012	5.653205	8.0263	3.997783	
Fiji	Incidence	Female	2013	5.758082	8.124521	3.943824	
Fiji	Incidence	Female	2014	5.876495	8.138601	3.915108	
Fiji	Incidence	Female	2015	6.062708	8.218825	4.066646	
Fiji	Incidence	Female	2016	6.225612	8.426313	4.123191	
Fiji	Incidence	Female	2017	6.364064	8.623495	4.197153	
Fiji	Incidence	Female	2018	6.602593	8.891893	4.514866	
Fiji	Incidence	Female	2019	6.777094	9.305619	4.611042	
Fiji	Incidence	Female	2020	6.966976	9.590468	4.774824	
Fiji	Incidence	Female	2021	7.068952	9.668994	4.875481	
Fiji	Incidence	Female	2022	7.663405	10.29255	5.252141	
Fiji	Incidence	Female	2023	8.062693	10.92632	5.416595	
Fiji	Deaths	Male	2010	9.091908	12.23493	7.030719	
Fiji	Deaths	Male	2011	9.189406	12.4667	7.191491	
Fiji	Deaths	Male	2012	9.249672	12.5903	7.235513	
Fiji	Deaths	Male	2013	9.377775	12.67265	7.133678	
Fiji	Deaths	Male	2014	9.597716	13.22205	7.120983	
Fiji	Deaths	Male	2015	9.869856	13.8825	7.189965	
Fiji	Deaths	Male	2016	10.09757	14.24798	7.06211	
Fiji	Deaths	Male	2017	10.31451	14.79659	7.271788	
Fiji	Deaths	Male	2018	10.63375	15.69031	7.513099	

Fiji	Deaths	Male	2019	10.86037	16.0442	7.602426	
Fiji	Deaths	Male	2020	11.13361	16.61359	7.837101	
Fiji	Deaths	Male	2021	11.6766	17.31818	8.298651	
Fiji	Deaths	Male	2022	12.33677	18.34627	8.786305	
Fiji	Deaths	Male	2023	13.03645	19.13327	9.272377	
Fiji	Deaths	Female	2010	5.31407	7.297312	3.941989	
Fiji	Deaths	Female	2011	5.473077	7.588075	3.961929	
Fiji	Deaths	Female	2012	5.566633	7.960551	3.937041	
Fiji	Deaths	Female	2013	5.674211	8.006091	3.892373	
Fiji	Deaths	Female	2014	5.792747	8.015309	3.843943	
Fiji	Deaths	Female	2015	5.978483	8.097713	4.011602	
Fiji	Deaths	Female	2016	6.143129	8.340709	4.068673	
Fiji	Deaths	Female	2017	6.284352	8.507437	4.146969	
Fiji	Deaths	Female	2018	6.519699	8.817028	4.470933	
Fiji	Deaths	Female	2019	6.69675	9.147593	4.583021	
Fiji	Deaths	Female	2020	6.891556	9.47401	4.717381	
Fiji	Deaths	Female	2021	6.996301	9.555522	4.831099	
Fiji	Deaths	Female	2022	7.588422	10.1836	5.216603	
Fiji	Deaths	Female	2023	7.994799	10.90817	5.394761	
Fiji	DALYs	Male	2010	255.5495	346.1486	198.19	
Fiji	DALYs	Male	2011	258.2771	354.051	202.4857	
Fiji	DALYs	Male	2012	259.5374	353.8762	201.7751	
Fiji	DALYs	Male	2013	262.6252	356.4812	200.2811	
Fiji	DALYs	Male	2014	267.8683	371.543	196.9215	
Fiji	DALYs	Male	2015	274.6393	391.0305	199.404	
Fiji	DALYs	Male	2016	279.8381	399.3934	196.7746	
Fiji	DALYs	Male	2017	284.6661	411.3741	202.579	
Fiji	DALYs	Male	2018	291.9262	430.6368	208.8209	
Fiji	DALYs	Male	2019	296.9509	442.1783	207.9961	
Fiji	DALYs	Male	2020	303.3879	454.546	214.3177	
Fiji	DALYs	Male	2021	316.3864	471.1461	224.8586	
Fiji	DALYs	Male	2022	332.7613	491.4752	239.0257	
Fiji	DALYs	Male	2023	349.0736	516.5413	246.3056	
Fiji	DALYs	Female	2010	147.1332	200.4142	108.6787	
Fiji	DALYs	Female	2011	151.4531	209.0669	109.1055	
Fiji	DALYs	Female	2012	153.4098	218.1658	108.906	

Fiji	DALYs	Female	2013	155.8885	219.6145	107.3538	
Fiji	DALYs	Female	2014	158.7652	220.4093	106.1483	
Fiji	DALYs	Female	2015	163.3045	222.2041	108.3965	
Fiji	DALYs	Female	2016	167.0142	226.5703	109.4066	
Fiji	DALYs	Female	2017	170.1327	229.1615	111.5713	
Fiji	DALYs	Female	2018	175.9933	237.5056	120.6559	
Fiji	DALYs	Female	2019	179.8473	247.0933	122.2158	
Fiji	DALYs	Female	2020	184.1015	253.3196	126.3581	
Fiji	DALYs	Female	2021	186.2249	254.1034	129.1388	
Fiji	DALYs	Female	2022	201.2326	272.7186	138.6622	
Fiji	DALYs	Female	2023	210.8344	286.85	142.6913	
Finland	Incidence	Male	2010	74.84517	79.79585	70.01332	
Finland	Incidence	Male	2011	73.60006	78.74841	68.44918	
Finland	Incidence	Male	2012	73.26585	79.53665	67.99284	
Finland	Incidence	Male	2013	74.27909	80.36625	68.8484	
Finland	Incidence	Male	2014	73.37913	80.2512	67.59953	
Finland	Incidence	Male	2015	73.78243	80.82101	67.62684	
Finland	Incidence	Male	2016	74.92222	82.48011	68.59462	
Finland	Incidence	Male	2017	74.95172	82.26401	67.64996	
Finland	Incidence	Male	2018	74.21519	82.16271	66.51535	
Finland	Incidence	Male	2019	74.50348	82.86355	66.75877	
Finland	Incidence	Male	2020	72.86583	81.10271	64.57665	
Finland	Incidence	Male	2021	76.51687	85.5477	68.05254	
Finland	Incidence	Male	2022	75.53981	84.73074	67.51415	
Finland	Incidence	Male	2023	79.95735	89.92126	70.74275	
Finland	Incidence	Female	2010	35.14373	39.2944	31.63094	
Finland	Incidence	Female	2011	36.51062	40.74244	32.30378	
Finland	Incidence	Female	2012	38.70603	43.29623	34.13498	
Finland	Incidence	Female	2013	39.68377	44.72013	35.23853	
Finland	Incidence	Female	2014	41.45427	46.57385	36.32784	
Finland	Incidence	Female	2015	41.82744	46.81831	36.88084	
Finland	Incidence	Female	2016	42.10666	47.22627	36.82263	
Finland	Incidence	Female	2017	42.23366	47.58527	36.69234	
Finland	Incidence	Female	2018	43.2619	48.95086	36.89075	
Finland	Incidence	Female	2019	44.65765	50.87044	38.09238	
Finland	Incidence	Female	2020	46.56869	53.47508	39.44251	

Finland	Incidence	Female	2021	47.7571	54.35705	40.76289	
Finland	Incidence	Female	2022	48.96537	55.69988	41.81262	
Finland	Incidence	Female	2023	51.91867	60.03856	44.12748	
Finland	Deaths	Male	2010	62.51912	65.51384	58.88161	
Finland	Deaths	Male	2011	61.29255	64.24493	57.77019	
Finland	Deaths	Male	2012	60.85072	64.19808	57.28846	
Finland	Deaths	Male	2013	61.60129	65.30763	58.10443	
Finland	Deaths	Male	2014	60.89282	64.18967	57.58918	
Finland	Deaths	Male	2015	61.12187	64.83316	57.37501	
Finland	Deaths	Male	2016	62.24258	66.22311	58.25602	
Finland	Deaths	Male	2017	62.3905	66.38111	57.39178	
Finland	Deaths	Male	2018	61.75504	66.45993	57.40384	
Finland	Deaths	Male	2019	61.97529	66.50335	57.40843	
Finland	Deaths	Male	2020	60.34275	64.56178	55.77098	
Finland	Deaths	Male	2021	63.13322	67.86823	57.93552	
Finland	Deaths	Male	2022	62.38404	67.88018	57.51539	
Finland	Deaths	Male	2023	66.12348	72.35271	60.41159	
Finland	Deaths	Female	2010	27.7223	29.91065	24.78816	
Finland	Deaths	Female	2011	28.76545	30.9784	25.51187	
Finland	Deaths	Female	2012	30.47823	32.82594	26.7174	
Finland	Deaths	Female	2013	31.1792	33.67376	27.50013	
Finland	Deaths	Female	2014	32.54367	35.13974	28.62268	
Finland	Deaths	Female	2015	32.75543	35.45807	28.90784	
Finland	Deaths	Female	2016	33.00139	35.86126	29.12517	
Finland	Deaths	Female	2017	33.22125	36.33533	29.55585	
Finland	Deaths	Female	2018	34.14511	37.60878	30.10382	
Finland	Deaths	Female	2019	35.20454	38.92591	30.88969	
Finland	Deaths	Female	2020	36.39546	40.63848	32.05839	
Finland	Deaths	Female	2021	37.09115	41.15962	32.61188	
Finland	Deaths	Female	2022	37.85655	41.89225	33.17481	
Finland	Deaths	Female	2023	40.21512	45.27129	34.07187	
Finland	DALYs	Male	2010	1370.497	1429.425	1305.23	
Finland	DALYs	Male	2011	1343.677	1398.081	1276.13	
Finland	DALYs	Male	2012	1320.636	1389.824	1260.132	
Finland	DALYs	Male	2013	1319.598	1393.001	1248.358	
Finland	DALYs	Male	2014	1284.018	1350	1220.26	

Finland	DALYs	Male	2015	1275.996	1351.374	1216.033	
Finland	DALYs	Male	2016	1282.836	1363.631	1218.162	
Finland	DALYs	Male	2017	1270.916	1361.731	1193.883	
Finland	DALYs	Male	2018	1248.385	1346.24	1175.145	
Finland	DALYs	Male	2019	1234.643	1318.891	1160.909	
Finland	DALYs	Male	2020	1195.107	1282.921	1115.907	
Finland	DALYs	Male	2021	1240.034	1334.133	1155.738	
Finland	DALYs	Male	2022	1210.64	1323.713	1115.561	
Finland	DALYs	Male	2023	1274.996	1393.082	1175.084	
Finland	DALYs	Female	2010	588.8222	627.3034	540.9739	
Finland	DALYs	Female	2011	606.6762	646.9774	555.0547	
Finland	DALYs	Female	2012	633.716	677.7261	580.3352	
Finland	DALYs	Female	2013	643.5812	691.4148	583.9703	
Finland	DALYs	Female	2014	662.1388	708.9761	601.9072	
Finland	DALYs	Female	2015	662.7688	707.2904	609.0782	
Finland	DALYs	Female	2016	665.2402	712.3347	605.7316	
Finland	DALYs	Female	2017	659.1461	711.0409	601.5128	
Finland	DALYs	Female	2018	666.7123	721.8757	600.1253	
Finland	DALYs	Female	2019	675.1876	731.3872	603.8763	
Finland	DALYs	Female	2020	697.8465	761.5116	625.2617	
Finland	DALYs	Female	2021	709.896	771.0435	640.4851	
Finland	DALYs	Female	2022	726.779	794.2316	653.956	
Finland	DALYs	Female	2023	763.9574	845.0029	674.2252	
France (metrop	Incidence	Male	2010	114.8028	124.2694	106.2159	
France (metrop	Incidence	Male	2011	116.1901	125.8573	107.7687	
France (metrop	Incidence	Male	2012	118.2632	128.5349	109.4026	
France (metrop	Incidence	Male	2013	119.7474	131.2801	111.1458	
France (metrop	Incidence	Male	2014	118.7956	130.5493	109.4385	
France (metrop	Incidence	Male	2015	120.2042	131.3871	110.3178	
France (metrop	Incidence	Male	2016	118.8414	130.4744	108.6359	
France (metrop	Incidence	Male	2017	116.8287	128.0953	106.0259	
France (metrop	Incidence	Male	2018	115.5701	125.9906	104.6524	
France (metrop	Incidence	Male	2019	112.3255	122.7171	101.2326	
France (metrop	Incidence	Male	2020	110.3055	122.0485	98.3241	
France (metrop	Incidence	Male	2021	106.4234	118.7494	94.979	
France (metrop	Incidence	Male	2022	114.8203	127.7755	102.8593	

France (metrop	Incidence	Male	2023	117.0741	131.7335	104.8773	
France (metrop	Incidence	Female	2010	35.41742	39.71053	31.61853	
France (metrop	Incidence	Female	2011	36.87781	41.17721	32.76137	
France (metrop	Incidence	Female	2012	39.61437	44.21602	35.17579	
France (metrop	Incidence	Female	2013	40.59801	45.36359	36.08416	
France (metrop	Incidence	Female	2014	42.67623	47.6677	37.80383	
France (metrop	Incidence	Female	2015	44.38225	49.91233	38.96715	
France (metrop	Incidence	Female	2016	45.29262	50.82182	39.85537	
France (metrop	Incidence	Female	2017	46.25055	52.04931	40.61959	
France (metrop	Incidence	Female	2018	46.54728	52.31984	40.58825	
France (metrop	Incidence	Female	2019	47.75479	54.26334	41.6973	
France (metrop	Incidence	Female	2020	47.33913	53.32633	41.57944	
France (metrop	Incidence	Female	2021	45.3921	50.93658	39.44771	
France (metrop	Incidence	Female	2022	49.68141	56.18925	42.98953	
France (metrop	Incidence	Female	2023	50.41759	57.1937	43.63068	
France (metrop	Deaths	Male	2010	90.9006	96.72544	85.32934	
France (metrop	Deaths	Male	2011	90.86131	96.77315	85.26793	
France (metrop	Deaths	Male	2012	91.56241	97.34974	85.88436	
France (metrop	Deaths	Male	2013	91.66449	97.37701	86.33171	
France (metrop	Deaths	Male	2014	89.98892	96.27159	84.88355	
France (metrop	Deaths	Male	2015	90.49408	96.4065	84.82593	
France (metrop	Deaths	Male	2016	88.94667	95.6934	83.00777	
France (metrop	Deaths	Male	2017	87.35589	94.35656	81.44536	
France (metrop	Deaths	Male	2018	86.3447	93.63545	80.49294	
France (metrop	Deaths	Male	2019	84.04762	90.41122	78.11394	
France (metrop	Deaths	Male	2020	82.45235	89.51179	76.31456	
France (metrop	Deaths	Male	2021	79.63843	86.78299	73.57627	
France (metrop	Deaths	Male	2022	86.38327	95.31298	79.37635	
France (metrop	Deaths	Male	2023	88.22408	97.39908	80.67088	
France (metrop	Deaths	Female	2010	28.61715	30.87592	25.39042	
France (metrop	Deaths	Female	2011	29.44192	31.98081	26.07866	
France (metrop	Deaths	Female	2012	31.33166	34.07799	27.81798	
France (metrop	Deaths	Female	2013	31.69041	34.49339	28.06214	
France (metrop	Deaths	Female	2014	32.85995	35.92923	29.22619	
France (metrop	Deaths	Female	2015	33.97836	36.88319	30.4032	
France (metrop	Deaths	Female	2016	34.33398	37.30427	30.63679	

France (metrop	Deaths	Female	2017	34.83347	37.90691	31.08474	
France (metrop	Deaths	Female	2018	34.886	37.82403	30.98332	
France (metrop	Deaths	Female	2019	35.70934	38.97127	31.40227	
France (metrop	Deaths	Female	2020	35.33271	38.7048	31.45512	
France (metrop	Deaths	Female	2021	33.84664	37.11269	29.99376	
France (metrop	Deaths	Female	2022	37.14528	40.70649	32.94486	
France (metrop	Deaths	Female	2023	37.50235	40.89587	33.03537	
France (metrop	DALYs	Male	2010	2203.871	2344.486	2071.609	
France (metrop	DALYs	Male	2011	2193.02	2327.144	2059.287	
France (metrop	DALYs	Male	2012	2182.493	2324.49	2047.505	
France (metrop	DALYs	Male	2013	2174.282	2308.545	2054.85	
France (metrop	DALYs	Male	2014	2127.96	2268.129	2014.532	
France (metrop	DALYs	Male	2015	2120.466	2267.052	2001.013	
France (metrop	DALYs	Male	2016	2072.733	2226.59	1938.565	
France (metrop	DALYs	Male	2017	2014.951	2166.959	1887.538	
France (metrop	DALYs	Male	2018	1975.899	2132.857	1848.514	
France (metrop	DALYs	Male	2019	1902.209	2040.662	1776.066	
France (metrop	DALYs	Male	2020	1853.891	2012.349	1729.301	
France (metrop	DALYs	Male	2021	1782.835	1946.553	1663.196	
France (metrop	DALYs	Male	2022	1925.938	2127.61	1781.295	
France (metrop	DALYs	Male	2023	1968.42	2186.471	1804.907	
France (metrop	DALYs	Female	2010	669.6898	716.1359	612.7886	
France (metrop	DALYs	Female	2011	688.6619	742.444	628.7657	
France (metrop	DALYs	Female	2012	725.6752	782.7077	662.3743	
France (metrop	DALYs	Female	2013	731.8545	786.3595	670.1417	
France (metrop	DALYs	Female	2014	757.8231	814.7237	697.4015	
France (metrop	DALYs	Female	2015	772.4401	827.9956	710.7817	
France (metrop	DALYs	Female	2016	778.2926	838.1776	714.9702	
France (metrop	DALYs	Female	2017	785.8146	845.6065	720.3826	
France (metrop	DALYs	Female	2018	782.0348	841.5554	716.523	
France (metrop	DALYs	Female	2019	793.6609	861.3723	725.7846	
France (metrop	DALYs	Female	2020	777.0419	847.9521	710.8173	
France (metrop	DALYs	Female	2021	740.4573	812.3984	673.8524	
France (metrop	DALYs	Female	2022	809.2025	891.2519	731.1357	
France (metrop	DALYs	Female	2023	823.183	903.1381	739.2532	
French Guyana	Incidence	Male	2010	114.8028	124.2694	106.2159	

French Guyana	Incidence	Male	2011	116.1901	125.8573	107.7687	
French Guyana	Incidence	Male	2012	118.2632	128.5349	109.4026	
French Guyana	Incidence	Male	2013	119.7474	131.2801	111.1458	
French Guyana	Incidence	Male	2014	118.7956	130.5493	109.4385	
French Guyana	Incidence	Male	2015	120.2042	131.3871	110.3178	
French Guyana	Incidence	Male	2016	118.8414	130.4744	108.6359	
French Guyana	Incidence	Male	2017	116.8287	128.0953	106.0259	
French Guyana	Incidence	Male	2018	115.5701	125.9906	104.6524	
French Guyana	Incidence	Male	2019	112.3255	122.7171	101.2326	
French Guyana	Incidence	Male	2020	110.3055	122.0485	98.3241	
French Guyana	Incidence	Male	2021	106.4234	118.7494	94.979	
French Guyana	Incidence	Male	2022	114.8203	127.7755	102.8593	
French Guyana	Incidence	Male	2023	117.0741	131.7335	104.8773	
French Guyana	Incidence	Female	2010	35.41742	39.71053	31.61853	
French Guyana	Incidence	Female	2011	36.87781	41.17721	32.76137	
French Guyana	Incidence	Female	2012	39.61437	44.21602	35.17579	
French Guyana	Incidence	Female	2013	40.59801	45.36359	36.08416	
French Guyana	Incidence	Female	2014	42.67623	47.6677	37.80383	
French Guyana	Incidence	Female	2015	44.38225	49.91233	38.96715	
French Guyana	Incidence	Female	2016	45.29262	50.82182	39.85537	
French Guyana	Incidence	Female	2017	46.25055	52.04931	40.61959	
French Guyana	Incidence	Female	2018	46.54728	52.31984	40.58825	
French Guyana	Incidence	Female	2019	47.75479	54.26334	41.6973	
French Guyana	Incidence	Female	2020	47.33913	53.32633	41.57944	
French Guyana	Incidence	Female	2021	45.3921	50.93658	39.44771	
French Guyana	Incidence	Female	2022	49.68141	56.18925	42.98953	
French Guyana	Incidence	Female	2023	50.41759	57.1937	43.63068	
French Guyana	Deaths	Male	2010	90.9006	96.72544	85.32934	
French Guyana	Deaths	Male	2011	90.86131	96.77315	85.26793	
French Guyana	Deaths	Male	2012	91.56241	97.34974	85.88436	
French Guyana	Deaths	Male	2013	91.66449	97.37701	86.33171	
French Guyana	Deaths	Male	2014	89.98892	96.27159	84.88355	
French Guyana	Deaths	Male	2015	90.49408	96.4065	84.82593	
French Guyana	Deaths	Male	2016	88.94667	95.6934	83.00777	
French Guyana	Deaths	Male	2017	87.35589	94.35656	81.44536	
French Guyana	Deaths	Male	2018	86.3447	93.63545	80.49294	

French Guyana	Deaths	Male	2019	84.04762	90.41122	78.11394	
French Guyana	Deaths	Male	2020	82.45235	89.51179	76.31456	
French Guyana	Deaths	Male	2021	79.63843	86.78299	73.57627	
French Guyana	Deaths	Male	2022	86.38327	95.31298	79.37635	
French Guyana	Deaths	Male	2023	88.22408	97.39908	80.67088	
French Guyana	Deaths	Female	2010	28.61715	30.87592	25.39042	
French Guyana	Deaths	Female	2011	29.44192	31.98081	26.07866	
French Guyana	Deaths	Female	2012	31.33166	34.07799	27.81798	
French Guyana	Deaths	Female	2013	31.69041	34.49339	28.06214	
French Guyana	Deaths	Female	2014	32.85995	35.92923	29.22619	
French Guyana	Deaths	Female	2015	33.97836	36.88319	30.4032	
French Guyana	Deaths	Female	2016	34.33398	37.30427	30.63679	
French Guyana	Deaths	Female	2017	34.83347	37.90691	31.08474	
French Guyana	Deaths	Female	2018	34.886	37.82403	30.98332	
French Guyana	Deaths	Female	2019	35.70934	38.97127	31.40227	
French Guyana	Deaths	Female	2020	35.33271	38.7048	31.45512	
French Guyana	Deaths	Female	2021	33.84664	37.11269	29.99376	
French Guyana	Deaths	Female	2022	37.14528	40.70649	32.94486	
French Guyana	Deaths	Female	2023	37.50235	40.89587	33.03537	
French Guyana	DALYs	Male	2010	2203.871	2344.486	2071.609	
French Guyana	DALYs	Male	2011	2193.02	2327.144	2059.287	
French Guyana	DALYs	Male	2012	2182.493	2324.49	2047.505	
French Guyana	DALYs	Male	2013	2174.282	2308.545	2054.85	
French Guyana	DALYs	Male	2014	2127.96	2268.129	2014.532	
French Guyana	DALYs	Male	2015	2120.466	2267.052	2001.013	
French Guyana	DALYs	Male	2016	2072.733	2226.59	1938.565	
French Guyana	DALYs	Male	2017	2014.951	2166.959	1887.538	
French Guyana	DALYs	Male	2018	1975.899	2132.857	1848.514	
French Guyana	DALYs	Male	2019	1902.209	2040.662	1776.066	
French Guyana	DALYs	Male	2020	1853.891	2012.349	1729.301	
French Guyana	DALYs	Male	2021	1782.835	1946.553	1663.196	
French Guyana	DALYs	Male	2022	1925.938	2127.61	1781.295	
French Guyana	DALYs	Male	2023	1968.42	2186.471	1804.907	
French Guyana	DALYs	Female	2010	669.6898	716.1359	612.7886	
French Guyana	DALYs	Female	2011	688.6619	742.444	628.7657	
French Guyana	DALYs	Female	2012	725.6752	782.7077	662.3743	

French Guyana	DALYs	Female	2013	731.8545	786.3595	670.1417	
French Guyana	DALYs	Female	2014	757.8231	814.7237	697.4015	
French Guyana	DALYs	Female	2015	772.4401	827.9956	710.7817	
French Guyana	DALYs	Female	2016	778.2926	838.1776	714.9702	
French Guyana	DALYs	Female	2017	785.8146	845.6065	720.3826	
French Guyana	DALYs	Female	2018	782.0348	841.5554	716.523	
French Guyana	DALYs	Female	2019	793.6609	861.3723	725.7846	
French Guyana	DALYs	Female	2020	777.0419	847.9521	710.8173	
French Guyana	DALYs	Female	2021	740.4573	812.3984	673.8524	
French Guyana	DALYs	Female	2022	809.2025	891.2519	731.1357	
French Guyana	DALYs	Female	2023	823.183	903.1381	739.2532	
French Polynes	Incidence	Male	2010	114.8028	124.2694	106.2159	
French Polynes	Incidence	Male	2011	116.1901	125.8573	107.7687	
French Polynes	Incidence	Male	2012	118.2632	128.5349	109.4026	
French Polynes	Incidence	Male	2013	119.7474	131.2801	111.1458	
French Polynes	Incidence	Male	2014	118.7956	130.5493	109.4385	
French Polynes	Incidence	Male	2015	120.2042	131.3871	110.3178	
French Polynes	Incidence	Male	2016	118.8414	130.4744	108.6359	
French Polynes	Incidence	Male	2017	116.8287	128.0953	106.0259	
French Polynes	Incidence	Male	2018	115.5701	125.9906	104.6524	
French Polynes	Incidence	Male	2019	112.3255	122.7171	101.2326	
French Polynes	Incidence	Male	2020	110.3055	122.0485	98.3241	
French Polynes	Incidence	Male	2021	106.4234	118.7494	94.979	
French Polynes	Incidence	Male	2022	114.8203	127.7755	102.8593	
French Polynes	Incidence	Male	2023	117.0741	131.7335	104.8773	
French Polynes	Incidence	Female	2010	35.41742	39.71053	31.61853	
French Polynes	Incidence	Female	2011	36.87781	41.17721	32.76137	
French Polynes	Incidence	Female	2012	39.61437	44.21602	35.17579	
French Polynes	Incidence	Female	2013	40.59801	45.36359	36.08416	
French Polynes	Incidence	Female	2014	42.67623	47.6677	37.80383	
French Polynes	Incidence	Female	2015	44.38225	49.91233	38.96715	
French Polynes	Incidence	Female	2016	45.29262	50.82182	39.85537	
French Polynes	Incidence	Female	2017	46.25055	52.04931	40.61959	
French Polynes	Incidence	Female	2018	46.54728	52.31984	40.58825	
French Polynes	Incidence	Female	2019	47.75479	54.26334	41.6973	
French Polynes	Incidence	Female	2020	47.33913	53.32633	41.57944	

French Polynes	Incidence	Female	2021	45.3921	50.93658	39.44771	
French Polynes	Incidence	Female	2022	49.68141	56.18925	42.98953	
French Polynes	Incidence	Female	2023	50.41759	57.1937	43.63068	
French Polynes	Deaths	Male	2010	90.9006	96.72544	85.32934	
French Polynes	Deaths	Male	2011	90.86131	96.77315	85.26793	
French Polynes	Deaths	Male	2012	91.56241	97.34974	85.88436	
French Polynes	Deaths	Male	2013	91.66449	97.37701	86.33171	
French Polynes	Deaths	Male	2014	89.98892	96.27159	84.88355	
French Polynes	Deaths	Male	2015	90.49408	96.4065	84.82593	
French Polynes	Deaths	Male	2016	88.94667	95.6934	83.00777	
French Polynes	Deaths	Male	2017	87.35589	94.35656	81.44536	
French Polynes	Deaths	Male	2018	86.3447	93.63545	80.49294	
French Polynes	Deaths	Male	2019	84.04762	90.41122	78.11394	
French Polynes	Deaths	Male	2020	82.45235	89.51179	76.31456	
French Polynes	Deaths	Male	2021	79.63843	86.78299	73.57627	
French Polynes	Deaths	Male	2022	86.38327	95.31298	79.37635	
French Polynes	Deaths	Male	2023	88.22408	97.39908	80.67088	
French Polynes	Deaths	Female	2010	28.61715	30.87592	25.39042	
French Polynes	Deaths	Female	2011	29.44192	31.98081	26.07866	
French Polynes	Deaths	Female	2012	31.33166	34.07799	27.81798	
French Polynes	Deaths	Female	2013	31.69041	34.49339	28.06214	
French Polynes	Deaths	Female	2014	32.85995	35.92923	29.22619	
French Polynes	Deaths	Female	2015	33.97836	36.88319	30.4032	
French Polynes	Deaths	Female	2016	34.33398	37.30427	30.63679	
French Polynes	Deaths	Female	2017	34.83347	37.90691	31.08474	
French Polynes	Deaths	Female	2018	34.886	37.82403	30.98332	
French Polynes	Deaths	Female	2019	35.70934	38.97127	31.40227	
French Polynes	Deaths	Female	2020	35.33271	38.7048	31.45512	
French Polynes	Deaths	Female	2021	33.84664	37.11269	29.99376	
French Polynes	Deaths	Female	2022	37.14528	40.70649	32.94486	
French Polynes	Deaths	Female	2023	37.50235	40.89587	33.03537	
French Polynes	DALYs	Male	2010	2203.871	2344.486	2071.609	
French Polynes	DALYs	Male	2011	2193.02	2327.144	2059.287	
French Polynes	DALYs	Male	2012	2182.493	2324.49	2047.505	
French Polynes	DALYs	Male	2013	2174.282	2308.545	2054.85	
French Polynes	DALYs	Male	2014	2127.96	2268.129	2014.532	

French Polynes	DALYs	Male	2015	2120.466	2267.052	2001.013	
French Polynes	DALYs	Male	2016	2072.733	2226.59	1938.565	
French Polynes	DALYs	Male	2017	2014.951	2166.959	1887.538	
French Polynes	DALYs	Male	2018	1975.899	2132.857	1848.514	
French Polynes	DALYs	Male	2019	1902.209	2040.662	1776.066	
French Polynes	DALYs	Male	2020	1853.891	2012.349	1729.301	
French Polynes	DALYs	Male	2021	1782.835	1946.553	1663.196	
French Polynes	DALYs	Male	2022	1925.938	2127.61	1781.295	
French Polynes	DALYs	Male	2023	1968.42	2186.471	1804.907	
French Polynes	DALYs	Female	2010	669.6898	716.1359	612.7886	
French Polynes	DALYs	Female	2011	688.6619	742.444	628.7657	
French Polynes	DALYs	Female	2012	725.6752	782.7077	662.3743	
French Polynes	DALYs	Female	2013	731.8545	786.3595	670.1417	
French Polynes	DALYs	Female	2014	757.8231	814.7237	697.4015	
French Polynes	DALYs	Female	2015	772.4401	827.9956	710.7817	
French Polynes	DALYs	Female	2016	778.2926	838.1776	714.9702	
French Polynes	DALYs	Female	2017	785.8146	845.6065	720.3826	
French Polynes	DALYs	Female	2018	782.0348	841.5554	716.523	
French Polynes	DALYs	Female	2019	793.6609	861.3723	725.7846	
French Polynes	DALYs	Female	2020	777.0419	847.9521	710.8173	
French Polynes	DALYs	Female	2021	740.4573	812.3984	673.8524	
French Polynes	DALYs	Female	2022	809.2025	891.2519	731.1357	
French Polynes	DALYs	Female	2023	823.183	903.1381	739.2532	
France, Guadel	Incidence	Male	2010	114.8028	124.2694	106.2159	
France, Guadel	Incidence	Male	2011	116.1901	125.8573	107.7687	
France, Guadel	Incidence	Male	2012	118.2632	128.5349	109.4026	
France, Guadel	Incidence	Male	2013	119.7474	131.2801	111.1458	
France, Guadel	Incidence	Male	2014	118.7956	130.5493	109.4385	
France, Guadel	Incidence	Male	2015	120.2042	131.3871	110.3178	
France, Guadel	Incidence	Male	2016	118.8414	130.4744	108.6359	
France, Guadel	Incidence	Male	2017	116.8287	128.0953	106.0259	
France, Guadel	Incidence	Male	2018	115.5701	125.9906	104.6524	
France, Guadel	Incidence	Male	2019	112.3255	122.7171	101.2326	
France, Guadel	Incidence	Male	2020	110.3055	122.0485	98.3241	
France, Guadel	Incidence	Male	2021	106.4234	118.7494	94.979	
France, Guadel	Incidence	Male	2022	114.8203	127.7755	102.8593	

France, Guadel	Incidence	Male	2023	117.0741	131.7335	104.8773	
France, Guadel	Incidence	Female	2010	35.41742	39.71053	31.61853	
France, Guadel	Incidence	Female	2011	36.87781	41.17721	32.76137	
France, Guadel	Incidence	Female	2012	39.61437	44.21602	35.17579	
France, Guadel	Incidence	Female	2013	40.59801	45.36359	36.08416	
France, Guadel	Incidence	Female	2014	42.67623	47.6677	37.80383	
France, Guadel	Incidence	Female	2015	44.38225	49.91233	38.96715	
France, Guadel	Incidence	Female	2016	45.29262	50.82182	39.85537	
France, Guadel	Incidence	Female	2017	46.25055	52.04931	40.61959	
France, Guadel	Incidence	Female	2018	46.54728	52.31984	40.58825	
France, Guadel	Incidence	Female	2019	47.75479	54.26334	41.6973	
France, Guadel	Incidence	Female	2020	47.33913	53.32633	41.57944	
France, Guadel	Incidence	Female	2021	45.3921	50.93658	39.44771	
France, Guadel	Incidence	Female	2022	49.68141	56.18925	42.98953	
France, Guadel	Incidence	Female	2023	50.41759	57.1937	43.63068	
France, Guadel	Deaths	Male	2010	90.9006	96.72544	85.32934	
France, Guadel	Deaths	Male	2011	90.86131	96.77315	85.26793	
France, Guadel	Deaths	Male	2012	91.56241	97.34974	85.88436	
France, Guadel	Deaths	Male	2013	91.66449	97.37701	86.33171	
France, Guadel	Deaths	Male	2014	89.98892	96.27159	84.88355	
France, Guadel	Deaths	Male	2015	90.49408	96.4065	84.82593	
France, Guadel	Deaths	Male	2016	88.94667	95.6934	83.00777	
France, Guadel	Deaths	Male	2017	87.35589	94.35656	81.44536	
France, Guadel	Deaths	Male	2018	86.3447	93.63545	80.49294	
France, Guadel	Deaths	Male	2019	84.04762	90.41122	78.11394	
France, Guadel	Deaths	Male	2020	82.45235	89.51179	76.31456	
France, Guadel	Deaths	Male	2021	79.63843	86.78299	73.57627	
France, Guadel	Deaths	Male	2022	86.38327	95.31298	79.37635	
France, Guadel	Deaths	Male	2023	88.22408	97.39908	80.67088	
France, Guadel	Deaths	Female	2010	28.61715	30.87592	25.39042	
France, Guadel	Deaths	Female	2011	29.44192	31.98081	26.07866	
France, Guadel	Deaths	Female	2012	31.33166	34.07799	27.81798	
France, Guadel	Deaths	Female	2013	31.69041	34.49339	28.06214	
France, Guadel	Deaths	Female	2014	32.85995	35.92923	29.22619	
France, Guadel	Deaths	Female	2015	33.97836	36.88319	30.4032	
France, Guadel	Deaths	Female	2016	34.33398	37.30427	30.63679	

France, Guadel	Deaths	Female	2017	34.83347	37.90691	31.08474	
France, Guadel	Deaths	Female	2018	34.886	37.82403	30.98332	
France, Guadel	Deaths	Female	2019	35.70934	38.97127	31.40227	
France, Guadel	Deaths	Female	2020	35.33271	38.7048	31.45512	
France, Guadel	Deaths	Female	2021	33.84664	37.11269	29.99376	
France, Guadel	Deaths	Female	2022	37.14528	40.70649	32.94486	
France, Guadel	Deaths	Female	2023	37.50235	40.89587	33.03537	
France, Guadel	DALYs	Male	2010	2203.871	2344.486	2071.609	
France, Guadel	DALYs	Male	2011	2193.02	2327.144	2059.287	
France, Guadel	DALYs	Male	2012	2182.493	2324.49	2047.505	
France, Guadel	DALYs	Male	2013	2174.282	2308.545	2054.85	
France, Guadel	DALYs	Male	2014	2127.96	2268.129	2014.532	
France, Guadel	DALYs	Male	2015	2120.466	2267.052	2001.013	
France, Guadel	DALYs	Male	2016	2072.733	2226.59	1938.565	
France, Guadel	DALYs	Male	2017	2014.951	2166.959	1887.538	
France, Guadel	DALYs	Male	2018	1975.899	2132.857	1848.514	
France, Guadel	DALYs	Male	2019	1902.209	2040.662	1776.066	
France, Guadel	DALYs	Male	2020	1853.891	2012.349	1729.301	
France, Guadel	DALYs	Male	2021	1782.835	1946.553	1663.196	
France, Guadel	DALYs	Male	2022	1925.938	2127.61	1781.295	
France, Guadel	DALYs	Male	2023	1968.42	2186.471	1804.907	
France, Guadel	DALYs	Female	2010	669.6898	716.1359	612.7886	
France, Guadel	DALYs	Female	2011	688.6619	742.444	628.7657	
France, Guadel	DALYs	Female	2012	725.6752	782.7077	662.3743	
France, Guadel	DALYs	Female	2013	731.8545	786.3595	670.1417	
France, Guadel	DALYs	Female	2014	757.8231	814.7237	697.4015	
France, Guadel	DALYs	Female	2015	772.4401	827.9956	710.7817	
France, Guadel	DALYs	Female	2016	778.2926	838.1776	714.9702	
France, Guadel	DALYs	Female	2017	785.8146	845.6065	720.3826	
France, Guadel	DALYs	Female	2018	782.0348	841.5554	716.523	
France, Guadel	DALYs	Female	2019	793.6609	861.3723	725.7846	
France, Guadel	DALYs	Female	2020	777.0419	847.9521	710.8173	
France, Guadel	DALYs	Female	2021	740.4573	812.3984	673.8524	
France, Guadel	DALYs	Female	2022	809.2025	891.2519	731.1357	
France, Guadel	DALYs	Female	2023	823.183	903.1381	739.2532	
France, La Réur	Incidence	Male	2010	114.8028	124.2694	106.2159	

France, La Réur	Incidence	Male	2011	116.1901	125.8573	107.7687	
France, La Réur	Incidence	Male	2012	118.2632	128.5349	109.4026	
France, La Réur	Incidence	Male	2013	119.7474	131.2801	111.1458	
France, La Réur	Incidence	Male	2014	118.7956	130.5493	109.4385	
France, La Réur	Incidence	Male	2015	120.2042	131.3871	110.3178	
France, La Réur	Incidence	Male	2016	118.8414	130.4744	108.6359	
France, La Réur	Incidence	Male	2017	116.8287	128.0953	106.0259	
France, La Réur	Incidence	Male	2018	115.5701	125.9906	104.6524	
France, La Réur	Incidence	Male	2019	112.3255	122.7171	101.2326	
France, La Réur	Incidence	Male	2020	110.3055	122.0485	98.3241	
France, La Réur	Incidence	Male	2021	106.4234	118.7494	94.979	
France, La Réur	Incidence	Male	2022	114.8203	127.7755	102.8593	
France, La Réur	Incidence	Male	2023	117.0741	131.7335	104.8773	
France, La Réur	Incidence	Female	2010	35.41742	39.71053	31.61853	
France, La Réur	Incidence	Female	2011	36.87781	41.17721	32.76137	
France, La Réur	Incidence	Female	2012	39.61437	44.21602	35.17579	
France, La Réur	Incidence	Female	2013	40.59801	45.36359	36.08416	
France, La Réur	Incidence	Female	2014	42.67623	47.6677	37.80383	
France, La Réur	Incidence	Female	2015	44.38225	49.91233	38.96715	
France, La Réur	Incidence	Female	2016	45.29262	50.82182	39.85537	
France, La Réur	Incidence	Female	2017	46.25055	52.04931	40.61959	
France, La Réur	Incidence	Female	2018	46.54728	52.31984	40.58825	
France, La Réur	Incidence	Female	2019	47.75479	54.26334	41.6973	
France, La Réur	Incidence	Female	2020	47.33913	53.32633	41.57944	
France, La Réur	Incidence	Female	2021	45.3921	50.93658	39.44771	
France, La Réur	Incidence	Female	2022	49.68141	56.18925	42.98953	
France, La Réur	Incidence	Female	2023	50.41759	57.1937	43.63068	
France, La Réur	Deaths	Male	2010	90.9006	96.72544	85.32934	
France, La Réur	Deaths	Male	2011	90.86131	96.77315	85.26793	
France, La Réur	Deaths	Male	2012	91.56241	97.34974	85.88436	
France, La Réur	Deaths	Male	2013	91.66449	97.37701	86.33171	
France, La Réur	Deaths	Male	2014	89.98892	96.27159	84.88355	
France, La Réur	Deaths	Male	2015	90.49408	96.4065	84.82593	
France, La Réur	Deaths	Male	2016	88.94667	95.6934	83.00777	
France, La Réur	Deaths	Male	2017	87.35589	94.35656	81.44536	
France, La Réur	Deaths	Male	2018	86.3447	93.63545	80.49294	

France, La Réur	Deaths	Male	2019	84.04762	90.41122	78.11394	
France, La Réur	Deaths	Male	2020	82.45235	89.51179	76.31456	
France, La Réur	Deaths	Male	2021	79.63843	86.78299	73.57627	
France, La Réur	Deaths	Male	2022	86.38327	95.31298	79.37635	
France, La Réur	Deaths	Male	2023	88.22408	97.39908	80.67088	
France, La Réur	Deaths	Female	2010	28.61715	30.87592	25.39042	
France, La Réur	Deaths	Female	2011	29.44192	31.98081	26.07866	
France, La Réur	Deaths	Female	2012	31.33166	34.07799	27.81798	
France, La Réur	Deaths	Female	2013	31.69041	34.49339	28.06214	
France, La Réur	Deaths	Female	2014	32.85995	35.92923	29.22619	
France, La Réur	Deaths	Female	2015	33.97836	36.88319	30.4032	
France, La Réur	Deaths	Female	2016	34.33398	37.30427	30.63679	
France, La Réur	Deaths	Female	2017	34.83347	37.90691	31.08474	
France, La Réur	Deaths	Female	2018	34.886	37.82403	30.98332	
France, La Réur	Deaths	Female	2019	35.70934	38.97127	31.40227	
France, La Réur	Deaths	Female	2020	35.33271	38.7048	31.45512	
France, La Réur	Deaths	Female	2021	33.84664	37.11269	29.99376	
France, La Réur	Deaths	Female	2022	37.14528	40.70649	32.94486	
France, La Réur	Deaths	Female	2023	37.50235	40.89587	33.03537	
France, La Réur	DALYs	Male	2010	2203.871	2344.486	2071.609	
France, La Réur	DALYs	Male	2011	2193.02	2327.144	2059.287	
France, La Réur	DALYs	Male	2012	2182.493	2324.49	2047.505	
France, La Réur	DALYs	Male	2013	2174.282	2308.545	2054.85	
France, La Réur	DALYs	Male	2014	2127.96	2268.129	2014.532	
France, La Réur	DALYs	Male	2015	2120.466	2267.052	2001.013	
France, La Réur	DALYs	Male	2016	2072.733	2226.59	1938.565	
France, La Réur	DALYs	Male	2017	2014.951	2166.959	1887.538	
France, La Réur	DALYs	Male	2018	1975.899	2132.857	1848.514	
France, La Réur	DALYs	Male	2019	1902.209	2040.662	1776.066	
France, La Réur	DALYs	Male	2020	1853.891	2012.349	1729.301	
France, La Réur	DALYs	Male	2021	1782.835	1946.553	1663.196	
France, La Réur	DALYs	Male	2022	1925.938	2127.61	1781.295	
France, La Réur	DALYs	Male	2023	1968.42	2186.471	1804.907	
France, La Réur	DALYs	Female	2010	669.6898	716.1359	612.7886	
France, La Réur	DALYs	Female	2011	688.6619	742.444	628.7657	
France, La Réur	DALYs	Female	2012	725.6752	782.7077	662.3743	

France, La Réur	DALYs	Female	2013	731.8545	786.3595	670.1417	
France, La Réur	DALYs	Female	2014	757.8231	814.7237	697.4015	
France, La Réur	DALYs	Female	2015	772.4401	827.9956	710.7817	
France, La Réur	DALYs	Female	2016	778.2926	838.1776	714.9702	
France, La Réur	DALYs	Female	2017	785.8146	845.6065	720.3826	
France, La Réur	DALYs	Female	2018	782.0348	841.5554	716.523	
France, La Réur	DALYs	Female	2019	793.6609	861.3723	725.7846	
France, La Réur	DALYs	Female	2020	777.0419	847.9521	710.8173	
France, La Réur	DALYs	Female	2021	740.4573	812.3984	673.8524	
France, La Réur	DALYs	Female	2022	809.2025	891.2519	731.1357	
France, La Réur	DALYs	Female	2023	823.183	903.1381	739.2532	
France, Martini	Incidence	Male	2010	114.8028	124.2694	106.2159	
France, Martini	Incidence	Male	2011	116.1901	125.8573	107.7687	
France, Martini	Incidence	Male	2012	118.2632	128.5349	109.4026	
France, Martini	Incidence	Male	2013	119.7474	131.2801	111.1458	
France, Martini	Incidence	Male	2014	118.7956	130.5493	109.4385	
France, Martini	Incidence	Male	2015	120.2042	131.3871	110.3178	
France, Martini	Incidence	Male	2016	118.8414	130.4744	108.6359	
France, Martini	Incidence	Male	2017	116.8287	128.0953	106.0259	
France, Martini	Incidence	Male	2018	115.5701	125.9906	104.6524	
France, Martini	Incidence	Male	2019	112.3255	122.7171	101.2326	
France, Martini	Incidence	Male	2020	110.3055	122.0485	98.3241	
France, Martini	Incidence	Male	2021	106.4234	118.7494	94.979	
France, Martini	Incidence	Male	2022	114.8203	127.7755	102.8593	
France, Martini	Incidence	Male	2023	117.0741	131.7335	104.8773	
France, Martini	Incidence	Female	2010	35.41742	39.71053	31.61853	
France, Martini	Incidence	Female	2011	36.87781	41.17721	32.76137	
France, Martini	Incidence	Female	2012	39.61437	44.21602	35.17579	
France, Martini	Incidence	Female	2013	40.59801	45.36359	36.08416	
France, Martini	Incidence	Female	2014	42.67623	47.6677	37.80383	
France, Martini	Incidence	Female	2015	44.38225	49.91233	38.96715	
France, Martini	Incidence	Female	2016	45.29262	50.82182	39.85537	
France, Martini	Incidence	Female	2017	46.25055	52.04931	40.61959	
France, Martini	Incidence	Female	2018	46.54728	52.31984	40.58825	
France, Martini	Incidence	Female	2019	47.75479	54.26334	41.6973	
France, Martini	Incidence	Female	2020	47.33913	53.32633	41.57944	

France, Martini	Incidence	Female	2021	45.3921	50.93658	39.44771	
France, Martini	Incidence	Female	2022	49.68141	56.18925	42.98953	
France, Martini	Incidence	Female	2023	50.41759	57.1937	43.63068	
France, Martini	Deaths	Male	2010	90.9006	96.72544	85.32934	
France, Martini	Deaths	Male	2011	90.86131	96.77315	85.26793	
France, Martini	Deaths	Male	2012	91.56241	97.34974	85.88436	
France, Martini	Deaths	Male	2013	91.66449	97.37701	86.33171	
France, Martini	Deaths	Male	2014	89.98892	96.27159	84.88355	
France, Martini	Deaths	Male	2015	90.49408	96.4065	84.82593	
France, Martini	Deaths	Male	2016	88.94667	95.6934	83.00777	
France, Martini	Deaths	Male	2017	87.35589	94.35656	81.44536	
France, Martini	Deaths	Male	2018	86.3447	93.63545	80.49294	
France, Martini	Deaths	Male	2019	84.04762	90.41122	78.11394	
France, Martini	Deaths	Male	2020	82.45235	89.51179	76.31456	
France, Martini	Deaths	Male	2021	79.63843	86.78299	73.57627	
France, Martini	Deaths	Male	2022	86.38327	95.31298	79.37635	
France, Martini	Deaths	Male	2023	88.22408	97.39908	80.67088	
France, Martini	Deaths	Female	2010	28.61715	30.87592	25.39042	
France, Martini	Deaths	Female	2011	29.44192	31.98081	26.07866	
France, Martini	Deaths	Female	2012	31.33166	34.07799	27.81798	
France, Martini	Deaths	Female	2013	31.69041	34.49339	28.06214	
France, Martini	Deaths	Female	2014	32.85995	35.92923	29.22619	
France, Martini	Deaths	Female	2015	33.97836	36.88319	30.4032	
France, Martini	Deaths	Female	2016	34.33398	37.30427	30.63679	
France, Martini	Deaths	Female	2017	34.83347	37.90691	31.08474	
France, Martini	Deaths	Female	2018	34.886	37.82403	30.98332	
France, Martini	Deaths	Female	2019	35.70934	38.97127	31.40227	
France, Martini	Deaths	Female	2020	35.33271	38.7048	31.45512	
France, Martini	Deaths	Female	2021	33.84664	37.11269	29.99376	
France, Martini	Deaths	Female	2022	37.14528	40.70649	32.94486	
France, Martini	Deaths	Female	2023	37.50235	40.89587	33.03537	
France, Martini	DALYs	Male	2010	2203.871	2344.486	2071.609	
France, Martini	DALYs	Male	2011	2193.02	2327.144	2059.287	
France, Martini	DALYs	Male	2012	2182.493	2324.49	2047.505	
France, Martini	DALYs	Male	2013	2174.282	2308.545	2054.85	
France, Martini	DALYs	Male	2014	2127.96	2268.129	2014.532	

France, Martini	DALYs	Male	2015	2120.466	2267.052	2001.013	
France, Martini	DALYs	Male	2016	2072.733	2226.59	1938.565	
France, Martini	DALYs	Male	2017	2014.951	2166.959	1887.538	
France, Martini	DALYs	Male	2018	1975.899	2132.857	1848.514	
France, Martini	DALYs	Male	2019	1902.209	2040.662	1776.066	
France, Martini	DALYs	Male	2020	1853.891	2012.349	1729.301	
France, Martini	DALYs	Male	2021	1782.835	1946.553	1663.196	
France, Martini	DALYs	Male	2022	1925.938	2127.61	1781.295	
France, Martini	DALYs	Male	2023	1968.42	2186.471	1804.907	
France, Martini	DALYs	Female	2010	669.6898	716.1359	612.7886	
France, Martini	DALYs	Female	2011	688.6619	742.444	628.7657	
France, Martini	DALYs	Female	2012	725.6752	782.7077	662.3743	
France, Martini	DALYs	Female	2013	731.8545	786.3595	670.1417	
France, Martini	DALYs	Female	2014	757.8231	814.7237	697.4015	
France, Martini	DALYs	Female	2015	772.4401	827.9956	710.7817	
France, Martini	DALYs	Female	2016	778.2926	838.1776	714.9702	
France, Martini	DALYs	Female	2017	785.8146	845.6065	720.3826	
France, Martini	DALYs	Female	2018	782.0348	841.5554	716.523	
France, Martini	DALYs	Female	2019	793.6609	861.3723	725.7846	
France, Martini	DALYs	Female	2020	777.0419	847.9521	710.8173	
France, Martini	DALYs	Female	2021	740.4573	812.3984	673.8524	
France, Martini	DALYs	Female	2022	809.2025	891.2519	731.1357	
France, Martini	DALYs	Female	2023	823.183	903.1381	739.2532	
Gabon	Incidence	Male	2010	5.453784	8.135236	2.724963	
Gabon	Incidence	Male	2011	5.527512	8.161447	2.732607	
Gabon	Incidence	Male	2012	5.641082	8.526593	2.770121	
Gabon	Incidence	Male	2013	5.905378	8.851223	2.979629	
Gabon	Incidence	Male	2014	6.116067	9.05608	3.043691	
Gabon	Incidence	Male	2015	6.446044	9.764758	3.236197	
Gabon	Incidence	Male	2016	6.571428	9.793652	3.329284	
Gabon	Incidence	Male	2017	6.66544	9.803354	3.305389	
Gabon	Incidence	Male	2018	6.821622	10.11232	3.41465	
Gabon	Incidence	Male	2019	7.048443	10.69535	3.524054	
Gabon	Incidence	Male	2020	7.363083	11.34603	3.628579	
Gabon	Incidence	Male	2021	7.547031	11.74841	3.729664	
Gabon	Incidence	Male	2022	8.479059	12.54551	4.15274	

Gabon	Incidence	Male	2023	8.891999	13.4507	4.581189	
Gabon	Incidence	Female	2010	3.260857	4.939	1.686746	
Gabon	Incidence	Female	2011	3.29241	4.840697	1.689141	
Gabon	Incidence	Female	2012	3.474372	5.183504	1.771547	
Gabon	Incidence	Female	2013	3.757129	5.564521	1.944824	
Gabon	Incidence	Female	2014	3.911775	5.808452	2.032328	
Gabon	Incidence	Female	2015	4.038233	6.302261	2.107654	
Gabon	Incidence	Female	2016	4.056597	6.396324	2.083909	
Gabon	Incidence	Female	2017	4.071479	6.389475	2.094753	
Gabon	Incidence	Female	2018	4.097654	6.377461	2.112458	
Gabon	Incidence	Female	2019	4.193713	6.47612	2.177867	
Gabon	Incidence	Female	2020	4.367492	6.933328	2.328105	
Gabon	Incidence	Female	2021	4.571364	7.302502	2.465631	
Gabon	Incidence	Female	2022	5.219201	8.460033	2.846023	
Gabon	Incidence	Female	2023	5.513411	9.003855	2.980384	
Gabon	Deaths	Male	2010	5.424758	8.032919	2.714302	
Gabon	Deaths	Male	2011	5.498774	8.128442	2.738927	
Gabon	Deaths	Male	2012	5.613144	8.495309	2.773189	
Gabon	Deaths	Male	2013	5.874284	8.739567	2.976948	
Gabon	Deaths	Male	2014	6.071517	9.083765	3.025607	
Gabon	Deaths	Male	2015	6.392973	9.693297	3.217764	
Gabon	Deaths	Male	2016	6.519401	9.745682	3.294834	
Gabon	Deaths	Male	2017	6.616427	9.720852	3.270914	
Gabon	Deaths	Male	2018	6.775283	10.01171	3.394339	
Gabon	Deaths	Male	2019	6.997699	10.55493	3.502432	
Gabon	Deaths	Male	2020	7.325187	11.26517	3.616008	
Gabon	Deaths	Male	2021	7.508404	11.67731	3.718253	
Gabon	Deaths	Male	2022	8.432897	12.45427	4.12905	
Gabon	Deaths	Male	2023	8.838433	13.41688	4.5184	
Gabon	Deaths	Female	2010	3.34921	5.086805	1.72095	
Gabon	Deaths	Female	2011	3.381307	4.970044	1.720998	
Gabon	Deaths	Female	2012	3.550934	5.326494	1.801818	
Gabon	Deaths	Female	2013	3.812927	5.599356	1.947377	
Gabon	Deaths	Female	2014	3.95546	5.838975	2.030675	
Gabon	Deaths	Female	2015	4.082221	6.359267	2.149253	
Gabon	Deaths	Female	2016	4.105248	6.42192	2.09399	

Gabon	Deaths	Female	2017	4.124799	6.462313	2.109258	
Gabon	Deaths	Female	2018	4.154515	6.449412	2.107363	
Gabon	Deaths	Female	2019	4.24545	6.550929	2.168604	
Gabon	Deaths	Female	2020	4.414825	6.96438	2.318249	
Gabon	Deaths	Female	2021	4.606369	7.351727	2.447262	
Gabon	Deaths	Female	2022	5.225787	8.474165	2.798942	
Gabon	Deaths	Female	2023	5.503869	9.020248	2.929197	
Gabon	DALYs	Male	2010	152.5412	231.6275	75.32569	
Gabon	DALYs	Male	2011	154.5855	228.6884	76.18866	
Gabon	DALYs	Male	2012	157.5063	237.9058	76.48674	
Gabon	DALYs	Male	2013	164.7007	249.7422	82.39592	
Gabon	DALYs	Male	2014	170.8949	254.4795	84.59865	
Gabon	DALYs	Male	2015	179.8557	271.6594	89.92444	
Gabon	DALYs	Male	2016	182.9814	269.7088	92.18849	
Gabon	DALYs	Male	2017	185.0357	272.3366	91.43547	
Gabon	DALYs	Male	2018	188.77	282.7638	93.53477	
Gabon	DALYs	Male	2019	194.6067	294.9423	96.56975	
Gabon	DALYs	Male	2020	201.5294	309.1539	98.19072	
Gabon	DALYs	Male	2021	206.0066	322.6248	100.4224	
Gabon	DALYs	Male	2022	230.7983	342.8207	112.1242	
Gabon	DALYs	Male	2023	241.9577	365.3226	126.5601	
Gabon	DALYs	Female	2010	85.66424	130.2889	45.27474	
Gabon	DALYs	Female	2011	86.59278	127.4001	45.64718	
Gabon	DALYs	Female	2012	92.43598	136.8086	48.76358	
Gabon	DALYs	Female	2013	101.5798	153.4103	54.29709	
Gabon	DALYs	Female	2014	106.4931	161.6495	56.69152	
Gabon	DALYs	Female	2015	109.7052	171.5121	57.37053	
Gabon	DALYs	Female	2016	109.7996	175.8095	57.96767	
Gabon	DALYs	Female	2017	109.7902	175.1264	58.24576	
Gabon	DALYs	Female	2018	110.1045	173.1581	58.4958	
Gabon	DALYs	Female	2019	112.7683	175.511	61.03995	
Gabon	DALYs	Female	2020	117.3091	183.8995	64.76267	
Gabon	DALYs	Female	2021	123.0956	198.9207	68.54499	
Gabon	DALYs	Female	2022	141.7936	234.29	80.47656	
Gabon	DALYs	Female	2023	150.3417	243.6988	82.78873	
The Republic of	Incidence	Male	2010	1.261145	1.792923	0.805048	

The Republic of	Incidence	Male	2011	1.149037	1.621846	0.743935	
The Republic of	Incidence	Male	2012	1.170741	1.665256	0.754204	
The Republic of	Incidence	Male	2013	1.131043	1.618045	0.75461	
The Republic of	Incidence	Male	2014	1.216602	1.772844	0.783434	
The Republic of	Incidence	Male	2015	1.225048	1.761108	0.809264	
The Republic of	Incidence	Male	2016	1.290722	1.841135	0.842991	
The Republic of	Incidence	Male	2017	1.450399	2.09912	0.937133	
The Republic of	Incidence	Male	2018	1.416324	2.039507	0.904678	
The Republic of	Incidence	Male	2019	1.499871	2.214003	0.941637	
The Republic of	Incidence	Male	2020	1.550972	2.281686	0.968594	
The Republic of	Incidence	Male	2021	1.572671	2.315113	0.994049	
The Republic of	Incidence	Male	2022	1.650309	2.414091	1.012278	
The Republic of	Incidence	Male	2023	1.771971	2.585842	1.080062	
The Republic of	Incidence	Female	2010	0.292242	0.418199	0.192625	
The Republic of	Incidence	Female	2011	0.269208	0.393759	0.176965	
The Republic of	Incidence	Female	2012	0.278561	0.410101	0.180028	
The Republic of	Incidence	Female	2013	0.280233	0.414917	0.179592	
The Republic of	Incidence	Female	2014	0.306001	0.441292	0.196139	
The Republic of	Incidence	Female	2015	0.300081	0.420313	0.192318	
The Republic of	Incidence	Female	2016	0.327199	0.463682	0.209076	
The Republic of	Incidence	Female	2017	0.381167	0.536921	0.249146	
The Republic of	Incidence	Female	2018	0.380667	0.539291	0.248441	
The Republic of	Incidence	Female	2019	0.41851	0.59522	0.281866	
The Republic of	Incidence	Female	2020	0.405726	0.563847	0.275993	
The Republic of	Incidence	Female	2021	0.426068	0.594238	0.292453	
The Republic of	Incidence	Female	2022	0.486403	0.689182	0.313637	
The Republic of	Incidence	Female	2023	0.512834	0.724604	0.328263	
The Republic of	Deaths	Male	2010	1.275134	1.809364	0.817645	
The Republic of	Deaths	Male	2011	1.15967	1.637429	0.756829	
The Republic of	Deaths	Male	2012	1.181832	1.685657	0.763636	
The Republic of	Deaths	Male	2013	1.143835	1.639203	0.761971	
The Republic of	Deaths	Male	2014	1.231087	1.794101	0.79548	
The Republic of	Deaths	Male	2015	1.240359	1.784203	0.82212	
The Republic of	Deaths	Male	2016	1.304956	1.864214	0.852894	
The Republic of	Deaths	Male	2017	1.465341	2.12818	0.943385	
The Republic of	Deaths	Male	2018	1.430335	2.055825	0.911917	

The Republic of	Deaths	Male	2019	1.513104	2.235947	0.956391	
The Republic of	Deaths	Male	2020	1.571386	2.29282	0.990365	
The Republic of	Deaths	Male	2021	1.585416	2.335219	1.006308	
The Republic of	Deaths	Male	2022	1.660841	2.423128	1.025275	
The Republic of	Deaths	Male	2023	1.77949	2.59338	1.083228	
The Republic of	Deaths	Female	2010	0.288751	0.415016	0.19057	
The Republic of	Deaths	Female	2011	0.264794	0.387695	0.173576	
The Republic of	Deaths	Female	2012	0.273163	0.402635	0.175899	
The Republic of	Deaths	Female	2013	0.275838	0.409215	0.178472	
The Republic of	Deaths	Female	2014	0.301474	0.430847	0.196211	
The Republic of	Deaths	Female	2015	0.296344	0.415177	0.191795	
The Republic of	Deaths	Female	2016	0.322481	0.457651	0.206136	
The Republic of	Deaths	Female	2017	0.374862	0.525393	0.245516	
The Republic of	Deaths	Female	2018	0.374097	0.529593	0.247805	
The Republic of	Deaths	Female	2019	0.411045	0.583929	0.27749	
The Republic of	Deaths	Female	2020	0.399082	0.546272	0.274674	
The Republic of	Deaths	Female	2021	0.417206	0.584718	0.287112	
The Republic of	Deaths	Female	2022	0.474299	0.667508	0.306643	
The Republic of	Deaths	Female	2023	0.498163	0.708676	0.32156	
The Republic of	DALYs	Male	2010	33.65096	47.55194	21.33242	
The Republic of	DALYs	Male	2011	30.78179	43.42718	19.63403	
The Republic of	DALYs	Male	2012	31.33748	44.33467	20.30944	
The Republic of	DALYs	Male	2013	30.15674	43.45544	20.18322	
The Republic of	DALYs	Male	2014	32.42071	47.23183	21.00961	
The Republic of	DALYs	Male	2015	32.66212	46.62846	21.53376	
The Republic of	DALYs	Male	2016	34.51291	48.58159	22.47756	
The Republic of	DALYs	Male	2017	38.79407	55.83478	24.97996	
The Republic of	DALYs	Male	2018	37.93168	54.78396	24.56838	
The Republic of	DALYs	Male	2019	40.21172	59.16798	25.11595	
The Republic of	DALYs	Male	2020	41.40096	61.66232	25.72106	
The Republic of	DALYs	Male	2021	42.34353	62.39196	26.66683	
The Republic of	DALYs	Male	2022	44.35572	64.96705	26.76081	
The Republic of	DALYs	Male	2023	47.73767	69.87103	29.13309	
The Republic of	DALYs	Female	2010	8.478256	12.40909	5.552065	
The Republic of	DALYs	Female	2011	7.884134	11.39501	5.205481	
The Republic of	DALYs	Female	2012	8.211355	11.94328	5.33276	

The Republic of	DALYs	Female	2013	8.195355	12.08323	5.220193	
The Republic of	DALYs	Female	2014	8.940901	12.83495	5.708463	
The Republic of	DALYs	Female	2015	8.729057	12.28904	5.547962	
The Republic of	DALYs	Female	2016	9.555956	13.6595	6.038461	
The Republic of	DALYs	Female	2017	11.15697	15.89269	7.166963	
The Republic of	DALYs	Female	2018	11.15983	15.96932	7.170219	
The Republic of	DALYs	Female	2019	12.25232	17.45301	8.042183	
The Republic of	DALYs	Female	2020	11.85193	16.52246	7.904941	
The Republic of	DALYs	Female	2021	12.50159	17.61249	8.469165	
The Republic of	DALYs	Female	2022	14.37237	20.68652	9.366799	
The Republic of	DALYs	Female	2023	15.25974	21.949	9.693068	
Georgia	Incidence	Male	2010	66.62623	72.80151	59.81854	
Georgia	Incidence	Male	2011	74.81022	81.86321	67.61706	
Georgia	Incidence	Male	2012	77.98378	84.16558	70.69658	
Georgia	Incidence	Male	2013	78.95712	85.76256	71.57738	
Georgia	Incidence	Male	2014	80.16319	86.12981	73.47267	
Georgia	Incidence	Male	2015	87.56807	94.6669	80.89261	
Georgia	Incidence	Male	2016	91.60979	99.38995	83.58465	
Georgia	Incidence	Male	2017	88.38367	96.86366	80.66546	
Georgia	Incidence	Male	2018	83.10919	92.51933	75.06945	
Georgia	Incidence	Male	2019	76.18952	84.32683	68.24342	
Georgia	Incidence	Male	2020	74.89748	83.09247	66.62107	
Georgia	Incidence	Male	2021	74.01754	82.50832	65.18985	
Georgia	Incidence	Male	2022	72.50137	82.36273	62.92042	
Georgia	Incidence	Male	2023	72.92224	85.90791	62.23397	
Georgia	Incidence	Female	2010	11.51774	13.77035	9.954809	
Georgia	Incidence	Female	2011	11.3926	13.37703	9.808296	
Georgia	Incidence	Female	2012	11.5015	13.48028	9.812324	
Georgia	Incidence	Female	2013	11.30862	13.17314	9.768145	
Georgia	Incidence	Female	2014	11.84887	13.77814	10.19077	
Georgia	Incidence	Female	2015	12.33694	14.85521	10.72044	
Georgia	Incidence	Female	2016	12.17605	14.36822	10.49637	
Georgia	Incidence	Female	2017	12.75806	14.95261	11.02655	
Georgia	Incidence	Female	2018	12.41743	14.45513	10.81052	
Georgia	Incidence	Female	2019	11.61387	13.76397	10.18354	
Georgia	Incidence	Female	2020	12.33527	14.60777	10.89587	

Georgia	Incidence	Female	2021	11.8118	13.89037	10.40801	
Georgia	Incidence	Female	2022	11.41963	13.52222	9.791642	
Georgia	Incidence	Female	2023	11.16433	13.21986	9.152321	
Georgia	Deaths	Male	2010	65.09093	71.43443	58.37356	
Georgia	Deaths	Male	2011	73.51605	80.44131	66.34205	
Georgia	Deaths	Male	2012	76.72345	82.84766	69.37892	
Georgia	Deaths	Male	2013	77.87889	84.14164	70.48501	
Georgia	Deaths	Male	2014	79.13989	84.99348	72.4242	
Georgia	Deaths	Male	2015	86.10837	93.22236	79.42128	
Georgia	Deaths	Male	2016	90.23567	97.16169	82.24436	
Georgia	Deaths	Male	2017	86.9768	95.16253	79.34755	
Georgia	Deaths	Male	2018	82.01485	91.24402	73.7478	
Georgia	Deaths	Male	2019	75.32805	83.68044	67.20329	
Georgia	Deaths	Male	2020	74.05553	82.31028	65.97835	
Georgia	Deaths	Male	2021	73.28892	81.46219	64.64105	
Georgia	Deaths	Male	2022	71.73376	81.14537	62.32602	
Georgia	Deaths	Male	2023	71.84441	84.5226	61.21125	
Georgia	Deaths	Female	2010	11.73331	13.96298	10.14956	
Georgia	Deaths	Female	2011	11.69576	13.74323	10.12843	
Georgia	Deaths	Female	2012	11.84441	13.91196	10.18246	
Georgia	Deaths	Female	2013	11.69028	13.64157	10.16691	
Georgia	Deaths	Female	2014	12.29259	14.29247	10.65863	
Georgia	Deaths	Female	2015	12.8467	15.46796	11.15197	
Georgia	Deaths	Female	2016	12.69483	15.04822	11.01783	
Georgia	Deaths	Female	2017	13.3535	15.64949	11.57701	
Georgia	Deaths	Female	2018	13.00851	15.19688	11.35378	
Georgia	Deaths	Female	2019	12.16876	14.43572	10.65145	
Georgia	Deaths	Female	2020	12.88266	15.2599	11.36609	
Georgia	Deaths	Female	2021	12.29442	14.49529	10.80798	
Georgia	Deaths	Female	2022	11.91415	14.10925	10.21663	
Georgia	Deaths	Female	2023	11.59143	13.82821	9.579681	
Georgia	DALYs	Male	2010	1773.826	1955.418	1595.613	
Georgia	DALYs	Male	2011	1963.72	2144.479	1760.381	
Georgia	DALYs	Male	2012	2042.78	2220.906	1848.91	
Georgia	DALYs	Male	2013	2059.529	2234.228	1864.954	
Georgia	DALYs	Male	2014	2082.877	2239.506	1913.717	

Georgia	DALYs	Male	2015	2291.203	2481.948	2112.747	
Georgia	DALYs	Male	2016	2380.972	2561.074	2175.096	
Georgia	DALYs	Male	2017	2293.155	2506.472	2091.138	
Georgia	DALYs	Male	2018	2137.487	2380.954	1926.665	
Georgia	DALYs	Male	2019	1940.272	2150.21	1730.829	
Georgia	DALYs	Male	2020	1899.214	2106.178	1690.958	
Georgia	DALYs	Male	2021	1866.8	2075.459	1640.263	
Georgia	DALYs	Male	2022	1827.226	2089.191	1594.272	
Georgia	DALYs	Male	2023	1843.427	2152.455	1573.821	
Georgia	DALYs	Female	2010	282.6606	338.9911	243.2006	
Georgia	DALYs	Female	2011	275.9794	323.1385	237.6566	
Georgia	DALYs	Female	2012	277.1318	326.5375	236.4125	
Georgia	DALYs	Female	2013	269.3098	314.6489	231.2915	
Georgia	DALYs	Female	2014	280.434	325.5376	241.6526	
Georgia	DALYs	Female	2015	289.557	347.5322	250.6846	
Georgia	DALYs	Female	2016	285.2411	338.3128	246.1325	
Georgia	DALYs	Female	2017	294.9836	346.1576	254.2073	
Georgia	DALYs	Female	2018	286.3491	334.919	248.891	
Georgia	DALYs	Female	2019	266.2908	315.3416	232.4831	
Georgia	DALYs	Female	2020	282.1567	334.772	248.9266	
Georgia	DALYs	Female	2021	271.2604	319.6181	238.9407	
Georgia	DALYs	Female	2022	260.7316	309.5725	225.1613	
Georgia	DALYs	Female	2023	255.4275	301.5843	211.2147	
Germany	Incidence	Male	2010	100.2574	105.3573	94.66053	
Germany	Incidence	Male	2011	100.2934	105.9491	94.10628	
Germany	Incidence	Male	2012	100.3231	105.3395	94.16482	
Germany	Incidence	Male	2013	102.7919	107.6842	96.81099	
Germany	Incidence	Male	2014	101.7234	107.3718	95.41387	
Germany	Incidence	Male	2015	100.2564	106.4726	95.02709	
Germany	Incidence	Male	2016	99.39146	105.6986	93.53574	
Germany	Incidence	Male	2017	98.52728	104.6866	93.26642	
Germany	Incidence	Male	2018	98.30072	105.2242	92.76075	
Germany	Incidence	Male	2019	95.94021	103.2106	90.4219	
Germany	Incidence	Male	2020	96.14268	103.9812	90.87481	
Germany	Incidence	Male	2021	96.00344	104.0227	90.33629	
Germany	Incidence	Male	2022	96.88809	104.9157	90.75076	

Germany	Incidence	Male	2023	98.58095	108.4248	91.3456	
Germany	Incidence	Female	2010	47.89537	52.24994	43.56779	
Germany	Incidence	Female	2011	49.42513	53.76505	44.90948	
Germany	Incidence	Female	2012	51.29653	55.40272	46.62055	
Germany	Incidence	Female	2013	53.66262	58.04443	48.61672	
Germany	Incidence	Female	2014	54.6985	58.78791	49.74949	
Germany	Incidence	Female	2015	56.13439	60.04846	50.61995	
Germany	Incidence	Female	2016	57.29424	61.3689	52.21262	
Germany	Incidence	Female	2017	57.75216	61.7675	51.80631	
Germany	Incidence	Female	2018	58.85488	63.29519	53.04	
Germany	Incidence	Female	2019	59.31698	63.42681	53.47016	
Germany	Incidence	Female	2020	60.31054	64.36202	54.72701	
Germany	Incidence	Female	2021	61.87563	65.46372	56.39443	
Germany	Incidence	Female	2022	64.10784	68.17121	58.55314	
Germany	Incidence	Female	2023	64.11497	69.50151	57.6798	
Germany	Deaths	Male	2010	83.81849	87.57089	79.43813	
Germany	Deaths	Male	2011	83.67136	87.44821	79.07798	
Germany	Deaths	Male	2012	83.52547	86.88267	79.1869	
Germany	Deaths	Male	2013	85.38466	89.25616	80.76903	
Germany	Deaths	Male	2014	84.41553	88.55919	80.0294	
Germany	Deaths	Male	2015	83.52038	87.69905	78.88944	
Germany	Deaths	Male	2016	83.09508	87.99619	78.78189	
Germany	Deaths	Male	2017	82.64489	87.62767	78.38516	
Germany	Deaths	Male	2018	82.5945	87.33229	78.13432	
Germany	Deaths	Male	2019	80.80347	86.53913	76.34037	
Germany	Deaths	Male	2020	81.00409	87.08698	76.42451	
Germany	Deaths	Male	2021	80.82218	86.96019	75.66578	
Germany	Deaths	Male	2022	81.83164	88.60733	76.1842	
Germany	Deaths	Male	2023	83.51188	91.80833	77.25799	
Germany	Deaths	Female	2010	38.09123	40.91084	34.38193	
Germany	Deaths	Female	2011	39.04484	41.98028	35.30785	
Germany	Deaths	Female	2012	40.39614	43.32638	36.63553	
Germany	Deaths	Female	2013	42.10908	44.96477	38.29671	
Germany	Deaths	Female	2014	42.8192	45.73836	38.71103	
Germany	Deaths	Female	2015	44.10698	46.86648	39.81211	
Germany	Deaths	Female	2016	45.16601	48.13012	40.84211	

Germany	Deaths	Female	2017	45.69365	48.88296	41.19936	
Germany	Deaths	Female	2018	46.70828	50.11708	42.02045	
Germany	Deaths	Female	2019	47.17636	50.66859	42.39526	
Germany	Deaths	Female	2020	48.03041	51.27762	43.37168	
Germany	Deaths	Female	2021	49.26894	52.36794	44.38997	
Germany	Deaths	Female	2022	51.24098	54.55674	46.56511	
Germany	Deaths	Female	2023	51.47024	56.01998	45.74263	
Germany	DALYs	Male	2010	1900.724	1985.796	1813.866	
Germany	DALYs	Male	2011	1884.278	1964.329	1792.417	
Germany	DALYs	Male	2012	1867.86	1937.311	1771.606	
Germany	DALYs	Male	2013	1900.836	1985.066	1803.168	
Germany	DALYs	Male	2014	1874.976	1963.009	1780.529	
Germany	DALYs	Male	2015	1839.167	1925.365	1759.139	
Germany	DALYs	Male	2016	1823.468	1921.187	1742.694	
Germany	DALYs	Male	2017	1797.408	1901.974	1714.461	
Germany	DALYs	Male	2018	1790.327	1895.514	1704.925	
Germany	DALYs	Male	2019	1738.001	1864.764	1652.998	
Germany	DALYs	Male	2020	1737.411	1864.775	1644.541	
Germany	DALYs	Male	2021	1732.053	1864.17	1632.753	
Germany	DALYs	Male	2022	1735.372	1884.57	1629.084	
Germany	DALYs	Male	2023	1763.086	1932.633	1643.865	
Germany	DALYs	Female	2010	859.9434	916.6746	795.8002	
Germany	DALYs	Female	2011	883.0875	944.7662	817.1442	
Germany	DALYs	Female	2012	906.8388	969.584	837.3675	
Germany	DALYs	Female	2013	939.0405	998.3119	864.7231	
Germany	DALYs	Female	2014	953.0135	1012.398	878.9869	
Germany	DALYs	Female	2015	973.8569	1028.233	895.0544	
Germany	DALYs	Female	2016	992.6179	1052.29	913.284	
Germany	DALYs	Female	2017	994.0948	1054.349	914.0192	
Germany	DALYs	Female	2018	1008.68	1069.262	930.5021	
Germany	DALYs	Female	2019	1013.109	1069.207	930.3576	
Germany	DALYs	Female	2020	1024.496	1078.013	943.5852	
Germany	DALYs	Female	2021	1047.051	1097.864	972.1381	
Germany	DALYs	Female	2022	1078.34	1133.339	1007.289	
Germany	DALYs	Female	2023	1077.314	1158.272	982.3008	
Ghana	Incidence	Male	2010	3.626616	5.071427	2.633992	

Ghana	Incidence	Male	2011	3.815734	5.197066	2.759272	
Ghana	Incidence	Male	2012	3.952988	5.464395	2.872864	
Ghana	Incidence	Male	2013	3.960005	5.518351	2.972116	
Ghana	Incidence	Male	2014	4.047329	5.511813	2.989025	
Ghana	Incidence	Male	2015	4.458775	6.04992	3.220568	
Ghana	Incidence	Male	2016	4.533416	6.313	3.250631	
Ghana	Incidence	Male	2017	4.617345	6.44606	3.2446	
Ghana	Incidence	Male	2018	4.682935	6.497854	3.254655	
Ghana	Incidence	Male	2019	4.788189	6.621124	3.256669	
Ghana	Incidence	Male	2020	5.01354	7.140389	3.360283	
Ghana	Incidence	Male	2021	5.253849	7.536414	3.548096	
Ghana	Incidence	Male	2022	5.610978	8.154529	3.873362	
Ghana	Incidence	Male	2023	5.599724	8.10785	3.794759	
Ghana	Incidence	Female	2010	1.459638	2.34613	0.950838	
Ghana	Incidence	Female	2011	1.578405	2.468102	1.025713	
Ghana	Incidence	Female	2012	1.654185	2.582405	1.074262	
Ghana	Incidence	Female	2013	1.688787	2.698773	1.06439	
Ghana	Incidence	Female	2014	1.716996	2.713145	1.050188	
Ghana	Incidence	Female	2015	1.825089	2.889381	1.133462	
Ghana	Incidence	Female	2016	1.903401	2.981412	1.194557	
Ghana	Incidence	Female	2017	1.982351	3.124747	1.255725	
Ghana	Incidence	Female	2018	2.099774	3.303258	1.401739	
Ghana	Incidence	Female	2019	2.22687	3.465643	1.529942	
Ghana	Incidence	Female	2020	2.332081	3.711377	1.604822	
Ghana	Incidence	Female	2021	2.486467	3.997856	1.672488	
Ghana	Incidence	Female	2022	2.864691	4.543693	1.879656	
Ghana	Incidence	Female	2023	3.025932	4.671847	1.936823	
Ghana	Deaths	Male	2010	3.661424	5.058966	2.626366	
Ghana	Deaths	Male	2011	3.849819	5.262569	2.7502	
Ghana	Deaths	Male	2012	3.987539	5.503606	2.896507	
Ghana	Deaths	Male	2013	3.995993	5.581434	2.953286	
Ghana	Deaths	Male	2014	4.081749	5.579652	3.0203	
Ghana	Deaths	Male	2015	4.499875	6.158396	3.219728	
Ghana	Deaths	Male	2016	4.575473	6.377795	3.296317	
Ghana	Deaths	Male	2017	4.660154	6.552593	3.277548	
Ghana	Deaths	Male	2018	4.719329	6.548439	3.260354	

Ghana	Deaths	Male	2019	4.820901	6.673956	3.230882	
Ghana	Deaths	Male	2020	5.050837	7.24546	3.349188	
Ghana	Deaths	Male	2021	5.295379	7.606671	3.541349	
Ghana	Deaths	Male	2022	5.630145	8.196372	3.884553	
Ghana	Deaths	Male	2023	5.61421	8.152001	3.802965	
Ghana	Deaths	Female	2010	1.401225	2.286493	0.91358	
Ghana	Deaths	Female	2011	1.509714	2.402976	0.98219	
Ghana	Deaths	Female	2012	1.577773	2.513043	1.0286	
Ghana	Deaths	Female	2013	1.612636	2.623163	1.015297	
Ghana	Deaths	Female	2014	1.640233	2.618907	1.007438	
Ghana	Deaths	Female	2015	1.742497	2.766731	1.078855	
Ghana	Deaths	Female	2016	1.815898	2.853436	1.134491	
Ghana	Deaths	Female	2017	1.890783	2.997078	1.201057	
Ghana	Deaths	Female	2018	2.000253	3.168717	1.334372	
Ghana	Deaths	Female	2019	2.119724	3.331708	1.460496	
Ghana	Deaths	Female	2020	2.212012	3.563176	1.531461	
Ghana	Deaths	Female	2021	2.347719	3.814842	1.578138	
Ghana	Deaths	Female	2022	2.69747	4.326058	1.771701	
Ghana	Deaths	Female	2023	2.84589	4.473408	1.824441	
Ghana	DALYs	Male	2010	96.46302	141.0495	69.6809	
Ghana	DALYs	Male	2011	101.6811	140.1782	73.06647	
Ghana	DALYs	Male	2012	105.4108	150.3259	79.16323	
Ghana	DALYs	Male	2013	105.4711	148.6024	79.32313	
Ghana	DALYs	Male	2014	107.8658	147.2433	80.76815	
Ghana	DALYs	Male	2015	118.5789	162.9549	86.81307	
Ghana	DALYs	Male	2016	120.4487	166.5579	86.49311	
Ghana	DALYs	Male	2017	122.6309	170.6634	87.33002	
Ghana	DALYs	Male	2018	124.6956	171.0364	86.72643	
Ghana	DALYs	Male	2019	127.6531	178.0345	89.2545	
Ghana	DALYs	Male	2020	133.3615	187.5278	92.59173	
Ghana	DALYs	Male	2021	139.2973	198.3212	95.90756	
Ghana	DALYs	Male	2022	149.6733	214.74	103.0111	
Ghana	DALYs	Male	2023	149.3138	214.8363	99.9105	
Ghana	DALYs	Female	2010	45.93561	69.58907	30.01071	
Ghana	DALYs	Female	2011	49.96475	73.19092	32.6753	
Ghana	DALYs	Female	2012	52.59013	76.99542	34.44202	

Ghana	DALYs	Female	2013	53.44611	80.93875	33.9437	
Ghana	DALYs	Female	2014	54.16157	82.59787	33.41008	
Ghana	DALYs	Female	2015	57.5263	89.96091	35.47533	
Ghana	DALYs	Female	2016	59.96454	92.63992	37.52116	
Ghana	DALYs	Female	2017	62.38698	96.37496	39.60315	
Ghana	DALYs	Female	2018	66.22315	100.2962	43.85738	
Ghana	DALYs	Female	2019	70.15062	106.6202	48.42662	
Ghana	DALYs	Female	2020	73.66336	112.9136	50.68318	
Ghana	DALYs	Female	2021	78.89465	121.3892	52.92191	
Ghana	DALYs	Female	2022	91.07843	136.9856	59.47672	
Ghana	DALYs	Female	2023	96.29664	143.0682	61.16732	
Greece	Incidence	Male	2010	119.8954	128.2832	111.7273	
Greece	Incidence	Male	2011	124.383	134.7127	115.7438	
Greece	Incidence	Male	2012	125.7005	134.8252	116.8317	
Greece	Incidence	Male	2013	128.327	137.3143	119.2878	
Greece	Incidence	Male	2014	132.806	142.5787	123.0714	
Greece	Incidence	Male	2015	133.8417	144.5858	123.0421	
Greece	Incidence	Male	2016	132.2242	141.9239	121.6622	
Greece	Incidence	Male	2017	133.8312	143.7222	123.252	
Greece	Incidence	Male	2018	130.5969	140.1805	120.3107	
Greece	Incidence	Male	2019	129.2923	138.6366	119.0812	
Greece	Incidence	Male	2020	127.4264	137.909	117.5399	
Greece	Incidence	Male	2021	127.7026	138.7167	116.9799	
Greece	Incidence	Male	2022	126.9887	139.3229	116.2657	
Greece	Incidence	Male	2023	130.4769	143.9895	118.8097	
Greece	Incidence	Female	2010	28.76175	32.08471	25.61386	
Greece	Incidence	Female	2011	29.9883	33.57369	26.5408	
Greece	Incidence	Female	2012	31.65971	35.41616	28.15654	
Greece	Incidence	Female	2013	32.51019	36.12342	28.85582	
Greece	Incidence	Female	2014	33.84983	37.54893	29.96732	
Greece	Incidence	Female	2015	35.22839	38.86166	31.38998	
Greece	Incidence	Female	2016	36.13246	39.80958	32.05205	
Greece	Incidence	Female	2017	36.76268	40.7465	32.32069	
Greece	Incidence	Female	2018	37.1627	41.25917	32.41113	
Greece	Incidence	Female	2019	38.44425	42.88392	34.04268	
Greece	Incidence	Female	2020	40.03471	44.55823	35.5466	

Greece	Incidence	Female	2021	41.70618	46.38963	37.02869	
Greece	Incidence	Female	2022	40.93898	45.75233	35.81601	
Greece	Incidence	Female	2023	41.29991	46.46831	35.48925	
Greece	Deaths	Male	2010	109.3393	114.8684	103.0674	
Greece	Deaths	Male	2011	113.7633	120.4006	107.1578	
Greece	Deaths	Male	2012	115.3342	121.1979	108.8018	
Greece	Deaths	Male	2013	118.1462	123.7973	111.1385	
Greece	Deaths	Male	2014	123.1094	129.7335	116.2152	
Greece	Deaths	Male	2015	125.3032	132.4752	118.3968	
Greece	Deaths	Male	2016	124.4266	130.7217	117.3739	
Greece	Deaths	Male	2017	126.3342	132.6537	119.0636	
Greece	Deaths	Male	2018	123.111	129.413	116.1902	
Greece	Deaths	Male	2019	122.0819	129.4349	114.7395	
Greece	Deaths	Male	2020	120.2436	128.4481	113.4343	
Greece	Deaths	Male	2021	120.4203	129.2326	112.6228	
Greece	Deaths	Male	2022	119.5285	129.0019	110.9091	
Greece	Deaths	Male	2023	122.5171	133.6085	113.4583	
Greece	Deaths	Female	2010	25.49861	27.8218	22.73217	
Greece	Deaths	Female	2011	26.76619	29.15221	23.86756	
Greece	Deaths	Female	2012	28.36089	30.89902	25.35834	
Greece	Deaths	Female	2013	29.09949	31.71354	26.10175	
Greece	Deaths	Female	2014	30.67028	33.41401	27.42774	
Greece	Deaths	Female	2015	32.12402	34.73711	28.73717	
Greece	Deaths	Female	2016	32.99909	35.42107	29.44614	
Greece	Deaths	Female	2017	33.70617	36.21148	29.84954	
Greece	Deaths	Female	2018	33.91756	36.80003	30.20317	
Greece	Deaths	Female	2019	35.21696	38.60295	31.58585	
Greece	Deaths	Female	2020	36.62764	40.34408	32.52213	
Greece	Deaths	Female	2021	37.99844	41.74873	33.97104	
Greece	Deaths	Female	2022	37.41603	41.39088	33.48017	
Greece	Deaths	Female	2023	37.55724	42.20286	32.83832	
Greece	DALYs	Male	2010	2427.839	2548.144	2290.892	
Greece	DALYs	Male	2011	2515.541	2656.196	2381.843	
Greece	DALYs	Male	2012	2534.935	2665.001	2405.038	
Greece	DALYs	Male	2013	2577.451	2697.118	2435.826	
Greece	DALYs	Male	2014	2647.997	2781.779	2499.556	

Greece	DALYs	Male	2015	2646.265	2784.347	2502.019	
Greece	DALYs	Male	2016	2606.932	2735.511	2464.027	
Greece	DALYs	Male	2017	2624.074	2751.08	2493.172	
Greece	DALYs	Male	2018	2554.584	2686.687	2418.276	
Greece	DALYs	Male	2019	2516.289	2651.229	2372.146	
Greece	DALYs	Male	2020	2476.018	2612.663	2349.119	
Greece	DALYs	Male	2021	2484.976	2669.502	2345.222	
Greece	DALYs	Male	2022	2465.567	2680.363	2300.572	
Greece	DALYs	Male	2023	2520.984	2756.301	2337.709	
Greece	DALYs	Female	2010	559.3006	604.7278	508.1091	
Greece	DALYs	Female	2011	581.7608	627.3041	528.0315	
Greece	DALYs	Female	2012	611.702	660.8437	556.9087	
Greece	DALYs	Female	2013	630.7054	680.0286	578.0186	
Greece	DALYs	Female	2014	650.5258	700.4341	594.877	
Greece	DALYs	Female	2015	674.4594	722.7321	620.003	
Greece	DALYs	Female	2016	692.3044	736.3698	637.7612	
Greece	DALYs	Female	2017	698.8845	745.7273	638.5426	
Greece	DALYs	Female	2018	706.3813	757.1098	643.796	
Greece	DALYs	Female	2019	727.8648	782.9446	666.5617	
Greece	DALYs	Female	2020	758.2335	815.5409	692.1099	
Greece	DALYs	Female	2021	789.0536	849.4728	726.1532	
Greece	DALYs	Female	2022	768.6904	837.7301	695.5687	
Greece	DALYs	Female	2023	774.5835	860.2132	691.4591	
Guam	Incidence	Male	2010	34.91616	40.13614	30.95444	
Guam	Incidence	Male	2011	37.5603	43.06068	33.22166	
Guam	Incidence	Male	2012	38.22127	44.13029	33.47991	
Guam	Incidence	Male	2013	43.22149	49.81885	37.46856	
Guam	Incidence	Male	2014	43.32761	50.14581	37.73116	
Guam	Incidence	Male	2015	44.26235	51.61409	38.36615	
Guam	Incidence	Male	2016	43.26203	50.12702	37.82362	
Guam	Incidence	Male	2017	38.40825	44.52317	33.68588	
Guam	Incidence	Male	2018	51.7133	59.09757	45.39072	
Guam	Incidence	Male	2019	49.35142	57.15981	43.49078	
Guam	Incidence	Male	2020	51.41889	59.63397	45.02577	
Guam	Incidence	Male	2021	47.0752	54.95095	40.48266	
Guam	Incidence	Male	2022	46.74062	54.25923	40.18819	

Guam	Incidence	Male	2023	47.79695	54.73553	41.43702	
Guam	Incidence	Female	2010	19.017	22.73064	15.86963	
Guam	Incidence	Female	2011	18.90749	22.68921	15.76146	
Guam	Incidence	Female	2012	19.69808	23.23314	16.43007	
Guam	Incidence	Female	2013	22.18842	25.88021	18.78609	
Guam	Incidence	Female	2014	23.27451	27.17776	19.61703	
Guam	Incidence	Female	2015	23.92125	27.89719	20.01753	
Guam	Incidence	Female	2016	23.75058	27.89076	20.01447	
Guam	Incidence	Female	2017	23.22452	27.30137	19.60827	
Guam	Incidence	Female	2018	25.72795	30.29631	21.99502	
Guam	Incidence	Female	2019	29.20229	33.95861	24.69506	
Guam	Incidence	Female	2020	31.06709	35.93033	26.14557	
Guam	Incidence	Female	2021	27.53712	32.44753	23.03348	
Guam	Incidence	Female	2022	27.58811	32.56838	23.04629	
Guam	Incidence	Female	2023	29.10719	34.62734	23.38074	
Guam	Deaths	Male	2010	34.21414	39.30341	30.30831	
Guam	Deaths	Male	2011	36.80953	42.02821	32.65346	
Guam	Deaths	Male	2012	37.46121	43.28112	32.76795	
Guam	Deaths	Male	2013	42.66212	49.08332	37.04243	
Guam	Deaths	Male	2014	42.68175	49.33403	37.21952	
Guam	Deaths	Male	2015	43.41208	50.45469	37.71261	
Guam	Deaths	Male	2016	42.63279	49.42445	37.33979	
Guam	Deaths	Male	2017	37.85405	43.77554	33.15673	
Guam	Deaths	Male	2018	50.98397	58.14249	44.78059	
Guam	Deaths	Male	2019	48.5461	56.07527	42.84333	
Guam	Deaths	Male	2020	50.36496	58.2151	44.22786	
Guam	Deaths	Male	2021	46.19356	53.62777	39.85761	
Guam	Deaths	Male	2022	45.8349	53.23153	39.2542	
Guam	Deaths	Male	2023	46.95095	53.94443	40.74828	
Guam	Deaths	Female	2010	18.37211	21.70392	15.43228	
Guam	Deaths	Female	2011	18.23774	21.71128	15.2409	
Guam	Deaths	Female	2012	19.01719	22.40919	15.82889	
Guam	Deaths	Female	2013	21.45256	24.9044	18.0736	
Guam	Deaths	Female	2014	22.50697	26.14541	19.10125	
Guam	Deaths	Female	2015	23.08407	26.97278	19.40026	
Guam	Deaths	Female	2016	22.89411	26.72314	19.24549	

Guam	Deaths	Female	2017	22.50129	26.2596	19.04826	
Guam	Deaths	Female	2018	24.85943	29.19328	21.25927	
Guam	Deaths	Female	2019	28.19626	32.70805	23.82266	
Guam	Deaths	Female	2020	29.91584	34.5576	25.14995	
Guam	Deaths	Female	2021	26.52394	31.18858	22.10238	
Guam	Deaths	Female	2022	26.50628	31.13301	22.21035	
Guam	Deaths	Female	2023	28.01036	33.23345	22.52316	
Guam	DALYs	Male	2010	919.3556	1063.214	810.9016	
Guam	DALYs	Male	2011	988.998	1138.71	871.9511	
Guam	DALYs	Male	2012	1007.633	1170.817	878.8059	
Guam	DALYs	Male	2013	1123.367	1302.619	976.4809	
Guam	DALYs	Male	2014	1130.808	1320.211	978.1067	
Guam	DALYs	Male	2015	1163.955	1359.171	1012.741	
Guam	DALYs	Male	2016	1133.386	1313.15	990.6642	
Guam	DALYs	Male	2017	1005.975	1169.397	878.5626	
Guam	DALYs	Male	2018	1345.019	1540.606	1184.766	
Guam	DALYs	Male	2019	1274.861	1476.22	1122.851	
Guam	DALYs	Male	2020	1330.818	1546.126	1170.674	
Guam	DALYs	Male	2021	1205.739	1404.335	1040.957	
Guam	DALYs	Male	2022	1190.994	1377.609	1015.093	
Guam	DALYs	Male	2023	1206.192	1392.173	1042.674	
Guam	DALYs	Female	2010	471.773	560.9613	392.4551	
Guam	DALYs	Female	2011	471.939	560.7929	391.1238	
Guam	DALYs	Female	2012	488.6865	575.8462	405.1787	
Guam	DALYs	Female	2013	548.2651	637.9466	462.4565	
Guam	DALYs	Female	2014	574.5942	666.1611	486.6406	
Guam	DALYs	Female	2015	595.4609	695.5871	499.5043	
Guam	DALYs	Female	2016	592.4203	693.2895	495.9267	
Guam	DALYs	Female	2017	575.4626	673.5757	481.7481	
Guam	DALYs	Female	2018	638.9576	750.7514	540.9388	
Guam	DALYs	Female	2019	717.9541	832.8291	611.2021	
Guam	DALYs	Female	2020	755.434	874.8485	637.4332	
Guam	DALYs	Female	2021	664.1058	777.8838	555.2486	
Guam	DALYs	Female	2022	664.6778	782.6754	560.4164	
Guam	DALYs	Female	2023	692.5899	821.6329	559.6481	
Guatemala	Incidence	Male	2010	4.481629	5.057297	3.989364	

Guatemala	Incidence	Male	2011	4.27725	4.827518	3.805199	
Guatemala	Incidence	Male	2012	4.186524	4.751708	3.741624	
Guatemala	Incidence	Male	2013	4.10099	4.630263	3.622355	
Guatemala	Incidence	Male	2014	3.982415	4.449104	3.55225	
Guatemala	Incidence	Male	2015	4.08081	4.570892	3.648911	
Guatemala	Incidence	Male	2016	4.077971	4.551366	3.647449	
Guatemala	Incidence	Male	2017	4.095651	4.586363	3.674588	
Guatemala	Incidence	Male	2018	4.2241	4.714005	3.812933	
Guatemala	Incidence	Male	2019	4.208226	4.683781	3.76947	
Guatemala	Incidence	Male	2020	4.256717	4.748051	3.797027	
Guatemala	Incidence	Male	2021	4.202082	4.6702	3.696228	
Guatemala	Incidence	Male	2022	3.925949	4.46242	3.408115	
Guatemala	Incidence	Male	2023	4.126484	4.990563	3.349661	
Guatemala	Incidence	Female	2010	3.622524	4.081358	3.111535	
Guatemala	Incidence	Female	2011	3.613689	4.08277	3.152759	
Guatemala	Incidence	Female	2012	3.410914	3.827748	2.954662	
Guatemala	Incidence	Female	2013	3.41536	3.83591	2.976203	
Guatemala	Incidence	Female	2014	3.46176	3.854391	3.033035	
Guatemala	Incidence	Female	2015	3.504633	3.957342	3.085386	
Guatemala	Incidence	Female	2016	3.657809	4.109951	3.241732	
Guatemala	Incidence	Female	2017	3.747076	4.200846	3.310698	
Guatemala	Incidence	Female	2018	3.839952	4.332626	3.336981	
Guatemala	Incidence	Female	2019	3.866852	4.301927	3.394164	
Guatemala	Incidence	Female	2020	3.816833	4.255626	3.313871	
Guatemala	Incidence	Female	2021	3.988852	4.473092	3.434803	
Guatemala	Incidence	Female	2022	3.958671	4.493933	3.423483	
Guatemala	Incidence	Female	2023	3.963469	4.662414	3.299197	
Guatemala	Deaths	Male	2010	4.578203	5.151388	4.070145	
Guatemala	Deaths	Male	2011	4.365862	4.909822	3.872337	
Guatemala	Deaths	Male	2012	4.272702	4.83016	3.825792	
Guatemala	Deaths	Male	2013	4.190485	4.726377	3.718973	
Guatemala	Deaths	Male	2014	4.070538	4.558916	3.624764	
Guatemala	Deaths	Male	2015	4.187392	4.653866	3.762086	
Guatemala	Deaths	Male	2016	4.167156	4.660931	3.736958	
Guatemala	Deaths	Male	2017	4.182868	4.663948	3.74814	
Guatemala	Deaths	Male	2018	4.30768	4.766558	3.901306	

Guatemala	Deaths	Male	2019	4.303872	4.794835	3.87353	
Guatemala	Deaths	Male	2020	4.350929	4.857392	3.880917	
Guatemala	Deaths	Male	2021	4.294575	4.736529	3.781936	
Guatemala	Deaths	Male	2022	3.988506	4.559709	3.452987	
Guatemala	Deaths	Male	2023	4.176509	5.020727	3.376803	
Guatemala	Deaths	Female	2010	3.504465	3.938944	2.994975	
Guatemala	Deaths	Female	2011	3.482467	3.947297	3.020529	
Guatemala	Deaths	Female	2012	3.297512	3.715457	2.851993	
Guatemala	Deaths	Female	2013	3.309479	3.719334	2.881378	
Guatemala	Deaths	Female	2014	3.341823	3.75721	2.943107	
Guatemala	Deaths	Female	2015	3.373867	3.771339	2.958074	
Guatemala	Deaths	Female	2016	3.512772	3.936082	3.113002	
Guatemala	Deaths	Female	2017	3.620618	4.036656	3.18773	
Guatemala	Deaths	Female	2018	3.703569	4.167391	3.239205	
Guatemala	Deaths	Female	2019	3.7486	4.188927	3.306302	
Guatemala	Deaths	Female	2020	3.682845	4.113599	3.215977	
Guatemala	Deaths	Female	2021	3.816504	4.247296	3.336188	
Guatemala	Deaths	Female	2022	3.778251	4.288213	3.288901	
Guatemala	Deaths	Female	2023	3.754194	4.39032	3.154818	
Guatemala	DALYs	Male	2010	113.4535	128.9391	99.31999	
Guatemala	DALYs	Male	2011	108.9911	123.153	96.39948	
Guatemala	DALYs	Male	2012	106.2201	121.3435	93.86727	
Guatemala	DALYs	Male	2013	103.815	118.6104	91.30135	
Guatemala	DALYs	Male	2014	100.9875	114.567	90.1033	
Guatemala	DALYs	Male	2015	102.3537	115.4944	91.57275	
Guatemala	DALYs	Male	2016	103.3069	116.5591	92.0525	
Guatemala	DALYs	Male	2017	103.8465	116.5462	92.74308	
Guatemala	DALYs	Male	2018	106.6496	120.2562	96.17021	
Guatemala	DALYs	Male	2019	105.0391	117.6947	93.85996	
Guatemala	DALYs	Male	2020	105.1984	118.8289	93.19023	
Guatemala	DALYs	Male	2021	102.8276	114.7511	89.92993	
Guatemala	DALYs	Male	2022	96.83277	111.1421	82.92186	
Guatemala	DALYs	Male	2023	102.1513	121.8933	82.31827	
Guatemala	DALYs	Female	2010	91.91092	103.2979	78.8661	
Guatemala	DALYs	Female	2011	92.19168	104.4986	79.82982	
Guatemala	DALYs	Female	2012	86.69843	98.12669	74.98058	

Guatemala	DALYs	Female	2013	86.10791	97.01918	75.02324	
Guatemala	DALYs	Female	2014	87.72169	98.21997	76.91573	
Guatemala	DALYs	Female	2015	88.82721	100.263	77.69757	
Guatemala	DALYs	Female	2016	92.66326	104.2041	82.30055	
Guatemala	DALYs	Female	2017	93.74478	105.3694	82.04926	
Guatemala	DALYs	Female	2018	95.50296	107.0842	82.96041	
Guatemala	DALYs	Female	2019	94.92015	106.2308	83.63201	
Guatemala	DALYs	Female	2020	93.02204	103.7721	80.80501	
Guatemala	DALYs	Female	2021	97.30748	108.9585	83.7974	
Guatemala	DALYs	Female	2022	96.48663	109.4157	83.59514	
Guatemala	DALYs	Female	2023	96.88188	113.0985	81.19305	
Guinea	Incidence	Male	2010	3.65404	4.895784	2.552562	
Guinea	Incidence	Male	2011	3.653703	4.949543	2.527842	
Guinea	Incidence	Male	2012	3.650159	4.954526	2.533625	
Guinea	Incidence	Male	2013	3.501189	4.767559	2.51404	
Guinea	Incidence	Male	2014	3.488438	4.649405	2.462888	
Guinea	Incidence	Male	2015	3.606872	4.878592	2.577512	
Guinea	Incidence	Male	2016	3.550895	4.858668	2.52738	
Guinea	Incidence	Male	2017	3.556388	5.155334	2.527262	
Guinea	Incidence	Male	2018	3.563516	5.187283	2.437127	
Guinea	Incidence	Male	2019	3.581628	5.369317	2.392202	
Guinea	Incidence	Male	2020	3.585585	5.250521	2.420643	
Guinea	Incidence	Male	2021	3.627222	5.15592	2.465638	
Guinea	Incidence	Male	2022	4.003334	5.539712	2.743675	
Guinea	Incidence	Male	2023	4.064243	5.844511	2.786101	
Guinea	Incidence	Female	2010	0.999457	1.480311	0.701166	
Guinea	Incidence	Female	2011	1.000231	1.460733	0.683212	
Guinea	Incidence	Female	2012	1.007251	1.447905	0.671959	
Guinea	Incidence	Female	2013	0.999739	1.408672	0.670336	
Guinea	Incidence	Female	2014	1.002733	1.438895	0.653398	
Guinea	Incidence	Female	2015	1.014316	1.430275	0.661269	
Guinea	Incidence	Female	2016	1.012354	1.373458	0.669867	
Guinea	Incidence	Female	2017	1.017084	1.378357	0.684796	
Guinea	Incidence	Female	2018	1.038727	1.406082	0.720121	
Guinea	Incidence	Female	2019	1.091132	1.459358	0.76299	
Guinea	Incidence	Female	2020	1.084556	1.470765	0.745728	

Guinea	Incidence	Female	2021	1.11286	1.514398	0.764588	
Guinea	Incidence	Female	2022	1.346279	1.88812	0.882386	
Guinea	Incidence	Female	2023	1.413488	1.908289	0.947287	
Guinea	Deaths	Male	2010	3.69842	4.948499	2.591608	
Guinea	Deaths	Male	2011	3.692683	5.034509	2.578438	
Guinea	Deaths	Male	2012	3.685622	5.058594	2.578063	
Guinea	Deaths	Male	2013	3.53497	4.799731	2.522642	
Guinea	Deaths	Male	2014	3.518563	4.715675	2.460909	
Guinea	Deaths	Male	2015	3.636694	4.907388	2.589307	
Guinea	Deaths	Male	2016	3.577612	4.880558	2.54467	
Guinea	Deaths	Male	2017	3.578756	5.180022	2.539584	
Guinea	Deaths	Male	2018	3.582098	5.207105	2.45273	
Guinea	Deaths	Male	2019	3.598511	5.406207	2.403205	
Guinea	Deaths	Male	2020	3.607521	5.279515	2.417038	
Guinea	Deaths	Male	2021	3.644687	5.183654	2.498733	
Guinea	Deaths	Male	2022	4.021463	5.600927	2.785279	
Guinea	Deaths	Male	2023	4.079745	5.857033	2.800292	
Guinea	Deaths	Female	2010	0.988029	1.466131	0.69191	
Guinea	Deaths	Female	2011	0.985931	1.435498	0.673994	
Guinea	Deaths	Female	2012	0.989214	1.412839	0.661002	
Guinea	Deaths	Female	2013	0.978849	1.380116	0.659059	
Guinea	Deaths	Female	2014	0.978685	1.404207	0.641079	
Guinea	Deaths	Female	2015	0.988189	1.395426	0.642667	
Guinea	Deaths	Female	2016	0.98422	1.339803	0.648309	
Guinea	Deaths	Female	2017	0.986473	1.337624	0.665952	
Guinea	Deaths	Female	2018	1.003784	1.363388	0.694076	
Guinea	Deaths	Female	2019	1.049579	1.406837	0.743644	
Guinea	Deaths	Female	2020	1.040685	1.411596	0.725597	
Guinea	Deaths	Female	2021	1.063051	1.446581	0.730862	
Guinea	Deaths	Female	2022	1.281122	1.796375	0.847512	
Guinea	Deaths	Female	2023	1.341937	1.810166	0.900534	
Guinea	DALYs	Male	2010	99.02679	133.57	68.10059	
Guinea	DALYs	Male	2011	99.38107	134.0995	68.73207	
Guinea	DALYs	Male	2012	99.44411	132.6361	68.48379	
Guinea	DALYs	Male	2013	95.3037	130.9557	68.4066	
Guinea	DALYs	Male	2014	95.10595	127.4561	68.11837	

Guinea	DALYs	Male	2015	98.362	132.9989	69.91254	
Guinea	DALYs	Male	2016	96.87296	133.4572	69.00991	
Guinea	DALYs	Male	2017	97.17462	140.0712	67.99572	
Guinea	DALYs	Male	2018	97.55264	142.6092	66.58979	
Guinea	DALYs	Male	2019	98.01412	146.257	66.28564	
Guinea	DALYs	Male	2020	97.86543	143.2715	66.68989	
Guinea	DALYs	Male	2021	99.20995	141.0609	67.1299	
Guinea	DALYs	Male	2022	109.1973	152.8532	74.82402	
Guinea	DALYs	Male	2023	110.9174	161.3538	74.89128	
Guinea	DALYs	Female	2010	29.16058	43.07865	20.53726	
Guinea	DALYs	Female	2011	29.38613	42.89611	19.91197	
Guinea	DALYs	Female	2012	29.82036	42.85931	19.90675	
Guinea	DALYs	Female	2013	29.77535	41.65298	19.71889	
Guinea	DALYs	Female	2014	30.04274	42.75077	19.42008	
Guinea	DALYs	Female	2015	30.49416	43.04053	19.73831	
Guinea	DALYs	Female	2016	30.51722	41.75737	19.93496	
Guinea	DALYs	Female	2017	30.75985	42.0452	20.95972	
Guinea	DALYs	Female	2018	31.62561	43.19229	21.6909	
Guinea	DALYs	Female	2019	33.50783	45.30093	23.09474	
Guinea	DALYs	Female	2020	33.43785	45.11753	22.65868	
Guinea	DALYs	Female	2021	34.56475	47.11122	23.36516	
Guinea	DALYs	Female	2022	41.96361	58.49442	27.12942	
Guinea	DALYs	Female	2023	44.22691	59.77568	29.04202	
Guinea-Bissau	Incidence	Male	2010	1.933533	2.861666	1.305056	
Guinea-Bissau	Incidence	Male	2011	1.951096	2.866875	1.326836	
Guinea-Bissau	Incidence	Male	2012	1.990751	2.931383	1.369928	
Guinea-Bissau	Incidence	Male	2013	1.912613	2.780731	1.287794	
Guinea-Bissau	Incidence	Male	2014	1.960476	2.843587	1.346408	
Guinea-Bissau	Incidence	Male	2015	2.097087	3.141244	1.448178	
Guinea-Bissau	Incidence	Male	2016	2.134786	3.228562	1.408436	
Guinea-Bissau	Incidence	Male	2017	2.211569	3.238295	1.469128	
Guinea-Bissau	Incidence	Male	2018	2.222134	3.169956	1.497454	
Guinea-Bissau	Incidence	Male	2019	2.280945	3.348126	1.532274	
Guinea-Bissau	Incidence	Male	2020	2.399488	3.612056	1.602035	
Guinea-Bissau	Incidence	Male	2021	2.319486	3.508396	1.526773	
Guinea-Bissau	Incidence	Male	2022	2.610413	4.027071	1.731402	

Guinea-Bissau	Incidence	Male	2023	2.685122	3.896918	1.770647	
Guinea-Bissau	Incidence	Female	2010	0.946743	1.362094	0.637975	
Guinea-Bissau	Incidence	Female	2011	0.978343	1.39144	0.663451	
Guinea-Bissau	Incidence	Female	2012	1.025218	1.47376	0.680073	
Guinea-Bissau	Incidence	Female	2013	1.041976	1.511384	0.682169	
Guinea-Bissau	Incidence	Female	2014	1.05084	1.540248	0.690292	
Guinea-Bissau	Incidence	Female	2015	1.094971	1.573292	0.722226	
Guinea-Bissau	Incidence	Female	2016	1.143474	1.590814	0.77018	
Guinea-Bissau	Incidence	Female	2017	1.200548	1.67771	0.816075	
Guinea-Bissau	Incidence	Female	2018	1.2493	1.729477	0.862268	
Guinea-Bissau	Incidence	Female	2019	1.314801	1.814572	0.914195	
Guinea-Bissau	Incidence	Female	2020	1.257389	1.72606	0.873045	
Guinea-Bissau	Incidence	Female	2021	1.22332	1.657621	0.836862	
Guinea-Bissau	Incidence	Female	2022	1.484498	1.996565	1.022388	
Guinea-Bissau	Incidence	Female	2023	1.579888	2.121445	1.101279	
Guinea-Bissau	Deaths	Male	2010	1.954214	2.87876	1.323597	
Guinea-Bissau	Deaths	Male	2011	1.967856	2.874639	1.344762	
Guinea-Bissau	Deaths	Male	2012	2.005917	2.938701	1.387127	
Guinea-Bissau	Deaths	Male	2013	1.927555	2.809809	1.299067	
Guinea-Bissau	Deaths	Male	2014	1.971594	2.854641	1.35311	
Guinea-Bissau	Deaths	Male	2015	2.103048	3.134003	1.455195	
Guinea-Bissau	Deaths	Male	2016	2.13642	3.224093	1.411401	
Guinea-Bissau	Deaths	Male	2017	2.21147	3.234661	1.481795	
Guinea-Bissau	Deaths	Male	2018	2.222517	3.164652	1.499565	
Guinea-Bissau	Deaths	Male	2019	2.27722	3.327324	1.5341	
Guinea-Bissau	Deaths	Male	2020	2.399502	3.601202	1.604905	
Guinea-Bissau	Deaths	Male	2021	2.316075	3.495109	1.52614	
Guinea-Bissau	Deaths	Male	2022	2.604354	4.006694	1.727561	
Guinea-Bissau	Deaths	Male	2023	2.673826	3.853061	1.759936	
Guinea-Bissau	Deaths	Female	2010	0.929431	1.340538	0.623803	
Guinea-Bissau	Deaths	Female	2011	0.957235	1.362904	0.648454	
Guinea-Bissau	Deaths	Female	2012	0.997691	1.431582	0.662525	
Guinea-Bissau	Deaths	Female	2013	1.011481	1.4588	0.664187	
Guinea-Bissau	Deaths	Female	2014	1.019021	1.483864	0.669421	
Guinea-Bissau	Deaths	Female	2015	1.05867	1.518687	0.700895	
Guinea-Bissau	Deaths	Female	2016	1.10204	1.530459	0.73708	

Guinea-Bissau	Deaths	Female	2017	1.154194	1.60583	0.788132	
Guinea-Bissau	Deaths	Female	2018	1.197144	1.660668	0.827379	
Guinea-Bissau	Deaths	Female	2019	1.25505	1.726735	0.877166	
Guinea-Bissau	Deaths	Female	2020	1.201437	1.639209	0.834896	
Guinea-Bissau	Deaths	Female	2021	1.169792	1.580674	0.800546	
Guinea-Bissau	Deaths	Female	2022	1.414474	1.911074	0.970913	
Guinea-Bissau	Deaths	Female	2023	1.502092	2.024234	1.041654	
Guinea-Bissau	DALYs	Male	2010	52.68187	78.83895	35.65524	
Guinea-Bissau	DALYs	Male	2011	53.38455	79.48195	35.94361	
Guinea-Bissau	DALYs	Male	2012	54.5245	81.17836	37.02909	
Guinea-Bissau	DALYs	Male	2013	52.28317	75.90828	35.06032	
Guinea-Bissau	DALYs	Male	2014	53.77334	78.22818	36.10474	
Guinea-Bissau	DALYs	Male	2015	57.82791	85.45889	39.50781	
Guinea-Bissau	DALYs	Male	2016	59.10191	89.51544	38.92606	
Guinea-Bissau	DALYs	Male	2017	61.26926	89.97587	40.35075	
Guinea-Bissau	DALYs	Male	2018	61.44642	88.4742	41.15005	
Guinea-Bissau	DALYs	Male	2019	63.25719	93.96729	41.92232	
Guinea-Bissau	DALYs	Male	2020	66.44503	100.764	43.92938	
Guinea-Bissau	DALYs	Male	2021	64.39616	98.29886	41.99198	
Guinea-Bissau	DALYs	Male	2022	72.40173	112.3305	47.56089	
Guinea-Bissau	DALYs	Male	2023	74.69612	110.8883	49.39749	
Guinea-Bissau	DALYs	Female	2010	28.39862	41.18885	19.20208	
Guinea-Bissau	DALYs	Female	2011	29.55213	41.85104	19.88538	
Guinea-Bissau	DALYs	Female	2012	31.31828	45.33262	20.73159	
Guinea-Bissau	DALYs	Female	2013	31.97009	46.68085	20.89566	
Guinea-Bissau	DALYs	Female	2014	32.27218	47.35144	20.8493	
Guinea-Bissau	DALYs	Female	2015	33.792	48.59395	22.18262	
Guinea-Bissau	DALYs	Female	2016	35.47041	49.97013	23.68551	
Guinea-Bissau	DALYs	Female	2017	37.37814	52.91777	24.91994	
Guinea-Bissau	DALYs	Female	2018	39.11831	54.93914	26.75073	
Guinea-Bissau	DALYs	Female	2019	41.46168	57.64385	28.6059	
Guinea-Bissau	DALYs	Female	2020	39.55839	55.32334	27.42503	
Guinea-Bissau	DALYs	Female	2021	38.40943	52.62138	26.29053	
Guinea-Bissau	DALYs	Female	2022	46.78617	64.19732	32.10347	
Guinea-Bissau	DALYs	Female	2023	49.96809	67.44903	34.25241	
Guyana	Incidence	Male	2010	6.394291	7.678695	5.556241	

Guyana	Incidence	Male	2011	6.427785	7.65919	5.602968	
Guyana	Incidence	Male	2012	6.498142	7.742968	5.4936	
Guyana	Incidence	Male	2013	6.887865	8.159495	5.8875	
Guyana	Incidence	Male	2014	6.827028	8.030754	5.769886	
Guyana	Incidence	Male	2015	6.755006	7.888816	5.706869	
Guyana	Incidence	Male	2016	6.675952	7.892069	5.639822	
Guyana	Incidence	Male	2017	6.539078	7.96687	5.542445	
Guyana	Incidence	Male	2018	6.71695	8.247523	5.735048	
Guyana	Incidence	Male	2019	6.167424	7.50265	5.211786	
Guyana	Incidence	Male	2020	7.218094	8.602927	6.075852	
Guyana	Incidence	Male	2021	7.928733	9.559134	6.604887	
Guyana	Incidence	Male	2022	8.325592	9.945894	6.772414	
Guyana	Incidence	Male	2023	9.518579	11.83892	7.644191	
Guyana	Incidence	Female	2010	3.669161	4.418034	3.012295	
Guyana	Incidence	Female	2011	3.850107	4.662019	3.173007	
Guyana	Incidence	Female	2012	3.893748	4.717143	3.180104	
Guyana	Incidence	Female	2013	3.963825	4.806621	3.185475	
Guyana	Incidence	Female	2014	4.11201	5.005186	3.342784	
Guyana	Incidence	Female	2015	4.165676	5.001667	3.36365	
Guyana	Incidence	Female	2016	4.082862	4.828132	3.349904	
Guyana	Incidence	Female	2017	4.023391	4.771293	3.334447	
Guyana	Incidence	Female	2018	4.248418	5.14303	3.512692	
Guyana	Incidence	Female	2019	4.435085	5.504315	3.708789	
Guyana	Incidence	Female	2020	5.215994	6.507794	4.34119	
Guyana	Incidence	Female	2021	5.94098	7.245004	4.904949	
Guyana	Incidence	Female	2022	6.477973	8.300372	5.043441	
Guyana	Incidence	Female	2023	7.606381	9.57013	5.712252	
Guyana	Deaths	Male	2010	6.237095	7.513619	5.420304	
Guyana	Deaths	Male	2011	6.275304	7.506381	5.442918	
Guyana	Deaths	Male	2012	6.333244	7.588688	5.33958	
Guyana	Deaths	Male	2013	6.70652	7.932155	5.719073	
Guyana	Deaths	Male	2014	6.637393	7.801311	5.615664	
Guyana	Deaths	Male	2015	6.561256	7.671174	5.55356	
Guyana	Deaths	Male	2016	6.5007	7.710716	5.490294	
Guyana	Deaths	Male	2017	6.371141	7.763236	5.419783	
Guyana	Deaths	Male	2018	6.56715	8.063465	5.611321	

Guyana	Deaths	Male	2019	6.03688	7.317725	5.103068	
Guyana	Deaths	Male	2020	7.093034	8.458009	5.974103	
Guyana	Deaths	Male	2021	7.728829	9.313212	6.448531	
Guyana	Deaths	Male	2022	8.100446	9.718477	6.635948	
Guyana	Deaths	Male	2023	9.218722	11.47441	7.393966	
Guyana	Deaths	Female	2010	3.565936	4.282922	2.928588	
Guyana	Deaths	Female	2011	3.747879	4.528029	3.074054	
Guyana	Deaths	Female	2012	3.787275	4.571439	3.099138	
Guyana	Deaths	Female	2013	3.852651	4.651995	3.111553	
Guyana	Deaths	Female	2014	3.989626	4.829068	3.259915	
Guyana	Deaths	Female	2015	4.053852	4.85935	3.280971	
Guyana	Deaths	Female	2016	3.991017	4.726241	3.277038	
Guyana	Deaths	Female	2017	3.938954	4.675131	3.261043	
Guyana	Deaths	Female	2018	4.164115	5.028585	3.428413	
Guyana	Deaths	Female	2019	4.35469	5.414605	3.645192	
Guyana	Deaths	Female	2020	5.136505	6.406377	4.248495	
Guyana	Deaths	Female	2021	5.767919	7.077106	4.784385	
Guyana	Deaths	Female	2022	6.290149	8.031147	4.877886	
Guyana	Deaths	Female	2023	7.366962	9.278689	5.521568	
Guyana	DALYs	Male	2010	180.4514	214.6613	155.5801	
Guyana	DALYs	Male	2011	180.967	214.6507	156.4865	
Guyana	DALYs	Male	2012	183.3236	218.8233	155.4037	
Guyana	DALYs	Male	2013	194.6641	231.4032	165.9519	
Guyana	DALYs	Male	2014	193.3709	228.4193	162.6601	
Guyana	DALYs	Male	2015	191.5291	223.6069	161.4585	
Guyana	DALYs	Male	2016	187.6035	221.1049	158.1443	
Guyana	DALYs	Male	2017	182.897	222.5132	154.2634	
Guyana	DALYs	Male	2018	185.7938	228.6736	157.5934	
Guyana	DALYs	Male	2019	170.7453	209.0779	143.9691	
Guyana	DALYs	Male	2020	198.2977	239.2875	165.4854	
Guyana	DALYs	Male	2021	218.8131	263.7942	182.8226	
Guyana	DALYs	Male	2022	228.713	275.6345	184.9066	
Guyana	DALYs	Male	2023	260.8002	322.9268	207.8519	
Guyana	DALYs	Female	2010	102.4721	123.4675	84.25185	
Guyana	DALYs	Female	2011	107.0777	129.8747	88.17971	
Guyana	DALYs	Female	2012	108.3002	130.6953	88.82855	

Guyana	DALYs	Female	2013	110.2481	134.7333	88.5536	
Guyana	DALYs	Female	2014	114.7042	139.488	92.89463	
Guyana	DALYs	Female	2015	115.2723	139.1679	93.65089	
Guyana	DALYs	Female	2016	111.7426	132.229	91.67958	
Guyana	DALYs	Female	2017	109.5503	130.5166	90.26971	
Guyana	DALYs	Female	2018	115.0315	139.6588	95.67219	
Guyana	DALYs	Female	2019	119.8316	147.6051	100.2822	
Guyana	DALYs	Female	2020	139.8473	172.2782	116.6347	
Guyana	DALYs	Female	2021	161.7262	198.6314	134.8381	
Guyana	DALYs	Female	2022	174.1111	222.302	135.3593	
Guyana	DALYs	Female	2023	202.011	253.5887	152.5116	
Haiti	Incidence	Male	2010	5.214712	7.499974	3.583905	
Haiti	Incidence	Male	2011	5.262783	7.62708	3.676077	
Haiti	Incidence	Male	2012	5.353827	7.831236	3.840198	
Haiti	Incidence	Male	2013	5.49674	7.982054	3.908984	
Haiti	Incidence	Male	2014	5.584277	8.038407	3.880922	
Haiti	Incidence	Male	2015	5.631557	8.101911	3.770811	
Haiti	Incidence	Male	2016	5.723769	8.351172	3.717687	
Haiti	Incidence	Male	2017	5.787069	8.567156	3.79247	
Haiti	Incidence	Male	2018	5.916439	8.529027	3.909309	
Haiti	Incidence	Male	2019	6.076784	9.056898	3.953023	
Haiti	Incidence	Male	2020	6.178959	9.293956	4.080091	
Haiti	Incidence	Male	2021	6.572071	10.31034	4.460393	
Haiti	Incidence	Male	2022	6.91426	10.52999	4.753608	
Haiti	Incidence	Male	2023	7.189468	10.81133	4.844323	
Haiti	Incidence	Female	2010	2.433319	3.81729	1.572091	
Haiti	Incidence	Female	2011	2.501761	3.931543	1.624251	
Haiti	Incidence	Female	2012	2.595728	4.124863	1.738856	
Haiti	Incidence	Female	2013	2.71076	4.254609	1.718034	
Haiti	Incidence	Female	2014	2.770892	4.172023	1.742631	
Haiti	Incidence	Female	2015	2.831971	4.2719	1.784711	
Haiti	Incidence	Female	2016	2.925909	4.457694	1.894429	
Haiti	Incidence	Female	2017	3.022587	4.546733	1.968203	
Haiti	Incidence	Female	2018	3.163897	4.635636	2.149568	
Haiti	Incidence	Female	2019	3.312281	4.852943	2.329257	
Haiti	Incidence	Female	2020	3.336337	4.946286	2.309452	

Haiti	Incidence	Female	2021	3.619495	5.317969	2.430832	
Haiti	Incidence	Female	2022	3.950978	5.740715	2.603865	
Haiti	Incidence	Female	2023	4.163218	6.148037	2.723012	
Haiti	Deaths	Male	2010	5.215034	7.533903	3.585085	
Haiti	Deaths	Male	2011	5.255652	7.573762	3.666292	
Haiti	Deaths	Male	2012	5.341909	7.719259	3.835444	
Haiti	Deaths	Male	2013	5.478176	7.972418	3.914247	
Haiti	Deaths	Male	2014	5.55759	7.988353	3.864782	
Haiti	Deaths	Male	2015	5.598686	8.033495	3.772518	
Haiti	Deaths	Male	2016	5.686712	8.245282	3.700344	
Haiti	Deaths	Male	2017	5.742742	8.446154	3.770573	
Haiti	Deaths	Male	2018	5.862452	8.498632	3.870464	
Haiti	Deaths	Male	2019	6.013862	8.911013	3.917603	
Haiti	Deaths	Male	2020	6.106592	9.172702	4.021019	
Haiti	Deaths	Male	2021	6.478343	10.10135	4.417324	
Haiti	Deaths	Male	2022	6.814471	10.39912	4.698013	
Haiti	Deaths	Male	2023	7.080214	10.47957	4.78801	
Haiti	Deaths	Female	2010	2.40544	3.778129	1.544649	
Haiti	Deaths	Female	2011	2.469916	3.907468	1.604331	
Haiti	Deaths	Female	2012	2.559932	4.084509	1.726005	
Haiti	Deaths	Female	2013	2.669755	4.192801	1.694211	
Haiti	Deaths	Female	2014	2.724828	4.094571	1.722877	
Haiti	Deaths	Female	2015	2.782557	4.209321	1.755382	
Haiti	Deaths	Female	2016	2.873344	4.342638	1.864519	
Haiti	Deaths	Female	2017	2.96083	4.447087	1.924069	
Haiti	Deaths	Female	2018	3.089246	4.561169	2.097796	
Haiti	Deaths	Female	2019	3.227649	4.715432	2.2686	
Haiti	Deaths	Female	2020	3.243933	4.821337	2.252422	
Haiti	Deaths	Female	2021	3.503635	5.127186	2.361236	
Haiti	Deaths	Female	2022	3.820009	5.534451	2.526738	
Haiti	Deaths	Female	2023	4.01931	5.91831	2.63659	
Haiti	DALYs	Male	2010	142.9356	202.5251	98.31944	
Haiti	DALYs	Male	2011	144.5447	214.0323	101.5195	
Haiti	DALYs	Male	2012	147.3028	217.8517	104.7068	
Haiti	DALYs	Male	2013	151.5265	220.675	106.481	
Haiti	DALYs	Male	2014	154.3563	223.976	107.3151	

Haiti	DALYs	Male	2015	155.8402	225.9115	103.2249	
Haiti	DALYs	Male	2016	158.5149	233.4352	104.2392	
Haiti	DALYs	Male	2017	160.7953	239.457	105.2593	
Haiti	DALYs	Male	2018	164.9545	241.3055	108.9872	
Haiti	DALYs	Male	2019	169.6791	253.2123	111.267	
Haiti	DALYs	Male	2020	172.5952	262.1899	114.2004	
Haiti	DALYs	Male	2021	184.1173	289.4528	124.1649	
Haiti	DALYs	Male	2022	193.5205	295.0148	132.5944	
Haiti	DALYs	Male	2023	201.4356	309.9882	134.7769	
Haiti	DALYs	Female	2010	67.86042	105.5408	45.02129	
Haiti	DALYs	Female	2011	69.82212	107.5739	45.60047	
Haiti	DALYs	Female	2012	72.54902	113.4093	48.01362	
Haiti	DALYs	Female	2013	75.92979	118.992	47.67809	
Haiti	DALYs	Female	2014	77.83004	117.1326	48.30344	
Haiti	DALYs	Female	2015	79.56406	117.8086	50.23305	
Haiti	DALYs	Female	2016	82.22805	125.423	53.83029	
Haiti	DALYs	Female	2017	85.52562	128.1754	56.44406	
Haiti	DALYs	Female	2018	90.23493	130.133	61.23255	
Haiti	DALYs	Female	2019	94.75124	138.3762	66.29905	
Haiti	DALYs	Female	2020	95.61495	140.263	65.35729	
Haiti	DALYs	Female	2021	104.5386	153.6621	70.37498	
Haiti	DALYs	Female	2022	114.0581	165.2401	75.4217	
Haiti	DALYs	Female	2023	120.6558	179.0307	79.83252	
Honduras	Incidence	Male	2010	5.336766	7.527114	3.762106	
Honduras	Incidence	Male	2011	5.464064	7.859107	3.907893	
Honduras	Incidence	Male	2012	5.775313	8.136297	4.144001	
Honduras	Incidence	Male	2013	5.879136	8.493862	4.188119	
Honduras	Incidence	Male	2014	5.954501	8.634513	4.297492	
Honduras	Incidence	Male	2015	6.043719	8.536383	4.243536	
Honduras	Incidence	Male	2016	5.978311	8.393339	4.233494	
Honduras	Incidence	Male	2017	6.067182	8.661564	4.16896	
Honduras	Incidence	Male	2018	6.217981	8.731018	4.115717	
Honduras	Incidence	Male	2019	6.381938	9.151709	4.313792	
Honduras	Incidence	Male	2020	6.130971	8.695265	4.209202	
Honduras	Incidence	Male	2021	6.587723	9.288607	4.555929	
Honduras	Incidence	Male	2022	6.822874	9.727741	4.635265	

Honduras	Incidence	Male	2023	7.133684	10.16062	4.82589	
Honduras	Incidence	Female	2010	5.794627	8.283074	3.963935	
Honduras	Incidence	Female	2011	6.026226	8.486836	4.155368	
Honduras	Incidence	Female	2012	6.188894	8.721571	4.095123	
Honduras	Incidence	Female	2013	6.330976	8.889766	4.172083	
Honduras	Incidence	Female	2014	6.480515	9.02868	4.160128	
Honduras	Incidence	Female	2015	6.636437	9.2199	4.266743	
Honduras	Incidence	Female	2016	6.737325	9.158583	4.40576	
Honduras	Incidence	Female	2017	6.858351	9.623502	4.531645	
Honduras	Incidence	Female	2018	7.048029	10.04069	4.625862	
Honduras	Incidence	Female	2019	7.269371	10.44349	4.846963	
Honduras	Incidence	Female	2020	6.946054	9.388296	4.70042	
Honduras	Incidence	Female	2021	7.800504	10.61334	5.342507	
Honduras	Incidence	Female	2022	8.133914	11.69132	5.412623	
Honduras	Incidence	Female	2023	8.340369	11.91312	5.469014	
Honduras	Deaths	Male	2010	5.53525	7.852122	3.903133	
Honduras	Deaths	Male	2011	5.655531	8.201194	4.010634	
Honduras	Deaths	Male	2012	5.991114	8.408217	4.308916	
Honduras	Deaths	Male	2013	6.106961	8.792373	4.327583	
Honduras	Deaths	Male	2014	6.193464	8.948873	4.470488	
Honduras	Deaths	Male	2015	6.294245	8.871755	4.40307	
Honduras	Deaths	Male	2016	6.238225	8.74938	4.38276	
Honduras	Deaths	Male	2017	6.339089	9.005172	4.323465	
Honduras	Deaths	Male	2018	6.504977	9.107058	4.287912	
Honduras	Deaths	Male	2019	6.684796	9.537685	4.531466	
Honduras	Deaths	Male	2020	6.49219	9.204949	4.435058	
Honduras	Deaths	Male	2021	6.953585	9.82391	4.788077	
Honduras	Deaths	Male	2022	7.190239	10.28536	4.860755	
Honduras	Deaths	Male	2023	7.537566	10.77417	5.076898	
Honduras	Deaths	Female	2010	5.619935	8.023865	3.856892	
Honduras	Deaths	Female	2011	5.832653	8.210098	4.014976	
Honduras	Deaths	Female	2012	5.992559	8.531133	3.922409	
Honduras	Deaths	Female	2013	6.132108	8.632641	4.01897	
Honduras	Deaths	Female	2014	6.278796	8.805197	4.04555	
Honduras	Deaths	Female	2015	6.42906	8.928013	4.147875	
Honduras	Deaths	Female	2016	6.530098	8.949673	4.230167	

Honduras	Deaths	Female	2017	6.650041	9.304365	4.347995	
Honduras	Deaths	Female	2018	6.836698	9.620798	4.522825	
Honduras	Deaths	Female	2019	7.050542	10.12071	4.697748	
Honduras	Deaths	Female	2020	6.756566	9.179903	4.562436	
Honduras	Deaths	Female	2021	7.536332	10.21237	5.170783	
Honduras	Deaths	Female	2022	7.891245	11.3343	5.238478	
Honduras	Deaths	Female	2023	8.115132	11.86763	5.30701	
Honduras	DALYs	Male	2010	135.9838	192.0915	95.73117	
Honduras	DALYs	Male	2011	139.8035	200.5884	100.1202	
Honduras	DALYs	Male	2012	147.6722	210.679	104.7918	
Honduras	DALYs	Male	2013	149.7743	219.0652	105.4724	
Honduras	DALYs	Male	2014	151.0578	219.656	107.3544	
Honduras	DALYs	Male	2015	152.6139	213.8484	107.1493	
Honduras	DALYs	Male	2016	150.3232	212.7119	105.8522	
Honduras	DALYs	Male	2017	151.9903	216.941	105.0605	
Honduras	DALYs	Male	2018	155.0139	220.3871	103.4366	
Honduras	DALYs	Male	2019	158.1171	227.1932	107.7782	
Honduras	DALYs	Male	2020	149.4717	212.6665	103.1123	
Honduras	DALYs	Male	2021	161.1385	225.3838	111.3278	
Honduras	DALYs	Male	2022	165.1665	235.6155	112.244	
Honduras	DALYs	Male	2023	170.9547	241.0995	115.1033	
Honduras	DALYs	Female	2010	154.5322	226.4289	107.2886	
Honduras	DALYs	Female	2011	161.227	231.3035	110.9843	
Honduras	DALYs	Female	2012	166.0564	232.383	109.2909	
Honduras	DALYs	Female	2013	169.4186	235.2686	112.8916	
Honduras	DALYs	Female	2014	172.7683	237.1628	112.5815	
Honduras	DALYs	Female	2015	176.2918	245.0863	114.7135	
Honduras	DALYs	Female	2016	178.1745	244.8597	115.4359	
Honduras	DALYs	Female	2017	180.6606	253.2072	117.7054	
Honduras	DALYs	Female	2018	184.7144	259.061	121.4412	
Honduras	DALYs	Female	2019	189.295	265.2421	128.283	
Honduras	DALYs	Female	2020	179.6208	246.7479	120.4727	
Honduras	DALYs	Female	2021	203.2305	277.6358	140.0495	
Honduras	DALYs	Female	2022	207.3185	291.8176	140.2558	
Honduras	DALYs	Female	2023	209.7153	301.7113	141.6972	
Hungary	Incidence	Male	2010	129.4132	135.8203	123.161	

Hungary	Incidence	Male	2011	128.8354	135.4928	123.1047	
Hungary	Incidence	Male	2012	127.5832	133.1197	121.861	
Hungary	Incidence	Male	2013	124.0103	129.6479	117.7832	
Hungary	Incidence	Male	2014	123.5958	129.0164	117.7668	
Hungary	Incidence	Male	2015	121.6897	127.2289	116.0979	
Hungary	Incidence	Male	2016	122.5246	128.8819	116.6787	
Hungary	Incidence	Male	2017	122.5956	129.2881	116.4967	
Hungary	Incidence	Male	2018	120.831	128.2095	114.0619	
Hungary	Incidence	Male	2019	113.6045	120.4345	108.0714	
Hungary	Incidence	Male	2020	110.5716	117.8152	105.09	
Hungary	Incidence	Male	2021	107.4096	114.3095	101.4549	
Hungary	Incidence	Male	2022	104.9612	113.1712	98.48474	
Hungary	Incidence	Male	2023	103.7345	111.9407	96.15649	
Hungary	Incidence	Female	2010	59.74426	63.08735	56.36046	
Hungary	Incidence	Female	2011	60.94572	64.59517	57.57221	
Hungary	Incidence	Female	2012	63.04221	67.10603	59.04928	
Hungary	Incidence	Female	2013	64.75313	68.83516	60.0243	
Hungary	Incidence	Female	2014	66.76203	70.69589	62.29303	
Hungary	Incidence	Female	2015	67.6916	71.5489	62.78766	
Hungary	Incidence	Female	2016	68.50428	72.43044	63.87079	
Hungary	Incidence	Female	2017	69.7415	73.74579	64.94588	
Hungary	Incidence	Female	2018	69.54189	73.19256	65.16278	
Hungary	Incidence	Female	2019	69.97065	73.32439	65.83888	
Hungary	Incidence	Female	2020	70.13777	73.3444	65.77477	
Hungary	Incidence	Female	2021	69.07092	72.19146	65.23857	
Hungary	Incidence	Female	2022	69.38759	72.8787	65.38669	
Hungary	Incidence	Female	2023	68.46915	72.33939	64.22043	
Hungary	Deaths	Male	2010	125.5663	131.2524	120.3528	
Hungary	Deaths	Male	2011	124.9689	130.6473	119.5438	
Hungary	Deaths	Male	2012	123.7869	129.0087	118.3901	
Hungary	Deaths	Male	2013	120.4037	126.0225	114.7996	
Hungary	Deaths	Male	2014	120.3922	125.5645	115.6856	
Hungary	Deaths	Male	2015	118.7285	124.0474	113.6064	
Hungary	Deaths	Male	2016	119.758	125.5364	114.5165	
Hungary	Deaths	Male	2017	120.0235	125.9614	114.8972	
Hungary	Deaths	Male	2018	118.4604	125.2741	112.6567	

Hungary	Deaths	Male	2019	111.612	117.8922	106.6606	
Hungary	Deaths	Male	2020	108.8457	115.4845	104.1312	
Hungary	Deaths	Male	2021	105.6677	112.299	100.5361	
Hungary	Deaths	Male	2022	103.4104	110.6528	97.17308	
Hungary	Deaths	Male	2023	102.2239	110.281	95.35387	
Hungary	Deaths	Female	2010	58.10323	61.22288	54.83298	
Hungary	Deaths	Female	2011	59.35478	62.69311	56.09239	
Hungary	Deaths	Female	2012	61.50611	65.00688	57.64914	
Hungary	Deaths	Female	2013	63.18957	66.76049	58.66357	
Hungary	Deaths	Female	2014	65.2225	68.62471	61.1135	
Hungary	Deaths	Female	2015	66.37448	69.60872	61.70658	
Hungary	Deaths	Female	2016	67.27151	70.79401	63.11335	
Hungary	Deaths	Female	2017	68.55875	71.92034	64.3309	
Hungary	Deaths	Female	2018	68.5392	71.61818	64.80982	
Hungary	Deaths	Female	2019	69.08655	72.24612	65.25019	
Hungary	Deaths	Female	2020	69.22594	71.8693	65.39256	
Hungary	Deaths	Female	2021	68.11168	70.49342	64.90306	
Hungary	Deaths	Female	2022	68.72524	71.78424	65.02281	
Hungary	Deaths	Female	2023	67.81101	71.35073	63.10639	
Hungary	DALYs	Male	2010	3301.573	3463.541	3178.959	
Hungary	DALYs	Male	2011	3273.735	3413.178	3137.335	
Hungary	DALYs	Male	2012	3220.582	3358.795	3085.742	
Hungary	DALYs	Male	2013	3109.393	3256.164	2956.841	
Hungary	DALYs	Male	2014	3067.651	3203.761	2944.681	
Hungary	DALYs	Male	2015	2999.404	3136.109	2872.717	
Hungary	DALYs	Male	2016	2989.687	3133.807	2865.452	
Hungary	DALYs	Male	2017	2963.36	3114.325	2835.545	
Hungary	DALYs	Male	2018	2896.086	3065.56	2753.479	
Hungary	DALYs	Male	2019	2690.791	2850.432	2575.095	
Hungary	DALYs	Male	2020	2588.001	2748.189	2473.149	
Hungary	DALYs	Male	2021	2493.453	2659.683	2373.127	
Hungary	DALYs	Male	2022	2415.653	2590.981	2273.686	
Hungary	DALYs	Male	2023	2382.285	2579.929	2222.459	
Hungary	DALYs	Female	2010	1484.829	1561.614	1411.363	
Hungary	DALYs	Female	2011	1500.024	1585.733	1422.828	
Hungary	DALYs	Female	2012	1535.676	1623.836	1453.246	

Hungary	DALYs	Female	2013	1567.643	1657.809	1469.835	
Hungary	DALYs	Female	2014	1605.882	1690.051	1509.287	
Hungary	DALYs	Female	2015	1608.759	1693.191	1499.059	
Hungary	DALYs	Female	2016	1613.162	1694.321	1519.942	
Hungary	DALYs	Female	2017	1628.391	1696.053	1541.263	
Hungary	DALYs	Female	2018	1604.153	1679.457	1523.222	
Hungary	DALYs	Female	2019	1593.365	1668.052	1515.085	
Hungary	DALYs	Female	2020	1582.676	1644.135	1509.373	
Hungary	DALYs	Female	2021	1547.407	1602.241	1486.129	
Hungary	DALYs	Female	2022	1530.855	1598.162	1452.887	
Hungary	DALYs	Female	2023	1506.529	1586.857	1416.218	
Iceland	Incidence	Male	2010	55.50352	63.71088	47.75807	
Iceland	Incidence	Male	2011	56.33362	64.44737	48.63135	
Iceland	Incidence	Male	2012	57.22159	65.72223	49.33969	
Iceland	Incidence	Male	2013	55.39629	64.59825	48.37996	
Iceland	Incidence	Male	2014	55.83989	65.17029	48.3636	
Iceland	Incidence	Male	2015	53.22533	61.93842	45.63288	
Iceland	Incidence	Male	2016	55.4998	64.40338	47.98264	
Iceland	Incidence	Male	2017	53.18069	62.3927	45.76	
Iceland	Incidence	Male	2018	50.63888	58.83846	43.16569	
Iceland	Incidence	Male	2019	46.50299	54.98748	39.56087	
Iceland	Incidence	Male	2020	45.30461	53.32817	38.73778	
Iceland	Incidence	Male	2021	44.86336	51.64016	38.33489	
Iceland	Incidence	Male	2022	47.59019	56.71631	40.6957	
Iceland	Incidence	Male	2023	49.0141	57.19726	41.96622	
Iceland	Incidence	Female	2010	60.57878	71.53534	51.6149	
Iceland	Incidence	Female	2011	61.86391	73.1596	52.83245	
Iceland	Incidence	Female	2012	63.52407	76.01034	54.20728	
Iceland	Incidence	Female	2013	63.62468	75.90729	53.25873	
Iceland	Incidence	Female	2014	65.51567	77.55896	55.18338	
Iceland	Incidence	Female	2015	66.29593	77.7879	56.14709	
Iceland	Incidence	Female	2016	67.00891	78.93982	56.52914	
Iceland	Incidence	Female	2017	64.21134	76.08167	53.95219	
Iceland	Incidence	Female	2018	64.11993	76.01858	53.80739	
Iceland	Incidence	Female	2019	63.88029	75.69798	53.39092	
Iceland	Incidence	Female	2020	61.6328	73.0415	52.34106	

Iceland	Incidence	Female	2021	61.55809	71.99322	52.29815	
Iceland	Incidence	Female	2022	67.99104	79.33388	57.66818	
Iceland	Incidence	Female	2023	67.1235	78.52579	57.22594	
Iceland	Deaths	Male	2010	43.80815	48.98472	39.59437	
Iceland	Deaths	Male	2011	44.41366	50.04131	40.03334	
Iceland	Deaths	Male	2012	44.97147	50.8568	40.34518	
Iceland	Deaths	Male	2013	43.34522	49.18582	38.84145	
Iceland	Deaths	Male	2014	43.76765	49.66081	38.96848	
Iceland	Deaths	Male	2015	41.53311	47.14979	36.98302	
Iceland	Deaths	Male	2016	43.12025	48.08382	38.68619	
Iceland	Deaths	Male	2017	40.92841	46.60437	36.61459	
Iceland	Deaths	Male	2018	39.09608	44.3711	34.67188	
Iceland	Deaths	Male	2019	36.07292	40.98204	32.0947	
Iceland	Deaths	Male	2020	35.20591	40.03097	31.2454	
Iceland	Deaths	Male	2021	34.80038	39.60589	30.76094	
Iceland	Deaths	Male	2022	37.04106	42.59254	32.51146	
Iceland	Deaths	Male	2023	38.22114	43.93637	33.82494	
Iceland	Deaths	Female	2010	48.7837	55.97489	42.2643	
Iceland	Deaths	Female	2011	49.78322	57.13835	42.86245	
Iceland	Deaths	Female	2012	50.80021	58.62127	44.02078	
Iceland	Deaths	Female	2013	50.68905	58.77212	43.51144	
Iceland	Deaths	Female	2014	51.76264	59.55639	44.56189	
Iceland	Deaths	Female	2015	52.35984	60.23293	45.43652	
Iceland	Deaths	Female	2016	53.15348	61.69457	46.10646	
Iceland	Deaths	Female	2017	50.87619	58.41714	44.33982	
Iceland	Deaths	Female	2018	50.74281	58.41341	43.78738	
Iceland	Deaths	Female	2019	50.71457	57.82665	43.78306	
Iceland	Deaths	Female	2020	48.65016	55.39835	42.44218	
Iceland	Deaths	Female	2021	48.75382	55.17167	42.20014	
Iceland	Deaths	Female	2022	54.35117	61.6636	47.17413	
Iceland	Deaths	Female	2023	53.19082	60.97805	45.62674	
Iceland	DALYs	Male	2010	947.7433	1058.611	855.1838	
Iceland	DALYs	Male	2011	961.0066	1077.229	864.8699	
Iceland	DALYs	Male	2012	959.7907	1074.988	865.8512	
Iceland	DALYs	Male	2013	937.6086	1060.788	843.5538	
Iceland	DALYs	Male	2014	933.2193	1054.196	841.2816	

Iceland	DALYs	Male	2015	887.7052	1002.048	797.322	
Iceland	DALYs	Male	2016	928.698	1030.387	833.7336	
Iceland	DALYs	Male	2017	896.9714	1008.711	803.4013	
Iceland	DALYs	Male	2018	846.2428	958.1045	757.4811	
Iceland	DALYs	Male	2019	774.6475	873.3116	689.8494	
Iceland	DALYs	Male	2020	751.7343	850.598	671.5371	
Iceland	DALYs	Male	2021	748.3995	846.7587	666.607	
Iceland	DALYs	Male	2022	782.5206	888.705	693.1553	
Iceland	DALYs	Male	2023	809.5949	921.8463	720.166	
Iceland	DALYs	Female	2010	1042.774	1189.083	920.5698	
Iceland	DALYs	Female	2011	1056.621	1217.369	921.596	
Iceland	DALYs	Female	2012	1078.493	1241.499	954.7619	
Iceland	DALYs	Female	2013	1076.296	1241.331	948.1905	
Iceland	DALYs	Female	2014	1104.753	1262.694	979.4821	
Iceland	DALYs	Female	2015	1112.352	1276.015	975.4201	
Iceland	DALYs	Female	2016	1117.725	1287.638	980.9862	
Iceland	DALYs	Female	2017	1072.125	1222.433	954.3075	
Iceland	DALYs	Female	2018	1068.546	1224.44	936.5064	
Iceland	DALYs	Female	2019	1067.645	1212.691	939.8555	
Iceland	DALYs	Female	2020	1036.704	1170.889	914.9247	
Iceland	DALYs	Female	2021	1030.844	1158.002	909.8921	
Iceland	DALYs	Female	2022	1122.547	1262.094	985.7604	
Iceland	DALYs	Female	2023	1121.547	1273.611	975.727	
India	Incidence	Male	2010	6.082706	8.792254	4.897528	
India	Incidence	Male	2011	6.438677	9.456212	5.265879	
India	Incidence	Male	2012	6.717507	9.879943	5.479404	
India	Incidence	Male	2013	6.765555	9.921137	5.52907	
India	Incidence	Male	2014	6.807332	9.629569	5.569639	
India	Incidence	Male	2015	6.973659	9.78738	5.707016	
India	Incidence	Male	2016	7.542492	10.33598	6.120009	
India	Incidence	Male	2017	7.883071	10.58883	6.36916	
India	Incidence	Male	2018	8.188265	10.76744	6.543903	
India	Incidence	Male	2019	8.2173	10.78997	6.38966	
India	Incidence	Male	2020	8.527191	11.36322	6.630433	
India	Incidence	Male	2021	10.5502	13.96464	8.133361	
India	Incidence	Male	2022	9.501803	12.52979	7.24183	

India	Incidence	Male	2023	9.79063	13.03513	7.514533	
India	Incidence	Female	2010	2.085836	2.831089	1.5793	
India	Incidence	Female	2011	2.155286	2.899472	1.648056	
India	Incidence	Female	2012	2.267703	3.014527	1.714994	
India	Incidence	Female	2013	2.429416	3.212431	1.799934	
India	Incidence	Female	2014	2.648968	3.472781	1.961604	
India	Incidence	Female	2015	2.850092	3.654615	2.080349	
India	Incidence	Female	2016	3.062358	3.909527	2.231301	
India	Incidence	Female	2017	3.2315	4.131645	2.406838	
India	Incidence	Female	2018	3.38169	4.356037	2.533257	
India	Incidence	Female	2019	3.518113	4.540814	2.644735	
India	Incidence	Female	2020	3.669619	4.763187	2.751862	
India	Incidence	Female	2021	4.11611	5.389714	3.12921	
India	Incidence	Female	2022	4.233914	5.647851	3.037404	
India	Incidence	Female	2023	4.469563	6.074264	3.317564	
India	Deaths	Male	2010	5.940758	8.553906	4.781145	
India	Deaths	Male	2011	6.286474	9.268192	5.139457	
India	Deaths	Male	2012	6.562969	9.653591	5.345799	
India	Deaths	Male	2013	6.610115	9.706547	5.407716	
India	Deaths	Male	2014	6.644406	9.425431	5.431278	
India	Deaths	Male	2015	6.809327	9.54865	5.569882	
India	Deaths	Male	2016	7.365048	10.08479	5.95897	
India	Deaths	Male	2017	7.699327	10.43144	6.237985	
India	Deaths	Male	2018	7.998188	10.52703	6.411098	
India	Deaths	Male	2019	8.020947	10.51558	6.278478	
India	Deaths	Male	2020	8.322717	11.12961	6.517891	
India	Deaths	Male	2021	10.21033	13.55632	7.858149	
India	Deaths	Male	2022	9.249865	12.17981	7.07658	
India	Deaths	Male	2023	9.521309	12.68182	7.261619	
India	Deaths	Female	2010	2.065043	2.810166	1.563476	
India	Deaths	Female	2011	2.132739	2.871223	1.623701	
India	Deaths	Female	2012	2.243162	2.985436	1.68771	
India	Deaths	Female	2013	2.400895	3.184269	1.781005	
India	Deaths	Female	2014	2.610991	3.434312	1.928626	
India	Deaths	Female	2015	2.807683	3.603949	2.052895	
India	Deaths	Female	2016	3.018396	3.861072	2.199387	

India	Deaths	Female	2017	3.189463	4.069059	2.364496	
India	Deaths	Female	2018	3.342109	4.324607	2.504592	
India	Deaths	Female	2019	3.479883	4.49182	2.614556	
India	Deaths	Female	2020	3.622617	4.695966	2.713479	
India	Deaths	Female	2021	4.031515	5.304641	3.057802	
India	Deaths	Female	2022	4.169475	5.584453	2.977648	
India	Deaths	Female	2023	4.399162	5.983132	3.251185	
India	DALYs	Male	2010	172.1125	250.6731	138.253	
India	DALYs	Male	2011	182.2352	265.3189	148.4304	
India	DALYs	Male	2012	189.9959	277.986	154.7286	
India	DALYs	Male	2013	191.1834	278.7911	155.9712	
India	DALYs	Male	2014	192.373	270.0122	156.9513	
India	DALYs	Male	2015	196.5047	273.7119	160.1548	
India	DALYs	Male	2016	211.8492	288.255	171.1085	
India	DALYs	Male	2017	220.4184	294.5625	177.2464	
India	DALYs	Male	2018	228.1634	297.7628	181.097	
India	DALYs	Male	2019	228.7831	300.6843	177.2979	
India	DALYs	Male	2020	236.4798	312.0344	182.8214	
India	DALYs	Male	2021	295.8356	392.8439	227.9635	
India	DALYs	Male	2022	263.3326	349.1089	199.8232	
India	DALYs	Male	2023	271.0605	359.3222	207.276	
India	DALYs	Female	2010	57.43864	78.2222	43.24979	
India	DALYs	Female	2011	59.33672	79.84172	45.32302	
India	DALYs	Female	2012	62.39916	83.22698	47.07284	
India	DALYs	Female	2013	66.80088	88.1727	49.53683	
India	DALYs	Female	2014	73.02438	95.61262	53.59908	
India	DALYs	Female	2015	78.44406	100.634	57.07943	
India	DALYs	Female	2016	83.93511	107.7413	61.2591	
India	DALYs	Female	2017	87.98081	112.9881	65.24962	
India	DALYs	Female	2018	91.50243	118.7429	68.21079	
India	DALYs	Female	2019	94.66019	121.8776	70.94026	
India	DALYs	Female	2020	98.54805	127.9675	74.18407	
India	DALYs	Female	2021	111.2516	145.8115	84.45203	
India	DALYs	Female	2022	113.3824	150.9646	80.8821	
India	DALYs	Female	2023	119.3337	164.2164	87.61752	
Indonesia	Incidence	Male	2010	15.62638	23.17684	10.77352	

Indonesia	Incidence	Male	2011	16.09393	23.39012	11.34632	
Indonesia	Incidence	Male	2012	16.54753	23.70034	11.80469	
Indonesia	Incidence	Male	2013	16.91509	23.32046	11.9363	
Indonesia	Incidence	Male	2014	17.23909	24.2198	11.87388	
Indonesia	Incidence	Male	2015	17.72682	24.76864	11.79237	
Indonesia	Incidence	Male	2016	18.25466	25.323	12.17332	
Indonesia	Incidence	Male	2017	18.75196	26.24057	12.53213	
Indonesia	Incidence	Male	2018	19.51572	27.27875	12.94164	
Indonesia	Incidence	Male	2019	20.31025	28.74887	13.69038	
Indonesia	Incidence	Male	2020	21.86907	30.55593	14.8659	
Indonesia	Incidence	Male	2021	25.18215	36.74089	16.87151	
Indonesia	Incidence	Male	2022	23.21594	32.36456	15.40654	
Indonesia	Incidence	Male	2023	24.01234	33.99689	15.87674	
Indonesia	Incidence	Female	2010	5.836523	8.41117	3.953489	
Indonesia	Incidence	Female	2011	6.096843	8.525305	4.110531	
Indonesia	Incidence	Female	2012	6.299391	8.899308	4.309931	
Indonesia	Incidence	Female	2013	6.456866	9.048748	4.371114	
Indonesia	Incidence	Female	2014	6.606522	9.108771	4.28801	
Indonesia	Incidence	Female	2015	6.827908	9.436095	4.466177	
Indonesia	Incidence	Female	2016	7.098672	9.849861	4.641033	
Indonesia	Incidence	Female	2017	7.383095	10.35482	5.010197	
Indonesia	Incidence	Female	2018	7.734416	10.53863	5.263451	
Indonesia	Incidence	Female	2019	8.092092	10.94926	5.612661	
Indonesia	Incidence	Female	2020	8.661734	11.79084	5.999513	
Indonesia	Incidence	Female	2021	9.671561	12.94932	6.671892	
Indonesia	Incidence	Female	2022	9.355237	12.30677	6.488039	
Indonesia	Incidence	Female	2023	9.842051	13.5422	6.714746	
Indonesia	Deaths	Male	2010	15.1879	22.43421	10.47221	
Indonesia	Deaths	Male	2011	15.61236	22.6621	11.01361	
Indonesia	Deaths	Male	2012	16.0276	22.99253	11.42603	
Indonesia	Deaths	Male	2013	16.36417	22.48609	11.59216	
Indonesia	Deaths	Male	2014	16.66246	23.32557	11.48786	
Indonesia	Deaths	Male	2015	17.12178	23.8557	11.39539	
Indonesia	Deaths	Male	2016	17.62387	24.43442	11.73133	
Indonesia	Deaths	Male	2017	18.09514	25.23826	12.10596	
Indonesia	Deaths	Male	2018	18.82291	26.29462	12.49131	

Indonesia	Deaths	Male	2019	19.58482	27.70926	13.19179	
Indonesia	Deaths	Male	2020	21.08813	29.49022	14.29348	
Indonesia	Deaths	Male	2021	24.2758	35.29789	16.37765	
Indonesia	Deaths	Male	2022	22.43197	31.27735	14.94156	
Indonesia	Deaths	Male	2023	23.22821	32.83303	15.45481	
Indonesia	Deaths	Female	2010	5.562457	8.04189	3.761874	
Indonesia	Deaths	Female	2011	5.79923	8.148089	3.917997	
Indonesia	Deaths	Female	2012	5.983217	8.498269	4.109771	
Indonesia	Deaths	Female	2013	6.124964	8.618946	4.17592	
Indonesia	Deaths	Female	2014	6.260293	8.633458	4.065282	
Indonesia	Deaths	Female	2015	6.463941	8.916822	4.223186	
Indonesia	Deaths	Female	2016	6.712935	9.317132	4.379058	
Indonesia	Deaths	Female	2017	6.974234	9.770144	4.701787	
Indonesia	Deaths	Female	2018	7.297715	9.891519	5.002942	
Indonesia	Deaths	Female	2019	7.627418	10.2935	5.312471	
Indonesia	Deaths	Female	2020	8.150609	11.12297	5.671046	
Indonesia	Deaths	Female	2021	9.06482	12.0319	6.273629	
Indonesia	Deaths	Female	2022	8.806401	11.59302	6.08871	
Indonesia	Deaths	Female	2023	9.272743	12.72945	6.310191	
Indonesia	DALYs	Male	2010	449.553	671.6082	308.5155	
Indonesia	DALYs	Male	2011	464.3964	686.3961	327.7748	
Indonesia	DALYs	Male	2012	478.4222	676.669	340.4539	
Indonesia	DALYs	Male	2013	489.7238	677.6122	343.3539	
Indonesia	DALYs	Male	2014	499.3466	702.8591	343.5823	
Indonesia	DALYs	Male	2015	513.2573	718.1514	340.6887	
Indonesia	DALYs	Male	2016	528.1248	736.9247	352.139	
Indonesia	DALYs	Male	2017	541.7574	755.6033	361.3234	
Indonesia	DALYs	Male	2018	562.8543	780.3862	369.9515	
Indonesia	DALYs	Male	2019	584.299	819.7907	392.0821	
Indonesia	DALYs	Male	2020	626.4482	874.4377	428.4571	
Indonesia	DALYs	Male	2021	718.1075	1046.312	472.9543	
Indonesia	DALYs	Male	2022	658.7056	915.1417	435.7789	
Indonesia	DALYs	Male	2023	677.8321	968.0283	452.4941	
Indonesia	DALYs	Female	2010	176.3886	252.8884	120.9009	
Indonesia	DALYs	Female	2011	184.6676	258.3758	124.0755	
Indonesia	DALYs	Female	2012	191.0978	268.8888	129.9661	

Indonesia	DALYs	Female	2013	196.1268	275.0676	131.5157	
Indonesia	DALYs	Female	2014	200.8743	278.0499	130.7442	
Indonesia	DALYs	Female	2015	207.6163	287.1596	135.6448	
Indonesia	DALYs	Female	2016	215.7825	300.0473	141.8943	
Indonesia	DALYs	Female	2017	224.2212	315.7402	153.5767	
Indonesia	DALYs	Female	2018	234.5653	322.2445	157.1603	
Indonesia	DALYs	Female	2019	244.8871	331.5779	167.4529	
Indonesia	DALYs	Female	2020	261.7707	359.199	179.6285	
Indonesia	DALYs	Female	2021	293.159	391.5996	200.6185	
Indonesia	DALYs	Female	2022	279.7567	369.9079	189.8769	
Indonesia	DALYs	Female	2023	292.9307	402.1804	199.3079	
Iran, Islamic Re	Incidence	Male	2010	11.74378	16.53217	9.405353	
Iran, Islamic Re	Incidence	Male	2011	11.96057	16.50112	9.482718	
Iran, Islamic Re	Incidence	Male	2012	11.99925	16.20704	9.595777	
Iran, Islamic Re	Incidence	Male	2013	12.28378	16.18631	9.907964	
Iran, Islamic Re	Incidence	Male	2014	12.44251	16.25316	10.13877	
Iran, Islamic Re	Incidence	Male	2015	12.82761	16.83641	10.41898	
Iran, Islamic Re	Incidence	Male	2016	13.24487	17.30461	10.80099	
Iran, Islamic Re	Incidence	Male	2017	13.60324	17.75993	11.24494	
Iran, Islamic Re	Incidence	Male	2018	13.98096	18.33085	11.49498	
Iran, Islamic Re	Incidence	Male	2019	14.36279	18.38402	11.98484	
Iran, Islamic Re	Incidence	Male	2020	14.61398	19.08875	12.38823	
Iran, Islamic Re	Incidence	Male	2021	13.97397	18.1348	11.77606	
Iran, Islamic Re	Incidence	Male	2022	14.37286	18.15131	11.83584	
Iran, Islamic Re	Incidence	Male	2023	14.73701	18.50233	11.90012	
Iran, Islamic Re	Incidence	Female	2010	5.132923	6.87843	3.718828	
Iran, Islamic Re	Incidence	Female	2011	5.301029	7.12319	3.878168	
Iran, Islamic Re	Incidence	Female	2012	5.330571	7.143637	3.852218	
Iran, Islamic Re	Incidence	Female	2013	5.498762	7.383652	4.085766	
Iran, Islamic Re	Incidence	Female	2014	5.598965	7.532025	4.159743	
Iran, Islamic Re	Incidence	Female	2015	5.81082	7.888415	4.274975	
Iran, Islamic Re	Incidence	Female	2016	6.110349	8.328647	4.551153	
Iran, Islamic Re	Incidence	Female	2017	6.325184	8.734805	4.786079	
Iran, Islamic Re	Incidence	Female	2018	6.51991	9.053653	4.897611	
Iran, Islamic Re	Incidence	Female	2019	6.810299	9.496492	5.067439	
Iran, Islamic Re	Incidence	Female	2020	6.85173	9.226788	5.168847	

Iran, Islamic Re	Incidence	Female	2021	6.583839	8.867336	4.965508	
Iran, Islamic Re	Incidence	Female	2022	6.673795	9.246269	4.878391	
Iran, Islamic Re	Incidence	Female	2023	6.943673	9.691745	5.058267	
Iran, Islamic Re	Deaths	Male	2010	11.60442	15.98965	9.430437	
Iran, Islamic Re	Deaths	Male	2011	11.80487	15.88081	9.483324	
Iran, Islamic Re	Deaths	Male	2012	11.78787	15.62309	9.433201	
Iran, Islamic Re	Deaths	Male	2013	12.07959	15.91677	9.763927	
Iran, Islamic Re	Deaths	Male	2014	12.19012	15.96298	9.918241	
Iran, Islamic Re	Deaths	Male	2015	12.53261	16.19632	10.16962	
Iran, Islamic Re	Deaths	Male	2016	12.90281	16.74989	10.53553	
Iran, Islamic Re	Deaths	Male	2017	13.19466	17.05755	10.73134	
Iran, Islamic Re	Deaths	Male	2018	13.49516	17.51382	11.11283	
Iran, Islamic Re	Deaths	Male	2019	13.79818	17.49669	11.49508	
Iran, Islamic Re	Deaths	Male	2020	13.85689	17.88463	11.8229	
Iran, Islamic Re	Deaths	Male	2021	13.14458	16.90383	11.18457	
Iran, Islamic Re	Deaths	Male	2022	13.46244	16.95288	11.041	
Iran, Islamic Re	Deaths	Male	2023	13.78286	17.37155	11.07802	
Iran, Islamic Re	Deaths	Female	2010	5.017217	6.732382	3.648765	
Iran, Islamic Re	Deaths	Female	2011	5.178534	6.947041	3.769967	
Iran, Islamic Re	Deaths	Female	2012	5.17747	6.953157	3.785636	
Iran, Islamic Re	Deaths	Female	2013	5.350783	7.134296	3.967986	
Iran, Islamic Re	Deaths	Female	2014	5.423957	7.233577	4.048824	
Iran, Islamic Re	Deaths	Female	2015	5.609537	7.519014	4.137856	
Iran, Islamic Re	Deaths	Female	2016	5.882569	7.836726	4.419214	
Iran, Islamic Re	Deaths	Female	2017	6.059141	8.169458	4.624073	
Iran, Islamic Re	Deaths	Female	2018	6.211555	8.475355	4.655618	
Iran, Islamic Re	Deaths	Female	2019	6.452976	8.800174	4.813271	
Iran, Islamic Re	Deaths	Female	2020	6.41327	8.493416	4.888595	
Iran, Islamic Re	Deaths	Female	2021	6.125071	8.143703	4.622099	
Iran, Islamic Re	Deaths	Female	2022	6.187198	8.467531	4.558135	
Iran, Islamic Re	Deaths	Female	2023	6.422081	8.792958	4.72139	
Iran, Islamic Re	DALYs	Male	2010	296.4307	409.6652	237.525	
Iran, Islamic Re	DALYs	Male	2011	301.1363	406.1515	239.7354	
Iran, Islamic Re	DALYs	Male	2012	303.1594	402.0604	246.1495	
Iran, Islamic Re	DALYs	Male	2013	309.1408	405.4351	250.9808	
Iran, Islamic Re	DALYs	Male	2014	313.6326	405.4083	256.0174	

Iran, Islamic Re	DALYs	Male	2015	322.6834	419.9347	262.5568	
Iran, Islamic Re	DALYs	Male	2016	331.4559	431.3913	269.9889	
Iran, Islamic Re	DALYs	Male	2017	338.8567	440.2415	276.0705	
Iran, Islamic Re	DALYs	Male	2018	347.3346	451.0758	283.9161	
Iran, Islamic Re	DALYs	Male	2019	354.4369	458.3254	292.5797	
Iran, Islamic Re	DALYs	Male	2020	361.912	467.5273	303.0134	
Iran, Islamic Re	DALYs	Male	2021	346.2323	448.615	289.6962	
Iran, Islamic Re	DALYs	Male	2022	354.8937	454.0869	291.6812	
Iran, Islamic Re	DALYs	Male	2023	363.0584	459.0041	290.8926	
Iran, Islamic Re	DALYs	Female	2010	129.9342	172.9678	93.98467	
Iran, Islamic Re	DALYs	Female	2011	133.2977	179.2819	96.10875	
Iran, Islamic Re	DALYs	Female	2012	134.2666	178.9909	97.7099	
Iran, Islamic Re	DALYs	Female	2013	137.4277	182.6217	102.3647	
Iran, Islamic Re	DALYs	Female	2014	139.9172	185.4467	103.0297	
Iran, Islamic Re	DALYs	Female	2015	144.7827	192.9828	106.127	
Iran, Islamic Re	DALYs	Female	2016	150.8998	202.2468	111.5132	
Iran, Islamic Re	DALYs	Female	2017	154.9638	210.9239	116.0282	
Iran, Islamic Re	DALYs	Female	2018	158.6888	218.5648	119.2218	
Iran, Islamic Re	DALYs	Female	2019	164.2437	224.0573	122.2612	
Iran, Islamic Re	DALYs	Female	2020	165.2415	222.1241	124.5462	
Iran, Islamic Re	DALYs	Female	2021	157.6801	213.0201	118.2419	
Iran, Islamic Re	DALYs	Female	2022	158.6072	217.2521	118.1611	
Iran, Islamic Re	DALYs	Female	2023	164.9745	227.7329	119.4129	
Iraq	Incidence	Male	2010	14.78717	18.84907	12.03829	
Iraq	Incidence	Male	2011	15.37748	19.25681	12.32841	
Iraq	Incidence	Male	2012	15.92997	20.18178	12.79958	
Iraq	Incidence	Male	2013	16.11044	20.23103	12.85827	
Iraq	Incidence	Male	2014	16.26735	20.49247	12.88445	
Iraq	Incidence	Male	2015	16.79726	21.15133	13.05566	
Iraq	Incidence	Male	2016	17.14909	21.57957	13.37699	
Iraq	Incidence	Male	2017	17.57184	22.29856	13.6533	
Iraq	Incidence	Male	2018	18.37801	23.66055	14.22375	
Iraq	Incidence	Male	2019	19.18036	25.13725	14.57179	
Iraq	Incidence	Male	2020	20.90266	27.21176	16.16801	
Iraq	Incidence	Male	2021	21.26803	27.77534	16.26241	
Iraq	Incidence	Male	2022	22.48274	29.32083	16.279	

Iraq	Incidence	Male	2023	23.63048	31.48759	16.72063	
Iraq	Incidence	Female	2010	5.395778	6.830223	4.241236	
Iraq	Incidence	Female	2011	5.668095	7.058802	4.471376	
Iraq	Incidence	Female	2012	5.946676	7.465638	4.756778	
Iraq	Incidence	Female	2013	6.069861	7.597993	4.917242	
Iraq	Incidence	Female	2014	6.187136	7.627945	5.087758	
Iraq	Incidence	Female	2015	6.322633	7.920187	5.171978	
Iraq	Incidence	Female	2016	6.482442	8.291402	5.067124	
Iraq	Incidence	Female	2017	6.667625	8.586593	5.034306	
Iraq	Incidence	Female	2018	6.962917	9.067932	5.055511	
Iraq	Incidence	Female	2019	7.396175	9.551758	5.356659	
Iraq	Incidence	Female	2020	7.673836	10.04985	5.541707	
Iraq	Incidence	Female	2021	8.160316	10.81434	6.000802	
Iraq	Incidence	Female	2022	8.780938	11.94567	6.312901	
Iraq	Incidence	Female	2023	9.206621	12.56011	6.554788	
Iraq	Deaths	Male	2010	14.28639	18.34783	11.66583	
Iraq	Deaths	Male	2011	14.84536	18.63646	11.89519	
Iraq	Deaths	Male	2012	15.36545	19.39805	12.32847	
Iraq	Deaths	Male	2013	15.53731	19.61541	12.38841	
Iraq	Deaths	Male	2014	15.68384	19.84148	12.36975	
Iraq	Deaths	Male	2015	16.16931	20.4255	12.59497	
Iraq	Deaths	Male	2016	16.4745	20.75346	12.88691	
Iraq	Deaths	Male	2017	16.85851	21.49526	12.96491	
Iraq	Deaths	Male	2018	17.6129	22.74342	13.45552	
Iraq	Deaths	Male	2019	18.34645	24.08498	13.84644	
Iraq	Deaths	Male	2020	19.87471	25.93257	15.23313	
Iraq	Deaths	Male	2021	20.11487	26.37943	15.23688	
Iraq	Deaths	Male	2022	21.25936	27.73199	15.54354	
Iraq	Deaths	Male	2023	22.30261	29.79979	15.75267	
Iraq	Deaths	Female	2010	5.038848	6.399889	3.949802	
Iraq	Deaths	Female	2011	5.291401	6.64281	4.169168	
Iraq	Deaths	Female	2012	5.546976	6.993212	4.386586	
Iraq	Deaths	Female	2013	5.65982	7.090206	4.529364	
Iraq	Deaths	Female	2014	5.766168	7.183889	4.712491	
Iraq	Deaths	Female	2015	5.879078	7.339971	4.805741	
Iraq	Deaths	Female	2016	6.011762	7.662646	4.655079	

Iraq	Deaths	Female	2017	6.172301	7.917091	4.639855	
Iraq	Deaths	Female	2018	6.435644	8.353076	4.627456	
Iraq	Deaths	Female	2019	6.819082	8.8006	4.877879	
Iraq	Deaths	Female	2020	7.009645	9.190025	5.043272	
Iraq	Deaths	Female	2021	7.40966	9.906108	5.360491	
Iraq	Deaths	Female	2022	7.977922	10.85902	5.726285	
Iraq	Deaths	Female	2023	8.350314	11.5138	5.943545	
Iraq	DALYs	Male	2010	409.4711	519.3573	330.9281	
Iraq	DALYs	Male	2011	423.37	529.7537	337.68	
Iraq	DALYs	Male	2012	436.092	546.2191	347.5524	
Iraq	DALYs	Male	2013	439.028	552.2801	352.3192	
Iraq	DALYs	Male	2014	441.9272	558.5548	349.5556	
Iraq	DALYs	Male	2015	455.8864	574.6416	355.6465	
Iraq	DALYs	Male	2016	464.7894	585.9084	362.4383	
Iraq	DALYs	Male	2017	475.2596	608.71	369.6298	
Iraq	DALYs	Male	2018	496.5256	642.5465	385.4307	
Iraq	DALYs	Male	2019	516.8604	667.6547	401.9072	
Iraq	DALYs	Male	2020	564.6146	737.6028	440.7014	
Iraq	DALYs	Male	2021	572.2695	749.5237	440.0604	
Iraq	DALYs	Male	2022	598.1211	786.4181	442.915	
Iraq	DALYs	Male	2023	626.5323	830.8707	454.7756	
Iraq	DALYs	Female	2010	160.3271	202.2001	127.3583	
Iraq	DALYs	Female	2011	167.0115	207.7239	133.9909	
Iraq	DALYs	Female	2012	173.8429	216.8225	142.2239	
Iraq	DALYs	Female	2013	176.2384	220.1307	143.59	
Iraq	DALYs	Female	2014	178.8493	219.8102	149.74	
Iraq	DALYs	Female	2015	182.4542	226.7956	151.3612	
Iraq	DALYs	Female	2016	186.6205	233.7758	146.6918	
Iraq	DALYs	Female	2017	191.4323	241.0733	147.3257	
Iraq	DALYs	Female	2018	199.4803	257.9223	147.991	
Iraq	DALYs	Female	2019	211.0268	270.6923	155.9479	
Iraq	DALYs	Female	2020	219.6938	287.2973	164.6241	
Iraq	DALYs	Female	2021	231.8208	305.379	175.5022	
Iraq	DALYs	Female	2022	245.6256	325.0392	183.8284	
Iraq	DALYs	Female	2023	256.1184	344.4763	189.8671	
Ireland	Incidence	Male	2010	51.61444	56.3407	47.85397	

Ireland	Incidence	Male	2011	54.07294	58.43137	49.51553	
Ireland	Incidence	Male	2012	55.05596	59.76748	50.07568	
Ireland	Incidence	Male	2013	55.98234	61.07214	50.77748	
Ireland	Incidence	Male	2014	55.80763	60.70573	50.724	
Ireland	Incidence	Male	2015	53.12406	57.55401	48.15925	
Ireland	Incidence	Male	2016	53.34852	58.12968	48.03088	
Ireland	Incidence	Male	2017	53.41847	58.37195	47.95965	
Ireland	Incidence	Male	2018	53.38221	58.6031	48.04607	
Ireland	Incidence	Male	2019	54.94831	60.185	49.33782	
Ireland	Incidence	Male	2020	54.49618	59.79991	48.51007	
Ireland	Incidence	Male	2021	52.60668	58.32316	47.06702	
Ireland	Incidence	Male	2022	58.3685	64.9784	51.83649	
Ireland	Incidence	Male	2023	59.96266	68.01153	53.21413	
Ireland	Incidence	Female	2010	38.0688	42.75342	34.14111	
Ireland	Incidence	Female	2011	39.56082	44.45524	35.41858	
Ireland	Incidence	Female	2012	40.3089	44.82939	35.75753	
Ireland	Incidence	Female	2013	41.81213	46.64889	36.77596	
Ireland	Incidence	Female	2014	44.25091	49.1213	38.9951	
Ireland	Incidence	Female	2015	43.5222	48.86707	38.43373	
Ireland	Incidence	Female	2016	45.18249	50.65549	40.01083	
Ireland	Incidence	Female	2017	45.67003	51.15518	39.88678	
Ireland	Incidence	Female	2018	43.91504	49.5284	38.36351	
Ireland	Incidence	Female	2019	44.75657	50.80133	38.31286	
Ireland	Incidence	Female	2020	45.95296	51.87311	39.77427	
Ireland	Incidence	Female	2021	44.4386	50.55343	38.1912	
Ireland	Incidence	Female	2022	50.39612	57.51052	43.1935	
Ireland	Incidence	Female	2023	53.41576	61.77005	45.57608	
Ireland	Deaths	Male	2010	46.41283	49.82169	43.33065	
Ireland	Deaths	Male	2011	48.45242	51.91621	45.38632	
Ireland	Deaths	Male	2012	49.0304	52.27211	45.6492	
Ireland	Deaths	Male	2013	49.54455	52.943	45.98063	
Ireland	Deaths	Male	2014	49.08213	52.3009	45.73826	
Ireland	Deaths	Male	2015	46.59427	49.59455	43.64574	
Ireland	Deaths	Male	2016	46.61971	49.87858	43.40943	
Ireland	Deaths	Male	2017	46.44201	49.52527	43.28465	
Ireland	Deaths	Male	2018	46.24103	49.54498	43.07045	

Ireland	Deaths	Male	2019	47.53852	51.10895	44.12579	
Ireland	Deaths	Male	2020	46.77284	50.2138	43.83963	
Ireland	Deaths	Male	2021	45.02123	48.93209	41.49926	
Ireland	Deaths	Male	2022	49.96171	55.15965	45.87623	
Ireland	Deaths	Male	2023	51.59438	58.40789	46.68541	
Ireland	Deaths	Female	2010	33.22026	36.00531	29.80019	
Ireland	Deaths	Female	2011	34.33092	37.24211	31.03521	
Ireland	Deaths	Female	2012	34.68214	37.73607	31.32445	
Ireland	Deaths	Female	2013	35.68039	38.967	32.13958	
Ireland	Deaths	Female	2014	37.42249	40.60292	33.64094	
Ireland	Deaths	Female	2015	36.59824	39.4731	33.29274	
Ireland	Deaths	Female	2016	37.8207	40.90425	34.44911	
Ireland	Deaths	Female	2017	38.10564	41.35722	34.42466	
Ireland	Deaths	Female	2018	36.37389	39.39197	32.31734	
Ireland	Deaths	Female	2019	36.71156	40.0134	32.46809	
Ireland	Deaths	Female	2020	37.28843	40.74927	33.44529	
Ireland	Deaths	Female	2021	35.86199	39.2223	32.13588	
Ireland	Deaths	Female	2022	40.7399	45.02784	35.95464	
Ireland	Deaths	Female	2023	43.50325	48.48841	38.04191	
Ireland	DALYs	Male	2010	1034.702	1106.641	976.7422	
Ireland	DALYs	Male	2011	1069.458	1141.31	1005.622	
Ireland	DALYs	Male	2012	1075.154	1143.372	1005.62	
Ireland	DALYs	Male	2013	1080.207	1150.868	1013.073	
Ireland	DALYs	Male	2014	1066.107	1135.411	998.285	
Ireland	DALYs	Male	2015	1000.299	1064.211	937.0321	
Ireland	DALYs	Male	2016	993.574	1064.116	930.7899	
Ireland	DALYs	Male	2017	989.68	1054.952	924.2657	
Ireland	DALYs	Male	2018	981.4808	1052.559	917.1774	
Ireland	DALYs	Male	2019	1001.037	1072.346	929.1674	
Ireland	DALYs	Male	2020	983.915	1057.814	916.1214	
Ireland	DALYs	Male	2021	939.8054	1022.964	870.8617	
Ireland	DALYs	Male	2022	1039.132	1151.779	956.4492	
Ireland	DALYs	Male	2023	1059.832	1197.857	961.5028	
Ireland	DALYs	Female	2010	724.5542	775.2692	667.7032	
Ireland	DALYs	Female	2011	743.9693	795.8212	684.5972	
Ireland	DALYs	Female	2012	746.6436	802.7118	683.646	

Ireland	DALYs	Female	2013	764.4455	825.3805	702.5679	
Ireland	DALYs	Female	2014	800.5633	859.6734	737.4135	
Ireland	DALYs	Female	2015	776.386	830.6034	718.2139	
Ireland	DALYs	Female	2016	796.4588	854.3764	735.455	
Ireland	DALYs	Female	2017	794.1283	853.4176	728.0937	
Ireland	DALYs	Female	2018	761.7938	817.2279	698.6915	
Ireland	DALYs	Female	2019	775.5022	838.1094	705.3439	
Ireland	DALYs	Female	2020	790.7452	864.4062	722.4601	
Ireland	DALYs	Female	2021	758.5852	827.3471	687.7755	
Ireland	DALYs	Female	2022	854.0408	940.1488	769.8556	
Ireland	DALYs	Female	2023	896.9652	994.346	799.7744	
Israel	Incidence	Male	2010	38.49192	41.95666	35.29634	
Israel	Incidence	Male	2011	37.97138	41.1991	34.70729	
Israel	Incidence	Male	2012	37.38376	41.0026	34.1622	
Israel	Incidence	Male	2013	38.14418	41.63593	34.66355	
Israel	Incidence	Male	2014	38.27448	41.8307	35.12256	
Israel	Incidence	Male	2015	38.39187	41.67177	35.17312	
Israel	Incidence	Male	2016	36.76301	39.81616	33.92487	
Israel	Incidence	Male	2017	36.86922	39.87598	34.09641	
Israel	Incidence	Male	2018	36.47748	39.75723	33.49106	
Israel	Incidence	Male	2019	35.78573	38.83081	32.56545	
Israel	Incidence	Male	2020	35.49466	38.72442	32.38739	
Israel	Incidence	Male	2021	35.29184	38.28657	32.10121	
Israel	Incidence	Male	2022	36.61901	39.94886	32.83927	
Israel	Incidence	Male	2023	38.10405	42.71402	32.76486	
Israel	Incidence	Female	2010	18.08122	20.1909	16.02235	
Israel	Incidence	Female	2011	18.81966	21.08803	16.63551	
Israel	Incidence	Female	2012	18.52539	20.68976	16.5385	
Israel	Incidence	Female	2013	18.4925	20.59008	16.42278	
Israel	Incidence	Female	2014	19.04259	21.14191	16.8631	
Israel	Incidence	Female	2015	18.8319	20.86914	16.62398	
Israel	Incidence	Female	2016	18.6152	20.61702	16.47197	
Israel	Incidence	Female	2017	18.36018	20.20386	15.93551	
Israel	Incidence	Female	2018	18.92378	21.00174	16.39523	
Israel	Incidence	Female	2019	19.19336	21.56131	16.67814	
Israel	Incidence	Female	2020	18.87615	21.37109	16.40276	

Israel	Incidence	Female	2021	19.38564	22.075	16.72253	
Israel	Incidence	Female	2022	19.6572	22.50593	16.84858	
Israel	Incidence	Female	2023	19.45837	22.44427	16.31409	
Israel	Deaths	Male	2010	35.74422	38.21446	33.10861	
Israel	Deaths	Male	2011	35.32377	37.67685	32.48706	
Israel	Deaths	Male	2012	34.7027	37.41718	31.97736	
Israel	Deaths	Male	2013	35.28105	37.70323	32.60899	
Israel	Deaths	Male	2014	35.34958	37.83302	32.80259	
Israel	Deaths	Male	2015	35.37803	37.69545	33.11469	
Israel	Deaths	Male	2016	33.93901	35.97653	31.89892	
Israel	Deaths	Male	2017	34.25912	36.37138	32.14024	
Israel	Deaths	Male	2018	33.75082	35.86493	31.68394	
Israel	Deaths	Male	2019	33.08454	35.10718	30.87403	
Israel	Deaths	Male	2020	32.70066	34.66438	30.80899	
Israel	Deaths	Male	2021	32.33024	34.32449	30.08365	
Israel	Deaths	Male	2022	33.54491	35.9402	30.78891	
Israel	Deaths	Male	2023	34.84937	38.90919	30.46028	
Israel	Deaths	Female	2010	16.79619	18.37666	14.81224	
Israel	Deaths	Female	2011	17.46996	19.28074	15.4995	
Israel	Deaths	Female	2012	17.16784	18.84396	15.33999	
Israel	Deaths	Female	2013	17.14166	18.75031	15.15114	
Israel	Deaths	Female	2014	17.64086	19.20805	15.65842	
Israel	Deaths	Female	2015	17.49517	19.0452	15.46926	
Israel	Deaths	Female	2016	17.31982	18.89982	15.26158	
Israel	Deaths	Female	2017	17.10602	18.62684	15.13468	
Israel	Deaths	Female	2018	17.59881	19.25829	15.3329	
Israel	Deaths	Female	2019	17.74249	19.49567	15.49383	
Israel	Deaths	Female	2020	17.3005	19.12177	15.10625	
Israel	Deaths	Female	2021	17.65427	19.65046	15.32615	
Israel	Deaths	Female	2022	17.86315	20.19276	15.47685	
Israel	Deaths	Female	2023	17.64358	20.12921	14.88271	
Israel	DALYs	Male	2010	834.805	890.7413	779.5088	
Israel	DALYs	Male	2011	813.3543	865.6032	756.926	
Israel	DALYs	Male	2012	797.3916	850.6398	740.9299	
Israel	DALYs	Male	2013	808.7188	863.1191	757.614	
Israel	DALYs	Male	2014	806.2601	865.0844	754.4934	

Israel	DALYs	Male	2015	805.5059	859.9798	752.2704	
Israel	DALYs	Male	2016	766.6924	815.355	721.5699	
Israel	DALYs	Male	2017	759.1882	808.5919	711.9653	
Israel	DALYs	Male	2018	749.0568	798.9176	705.6266	
Israel	DALYs	Male	2019	725.9093	770.9556	680.5125	
Israel	DALYs	Male	2020	710.88	755.8887	670.7914	
Israel	DALYs	Male	2021	702.3396	748.3241	656.4168	
Israel	DALYs	Male	2022	722.2437	778.2598	668.8783	
Israel	DALYs	Male	2023	747.6408	831.3524	659.0746	
Israel	DALYs	Female	2010	362.9166	392.9481	327.2256	
Israel	DALYs	Female	2011	376.1567	411.051	342.3582	
Israel	DALYs	Female	2012	367.2802	400.9852	335.9089	
Israel	DALYs	Female	2013	361.9007	393.0662	329.5073	
Israel	DALYs	Female	2014	370.1708	399.8408	336.5781	
Israel	DALYs	Female	2015	363.4154	392.9546	329.8389	
Israel	DALYs	Female	2016	356.5039	390.1772	324.2356	
Israel	DALYs	Female	2017	348.2124	378.6054	315.0015	
Israel	DALYs	Female	2018	356.6459	389.836	319.1645	
Israel	DALYs	Female	2019	359.5609	392.8945	322.7784	
Israel	DALYs	Female	2020	353.1151	389.1215	319.031	
Israel	DALYs	Female	2021	358.2746	397.5813	319.3843	
Israel	DALYs	Female	2022	362.1607	407.0685	323.5479	
Israel	DALYs	Female	2023	357.6158	404.3546	313.7624	
Italy	Incidence	Male	2010	104.574	112.3608	96.61009	
Italy	Incidence	Male	2011	105.4753	113.3564	96.86617	
Italy	Incidence	Male	2012	103.8993	112.4923	96.32381	
Italy	Incidence	Male	2013	101.6339	110.3962	93.54055	
Italy	Incidence	Male	2014	100.2209	109.7258	91.28481	
Italy	Incidence	Male	2015	100.5941	110.169	91.61996	
Italy	Incidence	Male	2016	95.63926	104.5205	86.81514	
Italy	Incidence	Male	2017	97.0743	105.7854	88.07956	
Italy	Incidence	Male	2018	94.675	103.0595	85.24178	
Italy	Incidence	Male	2019	92.46716	100.7338	83.33884	
Italy	Incidence	Male	2020	92.89733	100.4045	84.30713	
Italy	Incidence	Male	2021	89.45987	97.92071	80.65134	
Italy	Incidence	Male	2022	91.92912	101.3267	82.70039	

Italy	Incidence	Male	2023	89.99701	99.4336	81.2021	
Italy	Incidence	Female	2010	35.77123	40.21878	31.17382	
Italy	Incidence	Female	2011	36.86237	41.89226	31.91889	
Italy	Incidence	Female	2012	38.11236	43.29602	32.75073	
Italy	Incidence	Female	2013	38.35758	43.48806	32.73432	
Italy	Incidence	Female	2014	39.47591	44.82822	33.37389	
Italy	Incidence	Female	2015	41.23562	47.09361	34.97857	
Italy	Incidence	Female	2016	40.57474	46.38411	34.37265	
Italy	Incidence	Female	2017	42.35883	48.81537	35.61418	
Italy	Incidence	Female	2018	42.3996	48.72889	35.29283	
Italy	Incidence	Female	2019	42.87094	49.5478	35.77806	
Italy	Incidence	Female	2020	43.77314	50.03415	37.36084	
Italy	Incidence	Female	2021	44.23329	50.36961	37.47845	
Italy	Incidence	Female	2022	45.78248	52.28128	38.42013	
Italy	Incidence	Female	2023	44.99861	51.54216	37.37358	
Italy	Deaths	Male	2010	94.91489	99.52018	88.54674	
Italy	Deaths	Male	2011	96.05119	100.8394	89.6424	
Italy	Deaths	Male	2012	95.06139	100.1824	88.04833	
Italy	Deaths	Male	2013	93.24475	98.12991	86.55349	
Italy	Deaths	Male	2014	92.19514	97.45538	85.68517	
Italy	Deaths	Male	2015	93.1112	98.59755	86.12856	
Italy	Deaths	Male	2016	88.85579	94.54878	82.16963	
Italy	Deaths	Male	2017	90.63644	96.67357	83.51994	
Italy	Deaths	Male	2018	88.4982	94.72925	81.65637	
Italy	Deaths	Male	2019	86.57746	93.1217	79.83804	
Italy	Deaths	Male	2020	86.99003	93.07367	80.79171	
Italy	Deaths	Male	2021	83.75049	90.01985	77.05013	
Italy	Deaths	Male	2022	86.35177	93.48761	79.36155	
Italy	Deaths	Male	2023	84.40139	91.37939	77.3008	
Italy	Deaths	Female	2010	29.8956	32.86141	25.62894	
Italy	Deaths	Female	2011	30.79695	33.85442	26.52146	
Italy	Deaths	Female	2012	31.91697	35.19423	27.31458	
Italy	Deaths	Female	2013	32.11384	35.272	27.21463	
Italy	Deaths	Female	2014	33.19405	36.46989	28.0056	
Italy	Deaths	Female	2015	35.01198	38.74577	29.70086	
Italy	Deaths	Female	2016	34.49728	38.2592	29.22732	

Italy	Deaths	Female	2017	36.17645	40.14562	30.72844	
Italy	Deaths	Female	2018	36.28619	40.45937	30.87609	
Italy	Deaths	Female	2019	36.69715	41.1275	31.35017	
Italy	Deaths	Female	2020	37.47862	41.7535	32.42489	
Italy	Deaths	Female	2021	37.82707	42.10001	32.29438	
Italy	Deaths	Female	2022	39.26976	43.71414	33.3762	
Italy	Deaths	Female	2023	38.43812	43.13539	32.1838	
Italy	DALYs	Male	2010	1933.479	2026.351	1831.241	
Italy	DALYs	Male	2011	1946.891	2045.426	1839.635	
Italy	DALYs	Male	2012	1908.003	2000.081	1798.775	
Italy	DALYs	Male	2013	1862.417	1955.922	1761.699	
Italy	DALYs	Male	2014	1837.401	1935.017	1726.65	
Italy	DALYs	Male	2015	1836.777	1939.032	1732.227	
Italy	DALYs	Male	2016	1744.584	1844.153	1634.352	
Italy	DALYs	Male	2017	1761.274	1859.646	1653.626	
Italy	DALYs	Male	2018	1714.337	1818.798	1607.954	
Italy	DALYs	Male	2019	1666.325	1782.838	1563.45	
Italy	DALYs	Male	2020	1670.104	1785.958	1571.4	
Italy	DALYs	Male	2021	1610.001	1736.419	1500.199	
Italy	DALYs	Male	2022	1641.916	1783.805	1529.261	
Italy	DALYs	Male	2023	1609.186	1750.659	1488.442	
Italy	DALYs	Female	2010	611.5306	661.8511	546.9626	
Italy	DALYs	Female	2011	631.7774	683.9833	563.9071	
Italy	DALYs	Female	2012	651.1322	707.0654	583.8554	
Italy	DALYs	Female	2013	655.5561	709.342	581.1997	
Italy	DALYs	Female	2014	672.8709	727.8406	593.1943	
Italy	DALYs	Female	2015	699.181	762.5721	614.903	
Italy	DALYs	Female	2016	689.9707	753.6621	611.8595	
Italy	DALYs	Female	2017	717.0964	786.6873	634.7914	
Italy	DALYs	Female	2018	714.9274	789.732	632.4513	
Italy	DALYs	Female	2019	718.0435	793.6924	635.6656	
Italy	DALYs	Female	2020	729.6764	801.4509	652.0042	
Italy	DALYs	Female	2021	734.3159	808.7328	651.0709	
Italy	DALYs	Female	2022	753.3554	831.1791	658.1028	
Italy	DALYs	Female	2023	741.9619	820.4379	644.1481	
Jamaica	Incidence	Male	2010	23.85471	26.28312	21.37061	

Jamaica	Incidence	Male	2011	23.53766	26.20974	20.97102	
Jamaica	Incidence	Male	2012	25.47688	28.32259	22.74204	
Jamaica	Incidence	Male	2013	25.78113	29.11581	22.99795	
Jamaica	Incidence	Male	2014	25.84029	29.44836	23.01368	
Jamaica	Incidence	Male	2015	27.59086	31.88499	24.14489	
Jamaica	Incidence	Male	2016	28.70473	33.193	25.3316	
Jamaica	Incidence	Male	2017	29.86187	34.3455	26.38515	
Jamaica	Incidence	Male	2018	30.53892	35.24711	26.87833	
Jamaica	Incidence	Male	2019	30.91376	35.44949	26.56368	
Jamaica	Incidence	Male	2020	31.9269	36.53817	27.10299	
Jamaica	Incidence	Male	2021	33.49188	38.40554	28.73812	
Jamaica	Incidence	Male	2022	32.73376	38.7902	27.36222	
Jamaica	Incidence	Male	2023	31.04764	37.30238	25.38619	
Jamaica	Incidence	Female	2010	7.292093	8.470375	6.309623	
Jamaica	Incidence	Female	2011	7.795966	9.013241	6.636505	
Jamaica	Incidence	Female	2012	7.707835	9.137639	6.472251	
Jamaica	Incidence	Female	2013	8.321694	9.747921	6.90144	
Jamaica	Incidence	Female	2014	8.522618	9.958533	7.199057	
Jamaica	Incidence	Female	2015	8.715676	10.11045	7.308396	
Jamaica	Incidence	Female	2016	8.922136	10.25433	7.566718	
Jamaica	Incidence	Female	2017	9.07156	10.51294	7.575302	
Jamaica	Incidence	Female	2018	9.246947	10.79613	7.677281	
Jamaica	Incidence	Female	2019	9.472487	11.11099	7.844062	
Jamaica	Incidence	Female	2020	9.676402	11.46025	7.867639	
Jamaica	Incidence	Female	2021	10.20306	12.20945	8.274583	
Jamaica	Incidence	Female	2022	10.21072	12.24784	8.149801	
Jamaica	Incidence	Female	2023	10.66255	12.96605	8.556252	
Jamaica	Deaths	Male	2010	23.65073	25.96384	21.17698	
Jamaica	Deaths	Male	2011	23.33304	25.91503	20.94283	
Jamaica	Deaths	Male	2012	25.07832	27.89873	22.48346	
Jamaica	Deaths	Male	2013	25.37907	28.69624	22.71236	
Jamaica	Deaths	Male	2014	25.42067	29.11885	22.50006	
Jamaica	Deaths	Male	2015	27.01708	31.13611	23.92426	
Jamaica	Deaths	Male	2016	28.00445	32.45994	24.66227	
Jamaica	Deaths	Male	2017	29.05677	33.30956	25.84391	
Jamaica	Deaths	Male	2018	29.6685	33.74832	26.25227	

Jamaica	Deaths	Male	2019	29.9807	34.12999	26.06388	
Jamaica	Deaths	Male	2020	30.91589	35.23037	26.43193	
Jamaica	Deaths	Male	2021	32.4044	37.05611	27.8078	
Jamaica	Deaths	Male	2022	31.70125	37.13671	26.46354	
Jamaica	Deaths	Male	2023	30.1021	35.91516	24.79861	
Jamaica	Deaths	Female	2010	7.147148	8.220217	6.164858	
Jamaica	Deaths	Female	2011	7.607983	8.747208	6.466677	
Jamaica	Deaths	Female	2012	7.517459	8.753171	6.340232	
Jamaica	Deaths	Female	2013	8.107991	9.346045	6.785276	
Jamaica	Deaths	Female	2014	8.352576	9.683186	7.118154	
Jamaica	Deaths	Female	2015	8.493666	9.850397	7.204531	
Jamaica	Deaths	Female	2016	8.684745	9.903622	7.425771	
Jamaica	Deaths	Female	2017	8.792993	10.17818	7.501107	
Jamaica	Deaths	Female	2018	8.93492	10.41273	7.534969	
Jamaica	Deaths	Female	2019	9.113724	10.67113	7.469358	
Jamaica	Deaths	Female	2020	9.279969	10.98484	7.609576	
Jamaica	Deaths	Female	2021	9.721379	11.57671	7.904601	
Jamaica	Deaths	Female	2022	9.760677	11.67819	7.853885	
Jamaica	Deaths	Female	2023	10.22576	12.45542	8.168899	
Jamaica	DALYs	Male	2010	572.825	633.8822	508.3594	
Jamaica	DALYs	Male	2011	562.6048	625.9626	500.7339	
Jamaica	DALYs	Male	2012	616.1643	692.9874	551.6798	
Jamaica	DALYs	Male	2013	624.8934	703.1063	557.4961	
Jamaica	DALYs	Male	2014	631.9952	720.3109	558.2884	
Jamaica	DALYs	Male	2015	683.015	785.0957	601.5166	
Jamaica	DALYs	Male	2016	716.9644	828.779	630.2604	
Jamaica	DALYs	Male	2017	749.8261	857.3282	662.9127	
Jamaica	DALYs	Male	2018	767.6209	870.7507	675.4576	
Jamaica	DALYs	Male	2019	775.2231	890.8645	667.6986	
Jamaica	DALYs	Male	2020	798.2637	913.3371	685.089	
Jamaica	DALYs	Male	2021	833.7478	961.4063	711.6557	
Jamaica	DALYs	Male	2022	809.2768	943.8691	681.7748	
Jamaica	DALYs	Male	2023	761.8337	913.2307	623.8279	
Jamaica	DALYs	Female	2010	169.7986	197.6622	145.5748	
Jamaica	DALYs	Female	2011	182.9181	212.1651	156.6344	
Jamaica	DALYs	Female	2012	181.2993	213.2241	152.5919	

Jamaica	DALYs	Female	2013	195.4657	225.8233	163.5919	
Jamaica	DALYs	Female	2014	200.8597	232.3365	170.8778	
Jamaica	DALYs	Female	2015	207.6528	239.6339	176.0316	
Jamaica	DALYs	Female	2016	213.4956	244.6262	181.4306	
Jamaica	DALYs	Female	2017	218.2238	251.3963	184.177	
Jamaica	DALYs	Female	2018	222.6587	261.4081	186.0857	
Jamaica	DALYs	Female	2019	228.1581	271.4228	186.329	
Jamaica	DALYs	Female	2020	233.1776	277.752	190.0281	
Jamaica	DALYs	Female	2021	247.9053	298.274	199.9698	
Jamaica	DALYs	Female	2022	243.6407	290.4345	194.2073	
Jamaica	DALYs	Female	2023	250.4212	308.5638	200.2429	
Japan	Incidence	Male	2010	122.7426	130.7652	113.4175	
Japan	Incidence	Male	2011	127.1966	135.3469	117.7948	
Japan	Incidence	Male	2012	129.8929	138.886	120.3967	
Japan	Incidence	Male	2013	132.6003	141.6641	122.3131	
Japan	Incidence	Male	2014	135.5911	145.0927	124.5467	
Japan	Incidence	Male	2015	133.8445	142.994	123.3048	
Japan	Incidence	Male	2016	134.1317	143.2627	122.6714	
Japan	Incidence	Male	2017	136.9792	147.0251	124.1866	
Japan	Incidence	Male	2018	137.2996	148.0869	123.5674	
Japan	Incidence	Male	2019	139.6907	151.8775	126.0296	
Japan	Incidence	Male	2020	139.4291	152.0583	125.4827	
Japan	Incidence	Male	2021	141.6977	154.2909	127.9163	
Japan	Incidence	Male	2022	147.6694	159.393	133.5894	
Japan	Incidence	Male	2023	153.2111	166.1996	136.6705	
Japan	Incidence	Female	2010	45.79372	51.47874	37.88096	
Japan	Incidence	Female	2011	47.1354	53.64593	38.72277	
Japan	Incidence	Female	2012	48.80946	55.71585	39.93212	
Japan	Incidence	Female	2013	50.31094	57.46977	40.75095	
Japan	Incidence	Female	2014	51.28038	58.50511	41.00681	
Japan	Incidence	Female	2015	51.70044	59.57204	40.95784	
Japan	Incidence	Female	2016	52.28139	60.27655	40.82381	
Japan	Incidence	Female	2017	53.21699	61.99698	40.92398	
Japan	Incidence	Female	2018	55.01293	64.29938	42.00178	
Japan	Incidence	Female	2019	56.33746	65.90922	43.10411	
Japan	Incidence	Female	2020	56.67969	66.29847	43.59396	

Japan	Incidence	Female	2021	58.61329	68.43149	45.27424	
Japan	Incidence	Female	2022	61.80091	71.67953	48.15489	
Japan	Incidence	Female	2023	65.46142	77.46705	49.42589	
Japan	Deaths	Male	2010	88.65767	92.82909	82.57253	
Japan	Deaths	Male	2011	91.62266	96.12385	85.42334	
Japan	Deaths	Male	2012	93.36373	97.76203	86.82727	
Japan	Deaths	Male	2013	95.04455	99.44929	88.19481	
Japan	Deaths	Male	2014	97.08645	102.3825	89.93555	
Japan	Deaths	Male	2015	96.1932	101.4627	88.69758	
Japan	Deaths	Male	2016	96.96933	102.7853	88.78504	
Japan	Deaths	Male	2017	99.65846	106.4404	90.99909	
Japan	Deaths	Male	2018	100.3125	107.2411	91.59368	
Japan	Deaths	Male	2019	102.4584	109.9919	93.47681	
Japan	Deaths	Male	2020	102.6124	110.1919	93.56722	
Japan	Deaths	Male	2021	104.8993	112.5882	95.69073	
Japan	Deaths	Male	2022	109.8145	117.1275	99.48079	
Japan	Deaths	Male	2023	113.8387	122.9947	100.5106	
Japan	Deaths	Female	2010	33.63276	38.04822	27.11765	
Japan	Deaths	Female	2011	34.62705	39.32842	27.60298	
Japan	Deaths	Female	2012	35.89304	40.78216	28.41841	
Japan	Deaths	Female	2013	36.97244	42.21061	29.03138	
Japan	Deaths	Female	2014	37.62103	43.05859	29.23273	
Japan	Deaths	Female	2015	38.11917	43.6569	29.44348	
Japan	Deaths	Female	2016	38.77674	44.72777	29.75686	
Japan	Deaths	Female	2017	39.71603	46.2646	30.32379	
Japan	Deaths	Female	2018	41.2159	48.21834	31.24917	
Japan	Deaths	Female	2019	42.41182	49.4843	31.99655	
Japan	Deaths	Female	2020	42.81619	50.23938	32.31033	
Japan	Deaths	Female	2021	44.54513	52.43631	33.84827	
Japan	Deaths	Female	2022	47.21337	55.3953	35.97339	
Japan	Deaths	Female	2023	50.05477	60.19257	36.46615	
Japan	DALYs	Male	2010	1675.702	1739.522	1588.462	
Japan	DALYs	Male	2011	1715.95	1785.915	1624.565	
Japan	DALYs	Male	2012	1733.077	1801.22	1634.274	
Japan	DALYs	Male	2013	1749.139	1821.474	1651.202	
Japan	DALYs	Male	2014	1769.596	1845.984	1668.333	

Japan	DALYs	Male	2015	1730.169	1807.161	1628.422	
Japan	DALYs	Male	2016	1719.404	1810.53	1611.008	
Japan	DALYs	Male	2017	1739.977	1841.804	1630.537	
Japan	DALYs	Male	2018	1732.344	1838.214	1620.987	
Japan	DALYs	Male	2019	1749.927	1860.48	1630.428	
Japan	DALYs	Male	2020	1737.223	1849.469	1616.15	
Japan	DALYs	Male	2021	1751.665	1864.742	1630.304	
Japan	DALYs	Male	2022	1812.486	1918.432	1677.394	
Japan	DALYs	Male	2023	1878.764	2007.254	1701.332	
Japan	DALYs	Female	2010	570.0301	630.3515	484.6059	
Japan	DALYs	Female	2011	580.6764	644.1326	493.8337	
Japan	DALYs	Female	2012	593.1583	660.4879	500.5607	
Japan	DALYs	Female	2013	603.9452	674.2918	506.8353	
Japan	DALYs	Female	2014	610.1406	679.1779	507.3629	
Japan	DALYs	Female	2015	607.226	678.6432	500.7442	
Japan	DALYs	Female	2016	607.1683	683.7099	498.8376	
Japan	DALYs	Female	2017	610.4276	695.0613	499.8521	
Japan	DALYs	Female	2018	626.021	716.024	509.3644	
Japan	DALYs	Female	2019	635.1875	727.8655	512.1234	
Japan	DALYs	Female	2020	635.356	729.5727	509.1408	
Japan	DALYs	Female	2021	651.2135	746.5436	524.9537	
Japan	DALYs	Female	2022	682.8059	780.0756	552.4153	
Japan	DALYs	Female	2023	721.5682	841.5996	568.3674	
Jordan	Incidence	Male	2010	13.75862	16.40861	11.1035	
Jordan	Incidence	Male	2011	13.85209	16.5626	11.18941	
Jordan	Incidence	Male	2012	13.95044	16.60198	11.35946	
Jordan	Incidence	Male	2013	14.22878	17.28722	11.74387	
Jordan	Incidence	Male	2014	14.21385	17.30471	11.91679	
Jordan	Incidence	Male	2015	14.48143	17.84548	12.17885	
Jordan	Incidence	Male	2016	14.60865	17.75075	12.31135	
Jordan	Incidence	Male	2017	14.91717	18.06218	12.68512	
Jordan	Incidence	Male	2018	15.45498	18.76987	13.01469	
Jordan	Incidence	Male	2019	15.97444	19.44079	13.3979	
Jordan	Incidence	Male	2020	16.67287	20.49517	13.8384	
Jordan	Incidence	Male	2021	18.09113	22.23696	14.81643	
Jordan	Incidence	Male	2022	17.7342	22.05245	14.29047	

Jordan	Incidence	Male	2023	18.13566	22.94127	14.42078	
Jordan	Incidence	Female	2010	3.773462	4.713432	3.005453	
Jordan	Incidence	Female	2011	3.911596	4.828756	3.121146	
Jordan	Incidence	Female	2012	4.0128	4.986448	3.226598	
Jordan	Incidence	Female	2013	4.123059	5.109439	3.274186	
Jordan	Incidence	Female	2014	4.2012	5.2597	3.327198	
Jordan	Incidence	Female	2015	4.215473	5.274622	3.365886	
Jordan	Incidence	Female	2016	4.268032	5.303012	3.470829	
Jordan	Incidence	Female	2017	4.366102	5.352976	3.608078	
Jordan	Incidence	Female	2018	4.498401	5.466642	3.743099	
Jordan	Incidence	Female	2019	4.64436	5.671963	3.847604	
Jordan	Incidence	Female	2020	4.912737	5.945263	4.191997	
Jordan	Incidence	Female	2021	5.545588	6.833565	4.68127	
Jordan	Incidence	Female	2022	5.083111	6.291344	4.215502	
Jordan	Incidence	Female	2023	5.176789	6.586765	4.192256	
Jordan	Deaths	Male	2010	13.07987	15.54597	10.50734	
Jordan	Deaths	Male	2011	13.13849	15.655	10.5656	
Jordan	Deaths	Male	2012	13.20496	15.68827	10.71373	
Jordan	Deaths	Male	2013	13.43877	16.28989	11.06738	
Jordan	Deaths	Male	2014	13.3819	16.24635	11.23443	
Jordan	Deaths	Male	2015	13.59605	16.66163	11.41692	
Jordan	Deaths	Male	2016	13.68602	16.5416	11.40872	
Jordan	Deaths	Male	2017	13.94093	16.9545	11.61542	
Jordan	Deaths	Male	2018	14.41456	17.64405	12.0854	
Jordan	Deaths	Male	2019	14.85972	18.22917	12.37875	
Jordan	Deaths	Male	2020	15.49093	19.31973	12.85239	
Jordan	Deaths	Male	2021	16.75381	20.73059	13.74606	
Jordan	Deaths	Male	2022	16.4109	20.31381	13.17448	
Jordan	Deaths	Male	2023	16.74921	21.10641	13.28091	
Jordan	Deaths	Female	2010	3.481779	4.413355	2.790348	
Jordan	Deaths	Female	2011	3.596044	4.497117	2.892026	
Jordan	Deaths	Female	2012	3.679205	4.562745	2.966104	
Jordan	Deaths	Female	2013	3.770035	4.678352	2.990863	
Jordan	Deaths	Female	2014	3.827914	4.77201	2.997321	
Jordan	Deaths	Female	2015	3.82762	4.733653	3.0775	
Jordan	Deaths	Female	2016	3.868498	4.790264	3.183574	

Jordan	Deaths	Female	2017	3.951379	4.805344	3.300907	
Jordan	Deaths	Female	2018	4.067231	4.863431	3.409265	
Jordan	Deaths	Female	2019	4.192408	5.127542	3.491549	
Jordan	Deaths	Female	2020	4.409907	5.406639	3.748661	
Jordan	Deaths	Female	2021	4.941696	6.061675	4.216821	
Jordan	Deaths	Female	2022	4.552321	5.736332	3.798535	
Jordan	Deaths	Female	2023	4.633268	5.820832	3.801799	
Jordan	DALYs	Male	2010	359.7555	433.9514	293.3891	
Jordan	DALYs	Male	2011	360.1856	427.7173	299.0122	
Jordan	DALYs	Male	2012	361.1178	427.6554	303.2091	
Jordan	DALYs	Male	2013	366.8017	446.5	307.8988	
Jordan	DALYs	Male	2014	365.5396	448.8536	309.2575	
Jordan	DALYs	Male	2015	371.6759	455.1364	311.5402	
Jordan	DALYs	Male	2016	374.1167	460.7033	313.8954	
Jordan	DALYs	Male	2017	381.5871	474.73	322.0623	
Jordan	DALYs	Male	2018	394.7323	490.3351	333.8168	
Jordan	DALYs	Male	2019	408.0809	506.5096	345.334	
Jordan	DALYs	Male	2020	423.3465	524.0382	355.244	
Jordan	DALYs	Male	2021	458.3023	564.6445	379.7382	
Jordan	DALYs	Male	2022	448.8057	553.8315	364.3495	
Jordan	DALYs	Male	2023	456.8694	572.3128	363.3437	
Jordan	DALYs	Female	2010	101.5498	129.4985	81.21219	
Jordan	DALYs	Female	2011	104.5466	132.1935	84.25062	
Jordan	DALYs	Female	2012	106.5803	133.6131	85.57865	
Jordan	DALYs	Female	2013	108.7589	135.3814	84.55639	
Jordan	DALYs	Female	2014	110.2147	138.4121	85.0833	
Jordan	DALYs	Female	2015	110.1727	137.792	87.95928	
Jordan	DALYs	Female	2016	110.9737	138.2953	89.75334	
Jordan	DALYs	Female	2017	113.0352	138.0796	93.0026	
Jordan	DALYs	Female	2018	115.9634	139.5209	97.68955	
Jordan	DALYs	Female	2019	119.4717	145.1207	99.06499	
Jordan	DALYs	Female	2020	126.1474	157.3955	106.843	
Jordan	DALYs	Female	2021	142.5689	178.3988	120.2649	
Jordan	DALYs	Female	2022	129.7719	165.0253	107.3683	
Jordan	DALYs	Female	2023	131.1999	166.3497	105.743	
Kazakhstan	Incidence	Male	2010	36.95954	39.73694	34.42583	

Kazakhstan	Incidence	Male	2011	37.35699	40.00165	34.73229	
Kazakhstan	Incidence	Male	2012	36.50667	38.79737	34.11027	
Kazakhstan	Incidence	Male	2013	34.08295	36.41215	31.91304	
Kazakhstan	Incidence	Male	2014	31.27632	33.50066	29.50337	
Kazakhstan	Incidence	Male	2015	29.57347	31.52631	27.91421	
Kazakhstan	Incidence	Male	2016	30.34226	32.23559	28.62085	
Kazakhstan	Incidence	Male	2017	29.75211	31.56758	27.89584	
Kazakhstan	Incidence	Male	2018	29.30343	31.2535	27.37863	
Kazakhstan	Incidence	Male	2019	28.88292	30.89038	26.8636	
Kazakhstan	Incidence	Male	2020	29.55536	31.43144	27.49111	
Kazakhstan	Incidence	Male	2021	27.12501	28.81497	25.46967	
Kazakhstan	Incidence	Male	2022	24.60739	26.93426	22.77641	
Kazakhstan	Incidence	Male	2023	26.22251	29.03312	23.95881	
Kazakhstan	Incidence	Female	2010	7.590678	8.265866	6.9561	
Kazakhstan	Incidence	Female	2011	7.885177	8.684724	6.917208	
Kazakhstan	Incidence	Female	2012	7.742772	8.492309	6.648317	
Kazakhstan	Incidence	Female	2013	6.974096	7.623541	6.026424	
Kazakhstan	Incidence	Female	2014	6.581709	7.127558	5.756894	
Kazakhstan	Incidence	Female	2015	6.075749	6.642241	5.294451	
Kazakhstan	Incidence	Female	2016	5.841117	6.41216	5.117834	
Kazakhstan	Incidence	Female	2017	5.643578	6.180458	4.937902	
Kazakhstan	Incidence	Female	2018	5.452068	5.979483	4.82447	
Kazakhstan	Incidence	Female	2019	5.548129	6.063604	4.904111	
Kazakhstan	Incidence	Female	2020	6.043966	6.547867	5.402158	
Kazakhstan	Incidence	Female	2021	5.840716	6.328433	5.227588	
Kazakhstan	Incidence	Female	2022	4.720777	5.225901	4.155893	
Kazakhstan	Incidence	Female	2023	4.875356	5.440571	4.223536	
Kazakhstan	Deaths	Male	2010	35.99579	38.65351	33.52822	
Kazakhstan	Deaths	Male	2011	36.47531	39.06079	34.02478	
Kazakhstan	Deaths	Male	2012	35.62749	37.97909	33.3899	
Kazakhstan	Deaths	Male	2013	33.07003	35.33983	30.98447	
Kazakhstan	Deaths	Male	2014	30.23537	32.3574	28.52818	
Kazakhstan	Deaths	Male	2015	28.60715	30.46922	27.17733	
Kazakhstan	Deaths	Male	2016	29.38837	31.26702	27.79957	
Kazakhstan	Deaths	Male	2017	28.79339	30.58188	27.15138	
Kazakhstan	Deaths	Male	2018	28.37653	30.16534	26.56803	

Kazakhstan	Deaths	Male	2019	27.98547	29.99615	26.08783	
Kazakhstan	Deaths	Male	2020	28.65091	30.41174	26.5738	
Kazakhstan	Deaths	Male	2021	26.19342	27.84343	24.69267	
Kazakhstan	Deaths	Male	2022	23.66836	25.73512	21.98102	
Kazakhstan	Deaths	Male	2023	25.27372	27.8702	23.08833	
Kazakhstan	Deaths	Female	2010	7.588595	8.258202	6.968484	
Kazakhstan	Deaths	Female	2011	7.96072	8.745023	6.989717	
Kazakhstan	Deaths	Female	2012	7.799701	8.526705	6.70707	
Kazakhstan	Deaths	Female	2013	6.947777	7.571357	5.998073	
Kazakhstan	Deaths	Female	2014	6.51618	7.062019	5.69186	
Kazakhstan	Deaths	Female	2015	5.972093	6.525241	5.199086	
Kazakhstan	Deaths	Female	2016	5.742796	6.273097	5.005996	
Kazakhstan	Deaths	Female	2017	5.543914	6.063058	4.838527	
Kazakhstan	Deaths	Female	2018	5.347145	5.857981	4.719267	
Kazakhstan	Deaths	Female	2019	5.424728	5.938864	4.775058	
Kazakhstan	Deaths	Female	2020	5.886157	6.388131	5.26061	
Kazakhstan	Deaths	Female	2021	5.704546	6.176863	5.112473	
Kazakhstan	Deaths	Female	2022	4.587902	5.04848	4.011519	
Kazakhstan	Deaths	Female	2023	4.743569	5.283823	4.113041	
Kazakhstan	DALYs	Male	2010	1015.548	1088.749	944.1567	
Kazakhstan	DALYs	Male	2011	1018.693	1092.371	948.3396	
Kazakhstan	DALYs	Male	2012	992.7379	1059.741	928.1171	
Kazakhstan	DALYs	Male	2013	931.5222	997.0295	871.3458	
Kazakhstan	DALYs	Male	2014	854.1526	916.6594	806.029	
Kazakhstan	DALYs	Male	2015	799.4966	846.3539	756.7748	
Kazakhstan	DALYs	Male	2016	812.3033	868.1535	766.9877	
Kazakhstan	DALYs	Male	2017	793.7371	844.3072	749.5643	
Kazakhstan	DALYs	Male	2018	778.1849	823.8051	728.3503	
Kazakhstan	DALYs	Male	2019	762.4874	813.6358	714.4267	
Kazakhstan	DALYs	Male	2020	775.2995	823.9477	722.7791	
Kazakhstan	DALYs	Male	2021	714.1812	757.5407	675.8661	
Kazakhstan	DALYs	Male	2022	652.757	707.511	607.4403	
Kazakhstan	DALYs	Male	2023	691.9552	765.3214	633.5481	
Kazakhstan	DALYs	Female	2010	202.2238	220.6705	186.1724	
Kazakhstan	DALYs	Female	2011	206.0998	227.5649	182.7649	
Kazakhstan	DALYs	Female	2012	201.8602	221.2021	176.579	

Kazakhstan	DALYs	Female	2013	183.4455	199.732	159.8844	
Kazakhstan	DALYs	Female	2014	173.6954	187.948	152.4684	
Kazakhstan	DALYs	Female	2015	161.5547	175.4183	142.083	
Kazakhstan	DALYs	Female	2016	154.357	169.1534	135.8458	
Kazakhstan	DALYs	Female	2017	148.9678	163.559	131.5417	
Kazakhstan	DALYs	Female	2018	144.2133	158.2591	128.2259	
Kazakhstan	DALYs	Female	2019	146.7418	161.4956	131.4311	
Kazakhstan	DALYs	Female	2020	160.1218	174.7221	144.9853	
Kazakhstan	DALYs	Female	2021	152.9159	167.2855	137.6336	
Kazakhstan	DALYs	Female	2022	125.5722	139.2686	110.6896	
Kazakhstan	DALYs	Female	2023	129.1079	143.9752	113.2639	
Kenya	Incidence	Male	2010	1.624962	2.361775	1.152426	
Kenya	Incidence	Male	2011	1.655906	2.329402	1.202325	
Kenya	Incidence	Male	2012	1.677584	2.34271	1.178151	
Kenya	Incidence	Male	2013	1.689314	2.345804	1.176305	
Kenya	Incidence	Male	2014	1.725632	2.483749	1.22222	
Kenya	Incidence	Male	2015	1.782669	2.530891	1.258475	
Kenya	Incidence	Male	2016	1.826714	2.532061	1.2585	
Kenya	Incidence	Male	2017	1.856467	2.695547	1.309478	
Kenya	Incidence	Male	2018	1.928465	2.831676	1.378953	
Kenya	Incidence	Male	2019	1.985593	2.961951	1.394169	
Kenya	Incidence	Male	2020	2.330463	3.475981	1.648541	
Kenya	Incidence	Male	2021	2.664111	3.888687	1.847815	
Kenya	Incidence	Male	2022	2.254353	3.304115	1.504876	
Kenya	Incidence	Male	2023	2.377983	3.416117	1.606655	
Kenya	Incidence	Female	2010	0.949456	1.376116	0.661587	
Kenya	Incidence	Female	2011	0.967224	1.415406	0.666919	
Kenya	Incidence	Female	2012	0.978972	1.436768	0.646665	
Kenya	Incidence	Female	2013	0.971081	1.435187	0.657467	
Kenya	Incidence	Female	2014	0.944102	1.340706	0.614325	
Kenya	Incidence	Female	2015	0.997916	1.385761	0.647728	
Kenya	Incidence	Female	2016	1.04427	1.449823	0.683836	
Kenya	Incidence	Female	2017	1.099279	1.503056	0.718197	
Kenya	Incidence	Female	2018	1.154644	1.559727	0.758345	
Kenya	Incidence	Female	2019	1.201998	1.590896	0.822108	
Kenya	Incidence	Female	2020	1.229948	1.632943	0.831205	

Kenya	Incidence	Female	2021	1.401857	1.86576	0.966707	
Kenya	Incidence	Female	2022	1.445432	1.987224	0.968627	
Kenya	Incidence	Female	2023	1.535176	2.16129	1.053562	
Kenya	Deaths	Male	2010	1.571863	2.27874	1.107322	
Kenya	Deaths	Male	2011	1.601955	2.251727	1.153488	
Kenya	Deaths	Male	2012	1.62398	2.278431	1.139806	
Kenya	Deaths	Male	2013	1.636515	2.263252	1.145793	
Kenya	Deaths	Male	2014	1.672274	2.402153	1.184159	
Kenya	Deaths	Male	2015	1.726718	2.446062	1.21677	
Kenya	Deaths	Male	2016	1.768632	2.467409	1.2126	
Kenya	Deaths	Male	2017	1.79733	2.605085	1.261834	
Kenya	Deaths	Male	2018	1.867182	2.731132	1.322964	
Kenya	Deaths	Male	2019	1.923346	2.885198	1.350112	
Kenya	Deaths	Male	2020	2.279235	3.412574	1.596245	
Kenya	Deaths	Male	2021	2.60898	3.811542	1.81019	
Kenya	Deaths	Male	2022	2.190497	3.213248	1.466824	
Kenya	Deaths	Male	2023	2.310227	3.32805	1.542297	
Kenya	Deaths	Female	2010	0.907774	1.309463	0.633704	
Kenya	Deaths	Female	2011	0.922737	1.34455	0.640381	
Kenya	Deaths	Female	2012	0.932343	1.361622	0.613487	
Kenya	Deaths	Female	2013	0.925407	1.364034	0.627955	
Kenya	Deaths	Female	2014	0.898276	1.284382	0.586544	
Kenya	Deaths	Female	2015	0.947631	1.323587	0.616718	
Kenya	Deaths	Female	2016	0.989774	1.367578	0.646861	
Kenya	Deaths	Female	2017	1.040074	1.430494	0.681081	
Kenya	Deaths	Female	2018	1.091074	1.47326	0.716881	
Kenya	Deaths	Female	2019	1.135197	1.506186	0.781597	
Kenya	Deaths	Female	2020	1.159718	1.556388	0.786569	
Kenya	Deaths	Female	2021	1.317519	1.752868	0.910285	
Kenya	Deaths	Female	2022	1.359732	1.859186	0.914755	
Kenya	Deaths	Female	2023	1.444317	2.026954	0.992822	
Kenya	DALYs	Male	2010	47.97086	70.06091	34.6345	
Kenya	DALYs	Male	2011	48.82861	69.36797	36.26968	
Kenya	DALYs	Male	2012	49.32094	68.44119	34.6019	
Kenya	DALYs	Male	2013	49.51539	69.71737	35.13012	
Kenya	DALYs	Male	2014	50.49577	72.58889	35.90014	

Kenya	DALYs	Male	2015	52.13876	73.68004	36.93057	
Kenya	DALYs	Male	2016	53.39308	73.08947	38.20296	
Kenya	DALYs	Male	2017	54.15168	79.10746	38.59685	
Kenya	DALYs	Male	2018	56.14606	83.33253	40.69143	
Kenya	DALYs	Male	2019	57.66747	85.43943	40.89054	
Kenya	DALYs	Male	2020	66.36101	97.9708	47.10634	
Kenya	DALYs	Male	2021	75.53164	111.05	52.52211	
Kenya	DALYs	Male	2022	64.65841	96.00213	44.28332	
Kenya	DALYs	Male	2023	68.03314	97.59389	46.70504	
Kenya	DALYs	Female	2010	29.38037	42.97182	20.07621	
Kenya	DALYs	Female	2011	30.0127	44.04281	20.24711	
Kenya	DALYs	Female	2012	30.43285	44.89596	20.22739	
Kenya	DALYs	Female	2013	30.07255	44.31389	20.15073	
Kenya	DALYs	Female	2014	29.27175	41.86494	18.65287	
Kenya	DALYs	Female	2015	31.01107	43.0329	19.93522	
Kenya	DALYs	Female	2016	32.51438	44.41806	20.90133	
Kenya	DALYs	Female	2017	34.25789	46.74094	22.446	
Kenya	DALYs	Female	2018	35.97843	49.17444	23.39855	
Kenya	DALYs	Female	2019	37.41503	50.00497	25.59415	
Kenya	DALYs	Female	2020	38.26567	51.27957	25.6636	
Kenya	DALYs	Female	2021	43.69628	58.75733	29.8652	
Kenya	DALYs	Female	2022	44.99639	61.74807	30.43391	
Kenya	DALYs	Female	2023	47.65927	67.17807	32.46932	
Kuwait	Incidence	Male	2010	5.329358	6.40069	4.529502	
Kuwait	Incidence	Male	2011	5.677306	6.703876	4.802506	
Kuwait	Incidence	Male	2012	5.872797	7.00877	5.015959	
Kuwait	Incidence	Male	2013	6.099351	7.273664	5.198672	
Kuwait	Incidence	Male	2014	5.365226	6.773509	4.387979	
Kuwait	Incidence	Male	2015	5.886817	6.936275	5.043695	
Kuwait	Incidence	Male	2016	5.461948	6.404271	4.72318	
Kuwait	Incidence	Male	2017	5.800405	6.846632	4.951655	
Kuwait	Incidence	Male	2018	6.36525	7.549145	5.425996	
Kuwait	Incidence	Male	2019	6.023498	7.173389	5.099927	
Kuwait	Incidence	Male	2020	5.356023	6.396197	4.593388	
Kuwait	Incidence	Male	2021	5.323395	6.354683	4.534073	
Kuwait	Incidence	Male	2022	6.137861	7.379063	5.143836	

Kuwait	Incidence	Male	2023	5.781208	7.080722	4.75387	
Kuwait	Incidence	Female	2010	2.7344	3.356822	2.241247	
Kuwait	Incidence	Female	2011	2.822893	3.416302	2.285019	
Kuwait	Incidence	Female	2012	2.948724	3.591688	2.373792	
Kuwait	Incidence	Female	2013	2.824221	3.420269	2.275253	
Kuwait	Incidence	Female	2014	2.884506	3.529636	2.346089	
Kuwait	Incidence	Female	2015	2.943189	3.624897	2.41287	
Kuwait	Incidence	Female	2016	3.010106	3.677138	2.4485	
Kuwait	Incidence	Female	2017	3.151343	3.896871	2.556078	
Kuwait	Incidence	Female	2018	3.295007	3.985706	2.662754	
Kuwait	Incidence	Female	2019	3.412953	4.118524	2.784699	
Kuwait	Incidence	Female	2020	3.296314	3.986477	2.695564	
Kuwait	Incidence	Female	2021	3.394329	4.0757	2.724239	
Kuwait	Incidence	Female	2022	3.769359	4.535759	3.006347	
Kuwait	Incidence	Female	2023	3.899212	4.674496	3.040406	
Kuwait	Deaths	Male	2010	4.575389	5.489547	3.910111	
Kuwait	Deaths	Male	2011	4.85166	5.667643	4.156633	
Kuwait	Deaths	Male	2012	4.97452	5.837286	4.27436	
Kuwait	Deaths	Male	2013	5.152045	6.064573	4.425587	
Kuwait	Deaths	Male	2014	4.514516	5.569851	3.726452	
Kuwait	Deaths	Male	2015	4.905866	5.768408	4.223451	
Kuwait	Deaths	Male	2016	4.530498	5.3098	3.937304	
Kuwait	Deaths	Male	2017	4.776317	5.650763	4.182854	
Kuwait	Deaths	Male	2018	5.228115	6.099446	4.474673	
Kuwait	Deaths	Male	2019	4.925255	5.810593	4.244525	
Kuwait	Deaths	Male	2020	4.369364	5.117864	3.785389	
Kuwait	Deaths	Male	2021	4.337591	5.118539	3.70583	
Kuwait	Deaths	Male	2022	4.979594	5.942533	4.204701	
Kuwait	Deaths	Male	2023	4.682336	5.686737	3.826501	
Kuwait	Deaths	Female	2010	2.277644	2.739239	1.903972	
Kuwait	Deaths	Female	2011	2.321559	2.790526	1.904092	
Kuwait	Deaths	Female	2012	2.407728	2.867432	1.953605	
Kuwait	Deaths	Female	2013	2.297767	2.718966	1.865834	
Kuwait	Deaths	Female	2014	2.329571	2.823162	1.879593	
Kuwait	Deaths	Female	2015	2.356181	2.822951	1.906834	
Kuwait	Deaths	Female	2016	2.394953	2.870173	1.956996	

Kuwait	Deaths	Female	2017	2.490886	2.97966	2.04871	
Kuwait	Deaths	Female	2018	2.599429	3.094825	2.102441	
Kuwait	Deaths	Female	2019	2.682634	3.191113	2.219694	
Kuwait	Deaths	Female	2020	2.608097	3.127563	2.156057	
Kuwait	Deaths	Female	2021	2.687756	3.214509	2.23452	
Kuwait	Deaths	Female	2022	2.964375	3.496896	2.423281	
Kuwait	Deaths	Female	2023	3.062277	3.650989	2.452447	
Kuwait	DALYs	Male	2010	141.437	170.3908	120.4883	
Kuwait	DALYs	Male	2011	147.9501	173.5198	127.0422	
Kuwait	DALYs	Male	2012	153.0681	179.1606	131.5558	
Kuwait	DALYs	Male	2013	157.8151	184.9783	135.42	
Kuwait	DALYs	Male	2014	137.4542	170.6075	112.9042	
Kuwait	DALYs	Male	2015	150.9063	177.9932	130.0841	
Kuwait	DALYs	Male	2016	139.1853	163.8364	121.1542	
Kuwait	DALYs	Male	2017	146.1917	173.0967	127.5001	
Kuwait	DALYs	Male	2018	158.2048	184.6525	135.3079	
Kuwait	DALYs	Male	2019	148.9163	175.1607	128.1977	
Kuwait	DALYs	Male	2020	132.4134	154.9204	113.8064	
Kuwait	DALYs	Male	2021	131.1662	154.2568	111.6316	
Kuwait	DALYs	Male	2022	151.0055	179.4345	127.452	
Kuwait	DALYs	Male	2023	142.0907	172.4034	116.5759	
Kuwait	DALYs	Female	2010	69.54294	84.4114	58.00582	
Kuwait	DALYs	Female	2011	70.91623	84.85821	58.02706	
Kuwait	DALYs	Female	2012	73.04971	87.26841	59.39843	
Kuwait	DALYs	Female	2013	69.15245	82.39024	56.63926	
Kuwait	DALYs	Female	2014	70.25109	84.77327	57.00609	
Kuwait	DALYs	Female	2015	71.09668	84.98654	58.10193	
Kuwait	DALYs	Female	2016	71.73284	86.19698	58.8754	
Kuwait	DALYs	Female	2017	73.68919	88.42041	60.55623	
Kuwait	DALYs	Female	2018	75.97199	90.46809	62.30247	
Kuwait	DALYs	Female	2019	78.37032	93.496	64.76703	
Kuwait	DALYs	Female	2020	74.14344	88.39097	61.18843	
Kuwait	DALYs	Female	2021	75.83667	90.94711	63.48044	
Kuwait	DALYs	Female	2022	84.34085	99.65397	69.37943	
Kuwait	DALYs	Female	2023	86.68121	103.9436	69.39879	
Kyrgyzstan	Incidence	Male	2010	13.25156	14.34521	12.29139	

Kyrgyzstan	Incidence	Male	2011	13.49485	14.67857	12.39689	
Kyrgyzstan	Incidence	Male	2012	13.4868	14.6489	12.42958	
Kyrgyzstan	Incidence	Male	2013	13.8635	15.10209	12.88778	
Kyrgyzstan	Incidence	Male	2014	14.085	15.26434	13.05283	
Kyrgyzstan	Incidence	Male	2015	12.48136	13.53715	11.50072	
Kyrgyzstan	Incidence	Male	2016	12.93695	14.14079	11.91148	
Kyrgyzstan	Incidence	Male	2017	12.78615	14.20383	11.64163	
Kyrgyzstan	Incidence	Male	2018	12.98664	14.36316	11.89328	
Kyrgyzstan	Incidence	Male	2019	11.61413	12.87197	10.64659	
Kyrgyzstan	Incidence	Male	2020	10.09131	11.17898	9.096997	
Kyrgyzstan	Incidence	Male	2021	10.52092	11.69688	9.598623	
Kyrgyzstan	Incidence	Male	2022	10.6644	11.96317	9.61875	
Kyrgyzstan	Incidence	Male	2023	12.00823	13.70208	10.28467	
Kyrgyzstan	Incidence	Female	2010	3.748	4.276464	3.30037	
Kyrgyzstan	Incidence	Female	2011	3.646664	4.152322	3.213517	
Kyrgyzstan	Incidence	Female	2012	3.663706	4.22597	3.202402	
Kyrgyzstan	Incidence	Female	2013	3.717079	4.245432	3.27935	
Kyrgyzstan	Incidence	Female	2014	3.684086	4.254918	3.250606	
Kyrgyzstan	Incidence	Female	2015	3.627416	4.174054	3.146909	
Kyrgyzstan	Incidence	Female	2016	3.876531	4.460902	3.34501	
Kyrgyzstan	Incidence	Female	2017	3.589242	4.095301	3.096822	
Kyrgyzstan	Incidence	Female	2018	3.470108	4.029927	3.026721	
Kyrgyzstan	Incidence	Female	2019	3.237524	3.793635	2.784607	
Kyrgyzstan	Incidence	Female	2020	2.870986	3.392431	2.488597	
Kyrgyzstan	Incidence	Female	2021	3.279991	3.861503	2.837564	
Kyrgyzstan	Incidence	Female	2022	3.40103	4.054864	2.914186	
Kyrgyzstan	Incidence	Female	2023	3.774431	4.582114	3.181668	
Kyrgyzstan	Deaths	Male	2010	12.75359	13.78619	11.8454	
Kyrgyzstan	Deaths	Male	2011	12.99582	14.11029	11.9645	
Kyrgyzstan	Deaths	Male	2012	12.97533	14.0648	12.00552	
Kyrgyzstan	Deaths	Male	2013	13.37625	14.56773	12.45879	
Kyrgyzstan	Deaths	Male	2014	13.58604	14.69634	12.53526	
Kyrgyzstan	Deaths	Male	2015	12.03102	12.99693	11.08294	
Kyrgyzstan	Deaths	Male	2016	12.4775	13.64588	11.50433	
Kyrgyzstan	Deaths	Male	2017	12.33445	13.74794	11.26464	
Kyrgyzstan	Deaths	Male	2018	12.54618	13.86253	11.492	

Kyrgyzstan	Deaths	Male	2019	11.23063	12.41786	10.33087	
Kyrgyzstan	Deaths	Male	2020	9.778393	10.8584	8.830042	
Kyrgyzstan	Deaths	Male	2021	10.19382	11.29941	9.270499	
Kyrgyzstan	Deaths	Male	2022	10.30599	11.5227	9.27946	
Kyrgyzstan	Deaths	Male	2023	11.60535	13.20115	10.00922	
Kyrgyzstan	Deaths	Female	2010	3.68426	4.200777	3.245497	
Kyrgyzstan	Deaths	Female	2011	3.579269	4.072413	3.173088	
Kyrgyzstan	Deaths	Female	2012	3.592321	4.138384	3.140762	
Kyrgyzstan	Deaths	Female	2013	3.631304	4.144012	3.201287	
Kyrgyzstan	Deaths	Female	2014	3.609015	4.179453	3.191447	
Kyrgyzstan	Deaths	Female	2015	3.562564	4.105651	3.092577	
Kyrgyzstan	Deaths	Female	2016	3.79277	4.377368	3.286505	
Kyrgyzstan	Deaths	Female	2017	3.510692	3.997782	3.035846	
Kyrgyzstan	Deaths	Female	2018	3.392191	3.946192	2.956065	
Kyrgyzstan	Deaths	Female	2019	3.156565	3.6923	2.723529	
Kyrgyzstan	Deaths	Female	2020	2.812209	3.313633	2.42638	
Kyrgyzstan	Deaths	Female	2021	3.217318	3.786104	2.804579	
Kyrgyzstan	Deaths	Female	2022	3.327906	3.966939	2.867537	
Kyrgyzstan	Deaths	Female	2023	3.686294	4.454121	3.1178	
Kyrgyzstan	DALYs	Male	2010	379.0797	410.8666	350.8931	
Kyrgyzstan	DALYs	Male	2011	385.0013	420.0972	352.1323	
Kyrgyzstan	DALYs	Male	2012	385.4551	420.7738	356.0809	
Kyrgyzstan	DALYs	Male	2013	392.8358	430.4485	365.244	
Kyrgyzstan	DALYs	Male	2014	397.3443	432.9872	365.3357	
Kyrgyzstan	DALYs	Male	2015	351.2481	381.2241	322.2918	
Kyrgyzstan	DALYs	Male	2016	361.2211	397.5689	331.1819	
Kyrgyzstan	DALYs	Male	2017	354.4217	396.8157	323.0765	
Kyrgyzstan	DALYs	Male	2018	356.3428	398.1674	325.6947	
Kyrgyzstan	DALYs	Male	2019	316.7262	353.0746	289.3761	
Kyrgyzstan	DALYs	Male	2020	272.9832	304.1123	245.2103	
Kyrgyzstan	DALYs	Male	2021	283.2464	317.9791	255.9459	
Kyrgyzstan	DALYs	Male	2022	288.1617	324.0966	258.4269	
Kyrgyzstan	DALYs	Male	2023	323.5183	369.8046	279.8845	
Kyrgyzstan	DALYs	Female	2010	105.0001	119.5201	92.68349	
Kyrgyzstan	DALYs	Female	2011	102.8435	117.1216	90.08846	
Kyrgyzstan	DALYs	Female	2012	103.1606	119.9522	90.16566	

Kyrgyzstan	DALYs	Female	2013	104.5986	119.2622	92.01732	
Kyrgyzstan	DALYs	Female	2014	102.7696	118.0262	90.86635	
Kyrgyzstan	DALYs	Female	2015	100.0412	115.5643	87.21786	
Kyrgyzstan	DALYs	Female	2016	106.5797	122.9374	92.44734	
Kyrgyzstan	DALYs	Female	2017	98.53155	112.617	85.38163	
Kyrgyzstan	DALYs	Female	2018	94.76109	110.2694	82.67789	
Kyrgyzstan	DALYs	Female	2019	88.61508	103.862	75.70212	
Kyrgyzstan	DALYs	Female	2020	77.38234	90.96129	67.09204	
Kyrgyzstan	DALYs	Female	2021	87.42445	103.0062	76.17003	
Kyrgyzstan	DALYs	Female	2022	90.55979	106.9903	77.78963	
Kyrgyzstan	DALYs	Female	2023	100.2567	121.8615	84.4602	
Lao People's De	Incidence	Male	2010	11.33917	15.55776	8.046202	
Lao People's De	Incidence	Male	2011	11.70116	16.47501	8.528665	
Lao People's De	Incidence	Male	2012	12.14696	17.18141	8.752246	
Lao People's De	Incidence	Male	2013	12.72525	18.52328	9.242872	
Lao People's De	Incidence	Male	2014	13.04019	18.89419	9.064729	
Lao People's De	Incidence	Male	2015	13.84566	19.71629	9.585005	
Lao People's De	Incidence	Male	2016	14.24184	20.32781	9.764616	
Lao People's De	Incidence	Male	2017	14.43454	21.47013	10.00584	
Lao People's De	Incidence	Male	2018	14.8134	21.88874	10.01843	
Lao People's De	Incidence	Male	2019	15.32255	22.41115	10.40954	
Lao People's De	Incidence	Male	2020	15.9927	23.31118	10.97664	
Lao People's De	Incidence	Male	2021	16.32536	24.21633	11.38948	
Lao People's De	Incidence	Male	2022	17.94959	25.87876	12.31782	
Lao People's De	Incidence	Male	2023	18.61347	26.78303	12.73482	
Lao People's De	Incidence	Female	2010	4.848289	7.07967	3.158128	
Lao People's De	Incidence	Female	2011	5.15788	7.677486	3.330613	
Lao People's De	Incidence	Female	2012	5.414253	8.021287	3.45969	
Lao People's De	Incidence	Female	2013	5.8214	8.571441	3.729769	
Lao People's De	Incidence	Female	2014	6.155941	8.976328	3.826946	
Lao People's De	Incidence	Female	2015	6.623693	9.403872	4.115888	
Lao People's De	Incidence	Female	2016	6.993151	9.842217	4.39766	
Lao People's De	Incidence	Female	2017	7.292328	10.1923	4.595758	
Lao People's De	Incidence	Female	2018	7.61964	10.771	4.900273	
Lao People's De	Incidence	Female	2019	8.02418	11.50326	5.216849	
Lao People's De	Incidence	Female	2020	8.5276	12.00611	5.731497	

Lao People's De	Incidence	Female	2021	8.785624	12.38766	5.680096	
Lao People's De	Incidence	Female	2022	9.832314	13.95685	6.384956	
Lao People's De	Incidence	Female	2023	10.35077	14.61004	6.795224	
Lao People's De	Deaths	Male	2010	11.47154	15.5898	8.130893	
Lao People's De	Deaths	Male	2011	11.81959	16.69667	8.576112	
Lao People's De	Deaths	Male	2012	12.25486	17.39811	8.815737	
Lao People's De	Deaths	Male	2013	12.81983	18.58962	9.314443	
Lao People's De	Deaths	Male	2014	13.12036	19.00803	9.12966	
Lao People's De	Deaths	Male	2015	13.91115	19.76568	9.630893	
Lao People's De	Deaths	Male	2016	14.29347	20.51261	9.813179	
Lao People's De	Deaths	Male	2017	14.47224	21.47622	10.00385	
Lao People's De	Deaths	Male	2018	14.83577	21.98296	10.03426	
Lao People's De	Deaths	Male	2019	15.32554	22.44237	10.43905	
Lao People's De	Deaths	Male	2020	15.98014	23.26531	11.00448	
Lao People's De	Deaths	Male	2021	16.31019	24.32901	11.39494	
Lao People's De	Deaths	Male	2022	17.91237	25.81801	12.29377	
Lao People's De	Deaths	Male	2023	18.55328	26.5967	12.62693	
Lao People's De	Deaths	Female	2010	4.778039	6.990358	3.098059	
Lao People's De	Deaths	Female	2011	5.074753	7.534912	3.271309	
Lao People's De	Deaths	Female	2012	5.319086	7.921437	3.394714	
Lao People's De	Deaths	Female	2013	5.707202	8.362944	3.645122	
Lao People's De	Deaths	Female	2014	6.024726	8.770335	3.763958	
Lao People's De	Deaths	Female	2015	6.472125	9.283442	4.004888	
Lao People's De	Deaths	Female	2016	6.819969	9.594952	4.288883	
Lao People's De	Deaths	Female	2017	7.100341	9.915435	4.472078	
Lao People's De	Deaths	Female	2018	7.405611	10.44792	4.740591	
Lao People's De	Deaths	Female	2019	7.783466	11.21678	5.082094	
Lao People's De	Deaths	Female	2020	8.25749	11.60542	5.576815	
Lao People's De	Deaths	Female	2021	8.491806	12.00301	5.473772	
Lao People's De	Deaths	Female	2022	9.481711	13.44049	6.171018	
Lao People's De	Deaths	Female	2023	9.960481	14.04773	6.50528	
Lao People's De	DALYs	Male	2010	305.6918	420.695	217.9486	
Lao People's De	DALYs	Male	2011	316.197	444.081	231.4556	
Lao People's De	DALYs	Male	2012	328.7349	464.4098	237.8359	
Lao People's De	DALYs	Male	2013	345.1176	507.3118	250.4405	
Lao People's De	DALYs	Male	2014	354.266	513.3105	246.6402	

Lao People's De	DALYs	Male	2015	376.9877	537.9124	260.7684	
Lao People's De	DALYs	Male	2016	388.3999	556.4633	264.565	
Lao People's De	DALYs	Male	2017	394.2596	580.942	274.365	
Lao People's De	DALYs	Male	2018	405.1577	595.254	275.5599	
Lao People's De	DALYs	Male	2019	419.4762	612.4607	286.0141	
Lao People's De	DALYs	Male	2020	437.7047	637.7569	301.8939	
Lao People's De	DALYs	Male	2021	445.8609	649.3722	307.9086	
Lao People's De	DALYs	Male	2022	489.7605	693.9581	333.3361	
Lao People's De	DALYs	Male	2023	508.286	728.2584	354.864	
Lao People's De	DALYs	Female	2010	140.4272	207.2173	92.37999	
Lao People's De	DALYs	Female	2011	149.6755	223.9724	95.75956	
Lao People's De	DALYs	Female	2012	157.2823	231.1614	99.45589	
Lao People's De	DALYs	Female	2013	169.5465	251.4235	108.4388	
Lao People's De	DALYs	Female	2014	179.6732	264.4009	110.177	
Lao People's De	DALYs	Female	2015	193.9112	274.576	119.4393	
Lao People's De	DALYs	Female	2016	205.2818	288.892	127.239	
Lao People's De	DALYs	Female	2017	214.513	297.9212	134.4826	
Lao People's De	DALYs	Female	2018	224.5792	316.8426	144.2472	
Lao People's De	DALYs	Female	2019	236.8638	338.8922	153.2811	
Lao People's De	DALYs	Female	2020	251.8422	353.3286	167.6276	
Lao People's De	DALYs	Female	2021	259.2012	362.3914	168.9262	
Lao People's De	DALYs	Female	2022	289.6749	408.0636	187.6382	
Lao People's De	DALYs	Female	2023	305.3781	424.7895	199.3122	
Latvia	Incidence	Male	2010	94.7579	101.0007	89.39553	
Latvia	Incidence	Male	2011	93.48309	100.1515	88.46078	
Latvia	Incidence	Male	2012	92.10431	99.05279	86.86213	
Latvia	Incidence	Male	2013	92.32618	98.98954	87.04721	
Latvia	Incidence	Male	2014	91.24105	97.92543	84.86986	
Latvia	Incidence	Male	2015	85.81306	92.36564	80.26615	
Latvia	Incidence	Male	2016	88.46266	94.67643	82.49085	
Latvia	Incidence	Male	2017	89.51796	95.7129	83.81883	
Latvia	Incidence	Male	2018	87.77219	95.00315	81.70263	
Latvia	Incidence	Male	2019	88.71167	95.62399	82.46999	
Latvia	Incidence	Male	2020	88.27414	94.9782	82.25071	
Latvia	Incidence	Male	2021	90.3358	97.99516	84.18084	
Latvia	Incidence	Male	2022	93.18082	102.1063	85.91229	

Latvia	Incidence	Male	2023	91.50047	100.9395	84.28058	
Latvia	Incidence	Female	2010	18.24672	20.41215	16.35122	
Latvia	Incidence	Female	2011	18.9576	21.48591	16.89692	
Latvia	Incidence	Female	2012	19.53116	22.05967	17.25834	
Latvia	Incidence	Female	2013	19.61413	22.25916	17.28355	
Latvia	Incidence	Female	2014	19.56133	22.19184	17.34324	
Latvia	Incidence	Female	2015	20.03032	22.94729	17.7049	
Latvia	Incidence	Female	2016	19.98964	22.81538	17.55504	
Latvia	Incidence	Female	2017	21.11533	24.13687	18.41197	
Latvia	Incidence	Female	2018	21.93585	24.88386	19.11216	
Latvia	Incidence	Female	2019	21.52943	24.32796	18.87174	
Latvia	Incidence	Female	2020	22.57664	25.59075	19.89059	
Latvia	Incidence	Female	2021	24.6646	28.01664	21.85282	
Latvia	Incidence	Female	2022	24.62242	28.20299	21.34905	
Latvia	Incidence	Female	2023	23.65723	27.61969	20.4678	
Latvia	Deaths	Male	2010	89.49189	94.548	85.01861	
Latvia	Deaths	Male	2011	88.19962	93.23711	83.5975	
Latvia	Deaths	Male	2012	86.71423	91.71046	82.36826	
Latvia	Deaths	Male	2013	86.70564	90.90003	82.59595	
Latvia	Deaths	Male	2014	85.57086	90.12329	80.59729	
Latvia	Deaths	Male	2015	80.3239	84.56738	76.06354	
Latvia	Deaths	Male	2016	82.78837	87.26187	78.3182	
Latvia	Deaths	Male	2017	83.66553	88.32035	79.42744	
Latvia	Deaths	Male	2018	81.78413	87.19666	77.33633	
Latvia	Deaths	Male	2019	82.54468	87.20639	78.53759	
Latvia	Deaths	Male	2020	81.88897	87.30243	78.05325	
Latvia	Deaths	Male	2021	83.51792	89.14066	79.49084	
Latvia	Deaths	Male	2022	85.86171	92.4825	80.25246	
Latvia	Deaths	Male	2023	84.21305	91.20164	78.96635	
Latvia	Deaths	Female	2010	17.44766	19.54241	15.80553	
Latvia	Deaths	Female	2011	18.04602	20.40452	16.26363	
Latvia	Deaths	Female	2012	18.55132	20.94516	16.68292	
Latvia	Deaths	Female	2013	18.69289	21.3044	16.58967	
Latvia	Deaths	Female	2014	18.57694	21.18581	16.57155	
Latvia	Deaths	Female	2015	18.96972	21.80536	16.96644	
Latvia	Deaths	Female	2016	18.99124	21.50946	17.1086	

Latvia	Deaths	Female	2017	20.01014	22.84807	17.8062	
Latvia	Deaths	Female	2018	20.76632	23.49921	18.44377	
Latvia	Deaths	Female	2019	20.35247	22.92091	18.14512	
Latvia	Deaths	Female	2020	21.1378	23.77164	18.89489	
Latvia	Deaths	Female	2021	23.08492	25.91933	20.66984	
Latvia	Deaths	Female	2022	22.98452	25.97448	20.23035	
Latvia	Deaths	Female	2023	22.06166	25.29269	19.28884	
Latvia	DALYs	Male	2010	2205.747	2348.404	2097.459	
Latvia	DALYs	Male	2011	2153.924	2289.087	2045.692	
Latvia	DALYs	Male	2012	2102.281	2228.351	1990.245	
Latvia	DALYs	Male	2013	2086.798	2197.632	1968.846	
Latvia	DALYs	Male	2014	2041.527	2156.53	1915.263	
Latvia	DALYs	Male	2015	1906.928	2024.319	1796.121	
Latvia	DALYs	Male	2016	1942.627	2055.954	1835.551	
Latvia	DALYs	Male	2017	1948.59	2076.752	1842.106	
Latvia	DALYs	Male	2018	1908.248	2042.108	1799.108	
Latvia	DALYs	Male	2019	1921.82	2046.566	1823.709	
Latvia	DALYs	Male	2020	1913.387	2043.223	1820.302	
Latvia	DALYs	Male	2021	1979.621	2123.255	1876.384	
Latvia	DALYs	Male	2022	2056.218	2234.66	1908.886	
Latvia	DALYs	Male	2023	2013.415	2210.224	1870.565	
Latvia	DALYs	Female	2010	383.7084	429.3309	347.5085	
Latvia	DALYs	Female	2011	397.4808	448.7371	357.7427	
Latvia	DALYs	Female	2012	405.1237	455.5671	365.2201	
Latvia	DALYs	Female	2013	397.5273	450.5076	356.7041	
Latvia	DALYs	Female	2014	396.1549	455.0995	356.5377	
Latvia	DALYs	Female	2015	403.5863	462.2409	362.7983	
Latvia	DALYs	Female	2016	394.6318	448.5	355.4912	
Latvia	DALYs	Female	2017	415.566	471.4545	374.385	
Latvia	DALYs	Female	2018	427.5908	484.1293	380.3081	
Latvia	DALYs	Female	2019	415.4622	470.9753	371.5007	
Latvia	DALYs	Female	2020	440.2768	496.691	393.4432	
Latvia	DALYs	Female	2021	479.1367	538.0887	428.5155	
Latvia	DALYs	Female	2022	479.3596	542.9489	419.2277	
Latvia	DALYs	Female	2023	458.5765	524.6018	401.1931	
Lebanon	Incidence	Male	2010	30.71304	40.34811	23.77595	

Lebanon	Incidence	Male	2011	33.89823	43.52319	26.69854	
Lebanon	Incidence	Male	2012	37.73672	49.00951	29.71236	
Lebanon	Incidence	Male	2013	40.93359	53.49744	31.97107	
Lebanon	Incidence	Male	2014	39.08732	50.92906	31.24403	
Lebanon	Incidence	Male	2015	33.83047	43.02097	27.06331	
Lebanon	Incidence	Male	2016	30.1954	38.60265	24.16035	
Lebanon	Incidence	Male	2017	29.68103	37.22321	23.61998	
Lebanon	Incidence	Male	2018	33.81278	42.59561	27.68067	
Lebanon	Incidence	Male	2019	45.49938	58.30783	37.38242	
Lebanon	Incidence	Male	2020	60.24019	76.18447	49.53791	
Lebanon	Incidence	Male	2021	38.04564	47.86169	30.94634	
Lebanon	Incidence	Male	2022	39.26358	50.29519	30.70693	
Lebanon	Incidence	Male	2023	40.98105	54.0894	31.48463	
Lebanon	Incidence	Female	2010	15.30883	18.81877	11.98404	
Lebanon	Incidence	Female	2011	16.9229	21.07738	13.21598	
Lebanon	Incidence	Female	2012	18.80154	22.9791	14.75232	
Lebanon	Incidence	Female	2013	20.4464	24.99501	16.15975	
Lebanon	Incidence	Female	2014	19.99696	24.91942	15.77588	
Lebanon	Incidence	Female	2015	18.28112	22.69705	14.12357	
Lebanon	Incidence	Female	2016	16.85312	20.45034	13.34574	
Lebanon	Incidence	Female	2017	16.93149	20.86625	13.81044	
Lebanon	Incidence	Female	2018	18.94439	23.06862	15.09458	
Lebanon	Incidence	Female	2019	23.95631	29.2436	19.30273	
Lebanon	Incidence	Female	2020	29.37901	36.07562	23.84384	
Lebanon	Incidence	Female	2021	18.31412	22.66287	14.45291	
Lebanon	Incidence	Female	2022	22.02503	27.50721	16.51851	
Lebanon	Incidence	Female	2023	22.85542	29.24173	17.14865	
Lebanon	Deaths	Male	2010	30.53266	39.65756	23.94167	
Lebanon	Deaths	Male	2011	33.63989	43.1339	26.42721	
Lebanon	Deaths	Male	2012	37.41464	48.16652	29.54581	
Lebanon	Deaths	Male	2013	40.57071	51.75329	32.17073	
Lebanon	Deaths	Male	2014	38.69166	50.30501	31.1673	
Lebanon	Deaths	Male	2015	33.32179	42.41168	26.6903	
Lebanon	Deaths	Male	2016	29.4843	37.86728	23.46203	
Lebanon	Deaths	Male	2017	28.7703	36.42573	23.05563	
Lebanon	Deaths	Male	2018	32.54198	41.51246	26.62674	

Lebanon	Deaths	Male	2019	43.54992	56.0171	35.82481	
Lebanon	Deaths	Male	2020	57.32955	72.77758	47.58374	
Lebanon	Deaths	Male	2021	36.05792	45.62401	29.41231	
Lebanon	Deaths	Male	2022	37.30102	47.47541	29.38284	
Lebanon	Deaths	Male	2023	38.96395	50.79128	30.14707	
Lebanon	Deaths	Female	2010	14.894	18.47042	11.75578	
Lebanon	Deaths	Female	2011	16.48133	20.60687	12.75719	
Lebanon	Deaths	Female	2012	18.36196	22.62742	14.24003	
Lebanon	Deaths	Female	2013	19.99986	24.43176	15.64921	
Lebanon	Deaths	Female	2014	19.51285	24.01286	15.13766	
Lebanon	Deaths	Female	2015	17.66264	21.85671	13.51602	
Lebanon	Deaths	Female	2016	16.04595	19.5258	12.62	
Lebanon	Deaths	Female	2017	15.88925	19.33605	12.72775	
Lebanon	Deaths	Female	2018	17.62191	21.60587	13.9554	
Lebanon	Deaths	Female	2019	22.18765	26.95942	17.80253	
Lebanon	Deaths	Female	2020	27.11122	33.05794	22.04835	
Lebanon	Deaths	Female	2021	16.76595	20.73752	13.21751	
Lebanon	Deaths	Female	2022	20.2861	25.56487	15.13195	
Lebanon	Deaths	Female	2023	21.13058	27.79482	15.73259	
Lebanon	DALYs	Male	2010	732.4473	956.7505	568.8788	
Lebanon	DALYs	Male	2011	804.1933	1037.647	641.0746	
Lebanon	DALYs	Male	2012	887.8543	1120.355	702.2901	
Lebanon	DALYs	Male	2013	952.8204	1207.496	751.3439	
Lebanon	DALYs	Male	2014	902.5912	1147.743	726.7006	
Lebanon	DALYs	Male	2015	781.014	988.7773	623.5392	
Lebanon	DALYs	Male	2016	702.3694	891.4426	561.4978	
Lebanon	DALYs	Male	2017	696.6673	871.3945	561.2863	
Lebanon	DALYs	Male	2018	798.3848	1016.59	656.071	
Lebanon	DALYs	Male	2019	1068.545	1382.963	870.1656	
Lebanon	DALYs	Male	2020	1401.097	1779.608	1147.289	
Lebanon	DALYs	Male	2021	879.6555	1111.577	715.9486	
Lebanon	DALYs	Male	2022	924.3708	1183.157	731.5507	
Lebanon	DALYs	Male	2023	962.395	1239.182	752.9237	
Lebanon	DALYs	Female	2010	369.5761	456.7293	296.1876	
Lebanon	DALYs	Female	2011	402.9207	496.0507	319.4553	
Lebanon	DALYs	Female	2012	440.1282	534.5824	347.5018	

Lebanon	DALYs	Female	2013	471.1316	570.3869	377.6757	
Lebanon	DALYs	Female	2014	457.663	560.3289	367.4593	
Lebanon	DALYs	Female	2015	421.1672	510.9225	334.8919	
Lebanon	DALYs	Female	2016	394.3281	480.8816	315.5977	
Lebanon	DALYs	Female	2017	401.9214	494.7271	332.254	
Lebanon	DALYs	Female	2018	450.717	558.3345	367.9251	
Lebanon	DALYs	Female	2019	561.2392	707.8748	450.3048	
Lebanon	DALYs	Female	2020	676.9345	844.0387	551.2256	
Lebanon	DALYs	Female	2021	422.4551	528.8842	344.4277	
Lebanon	DALYs	Female	2022	510.9543	638.6861	394.0358	
Lebanon	DALYs	Female	2023	526.2455	657.891	399.6099	
Lesotho	Incidence	Male	2010	7.360852	11.36799	4.239118	
Lesotho	Incidence	Male	2011	7.746507	11.7743	4.406106	
Lesotho	Incidence	Male	2012	8.205729	12.51164	4.778782	
Lesotho	Incidence	Male	2013	8.855185	13.74536	4.964799	
Lesotho	Incidence	Male	2014	9.858001	15.72093	5.569967	
Lesotho	Incidence	Male	2015	10.75143	16.90312	6.001182	
Lesotho	Incidence	Male	2016	10.17275	16.11392	5.537231	
Lesotho	Incidence	Male	2017	9.429208	14.84555	5.259749	
Lesotho	Incidence	Male	2018	9.343056	15.39633	5.257068	
Lesotho	Incidence	Male	2019	9.463138	15.85761	5.328149	
Lesotho	Incidence	Male	2020	9.26974	15.22533	5.232962	
Lesotho	Incidence	Male	2021	9.767769	15.80808	5.414767	
Lesotho	Incidence	Male	2022	11.41434	18.1348	6.450829	
Lesotho	Incidence	Male	2023	12.25925	19.85373	6.940499	
Lesotho	Incidence	Female	2010	2.693545	4.157512	1.666849	
Lesotho	Incidence	Female	2011	2.822489	4.27045	1.699933	
Lesotho	Incidence	Female	2012	2.892901	4.416264	1.702062	
Lesotho	Incidence	Female	2013	2.961126	4.507972	1.791311	
Lesotho	Incidence	Female	2014	3.036962	4.714426	1.834977	
Lesotho	Incidence	Female	2015	3.130308	4.766044	1.883505	
Lesotho	Incidence	Female	2016	3.189376	4.788799	1.964189	
Lesotho	Incidence	Female	2017	3.256911	4.77471	2.034017	
Lesotho	Incidence	Female	2018	3.327995	4.822721	2.09394	
Lesotho	Incidence	Female	2019	3.389567	4.824929	2.121482	
Lesotho	Incidence	Female	2020	3.368576	4.845539	2.11373	

Lesotho	Incidence	Female	2021	3.774457	5.440763	2.3892	
Lesotho	Incidence	Female	2022	4.103501	6.207931	2.434902	
Lesotho	Incidence	Female	2023	4.23778	6.337257	2.599952	
Lesotho	Deaths	Male	2010	7.249133	11.10613	4.1851	
Lesotho	Deaths	Male	2011	7.612835	11.66217	4.305296	
Lesotho	Deaths	Male	2012	8.04908	12.16372	4.688931	
Lesotho	Deaths	Male	2013	8.668501	13.42318	4.841095	
Lesotho	Deaths	Male	2014	9.630728	15.30824	5.414726	
Lesotho	Deaths	Male	2015	10.50255	16.41997	5.882104	
Lesotho	Deaths	Male	2016	9.970191	15.71238	5.457902	
Lesotho	Deaths	Male	2017	9.273512	14.53414	5.182929	
Lesotho	Deaths	Male	2018	9.208345	15.0756	5.198348	
Lesotho	Deaths	Male	2019	9.345573	15.65196	5.26807	
Lesotho	Deaths	Male	2020	9.191137	14.98945	5.158763	
Lesotho	Deaths	Male	2021	9.708071	15.59795	5.356629	
Lesotho	Deaths	Male	2022	11.34074	17.98567	6.372318	
Lesotho	Deaths	Male	2023	12.16699	19.68286	6.814171	
Lesotho	Deaths	Female	2010	2.746348	4.225735	1.713081	
Lesotho	Deaths	Female	2011	2.871342	4.324186	1.753594	
Lesotho	Deaths	Female	2012	2.94303	4.422291	1.747508	
Lesotho	Deaths	Female	2013	3.014698	4.513489	1.827619	
Lesotho	Deaths	Female	2014	3.0954	4.745161	1.877444	
Lesotho	Deaths	Female	2015	3.195118	4.89549	1.934454	
Lesotho	Deaths	Female	2016	3.259459	4.912067	2.00542	
Lesotho	Deaths	Female	2017	3.331521	4.911633	2.066864	
Lesotho	Deaths	Female	2018	3.40691	4.984403	2.118978	
Lesotho	Deaths	Female	2019	3.476604	5.000631	2.132663	
Lesotho	Deaths	Female	2020	3.462231	4.931676	2.142219	
Lesotho	Deaths	Female	2021	3.867976	5.548639	2.428735	
Lesotho	Deaths	Female	2022	4.207427	6.279044	2.484774	
Lesotho	Deaths	Female	2023	4.346031	6.509646	2.65529	
Lesotho	DALYs	Male	2010	211.0748	331.799	121.3584	
Lesotho	DALYs	Male	2011	222.9111	339.3155	127.0855	
Lesotho	DALYs	Male	2012	236.7461	359.7723	137.8739	
Lesotho	DALYs	Male	2013	256.1978	400.4299	144.964	
Lesotho	DALYs	Male	2014	285.8147	456.7799	161.7011	

Lesotho	DALYs	Male	2015	311.0866	496.0526	173.3355	
Lesotho	DALYs	Male	2016	292.0052	467.0657	158.6215	
Lesotho	DALYs	Male	2017	268.4542	430.56	149.0088	
Lesotho	DALYs	Male	2018	264.4562	442.6603	149.5416	
Lesotho	DALYs	Male	2019	266.2047	448.7763	149.2399	
Lesotho	DALYs	Male	2020	258.7643	432.3138	145.9957	
Lesotho	DALYs	Male	2021	271.6881	444.8942	151.6399	
Lesotho	DALYs	Male	2022	315.6471	504.3668	176.4752	
Lesotho	DALYs	Male	2023	339.4275	550.7349	192.1073	
Lesotho	DALYs	Female	2010	72.31961	112.3268	43.91688	
Lesotho	DALYs	Female	2011	76.11841	116.4211	44.46559	
Lesotho	DALYs	Female	2012	77.96733	122.4676	45.40263	
Lesotho	DALYs	Female	2013	79.65206	123.7971	47.98768	
Lesotho	DALYs	Female	2014	81.44301	125.8879	49.2583	
Lesotho	DALYs	Female	2015	83.68838	126.3322	50.37284	
Lesotho	DALYs	Female	2016	84.98728	126.7979	51.89638	
Lesotho	DALYs	Female	2017	86.54271	128.1537	53.65022	
Lesotho	DALYs	Female	2018	88.19094	126.4451	55.6273	
Lesotho	DALYs	Female	2019	89.31382	126.1296	55.69161	
Lesotho	DALYs	Female	2020	88.39997	129.0858	54.02747	
Lesotho	DALYs	Female	2021	99.25869	144.1281	62.31273	
Lesotho	DALYs	Female	2022	107.6091	163.1589	65.79742	
Lesotho	DALYs	Female	2023	111.0046	166.0989	66.15661	
Liberia	Incidence	Male	2010	3.112013	4.486035	2.083464	
Liberia	Incidence	Male	2011	3.168772	4.57795	2.147622	
Liberia	Incidence	Male	2012	3.234074	4.765165	2.10366	
Liberia	Incidence	Male	2013	3.208111	4.773986	2.106266	
Liberia	Incidence	Male	2014	3.151517	4.645887	2.10725	
Liberia	Incidence	Male	2015	3.327009	4.810946	2.242036	
Liberia	Incidence	Male	2016	3.321574	4.770552	2.215534	
Liberia	Incidence	Male	2017	3.295541	4.758718	2.113923	
Liberia	Incidence	Male	2018	3.413389	5.026159	2.231709	
Liberia	Incidence	Male	2019	3.567555	5.398126	2.331663	
Liberia	Incidence	Male	2020	4.041551	6.223958	2.696737	
Liberia	Incidence	Male	2021	4.514077	6.883081	2.957324	
Liberia	Incidence	Male	2022	4.170462	6.629832	2.696375	

Liberia	Incidence	Male	2023	4.415742	6.851521	2.752621	
Liberia	Incidence	Female	2010	1.103918	1.663658	0.722881	
Liberia	Incidence	Female	2011	1.127144	1.700981	0.746821	
Liberia	Incidence	Female	2012	1.188496	1.814184	0.795706	
Liberia	Incidence	Female	2013	1.230506	1.897424	0.80468	
Liberia	Incidence	Female	2014	1.210997	1.891333	0.796261	
Liberia	Incidence	Female	2015	1.245476	1.870947	0.822625	
Liberia	Incidence	Female	2016	1.252719	1.802174	0.820387	
Liberia	Incidence	Female	2017	1.296096	1.900492	0.838763	
Liberia	Incidence	Female	2018	1.365328	1.933188	0.909861	
Liberia	Incidence	Female	2019	1.449771	1.999835	0.980358	
Liberia	Incidence	Female	2020	1.48592	2.030412	1.009516	
Liberia	Incidence	Female	2021	1.701503	2.312679	1.132775	
Liberia	Incidence	Female	2022	1.81998	2.57791	1.177932	
Liberia	Incidence	Female	2023	1.945808	2.8418	1.28493	
Liberia	Deaths	Male	2010	3.124154	4.504757	2.099891	
Liberia	Deaths	Male	2011	3.171073	4.61521	2.162166	
Liberia	Deaths	Male	2012	3.226009	4.754588	2.11766	
Liberia	Deaths	Male	2013	3.193334	4.782376	2.090996	
Liberia	Deaths	Male	2014	3.124541	4.627408	2.087348	
Liberia	Deaths	Male	2015	3.287015	4.763618	2.216072	
Liberia	Deaths	Male	2016	3.270492	4.715883	2.184251	
Liberia	Deaths	Male	2017	3.23807	4.686338	2.073222	
Liberia	Deaths	Male	2018	3.340623	4.919673	2.175825	
Liberia	Deaths	Male	2019	3.48045	5.271523	2.280227	
Liberia	Deaths	Male	2020	3.935557	6.066795	2.624301	
Liberia	Deaths	Male	2021	4.371573	6.658213	2.849517	
Liberia	Deaths	Male	2022	4.043874	6.41249	2.610443	
Liberia	Deaths	Male	2023	4.279424	6.62354	2.661493	
Liberia	Deaths	Female	2010	1.095358	1.649919	0.707245	
Liberia	Deaths	Female	2011	1.114597	1.696252	0.738693	
Liberia	Deaths	Female	2012	1.171326	1.789486	0.784326	
Liberia	Deaths	Female	2013	1.206966	1.861815	0.791851	
Liberia	Deaths	Female	2014	1.180761	1.843811	0.774591	
Liberia	Deaths	Female	2015	1.210407	1.814472	0.798817	
Liberia	Deaths	Female	2016	1.211126	1.737672	0.797098	

Liberia	Deaths	Female	2017	1.244733	1.81568	0.809609	
Liberia	Deaths	Female	2018	1.302343	1.854246	0.869523	
Liberia	Deaths	Female	2019	1.377472	1.8941	0.928015	
Liberia	Deaths	Female	2020	1.404533	1.912928	0.955811	
Liberia	Deaths	Female	2021	1.597738	2.16229	1.063747	
Liberia	Deaths	Female	2022	1.713322	2.421883	1.104149	
Liberia	Deaths	Female	2023	1.829047	2.667808	1.19978	
Liberia	DALYs	Male	2010	85.66137	123.0752	56.46369	
Liberia	DALYs	Male	2011	87.89687	126.4895	58.4187	
Liberia	DALYs	Male	2012	90.3137	133.101	59.01162	
Liberia	DALYs	Male	2013	90.16475	132.0182	59.1794	
Liberia	DALYs	Male	2014	89.38605	131.489	59.86633	
Liberia	DALYs	Male	2015	95.0111	137.2195	63.84494	
Liberia	DALYs	Male	2016	95.44961	136.6594	63.33089	
Liberia	DALYs	Male	2017	95.01432	137.4716	61.11622	
Liberia	DALYs	Male	2018	99.04744	146.1528	63.61983	
Liberia	DALYs	Male	2019	103.9923	157.2957	66.84129	
Liberia	DALYs	Male	2020	117.7005	181.1696	77.02455	
Liberia	DALYs	Male	2021	132.4172	201.4524	87.54882	
Liberia	DALYs	Male	2022	122.1905	194.2735	78.84682	
Liberia	DALYs	Male	2023	129.255	201.4173	81.92634	
Liberia	DALYs	Female	2010	32.3563	48.96213	21.38596	
Liberia	DALYs	Female	2011	33.29605	49.64175	22.19362	
Liberia	DALYs	Female	2012	35.34212	53.62564	23.76833	
Liberia	DALYs	Female	2013	36.91825	56.28701	24.04003	
Liberia	DALYs	Female	2014	36.73983	56.32287	24.10801	
Liberia	DALYs	Female	2015	38.0084	56.78941	24.97321	
Liberia	DALYs	Female	2016	38.6036	56.01126	24.74646	
Liberia	DALYs	Female	2017	40.46595	60.11243	25.86857	
Liberia	DALYs	Female	2018	43.07267	60.6636	28.62538	
Liberia	DALYs	Female	2019	45.93146	64.0178	30.80817	
Liberia	DALYs	Female	2020	47.35073	65.62459	31.76262	
Liberia	DALYs	Female	2021	54.49019	74.43239	36.52032	
Liberia	DALYs	Female	2022	58.14913	82.13051	37.94737	
Liberia	DALYs	Female	2023	62.2599	92.73423	41.46254	
Libya	Incidence	Male	2010	19.38218	28.07687	12.80262	

Libya	Incidence	Male	2011	19.04131	27.84437	12.60014	
Libya	Incidence	Male	2012	19.44583	28.92675	12.80045	
Libya	Incidence	Male	2013	19.80704	29.46724	13.04142	
Libya	Incidence	Male	2014	19.62584	29.12925	12.97027	
Libya	Incidence	Male	2015	19.6706	28.64468	12.60838	
Libya	Incidence	Male	2016	19.67337	29.35598	12.52248	
Libya	Incidence	Male	2017	20.07856	29.83305	12.64367	
Libya	Incidence	Male	2018	21.30072	31.80776	13.40809	
Libya	Incidence	Male	2019	22.15854	32.5171	14.06464	
Libya	Incidence	Male	2020	23.26857	33.46134	14.63049	
Libya	Incidence	Male	2021	25.72941	37.17672	16.52528	
Libya	Incidence	Male	2022	24.46956	37.38322	15.36806	
Libya	Incidence	Male	2023	25.80396	40.28668	16.30056	
Libya	Incidence	Female	2010	2.857005	4.113098	1.827194	
Libya	Incidence	Female	2011	2.915197	4.250233	1.880396	
Libya	Incidence	Female	2012	3.00928	4.31134	1.938906	
Libya	Incidence	Female	2013	3.001908	4.295175	1.907857	
Libya	Incidence	Female	2014	3.020512	4.339515	1.925442	
Libya	Incidence	Female	2015	2.98768	4.373823	1.897222	
Libya	Incidence	Female	2016	2.941518	4.262663	1.858299	
Libya	Incidence	Female	2017	3.040316	4.40733	1.989588	
Libya	Incidence	Female	2018	3.20746	4.66688	2.098646	
Libya	Incidence	Female	2019	3.354152	4.707845	2.156376	
Libya	Incidence	Female	2020	3.619362	5.035629	2.279221	
Libya	Incidence	Female	2021	4.239168	5.860548	2.679285	
Libya	Incidence	Female	2022	3.767739	5.32716	2.422435	
Libya	Incidence	Female	2023	3.927187	5.485578	2.537784	
Libya	Deaths	Male	2010	18.48317	26.85882	12.12065	
Libya	Deaths	Male	2011	18.16751	26.74026	11.849	
Libya	Deaths	Male	2012	18.52441	27.49762	12.14238	
Libya	Deaths	Male	2013	18.85686	28.08058	12.35985	
Libya	Deaths	Male	2014	18.70292	27.90378	12.30355	
Libya	Deaths	Male	2015	18.76042	27.46334	12.04621	
Libya	Deaths	Male	2016	18.76673	27.91534	11.91408	
Libya	Deaths	Male	2017	19.13332	28.61186	12.01931	
Libya	Deaths	Male	2018	20.25165	30.24201	12.76244	

Libya	Deaths	Male	2019	21.03524	30.99325	13.4338	
Libya	Deaths	Male	2020	22.13409	31.9866	13.97257	
Libya	Deaths	Male	2021	24.41041	35.26523	15.67793	
Libya	Deaths	Male	2022	23.2227	34.8478	14.62991	
Libya	Deaths	Male	2023	24.41572	37.6799	15.49948	
Libya	Deaths	Female	2010	2.609664	3.760724	1.665321	
Libya	Deaths	Female	2011	2.665215	3.872475	1.72681	
Libya	Deaths	Female	2012	2.746729	3.917612	1.774172	
Libya	Deaths	Female	2013	2.739177	3.91141	1.760071	
Libya	Deaths	Female	2014	2.762134	3.960097	1.786579	
Libya	Deaths	Female	2015	2.736974	3.995324	1.763772	
Libya	Deaths	Female	2016	2.696722	3.895873	1.729865	
Libya	Deaths	Female	2017	2.78538	4.020973	1.825611	
Libya	Deaths	Female	2018	2.93087	4.250414	1.944907	
Libya	Deaths	Female	2019	3.060447	4.328698	1.989771	
Libya	Deaths	Female	2020	3.305411	4.617908	2.121943	
Libya	Deaths	Female	2021	3.854371	5.30806	2.447937	
Libya	Deaths	Female	2022	3.440918	4.820601	2.198876	
Libya	Deaths	Female	2023	3.581776	5.01341	2.320962	
Libya	DALYs	Male	2010	524.4673	755.7274	358.3526	
Libya	DALYs	Male	2011	516.1382	750.793	350.1162	
Libya	DALYs	Male	2012	527.6088	776.1425	357.532	
Libya	DALYs	Male	2013	539.7859	792.6119	359.6624	
Libya	DALYs	Male	2014	537.6224	793.1523	359.2321	
Libya	DALYs	Male	2015	540.7014	782.4964	353.1194	
Libya	DALYs	Male	2016	542.1154	788.3503	350.7156	
Libya	DALYs	Male	2017	553.4021	814.5867	355.0204	
Libya	DALYs	Male	2018	587.0065	866.6765	374.4052	
Libya	DALYs	Male	2019	610.1706	896.9395	382.6275	
Libya	DALYs	Male	2020	637.504	919.4943	396.6429	
Libya	DALYs	Male	2021	706.6681	1015.664	458.6233	
Libya	DALYs	Male	2022	671.1087	1003.468	431.6973	
Libya	DALYs	Male	2023	708.7077	1088.531	456.1133	
Libya	DALYs	Female	2010	80.69341	114.8609	51.77134	
Libya	DALYs	Female	2011	82.40492	119.8518	53.46329	
Libya	DALYs	Female	2012	84.98435	119.5657	54.76304	

Libya	DALYs	Female	2013	85.01462	120.2145	53.93933	
Libya	DALYs	Female	2014	85.79042	122.4159	54.71922	
Libya	DALYs	Female	2015	85.13151	122.949	53.95398	
Libya	DALYs	Female	2016	83.97262	121.4374	53.23669	
Libya	DALYs	Female	2017	86.61013	124.2	56.37252	
Libya	DALYs	Female	2018	91.11341	129.4953	58.99968	
Libya	DALYs	Female	2019	94.92264	132.1243	60.57266	
Libya	DALYs	Female	2020	102.0114	141.8203	65.03473	
Libya	DALYs	Female	2021	119.8694	163.7261	75.0733	
Libya	DALYs	Female	2022	105.7105	150.119	67.91011	
Libya	DALYs	Female	2023	109.8145	153.69	71.40742	
Lithuania	Incidence	Male	2010	93.61757	98.75771	88.8382	
Lithuania	Incidence	Male	2011	95.66757	100.5616	90.37259	
Lithuania	Incidence	Male	2012	94.58754	100.2596	89.83403	
Lithuania	Incidence	Male	2013	93.65952	99.31926	88.69695	
Lithuania	Incidence	Male	2014	96.20958	102.388	90.91437	
Lithuania	Incidence	Male	2015	93.86397	100.7736	88.08402	
Lithuania	Incidence	Male	2016	90.83066	97.56075	85.14248	
Lithuania	Incidence	Male	2017	89.37678	96.48971	83.86001	
Lithuania	Incidence	Male	2018	89.29931	96.40771	83.37212	
Lithuania	Incidence	Male	2019	91.25811	98.81348	84.97614	
Lithuania	Incidence	Male	2020	87.07832	94.77317	81.06226	
Lithuania	Incidence	Male	2021	81.43556	88.44708	75.1658	
Lithuania	Incidence	Male	2022	80.17298	87.20145	74.32843	
Lithuania	Incidence	Male	2023	78.15533	86.76924	71.73719	
Lithuania	Incidence	Female	2010	15.0345	16.78397	13.66454	
Lithuania	Incidence	Female	2011	16.00742	17.78316	14.53069	
Lithuania	Incidence	Female	2012	17.40608	19.5625	15.60887	
Lithuania	Incidence	Female	2013	17.97085	20.12134	16.16543	
Lithuania	Incidence	Female	2014	18.63926	20.83384	16.66085	
Lithuania	Incidence	Female	2015	18.30433	20.36802	16.46865	
Lithuania	Incidence	Female	2016	18.95393	20.98305	17.06689	
Lithuania	Incidence	Female	2017	19.92131	22.17421	17.70376	
Lithuania	Incidence	Female	2018	20.33534	22.60243	18.06909	
Lithuania	Incidence	Female	2019	21.01065	23.42771	18.5792	
Lithuania	Incidence	Female	2020	20.87144	23.35599	18.61507	

Lithuania	Incidence	Female	2021	20.29094	22.44871	18.16104	
Lithuania	Incidence	Female	2022	20.97811	23.16758	18.44352	
Lithuania	Incidence	Female	2023	20.44332	22.83101	17.86367	
Lithuania	Deaths	Male	2010	86.21612	90.51497	82.09112	
Lithuania	Deaths	Male	2011	87.81487	92.1067	83.35427	
Lithuania	Deaths	Male	2012	86.5999	90.94119	82.23667	
Lithuania	Deaths	Male	2013	85.65939	90.79211	81.31389	
Lithuania	Deaths	Male	2014	88.06726	93.50629	83.3549	
Lithuania	Deaths	Male	2015	86.05025	92.16147	81.43613	
Lithuania	Deaths	Male	2016	83.33764	88.41113	79.07836	
Lithuania	Deaths	Male	2017	81.81131	87.02797	77.58245	
Lithuania	Deaths	Male	2018	81.67178	86.72517	77.40482	
Lithuania	Deaths	Male	2019	83.5129	88.57813	79.10133	
Lithuania	Deaths	Male	2020	79.7203	84.71879	75.56478	
Lithuania	Deaths	Male	2021	74.37194	79.52612	70.44699	
Lithuania	Deaths	Male	2022	73.09004	78.3086	68.62941	
Lithuania	Deaths	Male	2023	70.97601	77.43526	66.48161	
Lithuania	Deaths	Female	2010	13.93412	15.48845	12.77032	
Lithuania	Deaths	Female	2011	14.90345	16.6177	13.68692	
Lithuania	Deaths	Female	2012	16.11746	17.8248	14.72163	
Lithuania	Deaths	Female	2013	16.62525	18.41054	15.12551	
Lithuania	Deaths	Female	2014	17.33432	19.06375	15.85028	
Lithuania	Deaths	Female	2015	17.10469	18.91592	15.58734	
Lithuania	Deaths	Female	2016	17.69461	19.47687	16.05743	
Lithuania	Deaths	Female	2017	18.52545	20.45701	16.80813	
Lithuania	Deaths	Female	2018	18.94197	21.13543	17.17192	
Lithuania	Deaths	Female	2019	19.41656	21.54384	17.55158	
Lithuania	Deaths	Female	2020	19.27677	21.13668	17.49482	
Lithuania	Deaths	Female	2021	18.7274	20.55891	17.10274	
Lithuania	Deaths	Female	2022	19.30394	21.00711	17.36819	
Lithuania	Deaths	Female	2023	18.70718	20.5438	16.80531	
Lithuania	DALYs	Male	2010	2129.09	2242.799	2031.693	
Lithuania	DALYs	Male	2011	2157.658	2270.194	2058.446	
Lithuania	DALYs	Male	2012	2124.876	2237.747	2022.224	
Lithuania	DALYs	Male	2013	2100.968	2231.479	1993.238	
Lithuania	DALYs	Male	2014	2155.329	2293.238	2037.488	

Lithuania	DALYs	Male	2015	2090.473	2236.996	1968.494	
Lithuania	DALYs	Male	2016	1999.059	2131.983	1890.728	
Lithuania	DALYs	Male	2017	1957.601	2075.684	1851.582	
Lithuania	DALYs	Male	2018	1944.724	2069.833	1837.722	
Lithuania	DALYs	Male	2019	1963.114	2093.742	1857.579	
Lithuania	DALYs	Male	2020	1857.977	1987.445	1760.281	
Lithuania	DALYs	Male	2021	1744.452	1868.919	1645.406	
Lithuania	DALYs	Male	2022	1717.857	1863.469	1612.593	
Lithuania	DALYs	Male	2023	1683.055	1851.521	1569.487	
Lithuania	DALYs	Female	2010	312.6958	347.6555	284.2709	
Lithuania	DALYs	Female	2011	321.6606	359.9217	293.3947	
Lithuania	DALYs	Female	2012	351.1125	390.9906	317.4376	
Lithuania	DALYs	Female	2013	363.9932	406.6322	328.451	
Lithuania	DALYs	Female	2014	374.9322	417.0079	341.6204	
Lithuania	DALYs	Female	2015	364.3332	407.7451	329.9564	
Lithuania	DALYs	Female	2016	373.9143	415.8229	339.2864	
Lithuania	DALYs	Female	2017	391.3057	436.9435	354.9702	
Lithuania	DALYs	Female	2018	392.5647	436.5155	355.269	
Lithuania	DALYs	Female	2019	405.7332	445.1574	367.3938	
Lithuania	DALYs	Female	2020	399.102	438.4397	364.464	
Lithuania	DALYs	Female	2021	385.116	419.5559	351.6856	
Lithuania	DALYs	Female	2022	397.2785	431.8532	359.2548	
Lithuania	DALYs	Female	2023	386.5055	426.7736	346.496	
Luxembourg	Incidence	Male	2010	82.01551	92.02033	73.69135	
Luxembourg	Incidence	Male	2011	80.84756	89.31527	71.82843	
Luxembourg	Incidence	Male	2012	78.66838	87.02821	70.57485	
Luxembourg	Incidence	Male	2013	78.13085	87.1296	69.89729	
Luxembourg	Incidence	Male	2014	80.44661	90.81866	71.97196	
Luxembourg	Incidence	Male	2015	76.47185	86.29251	68.52186	
Luxembourg	Incidence	Male	2016	74.84452	84.44626	67.32963	
Luxembourg	Incidence	Male	2017	68.8312	77.61537	61.71112	
Luxembourg	Incidence	Male	2018	66.04816	74.36589	58.73282	
Luxembourg	Incidence	Male	2019	62.54363	71.1042	55.26478	
Luxembourg	Incidence	Male	2020	58.99923	66.94777	52.36417	
Luxembourg	Incidence	Male	2021	58.42708	66.33584	51.22442	
Luxembourg	Incidence	Male	2022	54.64768	62.31849	47.35267	

Luxembourg	Incidence	Male	2023	57.65192	66.26126	50.02742	
Luxembourg	Incidence	Female	2010	34.07444	40.38723	29.03536	
Luxembourg	Incidence	Female	2011	35.03217	41.51224	29.95596	
Luxembourg	Incidence	Female	2012	36.49418	43.40963	31.30799	
Luxembourg	Incidence	Female	2013	35.4605	41.8549	29.93357	
Luxembourg	Incidence	Female	2014	37.03269	43.84837	31.39385	
Luxembourg	Incidence	Female	2015	35.76122	42.36903	30.21501	
Luxembourg	Incidence	Female	2016	35.18862	41.41451	30.08221	
Luxembourg	Incidence	Female	2017	35.93792	42.30277	30.34506	
Luxembourg	Incidence	Female	2018	35.82713	42.31016	30.24849	
Luxembourg	Incidence	Female	2019	35.48301	41.64722	30.46894	
Luxembourg	Incidence	Female	2020	35.32936	41.51982	30.31324	
Luxembourg	Incidence	Female	2021	33.30708	38.81678	28.56351	
Luxembourg	Incidence	Female	2022	33.67213	40.0173	28.68915	
Luxembourg	Incidence	Female	2023	35.50994	42.18644	29.98104	
Luxembourg	Deaths	Male	2010	72.89839	79.81462	67.44107	
Luxembourg	Deaths	Male	2011	72.1185	79.16572	66.0243	
Luxembourg	Deaths	Male	2012	69.80171	77.23765	64.45119	
Luxembourg	Deaths	Male	2013	69.5053	76.70923	63.55146	
Luxembourg	Deaths	Male	2014	71.52872	79.28511	65.65497	
Luxembourg	Deaths	Male	2015	67.64903	74.45473	62.21951	
Luxembourg	Deaths	Male	2016	66.18059	72.47881	60.56972	
Luxembourg	Deaths	Male	2017	61.12034	66.93747	55.67497	
Luxembourg	Deaths	Male	2018	58.66753	64.1008	53.45122	
Luxembourg	Deaths	Male	2019	55.17536	59.88364	50.47503	
Luxembourg	Deaths	Male	2020	51.76123	56.64104	47.664	
Luxembourg	Deaths	Male	2021	51.01388	55.79172	46.55425	
Luxembourg	Deaths	Male	2022	47.73092	53.37947	43.31782	
Luxembourg	Deaths	Male	2023	50.27553	56.95389	44.97086	
Luxembourg	Deaths	Female	2010	29.46605	34.23528	25.36769	
Luxembourg	Deaths	Female	2011	30.26483	35.39584	26.19013	
Luxembourg	Deaths	Female	2012	31.45708	36.91474	27.17644	
Luxembourg	Deaths	Female	2013	30.40104	34.88516	26.31003	
Luxembourg	Deaths	Female	2014	31.848	36.71273	27.88633	
Luxembourg	Deaths	Female	2015	31.06253	35.92479	26.90013	
Luxembourg	Deaths	Female	2016	30.37466	35.05026	26.4546	

Luxembourg	Deaths	Female	2017	30.98865	35.9159	26.90091	
Luxembourg	Deaths	Female	2018	31.06215	35.97922	26.89852	
Luxembourg	Deaths	Female	2019	30.54524	34.97688	26.76908	
Luxembourg	Deaths	Female	2020	30.07566	34.5653	26.15181	
Luxembourg	Deaths	Female	2021	28.19652	32.0028	24.76887	
Luxembourg	Deaths	Female	2022	28.54924	32.80349	24.27775	
Luxembourg	Deaths	Female	2023	30.12613	34.8809	25.64849	
Luxembourg	DALYs	Male	2010	1652.223	1812.782	1522.724	
Luxembourg	DALYs	Male	2011	1606.424	1777.992	1468.667	
Luxembourg	DALYs	Male	2012	1560.603	1737.491	1436.503	
Luxembourg	DALYs	Male	2013	1532.398	1698.428	1395.455	
Luxembourg	DALYs	Male	2014	1576.078	1745.208	1444.004	
Luxembourg	DALYs	Male	2015	1508.425	1663.662	1388.006	
Luxembourg	DALYs	Male	2016	1482.464	1631.479	1356.889	
Luxembourg	DALYs	Male	2017	1349.543	1481.804	1231.367	
Luxembourg	DALYs	Male	2018	1285.266	1404.006	1165.718	
Luxembourg	DALYs	Male	2019	1208.424	1325.467	1099.035	
Luxembourg	DALYs	Male	2020	1125.825	1238.528	1023.088	
Luxembourg	DALYs	Male	2021	1101.569	1208.75	1002.008	
Luxembourg	DALYs	Male	2022	1030.371	1164.941	933.5588	
Luxembourg	DALYs	Male	2023	1092.515	1237.614	974.8509	
Luxembourg	DALYs	Female	2010	648.601	754.438	559.4139	
Luxembourg	DALYs	Female	2011	662.3088	777.3012	578.1323	
Luxembourg	DALYs	Female	2012	682.8348	807.3825	600.0521	
Luxembourg	DALYs	Female	2013	662.3456	770.9757	576.6746	
Luxembourg	DALYs	Female	2014	683.828	780.0651	603.8392	
Luxembourg	DALYs	Female	2015	655.5804	757.9666	572.4365	
Luxembourg	DALYs	Female	2016	650.6145	752.1409	571.0272	
Luxembourg	DALYs	Female	2017	657.5962	764.0916	572.5773	
Luxembourg	DALYs	Female	2018	646.6852	745.6925	560.4311	
Luxembourg	DALYs	Female	2019	635.1767	728.0286	558.2177	
Luxembourg	DALYs	Female	2020	625.0186	718.0843	542.798	
Luxembourg	DALYs	Female	2021	585.7915	667.3686	516.179	
Luxembourg	DALYs	Female	2022	594.3425	677.4266	506.8145	
Luxembourg	DALYs	Female	2023	626.7321	723.8359	537.6806	
Madagascar	Incidence	Male	2010	3.49041	5.314868	1.985937	

Madagascar	Incidence	Male	2011	3.518912	5.179885	2.018214	
Madagascar	Incidence	Male	2012	3.517907	5.053172	2.033362	
Madagascar	Incidence	Male	2013	3.536629	5.157041	2.008316	
Madagascar	Incidence	Male	2014	3.544351	5.197378	1.988569	
Madagascar	Incidence	Male	2015	3.59568	5.289096	2.02935	
Madagascar	Incidence	Male	2016	3.64769	5.315246	2.099884	
Madagascar	Incidence	Male	2017	3.649004	5.319894	2.108485	
Madagascar	Incidence	Male	2018	3.7388	5.485171	2.146188	
Madagascar	Incidence	Male	2019	3.805797	5.732967	2.118429	
Madagascar	Incidence	Male	2020	3.872501	5.836255	2.139102	
Madagascar	Incidence	Male	2021	4.340176	6.715593	2.400356	
Madagascar	Incidence	Male	2022	4.580902	6.976632	2.475664	
Madagascar	Incidence	Male	2023	4.684781	6.896701	2.555986	
Madagascar	Incidence	Female	2010	1.421699	2.066324	0.859301	
Madagascar	Incidence	Female	2011	1.424032	2.036948	0.868936	
Madagascar	Incidence	Female	2012	1.422979	2.032559	0.864846	
Madagascar	Incidence	Female	2013	1.421821	2.068523	0.870865	
Madagascar	Incidence	Female	2014	1.415712	2.048955	0.895297	
Madagascar	Incidence	Female	2015	1.436821	2.044756	0.911079	
Madagascar	Incidence	Female	2016	1.469393	2.082044	0.940161	
Madagascar	Incidence	Female	2017	1.50275	2.073729	0.977136	
Madagascar	Incidence	Female	2018	1.563723	2.107733	1.012979	
Madagascar	Incidence	Female	2019	1.614608	2.194318	1.042976	
Madagascar	Incidence	Female	2020	1.539561	2.075642	0.986369	
Madagascar	Incidence	Female	2021	1.685387	2.247505	1.092045	
Madagascar	Incidence	Female	2022	2.093582	2.891016	1.309758	
Madagascar	Incidence	Female	2023	2.241313	3.100258	1.366041	
Madagascar	Deaths	Male	2010	3.416668	5.174799	1.939321	
Madagascar	Deaths	Male	2011	3.442402	5.077618	1.974671	
Madagascar	Deaths	Male	2012	3.439357	4.937305	1.978733	
Madagascar	Deaths	Male	2013	3.455115	5.024029	1.960192	
Madagascar	Deaths	Male	2014	3.458769	5.071869	1.92808	
Madagascar	Deaths	Male	2015	3.507149	5.176597	1.977457	
Madagascar	Deaths	Male	2016	3.556078	5.188956	2.051353	
Madagascar	Deaths	Male	2017	3.556307	5.188436	2.05243	
Madagascar	Deaths	Male	2018	3.640962	5.34431	2.090905	

Madagascar	Deaths	Male	2019	3.702931	5.600453	2.06242	
Madagascar	Deaths	Male	2020	3.778323	5.681253	2.085075	
Madagascar	Deaths	Male	2021	4.227554	6.54832	2.341031	
Madagascar	Deaths	Male	2022	4.457302	6.807213	2.434262	
Madagascar	Deaths	Male	2023	4.544436	6.692967	2.484603	
Madagascar	Deaths	Female	2010	1.37075	1.993388	0.827694	
Madagascar	Deaths	Female	2011	1.373839	1.977552	0.836541	
Madagascar	Deaths	Female	2012	1.373541	1.983016	0.834262	
Madagascar	Deaths	Female	2013	1.372397	1.995462	0.841041	
Madagascar	Deaths	Female	2014	1.36563	1.978122	0.859345	
Madagascar	Deaths	Female	2015	1.385381	1.969342	0.874981	
Madagascar	Deaths	Female	2016	1.415547	2.012447	0.9021	
Madagascar	Deaths	Female	2017	1.44639	2.007699	0.94413	
Madagascar	Deaths	Female	2018	1.502646	2.031462	0.971773	
Madagascar	Deaths	Female	2019	1.548494	2.096516	0.997393	
Madagascar	Deaths	Female	2020	1.476114	1.994965	0.944517	
Madagascar	Deaths	Female	2021	1.609165	2.145051	1.038785	
Madagascar	Deaths	Female	2022	1.990398	2.767568	1.237942	
Madagascar	Deaths	Female	2023	2.123387	2.922654	1.280017	
Madagascar	DALYs	Male	2010	102.1341	153.8009	59.27753	
Madagascar	DALYs	Male	2011	103.1761	151.4327	59.81961	
Madagascar	DALYs	Male	2012	103.3396	148.3648	60.81627	
Madagascar	DALYs	Male	2013	104.1313	153.3928	59.84847	
Madagascar	DALYs	Male	2014	104.6168	154.1725	59.67817	
Madagascar	DALYs	Male	2015	106.1975	154.1062	60.75902	
Madagascar	DALYs	Male	2016	107.8295	157.6012	62.02059	
Madagascar	DALYs	Male	2017	107.8763	157.8215	62.68919	
Madagascar	DALYs	Male	2018	110.597	163.2038	63.41319	
Madagascar	DALYs	Male	2019	112.6128	169.6859	63.00172	
Madagascar	DALYs	Male	2020	113.9121	173.5579	63.1491	
Madagascar	DALYs	Male	2021	127.826	197.4469	70.40594	
Madagascar	DALYs	Male	2022	134.7097	203.0711	71.5129	
Madagascar	DALYs	Male	2023	138.3291	205.2373	75.95003	
Madagascar	DALYs	Female	2010	43.20598	62.86146	26.55972	
Madagascar	DALYs	Female	2011	43.22931	61.40447	26.86571	
Madagascar	DALYs	Female	2012	43.15339	61.30742	26.49544	

Madagascar	DALYs	Female	2013	43.12056	62.81799	26.42677	
Madagascar	DALYs	Female	2014	42.9783	62.23833	27.05369	
Madagascar	DALYs	Female	2015	43.61187	62.01541	27.79563	
Madagascar	DALYs	Female	2016	44.64026	63.01311	29.05013	
Madagascar	DALYs	Female	2017	45.69392	62.64621	29.68859	
Madagascar	DALYs	Female	2018	47.64531	64.24775	31.00048	
Madagascar	DALYs	Female	2019	49.31272	67.19118	31.8115	
Madagascar	DALYs	Female	2020	46.98059	63.09086	30.04824	
Madagascar	DALYs	Female	2021	51.74864	69.00386	33.74953	
Madagascar	DALYs	Female	2022	64.60245	88.90558	40.96406	
Madagascar	DALYs	Female	2023	69.63125	98.2685	43.02514	
Malawi	Incidence	Male	2010	1.492825	2.209517	1.061553	
Malawi	Incidence	Male	2011	1.546219	2.186672	1.092983	
Malawi	Incidence	Male	2012	1.56617	2.240741	1.118735	
Malawi	Incidence	Male	2013	1.562244	2.198292	1.102378	
Malawi	Incidence	Male	2014	1.580202	2.180299	1.114226	
Malawi	Incidence	Male	2015	1.605627	2.260635	1.119107	
Malawi	Incidence	Male	2016	1.629816	2.266809	1.134962	
Malawi	Incidence	Male	2017	1.651323	2.381649	1.131713	
Malawi	Incidence	Male	2018	1.691848	2.456201	1.137176	
Malawi	Incidence	Male	2019	1.743494	2.583237	1.183488	
Malawi	Incidence	Male	2020	1.821984	2.67948	1.232399	
Malawi	Incidence	Male	2021	2.774896	3.993363	1.826012	
Malawi	Incidence	Male	2022	2.032506	2.964182	1.325984	
Malawi	Incidence	Male	2023	2.055898	3.038737	1.377732	
Malawi	Incidence	Female	2010	0.513846	0.719879	0.34623	
Malawi	Incidence	Female	2011	0.543663	0.779123	0.35814	
Malawi	Incidence	Female	2012	0.562962	0.787626	0.359753	
Malawi	Incidence	Female	2013	0.568374	0.794531	0.354701	
Malawi	Incidence	Female	2014	0.577088	0.798277	0.382833	
Malawi	Incidence	Female	2015	0.590502	0.812092	0.373619	
Malawi	Incidence	Female	2016	0.603564	0.835396	0.368071	
Malawi	Incidence	Female	2017	0.625683	0.855632	0.399684	
Malawi	Incidence	Female	2018	0.655205	0.887526	0.434645	
Malawi	Incidence	Female	2019	0.687103	0.934013	0.460018	
Malawi	Incidence	Female	2020	0.688911	0.942535	0.462826	

Malawi	Incidence	Female	2021	0.851931	1.181649	0.561849	
Malawi	Incidence	Female	2022	0.861979	1.208761	0.556775	
Malawi	Incidence	Female	2023	0.926536	1.30163	0.609125	
Malawi	Deaths	Male	2010	1.44786	2.134213	1.037067	
Malawi	Deaths	Male	2011	1.497851	2.109054	1.064962	
Malawi	Deaths	Male	2012	1.516133	2.168533	1.086329	
Malawi	Deaths	Male	2013	1.511103	2.123768	1.066773	
Malawi	Deaths	Male	2014	1.526688	2.10359	1.083146	
Malawi	Deaths	Male	2015	1.550393	2.187948	1.095802	
Malawi	Deaths	Male	2016	1.572148	2.193203	1.113123	
Malawi	Deaths	Male	2017	1.591224	2.283924	1.104575	
Malawi	Deaths	Male	2018	1.627962	2.353678	1.103379	
Malawi	Deaths	Male	2019	1.675632	2.462688	1.152237	
Malawi	Deaths	Male	2020	1.753364	2.577716	1.197493	
Malawi	Deaths	Male	2021	2.700858	3.878884	1.791652	
Malawi	Deaths	Male	2022	1.951489	2.84466	1.285097	
Malawi	Deaths	Male	2023	1.968239	2.911577	1.318464	
Malawi	Deaths	Female	2010	0.488732	0.683189	0.331823	
Malawi	Deaths	Female	2011	0.515583	0.735692	0.341941	
Malawi	Deaths	Female	2012	0.532437	0.746829	0.34535	
Malawi	Deaths	Female	2013	0.5365	0.753717	0.338009	
Malawi	Deaths	Female	2014	0.543832	0.753636	0.364403	
Malawi	Deaths	Female	2015	0.555904	0.763367	0.355861	
Malawi	Deaths	Female	2016	0.56731	0.779577	0.351126	
Malawi	Deaths	Female	2017	0.587017	0.809205	0.379553	
Malawi	Deaths	Female	2018	0.613241	0.830427	0.4086	
Malawi	Deaths	Female	2019	0.641704	0.868504	0.431223	
Malawi	Deaths	Female	2020	0.640921	0.871954	0.433455	
Malawi	Deaths	Female	2021	0.786976	1.083648	0.525446	
Malawi	Deaths	Female	2022	0.797049	1.119007	0.517574	
Malawi	Deaths	Female	2023	0.855459	1.193837	0.565306	
Malawi	DALYs	Male	2010	44.04879	65.92848	31.04557	
Malawi	DALYs	Male	2011	45.74811	66.04177	32.08376	
Malawi	DALYs	Male	2012	46.42456	66.50537	32.48941	
Malawi	DALYs	Male	2013	46.40516	65.74007	32.31901	
Malawi	DALYs	Male	2014	47.0614	65.77961	32.50232	

Malawi	DALYs	Male	2015	47.8777	67.50276	32.53818	
Malawi	DALYs	Male	2016	48.71651	67.90353	32.78115	
Malawi	DALYs	Male	2017	49.48093	71.55146	33.00769	
Malawi	DALYs	Male	2018	50.8326	73.90413	33.46488	
Malawi	DALYs	Male	2019	52.47584	78.79743	34.86278	
Malawi	DALYs	Male	2020	54.64858	81.10302	36.33746	
Malawi	DALYs	Male	2021	81.05222	117.7683	53.12148	
Malawi	DALYs	Male	2022	60.98492	88.64098	39.10741	
Malawi	DALYs	Male	2023	61.92344	91.29467	41.06895	
Malawi	DALYs	Female	2010	16.45944	23.49283	11.09628	
Malawi	DALYs	Female	2011	17.48692	25.17702	11.43825	
Malawi	DALYs	Female	2012	18.18922	25.77767	11.20601	
Malawi	DALYs	Female	2013	18.42836	25.76922	11.22123	
Malawi	DALYs	Female	2014	18.75481	25.99091	11.79078	
Malawi	DALYs	Female	2015	19.21866	26.5805	11.81372	
Malawi	DALYs	Female	2016	19.69147	27.43061	11.9539	
Malawi	DALYs	Female	2017	20.47315	28.34191	12.56006	
Malawi	DALYs	Female	2018	21.51999	29.42867	13.80215	
Malawi	DALYs	Female	2019	22.63548	31.13151	14.83757	
Malawi	DALYs	Female	2020	22.83377	31.65211	15.07125	
Malawi	DALYs	Female	2021	28.37832	39.71625	18.79475	
Malawi	DALYs	Female	2022	28.74778	41.05755	18.19606	
Malawi	DALYs	Female	2023	30.97612	44.31637	19.9958	
Malaysia	Incidence	Male	2010	18.71729	22.12159	15.6616	
Malaysia	Incidence	Male	2011	18.85169	22.34163	15.64811	
Malaysia	Incidence	Male	2012	19.12366	22.56345	15.70581	
Malaysia	Incidence	Male	2013	19.55123	23.19195	15.81049	
Malaysia	Incidence	Male	2014	19.73675	23.20628	16.52034	
Malaysia	Incidence	Male	2015	19.79315	23.52858	16.86098	
Malaysia	Incidence	Male	2016	20.25789	24.32027	16.95271	
Malaysia	Incidence	Male	2017	20.58695	24.89087	17.35059	
Malaysia	Incidence	Male	2018	20.97181	25.66611	17.77592	
Malaysia	Incidence	Male	2019	21.12492	26.54253	18.23189	
Malaysia	Incidence	Male	2020	21.11854	26.53751	18.1429	
Malaysia	Incidence	Male	2021	21.70205	27.25821	18.63626	
Malaysia	Incidence	Male	2022	24.83145	30.54913	21.09326	

Malaysia	Incidence	Male	2023	24.1107	30.03837	20.10458	
Malaysia	Incidence	Female	2010	9.348761	11.45716	7.737068	
Malaysia	Incidence	Female	2011	9.405751	11.59549	7.891738	
Malaysia	Incidence	Female	2012	9.54825	11.54828	7.876103	
Malaysia	Incidence	Female	2013	9.880248	11.74953	8.202345	
Malaysia	Incidence	Female	2014	10.0174	11.75409	8.262985	
Malaysia	Incidence	Female	2015	10.22441	11.91812	8.469382	
Malaysia	Incidence	Female	2016	10.47228	12.36548	8.607823	
Malaysia	Incidence	Female	2017	10.66943	12.56057	8.890202	
Malaysia	Incidence	Female	2018	10.87662	13.13075	9.124512	
Malaysia	Incidence	Female	2019	10.78178	13.12383	9.097714	
Malaysia	Incidence	Female	2020	10.81484	13.49664	9.108135	
Malaysia	Incidence	Female	2021	12.05125	15.01906	9.896086	
Malaysia	Incidence	Female	2022	13.33637	16.37206	10.4919	
Malaysia	Incidence	Female	2023	13.46709	16.96426	10.37059	
Malaysia	Deaths	Male	2010	18.62286	22.15054	15.519	
Malaysia	Deaths	Male	2011	18.74735	22.17785	15.57096	
Malaysia	Deaths	Male	2012	19.00425	22.2577	15.52576	
Malaysia	Deaths	Male	2013	19.43124	23.09937	15.64996	
Malaysia	Deaths	Male	2014	19.63272	23.14802	16.26994	
Malaysia	Deaths	Male	2015	19.71312	23.58796	16.70474	
Malaysia	Deaths	Male	2016	20.192	24.27129	16.91814	
Malaysia	Deaths	Male	2017	20.52124	24.89803	17.18429	
Malaysia	Deaths	Male	2018	20.94402	25.76135	17.60706	
Malaysia	Deaths	Male	2019	21.13368	26.79488	18.1659	
Malaysia	Deaths	Male	2020	21.11659	26.83116	18.24396	
Malaysia	Deaths	Male	2021	21.73253	27.43968	18.87497	
Malaysia	Deaths	Male	2022	24.93359	30.57863	21.08272	
Malaysia	Deaths	Male	2023	24.2077	30.54639	20.36231	
Malaysia	Deaths	Female	2010	9.295858	11.35219	7.692241	
Malaysia	Deaths	Female	2011	9.346854	11.61915	7.807409	
Malaysia	Deaths	Female	2012	9.472957	11.56973	7.808043	
Malaysia	Deaths	Female	2013	9.791685	11.6059	8.195105	
Malaysia	Deaths	Female	2014	9.923172	11.67932	8.230236	
Malaysia	Deaths	Female	2015	10.11902	11.67179	8.418376	
Malaysia	Deaths	Female	2016	10.36238	12.06713	8.589929	

Malaysia	Deaths	Female	2017	10.55956	12.33535	8.854537	
Malaysia	Deaths	Female	2018	10.76097	12.84317	9.037914	
Malaysia	Deaths	Female	2019	10.65371	12.76075	9.027895	
Malaysia	Deaths	Female	2020	10.65898	13.1107	9.053469	
Malaysia	Deaths	Female	2021	11.84823	14.59208	9.767876	
Malaysia	Deaths	Female	2022	13.18098	16.31712	10.38361	
Malaysia	Deaths	Female	2023	13.29357	16.86724	10.27953	
Malaysia	DALYs	Male	2010	489.8592	590.9985	415.2711	
Malaysia	DALYs	Male	2011	493.978	587.946	413.9611	
Malaysia	DALYs	Male	2012	500.534	596.9178	422.8853	
Malaysia	DALYs	Male	2013	510.6127	607.6291	420.7574	
Malaysia	DALYs	Male	2014	514.4688	609.1243	434.8869	
Malaysia	DALYs	Male	2015	514.1219	614.6747	437.8037	
Malaysia	DALYs	Male	2016	524.8759	630.5207	442.8718	
Malaysia	DALYs	Male	2017	531.9283	644.043	449.6196	
Malaysia	DALYs	Male	2018	538.8598	663.9783	457.9772	
Malaysia	DALYs	Male	2019	539.3788	680.0703	461.0214	
Malaysia	DALYs	Male	2020	537.4833	674.7248	460.5614	
Malaysia	DALYs	Male	2021	551.0486	690.4028	475.0882	
Malaysia	DALYs	Male	2022	623.3842	767.8545	525.786	
Malaysia	DALYs	Male	2023	604.518	760.1931	499.7017	
Malaysia	DALYs	Female	2010	234.8446	286.5655	196.5325	
Malaysia	DALYs	Female	2011	236.5033	291.4782	199.0635	
Malaysia	DALYs	Female	2012	239.7748	289.9989	199.2053	
Malaysia	DALYs	Female	2013	247.735	295.6261	204.0163	
Malaysia	DALYs	Female	2014	251.4849	292.4852	209.287	
Malaysia	DALYs	Female	2015	257.1046	297.5973	218.2522	
Malaysia	DALYs	Female	2016	263.273	311.2293	218.3839	
Malaysia	DALYs	Female	2017	267.2278	317.852	225.3165	
Malaysia	DALYs	Female	2018	271.2708	331.6973	227.898	
Malaysia	DALYs	Female	2019	267.6693	330.9108	227.3412	
Malaysia	DALYs	Female	2020	268.3076	331.7603	225.3469	
Malaysia	DALYs	Female	2021	299.4096	369.1196	244.9285	
Malaysia	DALYs	Female	2022	324.9954	397.4545	257.6685	
Malaysia	DALYs	Female	2023	327.5441	418.8639	253.5867	
Maldives	Incidence	Male	2010	10.75059	13.63871	7.989387	

Maldives	Incidence	Male	2011	10.61348	13.45796	8.006371	
Maldives	Incidence	Male	2012	10.37795	13.17825	7.924557	
Maldives	Incidence	Male	2013	10.21997	12.78368	7.908122	
Maldives	Incidence	Male	2014	9.987667	12.33679	7.793046	
Maldives	Incidence	Male	2015	9.76747	12.08524	7.571598	
Maldives	Incidence	Male	2016	9.67941	12.00948	7.436826	
Maldives	Incidence	Male	2017	9.672242	12.16543	7.552678	
Maldives	Incidence	Male	2018	9.713782	12.35585	7.608416	
Maldives	Incidence	Male	2019	9.915884	12.96278	7.538782	
Maldives	Incidence	Male	2020	9.809341	12.95997	7.43167	
Maldives	Incidence	Male	2021	9.552642	12.73835	7.223891	
Maldives	Incidence	Male	2022	10.14173	13.34183	7.565802	
Maldives	Incidence	Male	2023	10.59088	14.27507	7.769969	
Maldives	Incidence	Female	2010	4.648261	6.173182	3.431283	
Maldives	Incidence	Female	2011	4.773031	6.321909	3.532781	
Maldives	Incidence	Female	2012	4.838261	6.375474	3.49858	
Maldives	Incidence	Female	2013	4.894488	6.479605	3.568917	
Maldives	Incidence	Female	2014	4.925877	6.462134	3.510062	
Maldives	Incidence	Female	2015	5.028202	6.641011	3.572606	
Maldives	Incidence	Female	2016	5.15555	6.623115	3.67417	
Maldives	Incidence	Female	2017	5.33428	6.822641	3.88483	
Maldives	Incidence	Female	2018	5.567684	7.014175	4.105861	
Maldives	Incidence	Female	2019	5.895483	7.477939	4.399658	
Maldives	Incidence	Female	2020	6.049425	7.853336	4.494547	
Maldives	Incidence	Female	2021	6.354391	8.344549	4.702126	
Maldives	Incidence	Female	2022	6.603695	8.706796	4.85345	
Maldives	Incidence	Female	2023	6.843007	8.934276	4.997266	
Maldives	Deaths	Male	2010	11.08277	13.92464	8.092073	
Maldives	Deaths	Male	2011	10.9463	13.87458	8.140971	
Maldives	Deaths	Male	2012	10.69757	13.59994	8.119712	
Maldives	Deaths	Male	2013	10.51806	13.28761	8.020265	
Maldives	Deaths	Male	2014	10.25765	12.79848	7.885239	
Maldives	Deaths	Male	2015	10.01533	12.44401	7.697956	
Maldives	Deaths	Male	2016	9.907822	12.28769	7.620925	
Maldives	Deaths	Male	2017	9.874102	12.37296	7.707911	
Maldives	Deaths	Male	2018	9.887729	12.66243	7.737097	

Maldives	Deaths	Male	2019	10.04729	13.17529	7.663892	
Maldives	Deaths	Male	2020	9.930915	13.21626	7.395484	
Maldives	Deaths	Male	2021	9.741646	12.96241	7.261588	
Maldives	Deaths	Male	2022	10.28327	13.46013	7.562411	
Maldives	Deaths	Male	2023	10.73363	14.19908	7.897686	
Maldives	Deaths	Female	2010	4.551762	6.068859	3.38473	
Maldives	Deaths	Female	2011	4.677339	6.178907	3.435853	
Maldives	Deaths	Female	2012	4.741857	6.294238	3.454145	
Maldives	Deaths	Female	2013	4.796954	6.368272	3.503049	
Maldives	Deaths	Female	2014	4.825648	6.33124	3.47791	
Maldives	Deaths	Female	2015	4.923603	6.47846	3.537686	
Maldives	Deaths	Female	2016	5.045101	6.509372	3.623136	
Maldives	Deaths	Female	2017	5.212713	6.626214	3.803641	
Maldives	Deaths	Female	2018	5.425801	6.812751	3.999119	
Maldives	Deaths	Female	2019	5.716572	7.173654	4.242221	
Maldives	Deaths	Female	2020	5.854135	7.605933	4.41852	
Maldives	Deaths	Female	2021	6.170991	8.076419	4.568311	
Maldives	Deaths	Female	2022	6.41959	8.397424	4.769891	
Maldives	Deaths	Female	2023	6.678047	8.714332	4.899991	
Maldives	DALYs	Male	2010	252.8603	318.0649	194.2841	
Maldives	DALYs	Male	2011	249.3768	309.9652	192.5417	
Maldives	DALYs	Male	2012	244.1897	304.5492	190.3882	
Maldives	DALYs	Male	2013	241.0806	297.0209	189.4232	
Maldives	DALYs	Male	2014	236.2127	289.1909	186.646	
Maldives	DALYs	Male	2015	231.2231	287.6428	179.2299	
Maldives	DALYs	Male	2016	229.2987	285.8804	176.9157	
Maldives	DALYs	Male	2017	229.3926	288.1308	178.612	
Maldives	DALYs	Male	2018	230.2288	293.3102	180.462	
Maldives	DALYs	Male	2019	234.676	305.597	179.8657	
Maldives	DALYs	Male	2020	230.3415	305.2304	172.9251	
Maldives	DALYs	Male	2021	218.9992	290.0079	167.5426	
Maldives	DALYs	Male	2022	234.2316	308.5111	176.4576	
Maldives	DALYs	Male	2023	243.9227	323.4805	182.4412	
Maldives	DALYs	Female	2010	113.2325	150.4109	84.63521	
Maldives	DALYs	Female	2011	115.3788	154.2259	84.78783	
Maldives	DALYs	Female	2012	116.2744	153.7381	84.17368	

Maldives	DALYs	Female	2013	116.9874	155.7224	84.62154	
Maldives	DALYs	Female	2014	117.1457	153.4322	83.73734	
Maldives	DALYs	Female	2015	118.7824	154.8195	84.71009	
Maldives	DALYs	Female	2016	120.9868	155.1757	86.36163	
Maldives	DALYs	Female	2017	124.3306	157.9041	90.03275	
Maldives	DALYs	Female	2018	128.6826	161.8182	94.9911	
Maldives	DALYs	Female	2019	134.7114	169.6812	101.0847	
Maldives	DALYs	Female	2020	136.3629	172.2743	102.1088	
Maldives	DALYs	Female	2021	139.8458	180.8168	104.3348	
Maldives	DALYs	Female	2022	143.8957	183.8532	107.7985	
Maldives	DALYs	Female	2023	146.6454	190.8146	107.3943	
Mali	Incidence	Male	2010	2.914745	4.109188	2.152892	
Mali	Incidence	Male	2011	2.831672	3.874181	2.140151	
Mali	Incidence	Male	2012	2.770561	3.826513	2.09429	
Mali	Incidence	Male	2013	2.665733	3.68543	2.003714	
Mali	Incidence	Male	2014	2.72125	3.787431	2.000856	
Mali	Incidence	Male	2015	2.9762	4.115148	2.160768	
Mali	Incidence	Male	2016	3.000135	4.070844	2.133701	
Mali	Incidence	Male	2017	3.112757	4.297338	2.207737	
Mali	Incidence	Male	2018	3.146021	4.390953	2.162057	
Mali	Incidence	Male	2019	3.111845	4.531305	2.098426	
Mali	Incidence	Male	2020	3.072725	4.407355	2.085044	
Mali	Incidence	Male	2021	3.243986	4.677841	2.17129	
Mali	Incidence	Male	2022	3.428198	4.920762	2.267657	
Mali	Incidence	Male	2023	3.585384	5.076238	2.522446	
Mali	Incidence	Female	2010	0.936688	1.339861	0.644974	
Mali	Incidence	Female	2011	0.942411	1.336284	0.662732	
Mali	Incidence	Female	2012	0.927644	1.34296	0.638035	
Mali	Incidence	Female	2013	0.92004	1.301846	0.621729	
Mali	Incidence	Female	2014	0.934897	1.328219	0.611554	
Mali	Incidence	Female	2015	1.007009	1.390901	0.674259	
Mali	Incidence	Female	2016	1.045565	1.428897	0.711037	
Mali	Incidence	Female	2017	1.097683	1.495001	0.762972	
Mali	Incidence	Female	2018	1.131778	1.548145	0.801678	
Mali	Incidence	Female	2019	1.146196	1.580795	0.803786	
Mali	Incidence	Female	2020	1.099022	1.458407	0.781564	

Mali	Incidence	Female	2021	1.171516	1.580905	0.834239	
Mali	Incidence	Female	2022	1.355855	1.774075	0.944113	
Mali	Incidence	Female	2023	1.438416	1.967468	0.993573	
Mali	Deaths	Male	2010	2.875901	4.060402	2.120452	
Mali	Deaths	Male	2011	2.797937	3.815143	2.109714	
Mali	Deaths	Male	2012	2.741848	3.774517	2.063947	
Mali	Deaths	Male	2013	2.643808	3.689023	1.990454	
Mali	Deaths	Male	2014	2.699687	3.761115	1.978181	
Mali	Deaths	Male	2015	2.953053	4.093836	2.150818	
Mali	Deaths	Male	2016	2.975291	4.058032	2.105222	
Mali	Deaths	Male	2017	3.087089	4.26394	2.179855	
Mali	Deaths	Male	2018	3.120513	4.348439	2.14251	
Mali	Deaths	Male	2019	3.08766	4.51355	2.076161	
Mali	Deaths	Male	2020	3.048305	4.404769	2.063769	
Mali	Deaths	Male	2021	3.209508	4.62912	2.154733	
Mali	Deaths	Male	2022	3.398477	4.893139	2.230856	
Mali	Deaths	Male	2023	3.551676	5.010887	2.495939	
Mali	Deaths	Female	2010	0.899677	1.282379	0.620624	
Mali	Deaths	Female	2011	0.904339	1.283896	0.636521	
Mali	Deaths	Female	2012	0.889238	1.2891	0.612627	
Mali	Deaths	Female	2013	0.883165	1.253232	0.595004	
Mali	Deaths	Female	2014	0.896621	1.27306	0.591535	
Mali	Deaths	Female	2015	0.964436	1.332795	0.644744	
Mali	Deaths	Female	2016	0.999926	1.368233	0.680595	
Mali	Deaths	Female	2017	1.0487	1.432315	0.732274	
Mali	Deaths	Female	2018	1.079665	1.48199	0.765378	
Mali	Deaths	Female	2019	1.092352	1.504342	0.76494	
Mali	Deaths	Female	2020	1.044521	1.387957	0.7515	
Mali	Deaths	Female	2021	1.109423	1.49425	0.785118	
Mali	Deaths	Female	2022	1.283842	1.676439	0.893242	
Mali	Deaths	Female	2023	1.360469	1.861539	0.940931	
Mali	DALYs	Male	2010	82.35769	115.3697	61.54288	
Mali	DALYs	Male	2011	79.78591	109.6028	60.52376	
Mali	DALYs	Male	2012	77.89386	108.1744	59.33315	
Mali	DALYs	Male	2013	74.52711	102.353	56.04241	
Mali	DALYs	Male	2014	76.01298	105.3544	56.1596	

Mali	DALYs	Male	2015	83.02204	114.3582	60.53436	
Mali	DALYs	Male	2016	83.7175	112.6405	60.17368	
Mali	DALYs	Male	2017	86.83174	120.0788	61.68124	
Mali	DALYs	Male	2018	87.71003	124.9507	60.48366	
Mali	DALYs	Male	2019	86.69322	125.2985	58.72147	
Mali	DALYs	Male	2020	85.67454	123.0704	58.30544	
Mali	DALYs	Male	2021	90.89049	131.2844	60.34455	
Mali	DALYs	Male	2022	95.46476	136.3117	63.49051	
Mali	DALYs	Male	2023	99.86558	142.4504	70.04014	
Mali	DALYs	Female	2010	28.58896	41.08651	19.67829	
Mali	DALYs	Female	2011	28.86402	40.95546	20.509	
Mali	DALYs	Female	2012	28.53921	41.18005	19.60676	
Mali	DALYs	Female	2013	28.22759	39.95904	18.89067	
Mali	DALYs	Female	2014	28.75168	40.75441	18.80769	
Mali	DALYs	Female	2015	31.04561	42.84317	20.75318	
Mali	DALYs	Female	2016	32.31537	44.31652	21.48434	
Mali	DALYs	Female	2017	33.97646	46.34442	23.42013	
Mali	DALYs	Female	2018	35.13932	48.00899	24.85547	
Mali	DALYs	Female	2019	35.66918	49.11794	25.17548	
Mali	DALYs	Female	2020	34.37679	45.64053	24.6036	
Mali	DALYs	Female	2021	36.87325	49.83658	26.22331	
Mali	DALYs	Female	2022	42.55265	56.2373	29.59666	
Mali	DALYs	Female	2023	45.20782	61.66773	31.4837	
Malta	Incidence	Male	2010	68.31661	76.01547	61.55758	
Malta	Incidence	Male	2011	67.24204	74.78931	60.72689	
Malta	Incidence	Male	2012	67.42621	74.99946	60.67871	
Malta	Incidence	Male	2013	64.59755	72.16773	58.09026	
Malta	Incidence	Male	2014	66.5116	74.37355	60.11682	
Malta	Incidence	Male	2015	66.50304	74.89455	59.71636	
Malta	Incidence	Male	2016	63.08153	71.4357	56.39478	
Malta	Incidence	Male	2017	61.48728	69.23356	54.37387	
Malta	Incidence	Male	2018	61.13275	68.55321	54.38104	
Malta	Incidence	Male	2019	57.12219	64.43548	50.40725	
Malta	Incidence	Male	2020	55.43245	62.02192	48.71187	
Malta	Incidence	Male	2021	52.26227	58.8178	46.03144	
Malta	Incidence	Male	2022	51.62484	58.10658	45.2447	

Malta	Incidence	Male	2023	51.40245	60.17635	43.46314	
Malta	Incidence	Female	2010	17.50266	21.24883	14.60734	
Malta	Incidence	Female	2011	17.5783	21.04858	14.81998	
Malta	Incidence	Female	2012	18.3404	22.18286	15.38328	
Malta	Incidence	Female	2013	18.41209	22.21141	15.45634	
Malta	Incidence	Female	2014	18.42189	22.09466	15.41762	
Malta	Incidence	Female	2015	19.4797	23.36908	15.93373	
Malta	Incidence	Female	2016	20.29706	24.54655	16.65615	
Malta	Incidence	Female	2017	20.44172	24.5187	16.7898	
Malta	Incidence	Female	2018	20.32606	24.80845	16.71418	
Malta	Incidence	Female	2019	19.53207	23.22089	16.10892	
Malta	Incidence	Female	2020	18.951	22.41053	15.73847	
Malta	Incidence	Female	2021	20.16605	24.36464	16.78304	
Malta	Incidence	Female	2022	20.11715	24.39085	16.74049	
Malta	Incidence	Female	2023	19.58101	23.43536	15.86314	
Malta	Deaths	Male	2010	62.7388	69.15964	57.47476	
Malta	Deaths	Male	2011	61.97579	67.59691	56.48222	
Malta	Deaths	Male	2012	62.4214	67.99153	56.51213	
Malta	Deaths	Male	2013	59.80624	65.53591	53.81029	
Malta	Deaths	Male	2014	61.49063	68.02302	55.57918	
Malta	Deaths	Male	2015	61.39813	68.41884	55.49997	
Malta	Deaths	Male	2016	58.23201	65.11443	52.67901	
Malta	Deaths	Male	2017	56.86323	63.94106	51.50479	
Malta	Deaths	Male	2018	56.64392	63.46997	50.55782	
Malta	Deaths	Male	2019	52.97185	59.55792	47.23585	
Malta	Deaths	Male	2020	51.80823	57.6834	46.62522	
Malta	Deaths	Male	2021	48.83062	54.52121	43.4495	
Malta	Deaths	Male	2022	48.36431	54.44464	42.76498	
Malta	Deaths	Male	2023	48.34012	56.02316	41.55446	
Malta	Deaths	Female	2010	15.5497	18.71313	13.30484	
Malta	Deaths	Female	2011	15.60042	18.78289	13.37891	
Malta	Deaths	Female	2012	16.24758	19.41433	13.99538	
Malta	Deaths	Female	2013	16.25532	19.48771	13.86285	
Malta	Deaths	Female	2014	16.19397	19.28075	13.68839	
Malta	Deaths	Female	2015	17.01661	20.4666	14.28645	
Malta	Deaths	Female	2016	17.6754	21.00901	14.86717	

Malta	Deaths	Female	2017	17.65411	20.94472	14.70421	
Malta	Deaths	Female	2018	17.57195	21.0735	14.80743	
Malta	Deaths	Female	2019	16.88231	20.15625	14.33135	
Malta	Deaths	Female	2020	16.44139	19.47194	13.97456	
Malta	Deaths	Female	2021	17.50226	20.74811	14.89583	
Malta	Deaths	Female	2022	17.4622	20.75332	14.49969	
Malta	Deaths	Female	2023	17.02211	20.16386	14.08265	
Malta	DALYs	Male	2010	1439.995	1574.971	1315.329	
Malta	DALYs	Male	2011	1396.677	1522.632	1278.12	
Malta	DALYs	Male	2012	1384.574	1509.756	1258.052	
Malta	DALYs	Male	2013	1322.57	1442.337	1207.628	
Malta	DALYs	Male	2014	1364.335	1505.385	1239.903	
Malta	DALYs	Male	2015	1360.606	1508.087	1229.368	
Malta	DALYs	Male	2016	1287.898	1430.477	1167.496	
Malta	DALYs	Male	2017	1247.287	1392.941	1129.474	
Malta	DALYs	Male	2018	1225.351	1374.885	1100.125	
Malta	DALYs	Male	2019	1139.081	1272.988	1018.869	
Malta	DALYs	Male	2020	1081.681	1203.834	976.8429	
Malta	DALYs	Male	2021	1020.806	1134.934	904.8156	
Malta	DALYs	Male	2022	1003.974	1128.262	885.4505	
Malta	DALYs	Male	2023	991.56	1142.803	859.1448	
Malta	DALYs	Female	2010	353.9572	422.4876	303.025	
Malta	DALYs	Female	2011	353.3419	421.8536	306.0195	
Malta	DALYs	Female	2012	364.686	434.633	313.4446	
Malta	DALYs	Female	2013	364.3523	435.7825	313.6952	
Malta	DALYs	Female	2014	361.4384	427.9101	306.3199	
Malta	DALYs	Female	2015	378.8516	450.7317	321.3912	
Malta	DALYs	Female	2016	390.2841	461.5758	331.8691	
Malta	DALYs	Female	2017	392.2289	464.215	332.4448	
Malta	DALYs	Female	2018	385.0485	461.1213	328.6162	
Malta	DALYs	Female	2019	366.5882	433.3707	313.4228	
Malta	DALYs	Female	2020	351.7666	415.6956	301.7564	
Malta	DALYs	Female	2021	369.8833	439.1948	315.6784	
Malta	DALYs	Female	2022	368.2641	440.4085	308.4183	
Malta	DALYs	Female	2023	357.1344	421.197	300.0337	
Mauritania	Incidence	Male	2010	6.075546	8.65051	3.863672	

Mauritania	Incidence	Male	2011	5.972401	8.249588	3.882375	
Mauritania	Incidence	Male	2012	6.228743	8.743149	4.131443	
Mauritania	Incidence	Male	2013	5.999996	8.33528	4.144958	
Mauritania	Incidence	Male	2014	5.780836	7.924345	3.981379	
Mauritania	Incidence	Male	2015	6.027029	8.359228	4.011924	
Mauritania	Incidence	Male	2016	5.806919	8.188654	3.798989	
Mauritania	Incidence	Male	2017	6.145892	8.657001	3.92038	
Mauritania	Incidence	Male	2018	6.076168	8.749001	4.013251	
Mauritania	Incidence	Male	2019	6.390792	9.411901	4.137393	
Mauritania	Incidence	Male	2020	6.611899	9.79212	4.35698	
Mauritania	Incidence	Male	2021	7.049896	10.25734	4.591292	
Mauritania	Incidence	Male	2022	7.47072	11.04314	4.696891	
Mauritania	Incidence	Male	2023	7.415386	10.87497	4.756632	
Mauritania	Incidence	Female	2010	1.807126	2.582121	1.214208	
Mauritania	Incidence	Female	2011	1.804242	2.549076	1.221146	
Mauritania	Incidence	Female	2012	1.909983	2.705027	1.269961	
Mauritania	Incidence	Female	2013	1.925478	2.753158	1.311965	
Mauritania	Incidence	Female	2014	1.854629	2.692669	1.250322	
Mauritania	Incidence	Female	2015	1.890934	2.730666	1.242532	
Mauritania	Incidence	Female	2016	1.851537	2.596691	1.233927	
Mauritania	Incidence	Female	2017	2.020079	2.838428	1.341931	
Mauritania	Incidence	Female	2018	2.045407	2.814398	1.396082	
Mauritania	Incidence	Female	2019	2.246753	3.10836	1.523387	
Mauritania	Incidence	Female	2020	2.124612	2.87435	1.453111	
Mauritania	Incidence	Female	2021	2.343154	3.202992	1.636106	
Mauritania	Incidence	Female	2022	2.791207	3.975591	1.955675	
Mauritania	Incidence	Female	2023	2.994936	4.18945	2.002316	
Mauritania	Deaths	Male	2010	6.110823	8.680308	3.870272	
Mauritania	Deaths	Male	2011	6.007179	8.316077	3.895234	
Mauritania	Deaths	Male	2012	6.259834	8.799057	4.15471	
Mauritania	Deaths	Male	2013	6.033964	8.400844	4.177551	
Mauritania	Deaths	Male	2014	5.809058	7.99326	4.006221	
Mauritania	Deaths	Male	2015	6.051733	8.421709	4.028313	
Mauritania	Deaths	Male	2016	5.825646	8.20718	3.798754	
Mauritania	Deaths	Male	2017	6.161566	8.660485	3.90521	
Mauritania	Deaths	Male	2018	6.095673	8.774216	4.029803	

Mauritania	Deaths	Male	2019	6.40722	9.472771	4.155309	
Mauritania	Deaths	Male	2020	6.650295	9.837805	4.368757	
Mauritania	Deaths	Male	2021	7.065433	10.26318	4.577935	
Mauritania	Deaths	Male	2022	7.48263	11.02387	4.712615	
Mauritania	Deaths	Male	2023	7.410867	10.92849	4.72348	
Mauritania	Deaths	Female	2010	1.772076	2.527416	1.182088	
Mauritania	Deaths	Female	2011	1.769914	2.492169	1.191153	
Mauritania	Deaths	Female	2012	1.872218	2.650589	1.244071	
Mauritania	Deaths	Female	2013	1.888157	2.690355	1.283655	
Mauritania	Deaths	Female	2014	1.816965	2.618352	1.224275	
Mauritania	Deaths	Female	2015	1.8504	2.653858	1.22029	
Mauritania	Deaths	Female	2016	1.810129	2.528311	1.204616	
Mauritania	Deaths	Female	2017	1.972305	2.770331	1.311471	
Mauritania	Deaths	Female	2018	1.995538	2.742616	1.356711	
Mauritania	Deaths	Female	2019	2.189869	3.042316	1.476625	
Mauritania	Deaths	Female	2020	2.074013	2.813434	1.405335	
Mauritania	Deaths	Female	2021	2.278176	3.124625	1.604358	
Mauritania	Deaths	Female	2022	2.703485	3.844317	1.883621	
Mauritania	Deaths	Female	2023	2.894711	4.055347	1.926526	
Mauritania	DALYs	Male	2010	161.1753	230.5594	103.1362	
Mauritania	DALYs	Male	2011	158.5207	217.846	102.6795	
Mauritania	DALYs	Male	2012	165.5944	232.1174	109.5508	
Mauritania	DALYs	Male	2013	159.2466	219.9956	109.5917	
Mauritania	DALYs	Male	2014	153.4448	210.2616	104.5868	
Mauritania	DALYs	Male	2015	159.9261	222.5189	106.8716	
Mauritania	DALYs	Male	2016	154.1599	217.7077	100.6068	
Mauritania	DALYs	Male	2017	163.0803	231.7101	104.2576	
Mauritania	DALYs	Male	2018	161.2047	232.2492	106.3452	
Mauritania	DALYs	Male	2019	169.3969	247.8034	109.0468	
Mauritania	DALYs	Male	2020	173.4707	253.9322	115.5941	
Mauritania	DALYs	Male	2021	185.6847	270.8314	120.2399	
Mauritania	DALYs	Male	2022	196.5919	286.0843	122.9582	
Mauritania	DALYs	Male	2023	196.1616	288.3072	126.372	
Mauritania	DALYs	Female	2010	52.08599	74.74984	35.33902	
Mauritania	DALYs	Female	2011	51.92198	72.75309	35.46404	
Mauritania	DALYs	Female	2012	54.92885	78.11493	36.75989	

Mauritania	DALYs	Female	2013	55.19736	79.15597	36.99512	
Mauritania	DALYs	Female	2014	53.14918	77.21625	35.87123	
Mauritania	DALYs	Female	2015	54.15782	77.50627	35.45472	
Mauritania	DALYs	Female	2016	53.00938	73.54977	35.13824	
Mauritania	DALYs	Female	2017	57.8132	80.30132	38.49737	
Mauritania	DALYs	Female	2018	58.59521	80.42906	40.38573	
Mauritania	DALYs	Female	2019	64.27381	87.93533	44.19891	
Mauritania	DALYs	Female	2020	60.55226	81.97451	42.16069	
Mauritania	DALYs	Female	2021	67.02773	91.625	46.73989	
Mauritania	DALYs	Female	2022	80.09788	114.2813	56.23142	
Mauritania	DALYs	Female	2023	86.1251	121.505	57.82257	
Mauritius	Incidence	Male	2010	17.56296	19.49603	15.74812	
Mauritius	Incidence	Male	2011	17.93912	20.10424	15.96805	
Mauritius	Incidence	Male	2012	18.58651	20.84096	16.65967	
Mauritius	Incidence	Male	2013	19.43209	21.79241	17.47321	
Mauritius	Incidence	Male	2014	19.18562	21.77847	17.24402	
Mauritius	Incidence	Male	2015	19.043	21.33392	17.2055	
Mauritius	Incidence	Male	2016	19.47288	21.66736	17.5398	
Mauritius	Incidence	Male	2017	19.10364	21.24926	17.02517	
Mauritius	Incidence	Male	2018	18.71427	21.09761	16.64752	
Mauritius	Incidence	Male	2019	19.20095	21.89331	17.02333	
Mauritius	Incidence	Male	2020	18.1585	20.94842	16.07876	
Mauritius	Incidence	Male	2021	18.78096	21.22332	16.8146	
Mauritius	Incidence	Male	2022	18.99532	21.34157	16.74288	
Mauritius	Incidence	Male	2023	20.90606	23.74734	18.3906	
Mauritius	Incidence	Female	2010	6.629163	7.845779	5.620054	
Mauritius	Incidence	Female	2011	6.857868	8.165839	5.902232	
Mauritius	Incidence	Female	2012	6.956539	8.258209	5.958535	
Mauritius	Incidence	Female	2013	7.148101	8.481036	6.083566	
Mauritius	Incidence	Female	2014	6.968184	8.247802	5.951665	
Mauritius	Incidence	Female	2015	7.332062	8.63023	6.316477	
Mauritius	Incidence	Female	2016	7.264835	8.697879	6.246498	
Mauritius	Incidence	Female	2017	7.495326	8.88984	6.385722	
Mauritius	Incidence	Female	2018	8.069708	9.64675	6.844255	
Mauritius	Incidence	Female	2019	7.92939	9.374701	6.656188	
Mauritius	Incidence	Female	2020	7.809442	9.086553	6.629172	

Mauritius	Incidence	Female	2021	7.710362	8.891823	6.547019	
Mauritius	Incidence	Female	2022	8.470862	9.785304	7.323841	
Mauritius	Incidence	Female	2023	8.93704	10.45323	7.662227	
Mauritius	Deaths	Male	2010	17.45685	19.35853	15.6893	
Mauritius	Deaths	Male	2011	17.84785	19.95334	15.99156	
Mauritius	Deaths	Male	2012	18.48057	20.72047	16.59803	
Mauritius	Deaths	Male	2013	19.22586	21.45634	17.31833	
Mauritius	Deaths	Male	2014	19.01443	21.43298	17.12887	
Mauritius	Deaths	Male	2015	18.88996	21.11118	17.07359	
Mauritius	Deaths	Male	2016	19.34845	21.43189	17.40944	
Mauritius	Deaths	Male	2017	18.99923	21.15846	16.90691	
Mauritius	Deaths	Male	2018	18.59641	20.76841	16.57776	
Mauritius	Deaths	Male	2019	19.01775	21.4771	16.98246	
Mauritius	Deaths	Male	2020	17.98377	20.48929	16.06962	
Mauritius	Deaths	Male	2021	18.69955	21.15768	16.83663	
Mauritius	Deaths	Male	2022	18.93285	21.10351	16.7409	
Mauritius	Deaths	Male	2023	20.89186	23.65913	18.39037	
Mauritius	Deaths	Female	2010	6.567215	7.74653	5.548117	
Mauritius	Deaths	Female	2011	6.787882	8.008635	5.806272	
Mauritius	Deaths	Female	2012	6.885448	8.162281	5.895052	
Mauritius	Deaths	Female	2013	7.074766	8.452309	6.018773	
Mauritius	Deaths	Female	2014	6.91412	8.196575	5.878355	
Mauritius	Deaths	Female	2015	7.248178	8.506755	6.219907	
Mauritius	Deaths	Female	2016	7.176904	8.567634	6.140034	
Mauritius	Deaths	Female	2017	7.434216	8.80109	6.345624	
Mauritius	Deaths	Female	2018	8.027051	9.519554	6.832596	
Mauritius	Deaths	Female	2019	7.887245	9.322556	6.685817	
Mauritius	Deaths	Female	2020	7.732334	8.964305	6.581295	
Mauritius	Deaths	Female	2021	7.607559	8.796432	6.475057	
Mauritius	Deaths	Female	2022	8.37283	9.660814	7.212621	
Mauritius	Deaths	Female	2023	8.858647	10.33471	7.606379	
Mauritius	DALYs	Male	2010	454.4998	506.3947	405.1399	
Mauritius	DALYs	Male	2011	461.6297	520.3302	410.7952	
Mauritius	DALYs	Male	2012	475.5408	534.7327	427.2094	
Mauritius	DALYs	Male	2013	500.9054	562.4256	449.737	
Mauritius	DALYs	Male	2014	493.6677	556.9118	444.7757	

Mauritius	DALYs	Male	2015	487.7664	546.3288	440.1752	
Mauritius	DALYs	Male	2016	494.0595	546.1827	447.5664	
Mauritius	DALYs	Male	2017	483.9661	541.7099	431.5192	
Mauritius	DALYs	Male	2018	474.7514	532.835	422.211	
Mauritius	DALYs	Male	2019	487.5076	549.9263	433.1129	
Mauritius	DALYs	Male	2020	458.9425	526.3932	407.4548	
Mauritius	DALYs	Male	2021	462.5766	520.7587	415.2465	
Mauritius	DALYs	Male	2022	466.868	521.8593	411.972	
Mauritius	DALYs	Male	2023	508.7115	578.9891	448.0742	
Mauritius	DALYs	Female	2010	165.0318	195.8216	139.7678	
Mauritius	DALYs	Female	2011	169.8372	200.9778	145.7011	
Mauritius	DALYs	Female	2012	171.6145	203.6195	147.585	
Mauritius	DALYs	Female	2013	175.8678	211.5534	150.3578	
Mauritius	DALYs	Female	2014	170.9118	204.5807	145.4841	
Mauritius	DALYs	Female	2015	179.6503	213.3046	153.9923	
Mauritius	DALYs	Female	2016	178.0905	212.5337	153.2605	
Mauritius	DALYs	Female	2017	182.1316	216.0667	155.3669	
Mauritius	DALYs	Female	2018	194.3576	231.5151	165.7805	
Mauritius	DALYs	Female	2019	190.4059	224.0583	160.7512	
Mauritius	DALYs	Female	2020	187.2628	216.6996	158.6687	
Mauritius	DALYs	Female	2021	183.7321	212.2769	156.3457	
Mauritius	DALYs	Female	2022	201.3493	233.6826	173.6304	
Mauritius	DALYs	Female	2023	209.6334	243.7994	180.0425	
Mexico	Incidence	Male	2010	8.832209	9.590165	8.208222	
Mexico	Incidence	Male	2011	8.892941	9.789745	8.234957	
Mexico	Incidence	Male	2012	8.558329	9.407947	7.926649	
Mexico	Incidence	Male	2013	8.41321	9.383176	7.728003	
Mexico	Incidence	Male	2014	8.367204	9.286876	7.716711	
Mexico	Incidence	Male	2015	8.39087	9.224912	7.737493	
Mexico	Incidence	Male	2016	8.409672	9.289888	7.743819	
Mexico	Incidence	Male	2017	8.531903	9.422516	7.896861	
Mexico	Incidence	Male	2018	8.399552	9.265503	7.706012	
Mexico	Incidence	Male	2019	8.236564	9.041136	7.548032	
Mexico	Incidence	Male	2020	8.185641	9.004226	7.527812	
Mexico	Incidence	Male	2021	7.935265	8.678584	7.299492	
Mexico	Incidence	Male	2022	7.572109	8.293322	6.931904	

Mexico	Incidence	Male	2023	7.545126	8.2342	6.905667	
Mexico	Incidence	Female	2010	4.961035	5.746703	4.414513	
Mexico	Incidence	Female	2011	5.01067	5.833963	4.43826	
Mexico	Incidence	Female	2012	4.851155	5.675352	4.290169	
Mexico	Incidence	Female	2013	4.934594	5.754175	4.359362	
Mexico	Incidence	Female	2014	5.008562	5.82439	4.434126	
Mexico	Incidence	Female	2015	5.106326	5.817625	4.540415	
Mexico	Incidence	Female	2016	5.244571	5.910742	4.653093	
Mexico	Incidence	Female	2017	5.418959	6.090664	4.799342	
Mexico	Incidence	Female	2018	5.501416	6.16091	4.885966	
Mexico	Incidence	Female	2019	5.450741	6.043457	4.881492	
Mexico	Incidence	Female	2020	5.391171	5.981179	4.848732	
Mexico	Incidence	Female	2021	5.639131	6.254942	5.08369	
Mexico	Incidence	Female	2022	5.404593	6.058212	4.830315	
Mexico	Incidence	Female	2023	5.52461	6.281282	4.899119	
Mexico	Deaths	Male	2010	8.963523	9.680255	8.366997	
Mexico	Deaths	Male	2011	9.014235	9.864313	8.344679	
Mexico	Deaths	Male	2012	8.683082	9.558173	8.029673	
Mexico	Deaths	Male	2013	8.534414	9.425462	7.845795	
Mexico	Deaths	Male	2014	8.478215	9.321049	7.783779	
Mexico	Deaths	Male	2015	8.496543	9.2755	7.859546	
Mexico	Deaths	Male	2016	8.502155	9.254499	7.857342	
Mexico	Deaths	Male	2017	8.605463	9.460093	7.973058	
Mexico	Deaths	Male	2018	8.459589	9.308194	7.811241	
Mexico	Deaths	Male	2019	8.305304	9.076974	7.675821	
Mexico	Deaths	Male	2020	8.23319	8.93747	7.628071	
Mexico	Deaths	Male	2021	7.976818	8.648384	7.423962	
Mexico	Deaths	Male	2022	7.617226	8.292131	7.035138	
Mexico	Deaths	Male	2023	7.579721	8.277035	6.998278	
Mexico	Deaths	Female	2010	4.752673	5.479747	4.198773	
Mexico	Deaths	Female	2011	4.765451	5.513189	4.233003	
Mexico	Deaths	Female	2012	4.603878	5.357178	4.097113	
Mexico	Deaths	Female	2013	4.660553	5.384092	4.149638	
Mexico	Deaths	Female	2014	4.736984	5.402317	4.233557	
Mexico	Deaths	Female	2015	4.817162	5.46599	4.318951	
Mexico	Deaths	Female	2016	4.940985	5.556205	4.431826	

Mexico	Deaths	Female	2017	5.094002	5.683187	4.60708	
Mexico	Deaths	Female	2018	5.172827	5.757649	4.689449	
Mexico	Deaths	Female	2019	5.125569	5.62521	4.651984	
Mexico	Deaths	Female	2020	5.05042	5.499861	4.583401	
Mexico	Deaths	Female	2021	5.273388	5.767606	4.789087	
Mexico	Deaths	Female	2022	5.060695	5.575008	4.58984	
Mexico	Deaths	Female	2023	5.164054	5.760997	4.605498	
Mexico	DALYs	Male	2010	210.0648	230.0583	195.3423	
Mexico	DALYs	Male	2011	210.7764	230.7496	195.4493	
Mexico	DALYs	Male	2012	201.6441	223.5107	185.8245	
Mexico	DALYs	Male	2013	197.7242	218.9071	181.0429	
Mexico	DALYs	Male	2014	196.5765	217.0894	180.2211	
Mexico	DALYs	Male	2015	196.8325	216.5709	180.8825	
Mexico	DALYs	Male	2016	197.2883	215.9832	181.5754	
Mexico	DALYs	Male	2017	200.9267	222.5884	186.5475	
Mexico	DALYs	Male	2018	198.7197	218.768	183.0298	
Mexico	DALYs	Male	2019	194.1396	212.9698	179.0369	
Mexico	DALYs	Male	2020	192.9591	210.8598	178.7432	
Mexico	DALYs	Male	2021	186.8782	204.5459	173.2258	
Mexico	DALYs	Male	2022	177.1274	193.6138	162.7985	
Mexico	DALYs	Male	2023	175.2919	192.4118	160.4885	
Mexico	DALYs	Female	2010	114.2988	133.0745	101.2046	
Mexico	DALYs	Female	2011	115.381	133.8913	102.9996	
Mexico	DALYs	Female	2012	111.0367	129.827	98.8022	
Mexico	DALYs	Female	2013	113.6118	132.8761	101.172	
Mexico	DALYs	Female	2014	114.5165	131.5355	102.2204	
Mexico	DALYs	Female	2015	116.6457	133.7982	104.0563	
Mexico	DALYs	Female	2016	118.9287	135.4364	106.5114	
Mexico	DALYs	Female	2017	122.5806	137.8963	109.398	
Mexico	DALYs	Female	2018	124.2984	139.1996	111.3929	
Mexico	DALYs	Female	2019	122.7626	135.8905	110.4486	
Mexico	DALYs	Female	2020	121.3075	132.8841	109.4409	
Mexico	DALYs	Female	2021	126.2359	138.8056	114.2818	
Mexico	DALYs	Female	2022	120.1584	133.2042	108.2143	
Mexico	DALYs	Female	2023	121.3221	136.347	107.7614	
Mongolia	Incidence	Male	2010	22.39725	30.47088	17.4043	

Mongolia	Incidence	Male	2011	23.31474	31.2472	18.05565	
Mongolia	Incidence	Male	2012	24.14674	31.60329	18.30825	
Mongolia	Incidence	Male	2013	24.45785	32.50137	18.62835	
Mongolia	Incidence	Male	2014	24.24291	32.63738	18.5906	
Mongolia	Incidence	Male	2015	23.01593	30.50432	18.03563	
Mongolia	Incidence	Male	2016	23.06781	29.74453	18.47794	
Mongolia	Incidence	Male	2017	23.57184	30.61347	19.19875	
Mongolia	Incidence	Male	2018	23.96811	31.8469	19.61967	
Mongolia	Incidence	Male	2019	23.67106	31.51088	19.35623	
Mongolia	Incidence	Male	2020	23.80232	32.48716	19.71601	
Mongolia	Incidence	Male	2021	27.11287	37.01684	22.38004	
Mongolia	Incidence	Male	2022	29.13782	39.0465	23.16518	
Mongolia	Incidence	Male	2023	28.88573	40.43595	22.1513	
Mongolia	Incidence	Female	2010	5.756747	7.741145	4.008945	
Mongolia	Incidence	Female	2011	5.804817	7.698825	4.033598	
Mongolia	Incidence	Female	2012	5.90292	7.85325	4.032987	
Mongolia	Incidence	Female	2013	5.991495	7.950628	4.078465	
Mongolia	Incidence	Female	2014	6.002871	8.102725	4.171379	
Mongolia	Incidence	Female	2015	5.918785	7.83325	4.197505	
Mongolia	Incidence	Female	2016	5.82728	7.438981	4.358215	
Mongolia	Incidence	Female	2017	5.839791	7.5596	4.625032	
Mongolia	Incidence	Female	2018	5.905374	7.738734	4.737807	
Mongolia	Incidence	Female	2019	5.803824	7.739138	4.597888	
Mongolia	Incidence	Female	2020	5.83235	7.500005	4.6492	
Mongolia	Incidence	Female	2021	6.674249	8.700276	5.220555	
Mongolia	Incidence	Female	2022	6.539843	8.353676	4.966468	
Mongolia	Incidence	Female	2023	6.582898	8.444732	4.99272	
Mongolia	Deaths	Male	2010	21.69879	29.34321	16.77479	
Mongolia	Deaths	Male	2011	22.56621	29.91056	17.50557	
Mongolia	Deaths	Male	2012	23.35447	30.48219	17.77791	
Mongolia	Deaths	Male	2013	23.64041	31.29937	18.08847	
Mongolia	Deaths	Male	2014	23.48666	31.1924	18.0949	
Mongolia	Deaths	Male	2015	22.29138	29.29052	17.55468	
Mongolia	Deaths	Male	2016	22.36522	28.55146	18.01039	
Mongolia	Deaths	Male	2017	22.84598	29.23304	18.62087	
Mongolia	Deaths	Male	2018	23.22656	30.47335	19.06984	

Mongolia	Deaths	Male	2019	22.95996	30.18323	18.86285	
Mongolia	Deaths	Male	2020	23.11008	31.09601	19.20986	
Mongolia	Deaths	Male	2021	26.31089	35.40287	21.81298	
Mongolia	Deaths	Male	2022	28.18498	37.32871	22.57095	
Mongolia	Deaths	Male	2023	27.89453	38.6147	21.423	
Mongolia	Deaths	Female	2010	5.63375	7.602053	3.943196	
Mongolia	Deaths	Female	2011	5.676946	7.587598	3.960961	
Mongolia	Deaths	Female	2012	5.771795	7.670506	3.979993	
Mongolia	Deaths	Female	2013	5.860105	7.619726	3.99758	
Mongolia	Deaths	Female	2014	5.878285	7.783614	4.107473	
Mongolia	Deaths	Female	2015	5.802963	7.686923	4.151017	
Mongolia	Deaths	Female	2016	5.726682	7.358459	4.318865	
Mongolia	Deaths	Female	2017	5.743387	7.470342	4.497146	
Mongolia	Deaths	Female	2018	5.814023	7.611343	4.65994	
Mongolia	Deaths	Female	2019	5.718044	7.425933	4.52689	
Mongolia	Deaths	Female	2020	5.750128	7.401136	4.623502	
Mongolia	Deaths	Female	2021	6.58272	8.459555	5.150911	
Mongolia	Deaths	Female	2022	6.435786	8.067169	4.890559	
Mongolia	Deaths	Female	2023	6.460171	8.238554	4.878123	
Mongolia	DALYs	Male	2010	645.2471	882.6428	500.0054	
Mongolia	DALYs	Male	2011	671.5226	919.4074	520.102	
Mongolia	DALYs	Male	2012	694.9224	930.7274	524.5647	
Mongolia	DALYs	Male	2013	703.9534	948.9214	530.2512	
Mongolia	DALYs	Male	2014	692.1961	948.7959	525.1686	
Mongolia	DALYs	Male	2015	656.0165	891.1397	507.7732	
Mongolia	DALYs	Male	2016	655.0821	863.5459	521.9681	
Mongolia	DALYs	Male	2017	666.747	887.1772	540.4166	
Mongolia	DALYs	Male	2018	676.5517	915.9891	546.3117	
Mongolia	DALYs	Male	2019	664.3522	905.2787	540.3547	
Mongolia	DALYs	Male	2020	665.5257	932.7902	544.6102	
Mongolia	DALYs	Male	2021	756.6804	1062.705	612.6806	
Mongolia	DALYs	Male	2022	817.8636	1122.469	642.2083	
Mongolia	DALYs	Male	2023	812.6629	1167.54	611.3032	
Mongolia	DALYs	Female	2010	166.1367	226.5422	114.6808	
Mongolia	DALYs	Female	2011	167.3372	227.2467	114.3585	
Mongolia	DALYs	Female	2012	170.2897	228.8332	114.9176	

Mongolia	DALYs	Female	2013	172.4468	235.4302	115.8561	
Mongolia	DALYs	Female	2014	171.7655	236.9015	118.7553	
Mongolia	DALYs	Female	2015	168.5734	226.0277	117.7571	
Mongolia	DALYs	Female	2016	164.6486	214.144	120.4887	
Mongolia	DALYs	Female	2017	163.8417	218.3486	127.3855	
Mongolia	DALYs	Female	2018	164.4602	222.137	130.8697	
Mongolia	DALYs	Female	2019	160.9348	218.8888	127.2217	
Mongolia	DALYs	Female	2020	161.5153	216.5678	127.0836	
Mongolia	DALYs	Female	2021	183.8974	249.7235	143.807	
Mongolia	DALYs	Female	2022	181.1773	237.7853	136.2911	
Mongolia	DALYs	Female	2023	182.9335	242.217	137.249	
Montenegro	Incidence	Male	2010	99.3726	121.2301	82.89288	
Montenegro	Incidence	Male	2011	101.2753	120.5306	82.64184	
Montenegro	Incidence	Male	2012	102.6482	122.6309	83.10578	
Montenegro	Incidence	Male	2013	104.9152	126.4497	83.58374	
Montenegro	Incidence	Male	2014	106.405	128.0895	82.5129	
Montenegro	Incidence	Male	2015	96.14787	115.4143	75.68258	
Montenegro	Incidence	Male	2016	97.95033	117.4062	76.5575	
Montenegro	Incidence	Male	2017	94.1053	112.5062	72.72201	
Montenegro	Incidence	Male	2018	98.97997	119.4813	77.45518	
Montenegro	Incidence	Male	2019	92.93178	112.8436	72.52207	
Montenegro	Incidence	Male	2020	100.1905	121.231	78.39298	
Montenegro	Incidence	Male	2021	104.5292	127.495	82.47043	
Montenegro	Incidence	Male	2022	106.7802	132.6863	83.82353	
Montenegro	Incidence	Male	2023	106.365	134.0626	82.24185	
Montenegro	Incidence	Female	2010	34.8731	45.21299	27.21186	
Montenegro	Incidence	Female	2011	35.8531	46.87321	27.43372	
Montenegro	Incidence	Female	2012	36.86473	48.09604	27.67992	
Montenegro	Incidence	Female	2013	38.13927	49.79883	28.61494	
Montenegro	Incidence	Female	2014	38.97944	50.8259	28.7683	
Montenegro	Incidence	Female	2015	39.11105	51.19424	28.77497	
Montenegro	Incidence	Female	2016	39.85378	51.87619	29.25015	
Montenegro	Incidence	Female	2017	38.52373	50.05898	28.35532	
Montenegro	Incidence	Female	2018	40.7767	52.75091	30.06348	
Montenegro	Incidence	Female	2019	37.98948	48.70905	28.60091	
Montenegro	Incidence	Female	2020	39.92736	50.50479	31.06454	

Montenegro	Incidence	Female	2021	38.28699	48.06281	28.74985	
Montenegro	Incidence	Female	2022	40.3669	51.97397	30.30291	
Montenegro	Incidence	Female	2023	42.28965	53.91461	31.82723	
Montenegro	Deaths	Male	2010	96.19448	117.2023	80.67551	
Montenegro	Deaths	Male	2011	98.22342	116.4913	80.86249	
Montenegro	Deaths	Male	2012	99.59794	118.9214	81.50975	
Montenegro	Deaths	Male	2013	101.6915	121.9172	81.68179	
Montenegro	Deaths	Male	2014	103.1779	123.5595	81.29557	
Montenegro	Deaths	Male	2015	93.29648	111.3839	72.58872	
Montenegro	Deaths	Male	2016	95.27678	112.4226	74.48674	
Montenegro	Deaths	Male	2017	90.95131	108.9159	71.02927	
Montenegro	Deaths	Male	2018	96.22916	115.9168	75.08806	
Montenegro	Deaths	Male	2019	89.32604	107.3939	69.25336	
Montenegro	Deaths	Male	2020	96.86996	117.4014	75.1352	
Montenegro	Deaths	Male	2021	100.9728	123.9024	78.94586	
Montenegro	Deaths	Male	2022	102.9037	127.6787	80.62874	
Montenegro	Deaths	Male	2023	102.4987	128.9704	78.52205	
Montenegro	Deaths	Female	2010	33.40496	43.28567	26.15899	
Montenegro	Deaths	Female	2011	34.37293	45.36107	26.31016	
Montenegro	Deaths	Female	2012	35.43077	46.494	26.37513	
Montenegro	Deaths	Female	2013	36.62169	47.57284	27.47726	
Montenegro	Deaths	Female	2014	37.48562	48.16326	27.41648	
Montenegro	Deaths	Female	2015	37.8246	49.02481	27.67112	
Montenegro	Deaths	Female	2016	38.51942	49.84257	28.09559	
Montenegro	Deaths	Female	2017	36.96119	47.92313	27.50892	
Montenegro	Deaths	Female	2018	39.62748	51.2027	29.26277	
Montenegro	Deaths	Female	2019	36.33792	46.60604	27.29011	
Montenegro	Deaths	Female	2020	38.62224	48.46595	29.89412	
Montenegro	Deaths	Female	2021	37.35247	47.20157	28.18094	
Montenegro	Deaths	Female	2022	39.27606	50.62717	29.63099	
Montenegro	Deaths	Female	2023	40.89626	51.96712	31.0206	
Montenegro	DALYs	Male	2010	2489.024	3030.278	2066.593	
Montenegro	DALYs	Male	2011	2512.728	3013.486	2044.835	
Montenegro	DALYs	Male	2012	2528.25	3036.081	2029.073	
Montenegro	DALYs	Male	2013	2566.241	3104.101	2027.358	
Montenegro	DALYs	Male	2014	2591.306	3150.684	2009.188	

Montenegro	DALYs	Male	2015	2324.968	2820.279	1790.044	
Montenegro	DALYs	Male	2016	2344.314	2818.201	1813.519	
Montenegro	DALYs	Male	2017	2271.573	2770.285	1753.701	
Montenegro	DALYs	Male	2018	2344.722	2871.133	1837.142	
Montenegro	DALYs	Male	2019	2241.509	2720.331	1743.032	
Montenegro	DALYs	Male	2020	2368.317	2898.164	1859.299	
Montenegro	DALYs	Male	2021	2455.402	3015.927	1933.235	
Montenegro	DALYs	Male	2022	2514.923	3131.148	1997.979	
Montenegro	DALYs	Male	2023	2489.903	3140.075	1922.789	
Montenegro	DALYs	Female	2010	866.9256	1125.765	676.3872	
Montenegro	DALYs	Female	2011	882.3241	1157.685	677.1325	
Montenegro	DALYs	Female	2012	896.4305	1176.395	682.6762	
Montenegro	DALYs	Female	2013	919.2999	1200.086	686.2066	
Montenegro	DALYs	Female	2014	933.7096	1197.943	681.875	
Montenegro	DALYs	Female	2015	924.4124	1198.6	681.5323	
Montenegro	DALYs	Female	2016	934.0594	1202.578	687.6186	
Montenegro	DALYs	Female	2017	911.7247	1171.449	672.624	
Montenegro	DALYs	Female	2018	938.4779	1196.16	698.1146	
Montenegro	DALYs	Female	2019	896.0443	1147.279	683.3448	
Montenegro	DALYs	Female	2020	914.8178	1145.418	719.4231	
Montenegro	DALYs	Female	2021	856.0791	1066.338	667.0358	
Montenegro	DALYs	Female	2022	902.1357	1141.157	703.3974	
Montenegro	DALYs	Female	2023	951.2032	1203.207	724.1722	
Morocco	Incidence	Male	2010	13.33002	20.87739	7.229574	
Morocco	Incidence	Male	2011	13.70798	21.85168	7.482666	
Morocco	Incidence	Male	2012	14.21566	23.39773	7.740696	
Morocco	Incidence	Male	2013	15.00712	24.29857	7.986635	
Morocco	Incidence	Male	2014	15.71753	25.33515	8.146301	
Morocco	Incidence	Male	2015	16.61282	26.73376	8.680237	
Morocco	Incidence	Male	2016	17.51965	27.9826	9.274588	
Morocco	Incidence	Male	2017	18.55712	29.66188	9.513743	
Morocco	Incidence	Male	2018	19.49317	31.71227	9.867453	
Morocco	Incidence	Male	2019	20.73317	34.15475	10.22106	
Morocco	Incidence	Male	2020	21.55803	34.16029	10.61575	
Morocco	Incidence	Male	2021	23.31226	37.0342	11.24815	
Morocco	Incidence	Male	2022	25.57587	39.48799	12.57533	

Morocco	Incidence	Male	2023	26.37579	41.93984	12.53291	
Morocco	Incidence	Female	2010	1.561849	2.390889	0.961397	
Morocco	Incidence	Female	2011	1.652087	2.537026	1.037024	
Morocco	Incidence	Female	2012	1.742486	2.65043	1.116709	
Morocco	Incidence	Female	2013	1.844807	2.772032	1.165347	
Morocco	Incidence	Female	2014	1.93717	2.872722	1.228689	
Morocco	Incidence	Female	2015	2.063256	2.982659	1.331778	
Morocco	Incidence	Female	2016	2.186379	3.136456	1.381061	
Morocco	Incidence	Female	2017	2.294484	3.350581	1.4318	
Morocco	Incidence	Female	2018	2.438466	3.56427	1.540594	
Morocco	Incidence	Female	2019	2.611422	3.71292	1.650504	
Morocco	Incidence	Female	2020	2.833744	4.140979	1.801204	
Morocco	Incidence	Female	2021	3.08059	4.385563	1.886332	
Morocco	Incidence	Female	2022	3.292335	4.63225	2.018497	
Morocco	Incidence	Female	2023	3.404481	4.853508	2.10843	
Morocco	Deaths	Male	2010	13.32164	20.86523	7.357926	
Morocco	Deaths	Male	2011	13.68936	21.74648	7.545672	
Morocco	Deaths	Male	2012	14.18344	23.40695	7.782992	
Morocco	Deaths	Male	2013	14.95198	24.18925	8.087031	
Morocco	Deaths	Male	2014	15.63929	25.23617	8.229001	
Morocco	Deaths	Male	2015	16.50282	26.54602	8.758337	
Morocco	Deaths	Male	2016	17.38112	27.78033	9.340346	
Morocco	Deaths	Male	2017	18.38737	29.44068	9.524931	
Morocco	Deaths	Male	2018	19.30139	31.27493	9.89547	
Morocco	Deaths	Male	2019	20.50855	33.52178	10.3006	
Morocco	Deaths	Male	2020	21.31327	33.85931	10.7036	
Morocco	Deaths	Male	2021	22.99784	36.59781	11.32754	
Morocco	Deaths	Male	2022	25.21909	38.50332	12.67348	
Morocco	Deaths	Male	2023	26.04417	41.30015	12.59212	
Morocco	Deaths	Female	2010	1.511737	2.308515	0.939812	
Morocco	Deaths	Female	2011	1.598364	2.44993	1.016046	
Morocco	Deaths	Female	2012	1.684049	2.56288	1.083688	
Morocco	Deaths	Female	2013	1.77962	2.671451	1.124045	
Morocco	Deaths	Female	2014	1.864977	2.794266	1.185777	
Morocco	Deaths	Female	2015	1.98279	2.850197	1.28286	
Morocco	Deaths	Female	2016	2.097447	3.006665	1.335697	

Morocco	Deaths	Female	2017	2.197611	3.182617	1.378692	
Morocco	Deaths	Female	2018	2.334225	3.431596	1.479127	
Morocco	Deaths	Female	2019	2.494899	3.567377	1.582019	
Morocco	Deaths	Female	2020	2.698492	3.940567	1.724894	
Morocco	Deaths	Female	2021	2.924617	4.145113	1.794775	
Morocco	Deaths	Female	2022	3.136592	4.400851	1.920443	
Morocco	Deaths	Female	2023	3.249858	4.593931	2.007973	
Morocco	DALYs	Male	2010	349.46	541.9473	185.8261	
Morocco	DALYs	Male	2011	359.1663	573.7102	193.6255	
Morocco	DALYs	Male	2012	372.3149	602.6955	199.9708	
Morocco	DALYs	Male	2013	393.1406	636.6671	205.0544	
Morocco	DALYs	Male	2014	411.4194	661.9375	210.0272	
Morocco	DALYs	Male	2015	434.3504	696.0427	226.9773	
Morocco	DALYs	Male	2016	457.5687	729.0644	240.9429	
Morocco	DALYs	Male	2017	484.352	772.1232	244.696	
Morocco	DALYs	Male	2018	507.9903	814.6045	255.9739	
Morocco	DALYs	Male	2019	539.1871	876.3942	264.2166	
Morocco	DALYs	Male	2020	558.4885	886.3075	273.2532	
Morocco	DALYs	Male	2021	602.1011	962.5006	287.2271	
Morocco	DALYs	Male	2022	656.3604	1007.136	315.5738	
Morocco	DALYs	Male	2023	670.6403	1077.576	310.6381	
Morocco	DALYs	Female	2010	43.21928	66.08767	27.15076	
Morocco	DALYs	Female	2011	45.67707	69.63071	28.80906	
Morocco	DALYs	Female	2012	48.18586	72.73334	31.13086	
Morocco	DALYs	Female	2013	51.10188	76.92169	32.4638	
Morocco	DALYs	Female	2014	53.70523	79.61094	34.37056	
Morocco	DALYs	Female	2015	57.03829	81.00664	36.72698	
Morocco	DALYs	Female	2016	60.47195	86.04645	38.87482	
Morocco	DALYs	Female	2017	63.54415	92.51823	40.38588	
Morocco	DALYs	Female	2018	67.4358	99.24319	42.9695	
Morocco	DALYs	Female	2019	72.21247	103.0729	46.02716	
Morocco	DALYs	Female	2020	78.35841	113.7198	50.49517	
Morocco	DALYs	Female	2021	85.04458	120.3202	53.1106	
Morocco	DALYs	Female	2022	89.45525	127.1009	54.75864	
Morocco	DALYs	Female	2023	91.21099	129.8614	56.92223	
Mozambique	Incidence	Male	2010	2.10243	2.997072	1.561087	

Mozambique	Incidence	Male	2011	2.063484	2.906842	1.529935	
Mozambique	Incidence	Male	2012	2.06693	2.954452	1.549317	
Mozambique	Incidence	Male	2013	2.043232	2.838574	1.514432	
Mozambique	Incidence	Male	2014	1.956625	2.646668	1.449638	
Mozambique	Incidence	Male	2015	1.907157	2.58158	1.380292	
Mozambique	Incidence	Male	2016	1.874704	2.515952	1.348213	
Mozambique	Incidence	Male	2017	1.862405	2.566251	1.336926	
Mozambique	Incidence	Male	2018	1.902936	2.677917	1.357922	
Mozambique	Incidence	Male	2019	1.999396	2.888113	1.423417	
Mozambique	Incidence	Male	2020	2.058877	2.968587	1.411354	
Mozambique	Incidence	Male	2021	2.307197	3.328465	1.626439	
Mozambique	Incidence	Male	2022	2.150878	3.121973	1.446832	
Mozambique	Incidence	Male	2023	2.170903	3.089472	1.423775	
Mozambique	Incidence	Female	2010	0.424061	0.609732	0.296356	
Mozambique	Incidence	Female	2011	0.412214	0.580873	0.285454	
Mozambique	Incidence	Female	2012	0.406455	0.567293	0.269285	
Mozambique	Incidence	Female	2013	0.399183	0.554648	0.264965	
Mozambique	Incidence	Female	2014	0.384884	0.530463	0.252229	
Mozambique	Incidence	Female	2015	0.381836	0.524935	0.24901	
Mozambique	Incidence	Female	2016	0.380303	0.515627	0.249663	
Mozambique	Incidence	Female	2017	0.381961	0.515003	0.254313	
Mozambique	Incidence	Female	2018	0.386276	0.516554	0.270337	
Mozambique	Incidence	Female	2019	0.401963	0.554146	0.288364	
Mozambique	Incidence	Female	2020	0.409365	0.558585	0.296968	
Mozambique	Incidence	Female	2021	0.406193	0.550845	0.296137	
Mozambique	Incidence	Female	2022	0.447539	0.628065	0.314198	
Mozambique	Incidence	Female	2023	0.464357	0.632652	0.304935	
Mozambique	Deaths	Male	2010	2.122303	2.981143	1.582533	
Mozambique	Deaths	Male	2011	2.084144	2.940311	1.541372	
Mozambique	Deaths	Male	2012	2.087659	2.967811	1.558747	
Mozambique	Deaths	Male	2013	2.066698	2.909955	1.524402	
Mozambique	Deaths	Male	2014	1.984023	2.718278	1.476328	
Mozambique	Deaths	Male	2015	1.940879	2.621239	1.404033	
Mozambique	Deaths	Male	2016	1.909178	2.558636	1.370327	
Mozambique	Deaths	Male	2017	1.898614	2.610481	1.364978	
Mozambique	Deaths	Male	2018	1.938905	2.713891	1.374687	

Mozambique	Deaths	Male	2019	2.033283	2.916749	1.453613	
Mozambique	Deaths	Male	2020	2.094911	3.003609	1.450088	
Mozambique	Deaths	Male	2021	2.376106	3.376621	1.655903	
Mozambique	Deaths	Male	2022	2.19311	3.166271	1.478688	
Mozambique	Deaths	Male	2023	2.208908	3.135204	1.444838	
Mozambique	Deaths	Female	2010	0.425486	0.610394	0.297901	
Mozambique	Deaths	Female	2011	0.414291	0.576736	0.287498	
Mozambique	Deaths	Female	2012	0.409027	0.571307	0.271139	
Mozambique	Deaths	Female	2013	0.402461	0.557806	0.266484	
Mozambique	Deaths	Female	2014	0.388719	0.534659	0.253663	
Mozambique	Deaths	Female	2015	0.386509	0.532016	0.253848	
Mozambique	Deaths	Female	2016	0.384749	0.52158	0.253227	
Mozambique	Deaths	Female	2017	0.386778	0.521039	0.260643	
Mozambique	Deaths	Female	2018	0.391202	0.521432	0.275138	
Mozambique	Deaths	Female	2019	0.406728	0.559006	0.290592	
Mozambique	Deaths	Female	2020	0.41331	0.561042	0.298637	
Mozambique	Deaths	Female	2021	0.408899	0.549716	0.30092	
Mozambique	Deaths	Female	2022	0.449767	0.624393	0.316962	
Mozambique	Deaths	Female	2023	0.46591	0.63065	0.307511	
Mozambique	DALYs	Male	2010	59.97302	86.68913	44.05181	
Mozambique	DALYs	Male	2011	58.65853	82.3392	43.08327	
Mozambique	DALYs	Male	2012	58.55661	83.10575	43.80694	
Mozambique	DALYs	Male	2013	57.60737	80.56457	43.51137	
Mozambique	DALYs	Male	2014	54.84494	73.82057	40.38604	
Mozambique	DALYs	Male	2015	53.01409	72.09605	38.54759	
Mozambique	DALYs	Male	2016	51.97315	70.38064	37.33999	
Mozambique	DALYs	Male	2017	51.47525	72.20031	36.90568	
Mozambique	DALYs	Male	2018	52.52154	74.90477	37.08244	
Mozambique	DALYs	Male	2019	55.21977	79.95654	38.88211	
Mozambique	DALYs	Male	2020	56.73739	82.02056	38.3022	
Mozambique	DALYs	Male	2021	62.07349	90.22377	43.43839	
Mozambique	DALYs	Male	2022	58.59593	85.639	39.19561	
Mozambique	DALYs	Male	2023	59.44515	85.26061	39.16556	
Mozambique	DALYs	Female	2010	12.39834	18.08547	8.561542	
Mozambique	DALYs	Female	2011	11.99591	17.29733	8.260584	
Mozambique	DALYs	Female	2012	11.77048	16.66151	7.801632	

Mozambique	DALYs	Female	2013	11.49895	16.1375	7.690446	
Mozambique	DALYs	Female	2014	11.04472	15.32978	7.22672	
Mozambique	DALYs	Female	2015	10.89599	14.97277	7.123413	
Mozambique	DALYs	Female	2016	10.84834	14.74793	7.111458	
Mozambique	DALYs	Female	2017	10.84579	14.74421	7.119935	
Mozambique	DALYs	Female	2018	10.92557	14.88408	7.601412	
Mozambique	DALYs	Female	2019	11.33194	15.63192	8.076925	
Mozambique	DALYs	Female	2020	11.53322	15.6869	8.395828	
Mozambique	DALYs	Female	2021	11.45802	15.76256	8.161355	
Mozambique	DALYs	Female	2022	12.65274	17.93303	8.863437	
Mozambique	DALYs	Female	2023	13.21908	18.27897	8.87655	
Myanmar	Incidence	Male	2010	15.21491	21.42133	10.22302	
Myanmar	Incidence	Male	2011	15.19834	21.00502	10.77216	
Myanmar	Incidence	Male	2012	15.42313	21.18059	11.26135	
Myanmar	Incidence	Male	2013	15.92917	21.92184	11.47797	
Myanmar	Incidence	Male	2014	16.32127	23.30191	11.54961	
Myanmar	Incidence	Male	2015	16.67209	23.42747	11.47009	
Myanmar	Incidence	Male	2016	17.37618	24.67857	11.83973	
Myanmar	Incidence	Male	2017	17.67358	25.18602	12.07052	
Myanmar	Incidence	Male	2018	18.23721	25.57954	12.00748	
Myanmar	Incidence	Male	2019	19.0068	26.86415	12.64883	
Myanmar	Incidence	Male	2020	20.29447	29.37965	13.75339	
Myanmar	Incidence	Male	2021	23.06828	35.08675	15.51382	
Myanmar	Incidence	Male	2022	21.38589	32.67424	14.22805	
Myanmar	Incidence	Male	2023	22.09429	33.13712	14.89601	
Myanmar	Incidence	Female	2010	7.648567	11.55253	4.943199	
Myanmar	Incidence	Female	2011	7.817134	11.54165	5.088291	
Myanmar	Incidence	Female	2012	7.94923	11.70922	5.184564	
Myanmar	Incidence	Female	2013	8.242196	12.02496	5.203883	
Myanmar	Incidence	Female	2014	8.559433	12.59993	5.403595	
Myanmar	Incidence	Female	2015	8.831949	13.06683	5.653306	
Myanmar	Incidence	Female	2016	9.24931	13.43583	5.814671	
Myanmar	Incidence	Female	2017	9.574876	14.03072	6.135902	
Myanmar	Incidence	Female	2018	9.934074	14.2977	6.582658	
Myanmar	Incidence	Female	2019	10.47694	14.60613	6.986142	
Myanmar	Incidence	Female	2020	11.05624	15.20656	7.216221	

Myanmar	Incidence	Female	2021	12.20992	16.93537	8.322922	
Myanmar	Incidence	Female	2022	12.25219	16.47531	8.362047	
Myanmar	Incidence	Female	2023	12.90493	17.9126	8.850398	
Myanmar	Deaths	Male	2010	15.45178	21.84529	10.35494	
Myanmar	Deaths	Male	2011	15.43	21.15975	10.92102	
Myanmar	Deaths	Male	2012	15.65091	21.42206	11.38394	
Myanmar	Deaths	Male	2013	16.14761	22.21644	11.61812	
Myanmar	Deaths	Male	2014	16.53083	23.56047	11.66313	
Myanmar	Deaths	Male	2015	16.87409	23.73973	11.54576	
Myanmar	Deaths	Male	2016	17.5726	24.96233	11.96419	
Myanmar	Deaths	Male	2017	17.85683	25.3616	12.16586	
Myanmar	Deaths	Male	2018	18.40693	25.78062	12.14795	
Myanmar	Deaths	Male	2019	19.16386	27.10756	12.74855	
Myanmar	Deaths	Male	2020	20.44302	29.50448	13.82524	
Myanmar	Deaths	Male	2021	23.18977	35.32035	15.47371	
Myanmar	Deaths	Male	2022	21.48189	32.71762	14.2197	
Myanmar	Deaths	Male	2023	22.17117	33.07528	14.88583	
Myanmar	Deaths	Female	2010	7.70022	11.72235	5.025703	
Myanmar	Deaths	Female	2011	7.871962	11.65711	5.079419	
Myanmar	Deaths	Female	2012	8.003532	11.75222	5.211117	
Myanmar	Deaths	Female	2013	8.288592	12.07565	5.228403	
Myanmar	Deaths	Female	2014	8.598285	12.70006	5.414641	
Myanmar	Deaths	Female	2015	8.862212	13.08337	5.666263	
Myanmar	Deaths	Female	2016	9.264691	13.44926	5.810818	
Myanmar	Deaths	Female	2017	9.57677	13.94981	6.111203	
Myanmar	Deaths	Female	2018	9.91852	14.27271	6.5669	
Myanmar	Deaths	Female	2019	10.44172	14.5971	6.931531	
Myanmar	Deaths	Female	2020	10.99706	15.26572	7.188237	
Myanmar	Deaths	Female	2021	12.10961	16.78717	8.224839	
Myanmar	Deaths	Female	2022	12.1419	16.433	8.225083	
Myanmar	Deaths	Female	2023	12.75833	17.80385	8.75465	
Myanmar	DALYs	Male	2010	403.2924	564.456	272.6385	
Myanmar	DALYs	Male	2011	402.7326	565.8796	288.1292	
Myanmar	DALYs	Male	2012	408.6862	568.8978	297.7507	
Myanmar	DALYs	Male	2013	422.672	581.1268	306.4938	
Myanmar	DALYs	Male	2014	433.1475	618.4921	308.0725	

Myanmar	DALYs	Male	2015	442.2128	622.1212	306.2021	
Myanmar	DALYs	Male	2016	460.5386	659.1504	313.6342	
Myanmar	DALYs	Male	2017	468.4899	672.4139	319.0437	
Myanmar	DALYs	Male	2018	483.7712	685.2685	318.5948	
Myanmar	DALYs	Male	2019	503.8788	712.5173	336.1212	
Myanmar	DALYs	Male	2020	536.6607	777.2727	364.9007	
Myanmar	DALYs	Male	2021	608.7738	921.5965	413.3662	
Myanmar	DALYs	Male	2022	565.5307	872.3068	378.504	
Myanmar	DALYs	Male	2023	584.6824	883.897	394.1379	
Myanmar	DALYs	Female	2010	208.4252	314.1771	134.1569	
Myanmar	DALYs	Female	2011	212.4702	313.2006	136.2843	
Myanmar	DALYs	Female	2012	215.6205	317.8864	141.6007	
Myanmar	DALYs	Female	2013	223.7773	324.1533	142.7894	
Myanmar	DALYs	Female	2014	232.4673	341.5841	146.7296	
Myanmar	DALYs	Female	2015	239.7269	354.6305	154.3943	
Myanmar	DALYs	Female	2016	251.2271	366.0216	159.8414	
Myanmar	DALYs	Female	2017	260.2157	381.2121	167.9728	
Myanmar	DALYs	Female	2018	270.342	389.3846	180.8767	
Myanmar	DALYs	Female	2019	285.1636	397.4545	190.8697	
Myanmar	DALYs	Female	2020	300.518	411.6244	201.2088	
Myanmar	DALYs	Female	2021	331.5556	454.4818	226.535	
Myanmar	DALYs	Female	2022	332.2101	442.8275	227.5183	
Myanmar	DALYs	Female	2023	350.9452	472.801	236.5413	
Namibia	Incidence	Male	2010	2.667214	3.50066	1.886116	
Namibia	Incidence	Male	2011	2.724204	3.535381	1.95403	
Namibia	Incidence	Male	2012	2.801311	3.687954	2.039347	
Namibia	Incidence	Male	2013	2.818707	3.707203	1.999741	
Namibia	Incidence	Male	2014	2.836115	3.742541	2.002687	
Namibia	Incidence	Male	2015	2.902567	3.812227	1.960026	
Namibia	Incidence	Male	2016	2.95475	4.045741	1.919782	
Namibia	Incidence	Male	2017	2.935322	4.109428	1.883788	
Namibia	Incidence	Male	2018	2.91818	4.141226	1.885634	
Namibia	Incidence	Male	2019	3.016915	4.20852	1.952698	
Namibia	Incidence	Male	2020	3.09835	4.297329	2.101758	
Namibia	Incidence	Male	2021	3.844926	5.406497	2.722318	
Namibia	Incidence	Male	2022	3.267353	4.556202	2.255117	

Namibia	Incidence	Male	2023	3.316211	4.848777	2.273386	
Namibia	Incidence	Female	2010	1.595632	2.231442	1.15199	
Namibia	Incidence	Female	2011	1.645901	2.219072	1.172007	
Namibia	Incidence	Female	2012	1.710252	2.338276	1.216567	
Namibia	Incidence	Female	2013	1.734563	2.38841	1.174737	
Namibia	Incidence	Female	2014	1.693679	2.335526	1.136878	
Namibia	Incidence	Female	2015	1.697433	2.313607	1.121713	
Namibia	Incidence	Female	2016	1.70885	2.365991	1.14881	
Namibia	Incidence	Female	2017	1.744365	2.393302	1.185825	
Namibia	Incidence	Female	2018	1.852815	2.484749	1.24083	
Namibia	Incidence	Female	2019	1.956566	2.681216	1.301875	
Namibia	Incidence	Female	2020	1.939612	2.588741	1.311138	
Namibia	Incidence	Female	2021	2.138984	2.862049	1.484797	
Namibia	Incidence	Female	2022	2.129766	2.880939	1.426827	
Namibia	Incidence	Female	2023	2.186571	3.009069	1.447527	
Namibia	Deaths	Male	2010	2.579147	3.377777	1.864307	
Namibia	Deaths	Male	2011	2.62738	3.388485	1.902375	
Namibia	Deaths	Male	2012	2.695781	3.524232	1.97319	
Namibia	Deaths	Male	2013	2.708333	3.538137	1.93334	
Namibia	Deaths	Male	2014	2.723505	3.581664	1.963111	
Namibia	Deaths	Male	2015	2.785466	3.642037	1.885493	
Namibia	Deaths	Male	2016	2.832211	3.845777	1.873765	
Namibia	Deaths	Male	2017	2.812451	3.898305	1.826985	
Namibia	Deaths	Male	2018	2.795621	3.930867	1.817603	
Namibia	Deaths	Male	2019	2.882712	3.995292	1.894968	
Namibia	Deaths	Male	2020	2.952356	4.116481	2.022492	
Namibia	Deaths	Male	2021	3.666633	5.180541	2.602493	
Namibia	Deaths	Male	2022	3.111868	4.353209	2.155764	
Namibia	Deaths	Male	2023	3.151936	4.621954	2.169042	
Namibia	Deaths	Female	2010	1.530374	2.124806	1.101693	
Namibia	Deaths	Female	2011	1.574916	2.118164	1.111921	
Namibia	Deaths	Female	2012	1.633739	2.225531	1.15307	
Namibia	Deaths	Female	2013	1.65697	2.28443	1.118413	
Namibia	Deaths	Female	2014	1.620617	2.230331	1.088951	
Namibia	Deaths	Female	2015	1.624634	2.201344	1.075576	
Namibia	Deaths	Female	2016	1.633534	2.260943	1.093634	

Namibia	Deaths	Female	2017	1.663108	2.289035	1.124254	
Namibia	Deaths	Female	2018	1.759048	2.36898	1.175271	
Namibia	Deaths	Female	2019	1.849965	2.524316	1.225957	
Namibia	Deaths	Female	2020	1.825113	2.430913	1.241324	
Namibia	Deaths	Female	2021	2.005161	2.689538	1.390215	
Namibia	Deaths	Female	2022	1.996448	2.71407	1.36087	
Namibia	Deaths	Female	2023	2.047981	2.802193	1.37684	
Namibia	DALYs	Male	2010	79.40073	104.8214	55.2448	
Namibia	DALYs	Male	2011	81.5073	107.0306	58.1831	
Namibia	DALYs	Male	2012	84.09072	111.5701	59.8029	
Namibia	DALYs	Male	2013	84.74701	113.1289	59.42522	
Namibia	DALYs	Male	2014	85.29372	113.569	57.33858	
Namibia	DALYs	Male	2015	87.36118	115.7525	57.32922	
Namibia	DALYs	Male	2016	89.11792	122.4471	57.21273	
Namibia	DALYs	Male	2017	88.54344	125.1785	56.27975	
Namibia	DALYs	Male	2018	87.9702	125.192	56.24774	
Namibia	DALYs	Male	2019	91.32698	127.9901	59.05686	
Namibia	DALYs	Male	2020	94.30084	131.5556	63.05078	
Namibia	DALYs	Male	2021	116.2353	162.832	81.77737	
Namibia	DALYs	Male	2022	99.11469	139.1912	67.48649	
Namibia	DALYs	Male	2023	100.9053	146.9546	67.96303	
Namibia	DALYs	Female	2010	49.00883	68.95951	35.59427	
Namibia	DALYs	Female	2011	50.73469	69.97245	35.72959	
Namibia	DALYs	Female	2012	52.75981	72.64559	36.38662	
Namibia	DALYs	Female	2013	53.33168	73.59723	36.01213	
Namibia	DALYs	Female	2014	51.79694	71.44129	34.36723	
Namibia	DALYs	Female	2015	51.83184	70.7323	33.96579	
Namibia	DALYs	Female	2016	52.23981	71.84986	34.96791	
Namibia	DALYs	Female	2017	53.48963	72.83758	36.16286	
Namibia	DALYs	Female	2018	57.12464	76.24019	38.56968	
Namibia	DALYs	Female	2019	60.67363	81.74395	40.65871	
Namibia	DALYs	Female	2020	60.65594	81.6631	40.73825	
Namibia	DALYs	Female	2021	67.00852	89.65962	46.12744	
Namibia	DALYs	Female	2022	66.6792	89.7796	44.33016	
Namibia	DALYs	Female	2023	68.49249	93.84464	45.05204	
Nepal	Incidence	Male	2010	4.584724	6.908054	3.072452	

Nepal	Incidence	Male	2011	4.664595	6.76748	3.123431	
Nepal	Incidence	Male	2012	4.867269	7.128319	3.217061	
Nepal	Incidence	Male	2013	4.969305	7.509712	3.196812	
Nepal	Incidence	Male	2014	5.177115	7.807476	3.275548	
Nepal	Incidence	Male	2015	5.212191	7.814335	3.41483	
Nepal	Incidence	Male	2016	5.433658	7.987442	3.555214	
Nepal	Incidence	Male	2017	5.485496	8.011508	3.538512	
Nepal	Incidence	Male	2018	5.64624	8.408966	3.656329	
Nepal	Incidence	Male	2019	5.753802	8.771429	3.815629	
Nepal	Incidence	Male	2020	5.828672	8.841409	3.711628	
Nepal	Incidence	Male	2021	6.85377	10.29535	4.415942	
Nepal	Incidence	Male	2022	7.005175	10.48708	4.496708	
Nepal	Incidence	Male	2023	7.370227	11.12352	4.702001	
Nepal	Incidence	Female	2010	2.022202	2.929742	1.310069	
Nepal	Incidence	Female	2011	2.016684	2.931036	1.341614	
Nepal	Incidence	Female	2012	2.0529	3.014662	1.378137	
Nepal	Incidence	Female	2013	2.107012	3.018588	1.369081	
Nepal	Incidence	Female	2014	2.190338	3.110274	1.441561	
Nepal	Incidence	Female	2015	2.215633	3.081261	1.429456	
Nepal	Incidence	Female	2016	2.336262	3.12505	1.506768	
Nepal	Incidence	Female	2017	2.420084	3.322688	1.660765	
Nepal	Incidence	Female	2018	2.523363	3.451037	1.690501	
Nepal	Incidence	Female	2019	2.646675	3.714653	1.774735	
Nepal	Incidence	Female	2020	2.766026	3.932186	1.797704	
Nepal	Incidence	Female	2021	3.22296	4.387299	2.220403	
Nepal	Incidence	Female	2022	3.466085	4.985502	2.310337	
Nepal	Incidence	Female	2023	3.818544	5.501536	2.531307	
Nepal	Deaths	Male	2010	4.517963	6.805274	3.016816	
Nepal	Deaths	Male	2011	4.592932	6.663702	3.072114	
Nepal	Deaths	Male	2012	4.788455	6.997369	3.160996	
Nepal	Deaths	Male	2013	4.885502	7.403715	3.116873	
Nepal	Deaths	Male	2014	5.087451	7.683325	3.211025	
Nepal	Deaths	Male	2015	5.11988	7.662427	3.335149	
Nepal	Deaths	Male	2016	5.337469	7.852696	3.489241	
Nepal	Deaths	Male	2017	5.385559	7.809732	3.475615	
Nepal	Deaths	Male	2018	5.541022	8.186348	3.589805	

Nepal	Deaths	Male	2019	5.645416	8.569714	3.748394	
Nepal	Deaths	Male	2020	5.712293	8.711794	3.652767	
Nepal	Deaths	Male	2021	6.683851	10.05391	4.33776	
Nepal	Deaths	Male	2022	6.863738	10.29213	4.411861	
Nepal	Deaths	Male	2023	7.226507	10.86701	4.589952	
Nepal	Deaths	Female	2010	1.986711	2.897304	1.288087	
Nepal	Deaths	Female	2011	1.983076	2.889994	1.321254	
Nepal	Deaths	Female	2012	2.019181	2.959876	1.353676	
Nepal	Deaths	Female	2013	2.073577	2.977114	1.351901	
Nepal	Deaths	Female	2014	2.157049	3.076422	1.419682	
Nepal	Deaths	Female	2015	2.185337	3.036415	1.41114	
Nepal	Deaths	Female	2016	2.307625	3.096697	1.485364	
Nepal	Deaths	Female	2017	2.393052	3.285958	1.641487	
Nepal	Deaths	Female	2018	2.498121	3.417926	1.680168	
Nepal	Deaths	Female	2019	2.623068	3.666806	1.738687	
Nepal	Deaths	Female	2020	2.745743	3.908888	1.7866	
Nepal	Deaths	Female	2021	3.18668	4.354825	2.18756	
Nepal	Deaths	Female	2022	3.436003	4.943514	2.288416	
Nepal	Deaths	Female	2023	3.776528	5.452041	2.495257	
Nepal	DALYs	Male	2010	129.2578	195.8387	86.58138	
Nepal	DALYs	Male	2011	131.9444	191.6067	88.27732	
Nepal	DALYs	Male	2012	138.0335	203.1692	91.09747	
Nepal	DALYs	Male	2013	141.097	211.1344	91.44372	
Nepal	DALYs	Male	2014	147.0997	222.3198	94.4148	
Nepal	DALYs	Male	2015	148.2035	224.6904	97.42782	
Nepal	DALYs	Male	2016	154.389	229.12	100.9185	
Nepal	DALYs	Male	2017	156.0441	231.2814	101.2583	
Nepal	DALYs	Male	2018	160.77	240.0838	103.8907	
Nepal	DALYs	Male	2019	163.8165	251.066	107.9073	
Nepal	DALYs	Male	2020	165.968	253.7869	104.9447	
Nepal	DALYs	Male	2021	196.4469	294.0199	124.6691	
Nepal	DALYs	Male	2022	198.2954	296.9787	128.0611	
Nepal	DALYs	Male	2023	207.3727	315.6982	133.9137	
Nepal	DALYs	Female	2010	57.23551	83.75042	37.23413	
Nepal	DALYs	Female	2011	57.02182	82.95619	38.38819	
Nepal	DALYs	Female	2012	58.01175	84.92313	39.07885	

Nepal	DALYs	Female	2013	59.41834	85.33304	38.62644	
Nepal	DALYs	Female	2014	61.60546	88.13013	39.98762	
Nepal	DALYs	Female	2015	62.08408	87.14832	39.98927	
Nepal	DALYs	Female	2016	65.13558	87.79821	42.50617	
Nepal	DALYs	Female	2017	67.22519	91.67809	45.85744	
Nepal	DALYs	Female	2018	69.81877	96.43517	47.1819	
Nepal	DALYs	Female	2019	72.90965	102.2293	48.4114	
Nepal	DALYs	Female	2020	75.61493	107.1834	49.19451	
Nepal	DALYs	Female	2021	88.33871	119.6033	60.80481	
Nepal	DALYs	Female	2022	94.07935	132.4915	63.07862	
Nepal	DALYs	Female	2023	103.882	149.095	69.46861	
The Netherland	Incidence	Male	2010	100.5425	106.1368	94.52484	
The Netherland	Incidence	Male	2011	100.7254	107.0948	94.95581	
The Netherland	Incidence	Male	2012	99.6877	105.7201	94.28889	
The Netherland	Incidence	Male	2013	99.61823	105.8914	94.29059	
The Netherland	Incidence	Male	2014	98.00322	104.511	91.88334	
The Netherland	Incidence	Male	2015	97.93224	104.2823	91.76883	
The Netherland	Incidence	Male	2016	97.69652	104.8071	91.43145	
The Netherland	Incidence	Male	2017	95.91691	103.8512	89.7309	
The Netherland	Incidence	Male	2018	93.5138	102.2664	87.32582	
The Netherland	Incidence	Male	2019	90.21101	97.76613	83.96963	
The Netherland	Incidence	Male	2020	87.27713	95.10109	81.72824	
The Netherland	Incidence	Male	2021	86.38838	94.36314	80.98195	
The Netherland	Incidence	Male	2022	87.27102	95.95263	80.95141	
The Netherland	Incidence	Male	2023	87.16047	96.35298	79.65785	
The Netherland	Incidence	Female	2010	60.15849	64.37072	55.6437	
The Netherland	Incidence	Female	2011	63.37949	67.60475	58.79062	
The Netherland	Incidence	Female	2012	65.70115	70.20036	60.60402	
The Netherland	Incidence	Female	2013	67.89395	72.5397	61.96687	
The Netherland	Incidence	Female	2014	69.10077	73.39893	63.08305	
The Netherland	Incidence	Female	2015	70.42467	74.69706	64.66437	
The Netherland	Incidence	Female	2016	71.28906	76.36945	64.15407	
The Netherland	Incidence	Female	2017	70.57267	75.96671	63.23377	
The Netherland	Incidence	Female	2018	71.12637	76.85613	63.83061	
The Netherland	Incidence	Female	2019	69.7357	76.12377	62.48031	
The Netherland	Incidence	Female	2020	68.80822	74.5774	62.35853	

The Netherlanc	Incidence	Female	2021	71.43967	77.3544	64.64712	
The Netherlanc	Incidence	Female	2022	72.73592	78.87514	65.21997	
The Netherlanc	Incidence	Female	2023	71.04502	76.96825	63.7539	
The Netherlanc	Deaths	Male	2010	90.009	95.23041	84.76116	
The Netherlanc	Deaths	Male	2011	89.94638	95.23659	83.53605	
The Netherlanc	Deaths	Male	2012	88.94901	93.86516	83.41962	
The Netherlanc	Deaths	Male	2013	88.68834	93.74567	83.41955	
The Netherlanc	Deaths	Male	2014	87.07316	92.51451	81.10299	
The Netherlanc	Deaths	Male	2015	87.30668	92.80545	81.35422	
The Netherlanc	Deaths	Male	2016	87.54743	93.57362	81.96783	
The Netherlanc	Deaths	Male	2017	86.2899	92.20205	80.68556	
The Netherlanc	Deaths	Male	2018	84.67948	90.89206	79.09194	
The Netherlanc	Deaths	Male	2019	82.06617	87.64433	76.90963	
The Netherlanc	Deaths	Male	2020	79.54322	85.6886	74.48494	
The Netherlanc	Deaths	Male	2021	78.74008	85.50977	73.14238	
The Netherlanc	Deaths	Male	2022	80.00836	87.48362	73.65411	
The Netherlanc	Deaths	Male	2023	80.99124	89.32383	73.49417	
The Netherlanc	Deaths	Female	2010	50.3695	53.25432	46.29097	
The Netherlanc	Deaths	Female	2011	52.6781	55.6813	48.26445	
The Netherlanc	Deaths	Female	2012	54.36451	57.4115	49.8831	
The Netherlanc	Deaths	Female	2013	55.95402	59.19476	50.90927	
The Netherlanc	Deaths	Female	2014	56.73786	59.97039	51.7351	
The Netherlanc	Deaths	Female	2015	58.01607	61.28266	52.98973	
The Netherlanc	Deaths	Female	2016	59.03885	62.77937	53.68932	
The Netherlanc	Deaths	Female	2017	58.91507	62.55976	53.457	
The Netherlanc	Deaths	Female	2018	59.75132	63.51148	53.93412	
The Netherlanc	Deaths	Female	2019	59.01264	62.75863	52.85411	
The Netherlanc	Deaths	Female	2020	58.4375	62.02979	53.14402	
The Netherlanc	Deaths	Female	2021	60.73066	64.36868	55.62527	
The Netherlanc	Deaths	Female	2022	62.52352	66.52301	56.95626	
The Netherlanc	Deaths	Female	2023	61.76559	66.34152	55.38567	
The Netherlanc	DALYs	Male	2010	1943.252	2039.417	1841.111	
The Netherlanc	DALYs	Male	2011	1926.591	2033.626	1818.148	
The Netherlanc	DALYs	Male	2012	1888.932	1992.686	1789.701	
The Netherlanc	DALYs	Male	2013	1874.488	1980.685	1779.25	
The Netherlanc	DALYs	Male	2014	1830.762	1943.662	1724.736	

The Netherland	DALYs	Male	2015	1813.182	1922.187	1699.48	
The Netherland	DALYs	Male	2016	1802.213	1926.843	1700.265	
The Netherland	DALYs	Male	2017	1769.568	1882.456	1664.636	
The Netherland	DALYs	Male	2018	1718.947	1832.545	1613.963	
The Netherland	DALYs	Male	2019	1656.915	1767.841	1558.281	
The Netherland	DALYs	Male	2020	1604.495	1724.815	1511.25	
The Netherland	DALYs	Male	2021	1588.694	1723.104	1485.652	
The Netherland	DALYs	Male	2022	1602.255	1740.483	1487.073	
The Netherland	DALYs	Male	2023	1590.083	1743.719	1464.761	
The Netherland	DALYs	Female	2010	1182.382	1244.084	1102.193	
The Netherland	DALYs	Female	2011	1231.354	1300.01	1143.063	
The Netherland	DALYs	Female	2012	1260.456	1326.775	1183.202	
The Netherland	DALYs	Female	2013	1284.787	1357.678	1189.293	
The Netherland	DALYs	Female	2014	1291.292	1356.744	1201.431	
The Netherland	DALYs	Female	2015	1299.972	1364.18	1205.058	
The Netherland	DALYs	Female	2016	1304.256	1371.72	1204.664	
The Netherland	DALYs	Female	2017	1282.775	1347.416	1187.76	
The Netherland	DALYs	Female	2018	1285.396	1354.097	1188.868	
The Netherland	DALYs	Female	2019	1254.703	1322.358	1149.064	
The Netherland	DALYs	Female	2020	1235.176	1302.445	1146.059	
The Netherland	DALYs	Female	2021	1281.406	1345.721	1196.866	
The Netherland	DALYs	Female	2022	1297.295	1369.601	1209.096	
The Netherland	DALYs	Female	2023	1263.049	1338.227	1161.541	
New Zealand	Incidence	Male	2010	49.62497	53.11677	46.28303	
New Zealand	Incidence	Male	2011	50.43439	53.5044	47.03766	
New Zealand	Incidence	Male	2012	50.53975	53.79805	47.13903	
New Zealand	Incidence	Male	2013	50.281	53.78233	46.42562	
New Zealand	Incidence	Male	2014	51.79614	55.41238	47.86322	
New Zealand	Incidence	Male	2015	51.9907	55.77889	47.88496	
New Zealand	Incidence	Male	2016	51.62649	55.90221	47.46967	
New Zealand	Incidence	Male	2017	49.62987	54.12646	45.19736	
New Zealand	Incidence	Male	2018	50.37653	55.43387	46.08646	
New Zealand	Incidence	Male	2019	49.95557	55.7295	45.70538	
New Zealand	Incidence	Male	2020	48.25588	54.12555	43.98691	
New Zealand	Incidence	Male	2021	50.28686	56.79348	45.70526	
New Zealand	Incidence	Male	2022	52.30818	59.72506	47.31086	

New Zealand	Incidence	Male	2023	54.89457	62.05427	49.04973	
New Zealand	Incidence	Female	2010	43.99533	47.95727	39.95136	
New Zealand	Incidence	Female	2011	44.85337	48.97813	41.00777	
New Zealand	Incidence	Female	2012	44.87094	49.06993	40.71369	
New Zealand	Incidence	Female	2013	46.24511	50.70962	41.95795	
New Zealand	Incidence	Female	2014	48.18946	52.72296	43.73209	
New Zealand	Incidence	Female	2015	49.81852	54.39183	44.70822	
New Zealand	Incidence	Female	2016	50.71557	55.52478	45.43716	
New Zealand	Incidence	Female	2017	52.66057	58.06664	46.77958	
New Zealand	Incidence	Female	2018	52.38886	57.95999	46.50123	
New Zealand	Incidence	Female	2019	52.16185	58.41661	46.09524	
New Zealand	Incidence	Female	2020	51.00732	57.60879	44.9015	
New Zealand	Incidence	Female	2021	53.74546	60.92359	46.82904	
New Zealand	Incidence	Female	2022	57.27232	65.36805	49.63385	
New Zealand	Incidence	Female	2023	61.00757	69.63949	52.98142	
New Zealand	Deaths	Male	2010	42.28967	44.60158	39.60378	
New Zealand	Deaths	Male	2011	42.74565	44.93939	40.01025	
New Zealand	Deaths	Male	2012	42.47408	44.37131	39.96092	
New Zealand	Deaths	Male	2013	41.98891	44.10497	39.50188	
New Zealand	Deaths	Male	2014	43.09142	45.56259	40.25919	
New Zealand	Deaths	Male	2015	43.00321	45.97272	39.92598	
New Zealand	Deaths	Male	2016	42.28665	45.3509	39.10144	
New Zealand	Deaths	Male	2017	40.47402	43.73934	37.17835	
New Zealand	Deaths	Male	2018	40.94989	44.6256	37.69415	
New Zealand	Deaths	Male	2019	40.42342	44.7212	37.18401	
New Zealand	Deaths	Male	2020	38.92033	43.2973	35.95167	
New Zealand	Deaths	Male	2021	40.5498	44.94396	37.15371	
New Zealand	Deaths	Male	2022	42.15916	46.96316	38.44154	
New Zealand	Deaths	Male	2023	44.17188	49.98223	40.01357	
New Zealand	Deaths	Female	2010	35.09828	37.43099	31.46588	
New Zealand	Deaths	Female	2011	35.48522	37.91166	31.95813	
New Zealand	Deaths	Female	2012	35.14714	37.45714	31.62511	
New Zealand	Deaths	Female	2013	35.76543	38.10408	32.06022	
New Zealand	Deaths	Female	2014	36.99745	39.58389	33.3323	
New Zealand	Deaths	Female	2015	37.82878	40.69151	34.30523	
New Zealand	Deaths	Female	2016	37.97948	40.98735	34.32095	

New Zealand	Deaths	Female	2017	39.1464	42.60995	35.25473	
New Zealand	Deaths	Female	2018	38.93571	42.82506	34.736	
New Zealand	Deaths	Female	2019	38.68761	43.06501	34.15679	
New Zealand	Deaths	Female	2020	37.47437	42.06338	33.11995	
New Zealand	Deaths	Female	2021	39.52802	44.87402	34.4505	
New Zealand	Deaths	Female	2022	42.0591	48.03334	36.87159	
New Zealand	Deaths	Female	2023	44.53848	51.21742	38.91247	
New Zealand	DALYs	Male	2010	910.7509	962.733	858.8276	
New Zealand	DALYs	Male	2011	909.1374	959.7178	856.8288	
New Zealand	DALYs	Male	2012	904.3765	946.3197	854.1532	
New Zealand	DALYs	Male	2013	890.1602	931.4947	840.8919	
New Zealand	DALYs	Male	2014	911.1662	963.7773	855.9729	
New Zealand	DALYs	Male	2015	909.1145	968.0791	849.234	
New Zealand	DALYs	Male	2016	889.916	946.5163	828.8476	
New Zealand	DALYs	Male	2017	847.3481	907.5313	785.2914	
New Zealand	DALYs	Male	2018	851.4242	924.3356	790.0407	
New Zealand	DALYs	Male	2019	846.3572	931.9897	782.1315	
New Zealand	DALYs	Male	2020	814.6008	902.2614	759.0856	
New Zealand	DALYs	Male	2021	843.7963	929.3215	776.4864	
New Zealand	DALYs	Male	2022	879.2292	972.0061	802.7712	
New Zealand	DALYs	Male	2023	912.3792	1031.374	833.5557	
New Zealand	DALYs	Female	2010	786.1571	837.5268	718.3199	
New Zealand	DALYs	Female	2011	789.0162	837.0314	726.016	
New Zealand	DALYs	Female	2012	775.6347	820.6299	717.8763	
New Zealand	DALYs	Female	2013	791.8671	839.5972	733.6697	
New Zealand	DALYs	Female	2014	816.8978	868.0458	755.696	
New Zealand	DALYs	Female	2015	832.8116	886.674	767.2807	
New Zealand	DALYs	Female	2016	832.9317	896.4264	764.5959	
New Zealand	DALYs	Female	2017	854.7917	930.7628	785.2845	
New Zealand	DALYs	Female	2018	843.2332	920.5269	767.1875	
New Zealand	DALYs	Female	2019	832.3525	927.1735	749.7134	
New Zealand	DALYs	Female	2020	811.2575	903.6423	734.6582	
New Zealand	DALYs	Female	2021	841.1998	948.2859	755.919	
New Zealand	DALYs	Female	2022	893.6737	1018.884	788.8385	
New Zealand	DALYs	Female	2023	947.1682	1075.952	844.9866	
Nicaragua	Incidence	Male	2010	4.869623	5.90674	3.734793	

Nicaragua	Incidence	Male	2011	4.967015	6.042628	3.843984	
Nicaragua	Incidence	Male	2012	5.015552	6.120704	3.952866	
Nicaragua	Incidence	Male	2013	5.100472	6.239285	4.141974	
Nicaragua	Incidence	Male	2014	5.180058	6.314292	4.21515	
Nicaragua	Incidence	Male	2015	5.298007	6.54173	4.357977	
Nicaragua	Incidence	Male	2016	5.267296	6.460734	4.396514	
Nicaragua	Incidence	Male	2017	5.396687	6.887706	4.430456	
Nicaragua	Incidence	Male	2018	5.629143	7.342811	4.615287	
Nicaragua	Incidence	Male	2019	6.161768	8.084626	5.053148	
Nicaragua	Incidence	Male	2020	6.371149	8.319066	5.201633	
Nicaragua	Incidence	Male	2021	6.354213	8.333751	5.117384	
Nicaragua	Incidence	Male	2022	5.73635	7.771745	4.435868	
Nicaragua	Incidence	Male	2023	5.254421	6.894645	3.94264	
Nicaragua	Incidence	Female	2010	3.468746	4.522252	2.757886	
Nicaragua	Incidence	Female	2011	3.568781	4.644473	2.789675	
Nicaragua	Incidence	Female	2012	3.663755	4.676659	2.912874	
Nicaragua	Incidence	Female	2013	3.780221	4.793228	3.088937	
Nicaragua	Incidence	Female	2014	3.914718	4.86068	3.208211	
Nicaragua	Incidence	Female	2015	4.006888	4.832042	3.318501	
Nicaragua	Incidence	Female	2016	4.081269	5.003573	3.384922	
Nicaragua	Incidence	Female	2017	4.182099	5.039329	3.523846	
Nicaragua	Incidence	Female	2018	4.380645	5.380861	3.682922	
Nicaragua	Incidence	Female	2019	4.803598	6.036013	4.075081	
Nicaragua	Incidence	Female	2020	4.913533	6.083561	4.151723	
Nicaragua	Incidence	Female	2021	5.344625	6.859823	4.437626	
Nicaragua	Incidence	Female	2022	5.142489	6.852875	4.02875	
Nicaragua	Incidence	Female	2023	4.742459	6.429331	3.67822	
Nicaragua	Deaths	Male	2010	4.84971	5.892328	3.675644	
Nicaragua	Deaths	Male	2011	4.938964	6.031411	3.827945	
Nicaragua	Deaths	Male	2012	4.98297	6.047244	3.886182	
Nicaragua	Deaths	Male	2013	5.062733	6.116324	4.055935	
Nicaragua	Deaths	Male	2014	5.14237	6.195102	4.156075	
Nicaragua	Deaths	Male	2015	5.249078	6.44838	4.285702	
Nicaragua	Deaths	Male	2016	5.208384	6.407296	4.278163	
Nicaragua	Deaths	Male	2017	5.328608	6.77966	4.365725	
Nicaragua	Deaths	Male	2018	5.547172	7.247446	4.571306	

Nicaragua	Deaths	Male	2019	6.047516	7.902559	4.991399	
Nicaragua	Deaths	Male	2020	6.24578	8.101988	5.106407	
Nicaragua	Deaths	Male	2021	6.194456	8.143913	4.988556	
Nicaragua	Deaths	Male	2022	5.553637	7.542504	4.252896	
Nicaragua	Deaths	Male	2023	5.066662	6.536945	3.776991	
Nicaragua	Deaths	Female	2010	3.256633	4.240238	2.592785	
Nicaragua	Deaths	Female	2011	3.340948	4.32476	2.626393	
Nicaragua	Deaths	Female	2012	3.421522	4.355377	2.733785	
Nicaragua	Deaths	Female	2013	3.52167	4.418231	2.891747	
Nicaragua	Deaths	Female	2014	3.642805	4.510964	2.993016	
Nicaragua	Deaths	Female	2015	3.713131	4.47992	3.061553	
Nicaragua	Deaths	Female	2016	3.765347	4.545742	3.145977	
Nicaragua	Deaths	Female	2017	3.843314	4.576536	3.242323	
Nicaragua	Deaths	Female	2018	4.006352	4.895615	3.414454	
Nicaragua	Deaths	Female	2019	4.357694	5.298318	3.687979	
Nicaragua	Deaths	Female	2020	4.420021	5.472422	3.764698	
Nicaragua	Deaths	Female	2021	4.748527	5.97839	3.964992	
Nicaragua	Deaths	Female	2022	4.536684	5.996522	3.59404	
Nicaragua	Deaths	Female	2023	4.177256	5.662049	3.262161	
Nicaragua	DALYs	Male	2010	122.93	152.4982	95.22111	
Nicaragua	DALYs	Male	2011	125.2635	154.4778	98.85091	
Nicaragua	DALYs	Male	2012	126.3098	157.2967	101.0027	
Nicaragua	DALYs	Male	2013	128.2005	157.738	103.8452	
Nicaragua	DALYs	Male	2014	129.4246	159.5172	105.2121	
Nicaragua	DALYs	Male	2015	132.0588	163.6733	109.5825	
Nicaragua	DALYs	Male	2016	130.9893	164.7195	109.7474	
Nicaragua	DALYs	Male	2017	133.6002	174.5661	109.6116	
Nicaragua	DALYs	Male	2018	138.8054	185.7994	114.7677	
Nicaragua	DALYs	Male	2019	151.242	203.0955	124.8613	
Nicaragua	DALYs	Male	2020	154.4636	204.9142	127.1111	
Nicaragua	DALYs	Male	2021	154.3469	206.751	125.3366	
Nicaragua	DALYs	Male	2022	141.0496	195.2147	108.7111	
Nicaragua	DALYs	Male	2023	129.6759	169.0196	99.16124	
Nicaragua	DALYs	Female	2010	83.77235	109.0655	67.00017	
Nicaragua	DALYs	Female	2011	86.00978	110.535	67.95316	
Nicaragua	DALYs	Female	2012	88.15188	112.3671	69.85747	

Nicaragua	DALYs	Female	2013	90.64827	114.848	73.29553	
Nicaragua	DALYs	Female	2014	93.24129	114.6593	76.28357	
Nicaragua	DALYs	Female	2015	94.95058	114.679	78.29936	
Nicaragua	DALYs	Female	2016	96.25978	117.3524	80.16206	
Nicaragua	DALYs	Female	2017	98.0585	119.0428	83.23105	
Nicaragua	DALYs	Female	2018	102.1157	125.2791	87.0446	
Nicaragua	DALYs	Female	2019	110.9298	135.6818	94.85639	
Nicaragua	DALYs	Female	2020	111.6939	137.6682	96.17674	
Nicaragua	DALYs	Female	2021	121.2819	156.0035	102.3421	
Nicaragua	DALYs	Female	2022	117.2271	152.6416	94.22736	
Nicaragua	DALYs	Female	2023	107.4343	143.584	84.59774	
Niger	Incidence	Male	2010	0.767859	1.14128	0.499923	
Niger	Incidence	Male	2011	0.767503	1.130775	0.490362	
Niger	Incidence	Male	2012	0.765314	1.086728	0.495797	
Niger	Incidence	Male	2013	0.750953	1.057296	0.501311	
Niger	Incidence	Male	2014	0.741777	1.067975	0.478009	
Niger	Incidence	Male	2015	0.840713	1.23802	0.525144	
Niger	Incidence	Male	2016	0.84117	1.196384	0.540217	
Niger	Incidence	Male	2017	0.811342	1.165166	0.523496	
Niger	Incidence	Male	2018	0.817728	1.171038	0.542744	
Niger	Incidence	Male	2019	0.793488	1.19463	0.536376	
Niger	Incidence	Male	2020	0.838906	1.266117	0.575096	
Niger	Incidence	Male	2021	0.804185	1.187593	0.547117	
Niger	Incidence	Male	2022	0.857641	1.28973	0.564758	
Niger	Incidence	Male	2023	0.920869	1.374331	0.597824	
Niger	Incidence	Female	2010	0.210906	0.310256	0.142953	
Niger	Incidence	Female	2011	0.209807	0.305068	0.140611	
Niger	Incidence	Female	2012	0.210658	0.307747	0.138853	
Niger	Incidence	Female	2013	0.210228	0.31616	0.135888	
Niger	Incidence	Female	2014	0.208753	0.310777	0.132675	
Niger	Incidence	Female	2015	0.221446	0.315816	0.145548	
Niger	Incidence	Female	2016	0.224763	0.314084	0.147306	
Niger	Incidence	Female	2017	0.229363	0.316889	0.150829	
Niger	Incidence	Female	2018	0.234926	0.321847	0.155989	
Niger	Incidence	Female	2019	0.238663	0.316075	0.159802	
Niger	Incidence	Female	2020	0.234793	0.309614	0.156387	

Niger	Incidence	Female	2021	0.241937	0.316156	0.164833	
Niger	Incidence	Female	2022	0.272189	0.369778	0.181498	
Niger	Incidence	Female	2023	0.287688	0.387705	0.193579	
Niger	Deaths	Male	2010	0.768176	1.136655	0.499312	
Niger	Deaths	Male	2011	0.76731	1.127154	0.489712	
Niger	Deaths	Male	2012	0.765799	1.091103	0.49893	
Niger	Deaths	Male	2013	0.75276	1.056379	0.504749	
Niger	Deaths	Male	2014	0.742669	1.065136	0.485117	
Niger	Deaths	Male	2015	0.841435	1.235655	0.528416	
Niger	Deaths	Male	2016	0.841254	1.196594	0.539364	
Niger	Deaths	Male	2017	0.813041	1.16622	0.523947	
Niger	Deaths	Male	2018	0.821653	1.178069	0.543457	
Niger	Deaths	Male	2019	0.797883	1.199347	0.540587	
Niger	Deaths	Male	2020	0.846064	1.274761	0.579434	
Niger	Deaths	Male	2021	0.809978	1.196645	0.55008	
Niger	Deaths	Male	2022	0.862817	1.297546	0.570816	
Niger	Deaths	Male	2023	0.92727	1.385876	0.599224	
Niger	Deaths	Female	2010	0.20283	0.299827	0.137516	
Niger	Deaths	Female	2011	0.201623	0.293205	0.13477	
Niger	Deaths	Female	2012	0.202355	0.295365	0.132682	
Niger	Deaths	Female	2013	0.201852	0.301888	0.131519	
Niger	Deaths	Female	2014	0.200028	0.297205	0.127607	
Niger	Deaths	Female	2015	0.211905	0.302936	0.138751	
Niger	Deaths	Female	2016	0.214528	0.297819	0.140521	
Niger	Deaths	Female	2017	0.219014	0.301541	0.144908	
Niger	Deaths	Female	2018	0.224085	0.308252	0.150086	
Niger	Deaths	Female	2019	0.227452	0.301164	0.153929	
Niger	Deaths	Female	2020	0.223643	0.294475	0.150363	
Niger	Deaths	Female	2021	0.229645	0.29748	0.157026	
Niger	Deaths	Female	2022	0.258044	0.348742	0.1719	
Niger	Deaths	Female	2023	0.271918	0.366016	0.183954	
Niger	DALYs	Male	2010	20.78551	31.34057	13.6055	
Niger	DALYs	Male	2011	20.77349	30.93175	13.37035	
Niger	DALYs	Male	2012	20.67411	29.38495	13.30269	
Niger	DALYs	Male	2013	20.23172	28.8359	13.30202	
Niger	DALYs	Male	2014	19.98627	28.62204	12.75222	

Niger	DALYs	Male	2015	22.64702	33.15771	14.09637	
Niger	DALYs	Male	2016	22.64164	32.04829	14.40668	
Niger	DALYs	Male	2017	21.78818	30.95914	14.0749	
Niger	DALYs	Male	2018	21.9093	31.51449	14.59048	
Niger	DALYs	Male	2019	21.18447	32.02204	14.40301	
Niger	DALYs	Male	2020	22.25014	33.74963	15.28862	
Niger	DALYs	Male	2021	21.34545	31.61078	14.54625	
Niger	DALYs	Male	2022	22.78014	34.23966	14.93802	
Niger	DALYs	Male	2023	24.4622	36.56056	15.90494	
Niger	DALYs	Female	2010	6.569076	9.652645	4.449914	
Niger	DALYs	Female	2011	6.538176	9.478055	4.394236	
Niger	DALYs	Female	2012	6.573659	9.527747	4.355107	
Niger	DALYs	Female	2013	6.570556	10.03894	4.227033	
Niger	DALYs	Female	2014	6.54369	9.854241	4.138721	
Niger	DALYs	Female	2015	6.962646	10.02295	4.480808	
Niger	DALYs	Female	2016	7.096149	10.13211	4.654646	
Niger	DALYs	Female	2017	7.247222	10.12393	4.724186	
Niger	DALYs	Female	2018	7.441215	10.23317	4.839182	
Niger	DALYs	Female	2019	7.564862	10.06358	4.917158	
Niger	DALYs	Female	2020	7.449158	9.825339	4.916241	
Niger	DALYs	Female	2021	7.722219	10.25659	5.246646	
Niger	DALYs	Female	2022	8.680399	11.97713	5.699489	
Niger	DALYs	Female	2023	9.227316	12.74685	6.186558	
Nigeria	Incidence	Male	2010	3.421907	5.032662	2.338945	
Nigeria	Incidence	Male	2011	3.434324	5.144929	2.271478	
Nigeria	Incidence	Male	2012	3.48829	5.208238	2.365202	
Nigeria	Incidence	Male	2013	3.445295	4.994796	2.338943	
Nigeria	Incidence	Male	2014	3.461879	5.116127	2.321913	
Nigeria	Incidence	Male	2015	3.715312	5.463128	2.452253	
Nigeria	Incidence	Male	2016	3.586065	5.280674	2.316968	
Nigeria	Incidence	Male	2017	3.566646	5.250581	2.306003	
Nigeria	Incidence	Male	2018	3.481356	5.276296	2.247788	
Nigeria	Incidence	Male	2019	3.431493	5.236246	2.186023	
Nigeria	Incidence	Male	2020	3.563439	5.400076	2.279994	
Nigeria	Incidence	Male	2021	3.580246	5.691055	2.313776	
Nigeria	Incidence	Male	2022	3.812776	5.796936	2.349311	

Nigeria	Incidence	Male	2023	3.939299	6.032967	2.395092	
Nigeria	Incidence	Female	2010	1.067004	1.540354	0.684054	
Nigeria	Incidence	Female	2011	1.090086	1.5346	0.676455	
Nigeria	Incidence	Female	2012	1.124943	1.576327	0.700263	
Nigeria	Incidence	Female	2013	1.176184	1.690258	0.730915	
Nigeria	Incidence	Female	2014	1.213946	1.74605	0.7684	
Nigeria	Incidence	Female	2015	1.280288	1.848138	0.836833	
Nigeria	Incidence	Female	2016	1.293425	1.885518	0.831015	
Nigeria	Incidence	Female	2017	1.322667	1.888461	0.875543	
Nigeria	Incidence	Female	2018	1.336266	1.903976	0.900408	
Nigeria	Incidence	Female	2019	1.354892	1.943251	0.915424	
Nigeria	Incidence	Female	2020	1.390222	1.955939	0.972104	
Nigeria	Incidence	Female	2021	1.44848	2.022441	1.022076	
Nigeria	Incidence	Female	2022	1.6531	2.305204	1.117657	
Nigeria	Incidence	Female	2023	1.754097	2.514937	1.192775	
Nigeria	Deaths	Male	2010	3.409899	5.03618	2.328506	
Nigeria	Deaths	Male	2011	3.423863	5.147086	2.274367	
Nigeria	Deaths	Male	2012	3.479076	5.18766	2.364062	
Nigeria	Deaths	Male	2013	3.436491	5.004117	2.328775	
Nigeria	Deaths	Male	2014	3.448156	5.104475	2.309328	
Nigeria	Deaths	Male	2015	3.699555	5.458825	2.431492	
Nigeria	Deaths	Male	2016	3.568923	5.271388	2.308535	
Nigeria	Deaths	Male	2017	3.549779	5.260499	2.274436	
Nigeria	Deaths	Male	2018	3.465313	5.256124	2.234539	
Nigeria	Deaths	Male	2019	3.415022	5.217747	2.183109	
Nigeria	Deaths	Male	2020	3.546696	5.385889	2.276068	
Nigeria	Deaths	Male	2021	3.555629	5.674487	2.305229	
Nigeria	Deaths	Male	2022	3.775318	5.764077	2.317953	
Nigeria	Deaths	Male	2023	3.89677	6.002919	2.355492	
Nigeria	Deaths	Female	2010	1.036672	1.496347	0.659218	
Nigeria	Deaths	Female	2011	1.05777	1.497768	0.654029	
Nigeria	Deaths	Female	2012	1.089599	1.53919	0.676431	
Nigeria	Deaths	Female	2013	1.136568	1.644678	0.705283	
Nigeria	Deaths	Female	2014	1.168939	1.68853	0.734636	
Nigeria	Deaths	Female	2015	1.230862	1.766893	0.810393	
Nigeria	Deaths	Female	2016	1.243437	1.819066	0.805893	

Nigeria	Deaths	Female	2017	1.272541	1.82011	0.848067	
Nigeria	Deaths	Female	2018	1.286796	1.841492	0.86911	
Nigeria	Deaths	Female	2019	1.305057	1.869709	0.886562	
Nigeria	Deaths	Female	2020	1.339035	1.890631	0.939275	
Nigeria	Deaths	Female	2021	1.392667	1.945364	0.983743	
Nigeria	Deaths	Female	2022	1.584255	2.217277	1.068825	
Nigeria	Deaths	Female	2023	1.678905	2.399024	1.140832	
Nigeria	DALYs	Male	2010	93.85163	136.4305	63.82159	
Nigeria	DALYs	Male	2011	94.10377	140.9608	61.35644	
Nigeria	DALYs	Male	2012	95.56024	141.4871	64.05772	
Nigeria	DALYs	Male	2013	94.43185	136.2705	63.83772	
Nigeria	DALYs	Male	2014	95.22073	140.4565	64.00694	
Nigeria	DALYs	Male	2015	102.3517	150.1714	66.84091	
Nigeria	DALYs	Male	2016	98.80061	143.7639	64.36533	
Nigeria	DALYs	Male	2017	98.23419	144.6101	63.92229	
Nigeria	DALYs	Male	2018	95.84392	144.3986	61.85202	
Nigeria	DALYs	Male	2019	94.45052	143.1536	60.22943	
Nigeria	DALYs	Male	2020	97.90732	146.9529	62.91304	
Nigeria	DALYs	Male	2021	98.62703	156.9517	63.2279	
Nigeria	DALYs	Male	2022	105.575	161.4201	65.37857	
Nigeria	DALYs	Male	2023	109.1941	163.0109	66.7611	
Nigeria	DALYs	Female	2010	32.2555	47.02142	20.73715	
Nigeria	DALYs	Female	2011	32.91643	46.46756	20.62123	
Nigeria	DALYs	Female	2012	34.01906	47.51388	21.44902	
Nigeria	DALYs	Female	2013	35.66591	50.82022	22.32023	
Nigeria	DALYs	Female	2014	37.00323	53.087	23.88475	
Nigeria	DALYs	Female	2015	39.05729	56.9852	25.23596	
Nigeria	DALYs	Female	2016	39.30492	57.38644	25.50975	
Nigeria	DALYs	Female	2017	40.04351	57.58884	26.53544	
Nigeria	DALYs	Female	2018	40.29219	57.1319	27.08129	
Nigeria	DALYs	Female	2019	40.73803	57.11697	27.65413	
Nigeria	DALYs	Female	2020	41.60384	57.48035	28.77126	
Nigeria	DALYs	Female	2021	43.30508	59.82206	30.0166	
Nigeria	DALYs	Female	2022	49.60977	69.22212	33.75021	
Nigeria	DALYs	Female	2023	52.65083	75.43955	35.61557	
North Macedor	Incidence	Male	2010	75.30303	86.39438	61.09535	

North Macedor	Incidence	Male	2011	78.32342	90.02438	63.02265	
North Macedor	Incidence	Male	2012	80.13784	91.26233	65.1864	
North Macedor	Incidence	Male	2013	81.33502	92.30678	66.11302	
North Macedor	Incidence	Male	2014	83.3447	95.67265	67.04308	
North Macedor	Incidence	Male	2015	84.75321	98.16953	67.64583	
North Macedor	Incidence	Male	2016	85.72244	98.96796	68.74734	
North Macedor	Incidence	Male	2017	88.48029	100.6286	71.4698	
North Macedor	Incidence	Male	2018	90.59275	103.5762	74.19835	
North Macedor	Incidence	Male	2019	96.65387	109.2935	79.34925	
North Macedor	Incidence	Male	2020	96.09397	108.0215	78.32724	
North Macedor	Incidence	Male	2021	92.85842	105.886	76.00427	
North Macedor	Incidence	Male	2022	94.1404	106.3846	77.64334	
North Macedor	Incidence	Male	2023	91.61157	106.7823	73.70673	
North Macedor	Incidence	Female	2010	18.46337	21.8681	15.63079	
North Macedor	Incidence	Female	2011	19.59638	23.24523	16.69815	
North Macedor	Incidence	Female	2012	20.45881	23.89396	16.9431	
North Macedor	Incidence	Female	2013	21.04539	24.56473	17.3199	
North Macedor	Incidence	Female	2014	22.14428	25.8784	17.90756	
North Macedor	Incidence	Female	2015	23.40713	27.54371	18.33806	
North Macedor	Incidence	Female	2016	24.47582	28.5907	18.73374	
North Macedor	Incidence	Female	2017	25.87778	29.85463	19.30361	
North Macedor	Incidence	Female	2018	26.83589	30.88818	19.71666	
North Macedor	Incidence	Female	2019	28.84805	33.92887	21.25464	
North Macedor	Incidence	Female	2020	29.01534	35.06372	20.73554	
North Macedor	Incidence	Female	2021	28.33913	34.8773	20.25922	
North Macedor	Incidence	Female	2022	28.87577	35.58078	20.77807	
North Macedor	Incidence	Female	2023	28.08183	34.94231	21.09811	
North Macedor	Deaths	Male	2010	72.44127	83.09054	59.45557	
North Macedor	Deaths	Male	2011	75.25974	86.27872	61.03134	
North Macedor	Deaths	Male	2012	77.17145	88.07805	62.462	
North Macedor	Deaths	Male	2013	78.16082	88.83417	63.34283	
North Macedor	Deaths	Male	2014	80.06177	91.93888	64.20096	
North Macedor	Deaths	Male	2015	81.53333	94.38177	64.45262	
North Macedor	Deaths	Male	2016	82.46095	95.06905	65.58089	
North Macedor	Deaths	Male	2017	84.98803	96.55693	68.13888	
North Macedor	Deaths	Male	2018	86.68394	99.13228	70.04649	

North Macedor	Deaths	Male	2019	92.23277	104.7737	75.19984	
North Macedor	Deaths	Male	2020	91.84498	104.1958	74.44478	
North Macedor	Deaths	Male	2021	88.42677	101.1431	72.15069	
North Macedor	Deaths	Male	2022	89.72247	101.9492	73.66724	
North Macedor	Deaths	Male	2023	87.6261	102.2211	69.5833	
North Macedor	Deaths	Female	2010	17.80119	20.92757	15.18314	
North Macedor	Deaths	Female	2011	18.88622	22.34096	16.21216	
North Macedor	Deaths	Female	2012	19.75456	23.03656	16.65666	
North Macedor	Deaths	Female	2013	20.24673	23.64933	16.87021	
North Macedor	Deaths	Female	2014	21.33928	24.96535	17.43384	
North Macedor	Deaths	Female	2015	22.56572	26.44234	17.86601	
North Macedor	Deaths	Female	2016	23.5108	27.32883	18.14709	
North Macedor	Deaths	Female	2017	24.80638	28.79323	18.66784	
North Macedor	Deaths	Female	2018	25.55674	29.16673	18.94205	
North Macedor	Deaths	Female	2019	27.4296	31.92773	20.31945	
North Macedor	Deaths	Female	2020	27.62183	33.13491	19.85014	
North Macedor	Deaths	Female	2021	26.88099	33.1451	19.19888	
North Macedor	Deaths	Female	2022	27.44049	33.60649	19.88493	
North Macedor	Deaths	Female	2023	26.82886	33.48241	20.2378	
North Macedor	DALYs	Male	2010	1931.596	2223.385	1549.212	
North Macedor	DALYs	Male	2011	1996.745	2286.663	1598.384	
North Macedor	DALYs	Male	2012	2025.139	2315.152	1633.417	
North Macedor	DALYs	Male	2013	2053.888	2328.929	1661.589	
North Macedor	DALYs	Male	2014	2094.066	2404.727	1714.027	
North Macedor	DALYs	Male	2015	2110.371	2443.221	1731.236	
North Macedor	DALYs	Male	2016	2118.947	2451.78	1742.171	
North Macedor	DALYs	Male	2017	2171.21	2475.041	1803.127	
North Macedor	DALYs	Male	2018	2216.432	2538.883	1829.382	
North Macedor	DALYs	Male	2019	2349.638	2675.2	1936.477	
North Macedor	DALYs	Male	2020	2294.774	2643.646	1873.913	
North Macedor	DALYs	Male	2021	2208.524	2548.647	1827.833	
North Macedor	DALYs	Male	2022	2223.381	2547.686	1837.35	
North Macedor	DALYs	Male	2023	2133.2	2486.392	1712.519	
North Macedor	DALYs	Female	2010	464.9303	550.093	395.2044	
North Macedor	DALYs	Female	2011	489.5725	571.8275	413.3806	
North Macedor	DALYs	Female	2012	506.999	587.4305	410.5089	

North Macedor	DALYs	Female	2013	522.4241	604.7386	422.9422	
North Macedor	DALYs	Female	2014	545.2859	639.168	431.5962	
North Macedor	DALYs	Female	2015	572.6273	669.1038	439.8406	
North Macedor	DALYs	Female	2016	597.4756	695.9031	447.0488	
North Macedor	DALYs	Female	2017	627.9993	718.2958	455.4572	
North Macedor	DALYs	Female	2018	651.9099	742.7162	466.3396	
North Macedor	DALYs	Female	2019	694.041	805.0608	497.9212	
North Macedor	DALYs	Female	2020	686.1583	826.031	483.1447	
North Macedor	DALYs	Female	2021	666.2658	820.1617	468.8477	
North Macedor	DALYs	Female	2022	674.4327	834.7667	474.5471	
North Macedor	DALYs	Female	2023	646.1271	797.5144	476.5712	
Norway	Incidence	Male	2010	71.65098	80.63808	64.70014	
Norway	Incidence	Male	2011	72.53753	81.71105	65.21318	
Norway	Incidence	Male	2012	71.22505	80.35172	63.83133	
Norway	Incidence	Male	2013	69.86118	78.52726	62.5159	
Norway	Incidence	Male	2014	68.44212	78.01164	61.32198	
Norway	Incidence	Male	2015	66.54507	75.56927	59.36954	
Norway	Incidence	Male	2016	65.28437	74.38889	57.84658	
Norway	Incidence	Male	2017	64.64079	73.65678	57.43123	
Norway	Incidence	Male	2018	63.92343	73.29732	56.44336	
Norway	Incidence	Male	2019	61.87169	70.8814	54.17205	
Norway	Incidence	Male	2020	61.574	70.42345	53.77696	
Norway	Incidence	Male	2021	59.86401	67.5991	52.51612	
Norway	Incidence	Male	2022	60.75988	68.06174	53.42161	
Norway	Incidence	Male	2023	62.64826	70.672	54.71364	
Norway	Incidence	Female	2010	54.73817	63.29438	47.46031	
Norway	Incidence	Female	2011	55.29365	65.15087	48.01599	
Norway	Incidence	Female	2012	56.68158	66.21688	49.14837	
Norway	Incidence	Female	2013	57.02295	66.65502	49.34935	
Norway	Incidence	Female	2014	56.77468	66.12352	48.51778	
Norway	Incidence	Female	2015	57.33051	66.67439	48.93026	
Norway	Incidence	Female	2016	58.50122	67.85906	49.77779	
Norway	Incidence	Female	2017	57.03357	66.12857	48.23508	
Norway	Incidence	Female	2018	58.73828	67.67557	49.31993	
Norway	Incidence	Female	2019	58.59636	67.27454	49.57911	
Norway	Incidence	Female	2020	56.24482	63.86893	47.50567	

Norway	Incidence	Female	2021	53.55895	60.83339	45.38759	
Norway	Incidence	Female	2022	52.7124	60.66845	45.02347	
Norway	Incidence	Female	2023	54.68722	63.56644	46.71646	
Norway	Deaths	Male	2010	57.65777	63.13758	52.85072	
Norway	Deaths	Male	2011	58.17071	63.83017	53.1958	
Norway	Deaths	Male	2012	56.93849	62.36395	52.05899	
Norway	Deaths	Male	2013	55.42902	61.24185	50.22883	
Norway	Deaths	Male	2014	53.98569	59.57927	49.08181	
Norway	Deaths	Male	2015	52.33448	57.51253	47.84133	
Norway	Deaths	Male	2016	51.24767	56.11413	46.82091	
Norway	Deaths	Male	2017	50.49957	55.58889	45.98148	
Norway	Deaths	Male	2018	49.61857	54.23934	45.18027	
Norway	Deaths	Male	2019	47.90327	52.27962	43.08503	
Norway	Deaths	Male	2020	47.61357	51.86476	42.93966	
Norway	Deaths	Male	2021	46.2566	50.5176	42.04236	
Norway	Deaths	Male	2022	47.05314	51.87771	42.76332	
Norway	Deaths	Male	2023	48.49591	53.36816	44.08577	
Norway	Deaths	Female	2010	42.29194	48.27687	36.60803	
Norway	Deaths	Female	2011	42.4746	48.37482	36.82682	
Norway	Deaths	Female	2012	43.44135	49.48461	37.70783	
Norway	Deaths	Female	2013	43.31465	49.09419	37.46617	
Norway	Deaths	Female	2014	42.93324	48.40781	37.33186	
Norway	Deaths	Female	2015	43.35745	48.70496	37.99185	
Norway	Deaths	Female	2016	44.19545	50.3444	38.79688	
Norway	Deaths	Female	2017	42.94532	48.35983	37.53446	
Norway	Deaths	Female	2018	44.18301	49.40239	38.70714	
Norway	Deaths	Female	2019	43.82814	48.89859	38.27362	
Norway	Deaths	Female	2020	41.99833	46.64224	36.87418	
Norway	Deaths	Female	2021	39.89205	44.36122	35.2153	
Norway	Deaths	Female	2022	39.16549	43.54382	34.79405	
Norway	Deaths	Female	2023	40.5973	46.11929	35.95644	
Norway	DALYs	Male	2010	1240.459	1358.366	1142.436	
Norway	DALYs	Male	2011	1240.928	1360.883	1144.668	
Norway	DALYs	Male	2012	1207.196	1325.641	1103.882	
Norway	DALYs	Male	2013	1172.225	1293.86	1070.926	
Norway	DALYs	Male	2014	1139.956	1254.844	1049.226	

Norway	DALYs	Male	2015	1093.056	1198.735	1003.06	
Norway	DALYs	Male	2016	1056.491	1152.542	974.7153	
Norway	DALYs	Male	2017	1034.663	1140.339	952.166	
Norway	DALYs	Male	2018	1013.136	1106.276	930.8329	
Norway	DALYs	Male	2019	969.262	1060.111	882.5927	
Norway	DALYs	Male	2020	960.3652	1045.147	874.9458	
Norway	DALYs	Male	2021	934.2641	1022.59	859.6843	
Norway	DALYs	Male	2022	950.111	1048.427	870.2925	
Norway	DALYs	Male	2023	979.3193	1076.472	888.3209	
Norway	DALYs	Female	2010	903.1352	1020.88	793.9861	
Norway	DALYs	Female	2011	905.6263	1035.603	800.9098	
Norway	DALYs	Female	2012	913.3289	1043.404	814.9216	
Norway	DALYs	Female	2013	910.5981	1032.723	811.0748	
Norway	DALYs	Female	2014	900.3291	1008.145	798.7888	
Norway	DALYs	Female	2015	897.7789	1004.793	799.2724	
Norway	DALYs	Female	2016	901.7766	1021.459	804.4296	
Norway	DALYs	Female	2017	871.7491	973.3641	776.9978	
Norway	DALYs	Female	2018	883.3257	983.3701	787.5281	
Norway	DALYs	Female	2019	878.765	977.6241	788.5497	
Norway	DALYs	Female	2020	841.3791	929.3544	756.6852	
Norway	DALYs	Female	2021	804.2435	889.9406	726.8683	
Norway	DALYs	Female	2022	798.768	879.7243	719.4773	
Norway	DALYs	Female	2023	833.7322	936.1592	742.4547	
Oman	Incidence	Male	2010	5.607943	7.224976	4.358921	
Oman	Incidence	Male	2011	5.766856	7.287189	4.47851	
Oman	Incidence	Male	2012	5.864797	7.42308	4.601623	
Oman	Incidence	Male	2013	5.647975	7.254368	4.443814	
Oman	Incidence	Male	2014	5.424583	6.901927	4.201696	
Oman	Incidence	Male	2015	5.252623	6.626311	4.012333	
Oman	Incidence	Male	2016	5.147291	6.618618	3.794446	
Oman	Incidence	Male	2017	5.100027	6.671333	3.694925	
Oman	Incidence	Male	2018	4.948867	6.708761	3.477783	
Oman	Incidence	Male	2019	4.794322	6.630622	3.339943	
Oman	Incidence	Male	2020	5.057992	7.077858	3.61155	
Oman	Incidence	Male	2021	5.36283	7.609362	3.793111	
Oman	Incidence	Male	2022	4.88892	7.256065	3.367288	

Oman	Incidence	Male	2023	5.144399	7.686008	3.561921	
Oman	Incidence	Female	2010	1.917188	2.680074	1.354659	
Oman	Incidence	Female	2011	1.974383	2.765715	1.360536	
Oman	Incidence	Female	2012	2.009715	2.736148	1.381245	
Oman	Incidence	Female	2013	2.034718	2.784929	1.400179	
Oman	Incidence	Female	2014	2.04872	2.842323	1.367452	
Oman	Incidence	Female	2015	2.037046	2.80844	1.348436	
Oman	Incidence	Female	2016	2.019068	2.760087	1.376859	
Oman	Incidence	Female	2017	2.048104	2.826756	1.415009	
Oman	Incidence	Female	2018	2.055342	2.810122	1.44099	
Oman	Incidence	Female	2019	2.058681	2.793393	1.456004	
Oman	Incidence	Female	2020	2.063043	2.758897	1.476614	
Oman	Incidence	Female	2021	2.260347	3.105547	1.609604	
Oman	Incidence	Female	2022	2.182173	3.030563	1.509752	
Oman	Incidence	Female	2023	2.274389	3.06928	1.546502	
Oman	Deaths	Male	2010	5.360862	6.932007	4.190021	
Oman	Deaths	Male	2011	5.501687	6.993095	4.303811	
Oman	Deaths	Male	2012	5.571297	7.076235	4.399728	
Oman	Deaths	Male	2013	5.34853	6.869344	4.202879	
Oman	Deaths	Male	2014	5.128156	6.50966	3.993793	
Oman	Deaths	Male	2015	4.958045	6.288276	3.764707	
Oman	Deaths	Male	2016	4.845486	6.166011	3.609299	
Oman	Deaths	Male	2017	4.786976	6.27271	3.453104	
Oman	Deaths	Male	2018	4.625061	6.215927	3.259223	
Oman	Deaths	Male	2019	4.449296	6.080377	3.108563	
Oman	Deaths	Male	2020	4.692901	6.522312	3.376503	
Oman	Deaths	Male	2021	4.948261	7.0279	3.554892	
Oman	Deaths	Male	2022	4.474619	6.693219	3.104067	
Oman	Deaths	Male	2023	4.671591	6.908206	3.2565	
Oman	Deaths	Female	2010	1.822026	2.57862	1.283564	
Oman	Deaths	Female	2011	1.871483	2.655009	1.292255	
Oman	Deaths	Female	2012	1.898224	2.592114	1.305602	
Oman	Deaths	Female	2013	1.916992	2.614425	1.315194	
Oman	Deaths	Female	2014	1.928987	2.678108	1.27664	
Oman	Deaths	Female	2015	1.919233	2.632325	1.26207	
Oman	Deaths	Female	2016	1.899728	2.588514	1.296639	

Oman	Deaths	Female	2017	1.924275	2.658554	1.329951	
Oman	Deaths	Female	2018	1.923522	2.611405	1.372687	
Oman	Deaths	Female	2019	1.914725	2.607474	1.36278	
Oman	Deaths	Female	2020	1.915264	2.578724	1.384527	
Oman	Deaths	Female	2021	2.08374	2.858637	1.453969	
Oman	Deaths	Female	2022	2.011913	2.777217	1.413053	
Oman	Deaths	Female	2023	2.079051	2.785411	1.432994	
Oman	DALYs	Male	2010	153.4044	196.4014	117.6363	
Oman	DALYs	Male	2011	157.4481	200.0257	122.0026	
Oman	DALYs	Male	2012	160.3105	202.7221	124.4062	
Oman	DALYs	Male	2013	154.4356	198.2306	120.3534	
Oman	DALYs	Male	2014	148.4232	188.7776	113.3568	
Oman	DALYs	Male	2015	144.2108	183.1504	108.4687	
Oman	DALYs	Male	2016	141.7148	180.9613	101.889	
Oman	DALYs	Male	2017	140.3847	184.074	99.52743	
Oman	DALYs	Male	2018	136.1925	185.0457	95.10258	
Oman	DALYs	Male	2019	132.1726	182.4767	90.44118	
Oman	DALYs	Male	2020	137.1527	191.787	97.25228	
Oman	DALYs	Male	2021	145.1946	205.7791	100.3746	
Oman	DALYs	Male	2022	133.9068	195.4115	89.10869	
Oman	DALYs	Male	2023	141.8283	208.8002	97.95018	
Oman	DALYs	Female	2010	52.43418	73.8583	36.96065	
Oman	DALYs	Female	2011	53.77717	75.94739	36.76976	
Oman	DALYs	Female	2012	54.44347	73.95224	37.39307	
Oman	DALYs	Female	2013	54.78265	74.83352	37.68416	
Oman	DALYs	Female	2014	54.97154	76.58259	36.20121	
Oman	DALYs	Female	2015	54.55572	75.78096	35.52848	
Oman	DALYs	Female	2016	53.91187	73.21471	36.52908	
Oman	DALYs	Female	2017	54.32449	73.36523	37.13564	
Oman	DALYs	Female	2018	54.11787	73.95162	38.04607	
Oman	DALYs	Female	2019	53.80639	73.44974	38.21568	
Oman	DALYs	Female	2020	52.84168	71.17141	38.1005	
Oman	DALYs	Female	2021	57.54117	78.65303	40.08559	
Oman	DALYs	Female	2022	55.21032	75.8702	38.86971	
Oman	DALYs	Female	2023	57.96386	78.63665	39.94916	
Pakistan	Incidence	Male	2010	3.922995	5.962227	2.775641	

Pakistan	Incidence	Male	2011	3.967055	5.684936	2.826573	
Pakistan	Incidence	Male	2012	4.042599	5.953346	2.852307	
Pakistan	Incidence	Male	2013	4.049868	6.090844	2.782597	
Pakistan	Incidence	Male	2014	4.077927	5.892891	2.853766	
Pakistan	Incidence	Male	2015	4.02822	5.985379	2.779032	
Pakistan	Incidence	Male	2016	4.046247	6.131496	2.756511	
Pakistan	Incidence	Male	2017	4.009461	6.272697	2.703913	
Pakistan	Incidence	Male	2018	4.033902	6.476482	2.712366	
Pakistan	Incidence	Male	2019	4.006033	6.848757	2.606786	
Pakistan	Incidence	Male	2020	4.234952	7.542978	2.801119	
Pakistan	Incidence	Male	2021	4.61695	8.123226	3.07817	
Pakistan	Incidence	Male	2022	4.503198	7.610114	2.89557	
Pakistan	Incidence	Male	2023	4.793386	7.965352	3.13712	
Pakistan	Incidence	Female	2010	2.909946	4.241403	1.94322	
Pakistan	Incidence	Female	2011	2.959498	4.315947	1.99345	
Pakistan	Incidence	Female	2012	3.027157	4.278358	1.990892	
Pakistan	Incidence	Female	2013	3.132348	4.410538	2.016805	
Pakistan	Incidence	Female	2014	3.201532	4.448179	2.081083	
Pakistan	Incidence	Female	2015	3.245813	4.492361	2.080361	
Pakistan	Incidence	Female	2016	3.317784	4.568499	2.103362	
Pakistan	Incidence	Female	2017	3.37112	4.594058	2.197815	
Pakistan	Incidence	Female	2018	3.428982	4.68334	2.349015	
Pakistan	Incidence	Female	2019	3.533313	4.850101	2.431177	
Pakistan	Incidence	Female	2020	3.799162	5.446889	2.625542	
Pakistan	Incidence	Female	2021	4.299256	6.099091	3.005896	
Pakistan	Incidence	Female	2022	4.208211	5.907613	2.752653	
Pakistan	Incidence	Female	2023	4.320233	6.241447	2.926516	
Pakistan	Deaths	Male	2010	3.920507	5.959191	2.739044	
Pakistan	Deaths	Male	2011	3.958114	5.731358	2.825106	
Pakistan	Deaths	Male	2012	4.026771	5.877356	2.855676	
Pakistan	Deaths	Male	2013	4.029057	6.131252	2.750733	
Pakistan	Deaths	Male	2014	4.051859	5.86732	2.816243	
Pakistan	Deaths	Male	2015	3.997532	5.885604	2.762812	
Pakistan	Deaths	Male	2016	4.01182	6.04717	2.739326	
Pakistan	Deaths	Male	2017	3.972116	6.152906	2.679575	
Pakistan	Deaths	Male	2018	3.993149	6.362209	2.705242	

Pakistan	Deaths	Male	2019	3.963353	6.699613	2.554419	
Pakistan	Deaths	Male	2020	4.185341	7.36676	2.770511	
Pakistan	Deaths	Male	2021	4.548532	7.988196	3.031991	
Pakistan	Deaths	Male	2022	4.458395	7.51044	2.875469	
Pakistan	Deaths	Male	2023	4.742259	7.809759	3.116814	
Pakistan	Deaths	Female	2010	2.821533	4.111415	1.88798	
Pakistan	Deaths	Female	2011	2.865808	4.192373	1.935971	
Pakistan	Deaths	Female	2012	2.927868	4.147034	1.931466	
Pakistan	Deaths	Female	2013	3.02709	4.265104	1.937437	
Pakistan	Deaths	Female	2014	3.091314	4.304718	2.0122	
Pakistan	Deaths	Female	2015	3.131414	4.312739	2.000036	
Pakistan	Deaths	Female	2016	3.197869	4.393515	2.020143	
Pakistan	Deaths	Female	2017	3.247592	4.453587	2.118214	
Pakistan	Deaths	Female	2018	3.300953	4.525192	2.270104	
Pakistan	Deaths	Female	2019	3.401081	4.651031	2.344283	
Pakistan	Deaths	Female	2020	3.640165	5.222442	2.508173	
Pakistan	Deaths	Female	2021	4.098157	5.793854	2.837712	
Pakistan	Deaths	Female	2022	4.041762	5.679372	2.641652	
Pakistan	Deaths	Female	2023	4.144804	6.015936	2.838143	
Pakistan	DALYs	Male	2010	108.5059	165.4988	75.71235	
Pakistan	DALYs	Male	2011	109.9887	160.1589	77.21312	
Pakistan	DALYs	Male	2012	112.4062	164.2177	79.69232	
Pakistan	DALYs	Male	2013	112.795	166.433	77.90433	
Pakistan	DALYs	Male	2014	113.781	163.8313	78.92035	
Pakistan	DALYs	Male	2015	112.5782	169.7257	77.47246	
Pakistan	DALYs	Male	2016	113.1759	174.3136	77.32506	
Pakistan	DALYs	Male	2017	112.2782	178.4349	75.69792	
Pakistan	DALYs	Male	2018	113.0781	183.2969	75.69761	
Pakistan	DALYs	Male	2019	112.3245	195.8986	71.94572	
Pakistan	DALYs	Male	2020	118.4693	213.5539	77.2742	
Pakistan	DALYs	Male	2021	129.7	225.138	85.14153	
Pakistan	DALYs	Male	2022	125.4424	211.5989	81.72161	
Pakistan	DALYs	Male	2023	133.6769	221.2662	87.03482	
Pakistan	DALYs	Female	2010	86.02907	125.3731	57.2209	
Pakistan	DALYs	Female	2011	87.69418	127.8571	59.00989	
Pakistan	DALYs	Female	2012	89.88364	125.9803	58.61948	

Pakistan	DALYs	Female	2013	93.08912	130.1491	61.26724	
Pakistan	DALYs	Female	2014	95.25971	132.3863	61.99344	
Pakistan	DALYs	Female	2015	96.66561	135.2351	61.76643	
Pakistan	DALYs	Female	2016	98.8867	136.8298	62.71735	
Pakistan	DALYs	Female	2017	100.4932	135.5013	65.557	
Pakistan	DALYs	Female	2018	102.2783	139.43	69.05397	
Pakistan	DALYs	Female	2019	105.2531	144.9191	71.78218	
Pakistan	DALYs	Female	2020	113.6867	162.4105	79.38781	
Pakistan	DALYs	Female	2021	129.5619	181.1197	91.12555	
Pakistan	DALYs	Female	2022	124.7708	173.7809	82.8808	
Pakistan	DALYs	Female	2023	128.2653	183.0684	88.41803	
Gaza Strip and	Incidence	Male	2010	11.16564	13.3982	9.115456	
Gaza Strip and	Incidence	Male	2011	11.29624	13.51683	9.306248	
Gaza Strip and	Incidence	Male	2012	11.32994	13.49657	9.298502	
Gaza Strip and	Incidence	Male	2013	11.53452	13.66704	9.355178	
Gaza Strip and	Incidence	Male	2014	11.65883	13.84051	9.574648	
Gaza Strip and	Incidence	Male	2015	12.40843	14.84209	10.24388	
Gaza Strip and	Incidence	Male	2016	12.63056	15.31147	10.52049	
Gaza Strip and	Incidence	Male	2017	12.99539	15.99367	11.06396	
Gaza Strip and	Incidence	Male	2018	13.26015	16.65945	11.23553	
Gaza Strip and	Incidence	Male	2019	13.44784	17.18076	11.28738	
Gaza Strip and	Incidence	Male	2020	12.97874	16.81966	10.48784	
Gaza Strip and	Incidence	Male	2021	13.55162	17.76889	11.00641	
Gaza Strip and	Incidence	Male	2022	14.49513	18.99953	11.45594	
Gaza Strip and	Incidence	Male	2023	15.55941	20.9612	12.13529	
Gaza Strip and	Incidence	Female	2010	2.937276	4.008264	2.184282	
Gaza Strip and	Incidence	Female	2011	3.011565	4.012542	2.228833	
Gaza Strip and	Incidence	Female	2012	3.065153	4.01168	2.282089	
Gaza Strip and	Incidence	Female	2013	3.069523	3.968714	2.314674	
Gaza Strip and	Incidence	Female	2014	3.116624	4.056285	2.342036	
Gaza Strip and	Incidence	Female	2015	3.182834	4.119357	2.358776	
Gaza Strip and	Incidence	Female	2016	3.252046	4.080974	2.479456	
Gaza Strip and	Incidence	Female	2017	3.356076	4.144879	2.567885	
Gaza Strip and	Incidence	Female	2018	3.433722	4.275561	2.677481	
Gaza Strip and	Incidence	Female	2019	3.492465	4.339452	2.745037	
Gaza Strip and	Incidence	Female	2020	3.426308	4.295465	2.644304	

Gaza Strip and	Incidence	Female	2021	3.61246	4.597919	2.719411	
Gaza Strip and	Incidence	Female	2022	3.800609	4.774149	2.858147	
Gaza Strip and	Incidence	Female	2023	4.013192	5.145302	3.007341	
Gaza Strip and	Deaths	Male	2010	10.88195	13.02725	8.898678	
Gaza Strip and	Deaths	Male	2011	10.98526	13.09365	9.017991	
Gaza Strip and	Deaths	Male	2012	10.99592	13.05654	8.946328	
Gaza Strip and	Deaths	Male	2013	11.18467	13.26225	9.058904	
Gaza Strip and	Deaths	Male	2014	11.28672	13.33429	9.207488	
Gaza Strip and	Deaths	Male	2015	11.97656	14.27455	9.839298	
Gaza Strip and	Deaths	Male	2016	12.14885	14.67403	10.06633	
Gaza Strip and	Deaths	Male	2017	12.45533	15.2695	10.52423	
Gaza Strip and	Deaths	Male	2018	12.68651	15.85657	10.80995	
Gaza Strip and	Deaths	Male	2019	12.83163	16.32234	10.77848	
Gaza Strip and	Deaths	Male	2020	12.36815	15.97738	10.0509	
Gaza Strip and	Deaths	Male	2021	12.88604	16.86633	10.47673	
Gaza Strip and	Deaths	Male	2022	13.70987	17.85178	10.80629	
Gaza Strip and	Deaths	Male	2023	14.71304	19.67692	11.55341	
Gaza Strip and	Deaths	Female	2010	2.855848	3.903316	2.115228	
Gaza Strip and	Deaths	Female	2011	2.923209	3.869335	2.1445	
Gaza Strip and	Deaths	Female	2012	2.969505	3.881024	2.212787	
Gaza Strip and	Deaths	Female	2013	2.966935	3.807666	2.247591	
Gaza Strip and	Deaths	Female	2014	3.005223	3.866642	2.242576	
Gaza Strip and	Deaths	Female	2015	3.061414	3.917267	2.265007	
Gaza Strip and	Deaths	Female	2016	3.116873	3.88939	2.366047	
Gaza Strip and	Deaths	Female	2017	3.204426	3.983956	2.471841	
Gaza Strip and	Deaths	Female	2018	3.269337	4.100206	2.558014	
Gaza Strip and	Deaths	Female	2019	3.314231	4.141827	2.605117	
Gaza Strip and	Deaths	Female	2020	3.250239	4.099817	2.529653	
Gaza Strip and	Deaths	Female	2021	3.416269	4.343262	2.600467	
Gaza Strip and	Deaths	Female	2022	3.571012	4.543247	2.705601	
Gaza Strip and	Deaths	Female	2023	3.76477	4.773105	2.814374	
Gaza Strip and	DALYs	Male	2010	304.2885	366.6576	251.0653	
Gaza Strip and	DALYs	Male	2011	306.5982	368.8731	256.3147	
Gaza Strip and	DALYs	Male	2012	305.5573	365.9655	255.126	
Gaza Strip and	DALYs	Male	2013	308.8846	369.5831	253.6971	
Gaza Strip and	DALYs	Male	2014	310.6633	372.5047	257.1918	

Gaza Strip and	DALYs	Male	2015	329.918	398.6888	273.6708	
Gaza Strip and	DALYs	Male	2016	334.38	409.1648	280.1653	
Gaza Strip and	DALYs	Male	2017	342.2106	424.9549	289.2842	
Gaza Strip and	DALYs	Male	2018	347.8572	439.5864	296.2607	
Gaza Strip and	DALYs	Male	2019	352.4135	453.0083	296.332	
Gaza Strip and	DALYs	Male	2020	338.5085	440.9703	275.6751	
Gaza Strip and	DALYs	Male	2021	349.9003	462.9383	281.512	
Gaza Strip and	DALYs	Male	2022	374.0966	491.4143	292.8708	
Gaza Strip and	DALYs	Male	2023	399.5056	536.9938	313.2924	
Gaza Strip and	DALYs	Female	2010	78.6527	107.7118	57.84731	
Gaza Strip and	DALYs	Female	2011	80.40583	107.6206	59.17371	
Gaza Strip and	DALYs	Female	2012	81.36121	106.7636	59.73216	
Gaza Strip and	DALYs	Female	2013	81.12495	104.9964	60.59667	
Gaza Strip and	DALYs	Female	2014	82.2251	106.3651	60.48497	
Gaza Strip and	DALYs	Female	2015	83.61711	107.3214	61.66162	
Gaza Strip and	DALYs	Female	2016	84.87967	105.7468	64.04883	
Gaza Strip and	DALYs	Female	2017	87.12233	107.8558	66.92943	
Gaza Strip and	DALYs	Female	2018	88.84837	110.6703	68.89162	
Gaza Strip and	DALYs	Female	2019	90.19911	111.5075	70.55215	
Gaza Strip and	DALYs	Female	2020	87.73811	110.9543	68.16483	
Gaza Strip and	DALYs	Female	2021	91.5516	116.5945	70.01178	
Gaza Strip and	DALYs	Female	2022	96.64017	122.1914	73.69629	
Gaza Strip and	DALYs	Female	2023	101.8984	129.4758	76.67123	
Panama	Incidence	Male	2010	13.01879	14.80395	11.49643	
Panama	Incidence	Male	2011	13.20479	14.87299	11.79299	
Panama	Incidence	Male	2012	13.02712	14.52724	11.50561	
Panama	Incidence	Male	2013	13.2174	14.79858	11.71928	
Panama	Incidence	Male	2014	13.00147	14.66405	11.51253	
Panama	Incidence	Male	2015	13.00246	14.59984	11.75167	
Panama	Incidence	Male	2016	12.5761	14.18795	11.35062	
Panama	Incidence	Male	2017	12.51629	14.15301	11.11054	
Panama	Incidence	Male	2018	12.33804	13.91991	10.85873	
Panama	Incidence	Male	2019	12.60374	14.37394	11.07318	
Panama	Incidence	Male	2020	11.90096	13.58355	10.52003	
Panama	Incidence	Male	2021	10.48089	12.00127	9.395112	
Panama	Incidence	Male	2022	10.89277	12.52447	9.600452	

Panama	Incidence	Male	2023	11.02939	12.83669	9.461152	
Panama	Incidence	Female	2010	6.383946	7.375726	5.450425	
Panama	Incidence	Female	2011	6.602325	7.509046	5.643471	
Panama	Incidence	Female	2012	6.689389	7.658319	5.712427	
Panama	Incidence	Female	2013	7.322576	8.388986	6.22126	
Panama	Incidence	Female	2014	7.472825	8.582835	6.340251	
Panama	Incidence	Female	2015	7.656897	8.819237	6.399591	
Panama	Incidence	Female	2016	7.379044	8.54137	6.123362	
Panama	Incidence	Female	2017	7.246353	8.475508	6.037679	
Panama	Incidence	Female	2018	7.325966	8.591066	6.120336	
Panama	Incidence	Female	2019	7.589784	9.03729	6.351441	
Panama	Incidence	Female	2020	6.947466	8.252359	5.816768	
Panama	Incidence	Female	2021	6.430646	7.588919	5.33138	
Panama	Incidence	Female	2022	6.787014	8.033867	5.576544	
Panama	Incidence	Female	2023	6.963899	8.381367	5.677267	
Panama	Deaths	Male	2010	12.91078	14.52334	11.44158	
Panama	Deaths	Male	2011	13.06054	14.52171	11.74901	
Panama	Deaths	Male	2012	12.82482	14.33114	11.44269	
Panama	Deaths	Male	2013	12.99933	14.4346	11.61144	
Panama	Deaths	Male	2014	12.81806	14.34139	11.47054	
Panama	Deaths	Male	2015	12.80514	14.27184	11.56513	
Panama	Deaths	Male	2016	12.3471	13.86721	11.12642	
Panama	Deaths	Male	2017	12.25314	13.77209	10.96253	
Panama	Deaths	Male	2018	12.14465	13.64627	10.78982	
Panama	Deaths	Male	2019	12.33865	13.93768	10.87737	
Panama	Deaths	Male	2020	11.63887	13.18203	10.26488	
Panama	Deaths	Male	2021	10.1966	11.5094	9.100248	
Panama	Deaths	Male	2022	10.59054	12.1111	9.293417	
Panama	Deaths	Male	2023	10.6892	12.3527	9.127394	
Panama	Deaths	Female	2010	5.802418	6.617886	4.98985	
Panama	Deaths	Female	2011	5.961522	6.745891	5.064906	
Panama	Deaths	Female	2012	6.015485	6.791182	5.137208	
Panama	Deaths	Female	2013	6.555335	7.356396	5.570977	
Panama	Deaths	Female	2014	6.672908	7.459952	5.651198	
Panama	Deaths	Female	2015	6.78898	7.715	5.718632	
Panama	Deaths	Female	2016	6.535344	7.451221	5.542208	

Panama	Deaths	Female	2017	6.371393	7.286053	5.356644	
Panama	Deaths	Female	2018	6.427428	7.39244	5.440567	
Panama	Deaths	Female	2019	6.580947	7.622103	5.576544	
Panama	Deaths	Female	2020	5.993085	7.007468	5.110497	
Panama	Deaths	Female	2021	5.521384	6.42906	4.675594	
Panama	Deaths	Female	2022	5.828194	6.778201	4.851093	
Panama	Deaths	Female	2023	5.940686	7.040125	4.920084	
Panama	DALYs	Male	2010	294.6703	333.1365	261.7706	
Panama	DALYs	Male	2011	298.6414	331.6852	267.975	
Panama	DALYs	Male	2012	296.515	331.3433	262.5486	
Panama	DALYs	Male	2013	301.2999	335.9935	267.7596	
Panama	DALYs	Male	2014	293.0459	328.6414	261.937	
Panama	DALYs	Male	2015	290.2076	327.1202	261.3971	
Panama	DALYs	Male	2016	280.7512	316.848	253.2738	
Panama	DALYs	Male	2017	279.4865	314.5913	248.909	
Panama	DALYs	Male	2018	272.056	305.887	241.9608	
Panama	DALYs	Male	2019	275.4868	310.5268	243.2483	
Panama	DALYs	Male	2020	258.1375	292.4965	226.3565	
Panama	DALYs	Male	2021	227.0678	255.8024	202.0547	
Panama	DALYs	Male	2022	233.9628	267.6606	207.0181	
Panama	DALYs	Male	2023	234.2004	270.6297	203.5843	
Panama	DALYs	Female	2010	135.8093	155.2707	118.0726	
Panama	DALYs	Female	2011	140.7193	159.2374	122.5613	
Panama	DALYs	Female	2012	142.1095	159.6853	123.5239	
Panama	DALYs	Female	2013	153.4159	171.9214	132.1104	
Panama	DALYs	Female	2014	155.284	174.4237	132.1389	
Panama	DALYs	Female	2015	158.6269	180.0891	134.0981	
Panama	DALYs	Female	2016	151.1762	172.945	129.1386	
Panama	DALYs	Female	2017	148.1795	170.214	127.4714	
Panama	DALYs	Female	2018	149.1074	172.1004	127.5315	
Panama	DALYs	Female	2019	152.5263	178.3783	130.7067	
Panama	DALYs	Female	2020	138.7316	162.9696	118.3942	
Panama	DALYs	Female	2021	126.0807	147.1069	108.063	
Panama	DALYs	Female	2022	130.9869	152.1334	110.4267	
Panama	DALYs	Female	2023	132.7813	156.1419	110.4467	
Papua New Gui	Incidence	Male	2010	11.82763	17.2698	7.585998	

Papua New Gui	Incidence	Male	2011	11.90095	17.47199	7.779028	
Papua New Gui	Incidence	Male	2012	11.9013	17.9815	7.794167	
Papua New Gui	Incidence	Male	2013	11.62159	17.41055	7.527008	
Papua New Gui	Incidence	Male	2014	11.77717	17.52329	7.606531	
Papua New Gui	Incidence	Male	2015	12.79082	18.82353	8.327809	
Papua New Gui	Incidence	Male	2016	12.60778	18.45913	8.270898	
Papua New Gui	Incidence	Male	2017	12.76579	18.77533	8.150535	
Papua New Gui	Incidence	Male	2018	12.86534	19.04426	8.033385	
Papua New Gui	Incidence	Male	2019	13.33287	19.679	8.616365	
Papua New Gui	Incidence	Male	2020	13.43332	19.87238	8.330411	
Papua New Gui	Incidence	Male	2021	13.83643	20.52678	8.558818	
Papua New Gui	Incidence	Male	2022	15.05482	23.36401	9.22204	
Papua New Gui	Incidence	Male	2023	15.63651	24.49879	9.070177	
Papua New Gui	Incidence	Female	2010	5.816432	8.842569	3.656922	
Papua New Gui	Incidence	Female	2011	6.005673	9.391044	3.78309	
Papua New Gui	Incidence	Female	2012	6.085901	9.846155	3.885888	
Papua New Gui	Incidence	Female	2013	5.940239	9.252583	3.935581	
Papua New Gui	Incidence	Female	2014	6.086416	9.460076	3.971888	
Papua New Gui	Incidence	Female	2015	6.460126	9.828089	4.045588	
Papua New Gui	Incidence	Female	2016	6.382434	9.587586	3.948282	
Papua New Gui	Incidence	Female	2017	6.538559	9.631909	4.087365	
Papua New Gui	Incidence	Female	2018	6.607394	9.817428	4.177135	
Papua New Gui	Incidence	Female	2019	7.007154	10.05811	4.520541	
Papua New Gui	Incidence	Female	2020	6.997487	10.59564	4.523546	
Papua New Gui	Incidence	Female	2021	7.018482	10.38394	4.54743	
Papua New Gui	Incidence	Female	2022	8.252861	12.04213	5.266135	
Papua New Gui	Incidence	Female	2023	8.653287	13.30139	5.510635	
Papua New Gui	Deaths	Male	2010	11.6885	17.04208	7.549884	
Papua New Gui	Deaths	Male	2011	11.74415	17.06467	7.707809	
Papua New Gui	Deaths	Male	2012	11.73854	17.67642	7.683143	
Papua New Gui	Deaths	Male	2013	11.46661	17.13658	7.434694	
Papua New Gui	Deaths	Male	2014	11.6174	17.24216	7.509637	
Papua New Gui	Deaths	Male	2015	12.61352	18.57232	8.228935	
Papua New Gui	Deaths	Male	2016	12.43811	18.27803	8.171486	
Papua New Gui	Deaths	Male	2017	12.59901	18.44334	8.001546	
Papua New Gui	Deaths	Male	2018	12.70273	18.83136	7.904787	

Papua New Gui	Deaths	Male	2019	13.16231	19.42644	8.503619	
Papua New Gui	Deaths	Male	2020	13.26716	19.66072	8.21613	
Papua New Gui	Deaths	Male	2021	13.6495	20.15621	8.475115	
Papua New Gui	Deaths	Male	2022	14.86327	23.0709	9.142529	
Papua New Gui	Deaths	Male	2023	15.445	24.06297	8.958024	
Papua New Gui	Deaths	Female	2010	5.717647	8.681114	3.590007	
Papua New Gui	Deaths	Female	2011	5.88806	9.186232	3.681348	
Papua New Gui	Deaths	Female	2012	5.958861	9.629529	3.79836	
Papua New Gui	Deaths	Female	2013	5.815938	9.057053	3.861062	
Papua New Gui	Deaths	Female	2014	5.948898	9.249527	3.881435	
Papua New Gui	Deaths	Female	2015	6.300512	9.601801	3.951489	
Papua New Gui	Deaths	Female	2016	6.223802	9.300607	3.856872	
Papua New Gui	Deaths	Female	2017	6.373539	9.332509	3.979415	
Papua New Gui	Deaths	Female	2018	6.439024	9.531411	4.05808	
Papua New Gui	Deaths	Female	2019	6.816886	9.751383	4.387482	
Papua New Gui	Deaths	Female	2020	6.803742	10.30598	4.401438	
Papua New Gui	Deaths	Female	2021	6.808778	10.08564	4.416899	
Papua New Gui	Deaths	Female	2022	7.998567	11.73266	5.112516	
Papua New Gui	Deaths	Female	2023	8.390124	12.84242	5.336777	
Papua New Gui	DALYs	Male	2010	339.4466	502.1496	213.6783	
Papua New Gui	DALYs	Male	2011	342.9367	510.8382	219.7763	
Papua New Gui	DALYs	Male	2012	343.2813	518.1338	222.8365	
Papua New Gui	DALYs	Male	2013	334.6161	504.7489	215.6124	
Papua New Gui	DALYs	Male	2014	338.8778	503.4493	219.299	
Papua New Gui	DALYs	Male	2015	367.9029	538.3128	238.1519	
Papua New Gui	DALYs	Male	2016	361.6442	532.9347	233.924	
Papua New Gui	DALYs	Male	2017	365.3043	546.7048	230.589	
Papua New Gui	DALYs	Male	2018	367.526	553.2526	228.838	
Papua New Gui	DALYs	Male	2019	380.3363	572.9784	243.419	
Papua New Gui	DALYs	Male	2020	381.6644	568.163	237.6398	
Papua New Gui	DALYs	Male	2021	392.3262	593.26	238.0228	
Papua New Gui	DALYs	Male	2022	423.5736	655.3009	254.8729	
Papua New Gui	DALYs	Male	2023	438.183	682.07	252.4489	
Papua New Gui	DALYs	Female	2010	160.7622	246.091	100.9069	
Papua New Gui	DALYs	Female	2011	166.9705	263.6207	104.8536	
Papua New Gui	DALYs	Female	2012	169.5905	272.4869	108.691	

Papua New Gui	DALYs	Female	2013	165.3559	257.2449	108.7338	
Papua New Gui	DALYs	Female	2014	169.9161	263.4069	111.1728	
Papua New Gui	DALYs	Female	2015	181.0094	275.4373	113.6626	
Papua New Gui	DALYs	Female	2016	178.6051	269.1828	110.4979	
Papua New Gui	DALYs	Female	2017	182.9055	268.7646	115.9784	
Papua New Gui	DALYs	Female	2018	184.7701	274.7476	116.2331	
Papua New Gui	DALYs	Female	2019	196.3869	283.6932	127.3071	
Papua New Gui	DALYs	Female	2020	195.4529	294.9654	124.7166	
Papua New Gui	DALYs	Female	2021	195.7935	293.1749	126.53	
Papua New Gui	DALYs	Female	2022	229.3797	335.5956	146.2578	
Papua New Gui	DALYs	Female	2023	239.7508	371.3456	151.7635	
Paraguay	Incidence	Male	2010	17.11059	19.79479	11.16013	
Paraguay	Incidence	Male	2011	17.48831	20.26837	11.27746	
Paraguay	Incidence	Male	2012	17.79326	20.55442	11.47118	
Paraguay	Incidence	Male	2013	18.05498	20.93837	11.69361	
Paraguay	Incidence	Male	2014	18.54971	21.45403	12.09699	
Paraguay	Incidence	Male	2015	19.0324	21.9439	12.44782	
Paraguay	Incidence	Male	2016	19.59368	22.4467	12.82867	
Paraguay	Incidence	Male	2017	20.19595	23.05369	13.32575	
Paraguay	Incidence	Male	2018	20.63416	23.34249	13.75934	
Paraguay	Incidence	Male	2019	21.15766	23.96695	14.36536	
Paraguay	Incidence	Male	2020	21.48168	24.14234	14.56371	
Paraguay	Incidence	Male	2021	22.03334	24.68972	14.90582	
Paraguay	Incidence	Male	2022	20.09905	23.40706	13.48761	
Paraguay	Incidence	Male	2023	20.99511	24.96664	13.56257	
Paraguay	Incidence	Female	2010	5.598074	6.596305	4.623474	
Paraguay	Incidence	Female	2011	5.808971	6.828612	4.665393	
Paraguay	Incidence	Female	2012	6.029262	7.12523	4.858386	
Paraguay	Incidence	Female	2013	6.156878	7.187094	4.931735	
Paraguay	Incidence	Female	2014	6.385464	7.435074	5.169255	
Paraguay	Incidence	Female	2015	6.602005	7.641692	5.392708	
Paraguay	Incidence	Female	2016	6.797135	7.906982	5.555483	
Paraguay	Incidence	Female	2017	6.97847	8.249512	5.667781	
Paraguay	Incidence	Female	2018	7.170541	8.426946	5.885047	
Paraguay	Incidence	Female	2019	7.419127	8.664169	6.083081	
Paraguay	Incidence	Female	2020	7.516262	8.862411	6.085001	

Paraguay	Incidence	Female	2021	7.970844	9.494539	6.114208	
Paraguay	Incidence	Female	2022	7.419327	9.051944	5.771602	
Paraguay	Incidence	Female	2023	7.842714	9.85033	6.163836	
Paraguay	Deaths	Male	2010	17.36583	20.00211	11.27024	
Paraguay	Deaths	Male	2011	17.75738	20.58923	11.37437	
Paraguay	Deaths	Male	2012	18.07972	20.8277	11.57118	
Paraguay	Deaths	Male	2013	18.3486	21.38686	11.81434	
Paraguay	Deaths	Male	2014	18.86148	21.82708	12.20904	
Paraguay	Deaths	Male	2015	19.35386	22.37657	12.51564	
Paraguay	Deaths	Male	2016	19.91872	22.90934	12.94464	
Paraguay	Deaths	Male	2017	20.52106	23.46829	13.52288	
Paraguay	Deaths	Male	2018	20.94953	23.79107	14.03302	
Paraguay	Deaths	Male	2019	21.45148	24.42679	14.36005	
Paraguay	Deaths	Male	2020	21.76604	24.68569	14.7767	
Paraguay	Deaths	Male	2021	22.28546	25.10853	15.07784	
Paraguay	Deaths	Male	2022	20.41834	23.97452	13.66541	
Paraguay	Deaths	Male	2023	21.24228	25.51156	13.75118	
Paraguay	Deaths	Female	2010	5.684974	6.7272	4.712855	
Paraguay	Deaths	Female	2011	5.892559	6.910111	4.718344	
Paraguay	Deaths	Female	2012	6.114453	7.220868	4.895763	
Paraguay	Deaths	Female	2013	6.243673	7.290974	5.041865	
Paraguay	Deaths	Female	2014	6.472859	7.435605	5.231717	
Paraguay	Deaths	Female	2015	6.691792	7.744771	5.513231	
Paraguay	Deaths	Female	2016	6.88911	8.01089	5.656145	
Paraguay	Deaths	Female	2017	7.064181	8.292847	5.759976	
Paraguay	Deaths	Female	2018	7.244399	8.442274	5.970778	
Paraguay	Deaths	Female	2019	7.473043	8.701201	6.130972	
Paraguay	Deaths	Female	2020	7.555701	8.847877	6.189335	
Paraguay	Deaths	Female	2021	7.98486	9.539021	6.196206	
Paraguay	Deaths	Female	2022	7.459385	9.151482	5.877194	
Paraguay	Deaths	Female	2023	7.86385	9.88819	6.179514	
Paraguay	DALYs	Male	2010	431.4243	497.6079	282.6556	
Paraguay	DALYs	Male	2011	439.3652	508.5992	287.0163	
Paraguay	DALYs	Male	2012	445.5465	514.0515	291.1361	
Paraguay	DALYs	Male	2013	451.0608	524.1012	296.2975	
Paraguay	DALYs	Male	2014	461.8064	535.4202	305.2076	

Paraguay	DALYs	Male	2015	472.089	545.9429	312.1875	
Paraguay	DALYs	Male	2016	484.7821	552.4264	321.1267	
Paraguay	DALYs	Male	2017	498.8245	562.768	333.0944	
Paraguay	DALYs	Male	2018	508.9845	571.5831	344.5811	
Paraguay	DALYs	Male	2019	521.7598	587.5597	357.8528	
Paraguay	DALYs	Male	2020	529.4959	595.2733	363.7941	
Paraguay	DALYs	Male	2021	544.0374	602.8827	372.9221	
Paraguay	DALYs	Male	2022	487.4007	557.1328	329.4968	
Paraguay	DALYs	Male	2023	510.1146	598.1314	336.801	
Paraguay	DALYs	Female	2010	140.5334	163.7719	115.3942	
Paraguay	DALYs	Female	2011	145.7483	170.3182	119.4065	
Paraguay	DALYs	Female	2012	151.2151	177.9146	124.2252	
Paraguay	DALYs	Female	2013	154.1407	178.8851	125.3123	
Paraguay	DALYs	Female	2014	159.7377	184.1845	130.2503	
Paraguay	DALYs	Female	2015	164.6327	190.8958	133.4376	
Paraguay	DALYs	Female	2016	168.9425	197.2294	136.1277	
Paraguay	DALYs	Female	2017	173.4508	202.6764	139.5373	
Paraguay	DALYs	Female	2018	178.5154	207.667	144.8202	
Paraguay	DALYs	Female	2019	185.1698	217.2085	149.2648	
Paraguay	DALYs	Female	2020	187.9923	221.4802	148.2826	
Paraguay	DALYs	Female	2021	199.3634	238.0498	152.1656	
Paraguay	DALYs	Female	2022	182.4384	223.6842	140.0123	
Paraguay	DALYs	Female	2023	192.3212	242.7983	149.1574	
Peru	Incidence	Male	2010	8.939471	10.93623	7.456607	
Peru	Incidence	Male	2011	8.726	10.64626	7.374669	
Peru	Incidence	Male	2012	8.655635	10.36035	7.413129	
Peru	Incidence	Male	2013	8.429642	10.14935	7.168863	
Peru	Incidence	Male	2014	8.247626	10.13071	7.061974	
Peru	Incidence	Male	2015	7.991686	10.01111	6.85882	
Peru	Incidence	Male	2016	8.208902	10.40876	7.046611	
Peru	Incidence	Male	2017	8.998742	11.48648	7.671096	
Peru	Incidence	Male	2018	9.481974	12.34582	8.040378	
Peru	Incidence	Male	2019	9.880961	13.04731	8.328315	
Peru	Incidence	Male	2020	10.79737	13.84852	9.061345	
Peru	Incidence	Male	2021	12.57542	16.0227	10.43713	
Peru	Incidence	Male	2022	11.60122	15.0036	9.523145	

Peru	Incidence	Male	2023	11.37244	14.7374	9.179065	
Peru	Incidence	Female	2010	8.950292	10.35235	7.702129	
Peru	Incidence	Female	2011	9.001188	10.57821	7.80263	
Peru	Incidence	Female	2012	9.117102	10.64808	7.853439	
Peru	Incidence	Female	2013	9.156596	10.78633	7.845386	
Peru	Incidence	Female	2014	9.128744	10.91583	7.678397	
Peru	Incidence	Female	2015	9.144513	11.41108	7.703193	
Peru	Incidence	Female	2016	9.659688	11.94896	8.106998	
Peru	Incidence	Female	2017	10.64785	13.16783	8.899713	
Peru	Incidence	Female	2018	11.22875	13.70498	9.441192	
Peru	Incidence	Female	2019	11.62693	14.34988	9.702276	
Peru	Incidence	Female	2020	11.54593	14.25252	9.622814	
Peru	Incidence	Female	2021	15.44997	19.3799	12.55716	
Peru	Incidence	Female	2022	14.62987	18.80419	11.37885	
Peru	Incidence	Female	2023	14.65678	18.87017	11.4262	
Peru	Deaths	Male	2010	8.868635	10.73233	7.363437	
Peru	Deaths	Male	2011	8.627818	10.47549	7.269422	
Peru	Deaths	Male	2012	8.552312	10.14474	7.234593	
Peru	Deaths	Male	2013	8.303638	9.979669	7.125099	
Peru	Deaths	Male	2014	8.09255	9.920953	6.964727	
Peru	Deaths	Male	2015	7.80739	9.711239	6.697147	
Peru	Deaths	Male	2016	7.995509	10.06531	6.831892	
Peru	Deaths	Male	2017	8.850177	11.22708	7.506868	
Peru	Deaths	Male	2018	9.300718	12.0499	7.934707	
Peru	Deaths	Male	2019	9.674678	12.72279	8.129077	
Peru	Deaths	Male	2020	10.52643	13.47395	9.003954	
Peru	Deaths	Male	2021	12.14027	15.44194	10.22395	
Peru	Deaths	Male	2022	11.25284	14.49457	9.159795	
Peru	Deaths	Male	2023	10.97943	14.26701	8.872427	
Peru	Deaths	Female	2010	7.618209	8.838119	6.547312	
Peru	Deaths	Female	2011	7.590633	8.785448	6.573316	
Peru	Deaths	Female	2012	7.61592	8.855719	6.652815	
Peru	Deaths	Female	2013	7.550997	8.936503	6.569631	
Peru	Deaths	Female	2014	7.430639	8.992861	6.379126	
Peru	Deaths	Female	2015	7.372127	8.809621	6.374106	
Peru	Deaths	Female	2016	7.759919	9.397031	6.664064	

Peru	Deaths	Female	2017	8.649747	10.48704	7.40888	
Peru	Deaths	Female	2018	9.127378	11.11456	7.762723	
Peru	Deaths	Female	2019	9.421987	11.67649	7.941464	
Peru	Deaths	Female	2020	9.236646	11.17641	7.863667	
Peru	Deaths	Female	2021	12.08853	14.87133	9.989008	
Peru	Deaths	Female	2022	11.50571	14.33474	9.286308	
Peru	Deaths	Female	2023	11.43848	14.29076	9.276814	
Peru	DALYs	Male	2010	209.3244	257.6818	176.4734	
Peru	DALYs	Male	2011	203.634	248.2669	172.7214	
Peru	DALYs	Male	2012	200.0575	242.2839	171.8626	
Peru	DALYs	Male	2013	193.1037	236.3971	167.1272	
Peru	DALYs	Male	2014	187.7815	234.2766	164.3485	
Peru	DALYs	Male	2015	181.547	228.7084	158.0171	
Peru	DALYs	Male	2016	186.6289	237.5398	161.9927	
Peru	DALYs	Male	2017	202.3117	257.4537	172.5543	
Peru	DALYs	Male	2018	214.2839	279.2605	182.2727	
Peru	DALYs	Male	2019	223.7832	295.4662	188.6397	
Peru	DALYs	Male	2020	241.4376	313.7904	204.2554	
Peru	DALYs	Male	2021	281.855	363.493	235.7168	
Peru	DALYs	Male	2022	257.8457	334.8943	211.4702	
Peru	DALYs	Male	2023	252.3504	329.5501	204.4795	
Peru	DALYs	Female	2010	184.2472	214.1364	161.1152	
Peru	DALYs	Female	2011	183.7512	212.2101	162.1428	
Peru	DALYs	Female	2012	182.8143	211.5303	161.3776	
Peru	DALYs	Female	2013	180.3855	208.3579	158.8797	
Peru	DALYs	Female	2014	177.3768	208.3382	155.6931	
Peru	DALYs	Female	2015	175.9822	208.1682	155.2124	
Peru	DALYs	Female	2016	185.2183	222.1466	162.1889	
Peru	DALYs	Female	2017	203.308	244.7176	177.4115	
Peru	DALYs	Female	2018	215.9759	258.4395	188.1671	
Peru	DALYs	Female	2019	223.3708	269.9532	193.3228	
Peru	DALYs	Female	2020	218.7925	260.1713	189.2582	
Peru	DALYs	Female	2021	289.6071	347.7459	244.8091	
Peru	DALYs	Female	2022	267.8353	326.7238	222.8258	
Peru	DALYs	Female	2023	267.1644	326.682	222.5633	
Philippines	Incidence	Male	2010	13.85541	16.32195	10.68543	

Philippines	Incidence	Male	2011	13.62588	16.07663	10.80126	
Philippines	Incidence	Male	2012	13.73719	16.23703	11.00103	
Philippines	Incidence	Male	2013	13.74192	15.99589	11.14264	
Philippines	Incidence	Male	2014	13.5588	15.92951	11.35926	
Philippines	Incidence	Male	2015	13.59065	16.20572	11.58717	
Philippines	Incidence	Male	2016	13.52182	16.62697	11.64732	
Philippines	Incidence	Male	2017	13.22531	16.45201	11.39572	
Philippines	Incidence	Male	2018	13.13654	16.93469	11.44255	
Philippines	Incidence	Male	2019	13.10555	17.52478	11.2932	
Philippines	Incidence	Male	2020	12.42739	16.79209	10.63044	
Philippines	Incidence	Male	2021	14.39305	19.45954	12.34615	
Philippines	Incidence	Male	2022	13.63969	18.57183	11.49849	
Philippines	Incidence	Male	2023	13.00304	17.27293	10.79564	
Philippines	Incidence	Female	2010	5.783268	7.198798	4.710304	
Philippines	Incidence	Female	2011	5.799741	7.152052	4.791368	
Philippines	Incidence	Female	2012	5.938937	7.244553	4.788689	
Philippines	Incidence	Female	2013	6.052254	7.355619	4.88503	
Philippines	Incidence	Female	2014	6.09774	7.264081	4.887801	
Philippines	Incidence	Female	2015	6.205676	7.598598	5.056007	
Philippines	Incidence	Female	2016	6.299446	7.692363	5.141911	
Philippines	Incidence	Female	2017	6.289065	7.628471	5.272772	
Philippines	Incidence	Female	2018	6.329527	7.738899	5.244188	
Philippines	Incidence	Female	2019	6.447498	8.12509	5.308319	
Philippines	Incidence	Female	2020	6.283591	7.980598	5.190266	
Philippines	Incidence	Female	2021	7.27184	9.27637	5.867783	
Philippines	Incidence	Female	2022	7.050129	9.082387	5.628249	
Philippines	Incidence	Female	2023	6.886144	8.794433	5.511379	
Philippines	Deaths	Male	2010	13.63432	16.06341	10.52823	
Philippines	Deaths	Male	2011	13.42091	15.82467	10.59149	
Philippines	Deaths	Male	2012	13.55098	16.02176	10.90444	
Philippines	Deaths	Male	2013	13.5712	15.78349	11.02785	
Philippines	Deaths	Male	2014	13.40023	15.71978	11.24575	
Philippines	Deaths	Male	2015	13.42807	15.96732	11.39041	
Philippines	Deaths	Male	2016	13.35889	16.36846	11.50226	
Philippines	Deaths	Male	2017	13.07501	16.20088	11.25955	
Philippines	Deaths	Male	2018	12.99845	16.7006	11.34763	

Philippines	Deaths	Male	2019	12.96756	17.2731	11.11669	
Philippines	Deaths	Male	2020	12.29535	16.57908	10.52738	
Philippines	Deaths	Male	2021	14.30594	19.31665	12.21029	
Philippines	Deaths	Male	2022	13.44774	18.23844	11.36584	
Philippines	Deaths	Male	2023	12.83361	16.95771	10.69669	
Philippines	Deaths	Female	2010	5.72667	7.130896	4.667001	
Philippines	Deaths	Female	2011	5.744539	7.131529	4.746916	
Philippines	Deaths	Female	2012	5.889698	7.220267	4.729497	
Philippines	Deaths	Female	2013	6.008718	7.3374	4.855387	
Philippines	Deaths	Female	2014	6.06357	7.256998	4.869689	
Philippines	Deaths	Female	2015	6.17076	7.550255	5.022997	
Philippines	Deaths	Female	2016	6.263888	7.642488	5.134516	
Philippines	Deaths	Female	2017	6.256195	7.548029	5.220553	
Philippines	Deaths	Female	2018	6.29665	7.691997	5.201425	
Philippines	Deaths	Female	2019	6.408292	8.077057	5.28971	
Philippines	Deaths	Female	2020	6.229214	7.915776	5.145387	
Philippines	Deaths	Female	2021	7.247438	9.271393	5.887035	
Philippines	Deaths	Female	2022	6.98081	9.012758	5.556291	
Philippines	Deaths	Female	2023	6.816812	8.679823	5.423245	
Philippines	DALYs	Male	2010	386.2162	458.1928	301.2175	
Philippines	DALYs	Male	2011	378.7356	450.0228	304.7678	
Philippines	DALYs	Male	2012	379.8239	451.7858	307.5606	
Philippines	DALYs	Male	2013	378.6369	446.9215	308.3105	
Philippines	DALYs	Male	2014	372.9184	445.5151	314.6672	
Philippines	DALYs	Male	2015	373.9646	453.8444	320.0383	
Philippines	DALYs	Male	2016	372.0385	466.0644	318.6961	
Philippines	DALYs	Male	2017	363.0591	460.5005	312.3868	
Philippines	DALYs	Male	2018	359.3304	471.7349	311.6993	
Philippines	DALYs	Male	2019	357.5645	484.8971	307.1121	
Philippines	DALYs	Male	2020	338.3909	461.0375	288.7992	
Philippines	DALYs	Male	2021	386.7705	526.6455	329.7378	
Philippines	DALYs	Male	2022	372.5461	516.9847	310.5732	
Philippines	DALYs	Male	2023	353.8917	480.6009	291.1396	
Philippines	DALYs	Female	2010	159.3563	198.3783	129.5815	
Philippines	DALYs	Female	2011	159.6008	196.8185	131.6003	
Philippines	DALYs	Female	2012	162.5816	198.4919	133.1204	

Philippines	DALYs	Female	2013	165.1435	200.6453	132.9394	
Philippines	DALYs	Female	2014	165.8693	197.9933	133.2792	
Philippines	DALYs	Female	2015	168.4761	204.3246	136.4262	
Philippines	DALYs	Female	2016	170.7156	208.5271	138.5895	
Philippines	DALYs	Female	2017	169.9837	206.5535	141.3687	
Philippines	DALYs	Female	2018	170.6352	209.2616	141.0014	
Philippines	DALYs	Female	2019	173.4955	218.9931	142.72	
Philippines	DALYs	Female	2020	169.194	214.6825	139.9167	
Philippines	DALYs	Female	2021	192.3945	243.6681	155.6587	
Philippines	DALYs	Female	2022	188.95	242.4284	151.0249	
Philippines	DALYs	Female	2023	183.9085	234.5923	146.7409	
Poland	Incidence	Male	2010	105.5474	110.5832	100.8439	
Poland	Incidence	Male	2011	105.1704	110.3156	100.1608	
Poland	Incidence	Male	2012	105.1357	109.9429	100.7637	
Poland	Incidence	Male	2013	104.8108	109.7554	99.77798	
Poland	Incidence	Male	2014	105.8004	110.665	101.4536	
Poland	Incidence	Male	2015	106.2217	111.4516	101.3534	
Poland	Incidence	Male	2016	104.7148	109.992	99.7942	
Poland	Incidence	Male	2017	104.2867	109.4439	99.3657	
Poland	Incidence	Male	2018	104.5882	110.5189	99.10674	
Poland	Incidence	Male	2019	99.23177	105.4858	94.3413	
Poland	Incidence	Male	2020	96.09737	101.905	91.48153	
Poland	Incidence	Male	2021	90.17757	95.35543	85.58859	
Poland	Incidence	Male	2022	92.26539	97.64273	87.08761	
Poland	Incidence	Male	2023	90.13483	95.69404	85.3234	
Poland	Incidence	Female	2010	37.9845	40.38063	35.32542	
Poland	Incidence	Female	2011	38.86877	41.62026	36.18419	
Poland	Incidence	Female	2012	40.42227	43.06925	37.73601	
Poland	Incidence	Female	2013	42.20722	45.05187	39.5352	
Poland	Incidence	Female	2014	44.66571	47.51688	42.11749	
Poland	Incidence	Female	2015	46.0395	49.00446	43.28744	
Poland	Incidence	Female	2016	46.70187	49.47359	43.93911	
Poland	Incidence	Female	2017	48.22667	51.08381	45.04155	
Poland	Incidence	Female	2018	49.50035	52.25519	46.09244	
Poland	Incidence	Female	2019	50.1372	52.98771	46.65944	
Poland	Incidence	Female	2020	49.81415	52.55723	46.49364	

Poland	Incidence	Female	2021	48.32279	50.94508	45.35343	
Poland	Incidence	Female	2022	49.72591	53.07624	46.35141	
Poland	Incidence	Female	2023	49.33083	52.83368	45.9184	
Poland	Deaths	Male	2010	106.6256	111.7184	101.8612	
Poland	Deaths	Male	2011	106.4065	111.4692	101.0587	
Poland	Deaths	Male	2012	106.632	111.3743	102.2487	
Poland	Deaths	Male	2013	106.6426	111.8705	101.7096	
Poland	Deaths	Male	2014	108.0146	113.1158	103.4192	
Poland	Deaths	Male	2015	108.6677	113.6772	103.8627	
Poland	Deaths	Male	2016	107.3721	112.4699	102.4878	
Poland	Deaths	Male	2017	107.375	112.4822	102.6225	
Poland	Deaths	Male	2018	108.0965	113.8604	102.5834	
Poland	Deaths	Male	2019	102.7677	108.7933	97.85308	
Poland	Deaths	Male	2020	99.69997	105.4978	95.13336	
Poland	Deaths	Male	2021	93.47256	98.64904	88.90765	
Poland	Deaths	Male	2022	95.97507	101.3914	90.74779	
Poland	Deaths	Male	2023	94.00211	99.58792	88.94326	
Poland	Deaths	Female	2010	37.75888	40.19435	34.99763	
Poland	Deaths	Female	2011	38.61649	41.42869	35.70685	
Poland	Deaths	Female	2012	40.22787	42.87109	37.37989	
Poland	Deaths	Female	2013	42.05346	44.90048	39.2053	
Poland	Deaths	Female	2014	44.58555	47.35867	41.68963	
Poland	Deaths	Female	2015	46.1296	49.09875	43.20839	
Poland	Deaths	Female	2016	47.06714	49.98799	44.0506	
Poland	Deaths	Female	2017	48.9073	51.9989	45.54156	
Poland	Deaths	Female	2018	50.55196	53.44688	46.99089	
Poland	Deaths	Female	2019	51.5943	54.69957	47.77238	
Poland	Deaths	Female	2020	51.44664	54.39691	47.90124	
Poland	Deaths	Female	2021	49.87933	52.56871	46.82366	
Poland	Deaths	Female	2022	51.73729	55.23286	48.00171	
Poland	Deaths	Female	2023	51.41681	55.24505	47.48076	
Poland	DALYs	Male	2010	2667.575	2789.004	2554.587	
Poland	DALYs	Male	2011	2650.183	2776.097	2520.862	
Poland	DALYs	Male	2012	2635.454	2756.184	2522.264	
Poland	DALYs	Male	2013	2606.949	2730.668	2484.664	
Poland	DALYs	Male	2014	2609.827	2746.215	2487.562	

Poland	DALYs	Male	2015	2605.586	2745.659	2481.306	
Poland	DALYs	Male	2016	2554.861	2700.746	2427.738	
Poland	DALYs	Male	2017	2523.635	2663.215	2400.163	
Poland	DALYs	Male	2018	2508.791	2658.794	2375.193	
Poland	DALYs	Male	2019	2365.731	2521.484	2255.042	
Poland	DALYs	Male	2020	2273.377	2419.949	2163.594	
Poland	DALYs	Male	2021	2127.732	2265.403	2014.149	
Poland	DALYs	Male	2022	2155.534	2300.826	2028.854	
Poland	DALYs	Male	2023	2087.833	2220.792	1969.483	
Poland	DALYs	Female	2010	928.323	983.1955	867.5149	
Poland	DALYs	Female	2011	943.3131	1003.07	883.9181	
Poland	DALYs	Female	2012	973.1633	1033.209	913.7258	
Poland	DALYs	Female	2013	1009.075	1070.929	953.419	
Poland	DALYs	Female	2014	1057.893	1117.928	1005.39	
Poland	DALYs	Female	2015	1080.803	1141.697	1027.186	
Poland	DALYs	Female	2016	1087.687	1146.846	1033.201	
Poland	DALYs	Female	2017	1115.31	1177.43	1053.944	
Poland	DALYs	Female	2018	1133.597	1191.421	1070.213	
Poland	DALYs	Female	2019	1137.067	1198.707	1066.13	
Poland	DALYs	Female	2020	1120.648	1179.576	1055.121	
Poland	DALYs	Female	2021	1085.153	1137.805	1027.642	
Poland	DALYs	Female	2022	1104.349	1179.262	1034.521	
Poland	DALYs	Female	2023	1090.626	1168.234	1017.02	
Portugal	Incidence	Male	2010	75.43293	80.54721	70.74558	
Portugal	Incidence	Male	2011	76.89771	83.17417	71.98499	
Portugal	Incidence	Male	2012	78.12274	83.97815	72.95101	
Portugal	Incidence	Male	2013	81.15492	87.27629	75.53881	
Portugal	Incidence	Male	2014	79.4072	85.42163	73.64946	
Portugal	Incidence	Male	2015	77.26841	83.88621	70.9811	
Portugal	Incidence	Male	2016	79.73353	86.34371	72.45596	
Portugal	Incidence	Male	2017	81.24136	88.22154	73.59653	
Portugal	Incidence	Male	2018	82.37061	88.91404	74.99663	
Portugal	Incidence	Male	2019	82.47709	90.19446	74.40272	
Portugal	Incidence	Male	2020	83.94256	92.04478	75.60404	
Portugal	Incidence	Male	2021	81.52484	89.56596	73.61102	
Portugal	Incidence	Male	2022	85.60204	95.1037	76.90519	

Portugal	Incidence	Male	2023	84.34201	94.06416	75.35326	
Portugal	Incidence	Female	2010	18.95585	21.4176	16.71229	
Portugal	Incidence	Female	2011	19.63848	22.16956	17.12534	
Portugal	Incidence	Female	2012	20.59844	23.14582	17.91852	
Portugal	Incidence	Female	2013	21.27933	23.94446	18.49408	
Portugal	Incidence	Female	2014	21.75071	24.34264	18.87528	
Portugal	Incidence	Female	2015	22.99714	25.99638	19.93177	
Portugal	Incidence	Female	2016	23.68912	26.84781	20.57083	
Portugal	Incidence	Female	2017	24.07631	27.27124	20.86149	
Portugal	Incidence	Female	2018	25.28068	28.71727	21.74983	
Portugal	Incidence	Female	2019	26.65849	30.37849	22.92012	
Portugal	Incidence	Female	2020	26.92899	30.94675	23.24033	
Portugal	Incidence	Female	2021	25.64657	29.43032	21.88376	
Portugal	Incidence	Female	2022	28.41762	32.38512	24.1336	
Portugal	Incidence	Female	2023	27.84658	31.93293	23.20294	
Portugal	Deaths	Male	2010	65.29639	68.54898	62.00203	
Portugal	Deaths	Male	2011	66.54484	70.09448	63.22793	
Portugal	Deaths	Male	2012	67.68189	71.12137	64.0803	
Portugal	Deaths	Male	2013	70.51602	74.75032	66.56751	
Portugal	Deaths	Male	2014	69.66577	73.70921	65.55716	
Portugal	Deaths	Male	2015	68.10541	72.10422	64.02417	
Portugal	Deaths	Male	2016	70.51009	74.69283	66.03697	
Portugal	Deaths	Male	2017	72.13908	76.53131	67.57732	
Portugal	Deaths	Male	2018	73.57218	78.19949	68.911	
Portugal	Deaths	Male	2019	73.45408	79.17181	68.45675	
Portugal	Deaths	Male	2020	74.62422	80.58675	69.65133	
Portugal	Deaths	Male	2021	72.43483	78.55703	66.95659	
Portugal	Deaths	Male	2022	76.09808	83.12162	69.89588	
Portugal	Deaths	Male	2023	75.03066	82.04714	68.23222	
Portugal	Deaths	Female	2010	16.6737	18.33454	14.82893	
Portugal	Deaths	Female	2011	17.15337	18.95132	15.18008	
Portugal	Deaths	Female	2012	18.00026	19.82234	15.8643	
Portugal	Deaths	Female	2013	18.48118	20.36516	16.19085	
Portugal	Deaths	Female	2014	18.76678	20.57058	16.50466	
Portugal	Deaths	Female	2015	19.83784	21.63174	17.3809	
Portugal	Deaths	Female	2016	20.43379	22.41765	18.01046	

Portugal	Deaths	Female	2017	20.8443	22.96528	18.27595	
Portugal	Deaths	Female	2018	21.92447	24.275	19.21243	
Portugal	Deaths	Female	2019	22.95354	25.49951	20.08863	
Portugal	Deaths	Female	2020	23.16285	25.71126	20.37645	
Portugal	Deaths	Female	2021	21.98246	24.47529	19.19744	
Portugal	Deaths	Female	2022	24.29636	27.06217	21.21255	
Portugal	Deaths	Female	2023	23.77786	26.75334	20.26799	
Portugal	DALYs	Male	2010	1581.251	1659.681	1512.243	
Portugal	DALYs	Male	2011	1610.364	1692.685	1542.822	
Portugal	DALYs	Male	2012	1628.786	1712.136	1559.675	
Portugal	DALYs	Male	2013	1682.6	1782.64	1599.413	
Portugal	DALYs	Male	2014	1627.42	1719.529	1549.593	
Portugal	DALYs	Male	2015	1574.81	1666.459	1489.949	
Portugal	DALYs	Male	2016	1619.702	1712.018	1519.745	
Portugal	DALYs	Male	2017	1644.399	1741.096	1548.772	
Portugal	DALYs	Male	2018	1657.977	1765.361	1555.986	
Portugal	DALYs	Male	2019	1656.153	1791.823	1544.529	
Portugal	DALYs	Male	2020	1674.281	1820.61	1564.154	
Portugal	DALYs	Male	2021	1617.638	1778.271	1495.675	
Portugal	DALYs	Male	2022	1693.193	1850.821	1555.062	
Portugal	DALYs	Male	2023	1663.51	1821.955	1520.865	
Portugal	DALYs	Female	2010	364.895	398.8428	329.0625	
Portugal	DALYs	Female	2011	378.3302	414.7078	340.0273	
Portugal	DALYs	Female	2012	393.0667	428.7992	352.0407	
Portugal	DALYs	Female	2013	404.1356	441.5451	364.4955	
Portugal	DALYs	Female	2014	411.4036	448.7225	373.0927	
Portugal	DALYs	Female	2015	431.5579	467.474	392.5295	
Portugal	DALYs	Female	2016	442.0856	482.4927	401.9734	
Portugal	DALYs	Female	2017	445.172	488.7751	402.7353	
Portugal	DALYs	Female	2018	464.8547	511.4108	416.6925	
Portugal	DALYs	Female	2019	488.4455	535.0599	439.9535	
Portugal	DALYs	Female	2020	486.6052	537.5344	442.9585	
Portugal	DALYs	Female	2021	460.7949	506.6478	415.9138	
Portugal	DALYs	Female	2022	509.1512	568.1859	456.4955	
Portugal	DALYs	Female	2023	497.7318	553.8288	438.3073	
Puerto Rico	Incidence	Male	2010	29.35719	32.11188	27.2495	

Puerto Rico	Incidence	Male	2011	28.60558	31.30495	26.57734	
Puerto Rico	Incidence	Male	2012	29.03993	31.71991	26.79943	
Puerto Rico	Incidence	Male	2013	28.89583	31.59722	26.49683	
Puerto Rico	Incidence	Male	2014	29.2955	32.1477	26.94682	
Puerto Rico	Incidence	Male	2015	29.24159	31.94865	26.81086	
Puerto Rico	Incidence	Male	2016	27.65656	30.49842	25.47152	
Puerto Rico	Incidence	Male	2017	28.23067	31.23354	25.94271	
Puerto Rico	Incidence	Male	2018	27.32457	30.17382	24.83798	
Puerto Rico	Incidence	Male	2019	27.26084	30.07649	24.86113	
Puerto Rico	Incidence	Male	2020	28.81031	31.46754	26.45625	
Puerto Rico	Incidence	Male	2021	28.90615	31.61727	26.40174	
Puerto Rico	Incidence	Male	2022	28.94177	32.12132	26.1862	
Puerto Rico	Incidence	Male	2023	31.09275	34.87039	27.99058	
Puerto Rico	Incidence	Female	2010	16.82119	19.76768	14.70051	
Puerto Rico	Incidence	Female	2011	17.62935	21.02215	15.31869	
Puerto Rico	Incidence	Female	2012	18.92836	22.43856	16.26938	
Puerto Rico	Incidence	Female	2013	18.91936	22.1381	16.24977	
Puerto Rico	Incidence	Female	2014	18.63785	21.87988	15.79481	
Puerto Rico	Incidence	Female	2015	18.69665	21.88232	15.82109	
Puerto Rico	Incidence	Female	2016	18.32799	21.24119	15.47298	
Puerto Rico	Incidence	Female	2017	18.81998	21.64865	15.87378	
Puerto Rico	Incidence	Female	2018	19.09046	21.96802	15.9936	
Puerto Rico	Incidence	Female	2019	19.43993	22.37682	16.37411	
Puerto Rico	Incidence	Female	2020	20.16421	23.23336	16.99559	
Puerto Rico	Incidence	Female	2021	20.04797	23.44538	16.87219	
Puerto Rico	Incidence	Female	2022	20.5371	24.08844	17.31892	
Puerto Rico	Incidence	Female	2023	23.11157	27.9254	19.36347	
Puerto Rico	Deaths	Male	2010	28.01965	30.29635	26.32517	
Puerto Rico	Deaths	Male	2011	27.27344	29.4696	25.45509	
Puerto Rico	Deaths	Male	2012	27.6813	29.99542	25.63119	
Puerto Rico	Deaths	Male	2013	27.49021	29.61967	25.41263	
Puerto Rico	Deaths	Male	2014	27.78582	30.1024	25.78077	
Puerto Rico	Deaths	Male	2015	27.52575	29.62511	25.33257	
Puerto Rico	Deaths	Male	2016	26.01282	28.19822	24.06826	
Puerto Rico	Deaths	Male	2017	26.65366	28.92743	24.84204	
Puerto Rico	Deaths	Male	2018	25.88812	28.09945	23.79317	

Puerto Rico	Deaths	Male	2019	25.83022	28.22087	23.95418	
Puerto Rico	Deaths	Male	2020	27.21334	29.54246	25.25969	
Puerto Rico	Deaths	Male	2021	27.34836	29.56064	25.30918	
Puerto Rico	Deaths	Male	2022	27.44761	29.90452	25.37015	
Puerto Rico	Deaths	Male	2023	29.54388	32.33812	27.03914	
Puerto Rico	Deaths	Female	2010	14.01641	15.68439	12.59433	
Puerto Rico	Deaths	Female	2011	14.6926	16.79513	13.14765	
Puerto Rico	Deaths	Female	2012	15.75086	18.11631	14.03125	
Puerto Rico	Deaths	Female	2013	15.60465	17.82168	13.81091	
Puerto Rico	Deaths	Female	2014	15.27698	17.38892	13.51821	
Puerto Rico	Deaths	Female	2015	15.21614	17.26177	13.55529	
Puerto Rico	Deaths	Female	2016	14.95208	16.83871	13.21396	
Puerto Rico	Deaths	Female	2017	15.46975	17.42618	13.56696	
Puerto Rico	Deaths	Female	2018	15.63524	17.64974	13.67635	
Puerto Rico	Deaths	Female	2019	15.92832	17.83727	13.89193	
Puerto Rico	Deaths	Female	2020	16.47406	18.26588	14.58638	
Puerto Rico	Deaths	Female	2021	16.32299	18.45385	14.58675	
Puerto Rico	Deaths	Female	2022	16.74779	19.24553	14.95397	
Puerto Rico	Deaths	Female	2023	18.78188	21.71287	16.50796	
Puerto Rico	DALYs	Male	2010	618.723	670.8649	581.2075	
Puerto Rico	DALYs	Male	2011	603.3533	654.6338	564.8808	
Puerto Rico	DALYs	Male	2012	606.2696	658.2238	564.0876	
Puerto Rico	DALYs	Male	2013	595.5401	645.5229	553.5519	
Puerto Rico	DALYs	Male	2014	600.8946	651.3065	556.667	
Puerto Rico	DALYs	Male	2015	604.0842	653.9875	560.7529	
Puerto Rico	DALYs	Male	2016	572.1353	621.4532	533.3182	
Puerto Rico	DALYs	Male	2017	576.8111	631.0803	537.1296	
Puerto Rico	DALYs	Male	2018	552.8778	602.4565	511.4243	
Puerto Rico	DALYs	Male	2019	548.502	599.2265	509.6845	
Puerto Rico	DALYs	Male	2020	577.3973	630.3023	536.7867	
Puerto Rico	DALYs	Male	2021	572.6424	620.8148	533.6345	
Puerto Rico	DALYs	Male	2022	566.3934	624.3	520.2405	
Puerto Rico	DALYs	Male	2023	598.1677	663.6199	548.528	
Puerto Rico	DALYs	Female	2010	299.2428	335.8297	271.9739	
Puerto Rico	DALYs	Female	2011	310.5518	355.5848	282.662	
Puerto Rico	DALYs	Female	2012	327.881	375.7108	297.1652	

Puerto Rico	DALYs	Female	2013	324.2964	371.8047	290.734	
Puerto Rico	DALYs	Female	2014	316.5518	363.2438	281.5445	
Puerto Rico	DALYs	Female	2015	315.2812	355.2846	282.2282	
Puerto Rico	DALYs	Female	2016	306.6571	342.9844	273.316	
Puerto Rico	DALYs	Female	2017	312.8485	351.0453	279.097	
Puerto Rico	DALYs	Female	2018	316.1814	354.8725	281.5586	
Puerto Rico	DALYs	Female	2019	319.3183	355.551	285.2286	
Puerto Rico	DALYs	Female	2020	326.8386	363.1078	292.0937	
Puerto Rico	DALYs	Female	2021	320.5762	362.5187	287.7651	
Puerto Rico	DALYs	Female	2022	321.533	368.5664	286.6742	
Puerto Rico	DALYs	Female	2023	355.1871	408.9055	317.4218	
Qatar	Incidence	Male	2010	2.875317	4.08703	2.117832	
Qatar	Incidence	Male	2011	2.754534	4.018737	2.021611	
Qatar	Incidence	Male	2012	2.754134	4.193353	1.998302	
Qatar	Incidence	Male	2013	2.663627	4.126449	1.916103	
Qatar	Incidence	Male	2014	2.627004	4.033899	1.835336	
Qatar	Incidence	Male	2015	2.830033	4.376785	1.976466	
Qatar	Incidence	Male	2016	2.757104	4.279796	1.916349	
Qatar	Incidence	Male	2017	2.797555	4.511242	1.962443	
Qatar	Incidence	Male	2018	2.939515	4.734789	2.038337	
Qatar	Incidence	Male	2019	2.98841	4.889144	2.031188	
Qatar	Incidence	Male	2020	2.889241	4.748699	2.003949	
Qatar	Incidence	Male	2021	2.944455	4.757357	2.005808	
Qatar	Incidence	Male	2022	3.020325	4.953957	2.037869	
Qatar	Incidence	Male	2023	3.721101	5.899214	2.431788	
Qatar	Incidence	Female	2010	1.64172	2.364047	1.138409	
Qatar	Incidence	Female	2011	1.593731	2.252754	1.083602	
Qatar	Incidence	Female	2012	1.549946	2.238389	1.066285	
Qatar	Incidence	Female	2013	1.502854	2.154659	1.050669	
Qatar	Incidence	Female	2014	1.494313	2.166772	1.009772	
Qatar	Incidence	Female	2015	1.524738	2.195373	1.009786	
Qatar	Incidence	Female	2016	1.459147	2.046759	0.974471	
Qatar	Incidence	Female	2017	1.445171	2.020213	0.99144	
Qatar	Incidence	Female	2018	1.458863	2.09561	1.015481	
Qatar	Incidence	Female	2019	1.478287	2.1157	1.046472	
Qatar	Incidence	Female	2020	1.47589	2.128262	1.047642	

Qatar	Incidence	Female	2021	1.482277	2.145115	1.046208	
Qatar	Incidence	Female	2022	1.558521	2.23271	1.055022	
Qatar	Incidence	Female	2023	1.889399	2.818182	1.294182	
Qatar	Deaths	Male	2010	2.506191	3.53216	1.841979	
Qatar	Deaths	Male	2011	2.392626	3.465979	1.760545	
Qatar	Deaths	Male	2012	2.383978	3.559948	1.741774	
Qatar	Deaths	Male	2013	2.300005	3.544446	1.665479	
Qatar	Deaths	Male	2014	2.261706	3.434647	1.597191	
Qatar	Deaths	Male	2015	2.425274	3.786475	1.697493	
Qatar	Deaths	Male	2016	2.352438	3.677067	1.650782	
Qatar	Deaths	Male	2017	2.374584	3.873398	1.685912	
Qatar	Deaths	Male	2018	2.480225	4.057036	1.73252	
Qatar	Deaths	Male	2019	2.504366	4.152741	1.735055	
Qatar	Deaths	Male	2020	2.401192	3.940915	1.709378	
Qatar	Deaths	Male	2021	2.425246	3.994379	1.686338	
Qatar	Deaths	Male	2022	2.475606	4.088135	1.709904	
Qatar	Deaths	Male	2023	3.044929	4.872596	2.031549	
Qatar	Deaths	Female	2010	1.452111	2.116398	1.031004	
Qatar	Deaths	Female	2011	1.400457	2.016749	0.962423	
Qatar	Deaths	Female	2012	1.352851	1.92869	0.928404	
Qatar	Deaths	Female	2013	1.302432	1.838066	0.910437	
Qatar	Deaths	Female	2014	1.284389	1.828173	0.874104	
Qatar	Deaths	Female	2015	1.300754	1.82538	0.876762	
Qatar	Deaths	Female	2016	1.237226	1.704178	0.836867	
Qatar	Deaths	Female	2017	1.217966	1.683755	0.831154	
Qatar	Deaths	Female	2018	1.220529	1.674229	0.855493	
Qatar	Deaths	Female	2019	1.227973	1.768038	0.874557	
Qatar	Deaths	Female	2020	1.21877	1.760279	0.852032	
Qatar	Deaths	Female	2021	1.209723	1.770075	0.838676	
Qatar	Deaths	Female	2022	1.258565	1.788866	0.850703	
Qatar	Deaths	Female	2023	1.514734	2.292897	1.046504	
Qatar	DALYs	Male	2010	88.68338	126.7371	65.321	
Qatar	DALYs	Male	2011	84.01239	122.9099	61.9308	
Qatar	DALYs	Male	2012	82.92389	125.281	60.9136	
Qatar	DALYs	Male	2013	78.97841	121.3308	57.55535	
Qatar	DALYs	Male	2014	76.51161	115.7367	53.71746	

Qatar	DALYs	Male	2015	81.12883	127.3816	56.91888	
Qatar	DALYs	Male	2016	78.0283	123.2563	54.67174	
Qatar	DALYs	Male	2017	78.40349	130.1848	55.79568	
Qatar	DALYs	Male	2018	81.95347	135.3052	57.43896	
Qatar	DALYs	Male	2019	82.81652	137.5975	57.1773	
Qatar	DALYs	Male	2020	79.41571	131.3877	56.10321	
Qatar	DALYs	Male	2021	80.47022	132.664	55.75832	
Qatar	DALYs	Male	2022	82.48692	134.8324	56.53296	
Qatar	DALYs	Male	2023	101.3229	159.8944	66.38134	
Qatar	DALYs	Female	2010	45.58157	66.78652	32.26834	
Qatar	DALYs	Female	2011	43.88128	63.53082	30.09907	
Qatar	DALYs	Female	2012	42.20425	59.82808	28.93279	
Qatar	DALYs	Female	2013	40.40666	57.67184	28.40665	
Qatar	DALYs	Female	2014	39.61393	56.69561	27.17133	
Qatar	DALYs	Female	2015	39.71561	54.66196	27.09293	
Qatar	DALYs	Female	2016	37.52182	51.98163	25.37532	
Qatar	DALYs	Female	2017	36.72645	50.46165	25.43369	
Qatar	DALYs	Female	2018	36.72033	51.3519	25.88089	
Qatar	DALYs	Female	2019	36.78441	53.77756	26.64037	
Qatar	DALYs	Female	2020	35.93436	53.00061	25.27349	
Qatar	DALYs	Female	2021	35.88069	53.2664	25.29768	
Qatar	DALYs	Female	2022	37.98436	54.76437	25.83668	
Qatar	DALYs	Female	2023	46.16249	69.61307	31.8405	
Korea, Republic	Incidence	Male	2010	64.14324	74.6882	51.72679	
Korea, Republic	Incidence	Male	2011	66.03986	76.38435	53.42277	
Korea, Republic	Incidence	Male	2012	69.18888	79.8174	56.30543	
Korea, Republic	Incidence	Male	2013	70.31119	81.67287	56.61678	
Korea, Republic	Incidence	Male	2014	71.62992	84.33523	57.86678	
Korea, Republic	Incidence	Male	2015	72.33656	84.49578	58.21219	
Korea, Republic	Incidence	Male	2016	73.97989	86.42703	59.70013	
Korea, Republic	Incidence	Male	2017	75.7108	87.58495	60.25289	
Korea, Republic	Incidence	Male	2018	79.18553	92.31835	64.08127	
Korea, Republic	Incidence	Male	2019	79.54142	93.71809	64.34005	
Korea, Republic	Incidence	Male	2020	83.44927	98.00886	67.57177	
Korea, Republic	Incidence	Male	2021	84.58606	99.40363	68.43648	
Korea, Republic	Incidence	Male	2022	92.39226	108.6041	75.05541	

Korea, Republic	Incidence	Male	2023	91.42367	109.4906	73.5111	
Korea, Republic	Incidence	Female	2010	26.3805	31.6071	21.70826	
Korea, Republic	Incidence	Female	2011	26.7375	32.08902	21.76673	
Korea, Republic	Incidence	Female	2012	28.13159	33.72689	22.77893	
Korea, Republic	Incidence	Female	2013	28.49835	33.84266	22.81889	
Korea, Republic	Incidence	Female	2014	28.74633	34.49288	22.32334	
Korea, Republic	Incidence	Female	2015	29.66719	35.87202	23.27098	
Korea, Republic	Incidence	Female	2016	30.12017	36.13671	23.07178	
Korea, Republic	Incidence	Female	2017	30.66421	37.33519	23.52506	
Korea, Republic	Incidence	Female	2018	31.69376	38.40747	23.86965	
Korea, Republic	Incidence	Female	2019	31.37205	38.68274	23.64773	
Korea, Republic	Incidence	Female	2020	32.50036	39.92512	24.5383	
Korea, Republic	Incidence	Female	2021	32.53918	40.12377	24.60715	
Korea, Republic	Incidence	Female	2022	36.74681	44.69206	28.14052	
Korea, Republic	Incidence	Female	2023	37.96935	45.55683	28.64752	
Korea, Republic	Deaths	Male	2010	51.53376	57.94217	40.30481	
Korea, Republic	Deaths	Male	2011	52.6207	58.77329	41.51449	
Korea, Republic	Deaths	Male	2012	54.55636	61.04646	42.5464	
Korea, Republic	Deaths	Male	2013	54.65568	61.58034	43.10882	
Korea, Republic	Deaths	Male	2014	55.01136	61.75361	43.6128	
Korea, Republic	Deaths	Male	2015	55.08295	62.07433	43.22502	
Korea, Republic	Deaths	Male	2016	55.9197	63.23573	44.16742	
Korea, Republic	Deaths	Male	2017	56.94788	63.8915	45.61537	
Korea, Republic	Deaths	Male	2018	59.37047	67.13635	47.95113	
Korea, Republic	Deaths	Male	2019	59.40542	68.00245	48.3301	
Korea, Republic	Deaths	Male	2020	62.30417	71.19725	50.61148	
Korea, Republic	Deaths	Male	2021	63.1842	73.14863	51.652	
Korea, Republic	Deaths	Male	2022	69.27877	79.70862	55.31675	
Korea, Republic	Deaths	Male	2023	68.42224	79.71254	54.84817	
Korea, Republic	Deaths	Female	2010	20.57193	24.14072	16.9692	
Korea, Republic	Deaths	Female	2011	20.64474	24.43905	16.63723	
Korea, Republic	Deaths	Female	2012	21.47413	25.10516	17.37127	
Korea, Republic	Deaths	Female	2013	21.35028	24.82873	17.03968	
Korea, Republic	Deaths	Female	2014	21.25718	25.29756	16.98785	
Korea, Republic	Deaths	Female	2015	21.78665	25.94075	17.35938	
Korea, Republic	Deaths	Female	2016	21.97588	25.83726	17.11583	

Korea, Republic	Deaths	Female	2017	22.36508	26.46164	17.45172	
Korea, Republic	Deaths	Female	2018	23.10577	27.07136	18.11502	
Korea, Republic	Deaths	Female	2019	22.79001	27.22202	17.95769	
Korea, Republic	Deaths	Female	2020	23.64099	28.29663	18.73558	
Korea, Republic	Deaths	Female	2021	23.69042	28.71268	18.86712	
Korea, Republic	Deaths	Female	2022	27.00668	31.84216	21.71741	
Korea, Republic	Deaths	Female	2023	27.88423	33.11953	21.298	
Korea, Republic	DALYs	Male	2010	1148.323	1288.028	921.3549	
Korea, Republic	DALYs	Male	2011	1159.376	1305.871	937.3853	
Korea, Republic	DALYs	Male	2012	1185.335	1349.99	949.1438	
Korea, Republic	DALYs	Male	2013	1178.072	1337.649	947.6126	
Korea, Republic	DALYs	Male	2014	1173.838	1320.21	944.8437	
Korea, Republic	DALYs	Male	2015	1158.223	1325.194	943.8751	
Korea, Republic	DALYs	Male	2016	1161.396	1332.126	948.339	
Korea, Republic	DALYs	Male	2017	1167.83	1340.496	976.4869	
Korea, Republic	DALYs	Male	2018	1200.634	1397.953	1004.44	
Korea, Republic	DALYs	Male	2019	1194.213	1397.306	1005.575	
Korea, Republic	DALYs	Male	2020	1231.177	1435.463	1035.506	
Korea, Republic	DALYs	Male	2021	1238.437	1454.96	1036.191	
Korea, Republic	DALYs	Male	2022	1337.073	1574.208	1113.507	
Korea, Republic	DALYs	Male	2023	1317.053	1538.078	1098.18	
Korea, Republic	DALYs	Female	2010	411.0811	480.8054	349.6879	
Korea, Republic	DALYs	Female	2011	407.7254	477.2953	344.2434	
Korea, Republic	DALYs	Female	2012	417.2247	479.1903	352.522	
Korea, Republic	DALYs	Female	2013	414.5228	478.2686	342.9145	
Korea, Republic	DALYs	Female	2014	409.1624	486.4193	337.3328	
Korea, Republic	DALYs	Female	2015	412.1764	493.3026	330.7824	
Korea, Republic	DALYs	Female	2016	410.5976	495.2816	328.3824	
Korea, Republic	DALYs	Female	2017	409.6244	507.6818	326.9402	
Korea, Republic	DALYs	Female	2018	416.8982	516.7121	331.4312	
Korea, Republic	DALYs	Female	2019	411.3604	514.3303	328.9991	
Korea, Republic	DALYs	Female	2020	420.6723	530.042	332.1522	
Korea, Republic	DALYs	Female	2021	418.8149	527.9066	333.8904	
Korea, Republic	DALYs	Female	2022	461.6394	581.772	373.2091	
Korea, Republic	DALYs	Female	2023	476.1593	586.9031	376.4219	
Republic of Mo	Incidence	Male	2010	47.92177	51.06586	45.01056	

Republic of Mo	Incidence	Male	2011	49.96323	52.96837	47.06409	
Republic of Mo	Incidence	Male	2012	50.04742	53.32742	47.27163	
Republic of Mo	Incidence	Male	2013	50.99708	53.99182	47.71151	
Republic of Mo	Incidence	Male	2014	51.09227	54.48158	47.81494	
Republic of Mo	Incidence	Male	2015	51.80382	55.43094	48.65337	
Republic of Mo	Incidence	Male	2016	53.17278	56.97627	49.63846	
Republic of Mo	Incidence	Male	2017	51.60707	55.75794	47.52893	
Republic of Mo	Incidence	Male	2018	53.56415	58.35344	49.78016	
Republic of Mo	Incidence	Male	2019	50.95829	55.47761	47.29287	
Republic of Mo	Incidence	Male	2020	48.03607	53.32863	44.56671	
Republic of Mo	Incidence	Male	2021	47.8651	51.9454	44.74837	
Republic of Mo	Incidence	Male	2022	52.35407	57.63484	48.08405	
Republic of Mo	Incidence	Male	2023	52.03262	58.82835	47.00715	
Republic of Mo	Incidence	Female	2010	10.12219	11.12548	9.134082	
Republic of Mo	Incidence	Female	2011	10.40948	11.55327	9.392983	
Republic of Mo	Incidence	Female	2012	10.66478	11.9934	9.497949	
Republic of Mo	Incidence	Female	2013	10.98324	12.31156	9.80292	
Republic of Mo	Incidence	Female	2014	11.0842	12.37642	9.889495	
Republic of Mo	Incidence	Female	2015	10.76249	12.1202	9.503839	
Republic of Mo	Incidence	Female	2016	10.77	12.22066	9.613469	
Republic of Mo	Incidence	Female	2017	11.2052	12.89766	9.927484	
Republic of Mo	Incidence	Female	2018	11.34688	13.00741	10.00196	
Republic of Mo	Incidence	Female	2019	10.98172	12.85787	9.691047	
Republic of Mo	Incidence	Female	2020	10.00886	11.75374	8.690703	
Republic of Mo	Incidence	Female	2021	9.664203	11.3722	8.370076	
Republic of Mo	Incidence	Female	2022	10.53533	12.50313	9.024259	
Republic of Mo	Incidence	Female	2023	11.44919	13.58065	9.816663	
Republic of Mo	Deaths	Male	2010	44.87936	47.7505	42.36347	
Republic of Mo	Deaths	Male	2011	46.7257	49.61893	44.16193	
Republic of Mo	Deaths	Male	2012	46.76665	49.81958	44.40637	
Republic of Mo	Deaths	Male	2013	47.52367	50.35287	44.70064	
Republic of Mo	Deaths	Male	2014	47.63626	50.68352	44.80255	
Republic of Mo	Deaths	Male	2015	48.41983	51.72629	45.645	
Republic of Mo	Deaths	Male	2016	49.60734	52.97156	46.45759	
Republic of Mo	Deaths	Male	2017	48.0109	52.20192	44.3863	
Republic of Mo	Deaths	Male	2018	49.72193	53.75759	46.16438	

Republic of Mo	Deaths	Male	2019	47.23618	51.30399	44.20891	
Republic of Mo	Deaths	Male	2020	44.52297	48.49508	41.55142	
Republic of Mo	Deaths	Male	2021	44.42224	48.0649	41.85081	
Republic of Mo	Deaths	Male	2022	48.52347	53.20325	44.59403	
Republic of Mo	Deaths	Male	2023	48.1426	53.74557	43.88445	
Republic of Mo	Deaths	Female	2010	9.707651	10.64123	8.776282	
Republic of Mo	Deaths	Female	2011	9.930753	11.04158	8.991135	
Republic of Mo	Deaths	Female	2012	10.14555	11.20605	9.074303	
Republic of Mo	Deaths	Female	2013	10.44086	11.56499	9.372694	
Republic of Mo	Deaths	Female	2014	10.54105	11.79949	9.372532	
Republic of Mo	Deaths	Female	2015	10.26882	11.5025	9.166395	
Republic of Mo	Deaths	Female	2016	10.25012	11.5663	9.08711	
Republic of Mo	Deaths	Female	2017	10.63515	12.13719	9.509186	
Republic of Mo	Deaths	Female	2018	10.75918	12.2834	9.579899	
Republic of Mo	Deaths	Female	2019	10.39433	11.96455	9.224983	
Republic of Mo	Deaths	Female	2020	9.469436	11.03909	8.310808	
Republic of Mo	Deaths	Female	2021	9.148432	10.61084	8.025242	
Republic of Mo	Deaths	Female	2022	9.969809	11.83468	8.631188	
Republic of Mo	Deaths	Female	2023	10.78361	12.63483	9.293951	
Republic of Mo	DALYs	Male	2010	1311.362	1405.799	1235.527	
Republic of Mo	DALYs	Male	2011	1351.981	1441.765	1271.809	
Republic of Mo	DALYs	Male	2012	1341.312	1432.99	1270.109	
Republic of Mo	DALYs	Male	2013	1363.414	1447.489	1280.901	
Republic of Mo	DALYs	Male	2014	1359.758	1453.638	1271.754	
Republic of Mo	DALYs	Male	2015	1364.316	1462.777	1281.986	
Republic of Mo	DALYs	Male	2016	1390.666	1490.715	1301.823	
Republic of Mo	DALYs	Male	2017	1342.044	1466.161	1244.072	
Republic of Mo	DALYs	Male	2018	1379.261	1503.364	1278.959	
Republic of Mo	DALYs	Male	2019	1302.777	1422.799	1226.669	
Republic of Mo	DALYs	Male	2020	1221.595	1338.078	1139.161	
Republic of Mo	DALYs	Male	2021	1199.555	1317.066	1126.49	
Republic of Mo	DALYs	Male	2022	1308.564	1440.842	1204.464	
Republic of Mo	DALYs	Male	2023	1294.533	1450.838	1173.709	
Republic of Mo	DALYs	Female	2010	246.1841	273.605	221.6107	
Republic of Mo	DALYs	Female	2011	252.4019	280.3524	226.8381	
Republic of Mo	DALYs	Female	2012	256.3683	286.0172	228.2613	

Republic of Mo	DALYs	Female	2013	262.4324	292.2124	234.4465	
Republic of Mo	DALYs	Female	2014	264.3396	296.4583	236.5435	
Republic of Mo	DALYs	Female	2015	255.3362	289.3798	227.2291	
Republic of Mo	DALYs	Female	2016	253.8555	288.8798	225.7629	
Republic of Mo	DALYs	Female	2017	261.9953	300.0742	232.834	
Republic of Mo	DALYs	Female	2018	262.4499	303.3905	231.3502	
Republic of Mo	DALYs	Female	2019	252.4183	295.9814	220.5782	
Republic of Mo	DALYs	Female	2020	228.902	267.7347	199.8433	
Republic of Mo	DALYs	Female	2021	216.159	253.6136	189.5145	
Republic of Mo	DALYs	Female	2022	233.9105	282.1814	202.2143	
Republic of Mo	DALYs	Female	2023	254.2737	301.1965	218.7094	
Romania	Incidence	Male	2010	81.49858	85.60677	77.39652	
Romania	Incidence	Male	2011	84.8797	89.53152	80.43989	
Romania	Incidence	Male	2012	84.8973	89.55009	80.36497	
Romania	Incidence	Male	2013	86.05799	90.65817	81.44797	
Romania	Incidence	Male	2014	89.53757	93.96966	84.47685	
Romania	Incidence	Male	2015	89.39348	93.81362	84.30153	
Romania	Incidence	Male	2016	88.88212	93.65374	84.03849	
Romania	Incidence	Male	2017	89.14224	94.5725	84.08172	
Romania	Incidence	Male	2018	88.6995	94.42315	82.73689	
Romania	Incidence	Male	2019	84.86902	91.67581	79.22313	
Romania	Incidence	Male	2020	83.78838	91.53261	78.33568	
Romania	Incidence	Male	2021	81.80235	90.21599	75.8664	
Romania	Incidence	Male	2022	86.92199	97.29546	80.05881	
Romania	Incidence	Male	2023	84.18409	93.8194	76.69867	
Romania	Incidence	Female	2010	19.74277	20.95062	18.45506	
Romania	Incidence	Female	2011	20.88931	22.12122	19.53905	
Romania	Incidence	Female	2012	21.76234	22.93291	20.41481	
Romania	Incidence	Female	2013	22.66891	24.00362	21.29335	
Romania	Incidence	Female	2014	23.49113	24.87232	21.78902	
Romania	Incidence	Female	2015	24.3536	25.92819	22.71282	
Romania	Incidence	Female	2016	24.98779	26.74152	23.01644	
Romania	Incidence	Female	2017	25.87466	27.95803	23.9473	
Romania	Incidence	Female	2018	26.4191	28.57142	24.21973	
Romania	Incidence	Female	2019	26.90126	29.74715	24.5456	
Romania	Incidence	Female	2020	26.86675	30.17184	24.10616	

Romania	Incidence	Female	2021	27.89318	31.53863	24.87549	
Romania	Incidence	Female	2022	27.9458	32.05239	24.61227	
Romania	Incidence	Female	2023	27.3237	31.32308	23.8463	
Romania	Deaths	Male	2010	79.16243	82.6828	75.27714	
Romania	Deaths	Male	2011	82.50252	87.04807	77.93339	
Romania	Deaths	Male	2012	82.62161	87.22027	78.24884	
Romania	Deaths	Male	2013	83.82994	88.25155	79.66339	
Romania	Deaths	Male	2014	87.2911	91.58551	82.84208	
Romania	Deaths	Male	2015	87.3251	91.53096	82.39847	
Romania	Deaths	Male	2016	86.97402	91.28979	82.01447	
Romania	Deaths	Male	2017	87.3467	92.17109	82.57189	
Romania	Deaths	Male	2018	86.94703	92.48829	81.4723	
Romania	Deaths	Male	2019	83.33895	89.67905	78.34505	
Romania	Deaths	Male	2020	82.2942	89.75581	77.41951	
Romania	Deaths	Male	2021	80.4193	88.41366	75.19306	
Romania	Deaths	Male	2022	85.22563	94.66992	78.73579	
Romania	Deaths	Male	2023	82.31723	91.50413	75.37649	
Romania	Deaths	Female	2010	19.71892	20.92772	18.39112	
Romania	Deaths	Female	2011	20.79001	22.011	19.38509	
Romania	Deaths	Female	2012	21.6695	22.87438	20.29523	
Romania	Deaths	Female	2013	22.56368	23.77398	21.13771	
Romania	Deaths	Female	2014	23.37017	24.71562	21.70281	
Romania	Deaths	Female	2015	24.33033	25.77959	22.79991	
Romania	Deaths	Female	2016	24.96962	26.71512	23.11815	
Romania	Deaths	Female	2017	25.90104	28.02749	24.00312	
Romania	Deaths	Female	2018	26.40588	28.62104	24.38218	
Romania	Deaths	Female	2019	26.91801	29.49006	24.50818	
Romania	Deaths	Female	2020	26.85378	30.07555	24.32266	
Romania	Deaths	Female	2021	27.92616	31.56677	25.12198	
Romania	Deaths	Female	2022	27.98884	32.22813	24.80879	
Romania	Deaths	Female	2023	27.27029	31.51606	23.79988	
Romania	DALYs	Male	2010	2148.017	2242.67	2046.411	
Romania	DALYs	Male	2011	2221.811	2343.023	2103.507	
Romania	DALYs	Male	2012	2203.965	2327.072	2091.579	
Romania	DALYs	Male	2013	2217.444	2334.745	2114.757	
Romania	DALYs	Male	2014	2296.263	2411.804	2177.51	

Romania	DALYs	Male	2015	2278.593	2392.183	2159.256	
Romania	DALYs	Male	2016	2254.892	2367.077	2126.7	
Romania	DALYs	Male	2017	2250.907	2382.233	2124.882	
Romania	DALYs	Male	2018	2229.47	2382.762	2089.565	
Romania	DALYs	Male	2019	2117.768	2287.898	1982.334	
Romania	DALYs	Male	2020	2082.205	2278.716	1952.908	
Romania	DALYs	Male	2021	2021.245	2241.148	1885.758	
Romania	DALYs	Male	2022	2150.347	2428.474	1981.854	
Romania	DALYs	Male	2023	2083.536	2331.076	1899.572	
Romania	DALYs	Female	2010	486.5045	514.4475	455.1537	
Romania	DALYs	Female	2011	515.0435	544.4936	482.8084	
Romania	DALYs	Female	2012	532.5034	558.6329	500.5808	
Romania	DALYs	Female	2013	552.0875	580.1929	520.2717	
Romania	DALYs	Female	2014	571.3024	603.8132	533.29	
Romania	DALYs	Female	2015	586.1766	618.9941	547.8577	
Romania	DALYs	Female	2016	600.0718	642.5884	557.8179	
Romania	DALYs	Female	2017	618.4907	671.7409	576.3481	
Romania	DALYs	Female	2018	632.1397	686.1876	582.2548	
Romania	DALYs	Female	2019	639.345	703.5768	581.9244	
Romania	DALYs	Female	2020	637.4204	715.4712	576.8793	
Romania	DALYs	Female	2021	655.727	743.0651	587.5739	
Romania	DALYs	Female	2022	655.0892	755.215	577.9298	
Romania	DALYs	Female	2023	641.3023	738.298	559.2325	
Russian Federa	Incidence	Male	2010	74.29732	78.8309	69.83281	
Russian Federa	Incidence	Male	2011	72.50166	77.02143	67.93217	
Russian Federa	Incidence	Male	2012	71.89231	76.70749	67.23658	
Russian Federa	Incidence	Male	2013	71.6013	76.19934	66.99074	
Russian Federa	Incidence	Male	2014	71.42271	75.51899	67.34682	
Russian Federa	Incidence	Male	2015	74.0796	78.2835	69.85494	
Russian Federa	Incidence	Male	2016	73.96646	77.80039	70.39518	
Russian Federa	Incidence	Male	2017	71.83913	75.6523	68.25923	
Russian Federa	Incidence	Male	2018	72.65211	76.27159	69.20076	
Russian Federa	Incidence	Male	2019	71.02469	74.69937	67.37538	
Russian Federa	Incidence	Male	2020	70.27713	74.27394	66.39062	
Russian Federa	Incidence	Male	2021	67.86867	71.74474	64.08421	
Russian Federa	Incidence	Male	2022	64.10638	67.96264	60.22293	

Russian Federa	Incidence	Male	2023	67.635	72.82378	62.32299	
Russian Federa	Incidence	Female	2010	13.39661	15.10347	11.49601	
Russian Federa	Incidence	Female	2011	13.35885	15.07072	11.35381	
Russian Federa	Incidence	Female	2012	13.66264	15.42211	11.56388	
Russian Federa	Incidence	Female	2013	13.97348	15.81022	11.88235	
Russian Federa	Incidence	Female	2014	14.27259	16.03135	12.27514	
Russian Federa	Incidence	Female	2015	15.00555	16.82995	12.97226	
Russian Federa	Incidence	Female	2016	15.11216	16.97867	13.11964	
Russian Federa	Incidence	Female	2017	15.41915	17.23807	13.59355	
Russian Federa	Incidence	Female	2018	15.81564	17.75349	14.03273	
Russian Federa	Incidence	Female	2019	15.70857	17.66357	13.8664	
Russian Federa	Incidence	Female	2020	15.87776	18.01032	13.94657	
Russian Federa	Incidence	Female	2021	15.65841	17.7482	13.65205	
Russian Federa	Incidence	Female	2022	14.51008	16.66911	12.49278	
Russian Federa	Incidence	Female	2023	14.96582	17.3817	12.8795	
Russian Federa	Deaths	Male	2010	68.49609	73.07597	64.38531	
Russian Federa	Deaths	Male	2011	66.5738	70.98462	62.62585	
Russian Federa	Deaths	Male	2012	65.80264	69.83323	61.59321	
Russian Federa	Deaths	Male	2013	65.33823	69.21554	61.2812	
Russian Federa	Deaths	Male	2014	65.15288	68.48555	61.46149	
Russian Federa	Deaths	Male	2015	67.50577	70.98223	63.78063	
Russian Federa	Deaths	Male	2016	67.21583	70.29863	64.15292	
Russian Federa	Deaths	Male	2017	64.97893	67.63374	61.94906	
Russian Federa	Deaths	Male	2018	65.44198	68.05827	62.68633	
Russian Federa	Deaths	Male	2019	63.87946	66.71421	60.89973	
Russian Federa	Deaths	Male	2020	63.19403	66.08514	60.19144	
Russian Federa	Deaths	Male	2021	61.00816	63.79548	58.06244	
Russian Federa	Deaths	Male	2022	57.56728	60.76929	54.33903	
Russian Federa	Deaths	Male	2023	60.90153	65.16585	56.87696	
Russian Federa	Deaths	Female	2010	12.56301	13.97136	10.83349	
Russian Federa	Deaths	Female	2011	12.45262	13.93688	10.68107	
Russian Federa	Deaths	Female	2012	12.69946	14.1873	10.86552	
Russian Federa	Deaths	Female	2013	12.94381	14.33863	11.05551	
Russian Federa	Deaths	Female	2014	13.2251	14.53222	11.45817	
Russian Federa	Deaths	Female	2015	13.8737	15.22812	11.96253	
Russian Federa	Deaths	Female	2016	13.87749	15.214	12.04097	

Russian Federa	Deaths	Female	2017	14.06159	15.43798	12.377	
Russian Federa	Deaths	Female	2018	14.33413	15.69256	12.85053	
Russian Federa	Deaths	Female	2019	14.17706	15.64736	12.71329	
Russian Federa	Deaths	Female	2020	14.29188	15.93369	12.78337	
Russian Federa	Deaths	Female	2021	14.05968	15.75956	12.46857	
Russian Federa	Deaths	Female	2022	12.98906	14.56342	11.41094	
Russian Federa	Deaths	Female	2023	13.38168	15.22966	11.57656	
Russian Federa	DALYs	Male	2010	1854.358	1984.021	1741.767	
Russian Federa	DALYs	Male	2011	1803.932	1934.272	1693.191	
Russian Federa	DALYs	Male	2012	1777.132	1887.432	1663.776	
Russian Federa	DALYs	Male	2013	1763.655	1865.941	1654.445	
Russian Federa	DALYs	Male	2014	1750.152	1838.82	1646.325	
Russian Federa	DALYs	Male	2015	1803.659	1893.543	1709.108	
Russian Federa	DALYs	Male	2016	1780.459	1863.903	1699.606	
Russian Federa	DALYs	Male	2017	1708.169	1777.879	1627.711	
Russian Federa	DALYs	Male	2018	1713.173	1780.828	1643.838	
Russian Federa	DALYs	Male	2019	1659.456	1729.22	1585.357	
Russian Federa	DALYs	Male	2020	1629.329	1707.992	1554.224	
Russian Federa	DALYs	Male	2021	1567.308	1640.648	1492	
Russian Federa	DALYs	Male	2022	1489.488	1570.575	1406.232	
Russian Federa	DALYs	Male	2023	1545.835	1654.877	1443.946	
Russian Federa	DALYs	Female	2010	296.4111	333.2783	255.968	
Russian Federa	DALYs	Female	2011	295.0617	331.4621	253.1495	
Russian Federa	DALYs	Female	2012	298.9862	334.9713	255.7915	
Russian Federa	DALYs	Female	2013	303.6724	337.6046	259.7318	
Russian Federa	DALYs	Female	2014	307.4437	338.0367	265.9457	
Russian Federa	DALYs	Female	2015	320.6831	353.4361	276.6661	
Russian Federa	DALYs	Female	2016	320.1841	350.7203	277.624	
Russian Federa	DALYs	Female	2017	322.5043	352.5702	284.1807	
Russian Federa	DALYs	Female	2018	327.3045	357.11	292.7142	
Russian Federa	DALYs	Female	2019	322.903	355.1438	289.5707	
Russian Federa	DALYs	Female	2020	324.2753	360.8199	290.6355	
Russian Federa	DALYs	Female	2021	318.2746	358.4	281.5566	
Russian Federa	DALYs	Female	2022	296.3528	334.9959	260.5945	
Russian Federa	DALYs	Female	2023	301.3426	344.605	260.9961	
Rwanda	Incidence	Male	2010	4.40985	6.557707	2.37965	

Rwanda	Incidence	Male	2011	4.568268	6.837547	2.483638	
Rwanda	Incidence	Male	2012	4.742402	7.028283	2.532047	
Rwanda	Incidence	Male	2013	4.949379	7.220727	2.537221	
Rwanda	Incidence	Male	2014	5.145244	7.63295	2.596827	
Rwanda	Incidence	Male	2015	5.312137	7.806543	2.631332	
Rwanda	Incidence	Male	2016	5.467602	8.1534	2.676078	
Rwanda	Incidence	Male	2017	5.648905	8.423596	2.797536	
Rwanda	Incidence	Male	2018	5.869386	8.845622	2.964008	
Rwanda	Incidence	Male	2019	6.170156	9.299879	3.109934	
Rwanda	Incidence	Male	2020	6.456276	9.624635	3.21237	
Rwanda	Incidence	Male	2021	7.193632	10.58875	3.624467	
Rwanda	Incidence	Male	2022	7.685748	11.26381	3.774544	
Rwanda	Incidence	Male	2023	8.230103	12.5043	4.014091	
Rwanda	Incidence	Female	2010	1.706919	2.580455	0.993387	
Rwanda	Incidence	Female	2011	1.773152	2.713847	1.02964	
Rwanda	Incidence	Female	2012	1.835692	2.797631	1.08245	
Rwanda	Incidence	Female	2013	1.926647	2.971894	1.133194	
Rwanda	Incidence	Female	2014	1.984895	3.0353	1.19545	
Rwanda	Incidence	Female	2015	2.040339	2.977567	1.229503	
Rwanda	Incidence	Female	2016	2.08627	2.999246	1.25999	
Rwanda	Incidence	Female	2017	2.167556	3.101191	1.300047	
Rwanda	Incidence	Female	2018	2.26641	3.220079	1.36432	
Rwanda	Incidence	Female	2019	2.399758	3.459381	1.465507	
Rwanda	Incidence	Female	2020	2.477733	3.558092	1.536144	
Rwanda	Incidence	Female	2021	2.622076	3.692451	1.612329	
Rwanda	Incidence	Female	2022	3.09831	4.485369	1.837864	
Rwanda	Incidence	Female	2023	3.377887	4.867012	1.98501	
Rwanda	Deaths	Male	2010	4.310249	6.397376	2.296728	
Rwanda	Deaths	Male	2011	4.462975	6.693827	2.405368	
Rwanda	Deaths	Male	2012	4.630178	6.896277	2.443117	
Rwanda	Deaths	Male	2013	4.827325	7.060475	2.446296	
Rwanda	Deaths	Male	2014	5.01328	7.411929	2.525973	
Rwanda	Deaths	Male	2015	5.171785	7.622908	2.55671	
Rwanda	Deaths	Male	2016	5.320218	7.999335	2.595421	
Rwanda	Deaths	Male	2017	5.493689	8.222653	2.682619	
Rwanda	Deaths	Male	2018	5.70362	8.612559	2.843338	

Rwanda	Deaths	Male	2019	5.992452	9.036321	2.985305	
Rwanda	Deaths	Male	2020	6.270831	9.394227	3.108708	
Rwanda	Deaths	Male	2021	6.99762	10.36171	3.514605	
Rwanda	Deaths	Male	2022	7.443076	10.95927	3.615513	
Rwanda	Deaths	Male	2023	7.95673	12.13701	3.851393	
Rwanda	Deaths	Female	2010	1.678841	2.572184	0.980973	
Rwanda	Deaths	Female	2011	1.745001	2.681285	1.009507	
Rwanda	Deaths	Female	2012	1.806654	2.775834	1.057419	
Rwanda	Deaths	Female	2013	1.895462	2.943964	1.115037	
Rwanda	Deaths	Female	2014	1.951723	2.976664	1.176189	
Rwanda	Deaths	Female	2015	2.005712	2.938964	1.205735	
Rwanda	Deaths	Female	2016	2.049971	2.931613	1.231557	
Rwanda	Deaths	Female	2017	2.12838	3.045023	1.272913	
Rwanda	Deaths	Female	2018	2.222329	3.164881	1.331792	
Rwanda	Deaths	Female	2019	2.350199	3.383606	1.428316	
Rwanda	Deaths	Female	2020	2.424133	3.470509	1.484537	
Rwanda	Deaths	Female	2021	2.560754	3.601905	1.558456	
Rwanda	Deaths	Female	2022	3.011244	4.365453	1.786294	
Rwanda	Deaths	Female	2023	3.270941	4.678953	1.91584	
Rwanda	DALYs	Male	2010	127.1431	190.2822	69.73928	
Rwanda	DALYs	Male	2011	131.5991	196.6979	72.49828	
Rwanda	DALYs	Male	2012	136.5825	199.1206	73.69574	
Rwanda	DALYs	Male	2013	142.6747	205.6041	74.87744	
Rwanda	DALYs	Male	2014	148.395	220.2193	76.35179	
Rwanda	DALYs	Male	2015	153.186	223.5584	78.54581	
Rwanda	DALYs	Male	2016	157.7392	232.7363	79.46408	
Rwanda	DALYs	Male	2017	163.026	241.3621	82.99927	
Rwanda	DALYs	Male	2018	169.5148	254.4897	87.81235	
Rwanda	DALYs	Male	2019	178.2379	271.7666	91.773	
Rwanda	DALYs	Male	2020	186.3022	274.3779	95.51414	
Rwanda	DALYs	Male	2021	206.5313	300.2547	106.4548	
Rwanda	DALYs	Male	2022	222.7073	325.8919	110.682	
Rwanda	DALYs	Male	2023	239.2754	359.2735	119.5661	
Rwanda	DALYs	Female	2010	49.21943	73.23157	28.80806	
Rwanda	DALYs	Female	2011	50.88612	77.49285	30.0238	
Rwanda	DALYs	Female	2012	52.51983	79.24614	31.70657	

Rwanda	DALYs	Female	2013	55.0199	83.71633	32.26531	
Rwanda	DALYs	Female	2014	56.62707	85.81487	34.26301	
Rwanda	DALYs	Female	2015	58.10586	84.94112	35.50099	
Rwanda	DALYs	Female	2016	59.36565	86.24763	36.02037	
Rwanda	DALYs	Female	2017	61.69619	88.42773	37.4745	
Rwanda	DALYs	Female	2018	64.65385	90.80542	39.40449	
Rwanda	DALYs	Female	2019	68.57243	98.27144	42.05751	
Rwanda	DALYs	Female	2020	70.88203	100.8967	45.14533	
Rwanda	DALYs	Female	2021	75.16793	105.2559	46.81593	
Rwanda	DALYs	Female	2022	89.64168	128.129	53.70244	
Rwanda	DALYs	Female	2023	98.50951	142.5168	58.98942	
Saint Lucia	Incidence	Male	2010	14.99404	17.76263	12.79877	
Saint Lucia	Incidence	Male	2011	15.00268	18.1721	12.79778	
Saint Lucia	Incidence	Male	2012	15.22131	18.69173	13.04573	
Saint Lucia	Incidence	Male	2013	15.75611	19.14296	13.23501	
Saint Lucia	Incidence	Male	2014	15.46821	18.87451	12.86004	
Saint Lucia	Incidence	Male	2015	15.95438	19.16837	13.44332	
Saint Lucia	Incidence	Male	2016	15.59998	18.67525	13.23073	
Saint Lucia	Incidence	Male	2017	15.45845	18.60089	13.20932	
Saint Lucia	Incidence	Male	2018	15.4096	18.26956	13.13734	
Saint Lucia	Incidence	Male	2019	14.67129	17.54933	12.65823	
Saint Lucia	Incidence	Male	2020	15.38025	18.27068	13.17459	
Saint Lucia	Incidence	Male	2021	16.07243	19.52248	13.5574	
Saint Lucia	Incidence	Male	2022	15.79216	18.99298	13.36153	
Saint Lucia	Incidence	Male	2023	16.12875	19.53842	13.13129	
Saint Lucia	Incidence	Female	2010	6.837293	7.997297	5.772214	
Saint Lucia	Incidence	Female	2011	7.107865	8.346653	5.924883	
Saint Lucia	Incidence	Female	2012	7.285862	8.59913	6.046369	
Saint Lucia	Incidence	Female	2013	7.352456	8.571404	6.160628	
Saint Lucia	Incidence	Female	2014	7.376388	8.656505	6.118255	
Saint Lucia	Incidence	Female	2015	7.604284	9.101549	6.329783	
Saint Lucia	Incidence	Female	2016	7.659195	9.063096	6.318125	
Saint Lucia	Incidence	Female	2017	7.474501	8.743258	6.255453	
Saint Lucia	Incidence	Female	2018	7.794341	9.133769	6.491381	
Saint Lucia	Incidence	Female	2019	7.988065	9.369812	6.679342	
Saint Lucia	Incidence	Female	2020	8.256118	9.818863	6.750726	

Saint Lucia	Incidence	Female	2021	8.904503	10.47509	7.217014	
Saint Lucia	Incidence	Female	2022	9.20989	11.05129	7.45451	
Saint Lucia	Incidence	Female	2023	10.03686	11.96323	8.245868	
Saint Lucia	Deaths	Male	2010	14.77987	17.47502	12.59942	
Saint Lucia	Deaths	Male	2011	14.6122	17.59154	12.51964	
Saint Lucia	Deaths	Male	2012	14.81757	17.94671	12.72213	
Saint Lucia	Deaths	Male	2013	15.26337	18.55567	12.84021	
Saint Lucia	Deaths	Male	2014	15.07854	18.36592	12.58703	
Saint Lucia	Deaths	Male	2015	15.45874	18.6736	13.05277	
Saint Lucia	Deaths	Male	2016	15.03206	18.04274	12.82945	
Saint Lucia	Deaths	Male	2017	14.91623	17.89734	12.77198	
Saint Lucia	Deaths	Male	2018	14.90791	17.74733	12.63562	
Saint Lucia	Deaths	Male	2019	14.1839	16.72561	12.33692	
Saint Lucia	Deaths	Male	2020	14.79245	17.49827	12.74743	
Saint Lucia	Deaths	Male	2021	15.41945	18.6149	13.07019	
Saint Lucia	Deaths	Male	2022	15.22553	18.32066	12.88323	
Saint Lucia	Deaths	Male	2023	15.56965	18.80472	12.71233	
Saint Lucia	Deaths	Female	2010	6.670227	7.71232	5.610603	
Saint Lucia	Deaths	Female	2011	6.899117	8.110343	5.746048	
Saint Lucia	Deaths	Female	2012	7.093705	8.32516	5.924868	
Saint Lucia	Deaths	Female	2013	7.156356	8.346508	5.937505	
Saint Lucia	Deaths	Female	2014	7.205324	8.42859	6.0055	
Saint Lucia	Deaths	Female	2015	7.394361	8.803062	6.162805	
Saint Lucia	Deaths	Female	2016	7.415284	8.781614	6.15137	
Saint Lucia	Deaths	Female	2017	7.201091	8.480436	6.046152	
Saint Lucia	Deaths	Female	2018	7.518141	8.744496	6.230295	
Saint Lucia	Deaths	Female	2019	7.697697	8.982986	6.460435	
Saint Lucia	Deaths	Female	2020	7.942428	9.35316	6.543778	
Saint Lucia	Deaths	Female	2021	8.525522	10.05853	6.946045	
Saint Lucia	Deaths	Female	2022	8.826527	10.55619	7.245898	
Saint Lucia	Deaths	Female	2023	9.607987	11.44886	7.869562	
Saint Lucia	DALYs	Male	2010	372.9436	443.7586	317.6523	
Saint Lucia	DALYs	Male	2011	378.294	456.0199	321.7681	
Saint Lucia	DALYs	Male	2012	385.3649	471.8188	327.9603	
Saint Lucia	DALYs	Male	2013	401.2028	492.2596	337.3229	
Saint Lucia	DALYs	Male	2014	393.331	482.2335	325.4186	

Saint Lucia	DALYs	Male	2015	407.0961	489.7686	340.6855	
Saint Lucia	DALYs	Male	2016	399.9255	479.1008	338.1088	
Saint Lucia	DALYs	Male	2017	395.1657	476.8269	335.9921	
Saint Lucia	DALYs	Male	2018	392.5854	468.4186	332.7348	
Saint Lucia	DALYs	Male	2019	373.3325	446.579	320.3083	
Saint Lucia	DALYs	Male	2020	394.6605	470.6041	336.635	
Saint Lucia	DALYs	Male	2021	414.4581	501.1147	347.9141	
Saint Lucia	DALYs	Male	2022	399.9522	484.9668	334.8625	
Saint Lucia	DALYs	Male	2023	404.6054	491.2546	325.6203	
Saint Lucia	DALYs	Female	2010	163.9065	191.5479	137.1915	
Saint Lucia	DALYs	Female	2011	170.7478	200.4921	141.7706	
Saint Lucia	DALYs	Female	2012	173.8545	205.7071	144.6357	
Saint Lucia	DALYs	Female	2013	176.2095	205.7756	146.8771	
Saint Lucia	DALYs	Female	2014	176.0892	205.9871	145.3782	
Saint Lucia	DALYs	Female	2015	182.1052	214.7616	152.3948	
Saint Lucia	DALYs	Female	2016	184.2663	218.0644	152.199	
Saint Lucia	DALYs	Female	2017	180.5832	213.5111	151.0259	
Saint Lucia	DALYs	Female	2018	186.7281	218.5777	155.3111	
Saint Lucia	DALYs	Female	2019	191.0056	223.2452	161.7755	
Saint Lucia	DALYs	Female	2020	197.4434	234.3401	162.3515	
Saint Lucia	DALYs	Female	2021	214.4684	252.0924	172.8434	
Saint Lucia	DALYs	Female	2022	217.8809	260.8268	176.3882	
Saint Lucia	DALYs	Female	2023	235.5962	281.6981	189.978	
Samoa	Incidence	Male	2010	7.374642	11.2365	3.667514	
Samoa	Incidence	Male	2011	7.397	10.90055	3.721548	
Samoa	Incidence	Male	2012	7.43285	11.36961	3.696883	
Samoa	Incidence	Male	2013	7.502512	11.86302	3.669981	
Samoa	Incidence	Male	2014	7.582817	11.90298	3.708087	
Samoa	Incidence	Male	2015	8.057764	12.19097	3.886551	
Samoa	Incidence	Male	2016	8.293995	12.38182	4.004614	
Samoa	Incidence	Male	2017	8.565943	12.74933	4.144073	
Samoa	Incidence	Male	2018	8.887513	13.51703	4.249334	
Samoa	Incidence	Male	2019	9.454531	14.92434	4.512835	
Samoa	Incidence	Male	2020	9.731971	14.88253	4.675236	
Samoa	Incidence	Male	2021	11.04583	16.3085	5.519202	
Samoa	Incidence	Male	2022	11.28023	17.08385	5.391125	

Samoa	Incidence	Male	2023	12.10891	18.54515	5.456285	
Samoa	Incidence	Female	2010	1.181196	1.718117	0.770724	
Samoa	Incidence	Female	2011	1.189619	1.716616	0.775107	
Samoa	Incidence	Female	2012	1.195403	1.712225	0.808349	
Samoa	Incidence	Female	2013	1.205474	1.749277	0.821912	
Samoa	Incidence	Female	2014	1.216357	1.707944	0.801048	
Samoa	Incidence	Female	2015	1.248665	1.798757	0.809998	
Samoa	Incidence	Female	2016	1.278997	1.858206	0.843911	
Samoa	Incidence	Female	2017	1.312426	1.866482	0.884293	
Samoa	Incidence	Female	2018	1.349378	1.874484	0.926859	
Samoa	Incidence	Female	2019	1.439095	1.964086	1.010202	
Samoa	Incidence	Female	2020	1.429065	1.965757	1.002337	
Samoa	Incidence	Female	2021	1.541487	2.12716	1.063717	
Samoa	Incidence	Female	2022	1.628257	2.233762	1.125045	
Samoa	Incidence	Female	2023	1.762059	2.447813	1.197647	
Samoa	Deaths	Male	2010	7.320776	11.14356	3.667546	
Samoa	Deaths	Male	2011	7.338789	10.80353	3.722304	
Samoa	Deaths	Male	2012	7.371251	11.2665	3.694709	
Samoa	Deaths	Male	2013	7.438226	11.74649	3.647503	
Samoa	Deaths	Male	2014	7.514618	11.78316	3.698375	
Samoa	Deaths	Male	2015	7.981823	12.04867	3.87934	
Samoa	Deaths	Male	2016	8.209305	12.25681	3.992595	
Samoa	Deaths	Male	2017	8.468519	12.61704	4.118478	
Samoa	Deaths	Male	2018	8.77826	13.36879	4.222341	
Samoa	Deaths	Male	2019	9.331998	14.72191	4.488269	
Samoa	Deaths	Male	2020	9.597016	14.66279	4.643279	
Samoa	Deaths	Male	2021	10.86623	16.00628	5.457157	
Samoa	Deaths	Male	2022	11.0996	16.82099	5.339732	
Samoa	Deaths	Male	2023	11.90644	18.19534	5.421746	
Samoa	Deaths	Female	2010	1.168595	1.708963	0.75446	
Samoa	Deaths	Female	2011	1.177201	1.703802	0.760305	
Samoa	Deaths	Female	2012	1.182999	1.693411	0.798203	
Samoa	Deaths	Female	2013	1.192818	1.732649	0.808851	
Samoa	Deaths	Female	2014	1.203201	1.695061	0.789803	
Samoa	Deaths	Female	2015	1.234426	1.776745	0.790922	
Samoa	Deaths	Female	2016	1.262826	1.834111	0.832341	

Samoa	Deaths	Female	2017	1.293855	1.810789	0.862635	
Samoa	Deaths	Female	2018	1.328501	1.833388	0.909475	
Samoa	Deaths	Female	2019	1.414995	1.937571	0.996167	
Samoa	Deaths	Female	2020	1.402517	1.914169	0.988233	
Samoa	Deaths	Female	2021	1.507614	2.063822	1.045836	
Samoa	Deaths	Female	2022	1.591611	2.169392	1.101257	
Samoa	Deaths	Female	2023	1.72102	2.402014	1.170116	
Samoa	DALYs	Male	2010	199.5625	302.5621	99.28389	
Samoa	DALYs	Male	2011	200.5348	298.2959	100.714	
Samoa	DALYs	Male	2012	201.6958	308.6532	100.0527	
Samoa	DALYs	Male	2013	203.6103	322.4268	99.13143	
Samoa	DALYs	Male	2014	205.8786	325.9894	100.559	
Samoa	DALYs	Male	2015	218.7274	329.6306	105.4669	
Samoa	DALYs	Male	2016	225.0763	335.3054	108.7035	
Samoa	DALYs	Male	2017	232.4986	345.1138	112.6754	
Samoa	DALYs	Male	2018	241.1849	366.6371	116.6026	
Samoa	DALYs	Male	2019	256.2848	404.2553	124.0172	
Samoa	DALYs	Male	2020	263.5205	400.9467	128.382	
Samoa	DALYs	Male	2021	298.8997	437.6784	148.076	
Samoa	DALYs	Male	2022	303.9233	458.3087	143.9947	
Samoa	DALYs	Male	2023	325.3922	497.6505	148.5317	
Samoa	DALYs	Female	2010	31.82715	46.27578	20.74109	
Samoa	DALYs	Female	2011	32.06808	45.78111	20.98339	
Samoa	DALYs	Female	2012	32.24114	45.86468	21.93672	
Samoa	DALYs	Female	2013	32.53465	47.18229	22.33236	
Samoa	DALYs	Female	2014	32.86651	46.14476	21.72078	
Samoa	DALYs	Female	2015	33.75782	48.90947	21.95855	
Samoa	DALYs	Female	2016	34.59447	50.00393	22.78315	
Samoa	DALYs	Female	2017	35.52752	50.30838	23.82963	
Samoa	DALYs	Female	2018	36.55171	51.038	25.1283	
Samoa	DALYs	Female	2019	38.96624	53.27261	27.16184	
Samoa	DALYs	Female	2020	38.72152	52.97277	26.84641	
Samoa	DALYs	Female	2021	41.79305	57.82351	28.29822	
Samoa	DALYs	Female	2022	43.85702	60.56994	30.24366	
Samoa	DALYs	Female	2023	47.26038	65.61688	32.35249	
Sao Tome and I	Incidence	Male	2010	10.49165	15.13868	7.214144	

Sao Tome and I	Incidence	Male	2011	10.51315	15.03334	7.373393	
Sao Tome and I	Incidence	Male	2012	10.32931	14.7892	7.453609	
Sao Tome and I	Incidence	Male	2013	10.56564	15.52889	7.610582	
Sao Tome and I	Incidence	Male	2014	10.87336	16.35676	7.625809	
Sao Tome and I	Incidence	Male	2015	11.32918	17.11984	7.87776	
Sao Tome and I	Incidence	Male	2016	11.66206	17.51417	8.087422	
Sao Tome and I	Incidence	Male	2017	11.82493	17.29181	8.134233	
Sao Tome and I	Incidence	Male	2018	11.99061	17.0858	8.248429	
Sao Tome and I	Incidence	Male	2019	12.36776	17.68212	8.247537	
Sao Tome and I	Incidence	Male	2020	14.51344	21.63315	8.624604	
Sao Tome and I	Incidence	Male	2021	14.57728	22.19579	8.819475	
Sao Tome and I	Incidence	Male	2022	14.12232	21.75097	8.991738	
Sao Tome and I	Incidence	Male	2023	14.25611	21.49749	9.878716	
Sao Tome and I	Incidence	Female	2010	3.085866	4.48049	2.07763	
Sao Tome and I	Incidence	Female	2011	3.117728	4.495078	2.034158	
Sao Tome and I	Incidence	Female	2012	3.107636	4.516062	2.066344	
Sao Tome and I	Incidence	Female	2013	3.230963	4.766213	2.049762	
Sao Tome and I	Incidence	Female	2014	3.478557	5.163648	2.157877	
Sao Tome and I	Incidence	Female	2015	3.630056	5.33939	2.283926	
Sao Tome and I	Incidence	Female	2016	3.78064	5.487472	2.419835	
Sao Tome and I	Incidence	Female	2017	3.942948	5.684289	2.518355	
Sao Tome and I	Incidence	Female	2018	4.05965	5.951037	2.613762	
Sao Tome and I	Incidence	Female	2019	4.189671	6.086579	2.705861	
Sao Tome and I	Incidence	Female	2020	4.602028	7.272859	2.884973	
Sao Tome and I	Incidence	Female	2021	4.681648	7.348161	2.81602	
Sao Tome and I	Incidence	Female	2022	4.702042	7.095215	2.931464	
Sao Tome and I	Incidence	Female	2023	4.878732	7.239392	3.1342	
Sao Tome and I	Deaths	Male	2010	10.82673	15.50268	7.508108	
Sao Tome and I	Deaths	Male	2011	10.81771	15.2317	7.638368	
Sao Tome and I	Deaths	Male	2012	10.60687	15.41309	7.636424	
Sao Tome and I	Deaths	Male	2013	10.82847	15.86768	7.810823	
Sao Tome and I	Deaths	Male	2014	11.11321	16.41029	7.794968	
Sao Tome and I	Deaths	Male	2015	11.53244	17.33741	8.016665	
Sao Tome and I	Deaths	Male	2016	11.82372	17.68719	8.191092	
Sao Tome and I	Deaths	Male	2017	11.95126	17.41391	8.231197	
Sao Tome and I	Deaths	Male	2018	12.08835	17.36002	8.391623	

Sao Tome and I	Deaths	Male	2019	12.44388	17.507	8.365365	
Sao Tome and I	Deaths	Male	2020	14.6763	22.02665	8.895676	
Sao Tome and I	Deaths	Male	2021	14.65742	22.07126	8.777341	
Sao Tome and I	Deaths	Male	2022	14.16922	22.00992	9.00688	
Sao Tome and I	Deaths	Male	2023	14.25404	20.96054	9.903578	
Sao Tome and I	Deaths	Female	2010	3.119632	4.49776	2.104978	
Sao Tome and I	Deaths	Female	2011	3.141619	4.529365	2.041608	
Sao Tome and I	Deaths	Female	2012	3.124639	4.507359	2.065777	
Sao Tome and I	Deaths	Female	2013	3.237958	4.756483	2.053655	
Sao Tome and I	Deaths	Female	2014	3.469883	5.049371	2.152151	
Sao Tome and I	Deaths	Female	2015	3.604996	5.229765	2.26326	
Sao Tome and I	Deaths	Female	2016	3.73883	5.390724	2.384602	
Sao Tome and I	Deaths	Female	2017	3.884456	5.50815	2.48919	
Sao Tome and I	Deaths	Female	2018	3.986115	5.765372	2.555615	
Sao Tome and I	Deaths	Female	2019	4.10285	5.83503	2.657321	
Sao Tome and I	Deaths	Female	2020	4.50595	6.998026	2.857114	
Sao Tome and I	Deaths	Female	2021	4.558279	7.023048	2.733796	
Sao Tome and I	Deaths	Female	2022	4.587693	6.895676	2.880585	
Sao Tome and I	Deaths	Female	2023	4.74593	7.055221	3.059967	
Sao Tome and I	DALYs	Male	2010	268.1267	395.362	182.7814	
Sao Tome and I	DALYs	Male	2011	270.5585	398.8467	188.1474	
Sao Tome and I	DALYs	Male	2012	267.2135	398.1837	193.0766	
Sao Tome and I	DALYs	Male	2013	274.6372	417.6377	196.199	
Sao Tome and I	DALYs	Male	2014	284.3215	432.3302	199.4859	
Sao Tome and I	DALYs	Male	2015	298.9071	453.0395	209.0111	
Sao Tome and I	DALYs	Male	2016	310.1205	464.3369	213.9196	
Sao Tome and I	DALYs	Male	2017	315.9516	469.1784	217.5522	
Sao Tome and I	DALYs	Male	2018	321.3308	465.1451	219.7394	
Sao Tome and I	DALYs	Male	2019	332.2774	488.6671	218.7475	
Sao Tome and I	DALYs	Male	2020	386.7455	590.3211	236.2436	
Sao Tome and I	DALYs	Male	2021	391.4945	596.8861	232.8379	
Sao Tome and I	DALYs	Male	2022	378.5233	571.824	242.3401	
Sao Tome and I	DALYs	Male	2023	382.6935	575.9464	262.3981	
Sao Tome and I	DALYs	Female	2010	85.45033	123.7042	57.06573	
Sao Tome and I	DALYs	Female	2011	86.77504	126.3501	56.19103	
Sao Tome and I	DALYs	Female	2012	86.72867	126.0652	57.43249	

Sao Tome and I	DALYs	Female	2013	90.78644	135.3565	57.67906	
Sao Tome and I	DALYs	Female	2014	98.78325	147.2631	61.11275	
Sao Tome and I	DALYs	Female	2015	103.9462	157.8042	64.88134	
Sao Tome and I	DALYs	Female	2016	108.971	159.5932	69.31856	
Sao Tome and I	DALYs	Female	2017	114.265	165.1038	71.4167	
Sao Tome and I	DALYs	Female	2018	118.0772	174.1796	75.03494	
Sao Tome and I	DALYs	Female	2019	122.2397	181.7211	77.78435	
Sao Tome and I	DALYs	Female	2020	132.9681	211.1419	81.4473	
Sao Tome and I	DALYs	Female	2021	135.5816	219.3468	82.02504	
Sao Tome and I	DALYs	Female	2022	136.2142	212	83.4777	
Sao Tome and I	DALYs	Female	2023	141.8642	210.9895	91.07517	
Saudi Arabia	Incidence	Male	2010	8.358393	10.7532	6.687495	
Saudi Arabia	Incidence	Male	2011	8.054903	11.0702	6.419868	
Saudi Arabia	Incidence	Male	2012	7.675501	11.05636	5.996535	
Saudi Arabia	Incidence	Male	2013	7.114936	10.81981	5.352463	
Saudi Arabia	Incidence	Male	2014	6.489076	10.25616	4.952983	
Saudi Arabia	Incidence	Male	2015	6.143905	10.01006	4.57281	
Saudi Arabia	Incidence	Male	2016	5.91635	9.619242	4.408808	
Saudi Arabia	Incidence	Male	2017	5.921296	9.54125	4.517505	
Saudi Arabia	Incidence	Male	2018	5.975787	9.744058	4.645458	
Saudi Arabia	Incidence	Male	2019	6.115875	9.958784	4.754621	
Saudi Arabia	Incidence	Male	2020	6.292436	10.0264	4.874937	
Saudi Arabia	Incidence	Male	2021	6.389363	10.36226	4.941787	
Saudi Arabia	Incidence	Male	2022	6.391413	10.18502	4.965851	
Saudi Arabia	Incidence	Male	2023	6.599712	10.30436	5.041873	
Saudi Arabia	Incidence	Female	2010	2.637839	3.56967	1.989976	
Saudi Arabia	Incidence	Female	2011	2.640398	3.539214	1.980229	
Saudi Arabia	Incidence	Female	2012	2.618978	3.624239	1.905065	
Saudi Arabia	Incidence	Female	2013	2.561367	3.600066	1.86049	
Saudi Arabia	Incidence	Female	2014	2.527236	3.615279	1.846598	
Saudi Arabia	Incidence	Female	2015	2.483611	3.704313	1.786964	
Saudi Arabia	Incidence	Female	2016	2.49606	3.677718	1.793837	
Saudi Arabia	Incidence	Female	2017	2.586958	3.831568	1.855829	
Saudi Arabia	Incidence	Female	2018	2.66543	4.020292	1.942128	
Saudi Arabia	Incidence	Female	2019	2.830232	4.178862	2.139097	
Saudi Arabia	Incidence	Female	2020	3.106325	4.546762	2.369863	

Saudi Arabia	Incidence	Female	2021	3.351145	4.996532	2.546337	
Saudi Arabia	Incidence	Female	2022	3.472766	4.976969	2.6159	
Saudi Arabia	Incidence	Female	2023	3.462151	5.026299	2.467545	
Saudi Arabia	Deaths	Male	2010	7.822998	9.971868	6.286964	
Saudi Arabia	Deaths	Male	2011	7.523711	10.22956	5.991055	
Saudi Arabia	Deaths	Male	2012	7.162626	10.19682	5.638542	
Saudi Arabia	Deaths	Male	2013	6.644803	9.995358	5.063478	
Saudi Arabia	Deaths	Male	2014	6.074101	9.452693	4.611931	
Saudi Arabia	Deaths	Male	2015	5.762182	9.285799	4.288213	
Saudi Arabia	Deaths	Male	2016	5.545374	8.830445	4.108434	
Saudi Arabia	Deaths	Male	2017	5.545813	8.753846	4.258527	
Saudi Arabia	Deaths	Male	2018	5.58873	8.902593	4.329669	
Saudi Arabia	Deaths	Male	2019	5.7057	9.088375	4.409985	
Saudi Arabia	Deaths	Male	2020	5.881426	9.197475	4.561274	
Saudi Arabia	Deaths	Male	2021	5.928185	9.406958	4.585539	
Saudi Arabia	Deaths	Male	2022	5.91359	9.412398	4.594857	
Saudi Arabia	Deaths	Male	2023	6.078497	9.458662	4.648505	
Saudi Arabia	Deaths	Female	2010	2.483284	3.342621	1.882941	
Saudi Arabia	Deaths	Female	2011	2.479336	3.309123	1.881599	
Saudi Arabia	Deaths	Female	2012	2.454145	3.329578	1.796156	
Saudi Arabia	Deaths	Female	2013	2.398397	3.298344	1.76382	
Saudi Arabia	Deaths	Female	2014	2.365995	3.282496	1.734687	
Saudi Arabia	Deaths	Female	2015	2.320813	3.306118	1.673099	
Saudi Arabia	Deaths	Female	2016	2.325399	3.315406	1.6715	
Saudi Arabia	Deaths	Female	2017	2.400856	3.459738	1.739894	
Saudi Arabia	Deaths	Female	2018	2.465324	3.618974	1.807326	
Saudi Arabia	Deaths	Female	2019	2.607756	3.787592	1.97417	
Saudi Arabia	Deaths	Female	2020	2.86077	4.14059	2.185759	
Saudi Arabia	Deaths	Female	2021	3.063746	4.506557	2.337819	
Saudi Arabia	Deaths	Female	2022	3.165462	4.523492	2.397298	
Saudi Arabia	Deaths	Female	2023	3.143221	4.620532	2.282409	
Saudi Arabia	DALYs	Male	2010	246.7895	323.1297	197.1284	
Saudi Arabia	DALYs	Male	2011	236.902	329.9307	187.775	
Saudi Arabia	DALYs	Male	2012	224.422	328.5895	172.4244	
Saudi Arabia	DALYs	Male	2013	206.2982	318.163	155.7787	
Saudi Arabia	DALYs	Male	2014	185.9369	300.5351	141.991	

Saudi Arabia	DALYs	Male	2015	174.274	292.8216	131.5745	
Saudi Arabia	DALYs	Male	2016	166.2068	277.8473	127.0497	
Saudi Arabia	DALYs	Male	2017	164.6681	273.2088	125.5198	
Saudi Arabia	DALYs	Male	2018	164.7304	273.5952	129.1708	
Saudi Arabia	DALYs	Male	2019	167.2512	277.439	130.9482	
Saudi Arabia	DALYs	Male	2020	169.558	278.2598	132.7407	
Saudi Arabia	DALYs	Male	2021	172.8184	284.1612	134.8853	
Saudi Arabia	DALYs	Male	2022	172.8681	279.9918	134.6018	
Saudi Arabia	DALYs	Male	2023	179.8585	285.797	136.9567	
Saudi Arabia	DALYs	Female	2010	75.1969	102.2896	55.9777	
Saudi Arabia	DALYs	Female	2011	74.7917	102.7366	55.62588	
Saudi Arabia	DALYs	Female	2012	73.57835	104.9975	53.30927	
Saudi Arabia	DALYs	Female	2013	71.23376	102.9295	51.38201	
Saudi Arabia	DALYs	Female	2014	69.50077	102.2915	49.37737	
Saudi Arabia	DALYs	Female	2015	67.73362	102.1711	48.43856	
Saudi Arabia	DALYs	Female	2016	67.50228	101.9256	47.74347	
Saudi Arabia	DALYs	Female	2017	69.45394	106.1419	49.38402	
Saudi Arabia	DALYs	Female	2018	71.05232	109.8329	51.60022	
Saudi Arabia	DALYs	Female	2019	74.92639	113.3687	55.99314	
Saudi Arabia	DALYs	Female	2020	81.0026	121.094	61.66616	
Saudi Arabia	DALYs	Female	2021	87.65662	132.0518	67.0336	
Saudi Arabia	DALYs	Female	2022	90.7463	130.993	68.49498	
Saudi Arabia	DALYs	Female	2023	90.76934	132.8027	65.06846	
Senegal	Incidence	Male	2010	2.711615	3.856564	1.961161	
Senegal	Incidence	Male	2011	2.736467	3.942735	1.997789	
Senegal	Incidence	Male	2012	2.715044	3.918537	1.991711	
Senegal	Incidence	Male	2013	2.574974	3.670482	1.850128	
Senegal	Incidence	Male	2014	2.669512	3.805465	1.891315	
Senegal	Incidence	Male	2015	2.660445	3.763288	1.874464	
Senegal	Incidence	Male	2016	2.684906	3.821766	1.841252	
Senegal	Incidence	Male	2017	2.701259	3.869182	1.857293	
Senegal	Incidence	Male	2018	2.552221	3.690642	1.737568	
Senegal	Incidence	Male	2019	2.724026	3.990725	1.845811	
Senegal	Incidence	Male	2020	2.704482	3.920588	1.850116	
Senegal	Incidence	Male	2021	2.803679	4.150371	1.890202	
Senegal	Incidence	Male	2022	3.028879	4.541948	2.044779	

Senegal	Incidence	Male	2023	3.138068	4.56603	2.136105	
Senegal	Incidence	Female	2010	0.495368	0.706806	0.3347	
Senegal	Incidence	Female	2011	0.514073	0.73177	0.346244	
Senegal	Incidence	Female	2012	0.499932	0.723848	0.334126	
Senegal	Incidence	Female	2013	0.506843	0.735349	0.343309	
Senegal	Incidence	Female	2014	0.537507	0.744731	0.364058	
Senegal	Incidence	Female	2015	0.527074	0.718588	0.354691	
Senegal	Incidence	Female	2016	0.562297	0.759935	0.374784	
Senegal	Incidence	Female	2017	0.572759	0.783004	0.389322	
Senegal	Incidence	Female	2018	0.562121	0.74937	0.39993	
Senegal	Incidence	Female	2019	0.628134	0.821718	0.434256	
Senegal	Incidence	Female	2020	0.596525	0.775684	0.425914	
Senegal	Incidence	Female	2021	0.593471	0.771713	0.40051	
Senegal	Incidence	Female	2022	0.765839	1.002107	0.51577	
Senegal	Incidence	Female	2023	0.827556	1.115032	0.56664	
Senegal	Deaths	Male	2010	2.784621	3.929673	2.017461	
Senegal	Deaths	Male	2011	2.813244	3.980268	2.055973	
Senegal	Deaths	Male	2012	2.793659	3.99046	2.058146	
Senegal	Deaths	Male	2013	2.649038	3.718082	1.903096	
Senegal	Deaths	Male	2014	2.74331	3.842945	1.947984	
Senegal	Deaths	Male	2015	2.736929	3.822364	1.928765	
Senegal	Deaths	Male	2016	2.76098	3.863774	1.90049	
Senegal	Deaths	Male	2017	2.777034	3.951087	1.915614	
Senegal	Deaths	Male	2018	2.622078	3.752389	1.790583	
Senegal	Deaths	Male	2019	2.794701	4.02644	1.891767	
Senegal	Deaths	Male	2020	2.797213	4.06257	1.900294	
Senegal	Deaths	Male	2021	2.918417	4.239561	1.959115	
Senegal	Deaths	Male	2022	3.101705	4.608564	2.091857	
Senegal	Deaths	Male	2023	3.206094	4.605078	2.188263	
Senegal	Deaths	Female	2010	0.494386	0.70659	0.334841	
Senegal	Deaths	Female	2011	0.514271	0.73246	0.346084	
Senegal	Deaths	Female	2012	0.501101	0.730514	0.334662	
Senegal	Deaths	Female	2013	0.508466	0.737356	0.343335	
Senegal	Deaths	Female	2014	0.538414	0.748366	0.36334	
Senegal	Deaths	Female	2015	0.528039	0.722944	0.356819	
Senegal	Deaths	Female	2016	0.562294	0.762352	0.375179	

Senegal	Deaths	Female	2017	0.571095	0.78464	0.387867	
Senegal	Deaths	Female	2018	0.559032	0.746557	0.397678	
Senegal	Deaths	Female	2019	0.623092	0.810147	0.430101	
Senegal	Deaths	Female	2020	0.591913	0.77477	0.426897	
Senegal	Deaths	Female	2021	0.587502	0.768897	0.396807	
Senegal	Deaths	Female	2022	0.753232	0.990171	0.50784	
Senegal	Deaths	Female	2023	0.811506	1.092659	0.562246	
Senegal	DALYs	Male	2010	72.49052	103.4792	51.94545	
Senegal	DALYs	Male	2011	73.02736	106.3357	53.29373	
Senegal	DALYs	Male	2012	72.27594	105.9449	52.85504	
Senegal	DALYs	Male	2013	68.41177	98.58178	48.87278	
Senegal	DALYs	Male	2014	71.07437	102.9057	50.20228	
Senegal	DALYs	Male	2015	70.86892	100.4982	50.15737	
Senegal	DALYs	Male	2016	71.59432	103.7575	48.59863	
Senegal	DALYs	Male	2017	72.08599	103.4785	49.21484	
Senegal	DALYs	Male	2018	68.17743	99.39496	46.56438	
Senegal	DALYs	Male	2019	72.88142	108.3904	48.82474	
Senegal	DALYs	Male	2020	71.8527	106.104	49.11712	
Senegal	DALYs	Male	2021	73.84169	109.3007	50.50731	
Senegal	DALYs	Male	2022	81.25932	121.285	54.85262	
Senegal	DALYs	Male	2023	84.26593	125.6345	57.73054	
Senegal	DALYs	Female	2010	13.94642	19.90708	9.492658	
Senegal	DALYs	Female	2011	14.38182	20.39333	9.800737	
Senegal	DALYs	Female	2012	13.88975	19.7923	9.34958	
Senegal	DALYs	Female	2013	13.97654	20.14595	9.389489	
Senegal	DALYs	Female	2014	14.84413	20.68573	10.07604	
Senegal	DALYs	Female	2015	14.58733	19.85947	9.748011	
Senegal	DALYs	Female	2016	15.6184	20.87258	10.39911	
Senegal	DALYs	Female	2017	15.99635	21.79358	10.89044	
Senegal	DALYs	Female	2018	15.75312	20.82701	11.12328	
Senegal	DALYs	Female	2019	17.67608	23.26481	12.31594	
Senegal	DALYs	Female	2020	16.81838	21.98185	12.02299	
Senegal	DALYs	Female	2021	16.80811	21.51404	11.40726	
Senegal	DALYs	Female	2022	21.86618	28.50718	14.82033	
Senegal	DALYs	Female	2023	23.71684	31.80127	15.9884	
Serbia	Incidence	Male	2010	121.6266	139.2584	94.0968	

Serbia	Incidence	Male	2011	122.7138	139.6082	94.21504	
Serbia	Incidence	Male	2012	122.2113	139.3354	93.05127	
Serbia	Incidence	Male	2013	121.5536	138.8132	92.21491	
Serbia	Incidence	Male	2014	121.2117	138.6778	91.23597	
Serbia	Incidence	Male	2015	120.7815	138.5507	90.08524	
Serbia	Incidence	Male	2016	119.3283	136.1217	91.62245	
Serbia	Incidence	Male	2017	118.8613	135.0156	91.22543	
Serbia	Incidence	Male	2018	117.1478	132.6596	92.65188	
Serbia	Incidence	Male	2019	115.9281	131.118	91.53541	
Serbia	Incidence	Male	2020	113.3222	127.8117	89.86594	
Serbia	Incidence	Male	2021	110.9987	124.7075	88.78877	
Serbia	Incidence	Male	2022	108.5658	120.8619	88.0096	
Serbia	Incidence	Male	2023	109.969	131.8931	90.56368	
Serbia	Incidence	Female	2010	42.10669	49.92464	36.4513	
Serbia	Incidence	Female	2011	43.46383	51.2858	38.13099	
Serbia	Incidence	Female	2012	44.70614	52.80038	38.9079	
Serbia	Incidence	Female	2013	45.85607	53.481	39.81557	
Serbia	Incidence	Female	2014	47.26831	55.35398	40.59855	
Serbia	Incidence	Female	2015	48.94373	58.02188	41.73792	
Serbia	Incidence	Female	2016	49.46075	57.8947	42.12193	
Serbia	Incidence	Female	2017	51.09152	60.14403	43.09829	
Serbia	Incidence	Female	2018	51.09421	60.17586	42.44564	
Serbia	Incidence	Female	2019	51.35906	60.09384	41.86826	
Serbia	Incidence	Female	2020	51.73678	59.2372	41.74927	
Serbia	Incidence	Female	2021	51.775	58.82874	40.95344	
Serbia	Incidence	Female	2022	51.41065	59.33102	41.55877	
Serbia	Incidence	Female	2023	52.24267	65.78604	41.50091	
Serbia	Deaths	Male	2010	113.8573	130.7059	88.93042	
Serbia	Deaths	Male	2011	114.7626	130.935	88.67851	
Serbia	Deaths	Male	2012	114.2597	131.1338	87.42584	
Serbia	Deaths	Male	2013	113.606	130.1891	87.2259	
Serbia	Deaths	Male	2014	113.1763	128.8578	86.33832	
Serbia	Deaths	Male	2015	112.7202	127.5707	85.72665	
Serbia	Deaths	Male	2016	111.2385	124.5384	86.08226	
Serbia	Deaths	Male	2017	110.898	125.1152	84.79828	
Serbia	Deaths	Male	2018	109.2272	122.9547	85.90291	

Serbia	Deaths	Male	2019	107.9008	121.3407	84.77394	
Serbia	Deaths	Male	2020	105.7662	119.2444	83.59677	
Serbia	Deaths	Male	2021	103.794	117.3105	83.7829	
Serbia	Deaths	Male	2022	101.2988	112.7957	83.25728	
Serbia	Deaths	Male	2023	102.5308	122.5377	84.60005	
Serbia	Deaths	Female	2010	40.2217	47.83979	34.93933	
Serbia	Deaths	Female	2011	41.44297	49.94983	36.48044	
Serbia	Deaths	Female	2012	42.56611	50.2818	37.24615	
Serbia	Deaths	Female	2013	43.57229	51.1288	37.78157	
Serbia	Deaths	Female	2014	44.96046	52.52631	38.4289	
Serbia	Deaths	Female	2015	46.59843	54.67926	39.74015	
Serbia	Deaths	Female	2016	47.12441	54.82651	40.94142	
Serbia	Deaths	Female	2017	48.7607	57.44143	41.93161	
Serbia	Deaths	Female	2018	48.80506	57.69208	41.25551	
Serbia	Deaths	Female	2019	49.15343	57.25803	40.91176	
Serbia	Deaths	Female	2020	49.65666	56.82894	40.79395	
Serbia	Deaths	Female	2021	49.86254	56.01714	40.08424	
Serbia	Deaths	Female	2022	49.4602	56.53047	40.8134	
Serbia	Deaths	Female	2023	50.33158	63.22348	40.11644	
Serbia	DALYs	Male	2010	2982.822	3396.184	2285.615	
Serbia	DALYs	Male	2011	2984.338	3383.064	2253.979	
Serbia	DALYs	Male	2012	2952.798	3361.924	2220.262	
Serbia	DALYs	Male	2013	2924.143	3344.541	2218.456	
Serbia	DALYs	Male	2014	2890.431	3267.275	2207.094	
Serbia	DALYs	Male	2015	2854.509	3223.718	2178.545	
Serbia	DALYs	Male	2016	2804.743	3148.977	2175.188	
Serbia	DALYs	Male	2017	2769.973	3136.186	2118.921	
Serbia	DALYs	Male	2018	2713.378	3054.265	2133.994	
Serbia	DALYs	Male	2019	2676.263	3024.779	2105.516	
Serbia	DALYs	Male	2020	2589.88	2955.629	2048.237	
Serbia	DALYs	Male	2021	2516.23	2882.601	2015.343	
Serbia	DALYs	Male	2022	2460.178	2764.544	2008.419	
Serbia	DALYs	Male	2023	2476.949	3011.335	2036.079	
Serbia	DALYs	Female	2010	1026.719	1206.994	885.8837	
Serbia	DALYs	Female	2011	1052.539	1235.991	924.4467	
Serbia	DALYs	Female	2012	1078.97	1253.085	943.7171	

Serbia	DALYs	Female	2013	1103.158	1275.176	946.4133	
Serbia	DALYs	Female	2014	1129.275	1304.402	970.1713	
Serbia	DALYs	Female	2015	1162.726	1342.886	981.6704	
Serbia	DALYs	Female	2016	1171.744	1347.675	972.4129	
Serbia	DALYs	Female	2017	1204.383	1389.598	986.7891	
Serbia	DALYs	Female	2018	1201.796	1395.707	972.9511	
Serbia	DALYs	Female	2019	1201.73	1389.929	954.194	
Serbia	DALYs	Female	2020	1204.027	1365.599	944.1349	
Serbia	DALYs	Female	2021	1197.807	1340.078	929.802	
Serbia	DALYs	Female	2022	1191.003	1354.562	935.3385	
Serbia	DALYs	Female	2023	1204.302	1482.693	939.7908	
Sierra Leone	Incidence	Male	2010	3.429562	4.943629	2.363614	
Sierra Leone	Incidence	Male	2011	3.465698	4.895844	2.480131	
Sierra Leone	Incidence	Male	2012	3.535471	5.111536	2.499713	
Sierra Leone	Incidence	Male	2013	3.455875	5.038555	2.415999	
Sierra Leone	Incidence	Male	2014	3.452221	4.987333	2.457541	
Sierra Leone	Incidence	Male	2015	3.633557	5.313954	2.512993	
Sierra Leone	Incidence	Male	2016	3.573145	5.183063	2.465954	
Sierra Leone	Incidence	Male	2017	3.583169	5.161939	2.504419	
Sierra Leone	Incidence	Male	2018	3.611975	5.262823	2.481353	
Sierra Leone	Incidence	Male	2019	3.687315	5.364755	2.516336	
Sierra Leone	Incidence	Male	2020	3.912917	5.818412	2.626192	
Sierra Leone	Incidence	Male	2021	3.869334	5.763063	2.610936	
Sierra Leone	Incidence	Male	2022	4.149294	6.282873	2.838679	
Sierra Leone	Incidence	Male	2023	4.396588	6.53315	3.045532	
Sierra Leone	Incidence	Female	2010	0.851835	1.243243	0.581208	
Sierra Leone	Incidence	Female	2011	0.86193	1.233826	0.580758	
Sierra Leone	Incidence	Female	2012	0.891904	1.264333	0.599069	
Sierra Leone	Incidence	Female	2013	0.895323	1.269105	0.601357	
Sierra Leone	Incidence	Female	2014	0.893715	1.260344	0.598602	
Sierra Leone	Incidence	Female	2015	0.910364	1.230115	0.606359	
Sierra Leone	Incidence	Female	2016	0.904574	1.223111	0.616896	
Sierra Leone	Incidence	Female	2017	0.927408	1.222305	0.64233	
Sierra Leone	Incidence	Female	2018	0.939272	1.211138	0.662199	
Sierra Leone	Incidence	Female	2019	0.967637	1.262111	0.687887	
Sierra Leone	Incidence	Female	2020	0.978941	1.289376	0.70909	

Sierra Leone	Incidence	Female	2021	1.006125	1.327159	0.707966	
Sierra Leone	Incidence	Female	2022	1.087976	1.46089	0.762792	
Sierra Leone	Incidence	Female	2023	1.168098	1.548551	0.814878	
Sierra Leone	Deaths	Male	2010	3.406685	4.927173	2.337175	
Sierra Leone	Deaths	Male	2011	3.441271	4.878854	2.456262	
Sierra Leone	Deaths	Male	2012	3.510143	5.099009	2.494855	
Sierra Leone	Deaths	Male	2013	3.429787	5.022167	2.412757	
Sierra Leone	Deaths	Male	2014	3.41816	4.988241	2.431689	
Sierra Leone	Deaths	Male	2015	3.599795	5.327442	2.499013	
Sierra Leone	Deaths	Male	2016	3.532009	5.143717	2.441251	
Sierra Leone	Deaths	Male	2017	3.536372	5.101416	2.468715	
Sierra Leone	Deaths	Male	2018	3.557728	5.204501	2.437009	
Sierra Leone	Deaths	Male	2019	3.625556	5.319492	2.465566	
Sierra Leone	Deaths	Male	2020	3.858358	5.741282	2.580553	
Sierra Leone	Deaths	Male	2021	3.801699	5.662039	2.548771	
Sierra Leone	Deaths	Male	2022	4.066551	6.133037	2.782016	
Sierra Leone	Deaths	Male	2023	4.301247	6.427659	2.978232	
Sierra Leone	Deaths	Female	2010	0.855531	1.253847	0.58545	
Sierra Leone	Deaths	Female	2011	0.864374	1.232444	0.586718	
Sierra Leone	Deaths	Female	2012	0.892844	1.264948	0.596898	
Sierra Leone	Deaths	Female	2013	0.8947	1.275647	0.605978	
Sierra Leone	Deaths	Female	2014	0.889455	1.260384	0.595441	
Sierra Leone	Deaths	Female	2015	0.904246	1.222794	0.602714	
Sierra Leone	Deaths	Female	2016	0.894479	1.209578	0.607887	
Sierra Leone	Deaths	Female	2017	0.913952	1.212195	0.631583	
Sierra Leone	Deaths	Female	2018	0.922792	1.2061	0.645326	
Sierra Leone	Deaths	Female	2019	0.947454	1.237151	0.672528	
Sierra Leone	Deaths	Female	2020	0.957433	1.263412	0.687571	
Sierra Leone	Deaths	Female	2021	0.979809	1.299068	0.688694	
Sierra Leone	Deaths	Female	2022	1.055619	1.414578	0.737041	
Sierra Leone	Deaths	Female	2023	1.127815	1.496965	0.788847	
Sierra Leone	DALYs	Male	2010	97.29875	137.7343	68.53451	
Sierra Leone	DALYs	Male	2011	98.54505	138.341	71.3194	
Sierra Leone	DALYs	Male	2012	100.7369	143.4523	71.80638	
Sierra Leone	DALYs	Male	2013	98.67014	141.8486	69.50652	
Sierra Leone	DALYs	Male	2014	99.10449	140.5766	70.3298	

Sierra Leone	DALYs	Male	2015	104.4826	149.2945	72.71527	
Sierra Leone	DALYs	Male	2016	103.2943	145.5872	71.7762	
Sierra Leone	DALYs	Male	2017	103.9274	144.8578	73.66936	
Sierra Leone	DALYs	Male	2018	105.1978	149.3327	72.99258	
Sierra Leone	DALYs	Male	2019	107.5394	153.8468	74.55651	
Sierra Leone	DALYs	Male	2020	113.4517	164.6369	77.21796	
Sierra Leone	DALYs	Male	2021	113.0033	168.554	76.94062	
Sierra Leone	DALYs	Male	2022	121.3599	181.5219	83.55152	
Sierra Leone	DALYs	Male	2023	128.7811	190.4819	89.06969	
Sierra Leone	DALYs	Female	2010	24.18278	35.38215	16.5149	
Sierra Leone	DALYs	Female	2011	24.63713	35.37926	16.55431	
Sierra Leone	DALYs	Female	2012	25.68087	36.2816	17.26481	
Sierra Leone	DALYs	Female	2013	25.94816	36.92875	17.37673	
Sierra Leone	DALYs	Female	2014	26.2205	36.87842	17.38968	
Sierra Leone	DALYs	Female	2015	26.87042	36.74995	17.88327	
Sierra Leone	DALYs	Female	2016	27.00218	36.70546	18.51624	
Sierra Leone	DALYs	Female	2017	27.90734	36.74264	19.23261	
Sierra Leone	DALYs	Female	2018	28.46185	37.01533	20.03256	
Sierra Leone	DALYs	Female	2019	29.49264	38.59567	20.97432	
Sierra Leone	DALYs	Female	2020	29.80564	39.37577	21.30176	
Sierra Leone	DALYs	Female	2021	30.86264	40.21661	21.67276	
Sierra Leone	DALYs	Female	2022	33.42304	44.36289	23.17791	
Sierra Leone	DALYs	Female	2023	36.20689	48.44293	25.07319	
Singapore	Incidence	Male	2010	35.73922	39.0267	32.96127	
Singapore	Incidence	Male	2011	36.49627	39.98585	33.78585	
Singapore	Incidence	Male	2012	37.99294	41.89735	34.91664	
Singapore	Incidence	Male	2013	38.79327	42.66545	35.54792	
Singapore	Incidence	Male	2014	39.14796	43.01788	35.55468	
Singapore	Incidence	Male	2015	38.66188	43.1379	34.85871	
Singapore	Incidence	Male	2016	38.6064	43.11292	34.38611	
Singapore	Incidence	Male	2017	39.76671	44.34921	35.23266	
Singapore	Incidence	Male	2018	39.47617	44.55857	34.7595	
Singapore	Incidence	Male	2019	39.77783	45.57968	35.0903	
Singapore	Incidence	Male	2020	39.65197	45.6628	34.57667	
Singapore	Incidence	Male	2021	40.73193	46.78371	35.52647	
Singapore	Incidence	Male	2022	42.04043	47.57943	37.13926	

Singapore	Incidence	Male	2023	35.14893	49.92221	23.32122	
Singapore	Incidence	Female	2010	20.59947	23.82203	17.78315	
Singapore	Incidence	Female	2011	21.38019	24.64916	18.57711	
Singapore	Incidence	Female	2012	22.42988	25.94373	19.43105	
Singapore	Incidence	Female	2013	22.59451	25.93272	19.52842	
Singapore	Incidence	Female	2014	22.86624	26.10313	19.59168	
Singapore	Incidence	Female	2015	22.99707	26.2328	19.59153	
Singapore	Incidence	Female	2016	22.61346	25.74153	19.07402	
Singapore	Incidence	Female	2017	22.83548	25.8676	19.21345	
Singapore	Incidence	Female	2018	22.86185	25.72249	18.95667	
Singapore	Incidence	Female	2019	23.47143	26.52345	19.1208	
Singapore	Incidence	Female	2020	22.96801	25.72313	18.95834	
Singapore	Incidence	Female	2021	24.81884	27.85697	20.63092	
Singapore	Incidence	Female	2022	26.56631	29.78766	21.84865	
Singapore	Incidence	Female	2023	28.26201	32.29181	23.16412	
Singapore	Deaths	Male	2010	29.45149	31.015	28.03346	
Singapore	Deaths	Male	2011	29.85434	31.30455	28.46142	
Singapore	Deaths	Male	2012	30.79852	32.45115	29.4213	
Singapore	Deaths	Male	2013	31.04217	32.64309	29.56132	
Singapore	Deaths	Male	2014	31.05582	32.67429	29.44048	
Singapore	Deaths	Male	2015	30.35353	32.04993	28.65172	
Singapore	Deaths	Male	2016	30.01628	31.62139	28.31813	
Singapore	Deaths	Male	2017	30.59091	32.38379	28.92596	
Singapore	Deaths	Male	2018	30.13791	32.14458	28.3873	
Singapore	Deaths	Male	2019	30.17311	32.25343	28.26486	
Singapore	Deaths	Male	2020	29.93343	32.21907	28.00614	
Singapore	Deaths	Male	2021	30.60069	32.78204	28.6794	
Singapore	Deaths	Male	2022	31.53091	33.78582	29.45385	
Singapore	Deaths	Male	2023	26.3492	35.59178	18.3889	
Singapore	Deaths	Female	2010	16.13596	17.71392	14.43546	
Singapore	Deaths	Female	2011	16.50655	18.06372	14.87709	
Singapore	Deaths	Female	2012	17.11519	18.72825	15.41889	
Singapore	Deaths	Female	2013	17.06784	18.53544	15.32351	
Singapore	Deaths	Female	2014	17.10589	18.47803	15.29028	
Singapore	Deaths	Female	2015	16.97316	18.3138	14.98437	
Singapore	Deaths	Female	2016	16.39174	17.75941	14.52891	

Singapore	Deaths	Female	2017	16.4655	17.93422	14.52089	
Singapore	Deaths	Female	2018	16.31147	17.81622	14.1849	
Singapore	Deaths	Female	2019	16.68389	18.3993	14.33518	
Singapore	Deaths	Female	2020	16.33312	17.99162	14.1849	
Singapore	Deaths	Female	2021	17.54525	19.33108	15.23213	
Singapore	Deaths	Female	2022	18.76795	20.70046	16.39169	
Singapore	Deaths	Female	2023	20.1826	22.76397	17.2165	
Singapore	DALYs	Male	2010	675.8964	705.7527	641.3199	
Singapore	DALYs	Male	2011	683.2667	717.4666	654.6399	
Singapore	DALYs	Male	2012	702.0608	739.3369	669.5749	
Singapore	DALYs	Male	2013	709.0399	742.9069	673.3169	
Singapore	DALYs	Male	2014	696.6181	734.9499	661.1519	
Singapore	DALYs	Male	2015	675.5348	711.8026	642.0385	
Singapore	DALYs	Male	2016	658.765	693.8256	627.0574	
Singapore	DALYs	Male	2017	669.0318	707.9472	629.8443	
Singapore	DALYs	Male	2018	652.351	694.921	612.1161	
Singapore	DALYs	Male	2019	646.2681	692.1436	605.0268	
Singapore	DALYs	Male	2020	630.4097	679.5094	590.995	
Singapore	DALYs	Male	2021	640.1336	690.2018	600.1727	
Singapore	DALYs	Male	2022	654.8895	703.5315	614.2613	
Singapore	DALYs	Male	2023	543.1309	736.705	381.7997	
Singapore	DALYs	Female	2010	355.0974	388.5856	326.4918	
Singapore	DALYs	Female	2011	366.5775	401.8918	337.3347	
Singapore	DALYs	Female	2012	378.3573	413.8645	347.4475	
Singapore	DALYs	Female	2013	372.3545	407.6913	340.7467	
Singapore	DALYs	Female	2014	367.799	396.2746	337.8369	
Singapore	DALYs	Female	2015	363.484	392.5794	328.751	
Singapore	DALYs	Female	2016	352.174	379.8213	320.5216	
Singapore	DALYs	Female	2017	345.4651	373.1667	312.7833	
Singapore	DALYs	Female	2018	343.1656	371.8788	306.6201	
Singapore	DALYs	Female	2019	345.4957	376.8151	307.1242	
Singapore	DALYs	Female	2020	332.6648	364.0353	292.8236	
Singapore	DALYs	Female	2021	354.9005	384.8785	313.7281	
Singapore	DALYs	Female	2022	375.2612	408.0217	336.2241	
Singapore	DALYs	Female	2023	394.4683	440.2029	343.9522	
Slovakia	Incidence	Male	2010	70.37814	89.47311	62.09238	

Slovakia	Incidence	Male	2011	70.81265	90.28275	62.32459	
Slovakia	Incidence	Male	2012	71.20585	91.52653	62.9755	
Slovakia	Incidence	Male	2013	72.5157	94.5673	64.65668	
Slovakia	Incidence	Male	2014	73.2703	96.05586	65.5669	
Slovakia	Incidence	Male	2015	67.08514	87.29531	60.38592	
Slovakia	Incidence	Male	2016	68.44279	90.62887	61.49755	
Slovakia	Incidence	Male	2017	69.42653	93.5254	62.98009	
Slovakia	Incidence	Male	2018	69.41787	94.2973	62.79333	
Slovakia	Incidence	Male	2019	68.91463	92.89304	62.43143	
Slovakia	Incidence	Male	2020	69.25532	93.60014	62.59414	
Slovakia	Incidence	Male	2021	70.8952	96.74552	64.45793	
Slovakia	Incidence	Male	2022	71.42061	97.49403	63.95951	
Slovakia	Incidence	Male	2023	69.93609	93.01161	62.279	
Slovakia	Incidence	Female	2010	20.68245	23.63654	17.94001	
Slovakia	Incidence	Female	2011	21.24325	24.15328	18.32029	
Slovakia	Incidence	Female	2012	21.91187	24.90861	18.5299	
Slovakia	Incidence	Female	2013	22.65046	25.69112	18.99872	
Slovakia	Incidence	Female	2014	23.4258	27.1044	19.64909	
Slovakia	Incidence	Female	2015	23.82942	27.34505	19.65996	
Slovakia	Incidence	Female	2016	24.61116	28.09057	19.8835	
Slovakia	Incidence	Female	2017	25.3213	28.86293	20.2891	
Slovakia	Incidence	Female	2018	25.54418	29.2905	20.48272	
Slovakia	Incidence	Female	2019	25.72113	28.98852	20.62465	
Slovakia	Incidence	Female	2020	26.25622	29.3981	21.04783	
Slovakia	Incidence	Female	2021	26.75977	29.64395	21.28361	
Slovakia	Incidence	Female	2022	26.29008	29.68695	20.89924	
Slovakia	Incidence	Female	2023	26.08324	30.02211	21.02679	
Slovakia	Deaths	Male	2010	69.24293	87.7324	61.43828	
Slovakia	Deaths	Male	2011	69.69319	88.36998	61.67629	
Slovakia	Deaths	Male	2012	70.14076	89.59258	62.31888	
Slovakia	Deaths	Male	2013	71.46889	92.82886	63.7324	
Slovakia	Deaths	Male	2014	72.24701	93.63969	64.88461	
Slovakia	Deaths	Male	2015	66.18777	84.81737	59.6088	
Slovakia	Deaths	Male	2016	67.56373	88.02932	60.78434	
Slovakia	Deaths	Male	2017	68.58815	91.35176	61.69091	
Slovakia	Deaths	Male	2018	68.61009	91.8488	62.38589	

Slovakia	Deaths	Male	2019	68.13668	90.49983	61.68539	
Slovakia	Deaths	Male	2020	68.50565	91.27132	62.10065	
Slovakia	Deaths	Male	2021	69.9618	94.10522	63.62316	
Slovakia	Deaths	Male	2022	70.42065	94.97194	62.97973	
Slovakia	Deaths	Male	2023	69.14769	90.61625	60.77343	
Slovakia	Deaths	Female	2010	20.60453	23.78295	18.06983	
Slovakia	Deaths	Female	2011	21.1465	24.17832	18.43611	
Slovakia	Deaths	Female	2012	21.80685	24.71356	18.83137	
Slovakia	Deaths	Female	2013	22.52537	25.72614	19.14562	
Slovakia	Deaths	Female	2014	23.26804	26.9978	19.74623	
Slovakia	Deaths	Female	2015	23.68391	27.16214	19.63863	
Slovakia	Deaths	Female	2016	24.4559	27.70614	19.87895	
Slovakia	Deaths	Female	2017	25.18171	28.33992	20.2536	
Slovakia	Deaths	Female	2018	25.40896	28.78909	20.40234	
Slovakia	Deaths	Female	2019	25.58627	28.69186	20.5266	
Slovakia	Deaths	Female	2020	26.12423	29.10521	20.93209	
Slovakia	Deaths	Female	2021	26.60204	29.27924	21.13566	
Slovakia	Deaths	Female	2022	26.16812	29.31281	20.89463	
Slovakia	Deaths	Female	2023	25.94203	29.70206	20.92807	
Slovakia	DALYs	Male	2010	1772.88	2300.409	1555.504	
Slovakia	DALYs	Male	2011	1770.486	2296.937	1556.648	
Slovakia	DALYs	Male	2012	1766.112	2331.41	1550.941	
Slovakia	DALYs	Male	2013	1786.092	2403.24	1584.333	
Slovakia	DALYs	Male	2014	1791.127	2400.511	1603.072	
Slovakia	DALYs	Male	2015	1627.872	2163.51	1468.932	
Slovakia	DALYs	Male	2016	1649.397	2240.711	1484.637	
Slovakia	DALYs	Male	2017	1659.555	2305.993	1506.087	
Slovakia	DALYs	Male	2018	1646.31	2306.054	1494.848	
Slovakia	DALYs	Male	2019	1622.566	2267.942	1465.253	
Slovakia	DALYs	Male	2020	1619.354	2278.246	1451.504	
Slovakia	DALYs	Male	2021	1659.226	2353.898	1496.679	
Slovakia	DALYs	Male	2022	1671.241	2368.031	1495.729	
Slovakia	DALYs	Male	2023	1622.11	2230.585	1444.035	
Slovakia	DALYs	Female	2010	502.6597	565.0444	431.3338	
Slovakia	DALYs	Female	2011	513.0042	579.6696	432.1293	
Slovakia	DALYs	Female	2012	526.0498	591.3939	436.116	

Slovakia	DALYs	Female	2013	540.6066	614.4945	444.6121	
Slovakia	DALYs	Female	2014	555.8691	641.569	458.259	
Slovakia	DALYs	Female	2015	561.3474	645.8033	455.656	
Slovakia	DALYs	Female	2016	576.1619	656.2937	458.721	
Slovakia	DALYs	Female	2017	587.9931	666.9721	463.2023	
Slovakia	DALYs	Female	2018	588.6481	669.5744	465.8202	
Slovakia	DALYs	Female	2019	588.3069	660.8588	467.5548	
Slovakia	DALYs	Female	2020	596.636	661.8978	475.0318	
Slovakia	DALYs	Female	2021	605.9509	671.596	478.9055	
Slovakia	DALYs	Female	2022	594.1709	671.6043	476.844	
Slovakia	DALYs	Female	2023	587.2338	687.1702	473.9184	
Slovenia	Incidence	Male	2010	93.40992	99.68835	87.68037	
Slovenia	Incidence	Male	2011	94.93547	102.4557	88.45494	
Slovenia	Incidence	Male	2012	91.78835	98.35562	85.05779	
Slovenia	Incidence	Male	2013	91.13031	97.85925	84.8256	
Slovenia	Incidence	Male	2014	95.34521	102.8372	88.65987	
Slovenia	Incidence	Male	2015	95.51357	103.9173	88.79893	
Slovenia	Incidence	Male	2016	94.81612	103.5683	87.11785	
Slovenia	Incidence	Male	2017	91.98237	101.0173	84.26484	
Slovenia	Incidence	Male	2018	90.66274	100.0327	81.72557	
Slovenia	Incidence	Male	2019	87.60363	95.66586	79.55531	
Slovenia	Incidence	Male	2020	84.99697	92.63171	77.58897	
Slovenia	Incidence	Male	2021	85.8417	94.09518	78.16711	
Slovenia	Incidence	Male	2022	87.24429	95.4565	79.16603	
Slovenia	Incidence	Male	2023	84.45916	93.28356	76.50072	
Slovenia	Incidence	Female	2010	37.25042	40.80597	33.73852	
Slovenia	Incidence	Female	2011	38.76328	42.54406	34.96572	
Slovenia	Incidence	Female	2012	40.08366	44.05408	36.45832	
Slovenia	Incidence	Female	2013	41.82228	45.76673	38.00342	
Slovenia	Incidence	Female	2014	45.22529	49.55941	40.5788	
Slovenia	Incidence	Female	2015	44.33069	49.28418	40.026	
Slovenia	Incidence	Female	2016	47.09113	52.1532	41.97218	
Slovenia	Incidence	Female	2017	49.55371	55.0828	43.59385	
Slovenia	Incidence	Female	2018	49.15055	55.14938	43.34655	
Slovenia	Incidence	Female	2019	48.80729	54.68361	43.02231	
Slovenia	Incidence	Female	2020	50.266	55.92682	44.4204	

Slovenia	Incidence	Female	2021	49.16518	54.89273	43.29284	
Slovenia	Incidence	Female	2022	50.64691	56.71668	44.01985	
Slovenia	Incidence	Female	2023	49.91871	56.5058	42.68475	
Slovenia	Deaths	Male	2010	83.44954	88.52243	78.66157	
Slovenia	Deaths	Male	2011	84.74017	89.74079	78.91997	
Slovenia	Deaths	Male	2012	81.80647	86.65685	76.54613	
Slovenia	Deaths	Male	2013	81.14156	86.06116	76.40571	
Slovenia	Deaths	Male	2014	84.69133	89.01583	79.7778	
Slovenia	Deaths	Male	2015	84.95071	88.84609	80.25922	
Slovenia	Deaths	Male	2016	84.37532	89.02904	78.93788	
Slovenia	Deaths	Male	2017	82.26576	87.25858	77.15626	
Slovenia	Deaths	Male	2018	81.73761	87.02917	75.28133	
Slovenia	Deaths	Male	2019	79.4027	84.96577	73.80091	
Slovenia	Deaths	Male	2020	77.08332	81.97103	72.21686	
Slovenia	Deaths	Male	2021	77.56757	83.6097	72.11746	
Slovenia	Deaths	Male	2022	79.1543	86.41317	73.34501	
Slovenia	Deaths	Male	2023	76.9191	84.26314	70.94364	
Slovenia	Deaths	Female	2010	32.82548	35.4625	29.95573	
Slovenia	Deaths	Female	2011	33.94799	36.55736	30.97717	
Slovenia	Deaths	Female	2012	34.88321	37.84201	31.85199	
Slovenia	Deaths	Female	2013	36.00453	39.30437	32.97691	
Slovenia	Deaths	Female	2014	38.70316	42.30968	35.33775	
Slovenia	Deaths	Female	2015	37.86878	41.20916	34.54027	
Slovenia	Deaths	Female	2016	40.2498	43.56159	36.36397	
Slovenia	Deaths	Female	2017	42.48582	46.12499	38.44547	
Slovenia	Deaths	Female	2018	42.42973	46.37296	38.05143	
Slovenia	Deaths	Female	2019	42.72638	46.07812	38.47112	
Slovenia	Deaths	Female	2020	44.02011	47.40552	40.12107	
Slovenia	Deaths	Female	2021	42.68058	46.27417	38.66782	
Slovenia	Deaths	Female	2022	44.24273	48.7146	39.4897	
Slovenia	Deaths	Female	2023	43.68165	48.26502	38.10238	
Slovenia	DALYs	Male	2010	2016.143	2140.487	1901.91	
Slovenia	DALYs	Male	2011	2035.285	2158.672	1896.215	
Slovenia	DALYs	Male	2012	1953.591	2076.815	1825.734	
Slovenia	DALYs	Male	2013	1919.316	2041.64	1807.313	
Slovenia	DALYs	Male	2014	1988.054	2103.434	1866.255	

Slovenia	DALYs	Male	2015	1971.901	2077.493	1855.753	
Slovenia	DALYs	Male	2016	1949.195	2065.571	1821.863	
Slovenia	DALYs	Male	2017	1882.631	2001.303	1760.697	
Slovenia	DALYs	Male	2018	1839.485	1969.716	1707.374	
Slovenia	DALYs	Male	2019	1759.745	1884.771	1638.529	
Slovenia	DALYs	Male	2020	1697.597	1813.906	1594.483	
Slovenia	DALYs	Male	2021	1718.351	1860.579	1594.627	
Slovenia	DALYs	Male	2022	1741.869	1928.707	1605.657	
Slovenia	DALYs	Male	2023	1681.479	1860.618	1544.664	
Slovenia	DALYs	Female	2010	729.0302	791.3415	673.746	
Slovenia	DALYs	Female	2011	756.2622	814.0091	697.2256	
Slovenia	DALYs	Female	2012	778.0637	838.376	719.9247	
Slovenia	DALYs	Female	2013	807.9325	873.1207	751.0437	
Slovenia	DALYs	Female	2014	859.7893	930.8562	798.8331	
Slovenia	DALYs	Female	2015	835.7107	905.5074	773.926	
Slovenia	DALYs	Female	2016	878.9183	948.9739	808.0479	
Slovenia	DALYs	Female	2017	920.1735	993.3046	844.8689	
Slovenia	DALYs	Female	2018	905.9758	984.6525	826.5719	
Slovenia	DALYs	Female	2019	883.1433	948.6118	798.8384	
Slovenia	DALYs	Female	2020	901.609	971.0153	826.5068	
Slovenia	DALYs	Female	2021	887.8737	961.3808	816.9756	
Slovenia	DALYs	Female	2022	912.3627	999.2384	827.7682	
Slovenia	DALYs	Female	2023	901.6272	995.7722	808.2291	
Solomon Island	Incidence	Male	2010	13.02212	18.80412	8.405478	
Solomon Island	Incidence	Male	2011	14.36608	20.25322	9.131529	
Solomon Island	Incidence	Male	2012	15.01635	21.51144	9.791881	
Solomon Island	Incidence	Male	2013	15.55406	22.33392	10.19565	
Solomon Island	Incidence	Male	2014	16.39727	23.15905	10.68647	
Solomon Island	Incidence	Male	2015	17.54279	24.45678	11.4808	
Solomon Island	Incidence	Male	2016	17.43455	24.71285	11.37301	
Solomon Island	Incidence	Male	2017	17.81793	25.40235	11.24676	
Solomon Island	Incidence	Male	2018	18.11238	26.38724	11.25776	
Solomon Island	Incidence	Male	2019	18.69848	27.03324	11.59939	
Solomon Island	Incidence	Male	2020	19.47974	29.65202	12.28345	
Solomon Island	Incidence	Male	2021	20.32262	30.53803	12.67256	
Solomon Island	Incidence	Male	2022	21.34574	31.56368	13.16647	

Solomon Island	Incidence	Male	2023	21.73456	32.21937	13.29275	
Solomon Island	Incidence	Female	2010	12.44834	18.90984	8.416708	
Solomon Island	Incidence	Female	2011	14.37714	22.11578	9.740881	
Solomon Island	Incidence	Female	2012	15.4792	23.65834	10.63506	
Solomon Island	Incidence	Female	2013	16.43634	25.03513	10.99397	
Solomon Island	Incidence	Female	2014	17.89825	26.5472	11.79648	
Solomon Island	Incidence	Female	2015	18.6522	27.65001	12.11939	
Solomon Island	Incidence	Female	2016	18.5075	27.55731	12.24876	
Solomon Island	Incidence	Female	2017	19.21177	28.32097	12.74744	
Solomon Island	Incidence	Female	2018	19.62307	27.69916	13.47116	
Solomon Island	Incidence	Female	2019	20.61237	30.07979	14.26907	
Solomon Island	Incidence	Female	2020	21.4742	31.53641	14.82078	
Solomon Island	Incidence	Female	2021	21.73353	31.7961	14.7262	
Solomon Island	Incidence	Female	2022	24.26551	35.20856	16.1896	
Solomon Island	Incidence	Female	2023	24.99953	34.65942	16.79387	
Solomon Island	Deaths	Male	2010	12.86355	18.53546	8.439929	
Solomon Island	Deaths	Male	2011	14.16794	19.89913	9.154179	
Solomon Island	Deaths	Male	2012	14.78609	21.09879	9.624453	
Solomon Island	Deaths	Male	2013	15.29099	21.88676	10.03718	
Solomon Island	Deaths	Male	2014	16.09173	22.72481	10.5934	
Solomon Island	Deaths	Male	2015	17.18493	23.94657	11.33874	
Solomon Island	Deaths	Male	2016	17.04849	24.08011	11.15598	
Solomon Island	Deaths	Male	2017	17.40463	24.7522	10.99085	
Solomon Island	Deaths	Male	2018	17.67571	25.69257	11.07045	
Solomon Island	Deaths	Male	2019	18.2364	26.34808	11.44214	
Solomon Island	Deaths	Male	2020	18.98557	28.94172	11.96448	
Solomon Island	Deaths	Male	2021	19.78267	29.52729	12.34156	
Solomon Island	Deaths	Male	2022	20.76835	30.65278	12.9047	
Solomon Island	Deaths	Male	2023	21.12227	31.30428	13.00966	
Solomon Island	Deaths	Female	2010	12.04289	18.64256	8.049283	
Solomon Island	Deaths	Female	2011	13.87979	21.39676	9.343439	
Solomon Island	Deaths	Female	2012	14.91936	23.02118	10.24462	
Solomon Island	Deaths	Female	2013	15.81511	24.17045	10.54361	
Solomon Island	Deaths	Female	2014	17.18446	25.73216	11.32283	
Solomon Island	Deaths	Female	2015	17.87861	26.89595	11.59411	
Solomon Island	Deaths	Female	2016	17.71078	26.55791	11.63191	

Solomon Island	Deaths	Female	2017	18.36426	27.40982	12.12042	
Solomon Island	Deaths	Female	2018	18.74321	27.11413	12.82037	
Solomon Island	Deaths	Female	2019	19.6823	29.25736	13.65316	
Solomon Island	Deaths	Female	2020	20.48907	30.79841	14.10539	
Solomon Island	Deaths	Female	2021	20.69073	30.99399	13.97526	
Solomon Island	Deaths	Female	2022	23.12521	34.23365	15.45492	
Solomon Island	Deaths	Female	2023	23.82029	33.64804	15.93298	
Solomon Island	DALYs	Male	2010	361.4945	529.3881	229.8	
Solomon Island	DALYs	Male	2011	399.5076	563.9231	253.7222	
Solomon Island	DALYs	Male	2012	418.6746	601.4622	273.3344	
Solomon Island	DALYs	Male	2013	434.7795	627.0602	285.3556	
Solomon Island	DALYs	Male	2014	459.2207	653.4879	296.8686	
Solomon Island	DALYs	Male	2015	492.445	690.9317	321.0087	
Solomon Island	DALYs	Male	2016	490.8508	697.9582	312.7966	
Solomon Island	DALYs	Male	2017	502.4019	718.1536	317.4087	
Solomon Island	DALYs	Male	2018	511.7622	749.0155	318.21	
Solomon Island	DALYs	Male	2019	528.9392	774.4187	329.3926	
Solomon Island	DALYs	Male	2020	551.5284	842.908	344.215	
Solomon Island	DALYs	Male	2021	576.3876	873.839	356.2883	
Solomon Island	DALYs	Male	2022	605.4157	897.0173	374.8061	
Solomon Island	DALYs	Male	2023	617.6487	919.9604	374.3749	
Solomon Island	DALYs	Female	2010	367.738	547.9057	249.8392	
Solomon Island	DALYs	Female	2011	426.0346	635.9463	291.9043	
Solomon Island	DALYs	Female	2012	459.7684	690.0038	316.8342	
Solomon Island	DALYs	Female	2013	489.1964	727.4004	324.5833	
Solomon Island	DALYs	Female	2014	533.6325	786.4288	353.3339	
Solomon Island	DALYs	Female	2015	556.6156	812.0591	365.0696	
Solomon Island	DALYs	Female	2016	553.1149	793.6949	369.6589	
Solomon Island	DALYs	Female	2017	574.5418	824.3145	384.0033	
Solomon Island	DALYs	Female	2018	587.7101	830.2043	408.1174	
Solomon Island	DALYs	Female	2019	617.3142	868.2703	426.5108	
Solomon Island	DALYs	Female	2020	643.8327	904.0573	447.7336	
Solomon Island	DALYs	Female	2021	654.5194	905.9274	446.0698	
Solomon Island	DALYs	Female	2022	726.8745	1011.287	488.1406	
Solomon Island	DALYs	Female	2023	748.6262	1037.208	504.9242	
Somalia	Incidence	Male	2010	1.224517	2.061261	0.625554	

Somalia	Incidence	Male	2011	1.246758	2.031984	0.64612	
Somalia	Incidence	Male	2012	1.263742	2.006244	0.667594	
Somalia	Incidence	Male	2013	1.277107	1.97292	0.690624	
Somalia	Incidence	Male	2014	1.289623	2.037905	0.717455	
Somalia	Incidence	Male	2015	1.312005	2.140137	0.746372	
Somalia	Incidence	Male	2016	1.344449	2.190558	0.765768	
Somalia	Incidence	Male	2017	1.362705	2.123799	0.788047	
Somalia	Incidence	Male	2018	1.372309	2.15836	0.803658	
Somalia	Incidence	Male	2019	1.379877	2.19407	0.80021	
Somalia	Incidence	Male	2020	1.464647	2.276314	0.834758	
Somalia	Incidence	Male	2021	1.923578	2.95781	1.113847	
Somalia	Incidence	Male	2022	1.488619	2.25381	0.826678	
Somalia	Incidence	Male	2023	1.535906	2.383408	0.876524	
Somalia	Incidence	Female	2010	0.46792	0.81687	0.259764	
Somalia	Incidence	Female	2011	0.474387	0.817405	0.270042	
Somalia	Incidence	Female	2012	0.479268	0.860483	0.265935	
Somalia	Incidence	Female	2013	0.481955	0.847271	0.265861	
Somalia	Incidence	Female	2014	0.490348	0.885693	0.26936	
Somalia	Incidence	Female	2015	0.502849	0.8764	0.278641	
Somalia	Incidence	Female	2016	0.520545	0.894912	0.297815	
Somalia	Incidence	Female	2017	0.535357	0.907122	0.302673	
Somalia	Incidence	Female	2018	0.547319	0.92021	0.319368	
Somalia	Incidence	Female	2019	0.562506	0.917526	0.332271	
Somalia	Incidence	Female	2020	0.517423	0.79935	0.312067	
Somalia	Incidence	Female	2021	0.590881	0.881934	0.351541	
Somalia	Incidence	Female	2022	0.608622	0.942784	0.357416	
Somalia	Incidence	Female	2023	0.636996	0.98585	0.377349	
Somalia	Deaths	Male	2010	1.19768	2.021839	0.60955	
Somalia	Deaths	Male	2011	1.220192	1.969808	0.6311	
Somalia	Deaths	Male	2012	1.237901	1.950398	0.653069	
Somalia	Deaths	Male	2013	1.253015	1.936069	0.675909	
Somalia	Deaths	Male	2014	1.266787	2.005656	0.703233	
Somalia	Deaths	Male	2015	1.290101	2.100595	0.728692	
Somalia	Deaths	Male	2016	1.323076	2.153123	0.749333	
Somalia	Deaths	Male	2017	1.341847	2.095216	0.778462	
Somalia	Deaths	Male	2018	1.351883	2.126714	0.792051	

Somalia	Deaths	Male	2019	1.360332	2.146693	0.788193	
Somalia	Deaths	Male	2020	1.460955	2.260373	0.829649	
Somalia	Deaths	Male	2021	1.933988	2.981673	1.111246	
Somalia	Deaths	Male	2022	1.479117	2.24416	0.813928	
Somalia	Deaths	Male	2023	1.524785	2.379854	0.869831	
Somalia	Deaths	Female	2010	0.451857	0.789951	0.250795	
Somalia	Deaths	Female	2011	0.458303	0.791744	0.259559	
Somalia	Deaths	Female	2012	0.463207	0.827899	0.256999	
Somalia	Deaths	Female	2013	0.466406	0.819995	0.257628	
Somalia	Deaths	Female	2014	0.474962	0.858561	0.260826	
Somalia	Deaths	Female	2015	0.487309	0.849062	0.269566	
Somalia	Deaths	Female	2016	0.504601	0.868269	0.287437	
Somalia	Deaths	Female	2017	0.519062	0.879843	0.292834	
Somalia	Deaths	Female	2018	0.530684	0.893753	0.310976	
Somalia	Deaths	Female	2019	0.545621	0.892	0.319195	
Somalia	Deaths	Female	2020	0.50276	0.77806	0.303027	
Somalia	Deaths	Female	2021	0.572038	0.847753	0.340855	
Somalia	Deaths	Female	2022	0.591239	0.915142	0.344508	
Somalia	Deaths	Female	2023	0.619137	0.960654	0.364098	
Somalia	DALYs	Male	2010	36.26458	61.38231	18.77859	
Somalia	DALYs	Male	2011	36.90978	61.56093	19.36651	
Somalia	DALYs	Male	2012	37.32318	60.30625	19.9941	
Somalia	DALYs	Male	2013	37.55716	57.88381	20.53809	
Somalia	DALYs	Male	2014	37.84491	59.38831	21.28038	
Somalia	DALYs	Male	2015	38.44799	62.76926	21.68959	
Somalia	DALYs	Male	2016	39.35798	63.74522	22.59763	
Somalia	DALYs	Male	2017	39.88188	61.45229	22.81991	
Somalia	DALYs	Male	2018	40.18601	63.09431	23.53175	
Somalia	DALYs	Male	2019	40.39048	64.89823	23.36673	
Somalia	DALYs	Male	2020	41.92211	66.65039	23.79976	
Somalia	DALYs	Male	2021	53.97482	82.6747	31.31433	
Somalia	DALYs	Male	2022	42.77799	64.77292	23.98539	
Somalia	DALYs	Male	2023	44.18457	67.32933	25.45223	
Somalia	DALYs	Female	2010	14.5839	25.37533	8.164456	
Somalia	DALYs	Female	2011	14.77231	25.45825	8.461968	
Somalia	DALYs	Female	2012	14.89378	26.37786	8.268298	

Somalia	DALYs	Female	2013	14.92493	26.17458	8.30108	
Somalia	DALYs	Female	2014	15.16107	27.3423	8.311966	
Somalia	DALYs	Female	2015	15.54055	27.3072	8.655433	
Somalia	DALYs	Female	2016	16.08547	27.44434	9.184541	
Somalia	DALYs	Female	2017	16.54494	27.91012	9.468461	
Somalia	DALYs	Female	2018	16.92783	28.52544	9.901481	
Somalia	DALYs	Female	2019	17.39273	28.07774	10.4148	
Somalia	DALYs	Female	2020	15.98611	24.61306	9.607917	
Somalia	DALYs	Female	2021	18.31176	27.32674	10.89126	
Somalia	DALYs	Female	2022	18.71613	28.9359	11.18436	
Somalia	DALYs	Female	2023	19.57188	30.41817	11.61874	
South Africa	Incidence	Male	2010	20.47489	25.12364	16.2255	
South Africa	Incidence	Male	2011	21.45259	26.13747	17.13971	
South Africa	Incidence	Male	2012	22.07904	26.94434	17.51665	
South Africa	Incidence	Male	2013	22.70947	27.69704	18.17637	
South Africa	Incidence	Male	2014	23.54119	28.94516	18.8657	
South Africa	Incidence	Male	2015	23.81262	29.18912	19.03752	
South Africa	Incidence	Male	2016	23.62986	28.82841	19.04874	
South Africa	Incidence	Male	2017	23.31671	27.92895	18.68619	
South Africa	Incidence	Male	2018	23.04565	28.0722	18.72801	
South Africa	Incidence	Male	2019	22.01816	27.00413	18.35317	
South Africa	Incidence	Male	2020	21.49801	26.0313	18.21612	
South Africa	Incidence	Male	2021	22.54217	27.62015	19.23362	
South Africa	Incidence	Male	2022	22.64968	27.51827	18.82231	
South Africa	Incidence	Male	2023	24.03355	29.51253	19.20809	
South Africa	Incidence	Female	2010	8.447379	10.11238	6.685753	
South Africa	Incidence	Female	2011	8.942419	10.60604	7.058077	
South Africa	Incidence	Female	2012	9.301057	10.9901	7.463878	
South Africa	Incidence	Female	2013	9.738566	11.61173	7.863302	
South Africa	Incidence	Female	2014	10.12682	11.87973	8.19509	
South Africa	Incidence	Female	2015	10.27731	11.92821	8.263339	
South Africa	Incidence	Female	2016	10.301	11.84897	8.227656	
South Africa	Incidence	Female	2017	10.28017	11.93946	8.211847	
South Africa	Incidence	Female	2018	10.24963	11.67001	8.318121	
South Africa	Incidence	Female	2019	9.491123	10.91142	7.915851	
South Africa	Incidence	Female	2020	8.767577	9.893763	7.243763	

South Africa	Incidence	Female	2021	8.611699	9.756316	7.215067	
South Africa	Incidence	Female	2022	9.407434	11.02509	7.72156	
South Africa	Incidence	Female	2023	10.70659	12.82919	8.479268	
South Africa	Deaths	Male	2010	19.96628	24.36219	15.77788	
South Africa	Deaths	Male	2011	20.90902	25.33533	16.70147	
South Africa	Deaths	Male	2012	21.50228	26.20914	16.99159	
South Africa	Deaths	Male	2013	22.10604	26.85078	17.67036	
South Africa	Deaths	Male	2014	22.92101	28.07113	18.37166	
South Africa	Deaths	Male	2015	23.19614	28.27373	18.63781	
South Africa	Deaths	Male	2016	23.03749	27.97553	18.5401	
South Africa	Deaths	Male	2017	22.74211	27.10991	18.1092	
South Africa	Deaths	Male	2018	22.48372	27.26126	18.15975	
South Africa	Deaths	Male	2019	21.46196	26.24072	17.92446	
South Africa	Deaths	Male	2020	20.93746	25.24762	17.57396	
South Africa	Deaths	Male	2021	21.92544	26.76007	18.52597	
South Africa	Deaths	Male	2022	21.98817	26.636	18.22047	
South Africa	Deaths	Male	2023	23.38643	28.50923	18.6519	
South Africa	Deaths	Female	2010	8.491654	10.20696	6.700544	
South Africa	Deaths	Female	2011	8.996356	10.65491	7.076792	
South Africa	Deaths	Female	2012	9.349283	11.07794	7.436436	
South Africa	Deaths	Female	2013	9.786936	11.6026	7.902854	
South Africa	Deaths	Female	2014	10.19315	11.94193	8.164762	
South Africa	Deaths	Female	2015	10.35893	12.11165	8.193576	
South Africa	Deaths	Female	2016	10.39099	12.01997	8.155093	
South Africa	Deaths	Female	2017	10.35862	11.93787	8.144754	
South Africa	Deaths	Female	2018	10.32955	11.76242	8.29853	
South Africa	Deaths	Female	2019	9.573646	10.9624	7.924061	
South Africa	Deaths	Female	2020	8.868295	10.0027	7.204898	
South Africa	Deaths	Female	2021	8.726036	9.981684	7.150134	
South Africa	Deaths	Female	2022	9.450373	11.09269	7.706405	
South Africa	Deaths	Female	2023	10.74205	12.81153	8.372405	
South Africa	DALYs	Male	2010	577.6936	711.3187	461.0264	
South Africa	DALYs	Male	2011	604.2851	742.7728	484.7241	
South Africa	DALYs	Male	2012	621.9085	761.7339	495.4992	
South Africa	DALYs	Male	2013	638.9349	787.4249	515.0587	
South Africa	DALYs	Male	2014	660.8037	810.4515	522.255	

South Africa	DALYs	Male	2015	666.9237	819.0738	528.9022	
South Africa	DALYs	Male	2016	660.0264	811.5971	524.1803	
South Africa	DALYs	Male	2017	650.0722	790.1345	516.4255	
South Africa	DALYs	Male	2018	640.9193	791.1375	514.0894	
South Africa	DALYs	Male	2019	612.4761	759.2688	495.3981	
South Africa	DALYs	Male	2020	597.534	732.1412	510.0423	
South Africa	DALYs	Male	2021	626.9169	780.4586	540.251	
South Africa	DALYs	Male	2022	632.7679	776.7981	521.3529	
South Africa	DALYs	Male	2023	667.5917	831.1472	528.7007	
South Africa	DALYs	Female	2010	222.9879	267.2351	177.0067	
South Africa	DALYs	Female	2011	235.1702	281.3437	185.6774	
South Africa	DALYs	Female	2012	244.611	293.2634	194.7642	
South Africa	DALYs	Female	2013	255.4694	307.403	205.2398	
South Africa	DALYs	Female	2014	264.0971	312.2034	211.5847	
South Africa	DALYs	Female	2015	266.7602	315.332	216.6142	
South Africa	DALYs	Female	2016	266.6967	311.1967	216.9465	
South Africa	DALYs	Female	2017	266.3161	308.8213	217.9621	
South Africa	DALYs	Female	2018	264.8621	305.8055	221.5518	
South Africa	DALYs	Female	2019	244.8493	285.4472	207.0502	
South Africa	DALYs	Female	2020	224.7417	258.1258	191.7692	
South Africa	DALYs	Female	2021	219.5974	252.3467	190.0366	
South Africa	DALYs	Female	2022	243.7291	291.7942	203.982	
South Africa	DALYs	Female	2023	276.9158	331.7403	223.3256	
South Sudan	Incidence	Male	2010	3.909321	5.934602	2.173949	
South Sudan	Incidence	Male	2011	3.939542	5.668602	2.183315	
South Sudan	Incidence	Male	2012	3.971264	5.671838	2.186598	
South Sudan	Incidence	Male	2013	4.013973	5.964377	2.231027	
South Sudan	Incidence	Male	2014	4.016755	5.884725	2.288444	
South Sudan	Incidence	Male	2015	4.253632	6.060538	2.463904	
South Sudan	Incidence	Male	2016	4.377048	6.285737	2.587487	
South Sudan	Incidence	Male	2017	4.538046	6.523683	2.634927	
South Sudan	Incidence	Male	2018	4.909148	7.206439	2.79528	
South Sudan	Incidence	Male	2019	5.085624	7.520735	2.896758	
South Sudan	Incidence	Male	2020	5.140242	7.927664	2.942371	
South Sudan	Incidence	Male	2021	4.983797	7.788955	2.882794	
South Sudan	Incidence	Male	2022	5.554941	8.629973	3.147412	

South Sudan	Incidence	Male	2023	5.794301	8.705158	3.231092	
South Sudan	Incidence	Female	2010	1.371416	2.067901	0.88236	
South Sudan	Incidence	Female	2011	1.390336	2.031192	0.887889	
South Sudan	Incidence	Female	2012	1.404276	2.02333	0.891557	
South Sudan	Incidence	Female	2013	1.431006	2.09978	0.890905	
South Sudan	Incidence	Female	2014	1.465043	2.106027	0.91952	
South Sudan	Incidence	Female	2015	1.539158	2.164233	0.953889	
South Sudan	Incidence	Female	2016	1.577428	2.238082	0.991783	
South Sudan	Incidence	Female	2017	1.600224	2.272941	1.026896	
South Sudan	Incidence	Female	2018	1.710496	2.361312	1.104167	
South Sudan	Incidence	Female	2019	1.792574	2.482141	1.163291	
South Sudan	Incidence	Female	2020	1.751003	2.372081	1.117787	
South Sudan	Incidence	Female	2021	1.712712	2.344715	1.100363	
South Sudan	Incidence	Female	2022	2.099838	2.833852	1.325298	
South Sudan	Incidence	Female	2023	2.207637	3.064941	1.376575	
South Sudan	Deaths	Male	2010	3.785074	5.748323	2.089333	
South Sudan	Deaths	Male	2011	3.811456	5.48784	2.11218	
South Sudan	Deaths	Male	2012	3.838036	5.470163	2.104252	
South Sudan	Deaths	Male	2013	3.873134	5.729041	2.128788	
South Sudan	Deaths	Male	2014	3.870597	5.669459	2.211244	
South Sudan	Deaths	Male	2015	4.095877	5.830498	2.371926	
South Sudan	Deaths	Male	2016	4.215099	6.022318	2.491449	
South Sudan	Deaths	Male	2017	4.372316	6.260917	2.548075	
South Sudan	Deaths	Male	2018	4.735151	6.9211	2.697543	
South Sudan	Deaths	Male	2019	4.908915	7.260667	2.808602	
South Sudan	Deaths	Male	2020	4.97921	7.679788	2.844693	
South Sudan	Deaths	Male	2021	4.819987	7.548541	2.754608	
South Sudan	Deaths	Male	2022	5.374494	8.313483	3.030597	
South Sudan	Deaths	Male	2023	5.601629	8.43831	3.124474	
South Sudan	Deaths	Female	2010	1.279882	1.931022	0.822184	
South Sudan	Deaths	Female	2011	1.295616	1.884265	0.82824	
South Sudan	Deaths	Female	2012	1.306542	1.882236	0.836438	
South Sudan	Deaths	Female	2013	1.328613	1.935266	0.837291	
South Sudan	Deaths	Female	2014	1.356333	1.943285	0.849202	
South Sudan	Deaths	Female	2015	1.421131	1.990325	0.881167	
South Sudan	Deaths	Female	2016	1.456542	2.064696	0.920258	

South Sudan	Deaths	Female	2017	1.480238	2.104614	0.953552	
South Sudan	Deaths	Female	2018	1.584072	2.179025	1.030982	
South Sudan	Deaths	Female	2019	1.659969	2.303742	1.069708	
South Sudan	Deaths	Female	2020	1.620615	2.188475	1.02989	
South Sudan	Deaths	Female	2021	1.583525	2.157133	1.008717	
South Sudan	Deaths	Female	2022	1.942732	2.630233	1.217214	
South Sudan	Deaths	Female	2023	2.044196	2.823981	1.270505	
South Sudan	DALYs	Male	2010	118.6118	180.7539	67.64516	
South Sudan	DALYs	Male	2011	119.6776	172.1054	67.81254	
South Sudan	DALYs	Male	2012	120.9044	174.2033	67.46758	
South Sudan	DALYs	Male	2013	122.5689	184.1247	69.16908	
South Sudan	DALYs	Male	2014	122.946	180.5717	70.49606	
South Sudan	DALYs	Male	2015	130.2488	186.3815	74.95527	
South Sudan	DALYs	Male	2016	133.8225	195.4356	77.79796	
South Sudan	DALYs	Male	2017	138.4057	201.4566	79.55305	
South Sudan	DALYs	Male	2018	148.9942	218.9303	84.4761	
South Sudan	DALYs	Male	2019	153.9267	229.716	87.65967	
South Sudan	DALYs	Male	2020	154.5482	239.089	88.44584	
South Sudan	DALYs	Male	2021	150.2989	234.1503	87.16254	
South Sudan	DALYs	Male	2022	166.896	261.147	93.40004	
South Sudan	DALYs	Male	2023	174.0652	261.9349	96.92323	
South Sudan	DALYs	Female	2010	46.1086	69.6613	29.79435	
South Sudan	DALYs	Female	2011	46.80742	68.83076	29.95924	
South Sudan	DALYs	Female	2012	47.3675	68.27174	29.87983	
South Sudan	DALYs	Female	2013	48.41288	71.35629	29.71508	
South Sudan	DALYs	Female	2014	49.78483	71.94931	30.88542	
South Sudan	DALYs	Female	2015	52.4615	73.89281	32.94882	
South Sudan	DALYs	Female	2016	53.63321	76.69268	33.63009	
South Sudan	DALYs	Female	2017	54.10096	77.93515	34.53299	
South Sudan	DALYs	Female	2018	57.45437	80.01266	37.35772	
South Sudan	DALYs	Female	2019	60.13264	83.88188	39.32048	
South Sudan	DALYs	Female	2020	58.86087	79.9223	38.02042	
South Sudan	DALYs	Female	2021	57.74159	78.82857	36.92564	
South Sudan	DALYs	Female	2022	70.41933	94.34773	44.51624	
South Sudan	DALYs	Female	2023	73.88009	103.1289	45.86684	
Spain	Incidence	Male	2010	99.12302	107.3745	91.3541	

Spain	Incidence	Male	2011	103.0512	111.27	95.08591	
Spain	Incidence	Male	2012	105.1168	114.4677	97.27845	
Spain	Incidence	Male	2013	103.8706	113.6168	95.61813	
Spain	Incidence	Male	2014	104.1165	113.3322	95.30304	
Spain	Incidence	Male	2015	104.2609	114.031	95.80201	
Spain	Incidence	Male	2016	103.02	113.3569	93.82926	
Spain	Incidence	Male	2017	102.5523	112.7467	92.76166	
Spain	Incidence	Male	2018	103.7122	113.819	93.33314	
Spain	Incidence	Male	2019	101.2011	112.0144	91.06593	
Spain	Incidence	Male	2020	100.4366	110.3435	90.40281	
Spain	Incidence	Male	2021	98.05868	108.783	88.06208	
Spain	Incidence	Male	2022	104.0736	115.7346	92.82965	
Spain	Incidence	Male	2023	101.3445	113.6318	90.41778	
Spain	Incidence	Female	2010	21.23555	23.77991	18.85578	
Spain	Incidence	Female	2011	22.53408	25.35161	20.00136	
Spain	Incidence	Female	2012	24.29744	27.46733	21.18904	
Spain	Incidence	Female	2013	25.1866	28.33912	21.92374	
Spain	Incidence	Female	2014	26.04242	29.16549	22.70832	
Spain	Incidence	Female	2015	27.43067	30.85397	23.98432	
Spain	Incidence	Female	2016	28.26324	32.08039	24.48288	
Spain	Incidence	Female	2017	29.58146	33.5002	25.39279	
Spain	Incidence	Female	2018	30.80936	34.66977	26.32867	
Spain	Incidence	Female	2019	31.41961	35.86063	26.89745	
Spain	Incidence	Female	2020	32.9066	36.90182	28.49567	
Spain	Incidence	Female	2021	33.24429	37.54748	28.51837	
Spain	Incidence	Female	2022	36.14327	40.83784	30.97343	
Spain	Incidence	Female	2023	35.66869	41.10981	30.19098	
Spain	Deaths	Male	2010	82.29234	85.69659	78.20736	
Spain	Deaths	Male	2011	84.79259	88.24058	80.06215	
Spain	Deaths	Male	2012	85.83883	89.47216	81.38443	
Spain	Deaths	Male	2013	84.59427	88.49798	80.14494	
Spain	Deaths	Male	2014	84.79339	88.61329	80.15797	
Spain	Deaths	Male	2015	85.14411	88.76378	80.78181	
Spain	Deaths	Male	2016	84.24253	87.74087	79.77432	
Spain	Deaths	Male	2017	84.11199	88.10491	79.77042	
Spain	Deaths	Male	2018	84.92291	89.28092	80.71989	

Spain	Deaths	Male	2019	82.68242	87.47545	78.37055	
Spain	Deaths	Male	2020	81.93877	87.12595	77.04673	
Spain	Deaths	Male	2021	79.92149	85.99061	74.51278	
Spain	Deaths	Male	2022	84.77613	91.85783	78.97294	
Spain	Deaths	Male	2023	82.54911	89.03313	76.54016	
Spain	Deaths	Female	2010	16.76336	17.90112	15.01558	
Spain	Deaths	Female	2011	17.67028	18.90736	15.82696	
Spain	Deaths	Female	2012	18.88529	20.21486	16.83156	
Spain	Deaths	Female	2013	19.44216	20.86833	17.30875	
Spain	Deaths	Female	2014	20.05495	21.53202	17.72579	
Spain	Deaths	Female	2015	21.16735	22.75674	18.87492	
Spain	Deaths	Female	2016	21.73347	23.3356	19.2525	
Spain	Deaths	Female	2017	22.76467	24.49111	20.23033	
Spain	Deaths	Female	2018	23.60324	25.32542	20.98191	
Spain	Deaths	Female	2019	23.88091	25.74341	21.20784	
Spain	Deaths	Female	2020	24.99289	26.70005	22.59311	
Spain	Deaths	Female	2021	25.15757	26.96643	22.52142	
Spain	Deaths	Female	2022	27.26771	29.40024	24.20528	
Spain	Deaths	Female	2023	26.85768	29.30789	23.48614	
Spain	DALYs	Male	2010	1870.027	1949.025	1781.947	
Spain	DALYs	Male	2011	1917.405	1992.128	1833.06	
Spain	DALYs	Male	2012	1926.291	2003.195	1844.673	
Spain	DALYs	Male	2013	1889.073	1966.122	1805.069	
Spain	DALYs	Male	2014	1883.938	1962.419	1798.336	
Spain	DALYs	Male	2015	1873.706	1946.844	1803.317	
Spain	DALYs	Male	2016	1847.137	1924.011	1771.605	
Spain	DALYs	Male	2017	1824.43	1908.385	1748.683	
Spain	DALYs	Male	2018	1838.832	1939.303	1754.136	
Spain	DALYs	Male	2019	1786.706	1895.441	1699.577	
Spain	DALYs	Male	2020	1757.581	1876.838	1667.067	
Spain	DALYs	Male	2021	1707.197	1845.717	1609.23	
Spain	DALYs	Male	2022	1805.216	1958.333	1694.645	
Spain	DALYs	Male	2023	1753.851	1888.315	1628.112	
Spain	DALYs	Female	2010	403.1937	424.5866	374.8364	
Spain	DALYs	Female	2011	421.1256	444.4488	389.8182	
Spain	DALYs	Female	2012	447.8839	471.0032	414.2148	

Spain	DALYs	Female	2013	461.8659	486.3974	427.704	
Spain	DALYs	Female	2014	474.6941	500.7878	439.2953	
Spain	DALYs	Female	2015	497.3485	524.4595	458.1692	
Spain	DALYs	Female	2016	511.5837	540.891	467.7793	
Spain	DALYs	Female	2017	528.9429	558.8674	483.6193	
Spain	DALYs	Female	2018	545.7008	577.4025	499.5831	
Spain	DALYs	Female	2019	553.9163	587.5818	506.4762	
Spain	DALYs	Female	2020	569.8876	603.2401	527.6913	
Spain	DALYs	Female	2021	569.8561	606.6079	524.9301	
Spain	DALYs	Female	2022	617.0928	663.5551	566.4251	
Spain	DALYs	Female	2023	607.5278	660.3069	548.9625	
Sri Lanka	Incidence	Male	2010	15.96538	18.87062	12.12291	
Sri Lanka	Incidence	Male	2011	15.95488	18.71943	12.18042	
Sri Lanka	Incidence	Male	2012	16.39663	19.42347	12.58236	
Sri Lanka	Incidence	Male	2013	17.23334	20.70916	13.15677	
Sri Lanka	Incidence	Male	2014	17.94909	21.43789	13.72445	
Sri Lanka	Incidence	Male	2015	18.10992	21.71143	13.84846	
Sri Lanka	Incidence	Male	2016	18.42982	22.69473	14.34762	
Sri Lanka	Incidence	Male	2017	19.26337	23.72103	14.86064	
Sri Lanka	Incidence	Male	2018	19.91079	24.97989	15.30941	
Sri Lanka	Incidence	Male	2019	19.75942	25.1565	15.06664	
Sri Lanka	Incidence	Male	2020	19.90033	26.14544	14.95061	
Sri Lanka	Incidence	Male	2021	19.67549	26.11688	14.73263	
Sri Lanka	Incidence	Male	2022	26.20207	34.64599	19.87667	
Sri Lanka	Incidence	Male	2023	28.03189	37.29318	21.38338	
Sri Lanka	Incidence	Female	2010	5.356596	6.365235	4.443546	
Sri Lanka	Incidence	Female	2011	5.56953	6.473616	4.598471	
Sri Lanka	Incidence	Female	2012	5.792946	6.759109	4.865441	
Sri Lanka	Incidence	Female	2013	6.133359	7.201012	5.116872	
Sri Lanka	Incidence	Female	2014	6.376467	7.711437	5.334568	
Sri Lanka	Incidence	Female	2015	6.640211	8.135987	5.443637	
Sri Lanka	Incidence	Female	2016	6.93178	8.643761	5.530955	
Sri Lanka	Incidence	Female	2017	7.406037	9.351135	5.739436	
Sri Lanka	Incidence	Female	2018	7.585271	9.650384	5.684839	
Sri Lanka	Incidence	Female	2019	7.582754	9.819462	5.65638	
Sri Lanka	Incidence	Female	2020	7.673781	10.03826	5.688146	

Sri Lanka	Incidence	Female	2021	8.286748	11.05439	6.023932	
Sri Lanka	Incidence	Female	2022	10.58496	14.45679	7.525136	
Sri Lanka	Incidence	Female	2023	11.18821	15.4642	7.791683	
Sri Lanka	Deaths	Male	2010	15.6533	18.6119	11.99762	
Sri Lanka	Deaths	Male	2011	15.63633	18.25209	12.01599	
Sri Lanka	Deaths	Male	2012	16.05214	18.97049	12.50064	
Sri Lanka	Deaths	Male	2013	16.87967	20.44088	13.03299	
Sri Lanka	Deaths	Male	2014	17.56606	21.09318	13.51938	
Sri Lanka	Deaths	Male	2015	17.7068	21.24835	13.61789	
Sri Lanka	Deaths	Male	2016	18.04297	22.16568	14.04923	
Sri Lanka	Deaths	Male	2017	18.89412	23.15803	14.73612	
Sri Lanka	Deaths	Male	2018	19.5293	24.49128	15.18011	
Sri Lanka	Deaths	Male	2019	19.39104	24.84376	14.78246	
Sri Lanka	Deaths	Male	2020	19.53453	25.46203	14.71471	
Sri Lanka	Deaths	Male	2021	19.3898	25.84929	14.57574	
Sri Lanka	Deaths	Male	2022	25.65986	33.99432	19.508	
Sri Lanka	Deaths	Male	2023	27.40324	36.14308	21.10182	
Sri Lanka	Deaths	Female	2010	5.243501	6.241583	4.373369	
Sri Lanka	Deaths	Female	2011	5.435853	6.321066	4.485387	
Sri Lanka	Deaths	Female	2012	5.64168	6.642218	4.651246	
Sri Lanka	Deaths	Female	2013	5.967259	7.010593	4.98303	
Sri Lanka	Deaths	Female	2014	6.190766	7.4525	5.173163	
Sri Lanka	Deaths	Female	2015	6.442183	7.936013	5.257683	
Sri Lanka	Deaths	Female	2016	6.706081	8.396602	5.347573	
Sri Lanka	Deaths	Female	2017	7.174939	9.095446	5.523913	
Sri Lanka	Deaths	Female	2018	7.331023	9.307438	5.49039	
Sri Lanka	Deaths	Female	2019	7.338102	9.453916	5.453521	
Sri Lanka	Deaths	Female	2020	7.364222	9.725162	5.411509	
Sri Lanka	Deaths	Female	2021	7.964198	10.62488	5.803703	
Sri Lanka	Deaths	Female	2022	10.10105	13.94403	7.240049	
Sri Lanka	Deaths	Female	2023	10.6714	14.76599	7.506544	
Sri Lanka	DALYs	Male	2010	420.1294	497.1635	309.2339	
Sri Lanka	DALYs	Male	2011	417.4271	487.8641	305.6915	
Sri Lanka	DALYs	Male	2012	426.7526	506.3298	315.5707	
Sri Lanka	DALYs	Male	2013	444.7289	537.4681	328.5243	
Sri Lanka	DALYs	Male	2014	460.6109	549.945	341.1504	

Sri Lanka	DALYs	Male	2015	463.329	555.0738	343.9321	
Sri Lanka	DALYs	Male	2016	468.1514	576.4523	352.8774	
Sri Lanka	DALYs	Male	2017	485.1761	594.1963	366.2747	
Sri Lanka	DALYs	Male	2018	498.332	629.0036	374.6863	
Sri Lanka	DALYs	Male	2019	489.6184	628.2837	368.1461	
Sri Lanka	DALYs	Male	2020	487.8496	641.0842	367.4226	
Sri Lanka	DALYs	Male	2021	476.1297	633.3225	357.3578	
Sri Lanka	DALYs	Male	2022	640.2669	855.329	479.5183	
Sri Lanka	DALYs	Male	2023	687.9415	913.684	513.5485	
Sri Lanka	DALYs	Female	2010	133.7903	160.3662	111.5345	
Sri Lanka	DALYs	Female	2011	138.7091	161.2309	115.3576	
Sri Lanka	DALYs	Female	2012	143.3523	167.51	119.7228	
Sri Lanka	DALYs	Female	2013	150.2665	175.8851	126.4264	
Sri Lanka	DALYs	Female	2014	155.2737	185.0624	131.348	
Sri Lanka	DALYs	Female	2015	160.2207	193.2445	134.9632	
Sri Lanka	DALYs	Female	2016	166.4717	204.4752	135.3953	
Sri Lanka	DALYs	Female	2017	175.927	218.713	137.0055	
Sri Lanka	DALYs	Female	2018	179.117	226.7832	136.0213	
Sri Lanka	DALYs	Female	2019	176.0841	224.4152	132.0735	
Sri Lanka	DALYs	Female	2020	177.7816	232.229	131.7822	
Sri Lanka	DALYs	Female	2021	189.4642	252.7529	140.6162	
Sri Lanka	DALYs	Female	2022	244.604	332.5411	178.5977	
Sri Lanka	DALYs	Female	2023	258.6066	356.481	183.6256	
Sudan	Incidence	Male	2010	7.349253	11.42366	4.725392	
Sudan	Incidence	Male	2011	7.411358	11.6135	4.954651	
Sudan	Incidence	Male	2012	7.475221	11.60122	5.222626	
Sudan	Incidence	Male	2013	7.598219	11.90473	5.194065	
Sudan	Incidence	Male	2014	7.595639	12.1938	4.840182	
Sudan	Incidence	Male	2015	7.966234	12.77387	5.015707	
Sudan	Incidence	Male	2016	8.08766	12.60321	4.971898	
Sudan	Incidence	Male	2017	8.292371	12.91593	5.206268	
Sudan	Incidence	Male	2018	8.393148	13.16059	5.164723	
Sudan	Incidence	Male	2019	8.513414	13.9257	5.183656	
Sudan	Incidence	Male	2020	8.940101	14.56072	5.36828	
Sudan	Incidence	Male	2021	9.394998	15.19313	5.537843	
Sudan	Incidence	Male	2022	9.877006	16.40178	5.762719	

Sudan	Incidence	Male	2023	10.12145	16.61331	5.749297	
Sudan	Incidence	Female	2010	2.756688	4.227999	1.869353	
Sudan	Incidence	Female	2011	2.83242	4.557083	1.906569	
Sudan	Incidence	Female	2012	2.89235	4.578456	1.916743	
Sudan	Incidence	Female	2013	3.004919	4.822101	2.024559	
Sudan	Incidence	Female	2014	3.101731	4.865039	2.064862	
Sudan	Incidence	Female	2015	3.264548	4.942055	2.170661	
Sudan	Incidence	Female	2016	3.408052	5.243052	2.251855	
Sudan	Incidence	Female	2017	3.543514	5.292668	2.331978	
Sudan	Incidence	Female	2018	3.61913	5.316947	2.406271	
Sudan	Incidence	Female	2019	3.761542	5.719695	2.498332	
Sudan	Incidence	Female	2020	4.058287	5.916956	2.648723	
Sudan	Incidence	Female	2021	4.336789	6.427571	2.749995	
Sudan	Incidence	Female	2022	4.506579	6.684599	2.80737	
Sudan	Incidence	Female	2023	4.612649	6.809664	2.792151	
Sudan	Deaths	Male	2010	7.512913	11.67671	4.855402	
Sudan	Deaths	Male	2011	7.568462	11.88769	5.077825	
Sudan	Deaths	Male	2012	7.629958	11.8316	5.310977	
Sudan	Deaths	Male	2013	7.757725	12.01401	5.306104	
Sudan	Deaths	Male	2014	7.745993	12.44913	4.946893	
Sudan	Deaths	Male	2015	8.11385	12.98532	5.175041	
Sudan	Deaths	Male	2016	8.225092	12.82806	5.138176	
Sudan	Deaths	Male	2017	8.424903	13.05141	5.32332	
Sudan	Deaths	Male	2018	8.522099	13.39098	5.316446	
Sudan	Deaths	Male	2019	8.635442	14.02479	5.268607	
Sudan	Deaths	Male	2020	9.067748	14.8806	5.463986	
Sudan	Deaths	Male	2021	9.502975	15.54401	5.587813	
Sudan	Deaths	Male	2022	9.981134	16.53155	5.806463	
Sudan	Deaths	Male	2023	10.21407	16.69403	5.853966	
Sudan	Deaths	Female	2010	2.705346	4.149335	1.860812	
Sudan	Deaths	Female	2011	2.774616	4.402911	1.853898	
Sudan	Deaths	Female	2012	2.82948	4.409017	1.878701	
Sudan	Deaths	Female	2013	2.935395	4.699496	1.985137	
Sudan	Deaths	Female	2014	3.022021	4.750408	2.025639	
Sudan	Deaths	Female	2015	3.173101	4.827649	2.123874	
Sudan	Deaths	Female	2016	3.306931	5.051241	2.189964	

Sudan	Deaths	Female	2017	3.431483	5.089804	2.247814	
Sudan	Deaths	Female	2018	3.498845	5.122769	2.34342	
Sudan	Deaths	Female	2019	3.635004	5.514155	2.433094	
Sudan	Deaths	Female	2020	3.908297	5.692131	2.53555	
Sudan	Deaths	Female	2021	4.160259	6.19121	2.616508	
Sudan	Deaths	Female	2022	4.329218	6.531114	2.696495	
Sudan	Deaths	Female	2023	4.427445	6.558088	2.718135	
Sudan	DALYs	Male	2010	189.2457	294.7153	120.493	
Sudan	DALYs	Male	2011	190.9729	297.5925	127.9032	
Sudan	DALYs	Male	2012	192.5084	296.5456	130.3173	
Sudan	DALYs	Male	2013	195.1157	304.7989	129.184	
Sudan	DALYs	Male	2014	194.8208	306.6947	121.7128	
Sudan	DALYs	Male	2015	204.0538	326.6249	125.5332	
Sudan	DALYs	Male	2016	207.0219	320.9609	124.8995	
Sudan	DALYs	Male	2017	212.2127	330.1408	132.781	
Sudan	DALYs	Male	2018	214.8583	336.7834	129.3932	
Sudan	DALYs	Male	2019	217.6579	361.1791	128.9955	
Sudan	DALYs	Male	2020	227.2211	372.2155	134.8419	
Sudan	DALYs	Male	2021	238.9444	378.2941	142.5558	
Sudan	DALYs	Male	2022	250.5299	420.3781	147.2214	
Sudan	DALYs	Male	2023	256.6945	422.7247	145.8556	
Sudan	DALYs	Female	2010	79.19097	123.337	52.50484	
Sudan	DALYs	Female	2011	81.42727	132.8151	55.11478	
Sudan	DALYs	Female	2012	83.17448	136.6335	54.98567	
Sudan	DALYs	Female	2013	86.25317	136.6526	57.00984	
Sudan	DALYs	Female	2014	89.05971	137.8048	59.18866	
Sudan	DALYs	Female	2015	93.73611	140.1557	61.42178	
Sudan	DALYs	Female	2016	97.6316	147.9388	64.41366	
Sudan	DALYs	Female	2017	101.5892	151.7718	66.94977	
Sudan	DALYs	Female	2018	103.9221	154.8089	69.11022	
Sudan	DALYs	Female	2019	107.7231	160.7574	70.63076	
Sudan	DALYs	Female	2020	116.1705	164.7644	76.35209	
Sudan	DALYs	Female	2021	124.369	182.5817	78.51448	
Sudan	DALYs	Female	2022	127.8597	185.8496	79.67069	
Sudan	DALYs	Female	2023	130.6349	189.6794	80.06943	
Suriname	Incidence	Male	2010	16.00483	20.65682	12.38347	

Suriname	Incidence	Male	2011	16.23831	20.90303	12.56495	
Suriname	Incidence	Male	2012	16.90523	21.69796	12.86336	
Suriname	Incidence	Male	2013	17.1368	22.44275	12.87993	
Suriname	Incidence	Male	2014	17.55387	23.55242	12.9043	
Suriname	Incidence	Male	2015	17.35661	23.36451	12.6278	
Suriname	Incidence	Male	2016	17.5516	23.46135	13.16005	
Suriname	Incidence	Male	2017	17.76966	23.64557	13.30534	
Suriname	Incidence	Male	2018	17.96141	24.1778	13.05094	
Suriname	Incidence	Male	2019	18.25796	24.65034	12.83248	
Suriname	Incidence	Male	2020	18.51143	24.94041	13.01492	
Suriname	Incidence	Male	2021	21.10968	28.21502	14.77241	
Suriname	Incidence	Male	2022	18.07785	23.77661	12.77624	
Suriname	Incidence	Male	2023	17.92708	23.81435	12.81404	
Suriname	Incidence	Female	2010	8.164911	10.23181	6.01985	
Suriname	Incidence	Female	2011	8.517289	11.08128	6.248313	
Suriname	Incidence	Female	2012	8.915545	11.46505	6.482745	
Suriname	Incidence	Female	2013	9.110852	12.05375	6.668767	
Suriname	Incidence	Female	2014	9.332829	12.29671	6.73268	
Suriname	Incidence	Female	2015	9.537273	12.84635	6.957269	
Suriname	Incidence	Female	2016	9.726065	13.35341	7.042186	
Suriname	Incidence	Female	2017	9.902557	14.09845	7.11911	
Suriname	Incidence	Female	2018	10.1222	14.80987	7.217381	
Suriname	Incidence	Female	2019	10.47432	15.51472	7.591925	
Suriname	Incidence	Female	2020	10.69638	15.68397	7.623265	
Suriname	Incidence	Female	2021	12.02093	17.2098	8.651149	
Suriname	Incidence	Female	2022	11.19513	15.93899	7.676568	
Suriname	Incidence	Female	2023	11.43241	15.68242	7.612219	
Suriname	Deaths	Male	2010	15.85324	20.36861	12.23176	
Suriname	Deaths	Male	2011	16.0831	20.74232	12.36949	
Suriname	Deaths	Male	2012	16.73021	21.55471	12.81027	
Suriname	Deaths	Male	2013	16.92133	22.22927	12.73241	
Suriname	Deaths	Male	2014	17.3521	23.35204	12.70146	
Suriname	Deaths	Male	2015	17.223	23.14997	12.47571	
Suriname	Deaths	Male	2016	17.43758	23.35818	12.97388	
Suriname	Deaths	Male	2017	17.66675	23.59293	13.29314	
Suriname	Deaths	Male	2018	17.89196	24.08942	12.97419	

Suriname	Deaths	Male	2019	18.20603	24.59506	12.80402	
Suriname	Deaths	Male	2020	18.43605	24.76463	12.89869	
Suriname	Deaths	Male	2021	20.89832	27.83555	14.6164	
Suriname	Deaths	Male	2022	18.00203	23.6897	12.6613	
Suriname	Deaths	Male	2023	17.89687	23.83425	12.81546	
Suriname	Deaths	Female	2010	8.027379	10.05956	5.919341	
Suriname	Deaths	Female	2011	8.363407	10.87413	6.114413	
Suriname	Deaths	Female	2012	8.771505	11.33108	6.384509	
Suriname	Deaths	Female	2013	8.966806	11.89377	6.525817	
Suriname	Deaths	Female	2014	9.218028	12.09004	6.662268	
Suriname	Deaths	Female	2015	9.436854	12.66361	6.885717	
Suriname	Deaths	Female	2016	9.621329	13.2829	6.947019	
Suriname	Deaths	Female	2017	9.806107	14.05111	7.060267	
Suriname	Deaths	Female	2018	10.03481	14.65037	7.152377	
Suriname	Deaths	Female	2019	10.39062	15.38543	7.438352	
Suriname	Deaths	Female	2020	10.59078	15.51529	7.555034	
Suriname	Deaths	Female	2021	11.82573	16.92568	8.417002	
Suriname	Deaths	Female	2022	11.06278	15.6943	7.529163	
Suriname	Deaths	Female	2023	11.32441	15.6481	7.526006	
Suriname	DALYs	Male	2010	423.7709	544.6489	324.9362	
Suriname	DALYs	Male	2011	429.0202	551.4781	331.2714	
Suriname	DALYs	Male	2012	446.8593	574.4865	339.8036	
Suriname	DALYs	Male	2013	454.9935	596.82	339.1217	
Suriname	DALYs	Male	2014	465.4996	624.6243	341.4914	
Suriname	DALYs	Male	2015	457.1536	624.2956	332.3546	
Suriname	DALYs	Male	2016	460.7384	622.4582	342.2936	
Suriname	DALYs	Male	2017	466.02	623.859	350.0021	
Suriname	DALYs	Male	2018	469.7438	628.5131	341.8365	
Suriname	DALYs	Male	2019	475.0542	638.4375	334.9176	
Suriname	DALYs	Male	2020	479.8677	643.2374	333.7848	
Suriname	DALYs	Male	2021	549.0573	731.7651	386.0093	
Suriname	DALYs	Male	2022	465.2579	609.2968	329.8533	
Suriname	DALYs	Male	2023	458.5856	613.3658	330.8489	
Suriname	DALYs	Female	2010	212.3983	264.666	157.5447	
Suriname	DALYs	Female	2011	221.158	284.9543	162.8931	
Suriname	DALYs	Female	2012	230.854	296.5629	168.8368	

Suriname	DALYs	Female	2013	235.4772	313.3189	172.6031	
Suriname	DALYs	Female	2014	240.0216	313.6593	172.6697	
Suriname	DALYs	Female	2015	244.5111	326.8314	178.2427	
Suriname	DALYs	Female	2016	249.0674	339.2459	180.4664	
Suriname	DALYs	Female	2017	253.2208	358.3571	182.9382	
Suriname	DALYs	Female	2018	258.5148	376.9229	183.8025	
Suriname	DALYs	Female	2019	266.3839	395.8775	192.4294	
Suriname	DALYs	Female	2020	271.0538	396.1372	193.829	
Suriname	DALYs	Female	2021	307.0325	434.1249	219.4905	
Suriname	DALYs	Female	2022	282.0347	395.4704	192.9054	
Suriname	DALYs	Female	2023	286.526	395.9589	192.4568	
Sweden	Incidence	Male	2010	48.44079	51.01485	45.50046	
Sweden	Incidence	Male	2011	48.59857	51.32466	45.80563	
Sweden	Incidence	Male	2012	47.39794	50.12538	44.8828	
Sweden	Incidence	Male	2013	45.72083	48.56296	43.25831	
Sweden	Incidence	Male	2014	45.07344	48.16726	42.50886	
Sweden	Incidence	Male	2015	44.11681	47.07032	41.65219	
Sweden	Incidence	Male	2016	43.77543	46.78327	41.00828	
Sweden	Incidence	Male	2017	42.98463	46.09639	39.9177	
Sweden	Incidence	Male	2018	41.30614	44.14891	38.34084	
Sweden	Incidence	Male	2019	39.37756	42.46176	36.3954	
Sweden	Incidence	Male	2020	38.68098	41.63608	35.7746	
Sweden	Incidence	Male	2021	36.51126	39.70364	33.85106	
Sweden	Incidence	Male	2022	35.95911	39.7683	33.15294	
Sweden	Incidence	Male	2023	36.78828	40.42091	33.44179	
Sweden	Incidence	Female	2010	45.32204	48.57727	41.09717	
Sweden	Incidence	Female	2011	45.7777	48.95973	41.65605	
Sweden	Incidence	Female	2012	46.3453	49.40369	42.12036	
Sweden	Incidence	Female	2013	47.21874	50.33969	42.96572	
Sweden	Incidence	Female	2014	47.62622	50.80206	42.96223	
Sweden	Incidence	Female	2015	46.94466	50.26761	42.08856	
Sweden	Incidence	Female	2016	47.00057	50.46886	42.19151	
Sweden	Incidence	Female	2017	47.68705	51.07168	42.59332	
Sweden	Incidence	Female	2018	46.48294	49.77357	41.25642	
Sweden	Incidence	Female	2019	45.39964	48.82515	40.27651	
Sweden	Incidence	Female	2020	44.6001	48.09009	40.10743	

Sweden	Incidence	Female	2021	43.94291	47.17988	39.25223	
Sweden	Incidence	Female	2022	44.74222	48.26559	39.80195	
Sweden	Incidence	Female	2023	45.37478	49.60641	39.986	
Sweden	Deaths	Male	2010	46.19385	48.37415	43.31669	
Sweden	Deaths	Male	2011	46.34747	48.75421	43.27728	
Sweden	Deaths	Male	2012	45.35166	47.80592	42.72738	
Sweden	Deaths	Male	2013	43.83376	46.44173	41.20666	
Sweden	Deaths	Male	2014	43.39053	46.10119	40.879	
Sweden	Deaths	Male	2015	42.8143	45.4487	40.27195	
Sweden	Deaths	Male	2016	42.92269	45.76416	40.08674	
Sweden	Deaths	Male	2017	42.55278	45.6738	39.73143	
Sweden	Deaths	Male	2018	41.13784	43.93497	38.20156	
Sweden	Deaths	Male	2019	39.45446	42.38148	36.71357	
Sweden	Deaths	Male	2020	38.98093	41.87886	36.29542	
Sweden	Deaths	Male	2021	37.04512	40.10034	34.26556	
Sweden	Deaths	Male	2022	36.85937	40.26361	33.78296	
Sweden	Deaths	Male	2023	37.79433	41.63164	34.40036	
Sweden	Deaths	Female	2010	41.28307	43.91948	37.06599	
Sweden	Deaths	Female	2011	41.94145	44.77521	37.77978	
Sweden	Deaths	Female	2012	42.59969	45.34526	38.32995	
Sweden	Deaths	Female	2013	43.43651	46.25187	38.80425	
Sweden	Deaths	Female	2014	43.99334	46.99595	39.22846	
Sweden	Deaths	Female	2015	43.71156	46.95868	38.86998	
Sweden	Deaths	Female	2016	43.95804	47.29106	39.29258	
Sweden	Deaths	Female	2017	45.06964	48.27956	40.31548	
Sweden	Deaths	Female	2018	44.2781	47.67845	39.12765	
Sweden	Deaths	Female	2019	43.53282	46.68579	38.2904	
Sweden	Deaths	Female	2020	43.03507	46.02572	38.45572	
Sweden	Deaths	Female	2021	42.45704	45.68603	37.40989	
Sweden	Deaths	Female	2022	43.8495	47.2226	38.50346	
Sweden	Deaths	Female	2023	44.5644	48.13869	38.65235	
Sweden	DALYs	Male	2010	942.2036	987.6864	893.038	
Sweden	DALYs	Male	2011	940.1052	987.985	888.1752	
Sweden	DALYs	Male	2012	912.1487	958.3413	866.2598	
Sweden	DALYs	Male	2013	875.8588	930.8981	832.4158	
Sweden	DALYs	Male	2014	862.7888	916.2562	818.6737	

Sweden	DALYs	Male	2015	842.04	894.1416	798.0253	
Sweden	DALYs	Male	2016	832.6556	886.5965	783.453	
Sweden	DALYs	Male	2017	815.0855	871.8664	764.0187	
Sweden	DALYs	Male	2018	781.7716	833.3308	733.3298	
Sweden	DALYs	Male	2019	742.3037	799.3808	694.4164	
Sweden	DALYs	Male	2020	723.9868	781.1666	676.1611	
Sweden	DALYs	Male	2021	678.7878	737.1137	630.1336	
Sweden	DALYs	Male	2022	662.9121	724.5864	613.065	
Sweden	DALYs	Male	2023	679.3398	745.2438	624.0997	
Sweden	DALYs	Female	2010	851.3242	899.04	783.152	
Sweden	DALYs	Female	2011	849.0771	900.7325	777.8385	
Sweden	DALYs	Female	2012	853.3628	898.3555	781.0963	
Sweden	DALYs	Female	2013	870.0118	920.0319	802.7166	
Sweden	DALYs	Female	2014	876.6405	925.5957	810.6996	
Sweden	DALYs	Female	2015	860.6152	915.5151	786.284	
Sweden	DALYs	Female	2016	861.2841	917.2941	786.614	
Sweden	DALYs	Female	2017	869.3222	924.4605	787.3938	
Sweden	DALYs	Female	2018	845.6369	901.6263	762.3684	
Sweden	DALYs	Female	2019	819.9762	871.9039	737.2073	
Sweden	DALYs	Female	2020	796.8952	847.2313	727.2722	
Sweden	DALYs	Female	2021	783.1505	833.2698	709.4913	
Sweden	DALYs	Female	2022	790.2569	843.441	714.458	
Sweden	DALYs	Female	2023	803.1484	859.743	716.6372	
Switzerland	Incidence	Male	2010	68.83653	74.95667	63.48998	
Switzerland	Incidence	Male	2011	69.08016	75.64046	63.86737	
Switzerland	Incidence	Male	2012	67.25766	73.97885	62.14874	
Switzerland	Incidence	Male	2013	67.34116	73.74126	62.03435	
Switzerland	Incidence	Male	2014	67.79758	74.23324	62.34753	
Switzerland	Incidence	Male	2015	66.89812	73.15463	61.63272	
Switzerland	Incidence	Male	2016	65.07766	71.51096	59.53439	
Switzerland	Incidence	Male	2017	64.32289	70.95803	58.46541	
Switzerland	Incidence	Male	2018	64.23491	70.84582	58.03725	
Switzerland	Incidence	Male	2019	61.43551	67.722	55.15107	
Switzerland	Incidence	Male	2020	60.36274	66.82618	54.47547	
Switzerland	Incidence	Male	2021	61.11632	68.31565	54.35048	
Switzerland	Incidence	Male	2022	65.07365	72.54668	58.15752	

Switzerland	Incidence	Male	2023	60.75447	68.46478	53.61518	
Switzerland	Incidence	Female	2010	38.08971	43.0343	33.89777	
Switzerland	Incidence	Female	2011	39.66467	44.60927	34.77592	
Switzerland	Incidence	Female	2012	40.64892	45.78948	35.56086	
Switzerland	Incidence	Female	2013	41.51447	47.27461	35.87194	
Switzerland	Incidence	Female	2014	41.6587	47.38704	35.77579	
Switzerland	Incidence	Female	2015	41.30916	47.20839	35.26058	
Switzerland	Incidence	Female	2016	42.88037	48.7242	36.14635	
Switzerland	Incidence	Female	2017	43.63826	49.4563	36.96594	
Switzerland	Incidence	Female	2018	44.01566	50.29161	37.00028	
Switzerland	Incidence	Female	2019	44.56956	51.31753	37.33311	
Switzerland	Incidence	Female	2020	43.43681	49.74786	36.63535	
Switzerland	Incidence	Female	2021	42.86693	49.25878	35.75775	
Switzerland	Incidence	Female	2022	46.04626	53.60033	38.09926	
Switzerland	Incidence	Female	2023	42.18863	49.11533	34.7569	
Switzerland	Deaths	Male	2010	59.26916	62.36308	55.71561	
Switzerland	Deaths	Male	2011	59.34952	62.4521	55.71634	
Switzerland	Deaths	Male	2012	57.79334	60.43673	54.67032	
Switzerland	Deaths	Male	2013	57.74023	60.41048	54.51248	
Switzerland	Deaths	Male	2014	57.99649	61.07184	54.44902	
Switzerland	Deaths	Male	2015	57.25106	60.73041	53.75171	
Switzerland	Deaths	Male	2016	55.71707	59.05878	52.36335	
Switzerland	Deaths	Male	2017	55.00488	58.48988	51.34678	
Switzerland	Deaths	Male	2018	54.79444	58.56406	50.88529	
Switzerland	Deaths	Male	2019	52.45387	55.79762	48.95483	
Switzerland	Deaths	Male	2020	51.43717	54.60121	47.72622	
Switzerland	Deaths	Male	2021	51.70523	55.1714	47.70728	
Switzerland	Deaths	Male	2022	55.43879	59.55217	51.06448	
Switzerland	Deaths	Male	2023	52.11384	57.03499	47.20757	
Switzerland	Deaths	Female	2010	31.03099	33.64549	27.45123	
Switzerland	Deaths	Female	2011	32.23563	35.04146	28.38267	
Switzerland	Deaths	Female	2012	33.02566	36.06753	29.15602	
Switzerland	Deaths	Female	2013	33.75928	36.97969	29.51036	
Switzerland	Deaths	Female	2014	33.74477	37.18301	29.32274	
Switzerland	Deaths	Female	2015	33.614	36.70531	29.21514	
Switzerland	Deaths	Female	2016	34.81502	37.83104	29.90527	

Switzerland	Deaths	Female	2017	35.45778	38.70172	30.78374	
Switzerland	Deaths	Female	2018	35.84528	39.24516	30.97392	
Switzerland	Deaths	Female	2019	36.25361	39.994	31.03702	
Switzerland	Deaths	Female	2020	35.42045	38.7271	30.87797	
Switzerland	Deaths	Female	2021	34.69786	38.19585	30.34077	
Switzerland	Deaths	Female	2022	37.49023	41.77173	32.12732	
Switzerland	Deaths	Female	2023	34.5762	38.55514	29.2075	
Switzerland	DALYs	Male	2010	1308.918	1366.249	1244.021	
Switzerland	DALYs	Male	2011	1302.337	1354.807	1239.641	
Switzerland	DALYs	Male	2012	1259.697	1311.925	1207.506	
Switzerland	DALYs	Male	2013	1253.282	1298.033	1198.455	
Switzerland	DALYs	Male	2014	1252.424	1306.286	1194.836	
Switzerland	DALYs	Male	2015	1227.229	1288.904	1166.681	
Switzerland	DALYs	Male	2016	1183.455	1243.959	1127.283	
Switzerland	DALYs	Male	2017	1163.236	1224.61	1098.669	
Switzerland	DALYs	Male	2018	1153.048	1216.975	1083.329	
Switzerland	DALYs	Male	2019	1087.985	1150.116	1020.453	
Switzerland	DALYs	Male	2020	1064.78	1127.896	994.4135	
Switzerland	DALYs	Male	2021	1076.83	1145.808	1004.447	
Switzerland	DALYs	Male	2022	1135.25	1213.045	1054.298	
Switzerland	DALYs	Male	2023	1051.662	1141.742	958.1581	
Switzerland	DALYs	Female	2010	694.0546	744.2395	633.7034	
Switzerland	DALYs	Female	2011	716.5724	769.9572	649.7725	
Switzerland	DALYs	Female	2012	724.646	786.267	653.9209	
Switzerland	DALYs	Female	2013	729.9826	793.8586	655.7688	
Switzerland	DALYs	Female	2014	725.9516	792.8664	649.0071	
Switzerland	DALYs	Female	2015	711.6597	769.9309	638.8534	
Switzerland	DALYs	Female	2016	734.8344	789.3201	652.9984	
Switzerland	DALYs	Female	2017	740.6367	797.5909	657.0419	
Switzerland	DALYs	Female	2018	736.031	796.0895	651.4901	
Switzerland	DALYs	Female	2019	737.356	801.9317	641.5595	
Switzerland	DALYs	Female	2020	708.2444	769.0844	629.0674	
Switzerland	DALYs	Female	2021	699.2921	769.3211	627.2444	
Switzerland	DALYs	Female	2022	744.2961	824.7543	657.5938	
Switzerland	DALYs	Female	2023	678.3818	755.0835	597.0613	
Syrian Arab Rep	Incidence	Male	2010	9.101995	10.95391	7.596877	

Syrian Arab Rep	Incidence	Male	2011	9.009545	10.70532	7.415141	
Syrian Arab Rep	Incidence	Male	2012	8.928283	10.70381	7.115633	
Syrian Arab Rep	Incidence	Male	2013	9.014165	11.21511	6.994294	
Syrian Arab Rep	Incidence	Male	2014	9.550941	12.21752	7.278654	
Syrian Arab Rep	Incidence	Male	2015	10.40243	13.78623	7.710455	
Syrian Arab Rep	Incidence	Male	2016	11.3676	15.6485	8.327711	
Syrian Arab Rep	Incidence	Male	2017	12.34969	17.0335	9.058002	
Syrian Arab Rep	Incidence	Male	2018	13.25074	17.9701	9.78749	
Syrian Arab Rep	Incidence	Male	2019	14.23462	19.06049	10.51332	
Syrian Arab Rep	Incidence	Male	2020	14.06282	18.93909	10.59495	
Syrian Arab Rep	Incidence	Male	2021	14.02584	18.83026	10.88163	
Syrian Arab Rep	Incidence	Male	2022	15.84542	21.86933	11.81707	
Syrian Arab Rep	Incidence	Male	2023	16.31422	22.66551	12.0322	
Syrian Arab Rep	Incidence	Female	2010	3.093202	3.766917	2.46543	
Syrian Arab Rep	Incidence	Female	2011	3.134011	3.846893	2.539621	
Syrian Arab Rep	Incidence	Female	2012	3.10296	3.888245	2.523318	
Syrian Arab Rep	Incidence	Female	2013	3.095584	3.947176	2.512574	
Syrian Arab Rep	Incidence	Female	2014	3.214792	4.11617	2.541337	
Syrian Arab Rep	Incidence	Female	2015	3.430578	4.437507	2.667615	
Syrian Arab Rep	Incidence	Female	2016	3.704978	4.918449	2.907341	
Syrian Arab Rep	Incidence	Female	2017	4.014662	5.383768	2.994036	
Syrian Arab Rep	Incidence	Female	2018	4.28665	5.779676	3.150286	
Syrian Arab Rep	Incidence	Female	2019	4.614562	6.287911	3.344198	
Syrian Arab Rep	Incidence	Female	2020	4.626468	6.405209	3.41068	
Syrian Arab Rep	Incidence	Female	2021	4.544011	6.112486	3.346814	
Syrian Arab Rep	Incidence	Female	2022	5.054191	6.845042	3.577866	
Syrian Arab Rep	Incidence	Female	2023	5.193342	7.126627	3.668793	
Syrian Arab Rep	Deaths	Male	2010	8.686364	10.41852	7.321534	
Syrian Arab Rep	Deaths	Male	2011	8.5991	10.25169	7.068449	
Syrian Arab Rep	Deaths	Male	2012	8.537305	10.25466	6.768573	
Syrian Arab Rep	Deaths	Male	2013	8.643523	10.78789	6.701526	
Syrian Arab Rep	Deaths	Male	2014	9.189724	11.775	6.982511	
Syrian Arab Rep	Deaths	Male	2015	10.03839	13.31236	7.452435	
Syrian Arab Rep	Deaths	Male	2016	10.98196	15.14888	8.097916	
Syrian Arab Rep	Deaths	Male	2017	11.92724	16.56172	8.765966	
Syrian Arab Rep	Deaths	Male	2018	12.78402	17.33912	9.350244	

Syrian Arab Rep	Deaths	Male	2019	13.7113	18.40704	10.00288	
Syrian Arab Rep	Deaths	Male	2020	13.55747	18.23174	10.08772	
Syrian Arab Rep	Deaths	Male	2021	13.52476	18.08344	10.34945	
Syrian Arab Rep	Deaths	Male	2022	15.22168	20.79707	11.37532	
Syrian Arab Rep	Deaths	Male	2023	15.66932	21.47444	11.43981	
Syrian Arab Rep	Deaths	Female	2010	2.893642	3.497658	2.318852	
Syrian Arab Rep	Deaths	Female	2011	2.935216	3.582697	2.380629	
Syrian Arab Rep	Deaths	Female	2012	2.916109	3.616865	2.374212	
Syrian Arab Rep	Deaths	Female	2013	2.921557	3.662222	2.375599	
Syrian Arab Rep	Deaths	Female	2014	3.049981	3.891378	2.408949	
Syrian Arab Rep	Deaths	Female	2015	3.271205	4.213924	2.5574	
Syrian Arab Rep	Deaths	Female	2016	3.542831	4.656989	2.80627	
Syrian Arab Rep	Deaths	Female	2017	3.841882	5.137068	2.878961	
Syrian Arab Rep	Deaths	Female	2018	4.101406	5.576051	3.04488	
Syrian Arab Rep	Deaths	Female	2019	4.411028	6.053647	3.257451	
Syrian Arab Rep	Deaths	Female	2020	4.435304	6.063906	3.308346	
Syrian Arab Rep	Deaths	Female	2021	4.367152	5.814765	3.223922	
Syrian Arab Rep	Deaths	Female	2022	4.834954	6.565535	3.462563	
Syrian Arab Rep	Deaths	Female	2023	4.973667	6.810366	3.535341	
Syrian Arab Rep	DALYs	Male	2010	246.4053	296.5995	202.2205	
Syrian Arab Rep	DALYs	Male	2011	243.4434	289.2597	198.8723	
Syrian Arab Rep	DALYs	Male	2012	241.0085	291.7068	192.4846	
Syrian Arab Rep	DALYs	Male	2013	242.8667	302.3179	189.778	
Syrian Arab Rep	DALYs	Male	2014	255.9199	331.426	195.9688	
Syrian Arab Rep	DALYs	Male	2015	276.3438	366.7566	204.8294	
Syrian Arab Rep	DALYs	Male	2016	299.3412	408.0813	220.4635	
Syrian Arab Rep	DALYs	Male	2017	322.5159	443.9411	238.3631	
Syrian Arab Rep	DALYs	Male	2018	343.2138	463.5779	252.0977	
Syrian Arab Rep	DALYs	Male	2019	365.8896	493.0627	267.8274	
Syrian Arab Rep	DALYs	Male	2020	358.7153	484.2511	265.331	
Syrian Arab Rep	DALYs	Male	2021	355.5414	473.5651	271.1023	
Syrian Arab Rep	DALYs	Male	2022	401.7859	550.4665	298.2994	
Syrian Arab Rep	DALYs	Male	2023	411.7052	569.675	299.7398	
Syrian Arab Rep	DALYs	Female	2010	86.07843	105.4923	69.97727	
Syrian Arab Rep	DALYs	Female	2011	86.73198	106.226	70.51718	
Syrian Arab Rep	DALYs	Female	2012	85.53219	105.8686	70.34651	

Syrian Arab Rep	DALYs	Female	2013	84.98359	107.5222	69.42968	
Syrian Arab Rep	DALYs	Female	2014	87.503	111.6953	69.82078	
Syrian Arab Rep	DALYs	Female	2015	92.16753	118.8218	73.74145	
Syrian Arab Rep	DALYs	Female	2016	98.22899	127.2281	77.09808	
Syrian Arab Rep	DALYs	Female	2017	105.1359	140.7085	79.61296	
Syrian Arab Rep	DALYs	Female	2018	110.939	151.6997	81.87862	
Syrian Arab Rep	DALYs	Female	2019	118.1133	163.6274	86.27171	
Syrian Arab Rep	DALYs	Female	2020	117.0948	162.9161	86.52661	
Syrian Arab Rep	DALYs	Female	2021	113.9132	153.6837	83.96619	
Syrian Arab Rep	DALYs	Female	2022	126.6427	172.9766	89.70521	
Syrian Arab Rep	DALYs	Female	2023	129.2134	179.8549	91.52695	
Türkiye	Incidence	Male	2010	49.55017	58.24394	32.48377	
Türkiye	Incidence	Male	2011	52.45873	60.75908	33.5706	
Türkiye	Incidence	Male	2012	54.23024	63.03306	34.10231	
Türkiye	Incidence	Male	2013	57.25807	66.47309	36.43806	
Türkiye	Incidence	Male	2014	58.80802	67.70345	37.58642	
Türkiye	Incidence	Male	2015	59.9569	69.20952	38.95183	
Türkiye	Incidence	Male	2016	61.29919	70.25257	40.99367	
Türkiye	Incidence	Male	2017	61.51913	71.74541	41.3037	
Türkiye	Incidence	Male	2018	61.32875	72.22545	40.73451	
Türkiye	Incidence	Male	2019	62.61964	74.86787	40.82033	
Türkiye	Incidence	Male	2020	66.25377	79.60441	42.56732	
Türkiye	Incidence	Male	2021	69.19439	85.01901	44.51083	
Türkiye	Incidence	Male	2022	68.50417	85.05123	43.49241	
Türkiye	Incidence	Male	2023	68.4737	87.5303	45.55778	
Türkiye	Incidence	Female	2010	8.881919	10.38398	7.323177	
Türkiye	Incidence	Female	2011	9.441374	10.8628	7.689136	
Türkiye	Incidence	Female	2012	9.88199	11.43924	8.235771	
Türkiye	Incidence	Female	2013	10.51196	12.20517	8.92793	
Türkiye	Incidence	Female	2014	11.12063	12.94314	9.529653	
Türkiye	Incidence	Female	2015	11.50121	13.39643	9.941939	
Türkiye	Incidence	Female	2016	11.80304	13.72017	10.0985	
Türkiye	Incidence	Female	2017	11.91494	13.84479	9.853528	
Türkiye	Incidence	Female	2018	11.92109	13.85632	9.657001	
Türkiye	Incidence	Female	2019	12.40042	14.72486	9.880628	
Türkiye	Incidence	Female	2020	13.06491	15.84416	10.41528	

Türkiye	Incidence	Female	2021	14.3956	17.99873	11.35598	
Türkiye	Incidence	Female	2022	14.27409	18.4619	11.06805	
Türkiye	Incidence	Female	2023	14.22419	18.81727	10.79886	
Türkiye	Deaths	Male	2010	47.50286	55.72413	31.54836	
Türkiye	Deaths	Male	2011	50.35886	58.35059	32.56566	
Türkiye	Deaths	Male	2012	52.07205	60.25421	33.35662	
Türkiye	Deaths	Male	2013	55.08527	64.09718	35.63166	
Türkiye	Deaths	Male	2014	56.70989	65.23888	36.84064	
Türkiye	Deaths	Male	2015	57.86931	66.62926	37.70661	
Türkiye	Deaths	Male	2016	59.0981	68.02606	39.47543	
Türkiye	Deaths	Male	2017	59.16805	68.40209	39.54883	
Türkiye	Deaths	Male	2018	58.85143	68.87411	38.84664	
Türkiye	Deaths	Male	2019	59.98507	71.24381	38.91822	
Türkiye	Deaths	Male	2020	63.45065	76.16537	40.93476	
Türkiye	Deaths	Male	2021	65.83878	80.88865	42.38342	
Türkiye	Deaths	Male	2022	65.0406	79.61431	41.06682	
Türkiye	Deaths	Male	2023	64.95013	81.61108	42.78482	
Türkiye	Deaths	Female	2010	8.630008	10.18965	7.190429	
Türkiye	Deaths	Female	2011	9.194406	10.58734	7.542372	
Türkiye	Deaths	Female	2012	9.627289	11.06557	8.109894	
Türkiye	Deaths	Female	2013	10.26722	11.88392	8.798454	
Türkiye	Deaths	Female	2014	10.88251	12.58743	9.243095	
Türkiye	Deaths	Female	2015	11.23656	13.03081	9.778899	
Türkiye	Deaths	Female	2016	11.50748	13.25214	9.827865	
Türkiye	Deaths	Female	2017	11.58475	13.39165	9.72451	
Türkiye	Deaths	Female	2018	11.55321	13.39459	9.477966	
Türkiye	Deaths	Female	2019	11.96904	14.17235	9.513571	
Türkiye	Deaths	Female	2020	12.58595	15.21989	9.950236	
Türkiye	Deaths	Female	2021	13.75435	17.23665	10.9001	
Türkiye	Deaths	Female	2022	13.61521	17.57589	10.53693	
Türkiye	Deaths	Female	2023	13.54991	17.90529	10.18869	
Türkiye	DALYs	Male	2010	1284.789	1508.394	841.9772	
Türkiye	DALYs	Male	2011	1346.168	1558.927	856.502	
Türkiye	DALYs	Male	2012	1383.366	1603.15	867.4956	
Türkiye	DALYs	Male	2013	1450.338	1694.084	923.1541	
Türkiye	DALYs	Male	2014	1479.568	1705.897	948.3662	

Türkiye	DALYs	Male	2015	1497.108	1731.049	970.5296	
Türkiye	DALYs	Male	2016	1519.561	1761.062	1010.812	
Türkiye	DALYs	Male	2017	1516.466	1766.631	1010.062	
Türkiye	DALYs	Male	2018	1503.212	1765.463	997.639	
Türkiye	DALYs	Male	2019	1523.652	1800.429	992.1185	
Türkiye	DALYs	Male	2020	1592.596	1933.471	1027.262	
Türkiye	DALYs	Male	2021	1661.074	2062.413	1075.716	
Türkiye	DALYs	Male	2022	1632.731	2036.797	1040.78	
Türkiye	DALYs	Male	2023	1620.678	2068.718	1084.291	
Türkiye	DALYs	Female	2010	220.4655	256.27	182.9716	
Türkiye	DALYs	Female	2011	231.6442	264.8427	188.5611	
Türkiye	DALYs	Female	2012	241.0907	275.5109	200.2557	
Türkiye	DALYs	Female	2013	254.5245	292.6333	215.1054	
Türkiye	DALYs	Female	2014	267.6268	306.0721	226.5166	
Türkiye	DALYs	Female	2015	275.6597	318.4751	235.2903	
Türkiye	DALYs	Female	2016	281.1761	322.1644	239.0204	
Türkiye	DALYs	Female	2017	282.1005	325.6181	235.6365	
Türkiye	DALYs	Female	2018	280.6469	324.3737	229.6931	
Türkiye	DALYs	Female	2019	289.7671	340.6167	231.3913	
Türkiye	DALYs	Female	2020	301.0348	364.7195	239.3927	
Türkiye	DALYs	Female	2021	331.0579	412.9093	263.5934	
Türkiye	DALYs	Female	2022	325.3302	417.2404	257.2386	
Türkiye	DALYs	Female	2023	321.5969	418.7179	244.0254	
Tajikistan	Incidence	Male	2010	5.19836	7.899155	4.065135	
Tajikistan	Incidence	Male	2011	5.085314	7.910855	3.966614	
Tajikistan	Incidence	Male	2012	4.861012	7.879468	3.722956	
Tajikistan	Incidence	Male	2013	4.711249	7.93815	3.605365	
Tajikistan	Incidence	Male	2014	4.676995	8.10961	3.583406	
Tajikistan	Incidence	Male	2015	4.673469	8.473356	3.568386	
Tajikistan	Incidence	Male	2016	4.819765	9.132109	3.683016	
Tajikistan	Incidence	Male	2017	4.927153	9.483748	3.734262	
Tajikistan	Incidence	Male	2018	4.945257	9.494295	3.679847	
Tajikistan	Incidence	Male	2019	5.240253	10.06798	3.837039	
Tajikistan	Incidence	Male	2020	4.487699	8.265654	3.364736	
Tajikistan	Incidence	Male	2021	4.900857	9.253045	3.608764	
Tajikistan	Incidence	Male	2022	6.050234	10.51936	4.368305	

Tajikistan	Incidence	Male	2023	6.473935	11.16966	4.671847	
Tajikistan	Incidence	Female	2010	2.347606	3.291221	1.895296	
Tajikistan	Incidence	Female	2011	2.333765	3.275084	1.885623	
Tajikistan	Incidence	Female	2012	2.32705	3.339787	1.901324	
Tajikistan	Incidence	Female	2013	2.317309	3.339487	1.908314	
Tajikistan	Incidence	Female	2014	2.380238	3.450225	1.957848	
Tajikistan	Incidence	Female	2015	2.478403	3.657278	2.044819	
Tajikistan	Incidence	Female	2016	2.597401	3.95759	2.116133	
Tajikistan	Incidence	Female	2017	2.740102	4.208545	2.19305	
Tajikistan	Incidence	Female	2018	2.823165	4.366043	2.285028	
Tajikistan	Incidence	Female	2019	3.050247	4.66973	2.421152	
Tajikistan	Incidence	Female	2020	3.002467	4.510392	2.387641	
Tajikistan	Incidence	Female	2021	3.347793	5.264941	2.603862	
Tajikistan	Incidence	Female	2022	4.05524	6.15887	3.073049	
Tajikistan	Incidence	Female	2023	4.240823	6.557207	3.080427	
Tajikistan	Deaths	Male	2010	5.099194	7.68254	3.9602	
Tajikistan	Deaths	Male	2011	4.987122	7.757845	3.867354	
Tajikistan	Deaths	Male	2012	4.765847	7.76986	3.648617	
Tajikistan	Deaths	Male	2013	4.620986	7.745707	3.531027	
Tajikistan	Deaths	Male	2014	4.586079	7.898984	3.499391	
Tajikistan	Deaths	Male	2015	4.582841	8.216132	3.500858	
Tajikistan	Deaths	Male	2016	4.728144	8.824798	3.613737	
Tajikistan	Deaths	Male	2017	4.826218	9.268723	3.652077	
Tajikistan	Deaths	Male	2018	4.834196	9.260144	3.592161	
Tajikistan	Deaths	Male	2019	5.115121	9.838333	3.745053	
Tajikistan	Deaths	Male	2020	4.411125	7.996778	3.328084	
Tajikistan	Deaths	Male	2021	4.818193	8.956683	3.557623	
Tajikistan	Deaths	Male	2022	5.923186	10.29462	4.291249	
Tajikistan	Deaths	Male	2023	6.324316	10.95273	4.570668	
Tajikistan	Deaths	Female	2010	2.296211	3.224	1.844208	
Tajikistan	Deaths	Female	2011	2.279507	3.212036	1.831767	
Tajikistan	Deaths	Female	2012	2.268169	3.271216	1.839611	
Tajikistan	Deaths	Female	2013	2.257423	3.259397	1.848211	
Tajikistan	Deaths	Female	2014	2.3139	3.360353	1.894418	
Tajikistan	Deaths	Female	2015	2.406097	3.553257	1.983792	
Tajikistan	Deaths	Female	2016	2.520011	3.835456	2.048183	

Tajikistan	Deaths	Female	2017	2.649854	4.069035	2.121264	
Tajikistan	Deaths	Female	2018	2.722726	4.217075	2.185488	
Tajikistan	Deaths	Female	2019	2.937805	4.502717	2.328161	
Tajikistan	Deaths	Female	2020	2.904996	4.365198	2.297385	
Tajikistan	Deaths	Female	2021	3.242531	5.123794	2.498247	
Tajikistan	Deaths	Female	2022	3.910552	6.010706	2.957451	
Tajikistan	Deaths	Female	2023	4.07369	6.341679	2.963381	
Tajikistan	DALYs	Male	2010	147.1134	221.9817	116.4408	
Tajikistan	DALYs	Male	2011	144.1266	223.1212	114.1695	
Tajikistan	DALYs	Male	2012	137.7411	221.7437	106.3346	
Tajikistan	DALYs	Male	2013	133.2304	223.8351	103.0881	
Tajikistan	DALYs	Male	2014	132.1079	228.7365	102.5027	
Tajikistan	DALYs	Male	2015	131.7631	243.1327	101.9815	
Tajikistan	DALYs	Male	2016	135.5391	260.7011	103.6747	
Tajikistan	DALYs	Male	2017	138.5548	265.0163	106.2426	
Tajikistan	DALYs	Male	2018	139.1754	266.3223	105.2811	
Tajikistan	DALYs	Male	2019	147.1661	278.7622	109.3228	
Tajikistan	DALYs	Male	2020	123.6441	229.422	93.69661	
Tajikistan	DALYs	Male	2021	134.1293	247.211	98.96323	
Tajikistan	DALYs	Male	2022	166.0351	288.4544	119.5223	
Tajikistan	DALYs	Male	2023	177.9125	302.9797	130.0705	
Tajikistan	DALYs	Female	2010	68.51339	94.50857	55.79395	
Tajikistan	DALYs	Female	2011	68.32646	94.42875	56.43946	
Tajikistan	DALYs	Female	2012	68.22223	96.02854	56.73219	
Tajikistan	DALYs	Female	2013	67.88666	96.67145	55.88589	
Tajikistan	DALYs	Female	2014	69.75889	99.86039	58.18473	
Tajikistan	DALYs	Female	2015	72.56491	105.8733	60.53091	
Tajikistan	DALYs	Female	2016	75.87643	114.7299	62.57793	
Tajikistan	DALYs	Female	2017	80.18985	121.8814	64.73671	
Tajikistan	DALYs	Female	2018	82.76831	126.0764	67.58891	
Tajikistan	DALYs	Female	2019	89.17804	134.295	71.41318	
Tajikistan	DALYs	Female	2020	86.29162	128.2132	68.86927	
Tajikistan	DALYs	Female	2021	95.32407	147.0656	75.28649	
Tajikistan	DALYs	Female	2022	115.8249	171.9606	87.9063	
Tajikistan	DALYs	Female	2023	121.8123	185.6523	87.50322	
Thailand	Incidence	Male	2010	41.5733	50.67885	34.10828	

Thailand	Incidence	Male	2011	42.6531	52.65149	35.38019	
Thailand	Incidence	Male	2012	44.25265	55.7435	37.56543	
Thailand	Incidence	Male	2013	45.1029	58.30169	38.33872	
Thailand	Incidence	Male	2014	44.55191	59.11648	38.11529	
Thailand	Incidence	Male	2015	45.82176	62.94742	39.48507	
Thailand	Incidence	Male	2016	47.25294	66.03122	40.19388	
Thailand	Incidence	Male	2017	45.73049	64.9329	38.59904	
Thailand	Incidence	Male	2018	45.74626	66.07814	38.09867	
Thailand	Incidence	Male	2019	48.47795	69.57924	39.4201	
Thailand	Incidence	Male	2020	50.5858	72.14269	40.69225	
Thailand	Incidence	Male	2021	53.45811	76.43475	42.54214	
Thailand	Incidence	Male	2022	58.80287	82.63178	46.62404	
Thailand	Incidence	Male	2023	58.40699	82.36635	44.67535	
Thailand	Incidence	Female	2010	21.18367	26.36306	17.07866	
Thailand	Incidence	Female	2011	21.39974	26.53825	17.4532	
Thailand	Incidence	Female	2012	21.62552	26.40274	17.84338	
Thailand	Incidence	Female	2013	21.91744	26.33843	18.41845	
Thailand	Incidence	Female	2014	22.01057	27.19296	18.3567	
Thailand	Incidence	Female	2015	22.4438	28.16264	18.74696	
Thailand	Incidence	Female	2016	22.95208	29.2872	19.15753	
Thailand	Incidence	Female	2017	22.28498	28.51438	18.33301	
Thailand	Incidence	Female	2018	22.31203	28.81444	18.58865	
Thailand	Incidence	Female	2019	23.49437	30.50665	19.50981	
Thailand	Incidence	Female	2020	24.01213	32.02935	19.97507	
Thailand	Incidence	Female	2021	24.93526	33.77084	20.29053	
Thailand	Incidence	Female	2022	27.37336	37.19279	21.83604	
Thailand	Incidence	Female	2023	27.59306	36.95863	22.0028	
Thailand	Deaths	Male	2010	41.79089	50.40929	34.06665	
Thailand	Deaths	Male	2011	42.8152	52.40617	34.85408	
Thailand	Deaths	Male	2012	44.33647	55.58331	37.37125	
Thailand	Deaths	Male	2013	45.1611	58.28773	38.23345	
Thailand	Deaths	Male	2014	44.5752	59.20994	38.10093	
Thailand	Deaths	Male	2015	45.82243	62.75771	39.51583	
Thailand	Deaths	Male	2016	47.20465	65.73771	40.1214	
Thailand	Deaths	Male	2017	45.54202	64.58332	38.86993	
Thailand	Deaths	Male	2018	45.43738	65.75918	38.3089	

Thailand	Deaths	Male	2019	48.1113	68.98263	39.4976	
Thailand	Deaths	Male	2020	50.10857	71.36461	40.64913	
Thailand	Deaths	Male	2021	52.72133	75.71553	42.2721	
Thailand	Deaths	Male	2022	58.29638	82.31094	45.96924	
Thailand	Deaths	Male	2023	57.92054	82.10541	44.92989	
Thailand	Deaths	Female	2010	21.04022	26.25974	17.03041	
Thailand	Deaths	Female	2011	21.26743	26.33721	17.35641	
Thailand	Deaths	Female	2012	21.44044	26.46424	17.62358	
Thailand	Deaths	Female	2013	21.69359	26.22341	18.18933	
Thailand	Deaths	Female	2014	21.77695	26.82543	18.25463	
Thailand	Deaths	Female	2015	22.18711	27.60555	18.56816	
Thailand	Deaths	Female	2016	22.63631	28.65196	18.94157	
Thailand	Deaths	Female	2017	21.87167	27.72712	18.05016	
Thailand	Deaths	Female	2018	21.82531	27.9688	18.27672	
Thailand	Deaths	Female	2019	22.9696	29.6059	19.28284	
Thailand	Deaths	Female	2020	23.41811	30.73335	19.4056	
Thailand	Deaths	Female	2021	24.27592	32.32231	20.03134	
Thailand	Deaths	Female	2022	26.80771	35.69132	21.63795	
Thailand	Deaths	Female	2023	26.95669	35.20378	21.38438	
Thailand	DALYs	Male	2010	1038.693	1288.172	849.7819	
Thailand	DALYs	Male	2011	1065.989	1338.807	900.232	
Thailand	DALYs	Male	2012	1105.783	1419.577	937.03	
Thailand	DALYs	Male	2013	1124.744	1475.567	968.1221	
Thailand	DALYs	Male	2014	1110.718	1494.269	956.4253	
Thailand	DALYs	Male	2015	1140.78	1574.535	988.6587	
Thailand	DALYs	Male	2016	1173.276	1639.88	999.6038	
Thailand	DALYs	Male	2017	1136.866	1625.334	968.0436	
Thailand	DALYs	Male	2018	1138.348	1641.768	953.783	
Thailand	DALYs	Male	2019	1202.124	1723.399	978.5943	
Thailand	DALYs	Male	2020	1252.133	1794.596	1009.224	
Thailand	DALYs	Male	2021	1327.799	1896.973	1041.147	
Thailand	DALYs	Male	2022	1441.092	2026.696	1123.67	
Thailand	DALYs	Male	2023	1427.394	2016.986	1097.169	
Thailand	DALYs	Female	2010	507.5636	630.565	415.7108	
Thailand	DALYs	Female	2011	509.7649	631.4978	422.1482	
Thailand	DALYs	Female	2012	513.9371	624.3502	427.8391	

Thailand	DALYs	Female	2013	519.0145	637.9222	442.826	
Thailand	DALYs	Female	2014	519.5221	650.7056	436.6323	
Thailand	DALYs	Female	2015	528.5848	670.2192	445.3639	
Thailand	DALYs	Female	2016	539.0215	691.2606	451.7608	
Thailand	DALYs	Female	2017	524.2262	674.9681	435.6569	
Thailand	DALYs	Female	2018	525.2035	683.4857	439.5003	
Thailand	DALYs	Female	2019	549.4348	714.3106	465.0604	
Thailand	DALYs	Female	2020	559.0814	741.4588	464.5834	
Thailand	DALYs	Female	2021	579.0529	776.3849	474.2592	
Thailand	DALYs	Female	2022	625.5106	837.2726	493.828	
Thailand	DALYs	Female	2023	630.8309	838.9282	490.1791	
Timor-Leste	Incidence	Male	2010	9.751105	16.66023	6.100802	
Timor-Leste	Incidence	Male	2011	10.23949	17.26566	6.514355	
Timor-Leste	Incidence	Male	2012	10.50814	17.35477	6.580122	
Timor-Leste	Incidence	Male	2013	10.43001	17.03167	6.709131	
Timor-Leste	Incidence	Male	2014	10.29954	17.05528	6.459802	
Timor-Leste	Incidence	Male	2015	10.8784	17.91775	6.738309	
Timor-Leste	Incidence	Male	2016	10.95672	18.149	6.826438	
Timor-Leste	Incidence	Male	2017	11.00417	18.40819	6.772203	
Timor-Leste	Incidence	Male	2018	11.02859	17.96401	6.846642	
Timor-Leste	Incidence	Male	2019	11.04477	18.04775	6.889555	
Timor-Leste	Incidence	Male	2020	11.59889	19.17366	7.307345	
Timor-Leste	Incidence	Male	2021	11.65815	19.71372	7.320868	
Timor-Leste	Incidence	Male	2022	11.85069	20.19595	7.48193	
Timor-Leste	Incidence	Male	2023	12.07175	20.74318	7.90359	
Timor-Leste	Incidence	Female	2010	4.346769	7.217114	2.564863	
Timor-Leste	Incidence	Female	2011	4.789572	7.831237	2.832142	
Timor-Leste	Incidence	Female	2012	5.051272	8.174424	2.987321	
Timor-Leste	Incidence	Female	2013	5.086734	8.226763	2.988351	
Timor-Leste	Incidence	Female	2014	5.08421	8.280472	3.112579	
Timor-Leste	Incidence	Female	2015	5.333626	8.643811	3.182023	
Timor-Leste	Incidence	Female	2016	5.488009	8.58064	3.282816	
Timor-Leste	Incidence	Female	2017	5.652894	8.520639	3.483675	
Timor-Leste	Incidence	Female	2018	5.744189	8.566285	3.625899	
Timor-Leste	Incidence	Female	2019	5.854878	8.641937	3.865668	
Timor-Leste	Incidence	Female	2020	6.221045	9.188844	4.190134	

Timor-Leste	Incidence	Female	2021	6.408085	9.37029	4.196728	
Timor-Leste	Incidence	Female	2022	6.683364	9.729898	4.157509	
Timor-Leste	Incidence	Female	2023	6.938426	10.36137	4.322755	
Timor-Leste	Deaths	Male	2010	9.923864	16.85643	6.176047	
Timor-Leste	Deaths	Male	2011	10.41636	17.40333	6.623908	
Timor-Leste	Deaths	Male	2012	10.68707	17.54662	6.676648	
Timor-Leste	Deaths	Male	2013	10.60736	17.24745	6.805464	
Timor-Leste	Deaths	Male	2014	10.47507	17.15475	6.605004	
Timor-Leste	Deaths	Male	2015	11.07587	18.21926	6.835338	
Timor-Leste	Deaths	Male	2016	11.15892	18.20717	6.961918	
Timor-Leste	Deaths	Male	2017	11.2138	18.5795	6.888607	
Timor-Leste	Deaths	Male	2018	11.24445	18.11639	6.951515	
Timor-Leste	Deaths	Male	2019	11.26241	18.17633	7.034978	
Timor-Leste	Deaths	Male	2020	11.87519	19.26311	7.429843	
Timor-Leste	Deaths	Male	2021	11.93652	20.1497	7.431961	
Timor-Leste	Deaths	Male	2022	12.12025	20.77798	7.663977	
Timor-Leste	Deaths	Male	2023	12.33998	21.06338	8.089604	
Timor-Leste	Deaths	Female	2010	4.291731	7.109866	2.540583	
Timor-Leste	Deaths	Female	2011	4.722878	7.695609	2.786594	
Timor-Leste	Deaths	Female	2012	4.977672	8.097529	2.959275	
Timor-Leste	Deaths	Female	2013	5.009669	8.131407	2.946223	
Timor-Leste	Deaths	Female	2014	5.00253	8.083351	3.085999	
Timor-Leste	Deaths	Female	2015	5.257008	8.475515	3.138121	
Timor-Leste	Deaths	Female	2016	5.407243	8.531285	3.239522	
Timor-Leste	Deaths	Female	2017	5.568238	8.429625	3.436857	
Timor-Leste	Deaths	Female	2018	5.65359	8.457714	3.563054	
Timor-Leste	Deaths	Female	2019	5.755366	8.532007	3.801308	
Timor-Leste	Deaths	Female	2020	6.133197	9.058054	4.154301	
Timor-Leste	Deaths	Female	2021	6.300652	9.201595	4.170956	
Timor-Leste	Deaths	Female	2022	6.567418	9.555136	4.113805	
Timor-Leste	Deaths	Female	2023	6.802783	10.13908	4.225961	
Timor-Leste	DALYs	Male	2010	252.4039	435.9381	158.358	
Timor-Leste	DALYs	Male	2011	265.7729	459.0122	168.0553	
Timor-Leste	DALYs	Male	2012	273.3886	468.8883	173.3411	
Timor-Leste	DALYs	Male	2013	271.4621	444.8761	174.8162	
Timor-Leste	DALYs	Male	2014	267.8246	443.3364	165.9682	

Timor-Leste	DALYs	Male	2015	282.5256	464.6134	176.3598	
Timor-Leste	DALYs	Male	2016	284.2492	481.0654	177.6365	
Timor-Leste	DALYs	Male	2017	285.0291	487.6294	175.2121	
Timor-Leste	DALYs	Male	2018	285.3355	480.8989	178.862	
Timor-Leste	DALYs	Male	2019	285.3845	477.9122	180.1122	
Timor-Leste	DALYs	Male	2020	298.74	502.8947	189.1058	
Timor-Leste	DALYs	Male	2021	299.8415	503.3031	189.178	
Timor-Leste	DALYs	Male	2022	304.5743	502.3809	194.0571	
Timor-Leste	DALYs	Male	2023	309.8058	521.4029	202.4293	
Timor-Leste	DALYs	Female	2010	119.8944	198.4809	70.70512	
Timor-Leste	DALYs	Female	2011	133.0123	217.2287	78.73529	
Timor-Leste	DALYs	Female	2012	140.9304	226.5465	83.05507	
Timor-Leste	DALYs	Female	2013	142.1824	231.2766	83.49713	
Timor-Leste	DALYs	Female	2014	142.3375	231.231	84.86469	
Timor-Leste	DALYs	Female	2015	149.1132	241.2081	88.82745	
Timor-Leste	DALYs	Female	2016	153.4875	237.9788	91.55111	
Timor-Leste	DALYs	Female	2017	158.1371	240.5781	96.25739	
Timor-Leste	DALYs	Female	2018	160.9446	240.8132	100.6727	
Timor-Leste	DALYs	Female	2019	164.3235	243.905	108.222	
Timor-Leste	DALYs	Female	2020	174.6017	262.7027	117.245	
Timor-Leste	DALYs	Female	2021	180.4201	262.9512	116.3641	
Timor-Leste	DALYs	Female	2022	187.3212	273.4972	114.5435	
Timor-Leste	DALYs	Female	2023	195.0605	286.3136	121.4211	
Togo	Incidence	Male	2010	2.717058	3.823635	1.801839	
Togo	Incidence	Male	2011	2.772861	3.898398	1.825014	
Togo	Incidence	Male	2012	2.791037	3.882069	1.842345	
Togo	Incidence	Male	2013	2.685027	3.646586	1.756144	
Togo	Incidence	Male	2014	2.723248	3.683404	1.857774	
Togo	Incidence	Male	2015	3.080652	4.198486	2.092251	
Togo	Incidence	Male	2016	3.162696	4.266753	2.133321	
Togo	Incidence	Male	2017	3.323227	4.437517	2.193928	
Togo	Incidence	Male	2018	3.406096	4.798484	2.23572	
Togo	Incidence	Male	2019	3.519676	4.969954	2.285282	
Togo	Incidence	Male	2020	3.676062	5.302052	2.412654	
Togo	Incidence	Male	2021	3.869376	5.528999	2.449048	
Togo	Incidence	Male	2022	4.342812	6.125229	2.830181	

Togo	Incidence	Male	2023	4.580561	6.471599	2.901637	
Togo	Incidence	Female	2010	1.084626	1.595271	0.710105	
Togo	Incidence	Female	2011	1.129078	1.618853	0.740404	
Togo	Incidence	Female	2012	1.155073	1.673868	0.759061	
Togo	Incidence	Female	2013	1.163138	1.715419	0.763355	
Togo	Incidence	Female	2014	1.173557	1.692492	0.771687	
Togo	Incidence	Female	2015	1.257683	1.73443	0.82045	
Togo	Incidence	Female	2016	1.335419	1.827751	0.880879	
Togo	Incidence	Female	2017	1.450251	1.985651	0.955518	
Togo	Incidence	Female	2018	1.510715	2.069094	1.014016	
Togo	Incidence	Female	2019	1.589226	2.223685	1.088463	
Togo	Incidence	Female	2020	1.614134	2.235646	1.113193	
Togo	Incidence	Female	2021	1.730067	2.388345	1.159196	
Togo	Incidence	Female	2022	1.984595	2.763977	1.26687	
Togo	Incidence	Female	2023	2.107717	3.028292	1.414647	
Togo	Deaths	Male	2010	2.649049	3.727561	1.756243	
Togo	Deaths	Male	2011	2.69979	3.748828	1.779357	
Togo	Deaths	Male	2012	2.71884	3.765495	1.802663	
Togo	Deaths	Male	2013	2.619639	3.571118	1.720088	
Togo	Deaths	Male	2014	2.658085	3.598742	1.817107	
Togo	Deaths	Male	2015	3.007716	4.095264	2.038304	
Togo	Deaths	Male	2016	3.082486	4.180493	2.09411	
Togo	Deaths	Male	2017	3.237536	4.34781	2.114811	
Togo	Deaths	Male	2018	3.313892	4.654047	2.175466	
Togo	Deaths	Male	2019	3.422899	4.830011	2.237853	
Togo	Deaths	Male	2020	3.580189	5.174071	2.362304	
Togo	Deaths	Male	2021	3.767823	5.412529	2.393167	
Togo	Deaths	Male	2022	4.22329	5.991141	2.748866	
Togo	Deaths	Male	2023	4.451961	6.339452	2.834593	
Togo	Deaths	Female	2010	1.059309	1.559096	0.693119	
Togo	Deaths	Female	2011	1.09998	1.582385	0.720843	
Togo	Deaths	Female	2012	1.124617	1.640124	0.735762	
Togo	Deaths	Female	2013	1.132678	1.678323	0.740329	
Togo	Deaths	Female	2014	1.141756	1.651912	0.750598	
Togo	Deaths	Female	2015	1.222986	1.691791	0.79885	
Togo	Deaths	Female	2016	1.294387	1.775003	0.85039	

Togo	Deaths	Female	2017	1.402602	1.920987	0.926824	
Togo	Deaths	Female	2018	1.459813	2.005273	0.984737	
Togo	Deaths	Female	2019	1.535171	2.144589	1.04979	
Togo	Deaths	Female	2020	1.558082	2.164373	1.071595	
Togo	Deaths	Female	2021	1.66804	2.303989	1.116449	
Togo	Deaths	Female	2022	1.91158	2.655173	1.220406	
Togo	Deaths	Female	2023	2.030468	2.894504	1.359379	
Togo	DALYs	Male	2010	79.31154	111.9949	52.43885	
Togo	DALYs	Male	2011	81.13177	116.2581	53.23287	
Togo	DALYs	Male	2012	81.44787	114.5454	53.16351	
Togo	DALYs	Male	2013	77.90456	105.4124	50.7546	
Togo	DALYs	Male	2014	78.84017	106.8067	54.29993	
Togo	DALYs	Male	2015	89.17114	120.2604	60.73687	
Togo	DALYs	Male	2016	91.74305	122.2065	60.94445	
Togo	DALYs	Male	2017	96.30889	131.7349	63.73941	
Togo	DALYs	Male	2018	98.83734	139.4075	64.4845	
Togo	DALYs	Male	2019	102.1561	145.2348	65.54439	
Togo	DALYs	Male	2020	106.3721	154.1088	68.95304	
Togo	DALYs	Male	2021	111.7665	158.2272	70.64567	
Togo	DALYs	Male	2022	125.1888	174.352	81.59629	
Togo	DALYs	Male	2023	131.6336	184.4076	84.14727	
Togo	DALYs	Female	2010	32.20773	47.19592	21.08161	
Togo	DALYs	Female	2011	33.59805	47.93597	22.0246	
Togo	DALYs	Female	2012	34.40201	49.49857	22.77235	
Togo	DALYs	Female	2013	34.61428	50.6653	22.80443	
Togo	DALYs	Female	2014	34.90768	50.47677	22.70028	
Togo	DALYs	Female	2015	37.3746	51.37994	24.46228	
Togo	DALYs	Female	2016	39.82986	54.32981	26.15064	
Togo	DALYs	Female	2017	43.44011	59.53072	28.60282	
Togo	DALYs	Female	2018	45.31255	62.04821	30.01695	
Togo	DALYs	Female	2019	47.63487	66.93898	32.46572	
Togo	DALYs	Female	2020	48.41272	66.97792	33.57334	
Togo	DALYs	Female	2021	51.88846	71.88995	34.50072	
Togo	DALYs	Female	2022	59.5156	82.3773	37.73222	
Togo	DALYs	Female	2023	63.07469	90.51211	42.73342	
Trinidad and To	Incidence	Male	2010	17.21897	19.64878	15.4422	

Trinidad and To	Incidence	Male	2011	17.10481	19.5878	15.26036	
Trinidad and To	Incidence	Male	2012	17.70841	20.11677	15.79469	
Trinidad and To	Incidence	Male	2013	18.2136	20.85889	16.01065	
Trinidad and To	Incidence	Male	2014	18.91048	21.92577	16.63807	
Trinidad and To	Incidence	Male	2015	18.93605	22.15033	16.10262	
Trinidad and To	Incidence	Male	2016	19.13292	22.36926	16.56705	
Trinidad and To	Incidence	Male	2017	19.67246	22.95262	16.84869	
Trinidad and To	Incidence	Male	2018	19.85862	23.10589	17.14305	
Trinidad and To	Incidence	Male	2019	20.7647	24.40062	17.80687	
Trinidad and To	Incidence	Male	2020	22.15905	26.01119	18.82003	
Trinidad and To	Incidence	Male	2021	25.21969	29.49467	21.62348	
Trinidad and To	Incidence	Male	2022	24.78953	29.04654	21.3134	
Trinidad and To	Incidence	Male	2023	22.92086	26.57402	19.50347	
Trinidad and To	Incidence	Female	2010	6.126471	7.268486	5.251604	
Trinidad and To	Incidence	Female	2011	6.315732	7.600914	5.456411	
Trinidad and To	Incidence	Female	2012	6.247242	7.551753	5.374908	
Trinidad and To	Incidence	Female	2013	6.464031	7.709404	5.504729	
Trinidad and To	Incidence	Female	2014	6.706953	8.031901	5.683642	
Trinidad and To	Incidence	Female	2015	6.686401	8.045359	5.57938	
Trinidad and To	Incidence	Female	2016	6.81677	8.153646	5.725196	
Trinidad and To	Incidence	Female	2017	7.007153	8.458814	5.909868	
Trinidad and To	Incidence	Female	2018	7.201397	8.689488	6.074804	
Trinidad and To	Incidence	Female	2019	7.438769	9.041716	6.27519	
Trinidad and To	Incidence	Female	2020	7.661286	9.303698	6.425948	
Trinidad and To	Incidence	Female	2021	8.217578	9.900431	6.98132	
Trinidad and To	Incidence	Female	2022	8.41114	9.857239	7.116564	
Trinidad and To	Incidence	Female	2023	8.996933	10.65693	7.508212	
Trinidad and To	Deaths	Male	2010	16.66246	18.8787	15.033	
Trinidad and To	Deaths	Male	2011	16.49585	18.79265	14.77813	
Trinidad and To	Deaths	Male	2012	17.07327	19.303	15.30905	
Trinidad and To	Deaths	Male	2013	17.53992	20.02674	15.56539	
Trinidad and To	Deaths	Male	2014	18.20128	21.01309	16.01625	
Trinidad and To	Deaths	Male	2015	18.21801	21.25533	15.58674	
Trinidad and To	Deaths	Male	2016	18.43077	21.34112	16.02439	
Trinidad and To	Deaths	Male	2017	18.95776	22.03575	16.40024	
Trinidad and To	Deaths	Male	2018	19.13947	22.18469	16.57921	

Trinidad and To	Deaths	Male	2019	20.02486	23.43545	17.16628	
Trinidad and To	Deaths	Male	2020	21.39069	25.02997	18.45479	
Trinidad and To	Deaths	Male	2021	24.32454	28.32569	21.00576	
Trinidad and To	Deaths	Male	2022	23.97958	27.97064	20.56066	
Trinidad and To	Deaths	Male	2023	22.14787	25.75151	19.01096	
Trinidad and To	Deaths	Female	2010	5.920417	6.997027	5.088395	
Trinidad and To	Deaths	Female	2011	6.042718	7.235385	5.259413	
Trinidad and To	Deaths	Female	2012	6.000593	7.167451	5.140412	
Trinidad and To	Deaths	Female	2013	6.201724	7.41622	5.282757	
Trinidad and To	Deaths	Female	2014	6.434177	7.701172	5.491831	
Trinidad and To	Deaths	Female	2015	6.412181	7.595215	5.39961	
Trinidad and To	Deaths	Female	2016	6.54581	7.742878	5.557686	
Trinidad and To	Deaths	Female	2017	6.725278	8.016527	5.680197	
Trinidad and To	Deaths	Female	2018	6.907062	8.256565	5.848042	
Trinidad and To	Deaths	Female	2019	7.136331	8.61748	6.00873	
Trinidad and To	Deaths	Female	2020	7.352	8.912461	6.170342	
Trinidad and To	Deaths	Female	2021	7.862067	9.528579	6.68428	
Trinidad and To	Deaths	Female	2022	8.07726	9.59033	6.86673	
Trinidad and To	Deaths	Female	2023	8.652598	10.30585	7.260514	
Trinidad and To	DALYs	Male	2010	443.6847	506.1784	397.5062	
Trinidad and To	DALYs	Male	2011	438.864	501.322	391.9133	
Trinidad and To	DALYs	Male	2012	453.1418	514.0304	402.7568	
Trinidad and To	DALYs	Male	2013	464.9137	534.8258	410.2968	
Trinidad and To	DALYs	Male	2014	482.1718	562.0068	422.1207	
Trinidad and To	DALYs	Male	2015	481.9128	567.3053	411.8396	
Trinidad and To	DALYs	Male	2016	485.068	562.9083	419.4518	
Trinidad and To	DALYs	Male	2017	497.2932	577.6113	431.1931	
Trinidad and To	DALYs	Male	2018	500.3661	583.2249	434.5881	
Trinidad and To	DALYs	Male	2019	520.578	608.9028	450.5706	
Trinidad and To	DALYs	Male	2020	552.5487	647.1283	471.1535	
Trinidad and To	DALYs	Male	2021	626.9454	733.4413	539.2681	
Trinidad and To	DALYs	Male	2022	609.5196	713.8803	520.9564	
Trinidad and To	DALYs	Male	2023	561.9387	651.8949	473.8017	
Trinidad and To	DALYs	Female	2010	151.2373	179.1395	129.3863	
Trinidad and To	DALYs	Female	2011	155.8008	185.6644	135.0708	
Trinidad and To	DALYs	Female	2012	153.0017	183.3822	131.0619	

Trinidad and To	DALYs	Female	2013	157.7365	189.2388	133.5596	
Trinidad and To	DALYs	Female	2014	163.1333	197.5733	139.0742	
Trinidad and To	DALYs	Female	2015	162.1643	193.039	136.0093	
Trinidad and To	DALYs	Female	2016	164.6708	195.0882	139.082	
Trinidad and To	DALYs	Female	2017	168.7772	201.076	142.0204	
Trinidad and To	DALYs	Female	2018	172.8813	206.8729	145.0647	
Trinidad and To	DALYs	Female	2019	177.6036	214.8297	149.2917	
Trinidad and To	DALYs	Female	2020	182.1361	221.6444	153.0548	
Trinidad and To	DALYs	Female	2021	195.9458	237.9013	165.4492	
Trinidad and To	DALYs	Female	2022	197.0487	234.0565	167.1617	
Trinidad and To	DALYs	Female	2023	207.7855	247.9067	174.2748	
Tunisia	Incidence	Male	2010	48.2929	64.43802	36.17002	
Tunisia	Incidence	Male	2011	48.60785	64.02817	36.6524	
Tunisia	Incidence	Male	2012	49.56391	64.30973	37.25026	
Tunisia	Incidence	Male	2013	50.26836	64.90701	37.59293	
Tunisia	Incidence	Male	2014	51.74409	66.65642	37.68817	
Tunisia	Incidence	Male	2015	52.72819	68.2591	38.74861	
Tunisia	Incidence	Male	2016	53.99956	70.41418	41.23553	
Tunisia	Incidence	Male	2017	54.79146	70.24121	42.42009	
Tunisia	Incidence	Male	2018	56.11333	72.41615	43.38165	
Tunisia	Incidence	Male	2019	57.88951	74.84856	44.89847	
Tunisia	Incidence	Male	2020	55.12974	72.21637	43.81243	
Tunisia	Incidence	Male	2021	51.81416	68.52273	41.64019	
Tunisia	Incidence	Male	2022	59.92742	77.56498	47.2885	
Tunisia	Incidence	Male	2023	69.71624	91.57557	53.85976	
Tunisia	Incidence	Female	2010	7.035201	9.747264	4.793849	
Tunisia	Incidence	Female	2011	7.260823	10.06248	4.987194	
Tunisia	Incidence	Female	2012	7.513571	10.31837	5.189335	
Tunisia	Incidence	Female	2013	7.711101	10.44059	5.407356	
Tunisia	Incidence	Female	2014	8.004055	10.68973	5.609222	
Tunisia	Incidence	Female	2015	8.297947	10.87436	5.840863	
Tunisia	Incidence	Female	2016	8.639093	11.47754	6.276995	
Tunisia	Incidence	Female	2017	9.047598	11.90401	6.69608	
Tunisia	Incidence	Female	2018	9.419148	11.82738	7.080595	
Tunisia	Incidence	Female	2019	9.989407	12.7511	7.551128	
Tunisia	Incidence	Female	2020	9.83939	12.73266	7.65365	

Tunisia	Incidence	Female	2021	9.301632	11.9495	7.287499	
Tunisia	Incidence	Female	2022	11.07029	14.20311	8.279674	
Tunisia	Incidence	Female	2023	13.2509	17.1559	9.745929	
Tunisia	Deaths	Male	2010	47.57455	63.4514	35.78961	
Tunisia	Deaths	Male	2011	47.78346	63.13989	35.83278	
Tunisia	Deaths	Male	2012	48.60651	63.41458	36.30378	
Tunisia	Deaths	Male	2013	49.17877	63.41883	36.79163	
Tunisia	Deaths	Male	2014	50.48324	65.44473	36.93975	
Tunisia	Deaths	Male	2015	51.31799	66.36522	37.67015	
Tunisia	Deaths	Male	2016	52.48532	68.21698	40.05667	
Tunisia	Deaths	Male	2017	53.09544	68.78317	41.06389	
Tunisia	Deaths	Male	2018	54.18911	70.68698	42.28426	
Tunisia	Deaths	Male	2019	55.7268	72.45691	43.353	
Tunisia	Deaths	Male	2020	52.85914	69.16469	42.57368	
Tunisia	Deaths	Male	2021	49.32422	65.40028	39.36141	
Tunisia	Deaths	Male	2022	56.44	73.42333	44.50675	
Tunisia	Deaths	Male	2023	65.59855	87.36611	50.36034	
Tunisia	Deaths	Female	2010	6.894826	9.61509	4.739438	
Tunisia	Deaths	Female	2011	7.107128	9.81931	4.886256	
Tunisia	Deaths	Female	2012	7.342165	10.04777	5.089531	
Tunisia	Deaths	Female	2013	7.521322	10.14433	5.312611	
Tunisia	Deaths	Female	2014	7.785459	10.33992	5.462314	
Tunisia	Deaths	Female	2015	8.051725	10.54971	5.753865	
Tunisia	Deaths	Female	2016	8.369479	11.0915	6.059554	
Tunisia	Deaths	Female	2017	8.73712	11.48056	6.414154	
Tunisia	Deaths	Female	2018	9.058231	11.4489	6.805448	
Tunisia	Deaths	Female	2019	9.564842	12.33954	7.155124	
Tunisia	Deaths	Female	2020	9.34177	12.11856	7.348264	
Tunisia	Deaths	Female	2021	8.74893	11.28531	6.881413	
Tunisia	Deaths	Female	2022	10.31177	13.38633	7.726384	
Tunisia	Deaths	Female	2023	12.34204	16.16578	9.062754	
Tunisia	DALYs	Male	2010	1177.655	1559.182	895.7416	
Tunisia	DALYs	Male	2011	1185.376	1550.97	901.5548	
Tunisia	DALYs	Male	2012	1209.077	1560.831	919.1028	
Tunisia	DALYs	Male	2013	1226.465	1566.797	928.2725	
Tunisia	DALYs	Male	2014	1261.808	1617.499	931.1821	

Tunisia	DALYs	Male	2015	1284.253	1650.764	949.01	
Tunisia	DALYs	Male	2016	1311.942	1694.659	1002.132	
Tunisia	DALYs	Male	2017	1329.718	1708.172	1037.498	
Tunisia	DALYs	Male	2018	1358.917	1763.768	1070.211	
Tunisia	DALYs	Male	2019	1393.341	1798.226	1104.583	
Tunisia	DALYs	Male	2020	1315.793	1719.4	1067.706	
Tunisia	DALYs	Male	2021	1229.99	1622.782	985.7358	
Tunisia	DALYs	Male	2022	1435.635	1851.569	1135.332	
Tunisia	DALYs	Male	2023	1656.496	2210.847	1282.093	
Tunisia	DALYs	Female	2010	171.6374	237.5577	119.9787	
Tunisia	DALYs	Female	2011	176.4895	243.5394	122.5346	
Tunisia	DALYs	Female	2012	182.039	249.3286	128.3155	
Tunisia	DALYs	Female	2013	186.2906	249.5473	132.1005	
Tunisia	DALYs	Female	2014	192.7528	254.6191	136.6246	
Tunisia	DALYs	Female	2015	199.207	258.6344	143.1412	
Tunisia	DALYs	Female	2016	206.5211	269.239	150.688	
Tunisia	DALYs	Female	2017	215.6506	280.6088	161.5979	
Tunisia	DALYs	Female	2018	223.701	284.4225	170.1569	
Tunisia	DALYs	Female	2019	235.3639	304.9228	178.9431	
Tunisia	DALYs	Female	2020	231.1875	302.8112	181.706	
Tunisia	DALYs	Female	2021	217.505	280.4627	170.7049	
Tunisia	DALYs	Female	2022	258.6368	337.5668	196.6126	
Tunisia	DALYs	Female	2023	305.7248	394.9189	225.4772	
Turkmenistan	Incidence	Male	2010	8.294939	9.163774	7.515082	
Turkmenistan	Incidence	Male	2011	9.157073	10.2588	8.265283	
Turkmenistan	Incidence	Male	2012	8.66171	9.654001	7.888118	
Turkmenistan	Incidence	Male	2013	8.721989	9.620917	7.886807	
Turkmenistan	Incidence	Male	2014	9.855815	10.94138	8.790693	
Turkmenistan	Incidence	Male	2015	9.2897	10.3787	8.292476	
Turkmenistan	Incidence	Male	2016	9.44468	10.5591	8.547108	
Turkmenistan	Incidence	Male	2017	9.404018	10.80097	8.421306	
Turkmenistan	Incidence	Male	2018	9.672616	11.49082	8.587178	
Turkmenistan	Incidence	Male	2019	9.611423	11.41753	8.506936	
Turkmenistan	Incidence	Male	2020	9.917425	11.88301	8.61055	
Turkmenistan	Incidence	Male	2021	10.43949	12.65313	8.671188	
Turkmenistan	Incidence	Male	2022	10.36865	12.67364	8.927292	

Turkmenistan	Incidence	Male	2023	10.5403	12.82359	8.86691	
Turkmenistan	Incidence	Female	2010	2.736966	3.186876	2.37423	
Turkmenistan	Incidence	Female	2011	2.884753	3.3109	2.501733	
Turkmenistan	Incidence	Female	2012	2.884024	3.315226	2.487981	
Turkmenistan	Incidence	Female	2013	2.829088	3.242792	2.42947	
Turkmenistan	Incidence	Female	2014	3.042675	3.519241	2.627576	
Turkmenistan	Incidence	Female	2015	2.959629	3.427277	2.552182	
Turkmenistan	Incidence	Female	2016	3.064266	3.565318	2.607742	
Turkmenistan	Incidence	Female	2017	3.219978	3.777286	2.697047	
Turkmenistan	Incidence	Female	2018	3.329161	3.940165	2.82175	
Turkmenistan	Incidence	Female	2019	3.462039	4.054165	2.956774	
Turkmenistan	Incidence	Female	2020	3.581894	4.285481	2.992839	
Turkmenistan	Incidence	Female	2021	3.811687	4.608678	3.163055	
Turkmenistan	Incidence	Female	2022	3.809783	4.76942	3.089677	
Turkmenistan	Incidence	Female	2023	3.85172	4.674	3.117249	
Turkmenistan	Deaths	Male	2010	7.880038	8.722382	7.148526	
Turkmenistan	Deaths	Male	2011	8.701566	9.736532	7.839246	
Turkmenistan	Deaths	Male	2012	8.226505	9.143908	7.511593	
Turkmenistan	Deaths	Male	2013	8.290012	9.157062	7.497331	
Turkmenistan	Deaths	Male	2014	9.363848	10.4236	8.382191	
Turkmenistan	Deaths	Male	2015	8.79154	9.798234	7.849223	
Turkmenistan	Deaths	Male	2016	8.94202	10.01234	8.099797	
Turkmenistan	Deaths	Male	2017	8.910037	10.20983	7.971696	
Turkmenistan	Deaths	Male	2018	9.171733	10.86525	8.146582	
Turkmenistan	Deaths	Male	2019	9.121079	10.83186	8.105068	
Turkmenistan	Deaths	Male	2020	9.416424	11.20669	8.147488	
Turkmenistan	Deaths	Male	2021	9.912312	11.94363	8.251233	
Turkmenistan	Deaths	Male	2022	9.861943	12.02004	8.521984	
Turkmenistan	Deaths	Male	2023	10.04445	12.13152	8.495605	
Turkmenistan	Deaths	Female	2010	2.625127	3.049633	2.286548	
Turkmenistan	Deaths	Female	2011	2.775301	3.181952	2.408139	
Turkmenistan	Deaths	Female	2012	2.761723	3.177247	2.381496	
Turkmenistan	Deaths	Female	2013	2.70213	3.094924	2.32308	
Turkmenistan	Deaths	Female	2014	2.898395	3.345396	2.506798	
Turkmenistan	Deaths	Female	2015	2.818206	3.265077	2.418053	
Turkmenistan	Deaths	Female	2016	2.919895	3.384887	2.482205	

Turkmenistan	Deaths	Female	2017	3.070719	3.597056	2.572349	
Turkmenistan	Deaths	Female	2018	3.175951	3.770049	2.699128	
Turkmenistan	Deaths	Female	2019	3.305172	3.85369	2.821681	
Turkmenistan	Deaths	Female	2020	3.422221	4.122156	2.869874	
Turkmenistan	Deaths	Female	2021	3.639417	4.422714	3.01023	
Turkmenistan	Deaths	Female	2022	3.64481	4.564942	2.957338	
Turkmenistan	Deaths	Female	2023	3.690637	4.478757	2.992717	
Turkmenistan	DALYs	Male	2010	249.2099	276.1905	225.1553	
Turkmenistan	DALYs	Male	2011	273.8603	306.6215	245.334	
Turkmenistan	DALYs	Male	2012	258.925	289.4662	235.6734	
Turkmenistan	DALYs	Male	2013	260.4289	288.306	234.7936	
Turkmenistan	DALYs	Male	2014	293.3946	325.5039	259.9966	
Turkmenistan	DALYs	Male	2015	279.0562	312.7306	248.556	
Turkmenistan	DALYs	Male	2016	282.8657	319.4113	255.5799	
Turkmenistan	DALYs	Male	2017	280.6996	323.892	250.8638	
Turkmenistan	DALYs	Male	2018	287.5855	341.7123	254.3579	
Turkmenistan	DALYs	Male	2019	284.4022	339.8648	250.2838	
Turkmenistan	DALYs	Male	2020	292.1074	350.4032	254.6789	
Turkmenistan	DALYs	Male	2021	306.5321	373.9613	255.6336	
Turkmenistan	DALYs	Male	2022	302.9205	373.411	259.8625	
Turkmenistan	DALYs	Male	2023	306.0554	369.4057	255.9673	
Turkmenistan	DALYs	Female	2010	82.18565	96.09872	70.74491	
Turkmenistan	DALYs	Female	2011	85.90251	98.73729	74.28317	
Turkmenistan	DALYs	Female	2012	86.63234	100.0244	74.23502	
Turkmenistan	DALYs	Female	2013	85.32873	97.59936	72.3785	
Turkmenistan	DALYs	Female	2014	92.05397	106.0459	78.82363	
Turkmenistan	DALYs	Female	2015	89.50989	104.2999	77.48454	
Turkmenistan	DALYs	Female	2016	92.26549	107.9894	78.54841	
Turkmenistan	DALYs	Female	2017	96.52131	114.2472	81.08434	
Turkmenistan	DALYs	Female	2018	99.48905	117.9112	84.77061	
Turkmenistan	DALYs	Female	2019	102.9116	121.0695	87.74438	
Turkmenistan	DALYs	Female	2020	105.6579	125.6233	88.3517	
Turkmenistan	DALYs	Female	2021	112.0989	135.4371	93.31436	
Turkmenistan	DALYs	Female	2022	111.3949	138.7958	90.06271	
Turkmenistan	DALYs	Female	2023	112.013	136.2444	90.96137	
Uganda	Incidence	Male	2010	2.753141	3.759613	1.98673	

Uganda	Incidence	Male	2011	2.758475	3.89558	2.024715	
Uganda	Incidence	Male	2012	2.772498	3.826694	1.983972	
Uganda	Incidence	Male	2013	2.760223	3.910782	1.949264	
Uganda	Incidence	Male	2014	2.797092	3.92201	2.033752	
Uganda	Incidence	Male	2015	2.841307	3.895661	2.042812	
Uganda	Incidence	Male	2016	2.816065	3.927004	2.019435	
Uganda	Incidence	Male	2017	2.790346	3.997754	1.983876	
Uganda	Incidence	Male	2018	2.805526	4.073662	1.991449	
Uganda	Incidence	Male	2019	2.809141	4.00641	1.959047	
Uganda	Incidence	Male	2020	2.88178	4.133379	1.948336	
Uganda	Incidence	Male	2021	3.433479	4.986789	2.382571	
Uganda	Incidence	Male	2022	3.191043	4.703247	2.235043	
Uganda	Incidence	Male	2023	3.384643	4.701193	2.315755	
Uganda	Incidence	Female	2010	1.580163	2.20761	1.087823	
Uganda	Incidence	Female	2011	1.61062	2.260239	1.125569	
Uganda	Incidence	Female	2012	1.686793	2.37254	1.162799	
Uganda	Incidence	Female	2013	1.766798	2.499978	1.221503	
Uganda	Incidence	Female	2014	1.795538	2.512041	1.218576	
Uganda	Incidence	Female	2015	1.844466	2.557331	1.222047	
Uganda	Incidence	Female	2016	1.863857	2.613886	1.231835	
Uganda	Incidence	Female	2017	1.913419	2.689375	1.269722	
Uganda	Incidence	Female	2018	1.984979	2.760821	1.346275	
Uganda	Incidence	Female	2019	2.051093	2.803229	1.422175	
Uganda	Incidence	Female	2020	2.090538	2.877815	1.45431	
Uganda	Incidence	Female	2021	2.374018	3.293288	1.598365	
Uganda	Incidence	Female	2022	2.494591	3.582502	1.683059	
Uganda	Incidence	Female	2023	2.710319	4.021078	1.796857	
Uganda	Deaths	Male	2010	2.654175	3.619728	1.94831	
Uganda	Deaths	Male	2011	2.657653	3.744675	1.964146	
Uganda	Deaths	Male	2012	2.673319	3.719807	1.934286	
Uganda	Deaths	Male	2013	2.66195	3.797469	1.896797	
Uganda	Deaths	Male	2014	2.694936	3.789743	1.975014	
Uganda	Deaths	Male	2015	2.734201	3.728801	1.975249	
Uganda	Deaths	Male	2016	2.710219	3.787064	1.93612	
Uganda	Deaths	Male	2017	2.685964	3.846397	1.909819	
Uganda	Deaths	Male	2018	2.700643	3.931435	1.907673	

Uganda	Deaths	Male	2019	2.704339	3.866598	1.882368	
Uganda	Deaths	Male	2020	2.775032	3.978788	1.86642	
Uganda	Deaths	Male	2021	3.317848	4.822327	2.296034	
Uganda	Deaths	Male	2022	3.060945	4.474227	2.135575	
Uganda	Deaths	Male	2023	3.244588	4.441318	2.234798	
Uganda	Deaths	Female	2010	1.519601	2.126724	1.047006	
Uganda	Deaths	Female	2011	1.549373	2.165752	1.077229	
Uganda	Deaths	Female	2012	1.617453	2.287885	1.118466	
Uganda	Deaths	Female	2013	1.684494	2.363339	1.158957	
Uganda	Deaths	Female	2014	1.706625	2.398195	1.157955	
Uganda	Deaths	Female	2015	1.750021	2.427441	1.164601	
Uganda	Deaths	Female	2016	1.768116	2.495341	1.168749	
Uganda	Deaths	Female	2017	1.814438	2.566775	1.211865	
Uganda	Deaths	Female	2018	1.879179	2.612115	1.267016	
Uganda	Deaths	Female	2019	1.938521	2.641597	1.344009	
Uganda	Deaths	Female	2020	1.970334	2.703487	1.369275	
Uganda	Deaths	Female	2021	2.226892	3.092486	1.490176	
Uganda	Deaths	Female	2022	2.337248	3.354832	1.572945	
Uganda	Deaths	Female	2023	2.535675	3.724278	1.678343	
Uganda	DALYs	Male	2010	83.33926	114.3053	59.08504	
Uganda	DALYs	Male	2011	83.63655	117.8892	60.06451	
Uganda	DALYs	Male	2012	83.87519	115.2967	58.29259	
Uganda	DALYs	Male	2013	83.49095	117.5163	58.1111	
Uganda	DALYs	Male	2014	84.79451	118.2884	59.1213	
Uganda	DALYs	Male	2015	86.35451	119.8772	61.24212	
Uganda	DALYs	Male	2016	85.62082	120.3243	60.86887	
Uganda	DALYs	Male	2017	84.85965	122.2583	60.38571	
Uganda	DALYs	Male	2018	85.33582	124.5865	60.42521	
Uganda	DALYs	Male	2019	85.36703	122.0951	59.30823	
Uganda	DALYs	Male	2020	87.34911	127.9782	59.04986	
Uganda	DALYs	Male	2021	102.9067	150.0518	71.60053	
Uganda	DALYs	Male	2022	96.89846	143.6205	67.9908	
Uganda	DALYs	Male	2023	102.6395	142.0979	69.68696	
Uganda	DALYs	Female	2010	48.72507	67.00839	33.68532	
Uganda	DALYs	Female	2011	49.52673	69.21281	34.48993	
Uganda	DALYs	Female	2012	52.14851	72.85952	36.27186	

Uganda	DALYs	Female	2013	55.32636	77.74163	38.19238	
Uganda	DALYs	Female	2014	56.61961	79.31115	37.45698	
Uganda	DALYs	Female	2015	58.34943	81.33874	37.62798	
Uganda	DALYs	Female	2016	58.94939	83.70626	37.94056	
Uganda	DALYs	Female	2017	60.51969	85.13703	38.85468	
Uganda	DALYs	Female	2018	62.96243	87.83982	42.29162	
Uganda	DALYs	Female	2019	65.20875	90.38744	44.84909	
Uganda	DALYs	Female	2020	66.63699	92.15004	45.91631	
Uganda	DALYs	Female	2021	75.79704	104.935	51.00212	
Uganda	DALYs	Female	2022	79.98739	114.604	53.27059	
Uganda	DALYs	Female	2023	86.98822	129.7738	56.74639	
Ukraine	Incidence	Male	2010	68.39074	72.62729	64.85249	
Ukraine	Incidence	Male	2011	69.86296	73.75265	66.30433	
Ukraine	Incidence	Male	2012	71.8547	76.20824	68.01011	
Ukraine	Incidence	Male	2013	72.18837	76.6122	68.11458	
Ukraine	Incidence	Male	2014	67.30348	71.72992	63.31851	
Ukraine	Incidence	Male	2015	61.61284	65.72788	57.37381	
Ukraine	Incidence	Male	2016	62.22992	66.75071	57.46394	
Ukraine	Incidence	Male	2017	64.37395	69.86897	59.24455	
Ukraine	Incidence	Male	2018	65.10731	71.84013	58.71821	
Ukraine	Incidence	Male	2019	65.12709	71.54342	58.67003	
Ukraine	Incidence	Male	2020	68.5317	75.92935	60.73364	
Ukraine	Incidence	Male	2021	66.87369	74.00014	59.28371	
Ukraine	Incidence	Male	2022	65.29099	74.48976	56.82451	
Ukraine	Incidence	Male	2023	68.23667	80.30737	57.6333	
Ukraine	Incidence	Female	2010	11.47069	12.26379	10.73854	
Ukraine	Incidence	Female	2011	11.70101	12.52166	11.02404	
Ukraine	Incidence	Female	2012	12.27962	13.20169	11.53487	
Ukraine	Incidence	Female	2013	12.28947	13.21818	11.49368	
Ukraine	Incidence	Female	2014	11.71758	12.4243	10.93728	
Ukraine	Incidence	Female	2015	10.90252	11.694	10.19907	
Ukraine	Incidence	Female	2016	11.26348	12.10095	10.5136	
Ukraine	Incidence	Female	2017	11.61548	12.49709	10.81361	
Ukraine	Incidence	Female	2018	11.41934	12.28479	10.57907	
Ukraine	Incidence	Female	2019	11.75923	12.71662	10.8355	
Ukraine	Incidence	Female	2020	12.46091	13.38299	11.42484	

Ukraine	Incidence	Female	2021	11.79907	12.63204	10.86025	
Ukraine	Incidence	Female	2022	11.42459	13.01117	9.989623	
Ukraine	Incidence	Female	2023	12.48593	14.5465	10.49485	
Ukraine	Deaths	Male	2010	59.20434	62.33039	56.42914	
Ukraine	Deaths	Male	2011	60.29099	63.48901	57.48029	
Ukraine	Deaths	Male	2012	61.96442	65.62935	58.88812	
Ukraine	Deaths	Male	2013	62.18183	66.04091	59.12898	
Ukraine	Deaths	Male	2014	57.8889	61.40632	54.89328	
Ukraine	Deaths	Male	2015	52.99547	56.32604	49.89822	
Ukraine	Deaths	Male	2016	53.51887	57.00873	49.87081	
Ukraine	Deaths	Male	2017	55.31322	59.63134	50.95819	
Ukraine	Deaths	Male	2018	55.86869	61.42563	50.30525	
Ukraine	Deaths	Male	2019	55.75334	61.06636	50.2688	
Ukraine	Deaths	Male	2020	58.47899	64.28476	52.06995	
Ukraine	Deaths	Male	2021	56.7318	63.01082	50.55061	
Ukraine	Deaths	Male	2022	55.14162	62.2837	47.96305	
Ukraine	Deaths	Male	2023	57.64282	67.80786	49.14558	
Ukraine	Deaths	Female	2010	10.21289	10.71115	9.753185	
Ukraine	Deaths	Female	2011	10.37461	10.85992	9.931626	
Ukraine	Deaths	Female	2012	10.88987	11.39485	10.33314	
Ukraine	Deaths	Female	2013	10.89618	11.42436	10.38957	
Ukraine	Deaths	Female	2014	10.3703	10.8379	9.901271	
Ukraine	Deaths	Female	2015	9.643779	10.04424	9.172365	
Ukraine	Deaths	Female	2016	9.933394	10.40095	9.460633	
Ukraine	Deaths	Female	2017	10.247	10.7734	9.716942	
Ukraine	Deaths	Female	2018	10.07107	10.61417	9.482873	
Ukraine	Deaths	Female	2019	10.33864	10.94918	9.66635	
Ukraine	Deaths	Female	2020	10.90662	11.49524	10.24975	
Ukraine	Deaths	Female	2021	10.23965	10.71552	9.710054	
Ukraine	Deaths	Female	2022	9.829375	10.95868	8.776186	
Ukraine	Deaths	Female	2023	10.73962	12.3558	9.238411	
Ukraine	DALYs	Male	2010	1619.984	1703.754	1545.488	
Ukraine	DALYs	Male	2011	1645.291	1734.857	1565.119	
Ukraine	DALYs	Male	2012	1686.28	1789.218	1600.937	
Ukraine	DALYs	Male	2013	1690.711	1801.547	1605.122	
Ukraine	DALYs	Male	2014	1574.462	1676.143	1491.478	

Ukraine	DALYs	Male	2015	1438.234	1530.918	1352.957	
Ukraine	DALYs	Male	2016	1449.565	1546.034	1350.831	
Ukraine	DALYs	Male	2017	1501.089	1617.23	1384.726	
Ukraine	DALYs	Male	2018	1518.114	1666.898	1369.523	
Ukraine	DALYs	Male	2019	1513.067	1656.83	1358.043	
Ukraine	DALYs	Male	2020	1577.181	1736.396	1395.407	
Ukraine	DALYs	Male	2021	1530.563	1699.805	1360.36	
Ukraine	DALYs	Male	2022	1499.543	1700.411	1307.074	
Ukraine	DALYs	Male	2023	1558.227	1834.225	1324.522	
Ukraine	DALYs	Female	2010	256.7798	270.2683	244.4098	
Ukraine	DALYs	Female	2011	259.4669	271.8307	247.9207	
Ukraine	DALYs	Female	2012	270.3096	283.1223	256.245	
Ukraine	DALYs	Female	2013	269.7783	283.315	257.4096	
Ukraine	DALYs	Female	2014	256.9931	269.1132	245.8491	
Ukraine	DALYs	Female	2015	238.8811	248.7834	227.4451	
Ukraine	DALYs	Female	2016	246.6446	258.6212	234.9321	
Ukraine	DALYs	Female	2017	253.9453	267.0279	240.619	
Ukraine	DALYs	Female	2018	249.7176	263.9794	235.2915	
Ukraine	DALYs	Female	2019	256.2847	272.9671	241.6555	
Ukraine	DALYs	Female	2020	269.3634	285.1032	254.1435	
Ukraine	DALYs	Female	2021	256.6561	270.0115	244.6889	
Ukraine	DALYs	Female	2022	253.5233	284.6821	226.7427	
Ukraine	DALYs	Female	2023	272.6701	315.1719	232.3381	
United Arab Em	Incidence	Male	2010	2.841935	3.972921	1.925587	
United Arab Em	Incidence	Male	2011	2.826896	3.95179	1.975576	
United Arab Em	Incidence	Male	2012	2.78122	3.808606	2.002845	
United Arab Em	Incidence	Male	2013	2.672754	3.574863	1.916108	
United Arab Em	Incidence	Male	2014	2.630622	3.518358	1.892354	
United Arab Em	Incidence	Male	2015	2.771314	3.798508	1.957668	
United Arab Em	Incidence	Male	2016	2.936061	4.07134	2.08153	
United Arab Em	Incidence	Male	2017	3.150331	4.444044	2.287603	
United Arab Em	Incidence	Male	2018	3.337266	4.840606	2.404514	
United Arab Em	Incidence	Male	2019	3.664264	5.262586	2.611129	
United Arab Em	Incidence	Male	2020	3.177353	4.789851	2.282195	
United Arab Em	Incidence	Male	2021	3.44629	5.136772	2.465117	
United Arab Em	Incidence	Male	2022	4.454707	6.583332	3.089927	

United Arab Em	Incidence	Male	2023	5.685988	8.375364	3.998524	
United Arab Em	Incidence	Female	2010	1.87115	2.666935	1.31713	
United Arab Em	Incidence	Female	2011	1.824769	2.557329	1.270547	
United Arab Em	Incidence	Female	2012	1.768438	2.421134	1.220148	
United Arab Em	Incidence	Female	2013	1.739483	2.402574	1.184348	
United Arab Em	Incidence	Female	2014	1.752902	2.400618	1.188451	
United Arab Em	Incidence	Female	2015	1.770839	2.356627	1.203861	
United Arab Em	Incidence	Female	2016	1.780394	2.378725	1.232367	
United Arab Em	Incidence	Female	2017	1.825946	2.462216	1.267108	
United Arab Em	Incidence	Female	2018	1.885243	2.526534	1.321433	
United Arab Em	Incidence	Female	2019	2.046794	2.728612	1.441771	
United Arab Em	Incidence	Female	2020	2.103264	2.834382	1.520766	
United Arab Em	Incidence	Female	2021	2.236196	3.043804	1.619283	
United Arab Em	Incidence	Female	2022	2.495287	3.375801	1.756128	
United Arab Em	Incidence	Female	2023	2.825731	3.805553	1.94715	
United Arab Em	Deaths	Male	2010	2.610975	3.660846	1.776438	
United Arab Em	Deaths	Male	2011	2.590654	3.61309	1.838434	
United Arab Em	Deaths	Male	2012	2.545092	3.493034	1.829414	
United Arab Em	Deaths	Male	2013	2.449864	3.312884	1.753322	
United Arab Em	Deaths	Male	2014	2.420403	3.238538	1.736956	
United Arab Em	Deaths	Male	2015	2.561818	3.515344	1.802257	
United Arab Em	Deaths	Male	2016	2.729525	3.78883	1.933126	
United Arab Em	Deaths	Male	2017	2.932229	4.153818	2.126525	
United Arab Em	Deaths	Male	2018	3.108052	4.524197	2.237545	
United Arab Em	Deaths	Male	2019	3.417919	4.910612	2.431896	
United Arab Em	Deaths	Male	2020	2.960789	4.468464	2.126556	
United Arab Em	Deaths	Male	2021	3.203962	4.784979	2.290619	
United Arab Em	Deaths	Male	2022	4.124375	6.13885	2.886961	
United Arab Em	Deaths	Male	2023	5.261431	7.727048	3.698042	
United Arab Em	Deaths	Female	2010	1.70071	2.430544	1.18192	
United Arab Em	Deaths	Female	2011	1.653315	2.331321	1.143083	
United Arab Em	Deaths	Female	2012	1.602164	2.179471	1.104421	
United Arab Em	Deaths	Female	2013	1.585056	2.188917	1.07593	
United Arab Em	Deaths	Female	2014	1.60446	2.179428	1.098213	
United Arab Em	Deaths	Female	2015	1.625453	2.168647	1.102933	
United Arab Em	Deaths	Female	2016	1.636817	2.192648	1.127459	

United Arab Em	Deaths	Female	2017	1.684067	2.257268	1.161931	
United Arab Em	Deaths	Female	2018	1.745398	2.32023	1.21473	
United Arab Em	Deaths	Female	2019	1.900424	2.52852	1.339561	
United Arab Em	Deaths	Female	2020	1.948702	2.65544	1.406902	
United Arab Em	Deaths	Female	2021	2.055676	2.742036	1.494878	
United Arab Em	Deaths	Female	2022	2.272672	3.083878	1.601031	
United Arab Em	Deaths	Female	2023	2.574334	3.4811	1.778547	
United Arab Em	DALYs	Male	2010	89.10802	124.6487	59.66815	
United Arab Em	DALYs	Male	2011	88.50749	122.7581	60.88431	
United Arab Em	DALYs	Male	2012	86.91643	118.9183	61.70853	
United Arab Em	DALYs	Male	2013	83.20726	110.1109	59.10918	
United Arab Em	DALYs	Male	2014	81.33552	108.6525	57.89214	
United Arab Em	DALYs	Male	2015	84.88946	114.8	60.42275	
United Arab Em	DALYs	Male	2016	88.70067	122.5284	63.52613	
United Arab Em	DALYs	Male	2017	94.37035	133.4923	68.64425	
United Arab Em	DALYs	Male	2018	98.94098	144.2591	71.28426	
United Arab Em	DALYs	Male	2019	106.6495	153.8461	76.4011	
United Arab Em	DALYs	Male	2020	90.94002	137.9873	65.68742	
United Arab Em	DALYs	Male	2021	96.91793	144.153	70.70389	
United Arab Em	DALYs	Male	2022	124.6293	184.5301	87.51082	
United Arab Em	DALYs	Male	2023	157.9129	229.7287	111.4114	
United Arab Em	DALYs	Female	2010	59.38285	85.1444	41.68016	
United Arab Em	DALYs	Female	2011	57.70733	81.66922	39.93492	
United Arab Em	DALYs	Female	2012	55.55286	77.28139	37.96995	
United Arab Em	DALYs	Female	2013	53.77044	74.82666	36.39125	
United Arab Em	DALYs	Female	2014	53.80518	73.335	36.20626	
United Arab Em	DALYs	Female	2015	54.16896	72.74343	36.75578	
United Arab Em	DALYs	Female	2016	54.26372	70.99689	36.91981	
United Arab Em	DALYs	Female	2017	55.06546	73.03349	37.54386	
United Arab Em	DALYs	Female	2018	55.96007	74.55297	39.09817	
United Arab Em	DALYs	Female	2019	59.387	79.69124	42.16067	
United Arab Em	DALYs	Female	2020	59.56659	82.63268	42.52229	
United Arab Em	DALYs	Female	2021	62.44284	84.51	45.34098	
United Arab Em	DALYs	Female	2022	69.6792	94.40723	48.9686	
United Arab Em	DALYs	Female	2023	78.51065	107.3019	54.62922	
United Kingdor	Incidence	Male	2010	83.22012	88.28911	78.90156	

United Kingdor	Incidence	Male	2011	83.7499	89.29352	79.24284	
United Kingdor	Incidence	Male	2012	83.16584	88.06702	78.22184	
United Kingdor	Incidence	Male	2013	83.94819	88.90579	78.26525	
United Kingdor	Incidence	Male	2014	83.18471	88.57909	77.20355	
United Kingdor	Incidence	Male	2015	81.13003	86.34154	75.63697	
United Kingdor	Incidence	Male	2016	80.22747	85.49444	74.9149	
United Kingdor	Incidence	Male	2017	79.27201	84.42009	73.68054	
United Kingdor	Incidence	Male	2018	78.58215	83.46293	72.7251	
United Kingdor	Incidence	Male	2019	76.83256	81.28659	71.16833	
United Kingdor	Incidence	Male	2020	76.05981	80.31611	70.91724	
United Kingdor	Incidence	Male	2021	72.74891	77.01062	67.84024	
United Kingdor	Incidence	Male	2022	73.96952	78.66436	68.7041	
United Kingdor	Incidence	Male	2023	79.44656	85.76281	73.73312	
United Kingdor	Incidence	Female	2010	66.89916	72.90583	60.88441	
United Kingdor	Incidence	Female	2011	67.20014	73.44065	61.07103	
United Kingdor	Incidence	Female	2012	69.58676	76.27156	62.80613	
United Kingdor	Incidence	Female	2013	69.89794	77.24391	62.37843	
United Kingdor	Incidence	Female	2014	69.80256	77.80103	61.87537	
United Kingdor	Incidence	Female	2015	70.41227	78.11772	62.31336	
United Kingdor	Incidence	Female	2016	69.74226	76.51253	62.1405	
United Kingdor	Incidence	Female	2017	69.69123	76.08269	62.07901	
United Kingdor	Incidence	Female	2018	69.49448	75.61497	61.63741	
United Kingdor	Incidence	Female	2019	68.33409	74.49669	60.3692	
United Kingdor	Incidence	Female	2020	68.76549	75.40972	61.44964	
United Kingdor	Incidence	Female	2021	67.31961	73.47209	59.61191	
United Kingdor	Incidence	Female	2022	69.25198	75.07654	61.74256	
United Kingdor	Incidence	Female	2023	74.4603	81.52023	66.52719	
United Kingdor	Deaths	Male	2010	69.12094	72.04015	65.83803	
United Kingdor	Deaths	Male	2011	69.17603	71.83433	66.00523	
United Kingdor	Deaths	Male	2012	68.34999	71.26242	64.48645	
United Kingdor	Deaths	Male	2013	68.65988	71.8827	64.89251	
United Kingdor	Deaths	Male	2014	67.89958	70.95969	63.83964	
United Kingdor	Deaths	Male	2015	66.37284	69.42708	62.36444	
United Kingdor	Deaths	Male	2016	65.77578	68.68167	61.74387	
United Kingdor	Deaths	Male	2017	65.05141	68.04048	61.25625	
United Kingdor	Deaths	Male	2018	64.34234	67.50284	60.55764	

United Kingdor	Deaths	Male	2019	62.6156	65.72961	58.49393	
United Kingdor	Deaths	Male	2020	61.65339	64.40197	58.36624	
United Kingdor	Deaths	Male	2021	58.77573	61.18584	55.46427	
United Kingdor	Deaths	Male	2022	59.84655	62.3948	56.0287	
United Kingdor	Deaths	Male	2023	64.27339	67.55146	60.79383	
United Kingdor	Deaths	Female	2010	54.30181	58.08262	49.36355	
United Kingdor	Deaths	Female	2011	54.22488	58.18692	48.92676	
United Kingdor	Deaths	Female	2012	55.89529	60.04027	50.40124	
United Kingdor	Deaths	Female	2013	55.85105	60.37841	50.22947	
United Kingdor	Deaths	Female	2014	55.52507	59.92447	49.68016	
United Kingdor	Deaths	Female	2015	56.1149	60.56565	50.36297	
United Kingdor	Deaths	Female	2016	55.63673	59.92337	50.19507	
United Kingdor	Deaths	Female	2017	55.6551	59.54131	49.89154	
United Kingdor	Deaths	Female	2018	55.28434	59.12061	49.54581	
United Kingdor	Deaths	Female	2019	54.00371	57.93946	48.32787	
United Kingdor	Deaths	Female	2020	53.99491	57.33447	48.84439	
United Kingdor	Deaths	Female	2021	52.66068	55.92188	47.61596	
United Kingdor	Deaths	Female	2022	54.22204	58.06414	48.60308	
United Kingdor	Deaths	Female	2023	58.26654	62.98385	52.17539	
United Kingdor	DALYs	Male	2010	1419.862	1475.317	1365.548	
United Kingdor	DALYs	Male	2011	1409.982	1460.274	1361.011	
United Kingdor	DALYs	Male	2012	1383.791	1434.066	1323.713	
United Kingdor	DALYs	Male	2013	1385.854	1442.492	1325.766	
United Kingdor	DALYs	Male	2014	1363.304	1416.467	1300.755	
United Kingdor	DALYs	Male	2015	1321.652	1373.131	1259.586	
United Kingdor	DALYs	Male	2016	1304.564	1355.763	1246.254	
United Kingdor	DALYs	Male	2017	1280.409	1327.902	1222.307	
United Kingdor	DALYs	Male	2018	1259.149	1306.587	1203.092	
United Kingdor	DALYs	Male	2019	1217.535	1264.936	1156.959	
United Kingdor	DALYs	Male	2020	1199.211	1243.847	1146.314	
United Kingdor	DALYs	Male	2021	1147.47	1188.702	1095.104	
United Kingdor	DALYs	Male	2022	1154.219	1198.142	1099.673	
United Kingdor	DALYs	Male	2023	1237.619	1295.99	1189.18	
United Kingdor	DALYs	Female	2010	1076.41	1137.591	995.6161	
United Kingdor	DALYs	Female	2011	1069.039	1132.743	990.1187	
United Kingdor	DALYs	Female	2012	1089.718	1161.336	1009.785	

United Kingdor	DALYs	Female	2013	1083.566	1155.224	998.1185	
United Kingdor	DALYs	Female	2014	1075.843	1151.738	989.4863	
United Kingdor	DALYs	Female	2015	1079.525	1154.529	993.2292	
United Kingdor	DALYs	Female	2016	1066.218	1132.134	981.4726	
United Kingdor	DALYs	Female	2017	1054.37	1113.844	970.5859	
United Kingdor	DALYs	Female	2018	1044.065	1101.748	957.4868	
United Kingdor	DALYs	Female	2019	1014.837	1077.251	930.5056	
United Kingdor	DALYs	Female	2020	1012.725	1065.977	936.0824	
United Kingdor	DALYs	Female	2021	988.0128	1041.759	913.0389	
United Kingdor	DALYs	Female	2022	1006.711	1067.848	927.3232	
United Kingdor	DALYs	Female	2023	1081.492	1154.016	987.3521	
Tanzania, Unite	Incidence	Male	2010	1.242814	1.700094	0.884529	
Tanzania, Unite	Incidence	Male	2011	1.274667	1.785408	0.89259	
Tanzania, Unite	Incidence	Male	2012	1.314132	1.779239	0.943842	
Tanzania, Unite	Incidence	Male	2013	1.320721	1.812983	0.948906	
Tanzania, Unite	Incidence	Male	2014	1.312435	1.795367	0.950954	
Tanzania, Unite	Incidence	Male	2015	1.317753	1.823484	0.948154	
Tanzania, Unite	Incidence	Male	2016	1.32462	1.848226	0.958206	
Tanzania, Unite	Incidence	Male	2017	1.3077	1.848016	0.940789	
Tanzania, Unite	Incidence	Male	2018	1.290648	1.84231	0.912422	
Tanzania, Unite	Incidence	Male	2019	1.273878	1.851128	0.875016	
Tanzania, Unite	Incidence	Male	2020	1.32282	1.967676	0.870985	
Tanzania, Unite	Incidence	Male	2021	1.452202	2.196173	0.9021	
Tanzania, Unite	Incidence	Male	2022	1.283219	1.873027	0.865184	
Tanzania, Unite	Incidence	Male	2023	1.363587	1.954668	0.922465	
Tanzania, Unite	Incidence	Female	2010	0.473413	0.666955	0.32757	
Tanzania, Unite	Incidence	Female	2011	0.480623	0.664063	0.329465	
Tanzania, Unite	Incidence	Female	2012	0.491566	0.687814	0.339376	
Tanzania, Unite	Incidence	Female	2013	0.49618	0.703314	0.335443	
Tanzania, Unite	Incidence	Female	2014	0.489923	0.69474	0.326553	
Tanzania, Unite	Incidence	Female	2015	0.486891	0.682172	0.324874	
Tanzania, Unite	Incidence	Female	2016	0.482796	0.681448	0.32459	
Tanzania, Unite	Incidence	Female	2017	0.47724	0.6511	0.327201	
Tanzania, Unite	Incidence	Female	2018	0.47067	0.631781	0.334545	
Tanzania, Unite	Incidence	Female	2019	0.464504	0.604175	0.328327	
Tanzania, Unite	Incidence	Female	2020	0.476872	0.650318	0.324135	

Tanzania, Unite	Incidence	Female	2021	0.556825	0.796729	0.340432	
Tanzania, Unite	Incidence	Female	2022	0.477522	0.658973	0.326689	
Tanzania, Unite	Incidence	Female	2023	0.514622	0.708667	0.349694	
Tanzania, Unite	Deaths	Male	2010	1.233402	1.676044	0.86414	
Tanzania, Unite	Deaths	Male	2011	1.265061	1.780151	0.879521	
Tanzania, Unite	Deaths	Male	2012	1.301492	1.766696	0.929315	
Tanzania, Unite	Deaths	Male	2013	1.306204	1.764248	0.939427	
Tanzania, Unite	Deaths	Male	2014	1.298623	1.765842	0.933082	
Tanzania, Unite	Deaths	Male	2015	1.304646	1.800076	0.934256	
Tanzania, Unite	Deaths	Male	2016	1.312337	1.822009	0.952047	
Tanzania, Unite	Deaths	Male	2017	1.296582	1.840801	0.92931	
Tanzania, Unite	Deaths	Male	2018	1.280659	1.829448	0.911699	
Tanzania, Unite	Deaths	Male	2019	1.265021	1.830945	0.869556	
Tanzania, Unite	Deaths	Male	2020	1.319241	1.957282	0.875267	
Tanzania, Unite	Deaths	Male	2021	1.451544	2.21056	0.916447	
Tanzania, Unite	Deaths	Male	2022	1.278319	1.877903	0.845806	
Tanzania, Unite	Deaths	Male	2023	1.354328	1.932991	0.908826	
Tanzania, Unite	Deaths	Female	2010	0.466492	0.656259	0.322347	
Tanzania, Unite	Deaths	Female	2011	0.473244	0.65696	0.326225	
Tanzania, Unite	Deaths	Female	2012	0.483005	0.679386	0.332755	
Tanzania, Unite	Deaths	Female	2013	0.485873	0.684988	0.328217	
Tanzania, Unite	Deaths	Female	2014	0.479627	0.684896	0.321059	
Tanzania, Unite	Deaths	Female	2015	0.477105	0.675481	0.317674	
Tanzania, Unite	Deaths	Female	2016	0.474194	0.675474	0.316707	
Tanzania, Unite	Deaths	Female	2017	0.470109	0.644335	0.323072	
Tanzania, Unite	Deaths	Female	2018	0.464819	0.626739	0.324373	
Tanzania, Unite	Deaths	Female	2019	0.459745	0.596797	0.326118	
Tanzania, Unite	Deaths	Female	2020	0.473265	0.643297	0.32299	
Tanzania, Unite	Deaths	Female	2021	0.553326	0.789563	0.341414	
Tanzania, Unite	Deaths	Female	2022	0.475164	0.658845	0.32703	
Tanzania, Unite	Deaths	Female	2023	0.509697	0.704423	0.343515	
Tanzania, Unite	DALYs	Male	2010	34.87962	49.07687	24.80871	
Tanzania, Unite	DALYs	Male	2011	35.75353	50.32341	25.40264	
Tanzania, Unite	DALYs	Male	2012	37.0488	50.97426	26.90788	
Tanzania, Unite	DALYs	Male	2013	37.37861	51.99918	27.0415	
Tanzania, Unite	DALYs	Male	2014	37.08365	51.87586	26.97338	

Tanzania, Unite	DALYs	Male	2015	37.13466	51.3411	27.4031	
Tanzania, Unite	DALYs	Male	2016	37.24478	51.8181	27.06059	
Tanzania, Unite	DALYs	Male	2017	36.68184	51.21839	26.2628	
Tanzania, Unite	DALYs	Male	2018	36.10851	51.76177	25.38145	
Tanzania, Unite	DALYs	Male	2019	35.52759	52.07244	24.1798	
Tanzania, Unite	DALYs	Male	2020	36.57849	54.10902	24.65696	
Tanzania, Unite	DALYs	Male	2021	39.87354	59.41019	25.06606	
Tanzania, Unite	DALYs	Male	2022	35.3403	50.78652	24.15578	
Tanzania, Unite	DALYs	Male	2023	37.71209	54.01032	25.42167	
Tanzania, Unite	DALYs	Female	2010	13.58671	19.29973	9.530401	
Tanzania, Unite	DALYs	Female	2011	13.81587	19.07154	9.669658	
Tanzania, Unite	DALYs	Female	2012	14.19831	19.62183	9.668898	
Tanzania, Unite	DALYs	Female	2013	14.46005	20.27784	9.692317	
Tanzania, Unite	DALYs	Female	2014	14.28202	20.05461	9.543735	
Tanzania, Unite	DALYs	Female	2015	14.14862	19.54069	9.432266	
Tanzania, Unite	DALYs	Female	2016	13.93681	19.48801	9.444832	
Tanzania, Unite	DALYs	Female	2017	13.66645	18.70384	9.433033	
Tanzania, Unite	DALYs	Female	2018	13.38531	18.03137	9.452033	
Tanzania, Unite	DALYs	Female	2019	13.12177	17.39967	9.17286	
Tanzania, Unite	DALYs	Female	2020	13.32899	18.28433	9.027255	
Tanzania, Unite	DALYs	Female	2021	15.35233	22.22438	9.536428	
Tanzania, Unite	DALYs	Female	2022	13.19354	18.26506	8.855392	
Tanzania, Unite	DALYs	Female	2023	14.35077	19.71028	9.714679	
United States o	Incidence	Male	2010	77.79335	84.17636	71.91287	
United States o	Incidence	Male	2011	77.69858	84.49643	72.19055	
United States o	Incidence	Male	2012	76.92884	83.60275	71.51925	
United States o	Incidence	Male	2013	76.2815	82.66745	70.50115	
United States o	Incidence	Male	2014	75.10094	81.23404	68.87905	
United States o	Incidence	Male	2015	72.08851	77.60386	66.26453	
United States o	Incidence	Male	2016	69.53111	75.3452	63.37476	
United States o	Incidence	Male	2017	67.99491	73.66826	61.53927	
United States o	Incidence	Male	2018	65.90506	71.78569	59.33277	
United States o	Incidence	Male	2019	64.38866	69.92116	58.00063	
United States o	Incidence	Male	2020	64.5227	69.84639	58.49496	
United States o	Incidence	Male	2021	62.81759	68.88515	57.10218	
United States o	Incidence	Male	2022	62.46586	69.51439	56.8888	

United States o	Incidence	Male	2023	60.21848	68.26023	54.05425	
United States o	Incidence	Female	2010	64.58426	70.98885	57.39928	
United States o	Incidence	Female	2011	64.57804	71.48979	57.38739	
United States o	Incidence	Female	2012	64.34511	71.25828	57.43319	
United States o	Incidence	Female	2013	64.17731	71.04829	57.2104	
United States o	Incidence	Female	2014	63.43503	70.69549	56.55228	
United States o	Incidence	Female	2015	62.01982	69.2862	54.65649	
United States o	Incidence	Female	2016	60.26352	67.09501	52.91166	
United States o	Incidence	Female	2017	59.4073	65.71073	52.15689	
United States o	Incidence	Female	2018	57.8823	63.92171	50.30201	
United States o	Incidence	Female	2019	56.62958	62.88852	49.1403	
United States o	Incidence	Female	2020	56.82599	62.13364	50.09072	
United States o	Incidence	Female	2021	56.0608	61.16418	49.71113	
United States o	Incidence	Female	2022	56.90405	62.59401	50.11161	
United States o	Incidence	Female	2023	56.9357	63.32936	49.93549	
United States o	Deaths	Male	2010	64.18981	67.25966	60.09205	
United States o	Deaths	Male	2011	64.23149	67.33639	60.03905	
United States o	Deaths	Male	2012	63.69466	67.1838	59.6367	
United States o	Deaths	Male	2013	63.30095	67.09081	59.28952	
United States o	Deaths	Male	2014	62.51101	66.54275	58.6134	
United States o	Deaths	Male	2015	60.26276	64.48587	56.521	
United States o	Deaths	Male	2016	58.31868	62.30851	54.64172	
United States o	Deaths	Male	2017	57.17855	61.05223	53.27906	
United States o	Deaths	Male	2018	55.48506	59.08439	51.46416	
United States o	Deaths	Male	2019	54.1854	58.17273	50.20114	
United States o	Deaths	Male	2020	54.10758	58.13015	50.43546	
United States o	Deaths	Male	2021	52.41938	57.04924	48.86823	
United States o	Deaths	Male	2022	52.04984	56.6413	48.42573	
United States o	Deaths	Male	2023	50.07015	55.5912	45.88462	
United States o	Deaths	Female	2010	50.79645	54.13413	45.15174	
United States o	Deaths	Female	2011	50.9383	54.46076	45.39776	
United States o	Deaths	Female	2012	50.831	54.25664	45.39408	
United States o	Deaths	Female	2013	50.80003	54.36521	45.44451	
United States o	Deaths	Female	2014	50.3847	54.04527	45.52782	
United States o	Deaths	Female	2015	49.56639	53.37309	44.3852	
United States o	Deaths	Female	2016	48.33765	52.28783	43.36785	

United States o	Deaths	Female	2017	47.77374	51.76983	43.04311	
United States o	Deaths	Female	2018	46.64635	50.76078	41.89609	
United States o	Deaths	Female	2019	45.62956	49.60064	40.81806	
United States o	Deaths	Female	2020	45.62039	49.0849	41.0638	
United States o	Deaths	Female	2021	44.71877	48.25151	40.26367	
United States o	Deaths	Female	2022	45.33668	49.05257	41.08635	
United States o	Deaths	Female	2023	45.26178	49.42996	40.86322	
United States o	DALYs	Male	2010	1431.527	1496.515	1358.324	
United States o	DALYs	Male	2011	1426.296	1493.394	1356.986	
United States o	DALYs	Male	2012	1409.675	1484.533	1343.938	
United States o	DALYs	Male	2013	1395.172	1477.051	1326.639	
United States o	DALYs	Male	2014	1374.08	1461.214	1302.672	
United States o	DALYs	Male	2015	1318.496	1406.638	1250.377	
United States o	DALYs	Male	2016	1270.463	1360.778	1202.086	
United States o	DALYs	Male	2017	1233.48	1317.353	1157.63	
United States o	DALYs	Male	2018	1189.951	1267.899	1114.569	
United States o	DALYs	Male	2019	1153.347	1240.546	1081.064	
United States o	DALYs	Male	2020	1149.939	1237.974	1078.266	
United States o	DALYs	Male	2021	1112.928	1213.585	1044.337	
United States o	DALYs	Male	2022	1096.943	1192.347	1027.335	
United States o	DALYs	Male	2023	1049.095	1167.53	968.4065	
United States o	DALYs	Female	2010	1081.21	1141.709	994.8743	
United States o	DALYs	Female	2011	1079.078	1139.136	991.706	
United States o	DALYs	Female	2012	1074.996	1136.303	993.241	
United States o	DALYs	Female	2013	1071.932	1137.742	992.1454	
United States o	DALYs	Female	2014	1061.147	1133.97	981.3747	
United States o	DALYs	Female	2015	1035.048	1111.986	951.4824	
United States o	DALYs	Female	2016	1006.746	1083.263	922.8144	
United States o	DALYs	Female	2017	987.8392	1064.907	906.0796	
United States o	DALYs	Female	2018	958.3741	1028.672	884.5208	
United States o	DALYs	Female	2019	930.2505	999.5631	849.8619	
United States o	DALYs	Female	2020	927.9536	995.6844	854.2146	
United States o	DALYs	Female	2021	911.2331	982.8707	840.0196	
United States o	DALYs	Female	2022	915.7065	990.1823	844.9084	
United States o	DALYs	Female	2023	911.5089	990.2232	833.364	
Uruguay	Incidence	Male	2010	79.66568	85.04864	74.50565	

Uruguay	Incidence	Male	2011	79.46003	85.62217	73.64497	
Uruguay	Incidence	Male	2012	77.17794	82.81434	72.08985	
Uruguay	Incidence	Male	2013	78.15113	83.57036	72.59086	
Uruguay	Incidence	Male	2014	77.82089	82.99125	72.89972	
Uruguay	Incidence	Male	2015	76.50907	81.67234	71.77191	
Uruguay	Incidence	Male	2016	76.5237	81.66457	71.03329	
Uruguay	Incidence	Male	2017	78.07692	83.86458	72.79113	
Uruguay	Incidence	Male	2018	78.80832	84.68569	73.01056	
Uruguay	Incidence	Male	2019	77.08375	82.65169	71.20385	
Uruguay	Incidence	Male	2020	73.87336	79.13676	68.00894	
Uruguay	Incidence	Male	2021	78.84659	84.70308	71.75845	
Uruguay	Incidence	Male	2022	90.04898	97.4917	81.61485	
Uruguay	Incidence	Male	2023	81.10622	90.3821	72.78057	
Uruguay	Incidence	Female	2010	20.63093	22.5572	18.63764	
Uruguay	Incidence	Female	2011	21.59792	23.5636	19.64749	
Uruguay	Incidence	Female	2012	21.90093	23.84653	20.19126	
Uruguay	Incidence	Female	2013	22.87122	25.13787	20.88868	
Uruguay	Incidence	Female	2014	23.75035	26.03791	21.73086	
Uruguay	Incidence	Female	2015	24.7492	26.80268	22.66244	
Uruguay	Incidence	Female	2016	25.67336	27.87304	23.69178	
Uruguay	Incidence	Female	2017	25.63814	27.59739	23.7466	
Uruguay	Incidence	Female	2018	27.21419	29.22158	25.02253	
Uruguay	Incidence	Female	2019	28.54818	30.58751	26.28184	
Uruguay	Incidence	Female	2020	28.67038	31.14943	26.13426	
Uruguay	Incidence	Female	2021	31.70438	34.83842	28.81236	
Uruguay	Incidence	Female	2022	36.07657	40.62136	32.60615	
Uruguay	Incidence	Female	2023	34.21728	38.89169	30.85669	
Uruguay	Deaths	Male	2010	78.87718	84.86654	73.60345	
Uruguay	Deaths	Male	2011	78.8352	84.59017	72.88752	
Uruguay	Deaths	Male	2012	76.74561	82.1245	71.87665	
Uruguay	Deaths	Male	2013	77.67537	82.91583	72.28301	
Uruguay	Deaths	Male	2014	77.39311	82.13119	73.003	
Uruguay	Deaths	Male	2015	75.96446	80.59407	71.80312	
Uruguay	Deaths	Male	2016	76.03793	81.1872	70.9775	
Uruguay	Deaths	Male	2017	77.77201	82.81313	72.77305	
Uruguay	Deaths	Male	2018	78.55164	84.07862	72.89982	

Uruguay	Deaths	Male	2019	76.78575	82.2133	71.27618	
Uruguay	Deaths	Male	2020	73.19464	78.44828	67.53719	
Uruguay	Deaths	Male	2021	78.31721	83.78159	71.66395	
Uruguay	Deaths	Male	2022	88.37297	95.43319	79.73062	
Uruguay	Deaths	Male	2023	79.23294	87.16731	71.00271	
Uruguay	Deaths	Female	2010	20.59363	22.63365	18.76531	
Uruguay	Deaths	Female	2011	21.5892	23.66649	19.58247	
Uruguay	Deaths	Female	2012	21.92775	23.79186	19.9924	
Uruguay	Deaths	Female	2013	22.85753	24.85419	20.75278	
Uruguay	Deaths	Female	2014	23.76298	25.91728	21.7163	
Uruguay	Deaths	Female	2015	24.75225	26.64006	22.66637	
Uruguay	Deaths	Female	2016	25.52784	27.33319	23.41011	
Uruguay	Deaths	Female	2017	25.48663	27.36959	23.47718	
Uruguay	Deaths	Female	2018	27.06201	28.75331	24.8829	
Uruguay	Deaths	Female	2019	28.3306	30.40281	25.9376	
Uruguay	Deaths	Female	2020	28.22406	30.46072	25.67013	
Uruguay	Deaths	Female	2021	31.3643	34.48179	28.59644	
Uruguay	Deaths	Female	2022	35.44847	39.95724	31.99839	
Uruguay	Deaths	Female	2023	33.48041	38.56881	30.33299	
Uruguay	DALYs	Male	2010	1887.515	2029.358	1768.694	
Uruguay	DALYs	Male	2011	1873.695	2021.437	1740.873	
Uruguay	DALYs	Male	2012	1815.831	1948.438	1705.114	
Uruguay	DALYs	Male	2013	1839.834	1972.879	1714.054	
Uruguay	DALYs	Male	2014	1825.732	1950.58	1717.736	
Uruguay	DALYs	Male	2015	1799.386	1914.275	1685.962	
Uruguay	DALYs	Male	2016	1787.028	1913.963	1672.709	
Uruguay	DALYs	Male	2017	1807.865	1926.355	1681.932	
Uruguay	DALYs	Male	2018	1816.147	1940.367	1685.154	
Uruguay	DALYs	Male	2019	1767.27	1889.877	1640.346	
Uruguay	DALYs	Male	2020	1692.746	1812.009	1558.088	
Uruguay	DALYs	Male	2021	1787.042	1913.651	1631.114	
Uruguay	DALYs	Male	2022	2083.28	2252.853	1863.308	
Uruguay	DALYs	Male	2023	1887.449	2078.621	1689.627	
Uruguay	DALYs	Female	2010	470.3353	512.8926	430.1193	
Uruguay	DALYs	Female	2011	492.0022	536.0819	452.0277	
Uruguay	DALYs	Female	2012	499.6014	542.3718	460.3153	

Uruguay	DALYs	Female	2013	520.5939	566.0209	479.9881	
Uruguay	DALYs	Female	2014	538.3051	585.9492	496.9937	
Uruguay	DALYs	Female	2015	557.8765	599.4632	516.7987	
Uruguay	DALYs	Female	2016	580.95	617.9585	539.654	
Uruguay	DALYs	Female	2017	578.5332	617.3468	538.4242	
Uruguay	DALYs	Female	2018	609.6741	647.2355	568.2036	
Uruguay	DALYs	Female	2019	636.4221	676.05	588.5995	
Uruguay	DALYs	Female	2020	637.9285	684.4969	587.4993	
Uruguay	DALYs	Female	2021	692.8795	757.2952	639.3403	
Uruguay	DALYs	Female	2022	793.0145	886.929	722.5402	
Uruguay	DALYs	Female	2023	756.0995	860.1662	682.4517	
Uzbekistan	Incidence	Male	2010	6.936134	7.57692	6.393465	
Uzbekistan	Incidence	Male	2011	7.995324	8.818059	7.261368	
Uzbekistan	Incidence	Male	2012	7.95867	8.665525	7.332237	
Uzbekistan	Incidence	Male	2013	8.322905	8.932977	7.722249	
Uzbekistan	Incidence	Male	2014	8.656262	9.228037	7.959104	
Uzbekistan	Incidence	Male	2015	8.173726	8.635875	7.667174	
Uzbekistan	Incidence	Male	2016	8.592809	9.014589	8.15459	
Uzbekistan	Incidence	Male	2017	8.709326	9.190009	8.283223	
Uzbekistan	Incidence	Male	2018	8.504242	8.931206	8.066147	
Uzbekistan	Incidence	Male	2019	8.912873	9.313855	8.406504	
Uzbekistan	Incidence	Male	2020	8.693833	9.284551	8.199436	
Uzbekistan	Incidence	Male	2021	8.738647	9.168933	8.271038	
Uzbekistan	Incidence	Male	2022	9.221008	9.785511	8.583017	
Uzbekistan	Incidence	Male	2023	9.476878	10.2135	8.653246	
Uzbekistan	Incidence	Female	2010	2.71462	3.092541	2.420884	
Uzbekistan	Incidence	Female	2011	2.748727	3.111994	2.46727	
Uzbekistan	Incidence	Female	2012	3.036666	3.410207	2.735595	
Uzbekistan	Incidence	Female	2013	3.050314	3.384676	2.76588	
Uzbekistan	Incidence	Female	2014	3.17163	3.541422	2.86746	
Uzbekistan	Incidence	Female	2015	3.073455	3.42619	2.784887	
Uzbekistan	Incidence	Female	2016	3.363344	3.710149	3.025334	
Uzbekistan	Incidence	Female	2017	3.47784	3.838944	3.11463	
Uzbekistan	Incidence	Female	2018	3.406952	3.77959	3.060018	
Uzbekistan	Incidence	Female	2019	3.311417	3.740936	2.94225	
Uzbekistan	Incidence	Female	2020	3.184835	3.65702	2.790988	

Uzbekistan	Incidence	Female	2021	3.131948	3.639796	2.720949	
Uzbekistan	Incidence	Female	2022	3.325524	3.917245	2.88041	
Uzbekistan	Incidence	Female	2023	3.432151	4.056507	2.88234	
Uzbekistan	Deaths	Male	2010	6.620689	7.24247	6.096314	
Uzbekistan	Deaths	Male	2011	7.656586	8.430697	6.948593	
Uzbekistan	Deaths	Male	2012	7.613651	8.288224	7.020282	
Uzbekistan	Deaths	Male	2013	7.952499	8.526518	7.361038	
Uzbekistan	Deaths	Male	2014	8.259419	8.811362	7.599089	
Uzbekistan	Deaths	Male	2015	7.794927	8.245102	7.322412	
Uzbekistan	Deaths	Male	2016	8.198732	8.60538	7.796986	
Uzbekistan	Deaths	Male	2017	8.346508	8.782093	7.938476	
Uzbekistan	Deaths	Male	2018	8.161537	8.541456	7.742082	
Uzbekistan	Deaths	Male	2019	8.538342	8.922762	8.054113	
Uzbekistan	Deaths	Male	2020	8.352678	8.938355	7.870152	
Uzbekistan	Deaths	Male	2021	8.407527	8.801474	7.972465	
Uzbekistan	Deaths	Male	2022	8.869914	9.407659	8.276401	
Uzbekistan	Deaths	Male	2023	9.121616	9.829465	8.361008	
Uzbekistan	Deaths	Female	2010	2.591583	2.95035	2.31311	
Uzbekistan	Deaths	Female	2011	2.639162	2.978699	2.371955	
Uzbekistan	Deaths	Female	2012	2.912442	3.258268	2.630014	
Uzbekistan	Deaths	Female	2013	2.918731	3.238415	2.649446	
Uzbekistan	Deaths	Female	2014	3.01158	3.372512	2.731932	
Uzbekistan	Deaths	Female	2015	2.919315	3.259324	2.644009	
Uzbekistan	Deaths	Female	2016	3.201156	3.528317	2.880464	
Uzbekistan	Deaths	Female	2017	3.31411	3.659745	2.977019	
Uzbekistan	Deaths	Female	2018	3.233346	3.586628	2.906405	
Uzbekistan	Deaths	Female	2019	3.154553	3.561213	2.802949	
Uzbekistan	Deaths	Female	2020	3.054458	3.514007	2.681475	
Uzbekistan	Deaths	Female	2021	3.009948	3.510347	2.606147	
Uzbekistan	Deaths	Female	2022	3.188692	3.762435	2.747425	
Uzbekistan	Deaths	Female	2023	3.292437	3.883535	2.767699	
Uzbekistan	DALYs	Male	2010	203.3139	222.5945	186.6733	
Uzbekistan	DALYs	Male	2011	232.7901	256.881	211.3918	
Uzbekistan	DALYs	Male	2012	232.3585	253.5897	214.8548	
Uzbekistan	DALYs	Male	2013	242.6213	261.1093	225.2971	
Uzbekistan	DALYs	Male	2014	252.682	269.8513	232.5654	

Uzbekistan	DALYs	Male	2015	237.8965	251.737	224.3079	
Uzbekistan	DALYs	Male	2016	249.6577	262.844	236.4529	
Uzbekistan	DALYs	Male	2017	249.4676	263.6036	237.2524	
Uzbekistan	DALYs	Male	2018	241.4344	252.7211	229.1609	
Uzbekistan	DALYs	Male	2019	252.1193	263.3689	237.6527	
Uzbekistan	DALYs	Male	2020	243.4729	259.7594	229.3839	
Uzbekistan	DALYs	Male	2021	243.0353	254.4463	230.2431	
Uzbekistan	DALYs	Male	2022	255.6452	270.8601	238.1928	
Uzbekistan	DALYs	Male	2023	261.3868	280.933	237.9221	
Uzbekistan	DALYs	Female	2010	81.47237	93.10369	72.20873	
Uzbekistan	DALYs	Female	2011	81.73458	92.13402	72.92026	
Uzbekistan	DALYs	Female	2012	90.27731	101.4458	80.92329	
Uzbekistan	DALYs	Female	2013	90.86178	101.4601	82.19574	
Uzbekistan	DALYs	Female	2014	95.89418	107.7774	85.77881	
Uzbekistan	DALYs	Female	2015	92.23276	102.9674	83.50668	
Uzbekistan	DALYs	Female	2016	99.69845	110.8512	89.2916	
Uzbekistan	DALYs	Female	2017	102.3711	113.3847	91.46154	
Uzbekistan	DALYs	Female	2018	100.4889	112.2318	89.66162	
Uzbekistan	DALYs	Female	2019	96.32003	109.3735	84.86396	
Uzbekistan	DALYs	Female	2020	91.29822	105.2803	80.086	
Uzbekistan	DALYs	Female	2021	89.0366	103.8527	77.15113	
Uzbekistan	DALYs	Female	2022	94.57972	112.2208	81.75317	
Uzbekistan	DALYs	Female	2023	97.01999	115.0073	81.90786	
Venezuela	Incidence	Male	2010	17.5732	18.73496	16.43786	
Venezuela	Incidence	Male	2011	17.81468	19.16356	16.49189	
Venezuela	Incidence	Male	2012	17.4695	18.83998	16.25112	
Venezuela	Incidence	Male	2013	17.66493	18.85314	16.3613	
Venezuela	Incidence	Male	2014	17.89451	19.18101	16.58064	
Venezuela	Incidence	Male	2015	17.63667	19.26117	16.32404	
Venezuela	Incidence	Male	2016	17.20201	18.86113	15.89202	
Venezuela	Incidence	Male	2017	17.86011	19.60417	16.4315	
Venezuela	Incidence	Male	2018	18.30243	20.57439	16.5247	
Venezuela	Incidence	Male	2019	20.60406	23.52893	18.24158	
Venezuela	Incidence	Male	2020	23.08276	26.45735	20.14617	
Venezuela	Incidence	Male	2021	24.48667	28.84572	20.9542	
Venezuela	Incidence	Male	2022	24.79361	29.97885	20.46832	

Venezuela	Incidence	Male	2023	28.30222	33.25112	23.54397	
Venezuela	Incidence	Female	2010	11.51417	12.42752	10.65523	
Venezuela	Incidence	Female	2011	11.91892	12.79601	10.93055	
Venezuela	Incidence	Female	2012	12.33337	13.41783	11.34707	
Venezuela	Incidence	Female	2013	12.75058	13.92108	11.65089	
Venezuela	Incidence	Female	2014	13.11928	14.30612	11.83452	
Venezuela	Incidence	Female	2015	13.4649	14.90705	12.17007	
Venezuela	Incidence	Female	2016	13.07336	14.56477	11.71402	
Venezuela	Incidence	Female	2017	13.17706	14.93003	11.68674	
Venezuela	Incidence	Female	2018	14.02179	16.29179	12.21117	
Venezuela	Incidence	Female	2019	15.49754	18.55808	13.17014	
Venezuela	Incidence	Female	2020	16.35037	20.26529	13.37287	
Venezuela	Incidence	Female	2021	16.4412	21.11902	13.21734	
Venezuela	Incidence	Female	2022	16.84014	22.81499	12.99178	
Venezuela	Incidence	Female	2023	17.32507	24.71233	13.21747	
Venezuela	Deaths	Male	2010	17.04161	18.15904	16.02913	
Venezuela	Deaths	Male	2011	17.25105	18.48628	16.04316	
Venezuela	Deaths	Male	2012	16.92387	18.22135	15.77229	
Venezuela	Deaths	Male	2013	17.12997	18.40386	15.82855	
Venezuela	Deaths	Male	2014	17.42675	18.76638	16.12821	
Venezuela	Deaths	Male	2015	17.25364	18.66761	16.08018	
Venezuela	Deaths	Male	2016	16.83674	18.3129	15.67215	
Venezuela	Deaths	Male	2017	17.466	19.10624	16.17984	
Venezuela	Deaths	Male	2018	17.90303	20.0462	16.26258	
Venezuela	Deaths	Male	2019	20.14871	22.83328	17.9424	
Venezuela	Deaths	Male	2020	22.62731	25.83437	19.8168	
Venezuela	Deaths	Male	2021	24.02805	27.99065	20.6893	
Venezuela	Deaths	Male	2022	24.38184	29.48238	20.28348	
Venezuela	Deaths	Male	2023	27.91381	33.05274	23.39341	
Venezuela	Deaths	Female	2010	10.46168	11.16295	9.702411	
Venezuela	Deaths	Female	2011	10.79071	11.54694	9.937184	
Venezuela	Deaths	Female	2012	11.14955	11.97777	10.26814	
Venezuela	Deaths	Female	2013	11.54707	12.31155	10.69885	
Venezuela	Deaths	Female	2014	11.95034	12.84308	11.00892	
Venezuela	Deaths	Female	2015	12.32126	13.3389	11.24768	
Venezuela	Deaths	Female	2016	11.97346	13.09084	10.87733	

Venezuela	Deaths	Female	2017	12.04859	13.46648	10.81522	
Venezuela	Deaths	Female	2018	12.83164	14.89588	11.25265	
Venezuela	Deaths	Female	2019	14.20327	17.07537	12.30325	
Venezuela	Deaths	Female	2020	15.02134	18.89104	12.5938	
Venezuela	Deaths	Female	2021	15.12657	19.56735	12.24123	
Venezuela	Deaths	Female	2022	15.58682	21.16228	12.18466	
Venezuela	Deaths	Female	2023	16.15469	23.04024	12.38617	
Venezuela	DALYs	Male	2010	452.0304	483.7936	424.0454	
Venezuela	DALYs	Male	2011	457.5293	493.7382	423.59	
Venezuela	DALYs	Male	2012	447.107	483.1447	414.2089	
Venezuela	DALYs	Male	2013	449.0232	484.0062	415.7844	
Venezuela	DALYs	Male	2014	451.6589	490.0487	418.0255	
Venezuela	DALYs	Male	2015	442.3355	482.9805	410.3802	
Venezuela	DALYs	Male	2016	432.6253	471.8162	401.4748	
Venezuela	DALYs	Male	2017	449.3571	492.9764	411.4314	
Venezuela	DALYs	Male	2018	458.7522	516.8787	413.1398	
Venezuela	DALYs	Male	2019	514.5632	586.5686	456.2417	
Venezuela	DALYs	Male	2020	570.5572	655.9893	496.6264	
Venezuela	DALYs	Male	2021	602.4535	710.3282	511.9203	
Venezuela	DALYs	Male	2022	609.9119	740.5982	497.5974	
Venezuela	DALYs	Male	2023	694.1157	824.1594	576.1719	
Venezuela	DALYs	Female	2010	271.5583	289.7428	252.3454	
Venezuela	DALYs	Female	2011	279.3746	298.7107	257.9736	
Venezuela	DALYs	Female	2012	288.1673	310.0406	268.2306	
Venezuela	DALYs	Female	2013	294.6235	318.2979	272.0115	
Venezuela	DALYs	Female	2014	300.8735	324.8013	275.4585	
Venezuela	DALYs	Female	2015	308.9702	335.4931	281.5709	
Venezuela	DALYs	Female	2016	302.2599	331.7016	273.8506	
Venezuela	DALYs	Female	2017	305.4678	343.6783	272.2029	
Venezuela	DALYs	Female	2018	323.3376	377.6723	284.8391	
Venezuela	DALYs	Female	2019	354.6012	427.6535	305.9519	
Venezuela	DALYs	Female	2020	371.894	468.2678	308.3709	
Venezuela	DALYs	Female	2021	374.1736	484.2702	303.9261	
Venezuela	DALYs	Female	2022	383.0933	521.2772	298.0809	
Venezuela	DALYs	Female	2023	392.984	559.1896	302.2801	
Viet Nam	Incidence	Male	2010	20.46446	26.30879	13.94878	

Viet Nam	Incidence	Male	2011	21.19981	26.72344	14.10814	
Viet Nam	Incidence	Male	2012	22.26544	28.24234	14.41519	
Viet Nam	Incidence	Male	2013	23.33028	29.94967	15.06658	
Viet Nam	Incidence	Male	2014	24.13535	31.30591	15.55652	
Viet Nam	Incidence	Male	2015	25.15165	32.10256	16.51862	
Viet Nam	Incidence	Male	2016	26.45676	32.63233	17.09924	
Viet Nam	Incidence	Male	2017	27.41232	33.26346	18.34389	
Viet Nam	Incidence	Male	2018	28.73734	34.71196	19.17486	
Viet Nam	Incidence	Male	2019	30.12346	36.7941	19.90525	
Viet Nam	Incidence	Male	2020	31.42766	39.0971	20.28046	
Viet Nam	Incidence	Male	2021	31.77426	39.77406	20.68522	
Viet Nam	Incidence	Male	2022	34.3754	44.89209	21.55055	
Viet Nam	Incidence	Male	2023	36.35535	47.7143	22.8815	
Viet Nam	Incidence	Female	2010	9.630191	12.95654	6.913207	
Viet Nam	Incidence	Female	2011	9.948454	13.33313	7.163681	
Viet Nam	Incidence	Female	2012	10.31466	13.70807	7.285517	
Viet Nam	Incidence	Female	2013	10.6995	14.08699	7.431961	
Viet Nam	Incidence	Female	2014	11.07726	14.96253	7.787763	
Viet Nam	Incidence	Female	2015	11.5312	16.05788	7.869681	
Viet Nam	Incidence	Female	2016	12.08982	17.10919	8.109477	
Viet Nam	Incidence	Female	2017	12.49881	16.94872	8.588597	
Viet Nam	Incidence	Female	2018	13.02479	17.91671	9.070551	
Viet Nam	Incidence	Female	2019	13.61023	18.82046	9.534138	
Viet Nam	Incidence	Female	2020	14.12192	19.2597	9.815665	
Viet Nam	Incidence	Female	2021	14.29863	19.76541	9.715417	
Viet Nam	Incidence	Female	2022	15.36933	21.25305	10.352	
Viet Nam	Incidence	Female	2023	16.45468	23.32055	11.08058	
Viet Nam	Deaths	Male	2010	19.73476	25.12057	13.74508	
Viet Nam	Deaths	Male	2011	20.39292	25.70932	13.8885	
Viet Nam	Deaths	Male	2012	21.36636	26.92823	14.2053	
Viet Nam	Deaths	Male	2013	22.33746	28.61069	14.81118	
Viet Nam	Deaths	Male	2014	23.07911	29.71359	15.09506	
Viet Nam	Deaths	Male	2015	23.98673	30.51112	15.82463	
Viet Nam	Deaths	Male	2016	25.18519	30.97571	16.3967	
Viet Nam	Deaths	Male	2017	26.05103	31.51349	17.62096	
Viet Nam	Deaths	Male	2018	27.27009	32.94377	18.34854	

Viet Nam	Deaths	Male	2019	28.54454	34.84334	18.95532	
Viet Nam	Deaths	Male	2020	29.73511	36.52154	19.4084	
Viet Nam	Deaths	Male	2021	30.05663	37.63872	20.00054	
Viet Nam	Deaths	Male	2022	32.44442	42.11947	20.64229	
Viet Nam	Deaths	Male	2023	34.3012	44.74705	21.98331	
Viet Nam	Deaths	Female	2010	9.181936	12.26251	6.697448	
Viet Nam	Deaths	Female	2011	9.460102	12.66075	6.863554	
Viet Nam	Deaths	Female	2012	9.784041	12.97324	7.001545	
Viet Nam	Deaths	Female	2013	10.12477	13.43739	7.020776	
Viet Nam	Deaths	Female	2014	10.46302	14.25869	7.285396	
Viet Nam	Deaths	Female	2015	10.85892	15.15786	7.393707	
Viet Nam	Deaths	Female	2016	11.35401	15.83557	7.698792	
Viet Nam	Deaths	Female	2017	11.71152	15.87765	8.061179	
Viet Nam	Deaths	Female	2018	12.17898	16.53786	8.537558	
Viet Nam	Deaths	Female	2019	12.69415	17.1252	9.038724	
Viet Nam	Deaths	Female	2020	13.13593	17.60648	9.110964	
Viet Nam	Deaths	Female	2021	13.2615	18.3318	9.129354	
Viet Nam	Deaths	Female	2022	14.20867	19.6381	9.627793	
Viet Nam	Deaths	Female	2023	15.19781	21.40499	10.18897	
Viet Nam	DALYs	Male	2010	568.6197	724.3462	380.8573	
Viet Nam	DALYs	Male	2011	590.2959	743.6181	385.8668	
Viet Nam	DALYs	Male	2012	620.7098	789.2394	399.2899	
Viet Nam	DALYs	Male	2013	651.3802	849.8977	413.9973	
Viet Nam	DALYs	Male	2014	673.3982	875.2815	429.1672	
Viet Nam	DALYs	Male	2015	700.868	903.1004	445.6524	
Viet Nam	DALYs	Male	2016	735.928	911.747	467.3733	
Viet Nam	DALYs	Male	2017	761.1711	926.5391	496.1799	
Viet Nam	DALYs	Male	2018	796.8203	970.4726	522.9915	
Viet Nam	DALYs	Male	2019	833.2464	1026.284	533.748	
Viet Nam	DALYs	Male	2020	865.808	1081.702	545.8202	
Viet Nam	DALYs	Male	2021	869.4922	1093.637	542.0171	
Viet Nam	DALYs	Male	2022	937.8848	1211.921	568.782	
Viet Nam	DALYs	Male	2023	987.7227	1306.444	607.4361	
Viet Nam	DALYs	Female	2010	261.8451	354.9883	185.3568	
Viet Nam	DALYs	Female	2011	270.6332	363.2329	189.31	
Viet Nam	DALYs	Female	2012	280.5018	371.6407	192.889	

Viet Nam	DALYs	Female	2013	291.0685	381.3514	198.43	
Viet Nam	DALYs	Female	2014	300.923	403.026	204.7623	
Viet Nam	DALYs	Female	2015	312.3809	433.841	211.9318	
Viet Nam	DALYs	Female	2016	326.3708	457.8598	217.6477	
Viet Nam	DALYs	Female	2017	336.4746	454.0406	229.4976	
Viet Nam	DALYs	Female	2018	349.5944	482.1239	242.8394	
Viet Nam	DALYs	Female	2019	364.0481	490.6435	253.1231	
Viet Nam	DALYs	Female	2020	375.5738	511.5968	257.7794	
Viet Nam	DALYs	Female	2021	377.8612	520.9112	255.2337	
Viet Nam	DALYs	Female	2022	404.0021	553.4448	272.7073	
Viet Nam	DALYs	Female	2023	429.5907	602.666	287.5831	
Yemen	Incidence	Male	2010	6.143808	9.227407	4.142892	
Yemen	Incidence	Male	2011	6.224261	9.28281	4.286337	
Yemen	Incidence	Male	2012	6.268344	9.556536	4.360986	
Yemen	Incidence	Male	2013	6.308528	9.56602	4.398088	
Yemen	Incidence	Male	2014	6.192054	9.248073	4.285591	
Yemen	Incidence	Male	2015	6.336222	9.431905	4.356061	
Yemen	Incidence	Male	2016	6.208995	9.427421	4.127765	
Yemen	Incidence	Male	2017	6.087424	9.078664	4.120329	
Yemen	Incidence	Male	2018	6.001027	8.868086	3.908886	
Yemen	Incidence	Male	2019	6.209053	9.560639	4.018174	
Yemen	Incidence	Male	2020	6.474359	9.977313	4.372646	
Yemen	Incidence	Male	2021	6.365575	9.800732	4.319889	
Yemen	Incidence	Male	2022	6.555984	9.989628	4.295737	
Yemen	Incidence	Male	2023	6.602378	10.30796	4.299256	
Yemen	Incidence	Female	2010	1.542785	2.449081	1.022587	
Yemen	Incidence	Female	2011	1.598195	2.440701	1.069883	
Yemen	Incidence	Female	2012	1.617503	2.627538	1.094481	
Yemen	Incidence	Female	2013	1.67799	2.734952	1.074317	
Yemen	Incidence	Female	2014	1.710142	2.740231	1.082425	
Yemen	Incidence	Female	2015	1.744235	2.679823	1.103001	
Yemen	Incidence	Female	2016	1.735415	2.658546	1.134913	
Yemen	Incidence	Female	2017	1.746222	2.685736	1.131021	
Yemen	Incidence	Female	2018	1.712495	2.641011	1.102455	
Yemen	Incidence	Female	2019	1.736706	2.697724	1.113687	
Yemen	Incidence	Female	2020	1.730982	2.647511	1.083308	

Yemen	Incidence	Female	2021	1.752462	2.629139	1.077461	
Yemen	Incidence	Female	2022	1.908088	2.929583	1.223192	
Yemen	Incidence	Female	2023	2.026562	3.168248	1.248724	
Yemen	Deaths	Male	2010	6.089046	9.129264	4.101764	
Yemen	Deaths	Male	2011	6.169982	9.186298	4.227217	
Yemen	Deaths	Male	2012	6.216553	9.424161	4.318795	
Yemen	Deaths	Male	2013	6.257419	9.486868	4.369028	
Yemen	Deaths	Male	2014	6.142996	9.177567	4.277786	
Yemen	Deaths	Male	2015	6.286657	9.369982	4.355222	
Yemen	Deaths	Male	2016	6.163382	9.357794	4.111091	
Yemen	Deaths	Male	2017	6.051576	9.002015	4.082223	
Yemen	Deaths	Male	2018	5.972882	8.737334	3.879713	
Yemen	Deaths	Male	2019	6.181328	9.417063	4.005238	
Yemen	Deaths	Male	2020	6.461616	9.917225	4.390757	
Yemen	Deaths	Male	2021	6.344875	9.748672	4.312614	
Yemen	Deaths	Male	2022	6.523573	9.844055	4.268812	
Yemen	Deaths	Male	2023	6.571102	10.26542	4.266879	
Yemen	Deaths	Female	2010	1.522419	2.407549	1.013848	
Yemen	Deaths	Female	2011	1.575347	2.392022	1.053493	
Yemen	Deaths	Female	2012	1.592687	2.563315	1.07136	
Yemen	Deaths	Female	2013	1.649841	2.66983	1.059188	
Yemen	Deaths	Female	2014	1.680119	2.686196	1.064114	
Yemen	Deaths	Female	2015	1.711671	2.622533	1.088937	
Yemen	Deaths	Female	2016	1.704127	2.598299	1.117053	
Yemen	Deaths	Female	2017	1.712305	2.632405	1.114282	
Yemen	Deaths	Female	2018	1.681337	2.574099	1.086831	
Yemen	Deaths	Female	2019	1.709584	2.630248	1.095443	
Yemen	Deaths	Female	2020	1.706219	2.588561	1.063438	
Yemen	Deaths	Female	2021	1.72739	2.575472	1.056103	
Yemen	Deaths	Female	2022	1.876179	2.903086	1.210404	
Yemen	Deaths	Female	2023	1.988063	3.080266	1.234852	
Yemen	DALYs	Male	2010	166.6861	250.9785	112.5635	
Yemen	DALYs	Male	2011	168.5371	254.4225	115.4929	
Yemen	DALYs	Male	2012	169.4855	259.3059	117.9219	
Yemen	DALYs	Male	2013	170.2438	256.1241	118.8555	
Yemen	DALYs	Male	2014	166.7621	249.3483	116.079	

Yemen	DALYs	Male	2015	170.382	260.7111	115.0127	
Yemen	DALYs	Male	2016	166.7747	255.6793	110.1787	
Yemen	DALYs	Male	2017	163.2408	244.4256	110.1187	
Yemen	DALYs	Male	2018	160.9715	240.7551	104.0326	
Yemen	DALYs	Male	2019	166.5421	256.5095	108.4897	
Yemen	DALYs	Male	2020	172.2809	266.0539	116.2007	
Yemen	DALYs	Male	2021	169.8817	262.3206	115.3522	
Yemen	DALYs	Male	2022	175.4596	268.493	115.6405	
Yemen	DALYs	Male	2023	176.4385	274.6472	114.446	
Yemen	DALYs	Female	2010	42.60964	66.52803	28.12813	
Yemen	DALYs	Female	2011	44.14923	68.31771	29.37432	
Yemen	DALYs	Female	2012	44.7325	73.89677	30.49845	
Yemen	DALYs	Female	2013	46.42483	76.12839	29.9954	
Yemen	DALYs	Female	2014	47.24991	75.63969	30.25498	
Yemen	DALYs	Female	2015	48.17646	74.29168	30.2603	
Yemen	DALYs	Female	2016	47.82102	73.07135	31.06521	
Yemen	DALYs	Female	2017	48.37894	75.52952	31.13125	
Yemen	DALYs	Female	2018	47.45774	74.18548	30.72732	
Yemen	DALYs	Female	2019	47.88221	75.07363	30.54303	
Yemen	DALYs	Female	2020	47.47217	72.90091	30.50643	
Yemen	DALYs	Female	2021	47.95334	72.30172	29.49268	
Yemen	DALYs	Female	2022	52.37123	78.74116	33.21077	
Yemen	DALYs	Female	2023	55.83857	87.84703	34.3101	
Zambia	Incidence	Male	2010	1.260195	1.747553	0.919906	
Zambia	Incidence	Male	2011	1.302946	1.769994	0.947239	
Zambia	Incidence	Male	2012	1.326034	1.819322	0.93949	
Zambia	Incidence	Male	2013	1.333649	1.85965	0.929362	
Zambia	Incidence	Male	2014	1.357874	1.875107	0.921938	
Zambia	Incidence	Male	2015	1.376531	1.911557	0.952757	
Zambia	Incidence	Male	2016	1.402589	1.982121	0.982032	
Zambia	Incidence	Male	2017	1.399853	1.949474	0.966224	
Zambia	Incidence	Male	2018	1.397428	1.986625	0.945951	
Zambia	Incidence	Male	2019	1.415064	2.03995	0.932116	
Zambia	Incidence	Male	2020	1.533753	2.237322	1.005426	
Zambia	Incidence	Male	2021	1.893358	2.800584	1.23657	
Zambia	Incidence	Male	2022	1.500243	2.17587	0.967398	

Zambia	Incidence	Male	2023	1.54518	2.261845	1.025028	
Zambia	Incidence	Female	2010	0.877172	1.237406	0.59927	
Zambia	Incidence	Female	2011	0.899131	1.250271	0.6236	
Zambia	Incidence	Female	2012	0.902491	1.245461	0.614826	
Zambia	Incidence	Female	2013	0.898129	1.250673	0.614276	
Zambia	Incidence	Female	2014	0.900363	1.276064	0.62166	
Zambia	Incidence	Female	2015	0.9166	1.292377	0.621271	
Zambia	Incidence	Female	2016	0.940203	1.295634	0.639113	
Zambia	Incidence	Female	2017	0.959456	1.274156	0.665148	
Zambia	Incidence	Female	2018	0.968072	1.289941	0.680124	
Zambia	Incidence	Female	2019	0.998492	1.327539	0.702829	
Zambia	Incidence	Female	2020	1.034851	1.376876	0.723924	
Zambia	Incidence	Female	2021	1.158478	1.56176	0.83263	
Zambia	Incidence	Female	2022	1.206407	1.665347	0.848541	
Zambia	Incidence	Female	2023	1.298369	1.820353	0.927614	
Zambia	Deaths	Male	2010	1.200247	1.672227	0.87	
Zambia	Deaths	Male	2011	1.237253	1.677689	0.892613	
Zambia	Deaths	Male	2012	1.255995	1.721351	0.894707	
Zambia	Deaths	Male	2013	1.260398	1.762099	0.880836	
Zambia	Deaths	Male	2014	1.280798	1.772362	0.872494	
Zambia	Deaths	Male	2015	1.297481	1.793242	0.902254	
Zambia	Deaths	Male	2016	1.32115	1.863065	0.923891	
Zambia	Deaths	Male	2017	1.318521	1.831502	0.910743	
Zambia	Deaths	Male	2018	1.316684	1.869698	0.891751	
Zambia	Deaths	Male	2019	1.333424	1.928348	0.877235	
Zambia	Deaths	Male	2020	1.446563	2.098545	0.948945	
Zambia	Deaths	Male	2021	1.787763	2.651958	1.165711	
Zambia	Deaths	Male	2022	1.418104	2.047332	0.91652	
Zambia	Deaths	Male	2023	1.460179	2.128805	0.963772	
Zambia	Deaths	Female	2010	0.825123	1.162867	0.565891	
Zambia	Deaths	Female	2011	0.845593	1.174174	0.587134	
Zambia	Deaths	Female	2012	0.84894	1.169768	0.579807	
Zambia	Deaths	Female	2013	0.845678	1.179764	0.5802	
Zambia	Deaths	Female	2014	0.84826	1.202077	0.588353	
Zambia	Deaths	Female	2015	0.863442	1.217444	0.590467	
Zambia	Deaths	Female	2016	0.885332	1.21378	0.60189	

Zambia	Deaths	Female	2017	0.903629	1.196362	0.622806	
Zambia	Deaths	Female	2018	0.911528	1.217045	0.642579	
Zambia	Deaths	Female	2019	0.939462	1.243099	0.658656	
Zambia	Deaths	Female	2020	0.972063	1.288814	0.679168	
Zambia	Deaths	Female	2021	1.086282	1.464709	0.781418	
Zambia	Deaths	Female	2022	1.126045	1.558013	0.792378	
Zambia	Deaths	Female	2023	1.208442	1.687748	0.86993	
Zambia	DALYs	Male	2010	38.96408	53.33834	28.64479	
Zambia	DALYs	Male	2011	40.56408	55.45188	29.43358	
Zambia	DALYs	Male	2012	41.51806	56.77296	29.2241	
Zambia	DALYs	Male	2013	41.95497	57.53644	29.39531	
Zambia	DALYs	Male	2014	42.87793	59.07481	29.32558	
Zambia	DALYs	Male	2015	43.47696	60.35116	30.0274	
Zambia	DALYs	Male	2016	44.32217	62.64447	31.21578	
Zambia	DALYs	Male	2017	44.1972	61.75407	30.50461	
Zambia	DALYs	Male	2018	44.04009	63.06827	29.81853	
Zambia	DALYs	Male	2019	44.53759	64.65307	29.21409	
Zambia	DALYs	Male	2020	48.02648	70.75707	31.33795	
Zambia	DALYs	Male	2021	58.78527	86.72314	38.7763	
Zambia	DALYs	Male	2022	46.65545	68.08596	29.83731	
Zambia	DALYs	Male	2023	47.98946	69.81994	32.28436	
Zambia	DALYs	Female	2010	28.10652	39.76558	19.09712	
Zambia	DALYs	Female	2011	28.78349	40.08724	19.9962	
Zambia	DALYs	Female	2012	28.83825	39.99625	19.75937	
Zambia	DALYs	Female	2013	28.58945	39.60126	19.50435	
Zambia	DALYs	Female	2014	28.58594	40.55895	19.43721	
Zambia	DALYs	Female	2015	29.04785	41.108	19.60067	
Zambia	DALYs	Female	2016	29.78546	41.23868	20.27946	
Zambia	DALYs	Female	2017	30.34464	40.37433	21.10641	
Zambia	DALYs	Female	2018	30.59665	40.53041	21.24916	
Zambia	DALYs	Female	2019	31.55342	42.16914	22.25892	
Zambia	DALYs	Female	2020	32.70655	44.1471	22.86541	
Zambia	DALYs	Female	2021	36.58531	49.66386	26.26415	
Zambia	DALYs	Female	2022	38.59776	53.25104	26.86432	
Zambia	DALYs	Female	2023	41.77592	59.73459	29.65798	
Zimbabwe	Incidence	Male	2010	9.177285	12.57378	6.691948	

Zimbabwe	Incidence	Male	2011	9.504558	13.02239	6.918858	
Zimbabwe	Incidence	Male	2012	9.811771	13.51595	7.176192	
Zimbabwe	Incidence	Male	2013	9.882365	13.77473	7.229126	
Zimbabwe	Incidence	Male	2014	9.708648	13.39365	6.972277	
Zimbabwe	Incidence	Male	2015	9.821844	13.91008	6.857421	
Zimbabwe	Incidence	Male	2016	10.17681	14.16481	7.065416	
Zimbabwe	Incidence	Male	2017	10.07067	14.22238	6.911526	
Zimbabwe	Incidence	Male	2018	10.31377	14.70964	6.9001	
Zimbabwe	Incidence	Male	2019	10.43296	14.56729	7.147622	
Zimbabwe	Incidence	Male	2020	10.62222	14.43862	7.501199	
Zimbabwe	Incidence	Male	2021	13.33935	18.1676	9.705011	
Zimbabwe	Incidence	Male	2022	10.59406	14.61027	7.374911	
Zimbabwe	Incidence	Male	2023	10.69755	14.67699	7.505049	
Zimbabwe	Incidence	Female	2010	2.699801	3.822924	1.772465	
Zimbabwe	Incidence	Female	2011	2.90176	4.030896	1.947442	
Zimbabwe	Incidence	Female	2012	2.980848	4.038624	1.993542	
Zimbabwe	Incidence	Female	2013	2.983925	4.105439	1.967722	
Zimbabwe	Incidence	Female	2014	2.977069	4.051413	1.940912	
Zimbabwe	Incidence	Female	2015	3.081806	4.187547	2.035422	
Zimbabwe	Incidence	Female	2016	3.192554	4.340707	2.156424	
Zimbabwe	Incidence	Female	2017	3.214954	4.354703	2.129389	
Zimbabwe	Incidence	Female	2018	3.347252	4.451169	2.293517	
Zimbabwe	Incidence	Female	2019	3.432138	4.592983	2.389158	
Zimbabwe	Incidence	Female	2020	3.473676	4.556319	2.38475	
Zimbabwe	Incidence	Female	2021	4.150312	5.256276	2.871418	
Zimbabwe	Incidence	Female	2022	3.617951	4.780598	2.403473	
Zimbabwe	Incidence	Female	2023	3.704627	5.021325	2.475219	
Zimbabwe	Deaths	Male	2010	9.149245	12.26144	6.683366	
Zimbabwe	Deaths	Male	2011	9.464194	12.76423	6.809477	
Zimbabwe	Deaths	Male	2012	9.766871	13.29993	7.124326	
Zimbabwe	Deaths	Male	2013	9.843231	13.70121	7.234788	
Zimbabwe	Deaths	Male	2014	9.691662	13.36831	6.880301	
Zimbabwe	Deaths	Male	2015	9.805731	13.84151	6.851193	
Zimbabwe	Deaths	Male	2016	10.15727	13.96035	7.052153	
Zimbabwe	Deaths	Male	2017	10.05574	14.00781	6.966122	
Zimbabwe	Deaths	Male	2018	10.2903	14.40973	6.938518	

Zimbabwe	Deaths	Male	2019	10.41043	14.37278	7.123267	
Zimbabwe	Deaths	Male	2020	10.63486	14.49498	7.528925	
Zimbabwe	Deaths	Male	2021	13.52978	18.41009	9.704094	
Zimbabwe	Deaths	Male	2022	10.6068	14.62633	7.465292	
Zimbabwe	Deaths	Male	2023	10.7376	14.68845	7.486569	
Zimbabwe	Deaths	Female	2010	2.73468	3.912099	1.817004	
Zimbabwe	Deaths	Female	2011	2.926125	4.067535	1.959299	
Zimbabwe	Deaths	Female	2012	3.008014	4.119033	2.009172	
Zimbabwe	Deaths	Female	2013	3.019394	4.178647	1.999903	
Zimbabwe	Deaths	Female	2014	3.023652	4.138283	1.960916	
Zimbabwe	Deaths	Female	2015	3.127512	4.295394	2.055481	
Zimbabwe	Deaths	Female	2016	3.240763	4.439254	2.152162	
Zimbabwe	Deaths	Female	2017	3.268074	4.44702	2.160106	
Zimbabwe	Deaths	Female	2018	3.398556	4.564241	2.297716	
Zimbabwe	Deaths	Female	2019	3.48631	4.707477	2.349786	
Zimbabwe	Deaths	Female	2020	3.5226	4.617245	2.408163	
Zimbabwe	Deaths	Female	2021	4.171327	5.312382	2.911396	
Zimbabwe	Deaths	Female	2022	3.684404	4.855914	2.451468	
Zimbabwe	Deaths	Female	2023	3.785558	5.153848	2.524265	
Zimbabwe	DALYs	Male	2010	256.1732	354.0689	183.8897	
Zimbabwe	DALYs	Male	2011	266.0132	373.8805	192.1031	
Zimbabwe	DALYs	Male	2012	274.5369	382.386	198.6938	
Zimbabwe	DALYs	Male	2013	275.7709	394.4897	196.5917	
Zimbabwe	DALYs	Male	2014	269.3848	386.8316	191.2618	
Zimbabwe	DALYs	Male	2015	272.1874	387.2551	190.0049	
Zimbabwe	DALYs	Male	2016	281.8362	388.5305	193.0583	
Zimbabwe	DALYs	Male	2017	278.3193	393.1954	190.9708	
Zimbabwe	DALYs	Male	2018	284.6642	406.8315	188.8928	
Zimbabwe	DALYs	Male	2019	287.5144	410.1002	194.6454	
Zimbabwe	DALYs	Male	2020	291.2253	401.7244	204.9127	
Zimbabwe	DALYs	Male	2021	357.3605	487.3909	254.0682	
Zimbabwe	DALYs	Male	2022	289.3938	407.0413	196.5359	
Zimbabwe	DALYs	Male	2023	289.5782	403.1862	202.8126	
Zimbabwe	DALYs	Female	2010	75.82113	107.819	51.05866	
Zimbabwe	DALYs	Female	2011	82.02448	113.3012	55.57697	
Zimbabwe	DALYs	Female	2012	83.91793	112.9026	56.22952	

Zimbabwe	DALYs	Female	2013	83.25518	112.5103	53.79349	
Zimbabwe	DALYs	Female	2014	82.19481	110.2651	52.03406	
Zimbabwe	DALYs	Female	2015	84.92668	114.9526	54.91746	
Zimbabwe	DALYs	Female	2016	87.63207	117.9438	57.76168	
Zimbabwe	DALYs	Female	2017	87.7257	118.7486	58.49971	
Zimbabwe	DALYs	Female	2018	91.02066	121.721	62.65805	
Zimbabwe	DALYs	Female	2019	93.01217	123.5296	65.65438	
Zimbabwe	DALYs	Female	2020	94.17392	121.9446	64.54319	
Zimbabwe	DALYs	Female	2021	113.5206	143.97	80.33348	
Zimbabwe	DALYs	Female	2022	96.74571	128.0288	66.84837	
Zimbabwe	DALYs	Female	2023	98.09954	133.0422	66.44316	
Vanuatu	Deaths	Male	2012	5.359956	8.309177	3.309147	
Vanuatu	Deaths	Female	2012	3.318198	5.555457	2.022192	
Vanuatu	Deaths	Male	2015	5.639466	8.809323	3.405257	
Vanuatu	Deaths	Female	2015	3.667142	6.016409	2.272016	
Vanuatu	Deaths	Male	2014	5.56524	8.682505	3.392025	
Vanuatu	Deaths	Female	2014	3.577647	5.884069	2.20125	
Vanuatu	Deaths	Male	2011	5.303559	8.034538	3.27396	
Vanuatu	Deaths	Female	2011	3.21788	5.350621	1.913716	
Vanuatu	Deaths	Male	2010	5.166165	8.034587	3.133915	
Vanuatu	Deaths	Female	2010	3.011347	4.989312	1.77527	
Vanuatu	Deaths	Male	2013	5.462047	8.355739	3.363131	
Vanuatu	Deaths	Female	2013	3.451879	5.849603	2.131966	
Vanuatu	Deaths	Male	2018	5.782282	9.409111	3.472456	
Vanuatu	Deaths	Female	2018	3.98724	6.49662	2.451515	
Vanuatu	Deaths	Male	2021	5.915647	9.436208	3.51865	
Vanuatu	Deaths	Female	2021	4.148504	6.343854	2.512157	
Vanuatu	Deaths	Male	2017	5.761074	9.263095	3.43088	
Vanuatu	Deaths	Female	2017	3.888061	6.128083	2.404795	
Vanuatu	Deaths	Male	2020	5.832212	9.649131	3.467966	
Vanuatu	Deaths	Female	2020	4.171268	6.434979	2.603783	
Vanuatu	Deaths	Male	2016	5.729669	9.097538	3.447993	
Vanuatu	Deaths	Female	2016	3.78146	6.122577	2.351584	
Vanuatu	Deaths	Male	2019	5.772719	9.483766	3.468802	
Vanuatu	Deaths	Female	2019	4.067642	6.374308	2.530954	
Vanuatu	Deaths	Male	2023	6.277124	10.24416	3.717049	

Vanuatu	Deaths	Female	2023	4.828338	7.33662	2.908075	
Vanuatu	Deaths	Male	2022	6.115643	10.15591	3.648916	
Vanuatu	Deaths	Female	2022	4.565702	6.824875	2.817919	
Vanuatu	DALYs	Male	2011	154.6861	240.3133	95.74112	
Vanuatu	DALYs	Female	2011	87.48367	145.5882	51.74725	
Vanuatu	DALYs	Male	2010	150.9872	234.8107	92.29162	
Vanuatu	DALYs	Female	2010	81.85444	136.6613	48.13567	
Vanuatu	DALYs	Male	2012	156.1329	246.3418	96.5901	
Vanuatu	DALYs	Female	2012	90.18398	150.6619	55.14203	
Vanuatu	DALYs	Male	2013	158.9049	249.1595	98.8724	
Vanuatu	DALYs	Female	2013	93.9165	159.367	58.44214	
Vanuatu	DALYs	Male	2014	161.7869	252.2871	98.84575	
Vanuatu	DALYs	Female	2014	97.45412	160.485	60.15469	
Vanuatu	DALYs	Male	2015	164.075	256.1181	98.86738	
Vanuatu	DALYs	Female	2015	99.96839	164.9197	62.26534	
Vanuatu	DALYs	Male	2016	166.9545	268.2683	101.742	
Vanuatu	DALYs	Female	2016	103.1966	167.2406	64.16433	
Vanuatu	DALYs	Male	2018	169.4904	277.5525	100.8779	
Vanuatu	DALYs	Female	2018	109.3674	178.1195	67.92228	
Vanuatu	DALYs	Male	2017	168.3558	275.8615	100.6348	
Vanuatu	DALYs	Female	2017	106.3204	169.2497	66.13966	
Vanuatu	DALYs	Male	2020	172.2642	286.6177	102.9224	
Vanuatu	DALYs	Female	2020	115.0164	174.7038	71.40841	
Vanuatu	DALYs	Male	2022	181.0341	301.2703	108.4286	
Vanuatu	DALYs	Female	2022	126.1655	190.4025	78.00886	
Vanuatu	DALYs	Male	2019	169.8324	284.0501	101.1753	
Vanuatu	DALYs	Female	2019	111.879	177.1181	69.79154	
Vanuatu	DALYs	Male	2021	175.6034	285.7228	105.8942	
Vanuatu	DALYs	Female	2021	114.44	177.6435	68.87881	
Vanuatu	DALYs	Male	2023	186.1438	306.9409	111.3054	
Vanuatu	DALYs	Female	2023	133.4756	204.5572	80.63093	
Vanuatu	Incidence	Male	2010	5.22981	8.137665	3.176369	
Vanuatu	Incidence	Female	2010	3.003856	4.984695	1.762382	
Vanuatu	Incidence	Male	2011	5.367515	8.185863	3.316417	
Vanuatu	Incidence	Female	2011	3.211829	5.344709	1.909289	
Vanuatu	Incidence	Male	2012	5.424844	8.457889	3.360787	

Vanuatu	Incidence	Female	2012	3.312614	5.543319	2.026083	
Vanuatu	Incidence	Male	2013	5.528544	8.494927	3.414232	
Vanuatu	Incidence	Female	2013	3.448458	5.85434	2.141498	
Vanuatu	Incidence	Male	2014	5.634412	8.800983	3.430265	
Vanuatu	Incidence	Female	2014	3.577006	5.893879	2.200976	
Vanuatu	Incidence	Male	2015	5.712115	8.931361	3.452578	
Vanuatu	Incidence	Female	2015	3.668796	6.034505	2.272283	
Vanuatu	Incidence	Male	2016	5.8074	9.267142	3.508452	
Vanuatu	Incidence	Female	2016	3.786766	6.122955	2.344118	
Vanuatu	Incidence	Male	2017	5.846396	9.453975	3.492776	
Vanuatu	Incidence	Female	2017	3.898595	6.156158	2.411956	
Vanuatu	Incidence	Male	2018	5.874598	9.580802	3.514707	
Vanuatu	Incidence	Female	2018	4.003141	6.537041	2.46414	
Vanuatu	Incidence	Male	2019	5.87159	9.680643	3.515147	
Vanuatu	Incidence	Female	2019	4.088069	6.414369	2.547837	
Vanuatu	Incidence	Male	2020	5.938444	9.830864	3.541472	
Vanuatu	Incidence	Female	2020	4.196619	6.434878	2.611357	
Vanuatu	Incidence	Male	2021	6.030317	9.681811	3.603792	
Vanuatu	Incidence	Female	2021	4.174622	6.405381	2.524432	
Vanuatu	Incidence	Male	2022	6.235198	10.42146	3.710553	
Vanuatu	Incidence	Female	2022	4.603671	6.89535	2.84711	
Vanuatu	Incidence	Male	2023	6.407778	10.56477	3.78708	
Vanuatu	Incidence	Female	2023	4.872859	7.429662	2.951934	
New Caledonia	Incidence	Male	2010	114.8028	124.2694	106.2159	
New Caledonia	Incidence	Male	2011	116.1901	125.8573	107.7687	
New Caledonia	Incidence	Male	2012	118.2632	128.5349	109.4026	
New Caledonia	Incidence	Male	2013	119.7474	131.2801	111.1458	
New Caledonia	Incidence	Male	2014	118.7956	130.5493	109.4385	
New Caledonia	Incidence	Male	2015	120.2042	131.3871	110.3178	
New Caledonia	Incidence	Male	2016	118.8414	130.4744	108.6359	
New Caledonia	Incidence	Male	2017	116.8287	128.0953	106.0259	
New Caledonia	Incidence	Male	2018	115.5701	125.9906	104.6524	
New Caledonia	Incidence	Male	2019	112.3255	122.7171	101.2326	
New Caledonia	Incidence	Male	2020	110.3055	122.0485	98.3241	
New Caledonia	Incidence	Male	2021	106.4234	118.7494	94.979	
New Caledonia	Incidence	Male	2022	114.8203	127.7755	102.8593	

New Caledonia	Incidence	Male	2023	117.0741	131.7335	104.8773	
New Caledonia	Incidence	Female	2010	35.41742	39.71053	31.61853	
New Caledonia	Incidence	Female	2011	36.87781	41.17721	32.76137	
New Caledonia	Incidence	Female	2012	39.61437	44.21602	35.17579	
New Caledonia	Incidence	Female	2013	40.59801	45.36359	36.08416	
New Caledonia	Incidence	Female	2014	42.67623	47.6677	37.80383	
New Caledonia	Incidence	Female	2015	44.38225	49.91233	38.96715	
New Caledonia	Incidence	Female	2016	45.29262	50.82182	39.85537	
New Caledonia	Incidence	Female	2017	46.25055	52.04931	40.61959	
New Caledonia	Incidence	Female	2018	46.54728	52.31984	40.58825	
New Caledonia	Incidence	Female	2019	47.75479	54.26334	41.6973	
New Caledonia	Incidence	Female	2020	47.33913	53.32633	41.57944	
New Caledonia	Incidence	Female	2021	45.3921	50.93658	39.44771	
New Caledonia	Incidence	Female	2022	49.68141	56.18925	42.98953	
New Caledonia	Incidence	Female	2023	50.41759	57.1937	43.63068	
New Caledonia	Deaths	Male	2010	90.9006	96.72544	85.32934	
New Caledonia	Deaths	Male	2011	90.86131	96.77315	85.26793	
New Caledonia	Deaths	Male	2012	91.56241	97.34974	85.88436	
New Caledonia	Deaths	Male	2013	91.66449	97.37701	86.33171	
New Caledonia	Deaths	Male	2014	89.98892	96.27159	84.88355	
New Caledonia	Deaths	Male	2015	90.49408	96.4065	84.82593	
New Caledonia	Deaths	Male	2016	88.94667	95.6934	83.00777	
New Caledonia	Deaths	Male	2017	87.35589	94.35656	81.44536	
New Caledonia	Deaths	Male	2018	86.3447	93.63545	80.49294	
New Caledonia	Deaths	Male	2019	84.04762	90.41122	78.11394	
New Caledonia	Deaths	Male	2020	82.45235	89.51179	76.31456	
New Caledonia	Deaths	Male	2021	79.63843	86.78299	73.57627	
New Caledonia	Deaths	Male	2022	86.38327	95.31298	79.37635	
New Caledonia	Deaths	Male	2023	88.22408	97.39908	80.67088	
New Caledonia	Deaths	Female	2010	28.61715	30.87592	25.39042	
New Caledonia	Deaths	Female	2011	29.44192	31.98081	26.07866	
New Caledonia	Deaths	Female	2012	31.33166	34.07799	27.81798	
New Caledonia	Deaths	Female	2013	31.69041	34.49339	28.06214	
New Caledonia	Deaths	Female	2014	32.85995	35.92923	29.22619	
New Caledonia	Deaths	Female	2015	33.97836	36.88319	30.4032	
New Caledonia	Deaths	Female	2016	34.33398	37.30427	30.63679	

New Caledonia	Deaths	Female	2017	34.83347	37.90691	31.08474	
New Caledonia	Deaths	Female	2018	34.886	37.82403	30.98332	
New Caledonia	Deaths	Female	2019	35.70934	38.97127	31.40227	
New Caledonia	Deaths	Female	2020	35.33271	38.7048	31.45512	
New Caledonia	Deaths	Female	2021	33.84664	37.11269	29.99376	
New Caledonia	Deaths	Female	2022	37.14528	40.70649	32.94486	
New Caledonia	Deaths	Female	2023	37.50235	40.89587	33.03537	
New Caledonia	DALYs	Male	2010	2203.871	2344.486	2071.609	
New Caledonia	DALYs	Male	2011	2193.02	2327.144	2059.287	
New Caledonia	DALYs	Male	2012	2182.493	2324.49	2047.505	
New Caledonia	DALYs	Male	2013	2174.282	2308.545	2054.85	
New Caledonia	DALYs	Male	2014	2127.96	2268.129	2014.532	
New Caledonia	DALYs	Male	2015	2120.466	2267.052	2001.013	
New Caledonia	DALYs	Male	2016	2072.733	2226.59	1938.565	
New Caledonia	DALYs	Male	2017	2014.951	2166.959	1887.538	
New Caledonia	DALYs	Male	2018	1975.899	2132.857	1848.514	
New Caledonia	DALYs	Male	2019	1902.209	2040.662	1776.066	
New Caledonia	DALYs	Male	2020	1853.891	2012.349	1729.301	
New Caledonia	DALYs	Male	2021	1782.835	1946.553	1663.196	
New Caledonia	DALYs	Male	2022	1925.938	2127.61	1781.295	
New Caledonia	DALYs	Male	2023	1968.42	2186.471	1804.907	
New Caledonia	DALYs	Female	2010	669.6898	716.1359	612.7886	
New Caledonia	DALYs	Female	2011	688.6619	742.444	628.7657	
New Caledonia	DALYs	Female	2012	725.6752	782.7077	662.3743	
New Caledonia	DALYs	Female	2013	731.8545	786.3595	670.1417	
New Caledonia	DALYs	Female	2014	757.8231	814.7237	697.4015	
New Caledonia	DALYs	Female	2015	772.4401	827.9956	710.7817	
New Caledonia	DALYs	Female	2016	778.2926	838.1776	714.9702	
New Caledonia	DALYs	Female	2017	785.8146	845.6065	720.3826	
New Caledonia	DALYs	Female	2018	782.0348	841.5554	716.523	
New Caledonia	DALYs	Female	2019	793.6609	861.3723	725.7846	
New Caledonia	DALYs	Female	2020	777.0419	847.9521	710.8173	
New Caledonia	DALYs	Female	2021	740.4573	812.3984	673.8524	
New Caledonia	DALYs	Female	2022	809.2025	891.2519	731.1357	
New Caledonia	DALYs	Female	2023	823.183	903.1381	739.2532	
**Supplementary F**							

Country	._Total_Inciden	cidence_Low 9!	cidence_High 9	S1_Total_Morta	S1_Mortality_Lo	S1_Mortality_High 95%UI	
Afghanistan	5645.104833	4690.351302	9039.491787	5296.926457	4395.09886	8272.242836	
Albania	577.9599745	1340.544979	1842.830555	552.26277	1347.049365	1713.518286	
Algeria	6831.12783	16931.444	19618.37756	6259.637356	14988.23963	17606.08789	
Angola	1814.724206	3670.563257	3670.563257	1666.782	3445.975472	3445.975472	
Argentina	10153.28235	6554.808181	7513.936786	8223.916281	5964.852572	6662.834448	
Armenia	791.1109955	445.2393668	635.2212097	650.7569265	402.580266	523.4671009	
Australia	7893.354847	5116.371754	5639.611952	5114.113218	4205.756283	4645.50741	
Austria	2400.698436	2152.697976	2469.70807	1728.299112	1916.387479	2205.810448	
Azerbaijan	2372.365506	2648.804563	3439.095192	1983.382021	2266.274466	2856.06371	
Bahamas	38.115841	12.37118663	208.7735397	30.349192	14.8158168	138.1699952	
Bahrain	238.7707115	446.3374845	2019.137477	205.4638685	402.8063155	1453.123232	
Bangladesh	18575.76545	78321.17262	84239.58719	17195.72435	72584.88526	77881.88023	
Barbados	25.050462	21.62652534	105.525112	19.8621655	20.58253503	75.07042029	
Belarus	2195.07151	2611.03548	3057.398271	1802.79543	2000.023227	2207.084103	
Belgium	3952.939106	2632.671976	2935.385783	2601.401176	2106.325201	2427.423473	
Belize	45.549144	21.42653603	287.9123087	41.920218	23.67992555	231.6677162	
Benin	391.3434545	292.6958545	1147.164532	366.909645	266.7777496	1148.322196	
Bhutan	61.9611665	178.66307	195.4047827	61.9611665	177.0801504	193.8218632	
Bolivia (Plurina	1414.523396	2486.663053	3717.34486	1205.54427	2242.949355	3289.745639	
Bosnia Herzego	909.240015	1028.148861	1202.409647	715.746873	879.3568893	1014.364622	
Botswana	139.833126	299.694628	1270.166407	131.354291	254.4825201	1257.856906	
Brazil	32465.64415	53598.51003	56937.53356	27700.00087	50526.5134	53132.37805	
Brunei Darussa	125.560961	177.1471556	497.9553892	110.253767	168.9356701	467.5428497	
Bulgaria	1521.963005	1396.71007	1643.082993	1287.204074	1281.07525	1516.251654	
Burkina Faso	1400.126648	2543.568266	2543.568266	1381.393637	2549.969698	2549.969698	
Burundi	843.080987	920.8930107	3579.91497	794.817547	875.4808184	3422.897138	
Cambodia	3685.650885	9000.136609	9373.311719	3292.961196	8479.603035	8811.966578	
Cameroon	1605.086411	455.9960189	19728.32709	1554.3459	467.6147593	17851.40917	
Canada	14890.38696	12168.62392	13225.11822	9916.684926	10252.37772	10869.61381	
Cape Verde	12.15408	8.446249839	129.2460698	9.639536	4.071233762	176.8146281	
Central African	222.0902535	128.2371959	1805.131236	216.853673	143.4881572	1701.012656	
Chad	805.0094406	797.3490188	1278.352913	781.6764978	749.3253728	1259.707929	
Chile	2658.941688	3001.843136	3696.315431	2364.645528	2856.776397	3392.113735	
China	520093.7116	908437.1441	935571.016	343235.2011	670123.7199	692037.1899	
Colombia	6123.890649	5594.118712	6480.163461	5177.814718	5025.148926	5646.059272	

Comoros	24.193138	23.78914496	76.18440754	24.193138	18.77339075	81.21468843	
Congo, Democr	4789.680668	3364.759946	63429.42818	4463.289303	3375.325627	59215.78134	
Congo, Republi	159.357786	146.5205569	708.5176797	145.6269333	130.112453	677.2046965	
Costa Rica	367.5688825	486.891025	765.5894591	266.640381	366.7026556	594.7075942	
Côte d'Ivoire	1366.477354	3139.536078	5651.182486	1227.07769	2822.067571	5149.767059	
Croatia	1249.350396	1373.496711	1668.838273	1001.971399	1240.489664	1465.375425	
Cuba	2838.04762	2812.426897	3358.43588	2314.190994	2533.772787	2905.13348	
Cyprus	424.068379	412.8496788	584.5386127	350.774322	334.7020183	532.1644394	
Czechia	2402.646341	1574.105558	1764.825064	2004.714026	1487.17648	1694.080727	
Denmark	2103.877985	1585.413809	1902.730299	1509.853806	1437.162815	1723.101558	
Djibouti	46.557185	86.3675283	190.5874446	39.6358655	83.61577343	180.1737516	
Dominican Rep	1679.726703	3788.081433	5269.71208	1420.260365	3552.881121	4661.361166	
Ecuador	1343.944386	808.2215016	1136.082633	1151.607672	771.6382089	1036.027049	
Egypt	14832.63476	29039.29033	34557.11002	13374.30221	27330.39792	32378.50213	
El Salvador	315.13928	605.4522246	1126.817091	284.6976815	601.0281004	1038.536409	
Equatorial Guir	85.11281	84.93987272	413.1312624	83.4829075	85.38627824	397.5647327	
Eritrea	184.9531165	369.9204048	708.6913501	162.2439345	341.0092947	632.4781143	
Estonia	333.821277	214.5517879	294.113853	262.5144975	178.6858461	243.5944284	
Eswatini	74.733186	39.9121791	311.9698163	74.733186	34.40636165	372.9910279	
Ethiopia	8326.198594	24088.69252	30699.93758	8101.176719	23388.41281	30246.354	
Fiji	69.801844	77.22446666	303.6735197	67.2960385	81.19498049	284.7782823	
Finland	1052.685899	1239.730772	1453.416476	776.986375	1027.70295	1298.979636	
France (metrop	24880.39301	25288.43654	27164.73921	16804.81255	19540.75293	20872.68925	
France, Guadel	40.3952385	46.5652902	47.37277798	34.471752	40.69002392	41.38961161	
France, La Réur	207.3929345	230.5052517	240.1380125	175.789641	192.1749016	200.8880599	
France, Martini	25.5888335	30.12218902	30.73401873	22.5850265	27.3799639	27.93105099	
French Guyana	72.8576575	83.49651011	83.49651011	59.8153065	68.67722015	68.67722015	
French Polynes	113.507569	126.6957797	135.44817	99.2813265	111.8686739	118.6093169	
Gabon	126.584795	130.7366478	988.7189862	116.3868605	122.8851252	942.2593126	
Gaza Strip and	1792.696018	2938.383821	4304.900231	1665.862474	2634.812524	3806.095678	
Georgia	883.753196	888.8673136	1231.22543	729.81298	783.9784783	995.0011271	
Germany	22358.55206	24445.07356	26254.53883	17000.22287	21997.53595	23434.43401	
Ghana	1286.896225	3243.865795	5461.410543	1261.419113	3187.508461	5487.421694	
Greece	2861.844419	3491.469205	4079.298588	2260.609111	3188.082102	3654.670521	
Guam	61.9020135	126.704587	129.8270463	59.6045115	124.8875402	128.000346	
Guatemala	948.468253	706.2832014	1132.13775	886.895706	697.355679	1046.473901	

Guinea	830.3412895	626.5290624	2382.587287	818.6547985	594.0322216	2380.622161	
Guinea-Bissau	77.1699855	21.47568494	1482.298954	73.7080035	20.79946077	1462.688453	
Guyana	40.80416	38.93030119	369.6172708	36.7025045	42.9567573	300.5811814	
Haiti	891.914188	1642.12267	2639.713159	809.7908615	1599.855663	2573.860226	
Honduras	1017.631223	1548.408968	2481.315483	735.060204	1249.995734	1841.190778	
Hungary	4318.847757	3333.219251	3682.866088	3623.101251	3146.470008	3437.106409	
Iceland	116.537755	58.2751195	116.7510985	66.753326	47.44964093	86.15176001	
India	99166.37351	315755.3911	333891.5199	90699.71764	297430.1101	315354.8966	
Indonesia	44418.98872	76501.48558	171860.0224	39608.26815	72612.55606	149701.0793	
Iran, Islamic Re	12312.60984	18965.11582	21099.93131	11186.37141	16179.80749	18079.88183	
Iraq	9809.809312	24488.22328	29583.30952	9086.05703	21746.61742	26827.33439	
Ireland	1879.99959	2042.529557	2534.953406	1163.13339	1539.883308	1970.056319	
Israel	3016.969087	2315.213617	2855.578239	1823.977508	1625.620259	2044.270717	
Italy	13105.58915	11244.66358	12041.93387	9691.444263	10074.07011	10715.43847	
Jamaica	346.340776	393.6665816	969.8559621	292.661782	371.2550569	789.1722346	
Japan	32575.96435	45642.12367	49995.90887	15396.1481	28194.68286	29521.70103	
Jordan	2415.48454	3704.472247	5092.758346	2250.178314	3266.144093	4646.950158	
Kazakhstan	6015.528378	2609.618748	2969.698312	5007.856695	2110.644599	2465.798148	
Kenya	2839.771476	5311.609252	8776.287284	2631.388041	5085.776279	8502.747222	
Korea, Democr	10283.00632	22745.52893	23534.61007	8635.617529	20579.61451	21253.83741	
Korea, Republic	12288.7182	20663.24119	21584.78977	8105.620853	14570.94003	15548.89018	
Kuwait	401.7400675	376.5296373	766.5058528	362.376197	317.4498013	690.354525	
Kyrgyzstan	1529.792791	1000.93772	1614.896162	1220.01595	884.1742622	1232.021849	
Lao People's De	1765.321451	5605.229957	5831.624507	1570.242161	5113.154686	5313.084737	
Latvia	402.8852	347.924189	453.0766168	336.289362	289.5150335	394.0062528	
Lebanon	1394.121847	2244.521458	3035.436345	1247.640032	1962.126485	2713.244418	
Lesotho	129.8259585	90.43059024	1966.526345	117.8536525	93.21949747	1870.878467	
Liberia	245.0758875	313.1932969	1090.719896	236.1624525	298.0166511	987.4238843	
Libya	1648.646786	2565.11184	3660.218911	1532.199866	2377.811685	3350.59302	
Lithuania	609.304771	433.0336331	558.0424718	498.045367	387.8154781	482.6991129	
Luxembourg	208.520255	96.97610893	167.3938683	166.1114665	89.29139097	144.6902502	
Madagascar	1143.724664	1145.602325	5095.923645	1063.928224	1073.914857	4865.646176	
Malawi	592.794995	598.2374653	3509.453094	568.801097	574.2037542	3385.001504	
Malaysia	6810.640689	7623.646765	18864.26217	5914.833795	7179.907669	16949.26751	
Maldives	76.2423425	77.00330706	114.313472	72.50644	74.13639175	104.4579493	
Mali	1501.494368	1976.394205	3859.466642	1501.494368	1936.623815	3964.660265	

Malta	108.4584595	52.45142757	90.72521029	84.8375005	46.51422481	83.83317103	
Mauritania	187.618984	41.3794802	3404.504896	178.203386	48.13028079	3193.723691	
Mauritius	95.9986155	101.1439501	207.5549611	88.2227015	97.42619707	203.9773014	
Mexico	8190.023518	6550.916552	7608.311109	7747.555821	6362.259985	7217.864328	
Mongolia	945.2669045	1278.644427	1872.265777	826.737891	1213.437047	1556.470875	
Montenegro	200.166292	201.1205652	292.1267888	165.803142	174.3245476	246.1218647	
Morocco	9280.716075	38321.67013	42982.12893	8387.657906	34156.68566	38636.50771	
Mozambique	967.2218124	733.1796949	1549.477419	932.8634726	697.1838742	1567.347765	
Myanmar	9868.144879	21547.04915	26426.37694	9079.649006	20242.95391	24873.4433	
Namibia	276.9654625	255.3589329	858.9869756	263.694958	227.6952629	848.7050477	
Nepal	3276.179967	9626.297662	10500.61568	2985.4693	9061.675264	9934.662444	
New Caledonia	144.2604865	156.4289492	164.6831027	106.7654835	123.9540601	130.0195677	
New Zealand	1593.995586	1848.337427	2246.176308	1131.865233	1373.772507	1709.343682	
Nicaragua	493.0976135	491.1879083	884.3134294	458.185044	476.9735351	777.9446282	
Niger	501.6107196	306.2788024	2290.510878	501.6107196	298.8972315	2360.918229	
Nigeria	6294.338933	10095.79126	14700.42639	5935.153377	9778.43354	14393.28306	
North Macedor	477.5536805	610.1512606	1032.466228	413.1117215	565.1353068	895.4732172	
Norway	1661.113296	980.7510208	1198.319698	1047.870824	758.3085833	912.35652	
Oman	415.900688	250.2515269	619.7897584	389.336102	204.8326287	579.9401409	
Pakistan	21949.44039	34114.41564	38321.08854	19345.07765	30360.77096	34168.45858	
Panama	365.849352	192.7609856	392.522997	298.307528	178.0446568	315.1286505	
Papua New Gui	1607.264238	1502.583269	6273.031025	1457.861088	1481.442481	5705.292103	
Paraguay	1116.454605	1488.819125	2085.995333	939.722182	1336.517365	1747.803059	
Peru	2759.432477	4679.429854	5844.891693	2454.917862	4417.559081	5374.685083	
Philippines	33927.30537	31741.01117	34828.79885	30179.20291	29570.55544	32480.75678	
Poland	12438.00066	12012.24145	12841.93966	10451.88001	11534.55985	12267.21005	
Portugal	2399.276541	2912.785867	3437.51872	1871.51836	2682.176086	3110.96485	
Puerto Rico	264.94961	263.46441	402.874213	194.3289675	200.4132271	362.179752	
Qatar	232.9459755	204.5859296	788.4197314	216.3936585	161.0266363	698.8753964	
Republic of Mo	557.906648	576.4443894	739.0709835	479.313238	510.2666952	624.2906577	
Romania	5045.902004	5799.985518	6501.742549	4442.90239	5477.689308	6056.045826	
Russian Federa	39559.86975	31467.34032	32788.19445	28507.83659	23161.21703	24279.17958	
Rwanda	664.9450075	1757.169566	3794.864543	619.8697625	1597.542331	3627.81979	
Saint Lucia	16.7328415	7.575020245	86.7851615	13.635504	7.958989367	67.0207649	
Samoa	93.903598	242.8715899	242.8715899	70.602587	190.8400489	190.8400489	
Sao Tome and I	46.3763875	5.569814339	1456.008909	46.3763875	5.203510703	1402.162275	

Saudi Arabia	2047.445398	1399.312591	1979.706928	1821.612283	1283.104336	1840.40842	
Senegal	864.884382	119.9389204	18786.01986	804.192756	123.7369173	16645.20976	
Serbia	2264.109139	2052.023965	2340.068326	1875.852389	1850.580968	2086.626133	
Sierra Leone	175.4623816	80.06282954	1123.925872	169.0119071	70.75418731	1087.234162	
Singapore	1506.89003	1446.30379	1848.324998	1386.375777	1171.931154	1712.044425	
Slovakia	1193.551846	1125.277449	1496.530759	1022.132356	1048.048553	1343.709155	
Slovenia	693.426802	618.4457875	767.6714983	516.159952	549.9544478	684.5764008	
Solomon Island	83.217744	180.7269247	433.3982108	71.219808	165.165331	339.544116	
Somalia	1301.679969	1739.608775	3001.739697	1245.938693	1732.858	2958.362397	
South Africa	15120.4133	21924.45017	22099.58004	14032.41752	20722.86561	20913.52144	
South Sudan	384.094353	201.4151819	5562.153282	356.7625745	203.271959	5291.438251	
Spain	12729.89378	15553.37376	16947.86205	9343.83718	13175.2769	14251.11394	
Sri Lanka	1747.623593	5577.904321	7027.74137	1613.443195	5182.661491	6642.061166	
Sudan	2123.332676	3772.007297	6205.715848	1996.092009	3621.488064	5854.433321	
Suriname	126.0626165	88.91209608	347.5091869	104.115893	90.0511895	281.6985936	
Sweden	1970.846195	1314.207081	1494.484903	1503.530111	1298.289636	1467.969763	
Switzerland	2264.012788	1617.808628	1917.995468	1498.177884	1340.876706	1553.062138	
Syrian Arab Rep	6979.704208	21821.57444	26402.68307	6714.750969	21281.58977	25713.73326	
Tajikistan	847.458243	1344.93849	2473.961559	769.7498515	1330.116159	2252.757074	
Tanzania, Unite	3303.62665	2483.896721	5763.494371	2914.763343	2304.798578	5466.698394	
Thailand	12298.95814	22094.88165	23600.03895	10170.83551	18865.5965	20160.89364	
The Netherland	6673.03987	5434.28747	5930.840112	4530.388865	4917.088693	5363.438472	
The Republic of	222.1652331	257.5327671	1222.986608	222.1652331	228.9535377	1295.665717	
Timor-Leste	196.2660135	298.9462962	433.128249	175.38376	288.5308022	392.6128196	
Togo	311.5202821	374.4553459	2696.976796	278.6800972	399.1271674	2137.344751	
Trinidad and To	167.732703	209.1897407	468.0191525	136.719096	193.7478565	365.3465708	
Tunisia	2743.598457	5702.219474	6983.670734	2457.922363	4791.970842	5890.484123	
Türkiye	36986.54941	72068.27272	76096.32266	34653.19568	68165.13822	74248.66292	
Turkmenistan	985.8843135	752.1540436	3781.743233	908.887439	751.0551443	3490.67897	
Uganda	2604.180008	3927.642437	7129.562911	2305.334318	3701.584387	6753.364619	
Ukraine	7305.928317	6874.953029	7282.916606	6074.623474	5701.415887	6040.738443	
United Arab Em	941.9881985	2538.204859	4759.705264	815.7986655	2205.788275	4455.078054	
United Kingdor	22796.64793	18106.69444	18960.95938	14791.45593	14248.36526	15135.48614	
United States o	122138.7741	58102.81155	59884.54176	64273.48005	42307.45214	43732.8903	
Uruguay	1013.877226	1197.149078	1575.526326	875.9767255	1104.976733	1469.77361	
Uzbekistan	4635.285075	7440.154897	9745.269004	3928.129368	6822.175422	8346.776214	

Vanuatu	40.785856	45.31977423	123.4457244	40.785856	40.78012824	121.8365197	
Venezuela	5043.375569	11637.75138	14169.366	4239.192331	10832.16966	12650.5369	
Viet Nam	23098.02248	72449.66191	77532.45526	21354.20408	65913.34932	70952.28318	
Yemen	2917.333404	3364.180397	4820.758491	2846.357001	3333.101197	4810.736646	
Zambia	1289.389405	1510.994807	3270.727705	1194.407846	1419.532853	3140.606781	
Zimbabwe	1674.806553	1919.829631	3458.695434	1559.508292	1823.401063	3406.629336	
**Supplementary G**							
COUNTRY	S2_Total_Incid	S2_Incidence_L	ce_High 95% UI	Total_Mortality	ty_Low 95% UI	S2_Mortality_High 95% UI	
Afghanistan	6716.01883	4816.031589	9379.584522	6028.168528	4395.09886	8272.242836	
Albania	1595.608795	1369.18201	1858.947527	1517.999577	1347.049365	1713.518286	
Algeria	18630.35311	17312.49995	20078.06873	16232.15074	14988.23963	17606.08789	
Angola	3805.734027	3805.734027	3805.734027	3445.975472	3445.975472	3445.975472	
Argentina	7732.344249	7117.82509	8400.731187	6303.968067	5964.852572	6662.834448	
Armenia	546.7113003	447.1062346	674.0916975	458.4665149	402.580266	523.4671009	
Australia	6846.096945	6330.72262	7410.742003	4419.827892	4205.756283	4645.50741	
Austria	3018.539147	2819.539251	3231.456414	2056.041037	1916.387479	2205.810448	
Azerbaijan	3099.769586	2698.768373	3572.004277	2541.716848	2266.274466	2856.06371	
Bahamas	56.56879006	11.10019803	343.5205719	44.77273884	14.8158168	138.1699952	
Bahrain	959.1864817	443.5054251	2155.799654	755.7639447	402.8063155	1453.123232	
Bangladesh	84960.45939	81793.5562	88265.55433	75184.9423	72584.88526	77881.88023	
Barbados	52.29099277	21.19898207	132.4355911	39.34016823	20.58253503	75.07042029	
Belarus	2982.461002	2724.153224	3270.888072	2100.97506	2000.023227	2207.084103	
Belgium	3615.803558	3427.572051	3814.942826	2261.254899	2106.325201	2427.423473	
Belize	75.60599273	21.57627461	354.6247126	68.12400874	23.67992555	231.6677162	
Benin	612.1743288	317.7332855	1195.473106	556.8145341	266.7777496	1148.322196	
Bhutan	190.0867011	181.4716227	198.7017795	185.4510068	177.0801504	193.8218632	
Bolivia (Plurina	3318.745212	2680.131439	4120.967041	2714.818592	2242.949355	3289.745639	
Bosnia Herzegc	1189.748062	1091.230525	1298.696571	944.24693	879.3568893	1014.364622	
Botswana	612.1591976	315.8872793	1200.100588	559.8586091	254.4825201	1257.856906	
Brazil	60021.80278	57856.59046	62268.45107	51812.78797	50526.5134	53132.37805	
Brunei Darussa	332.9715592	206.2053308	534.1074941	281.7402118	168.9356701	467.5428497	
Bulgaria	1650.211138	1515.569655	1798.220066	1393.353908	1281.07525	1516.251654	
Burkina Faso	2598.86768	2598.86768	2598.86768	2549.969698	2549.969698	2549.969698	
Burundi	1934.385467	963.9453308	3977.328946	1718.332476	875.4808184	3422.897138	
Cambodia	9409.304926	9204.974473	9613.635379	8643.701686	8479.603035	8811.966578	

Cameroon	3042.402918	447.9327529	21767.76537	2813.291876	467.6147593	17851.40917	
Canada	15148.04226	14424.12683	15908.42026	10556.198	10252.37772	10869.61381	
Cape Verde	44.84101039	20.23299716	117.7540725	29.33046634	4.071233762	176.8146281	
Central African	484.0489599	128.8634002	1824.752303	468.7902094	143.4881572	1701.012656	
Chad	1015.405929	814.8561125	1281.959197	965.8180155	749.3253728	1259.707929	
Chile	3595.652601	3181.789573	4062.985756	3112.81419	2856.776397	3392.113735	
China	1162201.199	1142381.054	1182388.031	680992.1949	670123.7199	692037.1899	
Colombia	6708.191238	6152.190728	7315.199763	5326.316167	5025.148926	5646.059272	
Comoros	40.79483702	23.63601314	75.11248479	41.26045821	18.77339075	81.21468843	
Congo, Democr	14684.5406	3308.219549	75221.0864	13310.82641	3375.325627	59215.78134	
Congo, Republi	325.0974522	154.6061527	712.6791325	291.823701	130.112453	677.2046965	
Costa Rica	679.3381544	544.0194325	850.5500944	465.8666549	366.7026556	594.7075942	
Côte d'Ivoire	4526.120498	3344.710126	6166.607771	3801.662517	2822.067571	5149.767059	
Croatia	1730.520016	1556.49918	1925.539416	1347.656076	1240.489664	1465.375425	
Cuba	3432.5564	3084.695192	3820.075642	2713.050127	2533.772787	2905.13348	
Cyprus	541.606063	432.2422132	690.5371602	421.9356972	334.7020183	532.1644394	
Czechia	1869.276439	1760.117406	1984.994049	1587.291438	1487.17648	1694.080727	
Denmark	1986.147456	1828.989515	2156.924426	1573.332122	1437.162815	1723.101558	
Djibouti	149.2977042	96.45287913	226.10985	123.5331852	83.61577343	180.1737516	
Dominican Rep	4785.938318	3951.921833	5797.593217	4070.115221	3552.881121	4661.361166	
Ecuador	1228.060231	987.2777105	1526.656854	894.0431335	771.6382089	1036.027049	
Egypt	33080.7072	30206.31191	36252.31235	29741.17231	27330.39792	32378.50213	
El Salvador	921.3521966	644.2736929	1317.938751	789.1612543	601.0281004	1038.536409	
Equatorial Guir	199.5332517	89.3241175	435.0524863	184.7774614	85.38627824	397.5647327	
Eritrea	530.6850253	379.8893218	742.5529509	459.7358568	341.0092947	632.4781143	
Estonia	305.9771694	260.7161766	359.7516499	208.2430351	178.6858461	243.5944284	
Eswatini	105.9223689	43.73201967	271.3373709	111.0619347	34.40636165	372.9910279	
Ethiopia	28407.32514	25385.99327	31784.00649	26596.11381	23388.41281	30246.354	
Fiji	154.4646973	74.70403473	326.7307958	151.7529606	81.19498049	284.7782823	
Finland	1654.531892	1557.825392	1757.609297	1155.265402	1027.70295	1298.979636	
France (metrop	35478.7109	34118.9813	36891.63087	20195.88147	19540.75293	20872.68925	
France, Guadel	56.29573044	55.10256043	57.48890045	41.03981776	40.69002392	41.38961161	
France, La Réur	266.3367218	261.3246592	271.3487843	196.5314808	192.1749016	200.8880599	
France, Martini	37.06278123	36.17865311	37.94690935	27.65550745	27.3799639	27.93105099	
French Guyana	95.90479776	95.90479776	95.90479776	68.67722015	68.67722015	68.67722015	
French Polynes	153.9598274	149.2985433	158.6211115	115.2389954	111.8686739	118.6093169	

Gabon	375.680807	142.0960691	983.3964735	335.8930099	122.8851252	942.2593126	
Gaza Strip and	3639.816479	2985.49731	4449.276564	3164.209778	2634.812524	3806.095678	
Georgia	1049.821956	887.9894924	1243.439657	882.505817	783.9784783	995.0011271	
Germany	29448.45682	28283.11753	30661.83794	22704.44121	21997.53595	23434.43401	
Ghana	4431.139672	3439.133266	5713.963665	4178.606416	3187.508461	5487.421694	
Greece	4118.61425	3783.982515	4483.815371	3413.111114	3188.082102	3654.670521	
Guam	131.4909031	129.9296734	133.0521327	126.4439431	124.8875402	128.000346	
Guatemala	943.904492	723.8531939	1233.40724	854.3528821	697.355679	1046.473901	
Guinea	1233.907611	655.6817918	2393.413795	1166.800788	594.0322216	2380.622161	
Guinea-Bissau	180.3957687	30.60285294	1699.800687	162.4102032	20.79946077	1462.688453	
Guyana	124.6585032	39.04311765	421.7932105	113.6836928	42.9567573	300.5811814	
Haiti	2368.695168	1846.142706	3039.691786	2032.044084	1599.855663	2573.860226	
Honduras	2018.426169	1547.113123	2637.695949	1516.628313	1249.995734	1841.190778	
Hungary	3796.969399	3585.474333	4021.189507	3288.560078	3146.470008	3437.106409	
Iceland	116.9675507	81.81979949	167.6726514	64.05151874	47.44964093	86.15176001	
India	338961.7045	329772.4299	348450.0185	306244.0105	297430.1101	315354.8966	
Indonesia	117789.6405	79584.29321	177371.634	103557.5604	72612.55606	149701.0793	
Iran, Islamic Re	21148.69779	20150.52954	22198.89234	17099.59725	16179.80749	18079.88183	
Iraq	27458.95149	25020.79979	30179.91285	24135.21912	21746.61742	26827.33439	
Ireland	3230.766793	2930.198566	3562.240459	1740.962715	1539.883308	1970.056319	
Israel	3153.867783	2770.400645	3598.698603	1822.565128	1625.620259	2044.270717	
Italy	13372.88888	12849.15943	13918.79022	10389.64762	10074.07011	10715.43847	
Jamaica	670.0838333	397.4725228	1145.060764	539.641134	371.2550569	789.1722346	
Japan	57205.84814	54510.05415	60035.60635	28850.56422	28194.68286	29521.70103	
Jordan	4445.924896	3803.015686	5215.137086	3888.454543	3266.144093	4646.950158	
Kazakhstan	2816.475522	2625.159797	3025.432992	2280.938613	2110.644599	2465.798148	
Kenya	7186.746684	5634.556337	9160.924948	6574.110762	5085.776279	8502.747222	
Korea, Democr	24964.63611	24491.07925	25450.82553	20913.63769	20579.61451	21253.83741	
Korea, Republi	26536.41532	25900.64015	27189.67486	15051.85661	14570.94003	15548.89018	
Kuwait	585.796073	408.6323948	842.8046312	464.1100457	317.4498013	690.354525	
Kyrgyzstan	1294.780925	999.8524651	1678.244957	1043.582961	884.1742622	1232.021849	
Lao People's De	6117.435331	5985.687199	6249.183464	5213.119712	5113.154686	5313.084737	
Latvia	433.5119045	377.8689182	498.890741	337.6349077	289.5150335	394.0062528	
Lebanon	2853.357877	2461.592436	3310.957074	2304.834507	1962.126485	2713.244418	
Lesotho	369.9707593	90.27767629	2589.33997	341.2506813	93.21949747	1870.878467	
Liberia	605.2475914	325.1494788	1144.522715	535.0908725	298.0166511	987.4238843	

Libya	3073.053095	2572.916406	3699.300041	2812.200906	2377.811685	3350.59302	
Lithuania	566.8833511	494.2841052	650.3442049	432.8071909	387.8154781	482.6991129	
Luxembourg	149.0760456	110.2038189	201.6933543	113.6690415	89.29139097	144.6902502	
Madagascar	2467.033511	1162.603202	5588.737442	2233.870336	1073.914857	4865.646176	
Malawi	1480.429358	622.549287	3618.320146	1361.689199	574.2037542	3385.001504	
Malaysia	12467.71051	8083.112183	19665.70593	10915.63375	7179.907669	16949.26751	
Maldives	93.49486251	77.2278989	114.5879731	87.61309977	74.13639175	104.4579493	
Mali	2786.013941	2053.966573	3792.66181	2762.149267	1936.623815	3964.660265	
Malta	80.77465651	62.59085401	104.3291091	62.40177376	46.51422481	83.83317103	
Mauritania	369.8697199	41.93572775	3684.608276	341.1976604	48.13028079	3193.723691	
Mauritius	153.1529404	111.2757475	211.5770377	140.9327084	97.42619707	203.9773014	
Mexico	7431.632739	6810.437543	8109.855183	6775.841065	6362.259985	7217.864328	
Mongolia	1577.58661	1283.93768	1941.84904	1374.070399	1213.437047	1556.470875	
Montenegro	248.4847433	203.4129921	304.001018	206.8751426	174.3245476	246.1218647	
Morocco	41273.86691	38966.88237	43751.36155	36311.61737	34156.68566	38636.50771	
Mozambique	1068.190793	742.560815	1535.21931	1044.137779	697.1838742	1567.347765	
Myanmar	25128.68861	22502.27375	28194.71367	22393.51251	20242.95391	24873.4433	
Namibia	478.2194952	271.9827528	842.353619	437.0730286	227.6952629	848.7050477	
Nepal	10419.43107	9992.960284	10869.62018	9486.188004	9061.675264	9934.662444	
New Caledonia	199.2052599	193.0314608	205.379059	126.9868139	123.9540601	130.0195677	
New Zealand	2474.627005	2248.029423	2723.377629	1532.059765	1373.772507	1709.343682	
Nicaragua	726.7761743	527.7786993	1004.897017	609.0976731	476.9735351	777.9446282	
Niger	752.9285937	306.2788024	2242.308673	761.2541379	298.8972315	2360.918229	
Nigeria	12944.13485	10783.33619	15538.2496	11866.27764	9778.43354	14393.28306	
North Macedor	831.9624005	622.4521724	1117.946148	710.0872696	565.1353068	895.4732172	
Norway	1444.838229	1306.071941	1598.29993	831.8452459	758.3085833	912.35652	
Oman	399.4355935	263.1314029	622.0759181	339.1205098	204.8326287	579.9401409	
Pakistan	37225.62038	35088.20395	39505.15701	32199.99746	30360.77096	34168.45858	
Panama	317.3333457	205.9876578	491.3538168	236.7234835	178.0446568	315.1286505	
Papua New Gui	3239.991811	1522.819422	6954.470704	2898.531044	1481.442481	5705.292103	
Paraguay	1834.836042	1515.911686	2229.345546	1527.440869	1336.517365	1747.803059	
Peru	6295.318148	5520.109378	7180.187565	4871.689463	4417.559081	5374.685083	
Philippines	34735.14193	33148.22983	36400.35025	30990.89232	29570.55544	32480.75678	
Poland	13096.43915	12607.71597	13604.79973	11894.97742	11534.55985	12267.21005	
Portugal	3743.13294	3394.475906	4132.555431	2887.841897	2682.176086	3110.96485	
Puerto Rico	377.0675212	314.2631715	453.3795822	269.3882766	200.4132271	362.179752	

Qatar	380.7684294	213.7602142	768.2714901	301.6042833	161.0266363	698.8753964	
Republic of Mo	675.9179662	591.3164069	773.8832532	564.2765664	510.2666952	624.2906577	
Romania	6541.764303	6128.7097	6985.794949	5759.261463	5477.689308	6056.045826	
Russian Federa	35136.61033	34508.77428	35775.88562	23713.32863	23161.21703	24279.17958	
Rwanda	2638.562792	1824.36036	3855.092317	2395.709434	1597.542331	3627.81979	
Saint Lucia	22.95678292	6.109904626	146.6874011	18.12666845	7.958989367	67.0207649	
Samoa	259.4051019	259.4051019	259.4051019	190.8400489	190.8400489	190.8400489	
Sao Tome and I	102.9013003	5.569814339	1511.075676	95.48371169	5.203510703	1402.162275	
Saudi Arabia	1831.736714	1533.548397	2192.497565	1535.950216	1283.104336	1840.40842	
Senegal	1560.97682	128.9445835	19813.38195	1419.962607	123.7369173	16645.20976	
Serbia	2362.734814	2189.861602	2550.842009	1964.846197	1850.580968	2086.626133	
Sierra Leone	299.9415515	80.06282954	1118.527139	279.4770535	70.75418731	1087.234162	
Singapore	1929.942689	1749.907522	2129.149077	1416.189086	1171.931154	1712.044425	
Slovakia	1382.040707	1175.474439	1627.48057	1186.178514	1048.048553	1343.709155	
Slovenia	824.1779044	728.8089237	932.4745368	613.4506126	549.9544478	684.5764008	
Solomon Island	281.1727913	182.8516177	437.6475968	236.7595358	165.165331	339.544116	
Somalia	2302.409691	1745.452488	3034.7487	2257.488898	1732.858	2958.362397	
South Africa	22520.4344	22439.92416	22600.94463	20818.19353	20722.86561	20913.52144	
South Sudan	972.9271801	201.5983084	6356.975575	895.9556547	203.271959	5291.438251	
Spain	20773.07432	19738.68002	21862.76152	13702.07296	13175.2769	14251.11394	
Sri Lanka	6476.733093	5791.087928	7251.29031	5862.324938	5182.661491	6642.061166	
Sudan	5082.122346	3923.113069	6587.067567	4607.298609	3621.488064	5854.433321	
Suriname	191.3399239	86.48052623	433.373932	159.8097194	90.0511895	281.6985936	
Sweden	1568.262908	1463.258297	1681.00021	1380.574544	1298.289636	1467.969763	
Switzerland	2196.078363	1997.885838	2414.467805	1443.293201	1340.876706	1553.062138	
Syrian Arab Rep	23815.36679	21690.37115	26161.93264	23388.06278	21281.58977	25713.73326	
Tajikistan	1945.507858	1360.540678	2786.543183	1730.168266	1330.116159	2252.757074	
Tanzania, Unite	3997.846602	2636.655011	6066.276569	3548.342705	2304.798578	5466.698394	
Thailand	24318.14236	23518.89535	25146.38362	19502.3487	18865.5965	20160.89364	
The Netherland	7144.447066	6808.668578	7496.611212	5135.522714	4917.088693	5363.438472	
The Republic of	486.1688183	257.9869393	1102.258375	477.7495939	228.9535377	1295.665717	
Timor-Leste	375.6817209	313.4187466	448.5101486	336.9701719	288.5308022	392.6128196	
Togo	1034.464451	384.1785449	2842.22222	911.3875485	399.1271674	2137.344751	
Trinidad and To	326.524893	208.526534	515.8729376	265.2581642	193.7478565	365.3465708	
Tunisia	6562.814866	5890.90215	7346.485566	5302.627714	4791.970842	5890.484123	
Türkiye	78895.16748	75612.56829	82462.88137	71139.98274	68165.13822	74248.66292	

Turkmenistan	1742.126681	771.0763825	3984.319821	1606.415133	751.0551443	3490.67897
Uganda	5887.30034	4365.16508	7946.824414	5004.176417	3701.584387	6753.364619
Ukraine	7490.934486	7271.544381	7717.972748	5868.341939	5701.415887	6040.738443
United Arab Em	3570.007835	2603.309746	4928.242831	3135.098312	2205.788275	4455.078054
United Kingdor	24459.81407	23995.92269	24932.40514	14685.29644	14248.36526	15135.48614
United States o	81004.35178	79771.09699	82256.76828	43014.28573	42307.45214	43732.8903
Uruguay	1581.708058	1350.97997	1856.625813	1273.14438	1104.976733	1469.77361
Uzbekistan	8758.073421	7537.005609	10189.86329	7542.84545	6822.175422	8346.776214
Vanuatu	74.76513106	45.74264872	124.2914734	73.25307133	40.78012824	121.8365197
Venezuela	13341.36619	11935.74512	14914.99559	11706.94781	10832.16966	12650.5369
Viet Nam	77915.45256	75365.88032	80556.15064	68385.39478	65913.34932	70952.28318
Yemen	4115.786064	3441.49762	4918.706458	4004.753673	3333.101197	4810.736646
Zambia	2329.155565	1584.542275	3425.192984	2110.63203	1419.532853	3140.606781
Zimbabwe	2633.720649	1988.734367	3490.316631	2490.183974	1823.401063	3406.629336
**Supplementary H**						
	**DALY PER CASE**			**MIR**		
COUNTRY	vlaue	lower_UI	Upper_UI	vlaue	lower_UI	Upper_UI
Afghanistan	28.11419248	12.53670093	62.65372895	1.007022186	0.457719184	2.21E+00
Albania	23.09218427	15.54223737	34.46885697	0.996896162	0.66845439	1.460331686
Algeria	25.18763409	11.0549264	55.72757034	0.952029419	0.426987715	2.117802801
Angola	29.15676217	12.80984988	66.90461229	0.973298372	0.41758501	2.244716564
Argentina	22.77925798	19.24311077	26.86424833	1.006014389	0.845311445	1.183186984
Armenia	23.94927117	19.92140535	29.1355577	1.00151667	0.829617835	1.213296675
Australia	13.50537855	11.17096386	16.28381655	0.685484427	0.554189693	0.832616941
Austria	18.42564466	15.70231692	21.7485943	0.867122067	0.72831517	1.02836233
Azerbaijan	27.95254505	13.96835829	55.19471473	0.959093723	0.483437437	1.914725828
Bahamas	25.47922349	19.9191802	32.44945339	0.947453697	0.7465612	1.207231709
Bahrain	26.24464779	14.27024313	47.24223083	0.899689541	0.493849641	1.628922946
Bangladesh	28.48007199	14.65458389	55.09123445	0.956201707	0.494387569	1.846455971
Barbados	21.86746661	16.36027957	29.30276448	0.956478433	0.710911667	1.27153091
Belarus	22.37040211	17.77242782	27.9442823	0.862309308	0.692184341	1.07505611
Belgium	18.63025563	15.64551549	22.39909626	0.901366824	0.739461667	1.091158889
Belize	25.94418153	19.99513561	33.8538798	0.956093129	0.737399983	1.241666518
Benin	28.85165022	15.62550812	52.60836349	0.977105584	0.533023796	1.794665002
Bhutan	28.94667264	14.74138837	57.33746044	0.965358795	0.492872128	1.874341714
Bolivia (Plurina	24.83306904	14.42273531	43.43780559	0.967576704	0.560216344	1.67843577

Bosnia Herzegc	23.61558774	16.69795678	33.86728162	0.991506388	0.699622462	1.397378118	
Botswana	28.17471516	15.30671104	52.00060011	0.976634709	0.54072421	1.749942762	
Brazil	23.42441404	20.83601845	26.44513813	0.998968315	0.878255325	1.130979682	
Brunei Darussa	24.01866238	15.68559959	36.34634975	0.940883641	0.619547639	1.423169024	
Bulgaria	24.20503103	20.67655878	28.51776508	0.975788466	0.839796263	1.14074579	
Burkina Faso	26.52659819	14.7718277	47.75184361	1.020308752	0.561297653	1.849392647	
Burundi	31.66493249	13.45285282	76.29495744	0.94660691	0.387362213	2.291256972	
Cambodia	27.17344041	15.21040142	49.29856439	0.999177387	0.545992813	1.823670337	
Cameroon	28.66669665	15.30841367	54.60695929	0.978221641	0.518196404	1.84736727	
Canada	15.56649748	12.88761989	18.90489367	0.798309071	0.65020008	0.97336795	
Cape Verde	25.79320258	14.25180518	47.410513	1.020940574	0.564742377	1.830559533	
Central African	28.92567672	10.56768584	80.30735504	0.983484114	0.353399333	2.719703493	
Chad	28.33236366	15.41266908	52.56454593	0.992211629	0.529971214	1.835370308	
Chile	20.73838267	18.01487073	24.09378301	0.981263882	0.83940778	1.145996979	
China	19.89622838	14.65888286	27.58933466	0.878180864	0.653766624	1.200798852	
Colombia	20.56027351	17.95713169	23.24998671	0.92430991	0.797319614	1.050145479	
Comoros	27.18240766	12.93251003	56.66812579	1.016880553	0.483896838	2.122616565	
Congo, Democratic Republic of		0.802583433	5.090239922	0.981261801	0.355035181	2.672254725	
Congo, Republic of		162.7915985	1033.997965	0.985364944	0.422311923	2.28870532	
Costa Rica	19.93864577	16.05993952	24.8381782	0.892010665	0.711860514	1.110036772	
Côte d'Ivoire	31.01431061	17.70520363	54.84370597	0.946095955	0.541230489	1.656225869	
Croatia	21.66985261	18.66823094	24.85588916	0.969152979	0.831422613	1.11259056	
Cuba	20.25413934	16.57563803	25.38095784	0.922486459	0.755317857	1.153834598	
Cyprus	18.96982486	11.57815549	30.74009984	0.898824527	0.535796091	1.469457773	
Czechia	18.94762271	16.74425931	21.51000117	0.896850798	0.790107204	1.021478441	
Denmark	17.04105387	14.82090843	19.79050869	0.888524636	0.75952606	1.034855412	
Djibouti	30.74653425	14.56258236	65.87515476	0.947731796	0.44668825	2.02473244	
Dominican Rep	24.31337494	16.49012786	35.92172616	1.007840098	0.680781291	1.508639202	
Ecuador	21.63374761	18.26602937	25.77932641	0.93219346	0.786046285	1.115715872	
Egypt	26.93214407	15.4214437	46.38565423	0.955292241	0.544289733	1.654250944	
El Salvador	22.35479623	14.43091683	34.37598491	0.960741208	0.632821703	1.474001562	
Equatorial Guir	29.62949428	12.29966111	70.34990029	0.959195806	0.401633354	2.301533286	
Eritrea	31.66369968	15.71319254	63.65435522	0.953625569	0.473757735	1.929589785	
Estonia	18.26234351	15.51127852	21.65666489	0.864132944	0.727694006	1.031578926	
Eswatini	30.78955331	16.57712398	55.89493557	0.953537823	0.532124873	1.693264851	
Ethiopia	29.40495467	16.22500343	53.38741507	0.978487005	0.545139368	1.781650985	

Fiji	26.53007686	15.42450743	45.42468128	0.996430264	0.585327438	1.695959551	
Finland	15.45498781	12.70993192	19.2432826	0.806091106	0.63866469	1.008192919	
France (metrop	16.6608235	13.90221335	20.02675726	0.750516833	0.621430762	0.90063823	
France, Guadel	N/A	N/A	N/A	N/A	N/A	N/A	
France, La Réur	N/A	N/A	N/A	N/A	N/A	N/A	
France, Martini	N/A	N/A	N/A	N/A	N/A	N/A	
French Guyana	N/A	N/A	N/A	N/A	N/A	N/A	
French Polynes	N/A	N/A	N/A	N/A	N/A	N/A	
Gabon	27.23367608	12.19503213	61.97753881	0.995685948	0.43511838	2.25974127	
Gaza Strip and	25.61932158	16.32503829	40.33568644	0.944109356	0.605566029	1.480087619	
Georgia	24.93751113	18.8404484	32.85069573	0.992772094	0.751675282	1.311197029	
Germany	17.45118309	15.36713969	20.23827409	0.829365595	0.710158795	0.966415481	
Ghana	28.49637891	16.10372976	51.59216734	0.98054232	0.542129198	1.770678172	
Greece	19.1810856	16.46184105	22.51071427	0.931660384	0.793114541	1.094349295	
Guam	24.69935195	19.40727846	30.9876086	0.974863224	0.770053021	1.222117178	
Guatemala	24.59756465	18.58774367	32.37832617	0.979282285	0.745272372	1.291450018	
Guinea	28.3644585	15.95299028	50.64347979	0.989199671	0.55617754	1.754675855	
Guinea-Bissau	29.26099586	16.67110775	51.03621661	0.978738925	0.556645444	1.72133209	
Guyana	27.02185356	19.33970363	37.66889392	0.96850945	0.694292802	1.350259462	
Haiti	28.38050818	15.47791254	52.38703838	0.977516695	0.531430782	1.774363249	
Honduras	24.6162584	14.79769229	41.21578665	1.010430552	0.600606997	1.68643759	
Hungary	22.56367588	20.27892793	25.10077781	0.987503404	0.886561788	1.098623425	
Iceland	16.62518318	13.09973882	21.27619787	0.786910982	0.618953272	1.019501021	
India	27.38569266	17.93956758	42.35168776	0.976067392	0.629496065	1.508331294	
Indonesia	28.66663018	15.9934851	51.55931151	0.960153465	0.537695169	1.714783007	
Iran, Islamic Re	24.3614864	16.43483151	36.04328729	0.932009513	0.633186534	1.371185133	
Iraq	26.86437228	16.5269425	44.09937129	0.933916502	0.56396118	1.555066141	
Ireland	17.25424906	14.30614401	21.48553479	0.838515933	0.679621259	1.045993861	
Israel	19.2000748	15.60737093	24.1731044	0.911924969	0.725306899	1.144785341	
Italy	17.4022579	14.51276223	21.41218792	0.909097682	0.742596694	1.122160489	
Jamaica	24.26407259	17.78759992	32.98146147	0.966811864	0.711177225	1.316364709	
Japan	11.87638801	9.42934245	15.17850719	0.749755172	0.574952918	0.978712483	
Jordan	25.22185023	17.44565267	36.38165508	0.917906611	0.626171573	1.340899903	
Kazakhstan	26.40317472	22.39150914	30.92169682	0.965313053	0.819985306	1.12815144	
Kenya	29.57333577	17.08489042	51.1272208	0.959360821	0.547247683	1.676696878	
Korea, Democr	25.13956522	13.78797668	46.15194864	0.974996716	0.536888427	1.756662938	

Korea, Republic	13.86072678	10.19394269	19.02856542	0.744309794	0.539676167	1.024836345	
Kuwait	23.89479221	17.47483739	32.17332969	0.802773931	0.592531094	1.081647729	
Kyrgyzstan	26.84924422	21.25342669	34.01943932	0.968927005	0.76910053	1.231788505	
Lao People's De	28.08304574	16.46679779	47.4164895	0.98458816	0.583930063	1.663396793	
Latvia	21.40178351	18.46536754	25.16106926	0.923161978	0.799769456	1.070615081	
Lebanon	23.31982916	15.93877767	34.51929774	0.941396016	0.638942244	1.409134503	
Lesotho	27.28880666	12.43671686	61.30941002	1.001302265	0.447844883	2.206151875	
Liberia	30.09514394	15.54029418	58.3842582	0.960323484	0.484940988	1.895805002	
Libya	27.52752896	12.49175909	59.26717002	0.941913084	0.425706506	2.056575791	
Lithuania	20.93335217	18.09626772	24.77428535	0.909727098	0.784089229	1.069531882	
Luxembourg	18.45865714	14.93363596	22.82243144	0.863109085	0.696815184	1.073013468	
Madagascar	30.03378685	14.20344113	63.53032131	0.962590521	0.447774279	2.057247329	
Malawi	31.18868129	17.04756258	56.39336976	0.946367748	0.530780933	1.694249191	
Malaysia	24.82071508	17.82185963	34.66777604	0.998350443	0.719983909	1.387226388	
Maldives	22.56337093	13.87779034	36.6318426	1.002494485	0.615173038	1.638066166	
Mali	28.87037874	16.91679134	49.54159015	0.977860319	0.574329227	1.668370869	
Malta	19.02094733	14.64669309	24.66845715	0.922217852	0.701905433	1.204712087	
Mauritania	27.13092011	14.67004356	50.55131886	0.98972619	0.535099341	1.830417279	
Mauritius	24.06607453	19.31793789	29.91382426	0.996854656	0.79930502	1.242620019	
Mexico	22.67904067	19.58070337	26.37270367	0.974198799	0.838559278	1.13020983	
Mongolia	28.06831762	16.20755281	49.3809379	0.96866053	0.568444622	1.630225483	
Montenegro	23.14684668	15.22936973	34.8273677	0.964623004	0.639179246	1.44513715	
Morocco	25.57911006	8.449506139	76.51908024	0.983752033	0.337129694	2.907646511	
Mozambique	27.58582836	13.80272905	55.20297843	1.014854639	0.517379467	2.001401307	
Myanmar	26.74491221	14.75432955	47.91412954	0.99776217	0.550270634	1.806371678	
Namibia	30.79353533	17.49000911	54.13155671	0.944814624	0.546466538	1.638767827	
Nepal		0.03293059	0.105033075	0.983472335	0.539559887	1.789832426	
New Caledonia	N/A	N/A	N/A	N/A	N/A	N/A	
New Zealand	16.04299075	13.43447989	19.21722655	0.765313525	0.627258965	0.925252829	
Nicaragua	23.70504117	16.27562882	35.69543519	0.92412902	0.631243515	1.348634936	
Niger	27.88779087	14.34828156	53.90055025	0.992114764	0.513242242	1.912660496	
Nigeria	28.47086798	14.50013555	55.86777277	0.97870772	0.497498278	1.90E+00	
North Macedon	23.21993367	17.22039062	30.99385455	0.956232648	0.700623812	1.276932481	
Norway	15.45344732	12.53637463	19.39608698	0.759433466	0.611680922	0.956317478	
Oman	27.12220696	14.4056452	48.80996959	0.909384984	0.496010848	1.62922291	
Pakistan	28.71423369	15.68281394	52.46722255	0.975599657	0.536071449	1.760092121	

Panama	20.3889368	16.01758625	25.52548329	0.923877068	0.726106174	1.160449825	
Papua New Gui	27.91585789	12.89634525	59.64123204	0.981604144	0.455526125	2.128400331	
Paraguay	24.35883403	15.99122197	37.11544887	1.00927581	0.657775894	1.547368018	
Peru	19.96488272	14.21154385	28.16889824	0.861538444	0.605258984	1.216043188	
Philippines	27.0431417	18.75678751	39.14480557	0.987976288	0.693485462	1.412047626	
Poland	22.77488583	20.51843184	25.37522379	1.042677121	0.935519693	1.155511063	
Portugal	19.23079206	15.95732419	23.18082962	0.880087625	0.717201841	1.064491996	
Puerto Rico	17.50170974	14.37723764	21.55066273	0.888475481	0.725851139	1.096389822	
Qatar	26.71338859	12.31674557	57.35271527	0.815227887	0.378594338	1.763472699	
Republic of Mo	24.3591277	20.38290801	29.34871799	0.928477685	0.781811155	1.11148573	
Romania	24.42277816	20.59165008	29.01424183	0.982992939	0.831287568	1.163251895	
Russian Federa	22.3054821	19.44735246	25.87265197	0.899171066	0.782261308	1.041424459	
Rwanda	29.10033938	13.13280754	64.24764593	0.967253693	0.431086601	2.160445791	
Saint Lucia	24.46588081	18.01003093	33.24323744	0.962234393	0.720013157	1.287053412	
Samoa	26.86585551	9.751339816	73.64443309	0.982471234	0.355075085	2.724098418	
Sao Tome and I	27.40931837	14.6410811	51.69978063	0.99300483	0.534752538	1.84264997	
Saudi Arabia	26.99700946	14.20561894	52.14507043	0.91777994	0.492230986	1.728436902	
Senegal	27.22137637	14.72831183	51.24748313	1.013295892	0.545433816	1.868070253	
Serbia	22.69279292	16.63519682	30.42565812	0.942285742	0.696477975	1.273081067	
Sierra Leone	29.65472486	15.95280863	54.9589723	0.975585452	0.523724403	1.826376478	
Singapore	14.80184221	9.279206891	24.27769972	0.734188055	0.449496302	1.203876539	
Slovakia	23.00329251	17.18096314	31.38933748	0.990370739	0.73907929	1.30168608	
Slovenia	19.22723134	16.19086545	23.02395476	0.897562173	0.750743594	1.073563559	
Solomon Island	29.21513931	17.25263649	49.98238365	0.961913604	0.561687117	1.646841318	
Somalia	29.32274797	13.02139614	66.18147426	0.986863402	0.435729267	2.224940992	
South Africa	27.16450604	19.94913504	37.80785249	0.982761053	0.722077324	1.329569296	
South Sudan	30.9812641	14.07097759	68.63755747	0.955548933	0.433130281	2.104269867	
Spain	17.23262585	14.41348571	20.95394172	0.798048989	0.657052625	0.974036981	
Sri Lanka	24.11345999	14.84158324	38.2641361	0.970447677	0.618924121	1.548501596	
Sudan	26.27406147	11.60493128	56.98393553	0.993948087	0.449028207	2.190686445	
Suriname	25.37540499	16.21046657	39.46058235	0.995240015	0.630735016	1.557469559	
Sweden	18.04627969	15.2798684	21.55505985	1.002560624	0.827306037	1.2055844	
Switzerland	16.80256214	13.56218393	20.81464805	0.842014611	0.666885288	1.046970675	
Syrian Arab Rep	25.14727238	14.68642421	42.885638	0.959778669	0.563693974	1.626991603	
Tajikistan	27.9631762	14.65060315	53.62522547	0.970567742	0.502948565	1.869394813	
Tanzania, Unite	27.72142152	15.41629217	50.09831716	0.992423956	0.541619831	1.803015921	

Thailand	23.90425679	14.39088129	39.38119292	0.986677011	0.606512862	1.610712038	
The Netherland	18.03293164	15.67329492	20.8053202	0.902168968	0.766725823	1.047450574	
The Republic of	27.58617493	13.88213811	54.82811023	0.996708268	0.495402334	1.985415043	
Timor-Leste	26.53640944	12.87148308	55.37407171	1.007337315	0.489199189	2.089680293	
Togo	29.1245178	15.94143051	52.53989215	0.969131354	0.533639855	1.753922452	
Trinidad and To	24.11551957	18.80438183	31.04952034	0.96499253	0.755178199	1.24039884	
Tunisia	23.64949561	14.85742187	37.56437078	0.939400128	0.589262848	1.498546587	
Türkiye	23.48664177	13.37250505	41.20661688	0.949237751	0.543034076	1.656097583	
Turkmenistan	29.04816465	22.19678248	38.15343604	0.954300441	0.73220386	1.248849689	
Uganda	31.12252983	17.95226608	53.90879205	0.948232691	0.557549859	1.624231437	
Ukraine	22.65927285	17.14442455	30.13376462	0.847469548	0.644183052	1.126776909	
United Arab Em	27.77421094	14.6641267	52.85526965	0.923032535	0.483002421	1.763535719	
United Kingdor	15.05703071	13.24017257	17.2375193	0.795912555	0.68449707	0.920198725	
United States o	16.72912707	14.07538666	20.18321594	0.813572355	0.674024459	0.978109282	
Uruguay	22.90132803	19.6551761	26.81172773	0.977395408	0.835546589	1.144124675	
Uzbekistan	27.7641732	23.75735094	32.27364887	0.961655973	0.823622459	1.124955957	
Vanuatu	28.34046089	13.59371705	59.00040921	0.984423715	0.472822157	2.038213655	
Venezuela	23.7928168	16.28974613	35.0422883	0.9648772	0.664378363	1.411195168	
Viet Nam	26.83653531	15.05129588	47.16993529	0.937276607	0.532908288	1.616090051	
Yemen	26.91530225	12.42241182	57.32434097	0.991966687	0.463726788	2.119179443	
Zambia	31.57849621	18.08289736	54.5991206	0.938349835	0.543443532	1.621822011	
Zimbabwe	26.90924763	15.30174107	47.18583327	1.00866927	0.570120347	1.773353875	

