# Supplementary material for: Global lung cancer burden, trends, and projections from 2010 to 2050: A population-level severity framework integrating DALYs-per-case and mortality-to-incidence ratio
Source: PLoS One. 2026 Jul 23;21(7):e0354350. doi: 10.1371/journal.pone.0354350 (PMC13395349; doi:10.1371/journal.pone.0354350)
Supplement: S2 File — (PDF) [file pone.0354350.s002.pdf]

Country	Raw DALYs	DALYs per Case	MIR	Quadrant (Raw DALYs)
Afghanista	19854.346	28.11419248	1.007022186	Q2: Low Burden / High MIR
Albania	24626.901	23.09218427	0.996896162	Q2: Low Burden / High MIR
Algeria	82518.628	25.18763409	0.952029419	Q4: High Burden / Low MIR
Angola	35057.332	29.15676217	0.973298372	Q1: High Burden / High MIF
Argentina	283559.48	22.77925798	1.006014389	Q1: High Burden / High MIF
Armenia	25131.642	23.94927117	1.00151667	Q2: Low Burden / High MIR
Australia	197566.06	13.50537855	0.685484427	Q4: High Burden / Low MIR
Austria	99037.979	18.42564466	0.867122067	Q4: High Burden / Low MIR
Azerbaijan	51409.377	27.95254505	0.959093723	Q4: High Burden / Low MIR
Bahamas	1744.0354	25.47922349	0.947453697	Q3: Low Burden / Low MIR
Bahrain	6288.3198	26.24464779	0.899689541	Q3: Low Burden / Low MIR
Bangladesł	513889.69	28.48007199	0.956201707	Q4: High Burden / Low MIR
Barbados	1163.8746	21.86746661	0.956478433	Q3: Low Burden / Low MIR
Belarus	97847.71	22.37040211	0.862309308	Q4: High Burden / Low MIR
Belgium	150874.73	18.63025563	0.901366824	Q4: High Burden / Low MIR
Belize	984.97538	25.94418153	0.956093129	Q3: Low Burden / Low MIR
Benin	3002.5976	28.85165022	0.977105584	Q2: Low Burden / High MIR
Bhutan	1399.1239	28.94667264	0.965358795	Q2: Low Burden / High MIR
Bolivia (Plu	36191.984	24.83306904	0.967576704	Q1: High Burden / High MIF
Bosnia Her	53241.922	23.61558774	0.991506388	Q1: High Burden / High MIF
Botswana	2728.7691	28.17471516	0.976634709	Q2: Low Burden / High MIR
Brazil	921636.03	23.42441404	0.998968315	Q1: High Burden / High MIF
Brunei Dar	2513.9731	24.01866238	0.940883641	Q3: Low Burden / Low MIR
Bulgaria	91494.625	24.20503103	0.975788466	Q1: High Burden / High MIF
Burkina Fa:	13055.73	26.52659819	1.020308752	Q2: Low Burden / High MIR
Burundi	9949.1773	31.66493249	0.94660691	Q3: Low Burden / Low MIR
Cambodia	46869.396	27.17344041	0.999177387	Q1: High Burden / High MIF
Cameroon	29612.521	28.66669665	0.978221641	Q1: High Burden / High MIF
Canada	466980.38	15.56649748	0.798309071	Q4: High Burden / Low MIR
Cape Verd	1566.489	25.79320258	1.020940574	Q2: Low Burden / High MIR
Central Afr	4150.5965	28.92567672	0.983484114	Q2: Low Burden / High MIR
Chad	11294.885	28.33236366	0.992211629	Q2: Low Burden / High MIR
Chile	85381.781	20.73838267	0.981263882	Q1: High Burden / High MIF
China	17358732	19.89622838	0.878180864	Q4: High Burden / Low MIR
Colombia	124691.2	20.56027351	0.92430991	Q4: High Burden / Low MIR
Comoros	441.01986	27.18240766	1.016880553	Q2: Low Burden / High MIR
Costa Rica	11326.021	19.93864577	0.892010665	Q3: Low Burden / Low MIR
Croatia	64924.926	21.66985261	0.969152979	Q1: High Burden / High MIF
Cuba	122075.38	20.25413934	0.922486459	Q4: High Burden / Low MIR
Cyprus	10375.231	18.96982486	0.898824527	Q3: Low Burden / Low MIR
Czechia	119280.68	18.94762271	0.896850798	Q4: High Burden / Low MIR
Côte d'Ivoi	19922.669	31.01431061	0.946095955	Q3: Low Burden / Low MIR
Denmark	90084.065	17.04105387	0.888524636	Q4: High Burden / Low MIR
Djibouti	2018.2264	30.74653425	0.947731796	Q3: Low Burden / Low MIR
Dominican	42602.02	24.31337494	1.007840098	Q1: High Burden / High MIF
Ecuador	26161.211	21.63374761	0.93219346	Q3: Low Burden / Low MIR
Egypt	338244.7	26.93214407	0.955292241	Q4: High Burden / Low MIR
El Salvador	17977.877	22.35479623	0.960741208	Q3: Low Burden / Low MIR
Equatorial	2698.1452	29.62949428	0.959195806	Q3: Low Burden / Low MIR

Eritrea	6800.4339	31.66369968	0.953625569	Q3: Low Burden / Low MIR
Estonia	13106.574	18.26234351	0.864132944	Q3: Low Burden / Low MIR
Eswatini	830.6971	30.78955331	0.953537823	Q3: Low Burden / Low MIR
Ethiopia	144310.63	29.40495467	0.978487005	Q1: High Burden / High MIF
Fiji	2634.966	26.53007686	0.996430264	Q2: Low Burden / High MIR
Finland	56462.392	15.45498781	0.806091106	Q4: High Burden / Low MIR
France (me	963127.55	16.6608235	0.750516833	Q4: High Burden / Low MIR
Gabon	3725.3234	27.23367608	0.995685948	Q2: Low Burden / High MIR
Gaza Strip	13704.762	25.61932158	0.944109356	Q3: Low Burden / Low MIR
Georgia	36426.233	24.93751113	0.992772094	Q1: High Burden / High MIF
Germany	1195279.7	17.45118309	0.829365595	Q4: High Burden / Low MIR
Ghana	40767.991	28.49637891	0.98054232	Q1: High Burden / High MIF
Greece	174075.03	19.1810856	0.931660384	Q4: High Burden / Low MIR
Guam	1501.7424	24.69935195	0.974863224	Q2: Low Burden / High MIR
Guatemala	16379.21	24.59756465	0.979282285	Q2: Low Burden / High MIR
Guinea	10867.89	28.3644585	0.989199671	Q2: Low Burden / High MIR
Guinea-Bis	1347.8711	29.26099586	0.978738925	Q2: Low Burden / High MIR
Guyana	1886.7346	27.02185356	0.96850945	Q2: Low Burden / High MIR
Haiti	21617.277	28.38050818	0.977516695	Q2: Low Burden / High MIR
Honduras	20451.82	24.6162584	1.010430552	Q2: Low Burden / High MIR
Hungary	185456.64	22.56367588	0.987503404	Q1: High Burden / High MIF
Iceland	3713.9183	16.62518318	0.786910982	Q3: Low Burden / Low MIR
India	2841336.3	27.38569266	0.976067392	Q1: High Burden / High MIF
Indonesia	1402361.2	28.66663018	0.960153465	Q4: High Burden / Low MIR
Iran, Islami	233627.29	24.3614864	0.932009513	Q4: High Burden / Low MIR
Iraq	197375.95	26.86437228	0.933916502	Q4: High Burden / Low MIR
Ireland	52056.837	17.25424906	0.838515933	Q4: High Burden / Low MIR
Israel	55173.204	19.2000748	0.911924969	Q4: High Burden / Low MIR
Italy	686519.17	17.4022579	0.909097682	Q4: High Burden / Low MIR
Jamaica	14321.249	24.26407259	0.966811864	Q2: Low Burden / High MIR
Japan	1600307.6	11.87638801	0.749755172	Q4: High Burden / Low MIR
Jordan	40386.145	25.22185023	0.917906611	Q4: High Burden / Low MIR
Kazakhstar	82340.923	26.40317472	0.965313053	Q1: High Burden / High MIF
Kenya	30108.603	29.57333577	0.959360821	Q4: High Burden / Low MIR
Korea, Der	225798	25.13956522	0.974996716	Q1: High Burden / High MIF
Korea, Rep	464119.98	13.86072678	0.744309794	Q4: High Burden / Low MIR
Kuwait	5668.3553	23.89479221	0.802773931	Q3: Low Burden / Low MIR
Kyrgyzstan	15279.605	26.84924422	0.968927005	Q2: Low Burden / High MIR
Lao People	30669.124	28.08304574	0.98458816	Q1: High Burden / High MIF
Latvia	22350.268	21.40178351	0.923161978	Q3: Low Burden / Low MIR
Lebanon	42473.842	23.31982916	0.941396016	Q4: High Burden / Low MIR
Lesotho	4264.9281	27.28880666	1.001302265	Q2: Low Burden / High MIR
Liberia	5507.7687	30.09514394	0.960323484	Q3: Low Burden / Low MIR
Libya	30944.401	27.52752896	0.941913084	Q4: High Burden / Low MIR
Lithuania	28221.096	20.93335217	0.909727098	Q3: Low Burden / Low MIR
Luxembour	5674.2836	18.45865714	0.863109085	Q3: Low Burden / Low MIR
Madagasca	31952.58	30.03378685	0.962590521	Q1: High Burden / High MIF
Malawi	9540.0628	31.18868129	0.946367748	Q3: Low Burden / Low MIR
Malaysia	161606.55	24.82071508	0.998350443	Q1: High Burden / High MIF
Maldives	1117.7547	22.56337093	1.002494485	Q2: Low Burden / High MIR

Mali	17605.464	28.87037874	0.977860319	Q2: Low Burden / High MIR
Malta	3844.3481	19.02094733	0.922217852	Q3: Low Burden / Low MIR
Mauritania	6450.5483	27.13092011	0.98972619	Q2: Low Burden / High MIR
Mauritius	4553.6646	24.06607453	0.996854656	Q2: Low Burden / High MIR
Mexico	194745.35	22.67904067	0.974198799	Q1: High Burden / High MIF
Mongolia	17083.625	28.06831762	0.96866053	Q2: Low Burden / High MIR
Montenegı	11233.547	23.14684668	0.964623004	Q2: Low Burden / High MIR
Morocco	141870.64	25.57911006	0.983752033	Q1: High Burden / High MIF
Mozambiq	11814.711	27.58582836	1.014854639	Q2: Low Burden / High MIR
Myanmar	266680.57	26.74491221	0.99776217	Q1: High Burden / High MIF
Namibia	2539.6489	30.79353533	0.944814624	Q3: Low Burden / Low MIR
New Zealaı	47597.968	16.04299075	0.765313525	Q4: High Burden / Low MIR
Nicaragua	8064.7343	23.70504117	0.92412902	Q3: Low Burden / Low MIR
Niger	4475.3324	27.88779087	0.992114764	Q2: Low Burden / High MIR
Nigeria	195061.92	28.47086798	0.97870772	Q1: High Burden / High MIF
North Mac	25675.712	23.21993367	0.956232648	Q3: Low Burden / Low MIR
Norway	49628.7	15.45344732	0.759433466	Q4: High Burden / Low MIR
Oman	5637.0489	27.12220696	0.909384984	Q3: Low Burden / Low MIR
Pakistan	319707.97	28.71423369	0.975599657	Q1: High Burden / High MIF
Panama	7694.4534	20.3889368	0.923877068	Q3: Low Burden / Low MIR
Papua New	38079.262	27.91585789	0.981604144	Q1: High Burden / High MIF
Paraguay	22124.136	24.35883403	1.00927581	Q2: Low Burden / High MIR
Peru	93831.972	19.96488272	0.861538444	Q4: High Burden / Low MIR
Philippines	309326.08	27.0431417	0.987976288	Q1: High Burden / High MIF
Poland	613986.05	22.77488583	1.042677121	Q1: High Burden / High MIF
Portugal	110957.7	19.23079206	0.880087625	Q4: High Burden / Low MIR
Puerto Ric	15529.686	17.50170974	0.888475481	Q3: Low Burden / Low MIR
Qatar	2616.418	26.71338859	0.815227887	Q3: Low Burden / Low MIR
Republic o	27057.498	24.3591277	0.928477685	Q3: Low Burden / Low MIR
Romania	259332.07	24.42277816	0.982992939	Q1: High Burden / High MIF
Russian Fe	1310068	22.3054821	0.899171066	Q4: High Burden / Low MIR
Rwanda	23276.164	29.10033938	0.967253693	Q2: Low Burden / High MIR
Saint Lucia	572.23856	24.46588081	0.962234393	Q2: Low Burden / High MIR
Samoa	414.10164	26.86585551	0.982471234	Q2: Low Burden / High MIR
Sao Tome a	583.65663	27.40931837	0.99300483	Q2: Low Burden / High MIR
Saudi Arab	48030.79	26.99700946	0.91777994	Q4: High Burden / Low MIR
Senegal	10194.514	27.22137637	1.013295892	Q2: Low Burden / High MIR
Serbia	165513.95	22.69279292	0.942285742	Q4: High Burden / Low MIR
Sierra Leor	6574.348	29.65472486	0.975585452	Q2: Low Burden / High MIR
Singapore	26774.168	14.80184221	0.734188055	Q3: Low Burden / Low MIR
Slovakia	59233.034	23.00329251	0.990370739	Q1: High Burden / High MIF
Slovenia	27377.63	19.22723134	0.897562173	Q3: Low Burden / Low MIR
Solomon Is	5858.6182	29.21513931	0.961913604	Q3: Low Burden / Low MIR
Somalia	7036.3114	29.32274797	0.986863402	Q2: Low Burden / High MIR
South Afric	299019.67	27.16450604	0.982761053	Q1: High Burden / High MIF
South Suda	12229.195	30.9812641	0.955548933	Q3: Low Burden / Low MIR
Spain	557637.66	17.23262585	0.798048989	Q4: High Burden / Low MIR
Sri Lanka	105508.62	24.11345999	0.970447677	Q1: High Burden / High MIF
Sudan	72870.819	26.27406147	0.993948087	Q1: High Burden / High MIF
Suriname	2205.1353	25.37540499	0.995240015	Q2: Low Burden / High MIR

Sweden	78529.979	18.04627969	1.002560624	Q1: High Burden / High MIF
Switzerlan(	77688.464	16.80256214	0.842014611	Q4: High Burden / Low MIR
Syrian Aral	47541.272	25.14727238	0.959778669	Q4: High Burden / Low MIR
Tajikistan	16064.855	27.9631762	0.970567742	Q2: Low Burden / High MIR
Tanzania, U	16864.636	27.72142152	0.992423956	Q2: Low Burden / High MIR
Thailand	665647.2	23.90425679	0.986677011	Q1: High Burden / High MIF
The Nethe	253521.9	18.03293164	0.902168968	Q4: High Burden / Low MIR
The Repub	789.1136	27.58617493	0.996708268	Q2: Low Burden / High MIR
Timor-Lest	3637.2677	26.53640944	1.007337315	Q2: Low Burden / High MIR
Togo	8256.8739	29.1245178	0.969131354	Q2: Low Burden / High MIR
Trinidad ar	5336.9964	24.11551957	0.96499253	Q2: Low Burden / High MIR
Tunisia	117033.06	23.64949561	0.939400128	Q4: High Burden / Low MIR
Turkmenis	11869.777	29.04816465	0.954300441	Q3: Low Burden / Low MIR
Türkiye	834335.05	23.48664177	0.949237751	Q4: High Burden / Low MIR
Uganda	43194.786	31.12252983	0.948232691	Q4: High Burden / Low MIR
Ukraine	339780.77	22.65927285	0.847469548	Q4: High Burden / Low MIR
United Ara	13759.058	27.77421094	0.923032535	Q3: Low Burden / Low MIR
United Kin	800861.15	15.05703071	0.795912555	Q4: High Burden / Low MIR
United Sta	3286071.5	16.72912707	0.813572355	Q4: High Burden / Low MIR
Uruguay	44140.612	22.90132803	0.977395408	Q1: High Burden / High MIF
Uzbekistan	63322.557	27.7641732	0.961655973	Q4: High Burden / Low MIR
Vanuatu	528.13656	28.34046089	0.984423715	Q2: Low Burden / High MIR
Venezuela	137996.88	23.7928168	0.9648772	Q1: High Burden / High MIF
Viet Nam	730104.33	26.83653531	0.937276607	Q4: High Burden / Low MIR
Yemen	41509.847	26.91530225	0.991966687	Q1: High Burden / High MIF
Zambia	9390.6921	31.57849621	0.938349835	Q3: Low Burden / Low MIR
Zimbabwe	30305.577	26.90924763	1.00866927	Q1: High Burden / High MIF

Quadrant (DALYs per Case)	Reclassified
Q1: High Burden / High MIR	Yes
Q2: Low Burden / High MIR	No
Q4: High Burden / Low MIR	No
Q1: High Burden / High MIR	No
Q2: Low Burden / High MIR	Yes
Q2: Low Burden / High MIR	No
Q3: Low Burden / Low MIR	Yes
Q3: Low Burden / Low MIR	Yes
Q4: High Burden / Low MIR	No
Q4: High Burden / Low MIR	Yes
Q4: High Burden / Low MIR	Yes
Q4: High Burden / Low MIR	No
Q3: Low Burden / Low MIR	No
Q3: Low Burden / Low MIR	Yes
Q3: Low Burden / Low MIR	Yes
Q4: High Burden / Low MIR	Yes
Q1: High Burden / High MIR	Yes
Q1: High Burden / High MIR	Yes
Q2: Low Burden / High MIR	Yes
Q2: Low Burden / High MIR	Yes
Q1: High Burden / High MIR	Yes
Q2: Low Burden / High MIR	Yes
Q3: Low Burden / Low MIR	No
Q2: Low Burden / High MIR	Yes
Q1: High Burden / High MIR	Yes
Q4: High Burden / Low MIR	Yes
Q1: High Burden / High MIR	No
Q1: High Burden / High MIR	No
Q3: Low Burden / Low MIR	Yes
Q1: High Burden / High MIR	Yes
Q1: High Burden / High MIR	Yes
Q1: High Burden / High MIR	Yes
Q2: Low Burden / High MIR	Yes
Q3: Low Burden / Low MIR	Yes
Q3: Low Burden / Low MIR	Yes
Q1: High Burden / High MIR	Yes
Q3: Low Burden / Low MIR	No
Q2: Low Burden / High MIR	Yes
Q3: Low Burden / Low MIR	Yes
Q3: Low Burden / Low MIR	No
Q3: Low Burden / Low MIR	Yes
Q4: High Burden / Low MIR	Yes
Q3: Low Burden / Low MIR	Yes
Q4: High Burden / Low MIR	Yes
Q2: Low Burden / High MIR	Yes
Q3: Low Burden / Low MIR	No
Q4: High Burden / Low MIR	No
Q3: Low Burden / Low MIR	No
Q4: High Burden / Low MIR	Yes

Q4: High Burden / Low MIR	Yes
Q3: Low Burden / Low MIR	No
Q4: High Burden / Low MIR	Yes
Q1: High Burden / High MIR	No
Q1: High Burden / High MIR	Yes
Q3: Low Burden / Low MIR	Yes
Q3: Low Burden / Low MIR	Yes
Q1: High Burden / High MIR	Yes
Q4: High Burden / Low MIR	Yes
Q2: Low Burden / High MIR	Yes
Q3: Low Burden / Low MIR	Yes
Q1: High Burden / High MIR	No
Q3: Low Burden / Low MIR	Yes
Q2: Low Burden / High MIR	No
Q2: Low Burden / High MIR	No
Q1: High Burden / High MIR	Yes
Q1: High Burden / High MIR	Yes
Q1: High Burden / High MIR	Yes
Q1: High Burden / High MIR	Yes
Q2: Low Burden / High MIR	No
Q2: Low Burden / High MIR	Yes
Q3: Low Burden / Low MIR	No
Q1: High Burden / High MIR	No
Q4: High Burden / Low MIR	No
Q3: Low Burden / Low MIR	Yes
Q4: High Burden / Low MIR	No
Q3: Low Burden / Low MIR	Yes
Q3: Low Burden / Low MIR	Yes
Q3: Low Burden / Low MIR	Yes
Q2: Low Burden / High MIR	No
Q3: Low Burden / Low MIR	Yes
Q4: High Burden / Low MIR	No
Q1: High Burden / High MIR	No
Q4: High Burden / Low MIR	No
Q2: Low Burden / High MIR	Yes
Q3: Low Burden / Low MIR	Yes
Q3: Low Burden / Low MIR	No
Q1: High Burden / High MIR	Yes
Q1: High Burden / High MIR	No
Q3: Low Burden / Low MIR	No
Q3: Low Burden / Low MIR	Yes
Q1: High Burden / High MIR	Yes
Q4: High Burden / Low MIR	Yes
Q4: High Burden / Low MIR	No
Q3: Low Burden / Low MIR	No
Q3: Low Burden / Low MIR	No
Q1: High Burden / High MIR	No
Q4: High Burden / Low MIR	Yes
Q2: Low Burden / High MIR	Yes
Q2: Low Burden / High MIR	No

Q1: High Burden / High MIR	Yes
Q3: Low Burden / Low MIR	No
Q1: High Burden / High MIR	Yes
Q2: Low Burden / High MIR	No
Q2: Low Burden / High MIR	Yes
Q1: High Burden / High MIR	Yes
Q2: Low Burden / High MIR	No
Q1: High Burden / High MIR	No
Q1: High Burden / High MIR	Yes
Q1: High Burden / High MIR	No
Q4: High Burden / Low MIR	Yes
Q3: Low Burden / Low MIR	Yes
Q3: Low Burden / Low MIR	No
Q1: High Burden / High MIR	Yes
Q1: High Burden / High MIR	No
Q3: Low Burden / Low MIR	No
Q3: Low Burden / Low MIR	Yes
Q4: High Burden / Low MIR	Yes
Q1: High Burden / High MIR	No
Q3: Low Burden / Low MIR	No
Q1: High Burden / High MIR	No
Q2: Low Burden / High MIR	No
Q3: Low Burden / Low MIR	Yes
Q1: High Burden / High MIR	No
Q2: Low Burden / High MIR	Yes
Q3: Low Burden / Low MIR	Yes
Q3: Low Burden / Low MIR	No
Q4: High Burden / Low MIR	Yes
Q3: Low Burden / Low MIR	No
Q2: Low Burden / High MIR	Yes
Q3: Low Burden / Low MIR	Yes
Q1: High Burden / High MIR	Yes
Q2: Low Burden / High MIR	No
Q1: High Burden / High MIR	Yes
Q1: High Burden / High MIR	Yes
Q4: High Burden / Low MIR	No
Q1: High Burden / High MIR	Yes
Q3: Low Burden / Low MIR	Yes
Q1: High Burden / High MIR	Yes
Q3: Low Burden / Low MIR	No
Q2: Low Burden / High MIR	Yes
Q3: Low Burden / Low MIR	No
Q1: High Burden / High MIR	Yes
Q1: High Burden / High MIR	Yes
Q1: High Burden / High MIR	No
Q4: High Burden / Low MIR	Yes
Q3: Low Burden / Low MIR	Yes
Q2: Low Burden / High MIR	Yes
Q1: High Burden / High MIR	No
Q1: High Burden / High MIR	Yes

Q2: Low Burden / High MIR	Yes
Q3: Low Burden / Low MIR	Yes
Q4: High Burden / Low MIR	No
Q1: High Burden / High MIR	Yes
Q1: High Burden / High MIR	Yes
Q2: Low Burden / High MIR	Yes
Q3: Low Burden / Low MIR	Yes
Q1: High Burden / High MIR	Yes
Q1: High Burden / High MIR	Yes
Q1: High Burden / High MIR	Yes
Q2: Low Burden / High MIR	No
Q3: Low Burden / Low MIR	Yes
Q4: High Burden / Low MIR	Yes
Q3: Low Burden / Low MIR	Yes
Q4: High Burden / Low MIR	No
Q3: Low Burden / Low MIR	Yes
Q4: High Burden / Low MIR	Yes
Q3: Low Burden / Low MIR	Yes
Q3: Low Burden / Low MIR	Yes
Q2: Low Burden / High MIR	Yes
Q4: High Burden / Low MIR	No
Q1: High Burden / High MIR	Yes
Q2: Low Burden / High MIR	Yes
Q4: High Burden / Low MIR	No
Q1: High Burden / High MIR	No
Q4: High Burden / Low MIR	Yes
Q1: High Burden / High MIR	No
