# Supplementary figures and images for: Global lung cancer burden, trends, and projections from 2010 to 2050: A population-level severity framework integrating DALYs-per-case and mortality-to-incidence ratio
Source: PLoS One. 2026 Jul 23;21(7):e0354350. doi: 10.1371/journal.pone.0354350 (PMC13395349; doi:10.1371/journal.pone.0354350)

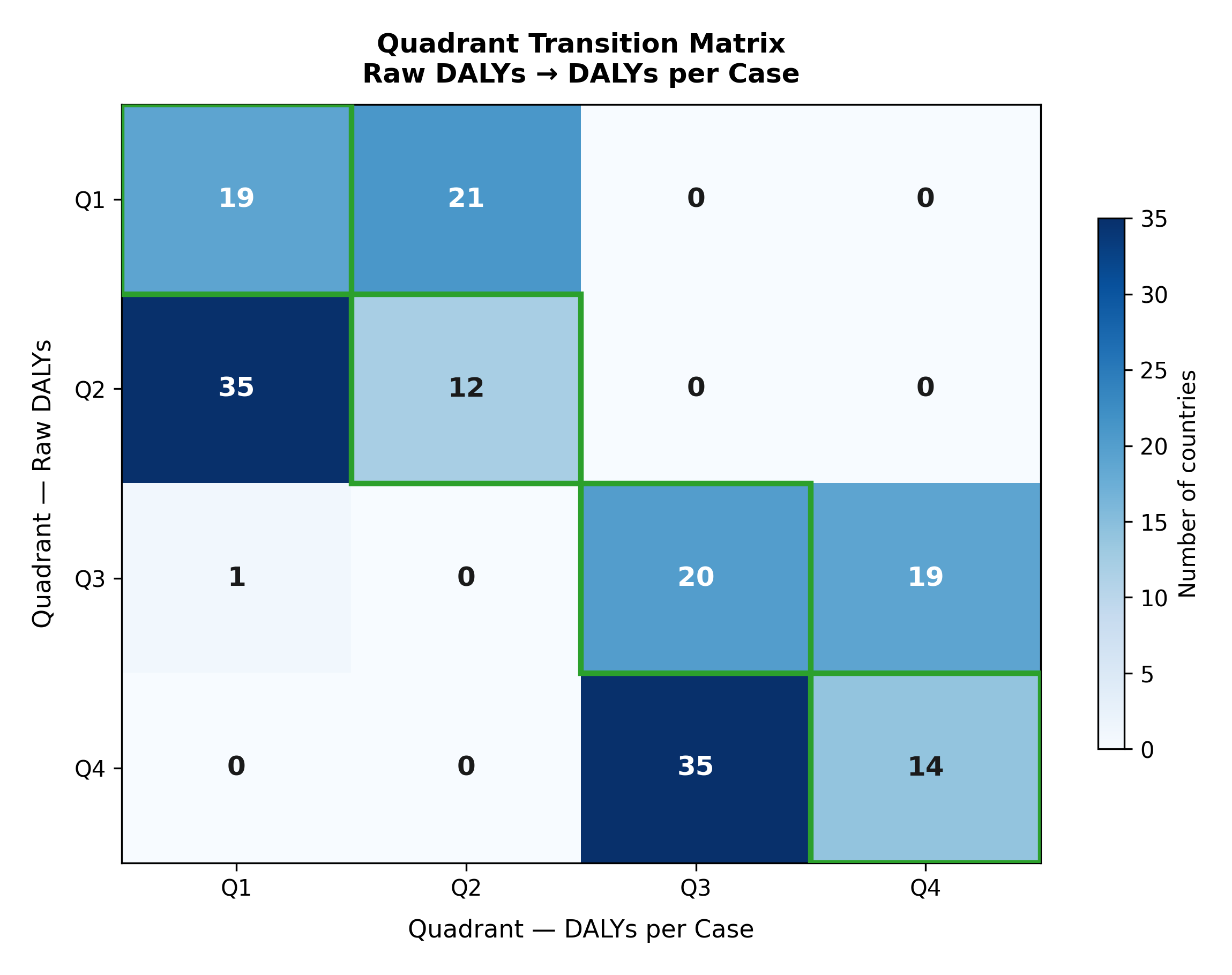

Supplement: S1 Fig — (PNG) [file pone.0354350.s003.png]

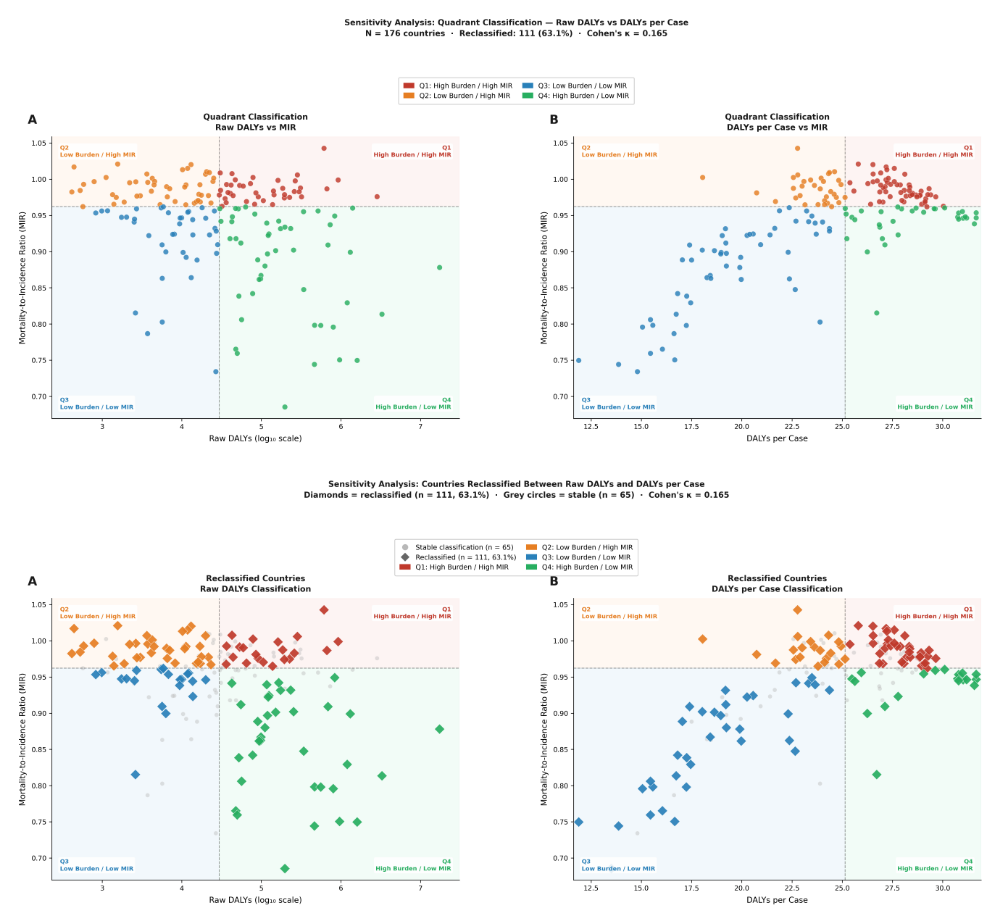

Supplement: S2 Fig — (PNG) [file pone.0354350.s004.png]
